# Cobalt(III)-catalyzed asymmetric ring-opening of 7-oxabenzonorbornadienes via indole C–H functionalization

**DOI:** 10.1038/s41467-023-36723-6

**Published:** 2023-02-25

**Authors:** Yang Zheng, Wen-Yun Zhang, Qing Gu, Chao Zheng, Shu-Li You

**Affiliations:** State Key Laboratory of Organometallic Chemistry, Shanghai Institute of Organic Chemistry, Chinese Academy of Sciences, 345 Lingling Lu, Shanghai, 200032 China

**Keywords:** Synthetic chemistry methodology, Asymmetric catalysis

## Abstract

Asymmetric ring-opening of 7-oxabenzonorbornadienes is achieved via Co-catalyzed indole C–H functionalization. The utilization of chiral Co-catalyst consisting of a binaphthyl-derived trisubstituted cyclopentadienyl ligand resulted in high yields (up to 99%) and excellent enantioselectivity (>99% ee) for the target products with tolerance for diverse functional groups. Opposite diastereoselectivities are obtained with chiral Co-catalyst or Cp*CoI_2_CO. Combined experimental and computational studies suggest *β*-oxygen elimination being the selectivity-determining step of the reaction. Meanwhile, the reactions of 7-azabenzonorbornadiene could also be executed in a diastereodivergent manner.

## Abstract (web summary)

Asymmetric ring-opening (ARO) reactions, which can help quickly build molecular complexity, have not been extensively shown via β-oxygen elimination. Here, the authors report a cobalt(III)-catalyzed ARO reaction, providing mechanistic insights and the origins of the diastereo- and enantioselectivity.

## Introduction

**Fig. 1:**
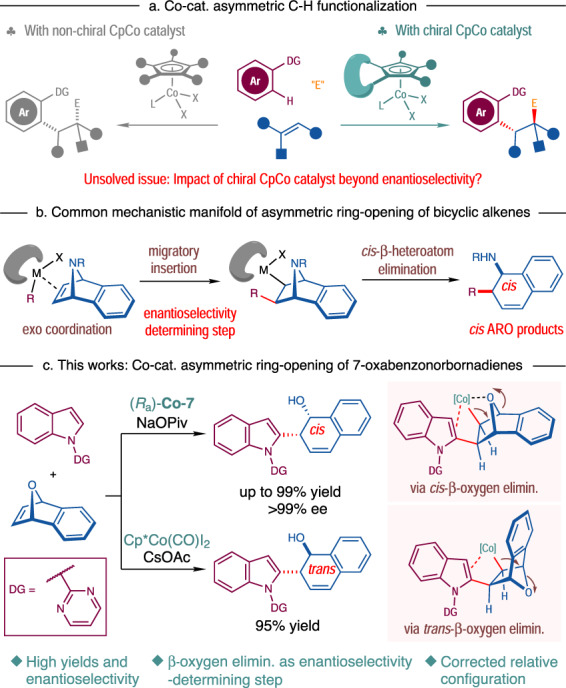
Asymmetric ring-opening of bicyclic olefins via Co-catalyzed C–H functionalization. **a** Co catalyzed asymmetric C–H functionalization. **b** Common mechanistic manifold of asymmetric ring-opening of bicyclic alkenes. **c** Co-catalyzed asymmetric ring-opening of 7-oxabenzonorbornadienes (this work).

Tremendous developments have been achieved in the past decades for transition-metal-catalyzed C–H functionalization reactions^1–8^. Recently, exploring 3d metal complexes in C–H functionalization has attracted considerable attention because of the earth-abundance and low toxicity of these metals compared with the 4d and 5d counterparts^9–12^. Pioneered by the groups of Matsunaga and Kanai^13,14^, Ackermann^15^, Ellman^16^, Glorius^17^, and Chang^18^, cyclopentadienyl (Cp)-Co complexes have been identified as competent catalysts for various C–H functionalization reactions, which often exhibit complementary reactivity and selectivity to those with Cp-Rh catalysts^14,18–26^. Particularly, the Cp-Co complexes derived from a chiral Cp ligand^27–29^ or achiral Cp ligand in combination with chiral counteranions^30–37^ were demonstrated suitable for asymmetric C–H functionalization reactions (Fig. 1a)^12^. In this regard, the groups of Ackermann^30^, Matsunaga^31–34^, Cramer^27–29^, and Shi^35–37^ independently reported Co-catalyzed asymmetric aryl C–H functionalization with unactivated olefins, maleimides or dioxazolones. Previously, the modifications of the Cp ring have resulted in an extensive array of Cp-metal catalysts^38–44^. However, whether the use of chiral Cp ligands in Co-catalyzed C–H functionalization reactions can influence the reaction outcomes of beyond enantioselectivity remains underdeveloped^13,27–29,45,46^.

Asymmetric ring-opening (ARO) of strained bicyclic olefins is an important platform for building-up molecular complexity (Fig. 1b)^47–49^. Merging ARO reactions with transition-metal-catalyzed C–H activation has become an area of recent interest^50–60^. In this regard, the groups of Zheng and Li^53^, and Cramer^54^ independently reported the ARO reactions of 7-azabenzonorbornadienes and other aza-bicyclic olefins under Rh-catalysis, in which *β-*nitrogen elimination was a key step. However, to the best of our knowledge, the C–H activation-induced ARO reactions of 7-oxabenzonorbornadienes via *β-*oxygen elimination remained elusive, probably due to the competitive dehydration^61,62^ or direct reductive elimination^63,64^ as a side reaction pathway. Mechanistically, it was regarded that the ARO reactions proceeded via the *exo*-coordination of a carbon–metal species to the olefin moiety, migratory insertion and *cis*-*β*-heteroatom elimination^47,53–60^. And the migratory insertion was speculated as the enantioselectivity-determining step in the ARO reactions (Fig. 1b)^47,53,54^. In line with our continuous interest in developing transition-metal-catalyzed asymmetric C–H functionalization reactions^65–71^, we envisioned that asymmetric indole C–H functionalization with 7-oxabenzonorbornadienes might be accomplished by using a chiral Cp-Co catalyst^56^ (Fig. 1c).

In this work, high yields and excellent stereoselectivity were obtained for a wide range of *cis-*ring-opening products under the optimal conditions. More interestingly, the utilization of Cp*Co(CO)I_2_ as the catalyst led to reversed diastereoselectivities favoring the *trans*-ring-opening products. The misassigned relative configuration of the products in the previous literature was corrected accordingly^56^. Combined experimental and computational studies suggested the mechanistic pictures of the CpCo-catalyzed ARO reactions where *cis*- and *trans*-*β*-oxygen elimination work as the selectivity-determining step for enantioselective and racemic reactions, respectively. Such results are different from the previous cognition^47,53–64^. As an extension, 7-azabenzonorbornadiene could also be applied in the similar ARO reactions in a diastereodivergent manner. Herein, we report the results from this study.

## Results

### Reaction development

Our study commenced with the optimization of the reaction conditions using *N*-pyrimidinylindole (**1a**, 0.05 mmol) and 7-oxabenzonorbornadiene (**2a**, 1.5 equiv) as model substrates (Table 1). When achiral catalyst Cp*CoI_2_CO (**Co-1**, 5 mol%) and CsOAc (30 mol%) were utilized, the reaction proceeded smoothly in trifluoroethanol (TFE, 0.3 mL) at 50 °C, leading to the designed ring-opening product **3aa** as a mixture of *cis* and *trans* isomers (10:90 dr) in a combined yield of 95% (entry 1). Variating the substituent of the Cp ring of the catalyst (**Co-2** and **Co-3**, R^1^ = H and Cy) did not provide better yields or ratio of *cis*-**3aa**/*trans*-**3aa** (entries 2 and 3). On the contrary, when the chiral Co-complexes consisting of a chiral binaphthyl-derived Cp ligand [(*R*_a_)-**Co-4**–(*R*_a_)-**Co-7**] were used, the diastereoselectivity of the reaction was reversed, with *cis*-**3aa** being the major product (entries 4–7). As the substituent of the Cp ring became bulkier (R^2^ = H, Me, *i*-Pr and *t*-Bu), both the ratio of *cis*-**3aa**/*trans*-**3aa** (80:20–95:5 dr) and the ee of *cis*-**3aa** (69 to >99%) increased steadily albeit with moderate combined yield of **3aa** (10–25%). It was found that the reaction efficiency could be significantly influenced by the additive and solvent. Replacing CsOAc to CsOPiv, NaOPiv or HOPiv resulted in the elevated combined yields of *cis-***3aa** (39–60%), high diastereoselectivity (up to 96:4 dr) and superior enantioselectivity (>99% ee), with NaOPiv as the best choice among tested (entries 8–10). Switching the solvent to MeOH, DCM or toluene totally shutdown the reaction. However, the utilization of hexafluoroisopropanol (HFIP) led to nearly quantitative formation of *cis*-**3aa** (98% yield) but with slightly decreased dr (93:7) and ee (98%) (entry 11). The stereochemical control could be restored to excellent level by employing mixed solvents of TFE and HFIP (entries 12 and 13). Overall, the optimal reaction outcomes (98% yield, 95:5 dr and >99% ee for *cis*-**3aa**) were obtained when the ratio of TFE and HFIP (v/v) was set to 3:1 (entry 13). In addition, the reactions between **1a** and **2a** were conducted under the (*R*_a_)-**Rh-1** catalysis (entry 14, also see the Supplementary Information for details)^53^, but both *cis*- and *trans*-**3aa** were not detected, while **1a** and **2a** were recovered. These results demonstrated that the Co-catalysts exhibit unique reactivity for the ARO reaction of 7-oxabenzonorbornadiene.

**Table 1:** Optimization of reaction conditions^a^

entry	[Co]	solvent	additive	yield (%)^b^	dr (*cis*-3aa/*trans*-3aa)^c^	ee of *cis*-3aa (%)^c^
1	**Co-1**	TFE	CsOAc	95	10:90	–
2	**Co-2**	TFE	CsOAc	67	43:57	–
3	**Co-3**	TFE	CsOAc	94	18:82	–
4^d^	(*R*_a_)-**Co-4**	TFE	CsOAc	10	80:20	69
5	(*R*_a_)-**Co-5**	TFE	CsOAc	12	90:10	>99
6	(*R*_a_)-**Co-6**	TFE	CsOAc	19	93:7	>99
7	(*R*_a_)-**Co-7**	TFE	CsOAc	25	95:5	>99
8	(*R*_a_)-**Co-7**	TFE	CsOPiv	39	96:4	>99
9	(*R*_a_)-**Co-7**	TFE	NaOPiv	60	96:4	>99
10	(*R*_a_)-**Co-7**	TFE	HOPiv	59	92:8	>99
11	(*R*_a_)-**Co-7**	HFIP	NaOPiv	98	93:7	98
12	(*R*_a_)-**Co-7**	TFE/HFIP (v/v = 1:1)	NaOPiv	98	94:6	99
13	(*R*_a_)-**Co-7**	TFE/HFIP (v/v = 3:1)^e^	NaOPiv	98	95:5	>99
14	(*R*_a_)-**Rh-1**	TFE/HFIP (v/v = 3:1)^e^	NaOPiv	ND	–	–

^a^Reaction conditions: **1a** (0.05 mmol), **2a** (0.075 mmol), **[Co]** (5 mol%), additive (30 mol%) in solvent (0.3 mL) at 50 °C for 18 h.

^b^Combined yield of *cis*-**3aa** and *trans*-**3aa**.

^c^The ratio of *cis*-**3aa**/*trans*-**3aa** and the ee value of *cis*-**3aa** were determined by HPLC with a chiral stationary phase.

^d^At 80 °C for 10 h.

^e^TFE (0.3 mL) and HFIP (0.1 mL) were used as the solvent. ND = not detected.

**Fig. 2:**
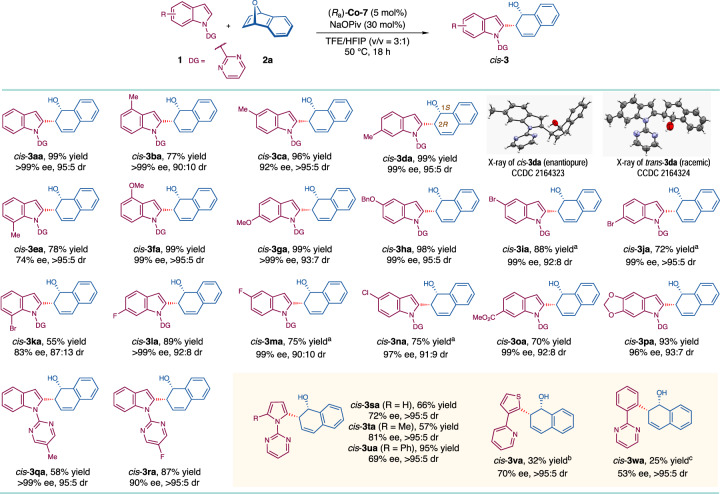
Substrate scope: Indoles and other arenes. Reaction conditions: **1** (0.10 mmol) **2a** (0.15 mmol), (*R*_a_)-**Co-7** (5 mol%), NaOPiv (30 mol%) in TFE/HFIP (0.4 mL, v/v = 3:1) at 50 °C for 18 h. Note: (a) In TFE/HFIP/DCM (0.5 mL, v/v/v = 3:1:1) at 50 °C for 18 h. (b) (*R*_a_)-**Co-7** (10 mol%), Zn(OAc)_2_ (30 mol%) instead of NaOPiv (30 mol%) was used, in TFE/HFIP (0.4 mL, v/v = 3:1) at 60 °C for 24 h. (c) (*R*_a_)-**Co-7** (10 mol%), Zn(OAc)_2_ (30 mol%) instead of NaOPiv (30 mol%) was used, in TFE/HFIP (0.4 mL, v/v = 3:1) at 70 °C for 24 h.

With the optimal conditions in hands, the scope of substituted indoles was next investigated (Fig. 2). Indoles bearing a methyl group at the C4-, C5- or C6-position were well tolerated. The corresponding products *cis*-**3ba**–**3da** were afforded in high yields (77–99%) with good to excellent diastereoselectivity and excellent enantioselectivity (90:10 to >95:5 dr and 92 to >99% ee). However, the reaction of C7-methyl substituted indole became sluggish where the yield and ee value of *cis*-**3ea** were decreased to 78% and 74%, respectively. The influences of other electron-donating groups including methoxy and benzyloxy at the C4-, C5- or C6-position were also investigated. The desired products *cis*-**3fa**–**3** **ha** were all obtained efficiently (98–99% yields, 93:7 to >95:5 dr, 99 to >99% ee). Furthermore, a variety of electron-deficient indoles bearing halogen substituents (fluoride, chloride and bromide) or an ester group (CO_2_Me) at these positions were well tolerated. In general, good yields (70–88%) and excellent stereochemical control (90:10 to >95:5 dr and 97 to >99% ee) were achieved for the formation of *cis*-**3ia**, **3ja**, **3la**–**3oa**. Notably, introducing a bromide group at the C7-position of the indole ring largely retarded the reaction, leading to *cis*-**3ka** in moderate yield (55%) with lowered stereoselectivity (87:13 dr and 83% ee). The reaction could further accommodate [1,3]dioxolo-substituted indole substrate and variations of the electronic properties of the *N*-pyrimidinyl directing group (*cis*-**3pa**–**3ra**, 58–93% yields, 93:7 to >95:5 dr and 90 to >99% ee). Pyrrole derivatives also well participated in the reaction and the C2-substituents (methyl or phenyl) did not lead to deleterious effects on the reaction outcomes (*cis*-**3sa**–**3ua**, 57–95% yields, >95:5 dr and 69–81% ee). Moreover, thiophene and benzene derivatives can also be applied in the reactions with 7-oxabenzonorbornadiene, albeit with diminished yields of *cis*-**3va** and *cis*-**3wa** (25–32% yields, >95:5 dr and 53–70% ee) mainly due to recovered substrates **1v** and **1w**. Samples of *cis*-**3da** (enantiopure) and *trans*-**3da** (racemic) were subjected to X-ray crystallographic analyses. Their structures, relative configurations, and the absolute configuration of *cis*-**3da** (1*S*,2*R*) were assigned unambiguously.

**Fig. 3:**
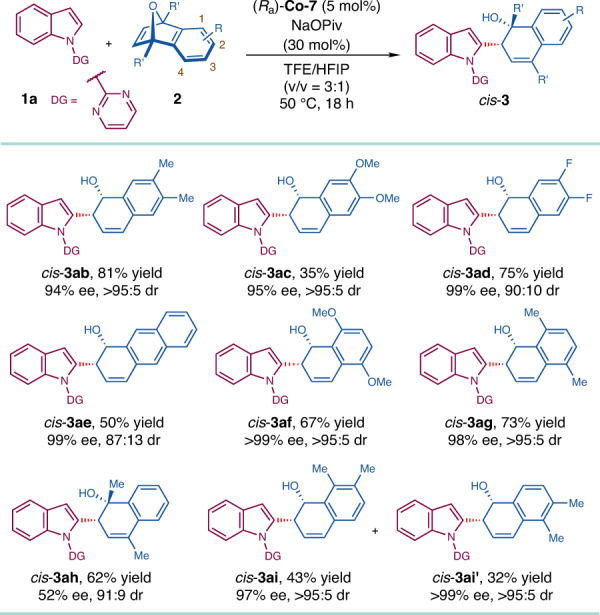
Substrate scope: 7-Oxabenzonorbornadienes. Reaction conditions: **1a** (0.10 mmol), **2** (0.15 mmol), (*R*_a_)-**Co-7** (5 mol%), NaOPiv (30 mol%) in TFE/HFIP (0.4 mL, v/v = 3:1) at 50 °C for 18 h.

The reactions of various 7-oxabenzonorbornadiene-derivatives were also considered (Fig. 3). Symmetrically disubstituted 7-oxabenzonorbornadienes (2,3-dimethyl, 2,3-dimethoxy, 2,3-difluoro, 2,3-benzo, 1,4-dimethoxy and 1,4-dimethyl) participated in the desired reactions smoothly, giving rise to *cis*-**3ab**, **3ad**–**3ag** in reasonable yields (50–81%) with good to excellent stereoselectivity (87:13 to >95:5 dr and 94 to >99% ee). To be noted, *cis*-**3ac** was afforded in only 35% yield, but with >95:5 dr and 95% ee. It was postulated that the electron-rich nature of *cis*-**3ac** caused the side reaction of dehydration^61^. Methyl-substituent at two bridge-head positions led to moderate yield (62%) and enantiopurity (52% ee) of *cis*-**3ah**. The reaction of unsymmetrically disubstituted (1,2-dimethyl) substrate generated a pair of regioisomers *cis*-**3ai** (43% yield) and *cis*-**3ai′** (32% yield), both of which were obtained with excellent diastereo- and enantioselectivity (up to >95:5 dr and >99% ee).

### Mechanistic studies

**Fig. 4:**
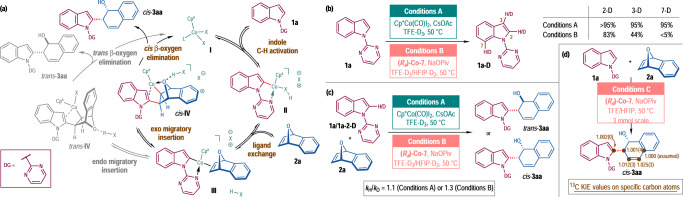
Proposed catalytic cycle and deuterium-labeling and kinetic isotope effect experiments. **a** Proposed catalytic cycle. **b** Deuterium-labeling reactions of **1a**. **c** H/D Kinetic isotope effect (KIE) experiments. (**d**) ^12^C/^13^C KIE experiments.

In order to understand the origins for the opposite diastereoselectivity with **Co-1** (Conditions A) or (*R*_a_)-**Co-7** (Conditions B) and for the enantioselectivity in the latter case, a series of mechanistic studies was performed. In a simplified catalytic cycle (Fig. 4a), the reaction starts with the C–H activation of indole substrate **1a** by a Cp-Co complex **I** (X = OAc or OPiv, L = CO or solvent), which leads to a cyclometallated Co complex **II**. Deuterium-labeling reactions of **1a** were conducted under both conditions using TFE-D_3_ or mixed solvents of TFE-D_3_ and HFIP-D_2_ (v/v = 3:1) (Fig. 4b). High deuteration ratio at the C2 position of the indole ring was observed in both cases (>95% for Conditions A and 83% for Conditions B), which implied the indole C–H activation step should be reversible. Meanwhile, H/D kinetic isotope effect (KIE) experiments using **1a** and **1a-2-D** revealed small *k*_H_/*k*_D_ values of 1.1 for Conditions A and 1.3 for Conditions B, respectively (Fig. 4c). These results suggested that the indole C–H activation was not involved in the turn-over limiting step of the catalytic cycle. Next, Co complex **II** undergoes ligand exchange with 7-oxabenzonorbornadiene **2a**, which generates an olefin-coordinated Co complex **III**. Starting from this intermediate, there exist two competing reaction pathways, *exo*- or *endo*-migratory insertion, leading to *cis*-**IV** and *trans*-**IV**, respectively. The subsequent C–O bond-cleavage proceeds via the *cis*- or *trans*-*β*-oxygen elimination, which could be assisted by acetic acid generated in the C–H activation step. The catalytic cycle is finally closed by releasing the products *cis*-**3aa** or *trans*-**3aa** with the concomitant regeneration of Co complex **I**. In addition, ^12^C/^13^C KIE at natural ^13^C abundance was determined by quantitative ^13^C NMR experiments for the reaction of **1a** with **2a** catalyzed by (*R*_a_)-**Co-7** (3 mmol scale)^72,73^. Significant ^12^C/^13^C KIE values were observed for both carbon atoms [1.025(3) and 1.012(3)] of the newly formed C=C double bond of *cis*-**3aa**, suggesting the *β*-oxygen elimination being the rate-determining step (Fig. 4d).

**Fig. 5:**
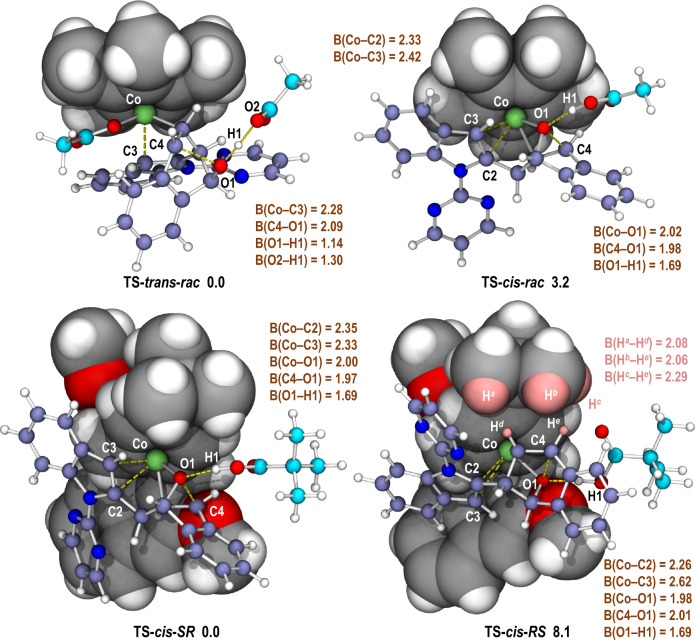
Optimized structures of the transition states for the *β*-oxygen elimination step. Calculated at the PWPB95-D3(BJ)/def2-TZVPP (SMD, TFE)//B3LYP-D3(BJ)/def2-SVP (gas) level of theory. The relative Gibbs free energies (Δ*G*_sol_) are in kcal/mol. The distances of forming/cleaving bonds are in Å.

DFT calculations were performed for the reactions between **1a** and **2a** with either **Co-1** or (*R*_a_)-**Co-7** as the catalyst (Fig. 5, also see the Supplementary Information and Supplementary Data 1 for details). The calculated energy profiles indicated that the *β*-oxygen elimination was the rate- and stereoselectivity-determining step in both cases, and thus all the precedent steps were reversible. In the reaction promoted by **Co-1**, the energy of the transition state for *trans*-*β*-elimination **TS-*trans*-*rac*** was lower than that for the *cis*-*β*-elimination transition state **TS-*cis*-*rac*** by 3.2 kcal/mol, which qualitatively reproduced the preferential formation of *trans*-**3a** over *cis*-**3a**. It should be noted that the *cis*-*β*-elimination relied on a temporary Co–O coordination bond [B(Co–O) = 2.02 Å] in a cationic transition state **TS-*cis*-*rac***, while in **TS-*trans*-*rac*** such interaction did not exist and the oxygen atom was located in the opposite side of the original 7-oxabenzonorbornadiene ring. Thus, an additional acetate anion was allowed to coordinate to the Co center in **TS-*trans*-*rac***, forging a highly charge-separate but overall neutral transition state. The variance on the coordination mode of the Co center in **TS-*cis*-*rac*** and **TS-*trans*-*rac*** was believed to contribute to the calculated energy difference between these two transition states. The situation was changed significantly when (*R*_a_)-**Co-7** was employed as the catalyst. The steric congestion around the Co center could not accommodate the coordination of an additional anion to the Co center as in **TS-*trans*-*rac***, which raised the energetic barrier of the *trans*-*β*-oxygen elimination, and thus made the *cis*-*β*-oxygen elimination the dominant reaction pathway. In order to shed light on the origin of the asymmetric induction with (*R*_a_)-**Co-7**, the key transition states of *cis*-*β*-oxygen elimination leading to (1*S*,2*R*)-**3aa** (**TS-*cis*-*SR***, 0.0 kcal/mol) and (1*R*,2*S*)-**3aa** (**TS-*cis*-*RS***, 8.1 kcal/mol), respectively, were located. The large energy difference between these two transition states was in well accordance with the exceedingly high enantioselectivity (>99% ee) observed in the most cases experimentally. The specific substrate orientation in **TS-*cis*-*RS*** induced severe destabilizing structural features including (1) weak coordination of the C2=C3 bond of the indole ring to the Co center [B(Co–C2) = 2.26 Å and B(Co–C3) = 2.62 Å] resulted from the geometric distortion of the substrates compared with that in **TS-*cis*-*SR*** [B(Co–C2) = 2.35 Å and B(Co–C3) = 2.33 Å], and (2) steric repulsion between the substrates and the *tert*-butyl substituent on the cyclopentadienyl ring in **TS-*cis*-*RS***, which was exemplified by the short distances between several pairs of hydrogen atoms in the two structural components [B(H^*a*^–H^*d*^) = 2.08 Å, B(H^*b*^–H^*e*^) = 2.06 Å and B(H^*c*^–H^*e*^) = 2.29 Å]. To be noted, some minor effects which cause diminished enantioselectivity for the substrates with certain substitution patterns might not be included in the computational model.

### Synthetic Applications

**Fig. 6:**
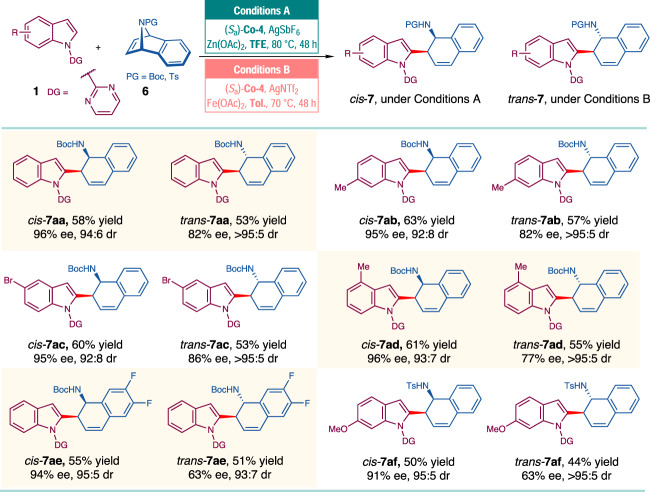
ARO reactions of 7-azabenzonorbornadiene. Reaction conditions A: **1** (0.15 mmol), **6** (0.10 mmol), (*S*_*a*_)-**Co-4** (10 mol%), AgSbF_6_ (20 mol%), Zn(OAc)_2_ (50 mol %) in TFE (1.0 mL) at 80 °C for 48 h. Reaction conditions B: **1** (0.15 mmol), **6** (0.10 mmol), (*S*_*a*_)-**Co-4** (10 mol%), AgNTf_2_ (20 mol%), Fe(OAc)_2_ (50 mol%) in toluene (1.0 mL) at 70 °C for 48 h.

As an extension, *N*-Boc 7-azabenzonorbornadiene **6** was also investigated in this chiral Cp-Co catalyzed ARO reactions (Fig. 6, see the Supplementary Information for details). In the presence of (*S*_a_)-**Co-4** as the catalyst, AgSbF_6_, Zn(OAc)_2_, as the additives, and TFE as the solvent, the *cis*-ring-opening products (*cis*-**7aa**–**7ae**) were delivered in morderate yields (55-63%) with excellent stereoselectivity (92:8 to 95:5 dr, 94–96% ee). More interestingly, when the solvent was changed to toluene, the ring-opening products with the opposite relative configuration (*trans*-**7aa**–**7ae**) were obtained in promising results (51–57% yields, 93:7 to >95:5 dr, 63–86% ee). Besides, the reactions of *N*-tosyl 7-azabenzonorbornadiene could also be executed in a diastereodivergent manner, giving the *cis*-**7af**/*trans*-**7af** in morderate yields (44–50%) with moderate to good stereoselectivity (95:5 to >95:5 dr, 63–91% ee). Unfortunatly, when 7-oxabenzonorbornadiene **2a** was subjected to the conditions for the synthesis of *trans*-**7**, only the dehydration product was delivered in almost quantitative yield^61,62^.

**Fig. 7:**
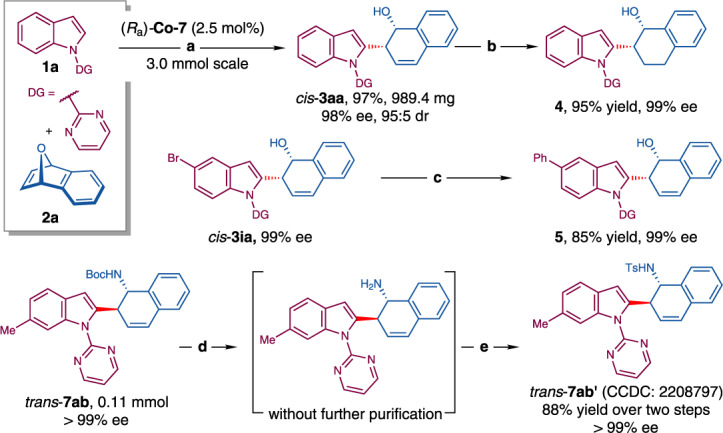
Gram-scale reaction and product transformations. Reaction conditions: (a) **1a** (3.0 mmol), **2a** (4.5 mmol), NaOPiv · H_2_O (0.9 mmol), (*R*_a_)-**Co-7** (0.075 mmol), TFE/HFIP (12.0 mL, v/v = 3:1), 50 °C, 48 h. (b) Pd/C (0.1 equiv), H_2_ (1 atm), EtOAc (0.1 M), rt., 24 h. (c) PhB(OH)_2_ (2.0 equiv), Pd_2_dba_3_ (2.5 mol%), SPhos (10 mol%), Na_2_CO_3_ (0.5 equiv), toluene/EtOH/H_2_O (1.25 mL, v/v/v = 3:1:1). (d) HCl (2.0 mL, 4 M in dioxane), rt., overnight. (e) DMAP (1.0 equiv), TsCl (1.2 equiv), Et_3_N (6.0 equiv), DCM (2.0 mL), rt., 12 h.

The Co-catalyzed indole C–H functionalization/asymmetric ring-opening of 7-oxabenzonorbornadienes could be performed on a gram-scale. The reaction between **1a** (3.0 mmol) and **2a** (4.5 mmol) proceeded well in the presence of a lowered loading of (*R*_a_)-**Co-7** (2.5 mol%), delivering *cis*-**3aa** in 97% yield (989.4 mg) with 95:5 dr and 98% ee after 48 h (Fig. 7a). Besides, *cis*-**3aa** and *cis*-**3ia** underwent hydrogenation and Suzuki–Miyaura cross-coupling reactions, respectively, generating the corresponding products **4** and **5** in good yields (95% and 85%) without the erosion of enantiopurity (Figs. 7b, 7c). In addition, the *N*-Boc protecting group of *trans*-**7ab** could be readily removed in the presence of hydrogen chloride (Fig. 7d), and the further transformation to *trans*-**7ab′** unambiguously assigned their absolute configuration (Fig. 7e).

## Discussion

In conclusion, we have developed Co-catalyzed ARO reactions of 7-oxabenzonorbornadienes via indole C–H activation. The reactions demonstrated opposite diastereoselectivity with binaphthyl-derived chiral cyclopentadienyl-derived cobalt catalysts when compared with that using Cp*Co(CO)I_2_. Good yields and excellent enantioselectivity were obtained for a wide range of substrates. Combined experimental and computational studies offered deep mechanistic insights into the reaction profile and the origins of the diastereo- and enantioselectivity. Moreover, the Co-catalyzed ARO reactions of 7-azabenzonorbornadiene were also realized in a diastereodivergent manner.

## Methods

### General procedure for the enantioselective synthesis of *cis-*3

A sealed tube with a magnetic stir bar was charged with (*R*_a_)-**Co-7** (4.0 mg, 0.005 mmol), NaOPiv · H_2_O (4.4 mg, 0.03 mmol), **1** (0.1 mmol), **2** (0.15 mmol), TFE (0.3 mL) and HFIP (0.1 mL) under argon atmosphere. The mixture was stirred at 50 °C for 18 h. Afterwards, the mixture was cooled to room temperature and quenched by saturated NH_4_Cl aqueous solution (20 mL). The resulted mixture was extracted with ethyl acetate (3 × 15 mL). The combined organic phase was dried over anhydrous Na_2_SO_4_ and filtered. All the volatiles were evaporated under reduced pressure. The remaining residue was purified by column chromatography on silica gel (hexanes/ethyl acetate = 8:1 to 4:1) to afford *cis*-**3**.

### General procedure for the enantioselective synthesis of *cis-*7

A sealed tube with a magnetic stir bar was charged with (*S*_a_)-**Co-4** (7.6 mg, 0.010 mmol), AgSbF_6_ (6.8 mg, 0.020 mmol), Zn(OAc)_2_ (9.2 mg, 0.050 mmol), **1** (0.15 mmol), **6** (24.3 mg, 0.10 mmol) and TFE (1.0 mL) under argon atmosphere. The mixture was stirred at 80 °C for 48 h. Then, the mixture was cooled to room temperature and quenched by saturated NH_4_Cl aqueous solution (20 mL). The resulted mixture was extracted with ethyl acetate (3 × 15 mL). The combined organic phase was dried over anhydrous Na_2_SO_4_ and filtered. All the volatiles were evaporated under reduced pressure. The remaining residue was purified by column chromatography on silica gel (hexanes/ethyl acetate = 10:1 to 6:1) to afford *cis*-**7**.

### General procedure for the enantioselective synthesis of *trans-*7

A sealed tube with a magnetic stir bar was charged with (*S*_a_)-**Co-4** (7.6 mg, 0.010 mmol), AgNTf_2_ (7.8 mg, 0.020 mmol), Fe(OAc)_2_ (8.7 mg, 0.050 mmol), **1** (0.15 mmol), **6** (24.3 mg, 0.10 mmol) and toluene (1.0 mL) under argon atmosphere. The mixture was stirred at 70 °C for 48 h. Afterwards, the mixture was cooled to room temperature and quenched by saturated NH_4_Cl aqueous solution (20 mL). The resulting mixture was extracted with ethyl acetate (3 × 15 mL). The combined organic phase was dried over anhydrous Na_2_SO_4_ and filtered. All the volatiles were evaporated under reduced pressure. The remaining residue was purified by column chromatography on silica gel (hexanes/ethyl acetate = 10:1 to 6:1) to afford *trans*-**7**.

## Supplementary information

Supplementary Information

Description of Additional Supplementary File

Supplementary Data 1

## Data Availability

The X-ray crystallographic coordinates for structures that support the findings of this study have been deposited at the Cambridge Crystallographic Data Centre (CCDC) with the accession code CCDC 2164323 (*cis*-**3da**), 2164324 (*trans*-**3da**) and CCDC 2208797 (*trans*-**7ab′**) (www.ccdc.cam.ac.uk/data_request/cif). The authors declare that all other data supporting the findings of this study are available within the article and Supplementary Information files, and also are available from the corresponding author upon request.

## References

[1] Park Y, Kim Y, Chang S (2017). Transition metal-catalyzed C–H amination: scope, mechanism, and applications. Chem. Rev..

[2] Gensch T, Hopkinson MN, Glorius F, Wencel-Delord J (2016). Mild metal-catalyzed C–H activation: examples and concepts. Chem. Soc. Rev..

[3] Wang F, Yu S, Li X (2016). Transition metal-catalysed couplings between arenes and strained or reactive rings: combination of C–H activation and ring scission. Chem. Soc. Rev..

[4] He J, Wasa M, Chan KSL, Shao Q, Yu J-Q (2017). Palladium-catalyzed transformations of alkyl C–H bonds. Chem. Rev..

[5] Dong Z, Ren Z, Thompson SJ, Xu Y, Dong G (2017). Transition-metal-catalyzed C–H alkylation using alkenes. Chem. Rev..

[6] Wozniak L (2020). Catalytic enantioselective functionalizations of C–H bonds by chiral iridium complexes. Chem. Rev..

[7] Liu C-X, Zhang W-W, Yin S-Y, Gu Q, You S-L (2021). Synthesis of atropisomers by transition-metal-catalyzed asymmetric C-H functionalization reactions. J. Am. Chem. Soc..

[8] Yu X, Zhang Z-Z, Niu J-L, Shi B-F (2022). Coordination-assisted, transition-metal-catalyzed enantioselective desymmetric C–H functionalization. Org. Chem. Front..

[9] Gandeepan P (2019). 3d Transition metals for C−H activation. Chem. Rev..

[10] Loup J, Dhawa U, Pesciaioli F, Wencel-Delord J, Ackermann L (2019). Enantioselective C−H activation with earth-abundant 3d transition metals. Angew. Chem. Int. Ed..

[11] Woźniak L, Cramer N (2019). Enantioselective C−H bond functionalizations by 3d transition-metal catalysts. Trend Chem..

[12] Zheng Y, Zheng C, Gu Q, You S-L (2022). Enantioselective C−H functionalization reactions enabled by cobalt catalysis. Chem. Catal..

[13] Yoshino T, Ikemoto H, Matsunaga S, Kanai M (2013). A cationic high-valent Cp^*^Co^III^ complex for the catalytic generation of nucleophilic organometallic species: directed C−H bond activation. Angew. Chem. Int. Ed..

[14] Ikemoto H, Yoshino T, Sakata K, Matsunaga S, Kanai M (2014). Pyrroloindolone synthesis via a Cp^*^Co^III^-catalyzed redox-neutral directed C−H alkenylation/annulation sequence. J. Am. Chem. Soc..

[15] Li J, Ackermann L (2015). Cobalt-catalyzed C−H cyanation of arenes and heteroarenes. Angew. Chem. Int. Ed..

[16] Hummel JR, Ellman JA (2015). Cobalt(III)-catalyzed synthesis of indazoles and furans by C–H bond functionalization/addition/cyclization cascades. J. Am. Chem. Soc..

[17] Yu D-G, Gensch T, de Azambuja F, Vásquez-Céspedes S, Glorius F (2014). Co(III)-catalyzed C–H activation/formal S_N_-type reactions: selective and efficient cyanation, halogenation, and allylation. J. Am. Chem. Soc..

[18] Park J, Chang S (2015). Comparative catalytic activity of group 9 [Cp^*^M^III^] complexes: cobalt-catalyzed C–H amidation of arenes with dioxazolones as amidating reagents. Angew. Chem. Int. Ed..

[19] Sun B, Yoshino T, Kanai M, Matsunaga S (2015). Cp^*^Co^III^ catalyzed site-selective C–H activation of unsymmetrical O-acyl oximes: synthesis of multisubstituted isoquinolines from terminal and internal alkynes. Angew. Chem. Int. Ed..

[20] Suzuki Y (2015). Dehydrative direct C–H allylation with allylic alcohols under [Cp^*^Co^III^] catalysis. Angew. Chem. Int. Ed..

[21] Lerchen A, Knecht T, Daniliuc CG, Glorius F (2016). Unnatural amino acid synthesis enabled by the regioselective cobalt(III)-catalyzed intermolecular carboamination of alkenes. Angew. Chem. Int. Ed..

[22] Kalsi D, Laskar RA, Barsu N, Premkumar JR, Sundararaju B (2016). C-8-selective allylation of quinoline: a case study of β-hydride vs β-hydroxy elimination. Org. Lett..

[23] Wang X, Lerchen A, Glorius F (2016). A comparative investigation: group 9 Cp^*^M(^III^)-catalyzed formal [4 + 2] cycloaddition as an atom-economic approach to quinazolines. Org. Lett..

[24] Zhou X (2017). Cp^*^Co^III^-catalyzed branch-selective hydroarylation of alkynes via C–H activation: efficient access to α-gem-vinylindoles. ACS Catal..

[25] Zhou X, Pan Y, Li X (2017). Catalyst-controlled regiodivergent alkyne insertion in the context of C−H activation and Diels–Alder reactions: synthesis of fused and bridged cycles. Angew. Chem. Int. Ed..

[26] Bera SS, Sk MR, Maji MS (2019). Weakly coordinating, ketone-directed (η^5^-pentamethylcyclopentadienyl)-cobalt(III)- and (η^5^-pentamethylcyclopentadienyl)-rhodium(III)-catalyzed C−H amidation of arenes: a route to acridone alkaloids. Chem. Eur. J..

[27] Ozols K, Jang YS, Cramer N (2019). Chiral cyclopentadienyl cobalt(III) complexes enable highly enantioselective 3d-metal-catalyzed C–H functionalizations. J. Am. Chem. Soc..

[28] Ozols K, Onodera S, Wozniak L, Cramer N (2021). Cobalt(III)-catalyzed enantioselective intermolecular carboamination by C–H functionalization. Angew. Chem. Int. Ed..

[29] Herraiz AG, Cramer N (2021). Cobalt(III)-catalyzed diastereo- and enantioselective three-component C–H functionalization. ACS Catal..

[30] Pesciaioli F (2018). Enantioselective cobalt(III)-catalyzed C−H activation enabled by chiral carboxylic acid cooperation. Angew. Chem. Int. Ed..

[31] Fukagawa S (2019). Enantioselective C(sp^3^)−H amidation of thioamides catalyzed by a cobalt(III)/chiral carboxylic acid hybrid system. Angew. Chem. Int. Ed..

[32] Kurihara T, Kojima M, Yoshino T, Matsunaga S (2019). Cp^*^Co^III^/chiral carboxylic acid-catalyzed enantioselective 1,4-addition reactions of indoles to maleimides. Asian J. Org. Chem..

[33] Sekine D (2019). Chiral 2-aryl ferrocene carboxylic acids for the catalytic asymmetric C(sp^3^)–H activation of thioamides. Organometallics.

[34] Hirata Y (2022). Cobalt(III)/chiral carboxylic acid-catalyzed enantioselective synthesis of benzothiadiazine-1-oxides via C−H activation. Angew. Chem. Int. Ed..

[35] Liu YH (2019). Cp^*^Co(III)/MPAA-catalyzed enantioselective amidation of ferrocenes directed by thioamides under mild conditions. Org. Lett..

[36] Liu YH (2021). Cp^*^Co(III)-catalyzed enantioselective hydroarylation of unactivated terminal alkenes via C-H activation. J. Am. Chem. Soc..

[37] Zhou Y-B, Zhou T, Qian P-F, Li J-Y, Shi B-F (2022). Synthesis of sulfur-stereogenic sulfoximines via Co(III)/chiral carboxylic acid-catalyzed enantioselective C–H amidation. ACS Catal..

[38] Piou T, Rovis T (2017). Electronic and steric tuning of a prototypical piano stool complex: Rh(III) catalysis for C–H functionalization. Acc. Chem. Res..

[39] Lee S, Semakul N, Rovis T (2020). Direct regio- and diastereoselective synthesis of δ-lactams from acrylamides and unactivated alkenes initiated by Rh^III^-catalyzed C−H activation. Angew. Chem. Int. Ed..

[40] Piou T, Rovis T (2015). Rhodium-catalysed *syn*-carboamination of alkenes via a transient directing group. Nature.

[41] Tanaka J, Nagashima Y, Dias AJA, Tanaka K (2021). Photo-induced *ortho*-C–H borylation of arenes through in situ generation of rhodium(II) ate complexes. J. Am. Chem. Soc..

[42] Shibata Y, Tanaka K (2011). Catalytic [2+2+1] cross-cyclotrimerization of silylacetylenes and two alkynyl esters to produce substituted silylfulvenes. Angew. Chem. Int. Ed..

[43] Wodrich MD, Ye B, Gonthier JF, Corminboeuf C, Cramer N (2014). Ligand-controlled regiodivergent pathways of rhodium(III)-catalyzed dihydroisoquinolone synthesis: experimental and computational studies of different cyclopentadienyl ligands. Chem. Eur. J..

[44] Tomita E (2020). Iridium(III) catalysts with an amide-pendant cyclopentadienyl ligand: double aromatic homologation reactions of benzamides by fourfold C−H activation. Angew. Chem. Int. Ed..

[45] Zell D, Bu Q, Feldt M, Ackermann L (2016). Mild C−H/C−C activation by *Z*-selective cobalt catalysis. Angew. Chem. Int. Ed..

[46] Tanaka R, Tanimoto I, Kojima M, Yoshino T, Matsunaga S (2019). Imidate as the intact directing group for the cobalt-catalyzed C–H allylation. J. Org. Chem..

[47] Kumar SV, Yen A, Lautens M, Guiry PJ (2021). Catalytic asymmetric transformations of oxa- and azabicyclic alkenes. Chem. Soc. Rev..

[48] Boutin R, Koh S, Tam W (2019). Recent advances in transition metal-catalyzed reactions of oxabenzonorbornadiene. Curr. Org. Synth..

[49] Rayabarapu DK, Cheng C-H (2007). New catalytic reactions of oxa- and azabicyclic alkenes. Acc. Chem. Res..

[50] Newton CG, Wang S-G, Oliveira CC, Cramer N (2017). Catalytic enantioselective transformations involving C–H bond cleavage by transition-metal complexes. Chem. Rev..

[51] Rej S, Chatani N (2019). Rhodium-catalyzed C(sp^2^)- or C(sp^3^)−H bond functionalization assisted by removable directing groups. Angew. Chem. Int. Ed..

[52] Kumar SV, Banerjeea S, Punniyamurthy T (2020). Transition metal-catalyzed coupling of heterocyclic alkenes via C–H functionalization: recent trends and applications. Org. Chem. Front..

[53] Yang X, Zheng G, Li X (2019). Rhodium(III)-catalyzed enantioselective coupling of indoles and 7-azabenzonorbornadienes by C–H activation/desymmetrization. Angew. Chem. Int. Ed..

[54] Wang S-G, Cramer N (2019). Enantioselective CpRh^x^(III)-catalyzed C–H functionalization/ring-opening route to chiral cyclopentenylamines. Angew. Chem. Int. Ed..

[55] Mi R, Zheng G, Qi Z, Li X (2019). Rhodium-catalyzed enantioselective oxidative [3+2] annulation of arenes and azabicyclic olefins through twofold C–H activation. Angew. Chem. Int. Ed..

[56] Muralirajan K, Prakash S, Cheng C-H (2017). Cobalt-catalyzed mild ring-opening addition of arenes C−H bond to 7-oxabicyclic alkenes. Adv. Synth. Catal..

[57] Tan H (2020). Cobalt-catalyzed ring-opening addition of azabenzonorbornadienes via C(sp^3^)–H bond activation of 8-methylquinoline. Chem. Commun..

[58] Vinayagam V, Mariappan A, Jana M, Jeganmohan M (2019). Rhodium(III)-catalyzed diastereoselective ring-opening of 7-azabenzonorbornadienes with aromatic ketoximes: synthesis of benzophenanthridine derivatives. J. Org. Chem..

[59] Aravindan N, Jeganmohan M (2021). A short total synthesis of benzophenanthridine alkaloids via a rhodium(III)-catalyzed C–H ring-opening reaction. J. Org. Chem..

[60] Aravindan N, Vinayagam V, Jeganmohan M (2022). A ruthenium-catalyzed cyclization to dihydrobenzo[*c*]phenanthridinone from 7-azabenzonorbornadienes with aryl amides. Org. Lett..

[61] Kong L (2016). Cobalt(III)-catalyzed C–C coupling of arenes with 7-oxabenzonorbornadiene and 2-vinyloxirane via C–H activation. Org. Lett..

[62] Liao G (2019). Synthesis of chiral aldehyde catalysts by Pd-catalyzed atroposelective C−H naphthylation. Angew. Chem. Int. Ed..

[63] Brandes DS, Sirvent A, Mercado BQ, Ellman JA (2021). Three-component 1,2-carboamidation of bridged bicyclic alkenes via Rh^III^-catalyzed addition of C–H bonds and amidating reagents. Org. Lett..

[64] Trifonova EA (2018). A planar-chiral rhodium(III) catalyst with a sterically demanding cyclopentadienyl ligand and its application in the enantioselective synthesis of dihydroisoquinolones. Angew. Chem. Int. Ed..

[65] Pan C, Yin S-Y, Wang S-B, Gu Q, You S-L (2021). Oxygen-linked cyclopentadienyl rhodium(III) complexes-catalyzed asymmetric C−H arylation of benzo[*h*]quinolines with 1-diazonaphthoquinones. Angew. Chem. Int. Ed..

[66] Wang Q (2022). Rhodium(III)-catalyzed enantioselective C–H activation/annulation of ferrocenecarboxamides with internal alkynes. ACS Catal..

[67] Wang Q, Zhang W-W, Zheng C, Gu Q, You S-L (2021). Enantioselective synthesis of azoniahelicenes by Rh-catalyzed C–H annulation with alkynes. J. Am. Chem. Soc..

[68] Cui W-J, Wu Z-J, Gu Q, You S-L (2020). Divergent synthesis of tunable cyclopentadienyl ligands and their application in Rh-catalyzed enantioselective synthesis of isoindolinone. J. Am. Chem. Soc..

[69] Wang Q (2020). Rhodium-catalyzed atroposelective oxidative C–H/C–H cross coupling reaction of 1-aryl isoquinoline derivatives with electron-rich heteroarenes. J. Am. Chem. Soc..

[70] Zheng J, Wang S-B, Zheng C, You S-L (2017). Asymmetric synthesis of spiropyrazolones via Rh-catalyzed C(sp^2^)-H functionalization/annulation reactions. Angew. Chem. Int. Ed..

[71] Zheng J, Cui W-J, Zheng C, You S-L (2016). Synthesis and application of chiral spiro Cp ligands in rhodium-catalyzed asymmetric oxidative coupling of biaryl compounds with alkenes. J. Am. Chem. Soc..

[72] van Dijk L (2021). Mechanistic investigation of Rh(I)-catalysed asymmetric Suzuki–Miyaura coupling with racemic allyl halides. Nat. Catal..

[73] Rathbun CM, Johnson JB (2011). Rhodium-catalyzed acylation with quinolinyl ketones: carbon−carbon single bond activation as the turnover-limiting step of catalysis. J. Am. Chem. Soc..

