# Supplementary material for: Cobalt(III)-catalyzed asymmetric ring-opening of 7-oxabenzonorbornadienes via indole C–H functionalization
Source: Nat Commun. 2023 Feb 25;14:1094. doi: 10.1038/s41467-023-36723-6 (PMC9968317; doi:10.1038/s41467-023-36723-6)
Supplement: Supplementary file 1 — Supplementary Information [file 41467_2023_36723_MOESM1_ESM.pdf]

Supplementary Information

Cobalt(III)-catalyzed asymmetric ring-opening of 7-oxabenzonorbornadienes via indole C–H functionalization

Yang Zheng, Wen-Yun Zhang, Qing Gu, Chao Zheng,* and Shu-Li You*

Laboratory of Organometallic Chemistry, Shanghai Institute of Organic Chemistry, Chinese Academy of Sciences, 345 Lingling Lu, Shanghai 200032, China.

E–mail: zhengchao@sioc.ac.cn or slyou@sioc.ac.cn

Table of Contents

1. Supplementary Methods S3

1.1 General information S3

1.2 General procedure and characterization of products S4

1.3 Gram-scale reaction and derivatization reactions S19

1.4 Mechanistic experiments S22

1.5 Control experiment S28

1.6 X-Ray crystal structures S31

1.7 Absolute configuration of *cis*-**7** S37

1.8 Computational calculations S38

1.9 Copies of NMR spectra S41

1.10 Copies of HPLC chromatograms S146

2. Supplementary References S194

1. Supplementary Notes

1.1 General information

Unless otherwise noted, materials were purchased from commercial suppliers (Shanghai Bidepharm, Adamas-beta®, J&K Scientific, TCI Shanghai and others) and used without further purification. All the solvents were treated according to standard methods. Flash column chromatography was performed using 200–300 mesh silica gel. ^{1}H and ^{13}C NMR spectra were recorded on Bruker, Agilent, and Varian instruments (400 MHz and 100 MHz, respectively) and internally referenced to tetramethylsilane signal or residual protic solvent signals. Data for ^{1}H NMR are recorded as follows: chemical shift (δ, ppm), multiplicity (s = singlet, d = doublet, t = triplet, sept = septet, m = multiplet, br = broad singlet, coupling constant (s) in Hz, integration). Data for ^{13}C NMR and ^{19}F NMR are reported in terms of chemical shift (δ, ppm). All air- and moisture-sensitive reactions were performed under an atmosphere of argon in flame-dried glassware. **Co-1** to **Co-7** were prepared according to the known procedures.[1]

1.2 General procedure and characterization of products

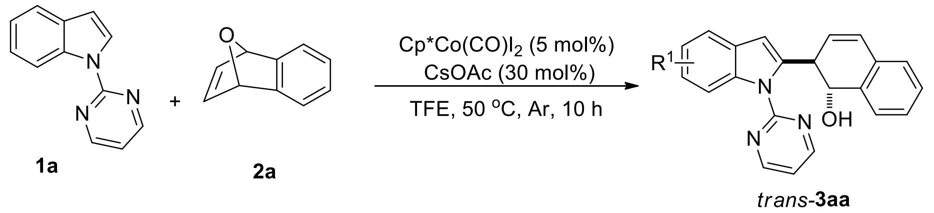

Procedure for the synthesis of *trans*-3aa.[2] A sealed tube with a magnetic stir bar was charged with Cp*Co(CO)I_2 (2.4 mg, 0.005 mmol), CsOAc (5.7 mg, 0.03 mmol), **1a** (19.5 mg, 0.1 mmol), **2a** (21.6 mg, 0.15 mmol), TFE (0.3 mL) under argon atmosphere. The mixture was stirred at 50 °C for 10 h. Then, the mixture was cooled to room temperature and quenched by saturated NH_4Cl aqueous solution (20 mL). The resulting mixture was extracted with ethyl acetate (3 × 15 mL). The combined organic phase was dried over anhydrous Na_2SO_4 and filtered. All the volatiles were evaporated under reduced pressure. The remaining residue was purified by column chromatography on silica gel (hexanes/ethyl acetate = 8:1 to 4:1) to afford *trans*-**3aa**.

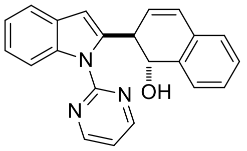

trans-**3aa**, 32.2 mg, 95% yield, pale yellow foam. **^{1}H NMR** (400 MHz, $CDCl_3$) δ 8.80 (d, *J* = 4.8 Hz, 2H), 8.17 (d, *J* = 8.0 Hz, 1H), 7.62-7.60 (m, 1H), 7.54 (dd, *J* = 7.6, 1.6 Hz, 1H), 7.29-7.26 (m, 2H), 7.24-7.15 (m, 3H), 7.15-7.11 (m, 1H), 6.68 (s, 1H), 6.58 (dd, *J* = 9.6, 2.4 Hz, 1H), 6.06 (dd, *J* = 9.6, 3.2 Hz, 1H), 5.12 (d, *J* = 10.4 Hz, 1H), 4.82 (br, 1H), 4.68 (dt, *J* = 10.4, 3.2 Hz, 1H). **^{13}C NMR** (100 MHz, $CDCl_3$) δ 158.4, 157.4, 141.8, 138.1, 136.9, 132.5, 130.5, 129.4, 128.1, 127.9, 127.7, 126.2, 125.9, 123.2, 122.4, 120.4, 117.6, 113.8, 106.8, 75.1, 41.9.
IR (thin film): v_{max} (cm^{-1}) = 3251, 3036, 2923, 2852, 1563, 1452, 1424, 1347, 1262, 1198, 1153, 1078, 991, 845, 792, 741, 690, 635, 522.
HRMS (ESI) calcd for $C_{22}H_{17}ON_3Na$ $[M+Na]^+$: 362.1269. Found: 362.1272.

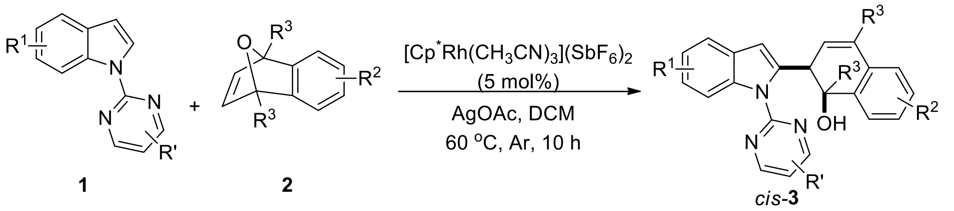

Procedure for the synthesis of racemic products of *cis*-3. A sealed tube with a magnetic stir bar was charged with $[Cp^*Rh(CH_3CN)_3](SbF_6)_2$ (4.2 mg, 0.005 mmol), AgOAc (36.7 mg, 0.22 mmol), **1** (0.1 mmol), **2** (0.15 mmol), and DCM (1.0 mL) under argon atmosphere. The mixture was stirred at 60 °C for 10 h. Afterwards, the mixture was cooled to room temperature and quenched by saturated NH_4Cl aqueous solution (20 mL). The resulting mixture was extracted with ethyl acetate (3 × 15 mL). The combined organic phase was dried over anhydrous Na_2SO_4 and filtered. All the volatiles were evaporated under reduced pressure. The remaining residue was purified by column chromatography on silica gel (hexanes/ethyl acetate = 8:1 to 4:1) to afford racemic products of *cis*-**3**.

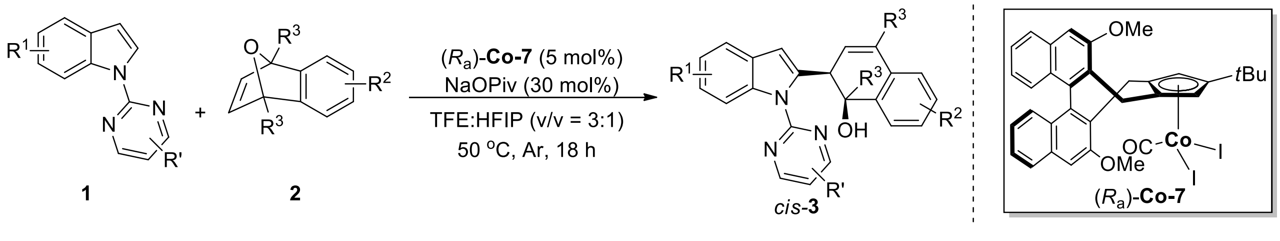

General procedure. A sealed tube with a magnetic stir bar was charged with (R_a)-**Co-7** (4.0 mg, 0.005 mmol), NaOPiv·H_2O (4.4 mg, 0.03 mmol), **1** (0.1 mmol), **2** (0.15 mmol), TFE (0.3 mL) and HFIP (0.1 mL) under argon atmosphere. The mixture was stirred at 50 °C for 18 h. Afterwards, the mixture was cooled to room temperature and quenched by saturated NH_4Cl aqueous solution (20 mL). The resulting mixture was extracted with ethyl acetate (3 × 15 mL). The combined organic phase was dried over anhydrous Na_2SO_4 and filtered. All the volatiles were evaporated under reduced pressure. The remaining residue was purified by column chromatography on silica gel

(hexanes/ethyl acetate = 8:1 to 4:1) to afford *cis*-**3**.

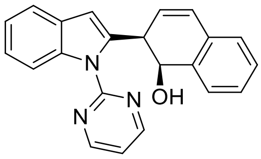

cis-**3aa**, 33.6 mg, 99% yield, pale yellow foam. **^{1}H NMR** (400 MHz, $CDCl_3$) δ 8.85 (d, *J* = 4.8 Hz, 2H), 8.12 (d, *J* = 8.4 Hz, 1H), 7.53-7.51 (m, 1H), 7.43 (d, *J* = 8.0 Hz, 1H), 7.32-7.28 (m, 2H), 7.26-7.18 (m, 3H), 7.14 (td, *J* = 7.6, 1.2 Hz, 1H), 6.66 (dd, *J* = 9.6, 1.6 Hz, 1H), 6.45 (s, 1H), 6.03 (m, 1H), 5.74 (br, 1H), 5.40 (d, *J* = 7.2 Hz, 1H), 4.69 (m, 1H). **^{13}C NMR** (100 MHz, $CDCl_3$) δ 158.5, 157.3, 137.2, 136.5, 136.4, 132.1, 129.2, 128.9, 128.3, 127.9, 127.6, 126.0, 125.97, 123.2, 122.3, 120.4, 117.7, 113.8, 109.3, 70.7, 38.8.
IR (thin film): v_{max} (cm^{-1}) = 3251, 3036, 2924, 2852, 1563, 1452, 1424, 1347, 1262, 1198, 1153, 1078, 991, 845, 792, 742, 690, 635, 522.
HRMS (ESI) calcd for $C_{22}H_{17}ON_3Na$ $[M+Na]^+$: 362.1269. Found: 362.1272.
HPLC conditions: Chiralcel AD–H column (4.6 mm × 250 mm), hexane/*i*-PrOH, 80:20 v/v, flow rate = 1 mL/min, λ = 254 nm, 25 °C. t_R (minor) = 16.49 min, t_R (major) = 19.89 min, ee > 99%. $[\alpha]_D^{35}$ = +211.6 (*c* = 1.0, $CHCl_3$).

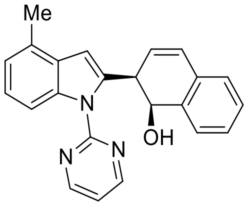

cis-**3ba**, 27.2 mg, 77% yield, pale yellow foam. **^{1}H NMR** (400 MHz, $CDCl_3$) δ 8.85 (d, *J* = 4.8 Hz, 2H), 7.94 (d, *J* = 8.4 Hz, 1H), 7.52-7.49 (m, 1H), 7.33-7.25 (m, 3H), 7.23-7.18 (m, 1H), 7.14-7.10 (m, 1H), 6.95 (d, *J* = 7.2 Hz, 1H), 6.67 (dd, *J* = 9.6, 1.6 Hz, 1H), 6.48 (s, 1H), 6.03 (dd, *J* = 9.6, 5.2 Hz, 1H), 5.45 (br, 1H), 5.35 (d, *J* = 7.2 Hz, 1H), 4.74-4.71 (m, 1H), 2.41 (s, 3H). **^{13}C NMR** (100 MHz, $CDCl_3$) δ 158.4, 157.4, 136.9, 136.1, 136.0, 132.0, 129.6, 128.9, 128.8, 128.2, 127.8, 127.6, 126.2, 126.0, 123.2, 122.5, 117.6, 111.2, 107.5, 70.4, 39.1, 18.6.
IR (thin film): v_{max} (cm^{-1}) = 3033, 2962, 2918, 2853, 1564, 1422, 1346, 1299, 1261, 1198, 1157, 1074, 906, 795, 768, 727, 700, 643, 523, 461.
HRMS (ESI) calcd for $C_{23}H_{19}ON_3Na$ $[M+Na]^+$: 376.1426. Found: 376.1424.
HPLC conditions: Chiralcel AD–H column (4.6 mm × 250 mm), hexane/*i*-PrOH, 80:20 v/v, flow rate = 1 mL/min, λ = 254 nm, 25 °C. t_R (minor) = 13.06 min, t_R (major) = 18.76 min, ee > 99%. $[\alpha]_D^{35}$ = +91.1 (*c* = 1.0, $CHCl_3$).

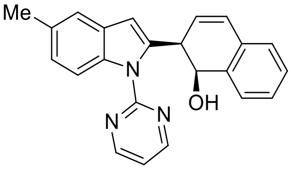

cis-**3ca**, 33.9 mg, 96% yield, pale yellow foam. **^{1}H NMR** (400 MHz, $CDCl_3$) δ 8.84 (d, *J* = 4.8 Hz, 2H), 8.03 (d, *J* = 8.4 Hz, 1H), 7.53-7.47 (m, 1H), 7.31-7.26 (m, 2H), 7.24-7.17 (m, 3H), 7.03 (dd, *J* = 8.8, 1.6 Hz, 1H), 6.65 (dd, *J* = 9.6, 1.6 Hz, 1H), 6.37 (s, 1H), 6.02 (dd, *J* = 9.6, 5.2 Hz, 1H), 5.62 (br, 1H), 5.38 (d, *J* = 7.2 Hz, 1H), 4.74-4.70 (m, 1H), 2.39 (s, 3H). **^{13}C NMR** (100 MHz, $CDCl_3$) δ 158.4, 157.5, 136.53, 136.48, 135.5, 132.2, 131.7, 129.5, 129.1, 128.3, 127.9, 127.6, 126.1, 126.0, 124.7, 120.2, 117.4, 113.7, 109.2, 70.7, 39.0, 21.4.
IR (thin film): v_{max} (cm^{-1}) = 2920, 1563, 1423, 1335, 1297, 1262, 1216, 1172, 1078, 867, 794, 749, 701, 665, 591.
HRMS (ESI) calcd for $C_{23}H_{19}ON_3Na$ $[M+Na]^+$: 376.1426. Found: 376.1428.
HPLC conditions: Chiralcel AD–H column (4.6 mm × 250 mm), hexane/*i*-PrOH, 80:20 v/v, flow rate = 1 mL/min, λ = 254 nm, 25 °C. t_R (minor) = 14.12 min, t_R (major) = 20.02 min, ee = 92%. $[\alpha]_D^{35}$ = +112.0 (*c* = 1.0, $CHCl_3$).

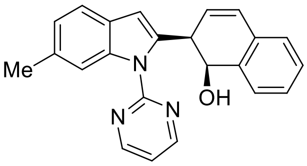

cis-**3da**, 35.0 mg, 99% yield, pale yellow foam. **^{1}H NMR** (400 MHz, $CDCl_3$) δ 8.84 (d, *J* = 4.8 Hz, 2H), 7.92 (s, 1H), 7.53-7.51 (m, 1H), 7.32-7.28 (m, 3H), 7.23-7.19 (m, 2H), 6.99 (dd, *J* = 8.0, 1.6 Hz, 1H), 6.65 (dd, *J* = 9.6, 1.6 Hz, 1H), 6.40 (s, 1H), 6.02 (dd, *J* = 9.6, 5.2 Hz, 1H), 5.83 (br, 1H), 5.39 (d, *J* = 7.2 Hz, 1H), 4.68-4.64 (m, 1H), 2.45 (s,

3H). **^{13}C NMR** (100 MHz, $CDCl_3$) δ 158.5, 157.4, 137.6, 136.5, 135.8, 133.1, 132.2, 129.1, 128.3, 127.8, 127.6, 127.0, 126.1, 126.0, 123.9, 120.0, 117.6, 113.8, 109.2, 70.7, 38.9, 22.2.
IR (thin film): v_{max} (cm^{-1}) = 3030, 1564, 1486, 1422, 1341, 1266, 1197, 1126, 1077, 907, 811, 794, 727, 701, 646, 597, 546, 520, 465.
HRMS (ESI) calcd for $C_{23}H_{19}ON_3Na$ $[M+Na]^+$: 376.1426. Found: 376.1430.
HPLC conditions: Chiralcel AD–H column (4.6 mm × 250 mm), hexane/*i*-PrOH, 80:20 v/v, flow rate = 1 mL/min, λ = 254 nm, 25 °C. t_R (minor) = 13.91 min, t_R (major) = 20.39 min, ee = 99%. $[\alpha]_D^{34}$ = +174.3 (*c* = 1.0, $CHCl_3$).

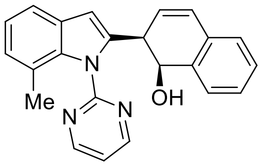

cis-**3ea**, 27.5 mg, 78% yield, pale yellow foam. **^{1}H NMR** (400 MHz, $CDCl_3$) δ 8.89 (d, *J* = 4.8 Hz, 2H), 7.48-7.46 (m, 1H), 7.38-7.31 (m, 2H), 7.28-7.25 (m, 2H), 7.19-7.14 (m, 1H), 7.06 (t, *J* = 7.6 Hz, 1H), 6.97 (d, *J* = 7.2 Hz, 1H), 6.67 (d, *J* = 9.6 Hz, 1H), 6.43 (s, 1H), 5.96 (dd, *J* = 9.6, 5.2 Hz, 1H), 5.28 (br, 1H), 5.24 (d, *J* = 7.2 Hz, 1H), 4.00-3.96 (m, 1H), 1.95 (s, 3H). **^{13}C NMR** (100 MHz, $CDCl_3$) δ 158.5, 158.1, 137.4, 137.1, 136.1, 132.0, 129.8, 128.5, 128.3, 127.8, 127.7, 126.1, 126.1, 125.8, 122.3, 121.9, 119.2, 118.5, 107.1, 70.4, 38.7, 20.5.
IR (thin film): v_{max} (cm^{-1}) = 2962, 1561, 1457, 1417, 1344, 1261, 1221, 1075, 907, 794, 764, 726, 636, 522.
HRMS (ESI) calcd for $C_{23}H_{19}ON_3Na$ $[M+Na]^+$: 376.1426. Found: 376.1430.
HPLC conditions: Chiralcel AD–H column (4.6 mm × 250 mm), hexane/*i*-PrOH, 80:20 v/v, flow rate = 1 mL/min, λ = 254 nm, 25 °C. t_R (minor) = 13.85 min, t_R (major) = 18.17 min, ee = 74%. $[\alpha]_D^{34}$ = +324.0 (*c* = 1.0, $CHCl_3$).

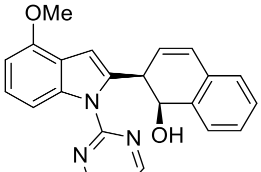

cis-**3fa**, 36.5 mg, 99% yield, pale yellow foam. **^{1}H NMR** (400 MHz, $CDCl_3$) δ 8.82 (d, *J* = 4.8 Hz, 2H), 7.72 (d, *J* = 8.4 Hz, 1H), 7.52-7.50 (m, 1H), 7.31-7.27 (m, 2H), 7.22-7.18 (m, 2H), 7.14 (t, *J* = 8.0 Hz, 1H), 6.65 (dd, *J* = 9.6, 1.6 Hz, 1H), 6.59 (d, *J* = 7.6 Hz, 2H), 6.01 (dd, *J* = 9.6, 5.2 Hz, 1H), 5.61 (br, 1H), 5.35 (d, *J* = 7.2 Hz, 1H), 4.70-4.66 (m, 1H), 3.87 (s, 3H). **^{13}C NMR** (100 MHz, $CDCl_3$) δ 158.4, 157.4, 152.6, 138.5, 136.3, 135.1, 132.1, 128.8, 128.2, 127.9, 127.7, 126.2, 126.0, 124.0, 119.7, 117.8, 107.1, 106.1, 102.4, 70.5, 55.4, 39.0.
IR (thin film): v_{max} (cm^{-1}) = 3003, 1564, 1493, 1423, 1356, 1303, 1254, 1216, 1187, 1145, 1110, 1077, 1036, 991, 820, 794, 747, 700, 664, 546, 521, 463.
HRMS (ESI) calcd for $C_{23}H_{19}O_2N_3Na$ $[M+Na]^+$: 392.1375. Found: 392.1377.
HPLC conditions: Chiralcel OD–H column (4.6 mm × 250 mm), hexane/*i*-PrOH, 80:20 v/v, flow rate = 1 mL/min, λ = 254 nm, 25 °C. t_R (minor) = 32.90 min, t_R (major) = 79.27 min, ee > 99%. $[\alpha]_D^{30}$ = +296.8 (*c* = 1.0, $CHCl_3$).

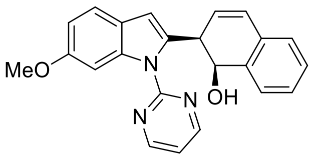

cis-**3ga**, 36.6 mg, 99% yield, pale yellow foam. **^{1}H NMR** (400 MHz, $CDCl_3$) δ 8.83 (d, *J* = 4.8 Hz, 2H), 7.75 (d, *J* = 2.3 Hz, 1H), 7.52-7.50 (m, 1H), 7.30 (d, *J* = 1.6 Hz, 1H), 7.29-7.28 (dd, *J* = 3.6, 2.0 Hz, 2H), 7.22 (t, *J* = 4.8 Hz, 1H), 7.20-7.16 (m, 1H), 6.81 (dd, *J* = 8.4, 2.4 Hz, 1H), 6.67-6.59 (m, 1H), 6.38 (s, 1H), 6.02 (dd, *J* = 9.6, 5.2 Hz, 1H), 5.62 (br, 1H), 5.44-5.29 (m, 1H), 4.71-4.67 (m, 1H), 3.83 (s, 3H). **^{13}C NMR** (100 MHz, $CDCl_3$) δ 158.4, 157.5, 157.2, 138.0, 136.5, 135.4, 132.2, 129.1, 128.3, 127.7, 127.6, 126.03, 126.0, 123.4, 120.7, 117.5, 111.1, 109.2, 98.9, 70.6, 55.9, 38.9.
IR (thin film): v_{max} (cm^{-1}) = 3249, 3230, 2923, 2780, 1615, 1568, 1485, 1434, 1348, 1269, 1233, 1199, 1160, 1136, 1082, 1026, 945, 844, 796, 780, 750, 701, 661, 608, 549, 465, 439.
HRMS (ESI) calcd for $C_{23}H_{19}O_2N_3Na$ $[M+Na]^+$: 392.1375. Found: 392.1377.
HPLC conditions: Chiralcel OD–H column (4.6 mm × 250 mm), hexane/*i*-PrOH, 80:20 v/v, flow rate = 1 mL/min, λ = 254 nm, 25 °C. t_R (minor) = 17.48 min, t_R (major) = 27.19 min, ee > 99%. $[\alpha]_D^{30}$ = +344.5 (*c* = 1.0, $CHCl_3$).

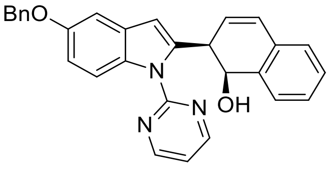

cis-**3ha**, 43.7 mg, 98% yield, pale yellow foam. **^{1}H NMR** (400 MHz, $CDCl_3$) δ 8.81 (d, *J* = 4.8 Hz, 2H), 8.07 (d, *J* = 9.2 Hz, 1H), 7.54-7.51 (m, 1H), 7.47-7.40 (m, 2H), 7.40-7.34 (m, 2H), 7.33-7.28 (m, 3H), 7.23-7.15 (m, 2H), 6.96-6.91 (m, 2H), 6.66 (dd, *J* = 9.6, 1.6 Hz, 1H), 6.37 (s, 1H), 6.02 (dd, *J* = 9.6, 5.2 Hz, 1H), 5.77 (br, 1H), 5.41 (d, *J* = 7.2 Hz, 1H), 5.07 (s, 2H), 4.73-4.70 (m, 1H). **^{13}C NMR** (100 MHz, $CDCl_3$) δ 158.4, 157.3, 154.8, 137.6, 137.1, 136.4, 132.2, 132.1, 129.9, 129.0, 128.6, 128.3, 127.89, 127.86, 127.6, 127.5, 126.0, 125.9, 117.4, 115.0, 113.3, 109.3, 104.0, 70.7, 70.6, 38.9.
IR (thin film): ν_{max} (cm^{-1}) = 3031, 1613, 1564, 1425, 1338, 1177, 1120, 1078, 1015, 793, 748, 698, 664, 549, 522, 464.
HRMS (ESI) calcd for $C_{29}H_{23}O_2N_3Na$ $[M+Na]^+$: 468.1688. Found: 468.1684.
HPLC conditions: Chiralcel AD–H column (4.6 mm × 250 mm), hexane/*i*-PrOH, 80:20 v/v, flow rate = 1 mL/min, λ = 254 nm, 25 °C. t_R (major) = 40.48 min, t_R (minor) = 64.22 min, ee = 99%. $[\alpha]_D^{26}$ = +323.8 (*c* = 1.0, $CHCl_3$).

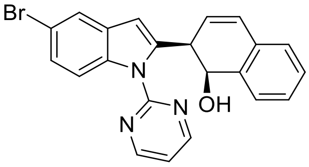

cis-**3ia**, 36.8 mg, 88% yield, pale yellow foam. **^{1}H NMR** (400 MHz, $CDCl_3$) δ 8.81 (d, *J* = 4.8 Hz, 2H), 8.07 (d, *J* = 9.2 Hz, 1H), 7.54-7.51 (m, 1H), 7.47-7.40 (m, 2H), 7.40-7.34 (m, 2H), 7.33-7.28 (m, 3H), 7.23-7.15 (m, 2H), 6.96-6.91 (m, 2H), 6.66 (dd, *J* = 9.6, 1.6 Hz, 1H), 6.37 (s, 1H), 6.02 (dd, *J* = 9.6, 5.2 Hz, 1H), 5.77 (br, 1H), 5.41 (d, *J* = 7.2 Hz, 1H), 5.07 (s, 2H), 4.73-4.70 (m, 1H). **^{13}C NMR** (100 MHz, $CDCl_3$) δ 158.6, 157.1, 138.1, 136.2, 135.9, 132.0, 131.0, 128.55, 128.47, 128.2, 127.8, 126.1, 125.9, 125.9, 122.9, 118.0, 115.5, 115.4, 108.4, 70.7, 38.8. **IR** (thin film): ν_{max} (cm^{-1}) = 2922, 2852, 1564, 1420, 1334, 1261, 1190, 1075, 864, 791, 745, 701, 582.
HRMS (ESI) calcd for $C_{22}H_{16}ON_3BrNa$ $[M+Na]^+$: 440.0374. Found: 440.0376.
HPLC conditions: Chiralcel OD–H column (4.6 mm × 250 mm), hexane/*i*-PrOH, 80:20 v/v, flow rate = 1 mL/min, λ = 254 nm, 25 °C. t_R (minor) = 12.56 min, t_R (major) = 16.61 min, ee = 99%. $[\alpha]_D^{30}$ = +44.5 (*c* = 1.0, $CHCl_3$).

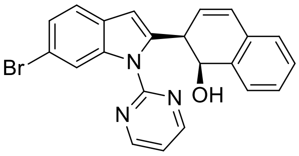

cis-**3ja**, 30.1 mg, 72% yield, pale yellow foam. **^{1}H NMR** (400 MHz, $CDCl_3$) δ 8.81 (d, *J* = 4.8 Hz, 2H), 8.07 (d, *J* = 9.2 Hz, 1H), 7.54-7.51 (m, 1H), 7.47-7.40 (m, 2H), 7.40-7.34 (m, 2H), 7.33-7.28 (m, 3H), 7.23-7.15 (m, 2H), 6.96-6.91 (m, 2H), 6.66 (dd, *J* = 9.6, 1.6 Hz, 1H), 6.37 (s, 1H), 6.02 (dd, *J* = 9.6, 5.2 Hz, 1H), 5.77 (br, 1H), 5.41 (d, *J* = 7.2 Hz, 1H), 5.07 (s, 2H), 4.73-4.70 (m, 1H). **^{13}C NMR** (100 MHz, $CDCl_3$) δ 158.6, 157.0, 137.8, 137.5, 136.3, 132.0, 128.6, 128.4, 128.10, 128., 127.7, 126.1, 125.9, 125.5, 121.4, 118.0, 117.0, 116.8, 109.0, 70.6, 38.8.
IR (thin film): ν_{max} (cm^{-1}) = 2921, 1566, 1424, 1340, 1288, 1195, 1120, 1072, 1017 918, 905, 866, 791, 757, 702, 646, 588, 553, 462, 424.
HRMS (ESI) calcd for $C_{22}H_{16}ON_3BrNa$ $[M+Na]^+$: 440.0374. Found: 440.0372.
HPLC conditions: Chiralcel AD–H column (4.6 mm × 250 mm), hexane/*i*-PrOH, 80:20 v/v, flow rate = 1 mL/min, λ = 254 nm, 25 °C. t_R (minor) = 17.77 min, t_R (major) = 28.72 min, ee = 99%. $[\alpha]_D^{30}$ = +175.7 (*c* = 1.0, $CHCl_3$).

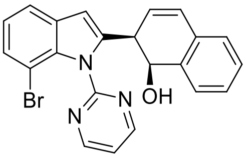

cis-**3ka**, 23.0 mg, 55% yield, pale yellow foam. **^{1}H NMR** (400 MHz, $CDCl_3$) δ 8.91 (d, *J* = 4.8 Hz, 2H), 7.48-7.38 (m, 3H), 7.33 (d, *J* = 7.6 Hz, 1H), 7.30-7.26 (m, 2H), 7.18-7.13 (m, 1H), 6.98 (t, *J* = 7.6 Hz, 1H), 6.67 (d, *J* = 9.6, 1H), 6.43 (s, 1H), 5.95 (dd, *J* = 9.6, 5.2 Hz, 1H), 5.22 (d, *J* = 6.8, 1H), 5.02 (br, 1H), 3.89-3.86 (m, 1H). **^{13}C NMR** (100 MHz, $CDCl_3$) δ 158.7, 157.2, 138.8, 135.92, 135.86, 132.0, 131.9, 128.8, 128.4, 127.8, 127.6, 127.2, 126.2, 122.7, 120.0, 106.3, 105.4, 70.2, 38.8.
IR (thin film): ν_{max} (cm^{-1}) = 2960, 2924, 2853, 1563, 1413, 1347, 1261, 1184, 1077, 1019, 796, 729, 701, 632.
HRMS (ESI) calcd for $C_{22}H_{16}ON_3BrNa$ $[M+Na]^+$: 440.0374. Found: 440.0374.

HPLC conditions: Chiralcel AD–H column (4.6 mm × 250 mm), hexane/*i*-PrOH, 80:20 v/v, flow rate = 1 mL/min, λ = 254 nm, 25 °C. t_R (minor) = 16.58 min, t_R (major) = 26.06 min, ee = 83%. $[\alpha]_D^{30}$ = +44.8 (*c* = 1.0, $CHCl_3$).

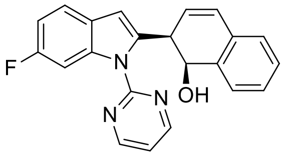

cis-**3la**, 31.8 mg, 89% yield, pale yellow foam. **^{1}H NMR** (400 MHz, $CDCl_3$) δ 8.85 (d, *J* = 4.8 Hz, 2H), 7.91 (dd, *J* = 10.8, 2.4 Hz, 1H), 7.53-7.50 (m, 1H), 7.36-7.25 (m, 4H), 7.23-7.16 (m, 1H), 6.91 (td, *J* = 8.8, 2.4 Hz, 1H), 6.66 (d, *J* = 9.6 Hz, 1H), 6.41 (s, 1H), 6.01 (dd, *J* = 9.6, 5.2 Hz, 1H), 5.61 (br, 1H), 5.39 (d, *J* = 7.2 Hz, 1H), 4.73-4.70 (m, 1H). **^{13}C NMR** (100 MHz, $CDCl_3$) δ 160.4 (d, *J* = 236.4 Hz), 158.4, 157.2, 137.2, 137.1-137.0 (m), 136.2, 131.9, 128.7, 128.3, 127.9, 127.6, 126.0, 125.9, 125.5 (d, *J* = 1.1 Hz), 120.8 (d, *J* = 9.9 Hz), 117.7, 110.5 (d, *J* = 24.1 Hz), 109.0, 101.2 (d, *J* = 28.5 Hz), 70.5, 38.8. **^{19}F NMR** (376 MHz, $CDCl_3$) δ -119.4.
IR (thin film): ν_{max} (cm^{-1}) = 2924, 1566, 1482, 1422, 1351, 1261, 1196, 1136, 1078, 1015, 953, 908, 847, 785, 729, 701, 647, 608, 521, 462.
HRMS (ESI) calcd for $C_{22}H_{16}ON_3FNa$ $[M+Na]^+$: 380.1175. Found: 380.1158.
HPLC conditions: Chiralcel AD–H column (4.6 mm × 250 mm), hexane/*i*-PrOH, 80:20 v/v, flow rate = 1 mL/min, λ = 254 nm, 25 °C. t_R (minor) = 15.20 min, t_R (major) = 22.55 min, ee > 99%. $[\alpha]_D^{29}$ = +163.2 (*c* = 1.0, $CHCl_3$).

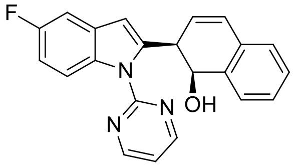

cis-**3ma**, 26.8 mg, 75% yield, pale yellow foam. **^{1}H NMR** (400 MHz, $CDCl_3$) δ 8.85 (d, *J* = 4.8 Hz, 2H), 8.07 (dd, *J* = 9.2, 4.8 Hz, 1H), 7.54-7.50 (m, 1H), 7.36-7.26 (m, 3H), 7.22-7.15 (m, 1H), 7.06 (dd, *J* = 8.8, 2.4 Hz, 1H), 6.93 (td, *J* = 9.2, 2.4 Hz, 1H), 6.67 (d, *J* = 9.6, 1H), 6.39 (s, 1H), 6.01 (dd, *J* = 9.6, 5.2 Hz, 1H), 5.68 (br, 1H), 5.41 (d, *J* = 7.2 Hz, 1H), 4.75-4.66 (m, 1H). **^{13}C NMR** (100 MHz, $CDCl_3$) δ 159.1 (d, *J* = 236.3 Hz), 158.5, 157.2, 138.4, 136.3, 133.6, 132.0, 130.1-129.9 (m), 128.6, 128.4, 128.1, 127.7, 126.1, 125.9, 117.8, 115.0 (d, *J* = 9.2 Hz), 111.0 (d, *J* = 25.0 Hz), 109.0 (d, *J* = 4.0 Hz), 105.5 (d, *J* = 23.4 Hz), 70.7, 38.9. **^{19}F NMR** (376 MHz, $CDCl_3$) δ -121.9.
IR (thin film): ν_{max} (cm^{-1}) = 2904, 1567, 1471, 1449, 1427, 1349, 1290, 1179, 1116, 1069, 989, 954, 864, 827, 802, 769, 725, 694, 651, 633, 606, 588, 560, 526, 493, 478, 430.
HRMS (ESI) calcd for $C_{22}H_{16}ON_3FNa$ $[M+Na]^+$: 380.1175. Found: 380.1178.
HPLC conditions: Chiralcel AD–H column (4.6 mm × 250 mm), hexane/*i*-PrOH, 80:20 v/v, flow rate = 1 mL/min, λ = 254 nm, 25 °C. t_R (minor) = 20.30 min, t_R (major) = 22.21 min, ee = 99%. $[\alpha]_D^{28}$ = +157.0 (*c* = 1.0, $CHCl_3$).

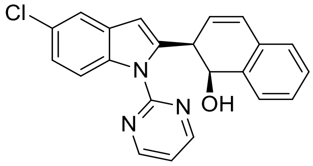

cis-**3na**, 28.0 mg, 75% yield, pale yellow foam. **^{1}H NMR** (400 MHz, $CDCl_3$) δ 8.88 (d, *J* = 4.8 Hz, 2H), 8.07 (d, *J* = 8.8 Hz, 1H), 7.53-7.51 (m, 1H), 7.40 (d, *J* = 2.0 Hz, 1H), 7.35-7.29 (m, 3H), 7.24-7.16 (m, 2H), 6.69 (d, *J* = 9.6 Hz, 1H), 6.39 (s, 1H), 6.03 (dd, *J* = 9.6, 5.2 Hz, 1H), 5.43 (d, *J* = 7.2 Hz, 1H), 4.73-4.69 (m, 1H). **^{13}C NMR** (100 MHz, $CDCl_3$) δ 158.6, 157.1, 138.2, 136.3, 135.5, 132.0, 130.4, 128.6, 128.5, 128.2, 127.7, 126.1, 125.9, 123.3, 119.8, 118.0, 115.1, 108.5, 70.7, 38.9.
IR (thin film): ν_{max} (cm^{-1}) = 3021, 1563, 1421, 1335, 1193, 1069, 862, 792, 746, 701, 633, 589, 519.
HRMS (ESI) calcd for $C_{22}H_{16}ON_3ClNa$ $[M+Na]^+$: 396.0880. Found: 396.0877.
HPLC conditions: Chiralcel AD–H column (4.6 mm × 250 mm), hexane/*i*-PrOH, 80:20 v/v, flow rate = 1 mL/min, λ = 254 nm, 25 °C. t_R (minor) = 11.65 min, t_R (major) = 15.26 min, ee =97%. $[\alpha]_D^{35}$ = +71.0 (*c* = 1.0, $CHCl_3$).

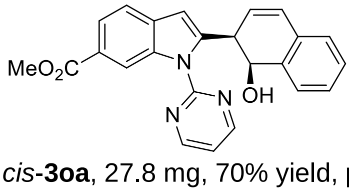

cis-**3oa**, 27.8 mg, 70% yield, pale yellow foam. **^{1}H NMR** (400 MHz, $CDCl_3$) δ 8.90 (d, *J* = 4.8 Hz, 2H), 8.77 (s, 1H), 7.84 (dd, *J* = 8.0, 1.6 Hz, 1H), 7.52-7.50 (m, 1H), 7.44 (d, *J* = 8.0 Hz, 1H), 7.33-7.28 (m, 3H), 7.22-7.15 (m, 1H), 6.67 (d, *J* = 9.6 Hz, 1H), 6.46 (s, 1H), 6.00 (dd, *J* = 9.6, 5.2 Hz, 1H), 5.70 (br, 1H), 5.43 (d, *J* = 7.2 Hz, 1H), 4.73-

4.60 (m, 1H), 3.91 (s, 3H). **^{13}C NMR** (100 MHz, $CDCl_3$) δ 168.1, 158.8, 157.0, 140.3, 136.6, 136.2, 133.0, 131.9, 128.5, 128.4, 128.3, 127.7, 126.1, 125.9, 124.8, 123.4, 120.0, 118.3, 115.9, 108.9, 70.7, 52.1, 38.9.
IR (thin film): ν_{max} (cm^{-1}) = 2945, 1711, 1567, 1537, 1477, 1430, 1355, 1306, 1286, 1240, 1201, 1118, 1095, 982, 906, 854, 796, 763, 739, 706, 683, 657, 632, 559, 516, 461.
HRMS (ESI) calcd for $C_{24}H_{19}O_3N_3Na$ $[M+Na]^+$: 420.1324. Found: 420.1326.
HPLC conditions: Chiralcel AD–H column (4.6 mm × 250 mm), hexane/*i*-PrOH, 80:20 v/v, flow rate = 1 mL/min, λ = 254 nm, 25 °C. t_R (minor) = 25.44 min, t_R (major) = 37.96 min, ee > 99%. $[\alpha]_D^{30}$ = +352.7 (*c* = 1.0, $CHCl_3$).

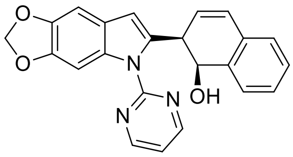

cis-**3pa**, 35.7 mg, 93% yield, pale yellow foam. **^{1}H NMR** (400 MHz, $CDCl_3$) δ 8.83 (d, *J* = 4.8 Hz, 2H), 7.69 (s, 1H), 7.54-7.50 (m, 1H), 7.31-7.27 (m, 2H), 7.25-7.22 (m, 1H), 7.20-7.16 (m, 1H), 6.79 (s, 1H), 6.63 (d, *J* = 9.6 Hz, 1H), 6.30 (s, 1H), 5.99 (dd, *J* = 9.6, 5.2 Hz, 1H), 5.91 (dd, *J* = 9.6, 1.6 Hz, 2H), 5.76 (br, 1H), 5.37 (d, *J* = 7.2 Hz, 1H), 4.67-4.63 (m, 1H). **^{13}C NMR** (100 MHz, $CDCl_3$) δ 158.4, 157.3, 145.4, 144.2, 136.4, 134.9, 132.1, 132.0, 129.0, 128.3, 127.8, 127.6, 126.0, 125.9, 123.4, 117.6, 109.5, 100.9, 99.1, 95.8, 70.7, 38.8.
IR (thin film): ν_{max} (cm^{-1}) = 2892, 1565, 1460, 1424, 1343, 1283, 1190, 1160, 1082, 1036, 941, 849, 789, 729, 700, 643, 522.
HRMS (ESI) calcd for $C_{23}H_{17}O_3N_3Na$ $[M+Na]^+$: 406.1168. Found: 406.1168.
HPLC conditions: Chiralcel AD–H column (4.6 mm × 250 mm), hexane/*i*-PrOH, 80:20 v/v, flow rate 1 mL/min, λ = 254 nm, 25 °C. t_R (major) = 39.44 min, t_R (minor) = 43.05 min, ee = 96%. $[\alpha]_D^{30}$ = +214.8 (*c* = 1.0, $CHCl_3$).

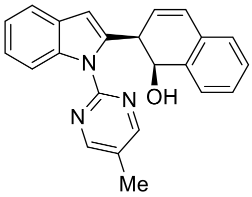

cis-**3qa**, 20.5 mg, 58% yield, pale yellow foam. **^{1}H NMR** (400 MHz, $CDCl_3$) δ 8.68 (s, 2H), 8.02 (d, *J* = 8.4 Hz, 1H), 7.54-7.49 (m, 1H), 7.42 (d, *J* = 7.6 Hz, 1H), 7.31-7.28 (m, 2H), 7.23-7.16 (m, 2H), 7.16-7.08 (m, 1H), 6.65 (d, *J* = 9.6 Hz, 1H), 6.41 (s, 1H), 6.01 (dd, *J* = 9.6, 5.2 Hz, 1H), 5.88 (br, 1H), 5.39 (d, *J* = 7.2 Hz, 1H), 4.63-4.59 (m, 1H), 2.40 (s, 3H). **^{13}C NMR** (100 MHz, $CDCl_3$) δ 158.5, 155.4, 137.2, 136.5, 136.3, 132.1, 129.1, 128.9, 128.3, 127.9, 127.6, 127.4, 126.0, 125.9, 123.0, 122.0, 120.4, 113.4, 108.5, 70.7, 38.7, 15.3.
IR (thin film): ν_{max} (cm^{-1}) = 2921, 2779, 1589, 1561, 1436, 1347, 1299, 1198, 1114, 1081, 1016, 786, 735, 701, 662, 608, 548, 522, 465.
HRMS (ESI) calcd for $C_{23}H_{19}ON_3Na$ $[M+Na]^+$: 376.1426. Found: 376.1425.
HPLC conditions: Chiralcel AD–H column (4.6 mm × 250 mm), hexane/*i*-PrOH, 80:20 v/v, flow rate = 1 mL/min, λ = 254 nm, 25 °C. t_R (major) = 23.19 min, t_R (minor) = 24.22 min, ee > 99%. $[\alpha]_D^{31}$ = +213.2 (*c* = 1.0, $CHCl_3$).

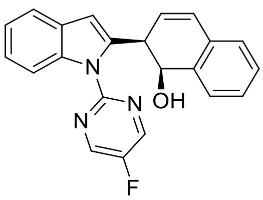

cis-**3ra**, 31.1 mg, 87% yield, pale yellow foam. **^{1}H NMR** (400 MHz, $CDCl_3$) δ 8.70 (s, 2H), 8.03 (d, *J* = 8.4 Hz, 1H), 7.46 (d, *J* = 7.6 Hz, 2H), 7.34-7.27 (m, 2H), 7.25-7.13 (m, 3H), 6.67 (dd, *J* = 9.6, 1.6 Hz, 1H), 6.51 (s, 1H), 6.05 (dd, *J* = 9.6, 4.8 Hz, 1H), 5.26 (d, *J* = 6.8 Hz, 1H), 4.72-4.69 (m, 1H), 4.36 (br, 1H). **^{13}C NMR** (100 MHz, $CDCl_3$) δ 156.4, 153.8, 153.7 (d, *J* = 3.3 Hz), 146.3 (d, *J* = 21.8 Hz), 137.2, 136.5 (d, *J* = 81.7 Hz), 132.1, 129.0, 128.8, 128.3, 128.0, 127.98, 126.4, 126.3, 123.3, 122.3, 120.5, 113.4, 108.8, 70.4, 39.2. **^{19}F NMR** (376 MHz, $CDCl_3$) δ -142.0.
IR (thin film): ν_{max} (cm^{-1}) = 2919, 1568, 1550, 1436, 1347, 1291, 1245, 1195, 1078, 1015, 922, 793, 742, 703, 676, 640, 603, 586, 549, 520, 468, 432.
HRMS (ESI) calcd for $C_{22}H_{16}ON_3FNa$ $[M+Na]^+$: 380.1175. Found: 380.1179.
HPLC conditions: Chiralcel AD–H column (4.6 mm × 250 mm), hexane/*i*-PrOH, 80:20 v/v, flow rate = 1 mL/min, λ = 254 nm, 25 °C. t_R (minor) = 16.12 min, t_R (major) = 22.41 min, ee = 90%. $[\alpha]_D^{30}$ = +141.2 (*c* = 1.0, $CHCl_3$).

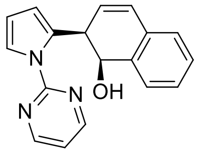

cis-**3sa**, 19.1 mg, 66% yield, pale yellow oil. **^{1}H NMR** (400 MHz, $CDCl_3$) δ 8.70 (d, *J* = 4.8 Hz, 2H), 7.70-7.69 (m, 1H), 7.48-7.45 (m, 1H), 7.30-7.26 (m, 2H), 7.18-7.15 (m, 2H), 6.63 (dd, *J* = 9.6, 1.6 Hz, 1H), 6.20 (t, *J* = 3.2 Hz, 1H), 6.14-6.13 (m, 1H), 6.03 (dd, *J* = 9.6, 4.8 Hz, 1H), 5.25 (d, *J* = 6.4 Hz, 1H), 4.98-4.89 (m, 1H), 4.68 (br, 1H). **^{13}C NMR** (100 MHz, $CDCl_3$) δ 158.4, 157.3, 136.4, 132.3, 130.2, 129.6, 128.0, 127.8, 127.5, 126.5, 126.1, 122.6, 117.7, 114.9, 110.7, 70.6, 39.2.
IR (thin film): ν_{max} (cm^{-1}) = 3365, 2906, 1575, 1484, 1447, 1406, 1280, 1193, 1144, 1119, 1072, 995, 941, 916, 880, 815, 799, 763, 724, 684, 640, 598, 537, 515, 494, 423.
HRMS (ESI) calcd for $C_{18}H_{15}ON_3Na$ $[M+Na]^+$: 312.1113. Found: 312.1114.
HPLC conditions: Chiralcel OD–H column (4.6 mm × 250 mm), hexane/*i*-PrOH, 85:15 v/v, flow rate = 1 mL/min, λ = 254 nm, 25 °C. t_R (minor) = 8.91 min, t_R (major) = 13.93 min, ee = 72%. $[\alpha]_D^{31}$ = -9.7 (*c* = 1.0, $CHCl_3$).

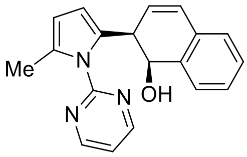

cis-**3ta**, 17.3 mg, 57% yield, pale yellow oil. **^{1}H NMR** (400 MHz, $CDCl_3$) δ 8.83 (d, *J* = 4.8 Hz, 2H), 7.51-7.48 (m, 1H), 7.30 (t, *J* = 4.8 Hz, 1H), 7.26-7.21 (m, 2H), 7.14-7.08 (m, 1H), 6.55 (d, *J* = 9.6 Hz, 1H), 5.93-5.89 (m, 3H), 5.46 (br, 1H), 5.15 (d, *J* = 7.2 Hz, 1H), 4.18-4.15 (m, 1H), 2.31 (s, 3H). **^{13}C NMR** (100 MHz, $CDCl_3$) δ 158.4, 157.3, 136.6, 132.3, 131.4, 129.3, 128.9, 128.0, 127.5, 127.4, 126.1, 125.9, 118.6, 111.4, 110.2, 70.7, 38.7, 14.8.
IR (thin film): ν_{max} (cm^{-1}) = 2922, 1561, 1423, 1264, 1219, 1077, 1021, 814, 768, 698, 639, 616, 543, 519.
HRMS (ESI) calcd for $C_{19}H_{17}ON_3Na$ $[M+Na]^+$: 326.1269. Found: 326.1266.
HPLC conditions: Chiralcel OD–H column (4.6 mm × 250 mm), hexane/*i*-PrOH, 85:15 v/v, flow rate = 1 mL/min, λ = 254 nm, 25 °C. t_R (minor) = 10.89 min, t_R (major) = 14.36 min, ee = 81%. $[\alpha]_D^{35}$ = -42.8 (*c* = 1.0, $CHCl_3$).

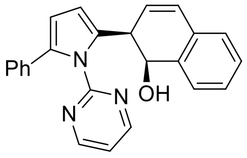

cis-**3ua**, 34.7 mg, 95% yield, pale yellow foam. **^{1}H NMR** (400 MHz, $CDCl_3$) δ 8.72 (d, *J* = 4.8 Hz, 2H), 7.52-7.50 (m, 1H), 7.30-7.23 (m, 3H), 7.21-7.10 (m, 4H), 7.03-6.95 (m, 2H), 6.61 (d, *J* = 9.6 Hz, 1H), 6.30 (d, *J* = 3.6 Hz, 1H), 6.08 (d, *J* = 3.6 Hz, 1H), 5.94 (dd, *J* = 9.6, 5.2 Hz, 1H), 5.24 (br, 1H), 5.17 (d, *J* = 7.2 Hz, 1H), 4.10-4.07 (m, 1H). **^{13}C NMR** (100 MHz, $CDCl_3$) δ 158.5, 157.7, 136.4, 136.1, 133.8, 132.2, 131.6, 128.6, 128.20, 128.17, 127.9, 127.6, 126.3, 126.1, 126.0, 119.2, 111.9, 111.8, 70.6, 38.7.
IR (thin film): ν_{max} (cm^{-1}) = 2784, 1571, 1447, 1423, 1319, 1281, 1229, 1188, 1120, 1084, 1027, 965, 906, 812, 792, 775, 750, 696, 642, 604, 549, 524, 463.
HRMS (ESI) calcd for $C_{24}H_{19}ON_3Na$ $[M+Na]^+$: 388.1426. Found: 388.1425.
HPLC conditions: Chiralcel AD–H column (4.6 mm × 250 mm), hexane/*i*-PrOH, 80:20 v/v, flow rate = 1 mL/min, λ = 254 nm, 25 °C. t_R (minor) = 17.05 min, t_R (major) = 23.14 min, ee = 69%. $[\alpha]_D^{34}$ = -61.3 (*c* = 1.0, $CHCl_3$).

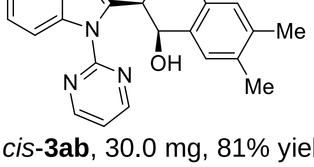

cis-**3ab**, 30.0 mg, 81% yield, pale yellow foam. **^{1}H NMR** (400 MHz, $CDCl_3$) δ 8.85 (d, *J* = 4.8 Hz, 2H), 8.12 (d, *J* = 8.4 Hz, 1H), 7.45 (d, *J* = 7.6 Hz, 1H), 7.28 (s, 1H), 7.24 (d, *J* = 5.2 Hz, 1H), 7.22-7.18 (m, 1H), 7.17-7.13 (m, 1H), 6.98 (s, 1H), 6.61 (d, *J* = 9.6 Hz, 1H), 6.50 (s, 1H), 5.96 (dd, *J* = 9.6, 5.2 Hz, 1H), 5.45 (br, 1H), 5.32 (d, *J* = 7.2 Hz, 1H), 4.70-4.67 (m, 1H), 2.29 (s, 3H), 2.26 (s, 3H). **^{13}C NMR** (100 MHz, $CDCl_3$) δ 158.5, 157.4, 153.3, 137.2, 137.1, 136.7, 135.7, 133.7, 129.7, 129.3, 127.9, 127.8, 127.6, 123.2, 122.2, 120.4, 117.6, 113.8, 109.2, 70.5, 39.2, 19.9, 19.6.
IR (thin film): ν_{max} (cm^{-1}) = 2916, 1562, 1500, 1451, 1420, 1345, 1258, 1215, 1019, 881, 798, 743, 703, 618, 480, 425.
HRMS (ESI) calcd for $C_{24}H_{21}ON_3Na$ $[M+Na]^+$: 390.1582. Found: 390.1578.

HPLC conditions: Chiralcel AD–H column (4.6 mm × 250 mm), hexane/*i*-PrOH, 80:20 v/v, flow rate = 1 mL/min, λ = 254 nm, 25 °C. t_R (minor) = 16.07 min, t_R (major) = 18.63 min, ee = 94%. $[\alpha]_D^{30}$ = +163.8 (*c* = 1.0, $CHCl_3$).

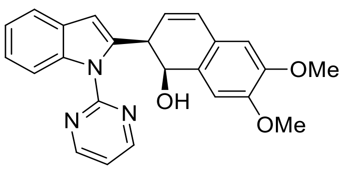

cis-**3ac**, 14.0 mg, 35% yield, pale yellow foam. **^{1}H NMR** (400 MHz, $CDCl_3$) δ 8.86 (d, *J* = 4.8 Hz, 2H), 8.11 (d, *J* = 8.4 Hz, 1H), 7.44 (d, *J* = 7.6 Hz, 1H), 7.30-7.25 (m, 1H), 7.23-7.19 (m, 1H), 7.15 (t, *J* = 7.6 Hz, 1H), 7.10 (s, 1H), 6.75 (s, 1H), 6.56 (d, *J* = 9.6 Hz, 1H), 6.46 (s, 1H), 5.92 (dd, *J* = 9.6, 5.2 Hz, 1H), 5.88 (br, 1H), 5.36 (d, *J* = 7.2 Hz, 1H), 4.65-4.57 (m, 1H), 3.94 (s, 3H), 3.88 (s, 3H). **^{13}C NMR** (100 MHz, $CDCl_3$) δ 158.5, 157.3, 149.0, 148.0, 137.2, 136.7, 129.6, 129.3, 127.4, 127.1, 124.8, 123.2, 122.3, 120.4, 117.7, 113.8, 109.7, 109.6, 109.3, 70.7, 56.2, 56.0, 38.7.
IR (thin film): v_{max} (cm^{-1}) = 2926, 1562, 1505, 1452, 1417, 1345, 1303, 1243, 1197, 1161, 1127, 1005, 889, 855, 828, 796, 744, 629, 478.
HRMS (ESI) calcd for $C_{24}H_{21}O_3N_3Na$ $[M+Na]^+$: 422.1481. Found: 422.1482.
HPLC conditions: Chiralcel OD–H column (4.6 mm × 250 mm), hexane/*i*-PrOH, 80:20 v/v, flow rate = 1 mL/min, λ = 254 nm, 25 °C. t_R (minor) = 26.45 min, t_R (major) = 41.80 min, ee = 95%. $[\alpha]_D^{30}$ = +14.9 (*c* = 1.0, $CHCl_3$).

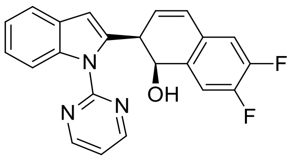

cis-**3ad**, 28.2 mg, 75% yield, pale yellow foam. **^{1}H NMR** (400 MHz, $CDCl_3$) δ 8.88 (d, *J* = 4.8 Hz, 2H), 8.10 (d, *J* = 8.4 Hz, 1H), 7.43 (d, *J* = 7.8 Hz, 1H), 7.37-7.32 (m, 1H), 7.29 (t, *J* = 4.8 Hz, 1H), 7.24-7.19 (m, 1H), 7.18-7.12 (m, 1H), 6.99 (dd, *J* = 10.8, 7.6 Hz, 1H), 6.55 (d, *J* = 9.6 Hz, 1H), 6.37-6.36 (m, 2H), 6.03 (dd, *J* = 9.6, 5.6 Hz, 1H), 5.37 (d, *J* = 7.6 Hz, 1H), 4.61-4.57 (m, 1H). **^{13}C NMR** (100 MHz, $CDCl_3$) δ 158.6, 157.1, 150.29 (dd, *J* = 248.3, 12.7 Hz), 149.64 (dd, *J* = 246.0, 13.2 Hz), 137.2, 135.2, 133.6 (dd, *J* = 5.5, 3.5 Hz), 129.7 (d, *J* = 2.6 Hz), 129.1, 128.8 (dd, *J* = 6.4, 3.9 Hz), 126.4-126.2 (m), 123.4, 122.5, 120.5, 117.8, 115.5 (d, *J* = 18.9 Hz), 114.6 (d, *J* = 18.0 Hz), 113.9, 109.5, 70.2, 37.8. **^{19}F NMR** (376 MHz, $CDCl_3$) δ -138.6, -141.2.
IR (thin film): v_{max} (cm^{-1}) = 2923, 1564, 1505, 1452, 1427, 1349, 1311, 1261, 1200, 1149, 1104, 1081, 907, 879, 798, 725, 640, 593, 551, 437.
HRMS (ESI) calcd for $C_{22}H_{15}ON_3F_2Na$ $[M+Na]^+$: 398.1081. Found: 398.1084.
HPLC conditions: Chiralcel AD–H column (4.6 mm × 250 mm), hexane/*i*-PrOH, 85:15 v/v, flow rate = 1 mL/min, λ = 254 nm, 25 °C. t_R (major) = 16.95 min, t_R (minor) = 18.19 min, ee = 99%. $[\alpha]_D^{35}$ = +286.1 (*c* = 1.0, $CHCl_3$).

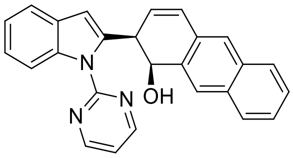

cis-**3ae**, 19.5 mg, 50% yield, pale yellow foam. **^{1}H NMR** (400 MHz, $CDCl_3$) δ 8.91 (d, *J* = 4.8 Hz, 2H), 8.09 (d, *J* = 8.4 Hz, 1H), 7.96 (s, 1H), 7.84 (d, *J* = 7.6 Hz, 1H), 7.79 (d, *J* = 7.6 Hz, 1H), 7.63 (s, 1H), 7.48-7.39 (m, 2H), 7.37-7.27 (m, 2H), 7.20-7.16 (m, 1H), 7.09 (td, *J* = 7.6, 1.2 Hz, 1H), 6.84 (d, *J* = 9.6 Hz, 1H), 6.34 (br, 1H), 6.30 (s, 1H), 6.11 (dd, *J* = 9.6, 6.0 Hz, 1H), 5.58 (d, *J* = 7.2 Hz, 1H), 4.81-4.65 (m, 1H). **^{13}C NMR** (100 MHz, $CDCl_3$) δ 158.6, 157.3, 137.2, 136.2, 135.1, 133.9, 133.2, 131.0, 129.6, 129.2, 128.3, 128.2, 127.8, 126.0, 125.9, 124.7, 124.6, 123.2, 122.3, 120.5, 117.7, 113.8, 109.4, 71.2, 38.8.
IR (thin film): v_{max} (cm^{-1}) = 2920, 2851, 1665, 1563, 1452, 1424, 1346, 1261, 1198, 1150, 1081, 886, 800, 742, 696, 637, 616, 477, 433.
HRMS (ESI) calcd for $C_{26}H_{19}ON_3Na$ $[M+Na]^+$: 412.1426. Found: 412.1421.
HPLC conditions: Chiralcel AD–H column (4.6 mm × 250 mm), hexane/*i*-PrOH, 80:20 v/v, flow rate = 1 mL/min, λ = 254 nm, 25 °C. t_R (minor) = 27.05 min, t_R (major) = 31.74 min, ee = 99%. $[\alpha]_D^{31}$ = +24.3 (*c* = 1.0, $CHCl_3$).

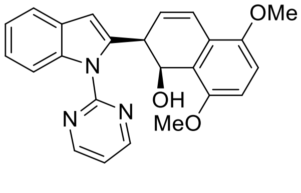

cis-**3af**, 26.8 mg, 67% yield, pale yellow foam. **^{1}H NMR** (400 MHz, $CDCl_3$) δ 8.76 (d, *J* = 4.8 Hz, 2H), 8.28 (d, *J* = 8.0 Hz, 1H), 7.61 (d, *J* = 7.6 Hz, 1H), 7.31-7.26 (m, 1H), 7.22 (td, *J* = 7.2, 1.2 Hz, 1H), 7.16 (t, *J* = 4.8 Hz, 1H), 7.01 (dd, *J* = 10.0, 3.2 Hz, 1H), 6.90 (s, 1H), 6.85-6.76 (m, 2H), 6.07 (dt, *J* = 10.0, 2.0 Hz, 1H), 5.37 (d, *J* = 4.8 Hz, 1H), 4.95 (dt, *J* = 5.2, 2.8 Hz, 1H), 3.83 (s, 3H), 3.80 (s, 3H). **^{13}C NMR** (100 MHz, $CDCl_3$) δ 158.4, 158.1, 151.2, 149.8, 139.5, 137.4, 129.2, 129.0, 125.0, 123.3, 122.5, 122.2, 121.2, 120.3, 117.5, 114.1, 111.4, 111.3, 109.0, 62.7, 56.6, 56.4, 41.5.
IR (thin film): ν_{max} (cm^{-1}) = 2934, 1563, 1481, 1452, 1421, 1345, 1257, 1188, 1084, 956, 855, 798, 745, 664.
HRMS (ESI) calcd for $C_{24}H_{21}O_3N_3Na$ $[M+Na]^+$: 422.1481. Found: 422.1480.
HPLC conditions: Chiralcel OD–H column (4.6 mm × 250 mm), hexane/*i*-PrOH, 80:20 v/v, flow rate 1 mL/min, λ = 254 nm, 25 °C. t_R (minor) = 26.79 min, t_R (major) = 49.94 min, ee > 99%. $[\alpha]_D^{31}$ = -112.4 (*c* = 1.0, $CHCl_3$).

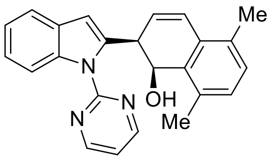

cis-**3ag**, 26.8 mg, 73% yield, pale yellow foam. **^{1}H NMR** (400 MHz, $CDCl_3$) δ 8.77 (d, *J* = 4.8 Hz, 2H), 8.29 (d, *J* = 8.0 Hz, 1H), 7.67-7.56 (m, 1H), 7.33-7.27 (m, 1H), 7.25-7.22 (m, 1H), 7.17 (td, *J* = 4.8, 1.2 Hz, 1H), 7.09-6.99 (m, 2H), 6.91-6.87 (m, 2H), 6.12 (dt, *J* = 10.0, 2.0 Hz, 1H), 5.26-5.24 (m, 1H), 4.93-4.91 (m, 1H), 2.37 (s, 3H), 2.35 (s, 3H). **^{13}C NMR** (100 MHz, $CDCl_3$) δ 158.4, 158.2, 139.7, 137.3, 134.0, 133.7, 131.9, 130.4, 130.2, 129.8, 129.2, 129.0, 124.7, 123.4, 122.3, 120.3, 117.5, 114.2, 108.8, 65.6, 41.7, 19.2, 18.5.
IR (thin film): ν_{max} (cm^{-1}) = 2922, 1562, 1452, 1421, 1345, 1259, 1193, 1077, 995, 907, 859, 805, 728, 679, 641, 449.
HRMS (ESI) calcd for $C_{24}H_{21}ON_3Na$ $[M+Na]^+$: 390.1582. Found: 390.1582.
HPLC conditions: Chiralcel OD–H column (4.6 mm × 250 mm), hexane/*i*-PrOH, 85:15 v/v, flow rate = 1 mL/min, λ = 254 nm, 25 °C. t_R (minor) = 9.88 min, t_R (major) = 23.49 min, ee = 98%. $[\alpha]_D^{31}$ = -94.5 (*c* = 1.0, $CHCl_3$).

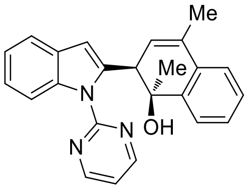

cis-**3ah**, 22.8 mg, 62% yield, pale yellow foam. **^{1}H NMR** (400 MHz, $CDCl_3$) δ 8.77 (d, *J* = 4.8 Hz, 2H), 8.12-8.10 (m, 1H), 7.86 (dd, *J* = 7.6, 1.2 Hz, 1H), 7.63-7.61 (m, 1H), 7.35 (td, *J* = 7.6, 1.2 Hz, 1H), 7.32-7.26 (m, 2H), 7.26-7.21 (m, 2H), 7.19 (t, *J* = 4.8 Hz, 1H), 6.81 (s, 1H), 6.32 (s, 1H), 5.89 (dd, *J* = 2.4, 1.6 Hz, 1H), 4.89 (t, *J* = 2.4 Hz, 1H), 2.10 (dd, *J* = 2.4, 1.6 Hz, 3H), 1.42 (s, 3H). **^{13}C NMR** (100 MHz, $CDCl_3$) δ 158.3, 157.2, 146.0, 140.5, 136.7, 133.7, 132.9, 129.6, 129.4, 128.2, 127.0, 124.0, 123.14, 123.10, 122.4, 120.2, 117.6, 114.0, 109.0, 75.5, 44.4, 24.1, 19.0.
IR (thin film): ν_{max} (cm^{-1}) = 3268, 2925, 1565, 1430, 1350, 1293, 1263, 1202, 1159, 1087, 1035, 958, 860, 802, 759, 746, 719, 666, 630, 579, 560, 538, 464, 442.
HRMS (ESI) calcd for $C_{24}H_{21}ON_3Na$ $[M+Na]^+$: 390.1582. Found: 390.1583.
HPLC conditions: Chiralcel AD–H column (4.6 mm × 250 mm), hexane/*i*-PrOH, 90:10 v/v, flow rate = 1 mL/min, λ = 254 nm, 25 °C. t_R (major) = 10.18 min, t_R (minor) = 14.50 min, ee = 52%. $[\alpha]_D^{31}$ = -6.5 (*c* = 1.0, $CHCl_3$).

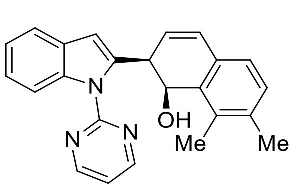

cis-**3ai**, 16.0 mg, 43% yield, pale yellow foam. **^{1}H NMR** (400 MHz, $CDCl_3$) δ 8.77 (d, *J* = 4.8 Hz, 2H), 8.28 (d, *J* = 8.0 Hz, 1H), 7.63-761 (m, 1H), 7.31-7.26 (m, 1H), 7.25-7.21 (m, 1H), 7.18 (t, *J* = 4.8 Hz, 1H), 7.13 (d, *J* = 7.6 Hz, 1H), 6.97 (d, *J* = 7.6 Hz, 1H), 6.89 (s, 1H), 6.63 (dd, *J* = 9.6, 3.2 Hz, 1H), 5.99 (dt, *J* = 9.6, 1.6 Hz, 1H), 5.33 (d, *J* = 4.4 Hz, 1H), 4.93-4.87 (m, 1H), 2.32 (s, 3H), 2.30 (s, 3H). **^{13}C NMR** (100 MHz, $CDCl_3$) δ 158.4, 158.1, 139.8, 137.4, 137.0, 135.2, 133.6, 130.2, 130.0, 129.2, 128.1, 127.6, 124.7, 123.4, 122.3, 120.3, 117.6, 114.2, 108.9, 65.6, 42.2, 20.9, 14.4.
IR (thin film): ν_{max} (cm^{-1}) = 2962, 1564, 1452, 1425, 1346, 1259, 1015, 790, 739, 593.
HRMS (ESI) calcd for $C_{24}H_{21}ON_3Na$ $[M+Na]^+$: 390.1582. Found: 390.1579.
HPLC conditions: Chiralcel AD–H column (4.6 mm × 250 mm), hexane/*i*-PrOH, 80:20 v/v, flow rate = 1 mL/min, λ = 254 nm, 25 °C. t_R (minor) = 25.23 min, t_R (major) = 36.13 min, ee = 97%. $[\alpha]_D^{35}$ = -259.9 (*c* = 1.0, $CHCl_3$).

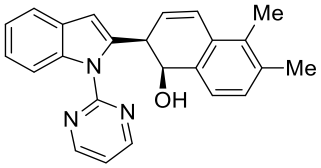

cis-**3ai'**, 11.8 mg, 32% yield, pale yellow foam. **^{1}H NMR** (400 MHz, $CDCl_3$) δ 8.85 (d, *J* = 4.8 Hz, 2H), 8.14 (d, *J* = 8.4 Hz, 1H), 7.45 (d, *J* = 7.6 Hz, 1H), 7.25-7.23 (m, 2H), 7.22-7.18 (m, 1H), 7.18-7.12 (m, 1H), 7.10 (d, *J* = 7.6 Hz, 1H), 6.98 (dd, *J* = 10.0, 1.6 Hz, 1H), 6.49 (s, 1H), 6.09 (dd, *J* = 10.0, 4.8 Hz, 1H), 5.25 (d, *J* = 6.8 Hz, 1H), 5.11 (br, 1H), 4.70 (ddd, *J* = 6.8, 4.8, 1.6 Hz, 1H), 2.35 (s, 3H), 2.33 (s, 3H). **^{13}C NMR** (100 MHz, $CDCl_3$) δ 158.5, 157.5, 137.1, 136.2, 134.3, 132.1, 130.1, 129.5, 129.3, 129.0, 125.1, 123.7, 123.2, 122.3, 120.4, 117.6, 113.9, 109.1, 103.5, 71.1, 38.8, 20.8, 14.8.
IR (thin film): ν_{max} (cm^{-1}) = 2962, 1565, 1452, 1427, 1347, 1301, 1259, 1014, 792, 749, 703.
HRMS (ESI) calcd for $C_{24}H_{21}ON_3Na$ $[M+Na]^+$: 390.1582. Found: 390.1568.
HPLC conditions: Chiralcel OD–H column (4.6 mm × 250 mm), hexane/*i*-PrOH, 85:15 v/v, flow rate = 1 mL/min, λ = 254 nm, 25 °C. t_R (minor) = 14.98 min, t_R (major) = 32.95 min, ee > 99%. $[\alpha]_D^{36}$ = +38.5 (*c* = 1.0, $CHCl_3$).

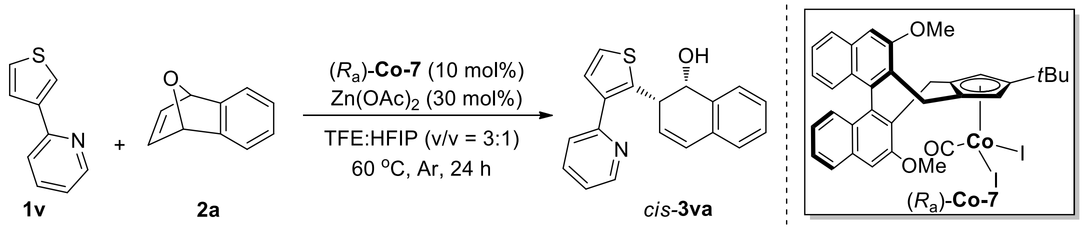

Procedure for the synthesis of *cis*-3va. A sealed tube with a magnetic stir bar was charged with (R_a)**-Co-7** (8.0 mg, 0.010 mmol), $Zn(OAc)_2$ (5.5 mg, 0.030 mmol), **1v** (16.1 mg, 0.10 mmol), **2a** (21.6 mg, 0.15 mmol), TFE (0.3 mL) and HFIP (0.1 mL) under argon atmosphere. The mixture was stirred at 60 °C for 24 h. Then, the mixture was cooled to room temperature and quenched by saturated NH_4Cl aqueous solution (20 mL). The resulting mixture was extracted with ethyl acetate (3 × 15 mL). The combined organic phase was dried over anhydrous Na_2SO_4 and filtered. All the volatiles were evaporated under reduced pressure. The remaining residue was purified by column chromatography on silica gel (hexanes/ethyl acetate = 8:1 to 4:1) to afford *cis*-**3va**.

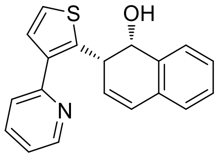

cis-**3va**, 9.8 mg, 32% yield, yellow oil. **^{1}H NMR** (400 MHz, $CDCl_3$) δ 8.61-8.52 (m, 1H), 7.87 (d, *J* = 7.6 Hz, 1H), 7.82 (td, *J* = 7.6, 1.6 Hz, 1H), 7.59 (d, *J* = 8.0 Hz, 1H), 7.41-7.32 (m, 2H), 7.28-7.24 (m, 3H), 7.11 (d, *J* = 7.2 Hz, 1H), 6.55 (dd, *J* = 9.6, 3.2 Hz, 1H), 6.05 (dd, *J* = 9.6, 2.4 Hz, 1H), 5.03 (d, *J* = 13.2 Hz, 1H), 4.66 (dt, *J* = 13.2, 2.8 Hz, 1H). **^{13}C NMR** (100 MHz, $CDCl_3$) δ 153.8, 148.0, 146.5, 140.1, 138.8, 137.8, 132.4, 130.8, 128.5, 128.3, 128.0, 127.2, 126.0, 125.0, 123.8, 123.6, 122.2, 76.1, 43.1.
IR (thin film): ν_{max} (cm^{-1}) = 3062, 2921, 2851, 2815, 1726, 1591, 1567, 1533, 1476, 1449, 1374, 1282, 1248, 1195, 1154, 1114, 1076, 1034, 1000, 959, 905, 859, 832, 784, 767, 721, 692, 672, 649, 628.
HRMS (ESI) calcd for $C_{19}H_{15}ONS$ $[M-H_2O+H]^+$: 288.0847. Found: 288.0844.
HPLC conditions: Chiralcel AD–H column (4.6 mm × 250 mm), hexane/*i*-PrOH, 90:10 v/v, flow rate = 1 mL/min, λ = 303 nm, 25 °C. t_R (major) = 9.92 min, t_R (minor) = 13.14 min, ee = 70%. $[\alpha]_D^{25}$ = -23.6 (*c* = 0.2, $CHCl_3$).

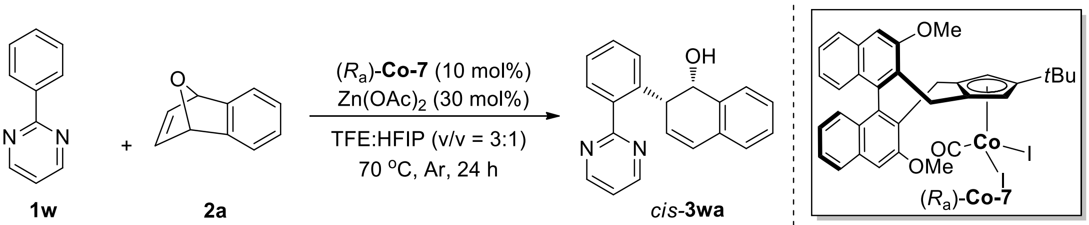

Procedure for the synthesis of *cis*-3wa. A sealed tube with a magnetic stir bar was charged with (R_a)**-Co-7** (8.0 mg, 0.010 mmol), $Zn(OAc)_2$ (5.5 mg, 0.030 mmol), **1w** (16.1 mg, 0.10 mmol), **2a** (21.6 mg, 0.15 mmol), TFE (0.3 mL) and HFIP (0.1 mL) under argon atmosphere. The mixture was stirred at 70 °C for 24 h. Then, the mixture was cooled to room temperature and quenched by saturated NH_4Cl aqueous solution (20 mL). The resulting mixture was extracted with ethyl acetate (3 × 15 mL). The combined organic phase was dried over anhydrous Na_2SO_4 and

filtered. All the volatiles were evaporated under reduced pressure. The remaining residue was purified by column chromatography on silica gel (hexanes/ethyl acetate = 8:1 to 4:1) to afford *cis*-**3wa**.

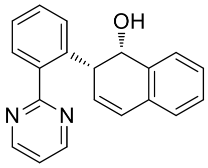

cis-**3wa**, 7.5 mg, 25% yield, yellow oil. **^{1}H NMR** (400 MHz, $CDCl_3$) δ 8.83 (d, *J* = 4.8 Hz, 2H), 7.85 (dd, *J* = 8.0, 1.6 Hz, 1H), 7.80 (d, *J* = 7.6 Hz, 1H), 7.75 (d, *J* = 8.0 Hz, 1H), 7.55 (td, *J* = 7.6, 1.6 Hz, 1H), 7.47-7.40 (m, 1H), 7.32 (t, *J* = 7.6 Hz, 1H), 7.28 (t, *J* = 4.8 Hz, 1H), 7.23 (t, *J* = 7.2 Hz, 1H), 7.06 (d, *J* = 7.2 Hz, 1H), 6.71 (d, *J* = 6.4 Hz, 1H), 6.51 (dd, *J* = 9.6, 2.8 Hz, 1H), 5.86 (dd, *J* = 9.6, 2.4 Hz, 1H), 5.18-5.05 (m, 1H), 4.41 (dt, *J* = 13.2, 2.4 Hz, 1H). **^{13}C NMR** (100 MHz, $CDCl_3$) δ 166.8, 157.3, 143.2, 140.1, 138.6, 133.0, 132.9, 131.4, 131.1, 129.5, 128.5, 128.4, 127.6, 127.4, 126.1, 125.0, 119.3, 76.4, 45.6.
IR (thin film): ν_{max} (cm^{-1}) = 3158, 3066, 2921, 2851, 2825, 1635, 1571, 1555, 1451, 1433, 1413, 1293, 1245, 1193, 1154, 1114, 1076, 1032, 996, 951, 902, 870, 826, 779, 755, 687, 648.
HRMS (ESI) calcd for $C_{20}H_{16}ON_2$ $[M-H_2O+H]^+$:283.1235. Found: 283.1233.
HPLC conditions: Chiralcel AD–H column (4.6 mm × 250 mm), hexane/*i*-PrOH, 85:15 v/v, flow rate = 1 mL/min, λ = 254 nm, 25 °C. t_R (major) = 9.57 min, t_R (minor) = 23.40 min, ee = 53%. $[\alpha]_D^{28}$ = -20.0 (*c* = 0.2, $CHCl_3$).

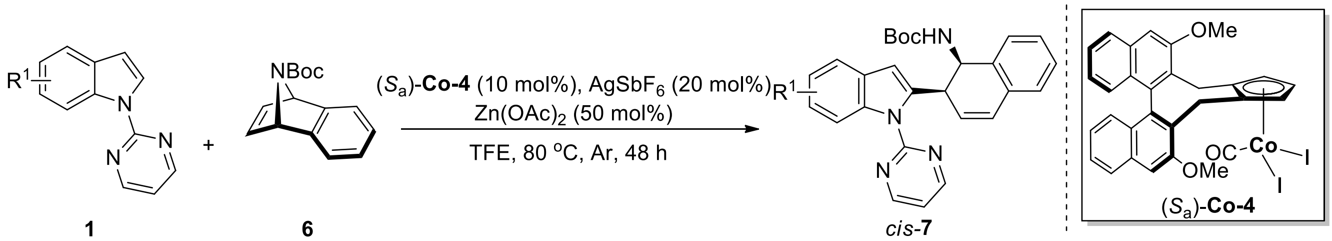

General procedure. A sealed tube with a magnetic stir bar was charged with (S_a)**-Co-4** (7.6 mg, 0.010 mmol), $AgSbF_6$ (6.8 mg, 0.020 mmol), $Zn(OAc)_2$ (9.2 mg, 0.050 mmol), **1** (0.15 mmol), **6** (24.3 mg, 0.10 mmol) and TFE (1.0 mL) under argon atmosphere. The mixture was stirred at 80 °C for 48 h. Then, the mixture was cooled to room temperature and quenched by saturated NH_4Cl aqueous solution (20 mL). The resulting mixture was extracted with ethyl acetate (3 × 15 mL). The combined organic phase was dried over anhydrous Na_2SO_4 and filtered. All the volatiles were evaporated under reduced pressure. The remaining residue was purified by column chromatography on silica gel (hexanes/ethyl acetate = 10:1 to 6:1) to afford *cis*-**7**.

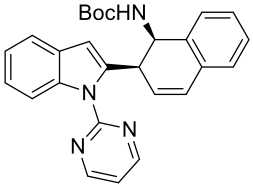

cis-**7aa**, 25.4 mg, 58% yield, yellow oil. **^{1}H NMR** (400 MHz, $CDCl_3$) δ 8.80 (d, *J* = 4.8 Hz, 2H), 8.30 (d, *J* = 8.4 Hz, 1H), 7.46 (d, *J* = 7.6 Hz, 1H), 7.30 (d, *J* = 8.4 Hz, 2H), 7.26-7.21 (m, 2H), 7.19-7.14 (m, 3H), 6.65 (d, *J* = 9.6 Hz, 1H), 6.48 (s, 1H), 6.29 (dd, *J* = 9.6, 4.0 Hz, 1H), 5.45 (dd, *J* = 10.0, 6.0 Hz, 1H), 5.13 (d, *J* = 5.6 Hz, 1H), 4.95 (d, *J* = 10.4 Hz, 1H), 1.20 (s, 9H). **^{13}C NMR** (100 MHz, $CDCl_3$) δ 158.4, 155.2, 138.8, 137.2, 135.4, 133.0, 130.5, 129.2, 128.2, 128.1, 128.0, 126.61, 126.56, 123.1, 122.0, 120.1, 117.3, 114.4, 107.6, 79.3, 51.4, 38.7, 28.3.
IR (thin film): ν_{max} (cm^{-1}) = 2962, 2920, 1707, 1616, 1562, 1485, 1421, 1345, 1262, 1157, 1098, 1027, 923, 860, 804, 629, 527, 478, 442.
HRMS (ESI) calcd for $C_{27}H_{26}O_2N_4Na$ $[M+Na]^+$: 461.1953. Found: 461.1942.
HPLC conditions: Chiralcel AD–3 column (4.6 mm × 250 mm), hexane/*i*-PrOH, 80:20 v/v, flow rate = 0.7 mL/min, λ = 254 nm, 30 °C. t_R (major) = 9.84 min, t_R (minor) = 15.27 min, ee = 96%. $[\alpha]_D^{28}$ = -26.1 (*c* = 0.5, $CHCl_3$).

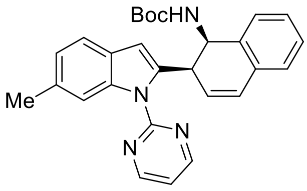

cis-**7ab**, 28.5 mg, 63% yield, yellow oil. **^{1}H NMR** (400 MHz, $CDCl_3$) δ 8.83 (d, *J* = 4.8 Hz, 2H), 8.11 (s, 1H), 7.36 (d, *J* = 8.0 Hz, 1H), 7.33-7.29 (m, 2H), 7.28-7.24 (m, 1H), 7.21-7.15 (m, 2H), 7.02 (d, *J* = 8.0 Hz, 1H), 6.70-6.62 (m, 1H), 6.44 (s, 1H), 6.31 (dd, *J* = 10.0, 4.4 Hz, 1H), 5.45 (dd, *J* = 10.0, 6.4 Hz, 1H), 5.10 (d, *J* = 6.0 Hz, 1H), 4.97 (d, *J* = 10.0 Hz, 1H), 2.50 (s, 3H), 1.25 (s, 9H). **^{13}C NMR** (100 MHz, $CDCl_3$) δ 158.38, 158.34, 155.3, 138.0, 137.5, 135.5, 133.0, 132.9, 130.6, 128.1, 127.9, 127.8, 126.9, 126.5, 126.4, 123.5, 119.7, 117.2, 114.2, 107.4, 79.3, 51.5, 38.5, 28.3, 22.2.
IR (thin film): v_{max} (cm^{-1}) = 2976, 2922, 1705, 1564, 1487, 1422, 1365, 1340, 1226, 1161, 1054, 907, 861, 813, 777, 727, 645.
HRMS (ESI) calcd for $C_{28}H_{28}O_2N_4Na$ $[M+Na]^+$: 475.2110. Found: 475.2111.
HPLC conditions: Chiralcel IG column (4.6 mm × 250 mm), hexane/*i*-PrOH, 90:10 v/v, flow rate = 0.7 mL/min, λ = 254 nm, 30 °C. t_R (major) = 21.63 min, t_R (minor) = 23.96 min, ee = 95%. $[\alpha]_D^{30}$ = -46.1 (*c* = 0.5, $CHCl_3$).

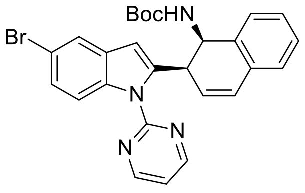

cis-**7ac**, 31.0 mg, 60% yield, yellow oil. **^{1}H NMR** (400 MHz, $CDCl_3$) δ 8.78 (d, *J* = 4.8 Hz, 2H), 8.21 (d, *J* = 8.8 Hz, 1H), 7.58 (s, 1H), 7.34-7.21 (m, 4H), 7.19-7.12 (m, 2H), 6.65 (dd, *J* = 9.6, 2.4 Hz, 1H), 6.43 (s, 1H), 6.22 (dd, *J* = 9.6, 4.0 Hz, 1H), 5.43 (dd, *J* = 10.4, 6.0 Hz, 1H), 5.15-5.12 (m, 1H), 4.88 (d, *J* = 10.4 Hz, 1H), 1.17 (s, 9H). **^{13}C NMR** (100 MHz, $CDCl_3$) δ 158.3, 158.0, 154.9, 140.5, 135.8, 135.1, 132.7, 130.9, 123.0, 128.2, 128.1, 128.0, 126.9, 126.5, 125.6, 122.3, 117.5, 116.2, 115.0, 106.8, 79.3, 51.1, 39.1, 28.2.
IR (thin film): v_{max} (cm^{-1}) = 3421, 2975, 2928, 1705, 1563, 1489, 1443, 1419, 1364, 1334, 1292, 1259, 1226, 1158, 1055, 1015, 990, 944, 906, 862, 795, 775, 733, 699, 675, 645, 632.
HRMS (ESI) calcd for $C_{27}H_{25}O_2N_4BrNa$ $[M+Na]^+$: 539.1059. Found: 539.1050.
HPLC conditions: Chiralcel IG column (4.6 mm × 250 mm), hexane/*i*-PrOH, 80:20 v/v, flow rate = 0.7 mL/min, λ = 254 nm, 30 °C. t_R (minor) = 16.85 min, t_R (major) = 24.92 min, ee = 95%. $[\alpha]_D^{29}$ = -74.2 (*c* = 0.2, $CHCl_3$).

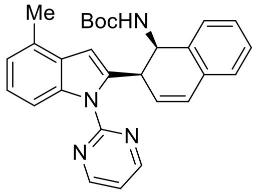

cis-**7ad**, 27.6 mg, 61% yield, yellow oil. **^{1}H NMR** (400 MHz, $CDCl_3$) δ 8.79 (d, *J* = 4.8 Hz, 2H), 8.13 (d, *J* = 8.4 Hz, 1H), 7.30 (t, *J* = 6.0 Hz, 2H), 7.25-7.10 (m, 4H), 6.97 (d, *J* = 7.2 Hz, 1H), 6.66 (dd, *J* = 9.6, 2.4 Hz, 1H), 6.52 (s, 1H), 6.27 (dd, *J* = 9.6, 4.0 Hz, 1H), 5.43 (dd, *J* = 10.4, 6.4 Hz, 1H), 5.15 (s, 1H), 4.95 (d, *J* = 10.4 Hz, 1H), 2.47 (s, 3H), 1.17 (s, 9H). **^{13}C NMR** (100 MHz, $CDCl_3$) δ 158.5, 158.3, 155.1, 138.3, 137.0, 135.4, 132.9, 130.6, 129.2, 128.8, 128.1, 128.0, 126.9, 126.5, 123.1, 122.3, 117.3, 112.0, 106.1, 79.2, 51.4, 39.0, 28.3, 18.7.
IR (thin film): v_{max} (cm^{-1}) = 2975, 2923, 1705, 1564, 1488, 1424, 1364, 1345, 1299, 1281, 1247, 1227, 1157, 1106, 1089, 1054, 1026, 907, 860, 806, 769, 728, 640, 619.
HRMS (ESI) calcd for $C_{28}H_{28}O_2N_4Na$ $[M+Na]^+$: 475.2110. Found: 475.2108.
HPLC conditions: Chiralcel AD-H column (4.6 mm × 250 mm), hexane/*i*-PrOH, 90:10 v/v, flow rate = 0.7 mL/min, λ = 254 nm, 30 °C. t_R (major) = 11.17 min, t_R (minor) = 18.74 min, ee = 96%. $[\alpha]_D^{29}$ = -33.2 (*c* = 0.5, $CHCl_3$).

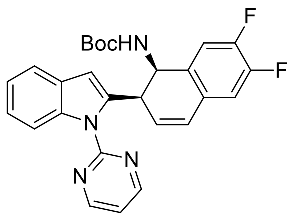

cis-**7ae**, 26.1 mg, 55% yield, yellow oil. **^{1}H NMR** (400 MHz, $CDCl_3$) δ 8.82 (d, *J* = 4.8 Hz, 2H), 8.29 (d, *J* = 8.4 Hz, 1H), 7.47 (d, *J* = 7.6 Hz, 1H), 7.26-7.21 (m, 1H), 7.21-7.16 (m, 2H), 7.11 (dd, *J* = 10.8, 7.6 Hz, 1H), 6.97 (dd, *J* = 10.8, 7.6 Hz, 1H), 6.53 (d, *J* = 9.6 Hz, 1H), 6.42 (s, 1H), 6.32 (dd, *J* = 9.6, 4.8 Hz, 1H), 5.39 (t, *J* = 8.4 Hz, 1H), 5.09 (d, *J* = 6.0 Hz, 1H), 4.94 (d, *J* = 10.0 Hz, 1H), 1.23 (s, 9H). **^{13}C NMR** (100 MHz, $CDCl_3$) δ 158.44, 158.25, 155.23, 149.85 (dd, *J* = 248, 7.4 Hz), 149.7 (dd, *J* = 248, 7.4 Hz), 137.7, 137.2, 132.4-132.3 (m), 131.22, 129.9-129.8 (m), 126.2, 123.4, 122.2, 120.2, 117.5, 115.9 (d, *J* = 18.8 Hz), 115.3 (d, *J* = 18.8 Hz), 114.4, 107.6, 79.8, 51.0, 37.9, 29.9, 28.3. **^{19}F NMR** (376 MHz, $CDCl_3$) δ -138.14 (d, *J* = 20.8 Hz), -140.16 (d, *J* = 20.8 Hz).

IR (thin film): v_{max} (cm^{-1}) = 2925, 2854, 2350, 1708, 1577, 1564, 1504, 1454, 1427, 1367, 1347, 1309, 1248, 1164, 1083, 1050, 1021, 883, 806, 784, 749, 663, 637, 619.
HRMS (ESI) calcd for $C_{27}H_{24}F_2N_4NaO_2$ $[M+Na]^+$: 497.1765. Found: 497.1763.
HPLC conditions: [Waters upc, SFC system] Chiralcel IG-3 column (4.6 mm × 250 mm), CO_2/EtOH, 80:20 v/v, flow rate = 1.0 mL/min, λ = 210 nm, 20 °C. t_R (major) = 14.46 min, t_R (minor) = 17.08 min, ee = 94%. $[\alpha]_D^{30}$ = -72.1 (*c* = 0.2, $CHCl_3$).

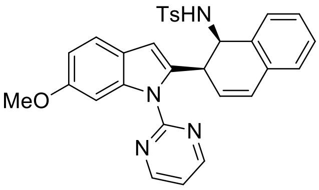

cis-**7af**, 26.1 mg, 50% yield, pale yellow foam. **^{1}H NMR** (400 MHz, $CDCl_3$) 8.74 (d, *J* = 4.8 Hz, 2H), 7.88 (d, *J* = 2.4 Hz, 1H), 7.43 (d, *J* = 7.2 Hz, 1H), 7.32-7.21 (m, 5H), 7.19-7.10 (m, 2H), 6.85 (dd, *J* = 8.4, 2.4 Hz, 1H), 6.67 (d, *J* = 8.0 Hz, 2H), 6.61 (dd, *J* = 9.6, 2.4 Hz, 1H), 6.35 (s, 1H), 6.11 (dd, *J* = 9.6, 3.6 Hz, 1H), 5.41 (d, *J* = 8.8 Hz, 1H), 5.11 (dd, *J* = 8.8, 6.0 Hz, 1H), 4.82 (dt, *J* = 5.6, 2.8 Hz, 1H), 3.89 (s, 3H), 2.14 (s, 3H). The analytical data are in accordance with those of the previous report.[12]
HPLC conditions: Chiralcel AD–H column (4.6 mm × 250 mm), hexane/*i*-PrOH, 70:30 v/v, flow rate = 1.0 mL/min, λ = 254 nm, 25 °C. t_R (major) = 12.31 min, t_R (minor) = 16.44 min, ee = 91%. $[\alpha]_D^{29}$ = +39.9 (*c* = 0.5, $CHCl_3$).

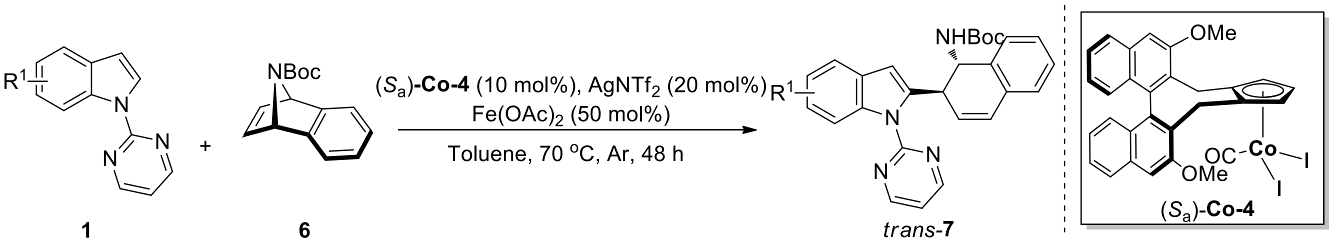

General procedure. A sealed tube with a magnetic stir bar was charged with (S_a)**-Co-4** (7.6 mg, 0.010 mmol), $AgNTf_2$ (7.8 mg, 0.020 mmol), $Fe(OAc)_2$ (8.7 mg, 0.050 mmol), **1** (0.15 mmol), **6** (24.3 mg, 0.10 mmol) and toluene (1.0 mL) under argon atmosphere. The mixture was stirred at 70 °C for 48 h. Afterwards, the mixture was cooled to room temperature and quenched by saturated NH_4Cl aqueous solution (20 mL). The resulting mixture was extracted with ethyl acetate (3 × 15 mL). The combined organic phase was dried over anhydrous Na_2SO_4 and filtered. All the volatiles were evaporated under reduced pressure. The remaining residue was purified by column chromatography on silica gel (hexanes/ethyl acetate = 10:1 to 6:1) to afford *trans*-**7**.

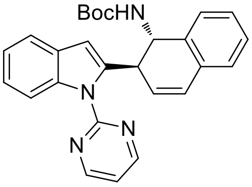

trans-**7aa**, 23.2 mg, 53% yield, yellow oil. **^{1}H NMR** (400 MHz, $CDCl_3$) δ 8.82 (d, *J* = 4.8 Hz, 2H), 8.23 (d, *J* = 8.4 Hz, 1H), 7.44 (d, *J* = 7.6 Hz, 1H), 7.30-7.25 (m, 1H), 7.23-7.18 (m, 3H), 7.15 (d, *J* = 7.6 Hz, 2H), 6.65 (d, *J* = 9.6 Hz, 1H), 6.47 (s, 1H), 6.11 (dd, *J* = 9.6, 4.4 Hz, 1H), 5.47 (d, *J* = 9.2 Hz, 1H), 5.24 (t, *J* = 7.2 Hz, 1H), 4.90 (t, *J* = 5.2 Hz, 1H), 1.35 (s, 9H). **^{13}C NMR** (100 MHz, $CDCl_3$) δ 158.5, 158.1, 155.2, 139.3, 137.4, 135.2, 132.5, 129.14, 129.09, 128.3, 128.1, 128.0, 127.9, 126.5, 123.1, 122.0, 120.3, 117.5, 114.0, 107.0, 79.3, 53.5, 40.2, 28.5.
IR (thin film): v_{max} (cm^{-1}) = 3041, 2974, 2928, 2246, 1704, 1562, 1489, 1452, 1423, 1346, 1259, 1158, 1054, 1016, 908, 859, 792, 728, 644, 530, 477.
HRMS (ESI) calcd for $C_{27}H_{26}O_2N_4Na$ $[M+Na]^+$: 461.1953. Found: 461.1938.
HPLC conditions: Chiralcel AD-3 column (4.6 mm × 250 mm), hexane/*i*-PrOH, 80:20 v/v, flow rate = 0.7 mL/min, λ = 254 nm, 30 °C. t_R (major) = 10.32 min, t_R (minor) = 16.41 min, ee = 82%. $[\alpha]_D^{32}$ = -167.9 (*c* = 0.5, $CHCl_3$).

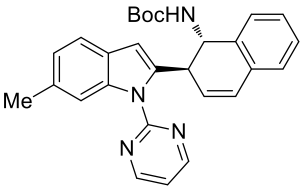

trans-**7ab**, 25.8 mg, 57% yield, yellow foam. **^{1}H NMR** (400 MHz, $CDCl_3$) δ 8.83 (d, *J* = 4.8 Hz, 2H), 8.03 (s, 1H), 7.31 (d, *J* = 8.0 Hz, 1H), 7.26-7.16 (m, 3H), 7.16-7.11 (m, 1H), 6.97 (d, *J* = 8.0 Hz, 1H), 6.63 (d, *J* = 9.6 Hz, 1H), 6.42 (s, 1H), 6.10 (dd, *J* = 9.6, 4.8 Hz, 1H), 5.49 (d, *J* = 9.2 Hz, 1H), 5.29-5.11 (m, 1H), 4.90-4.80 (m, 1H), 2.46 (s, 3H), 1.36 (s, 9H). **^{13}C NMR** (100 MHz, $CDCl_3$) δ 158.4, 158.1, 155.2, 138.5, 137.7, 135.5, 135.2, 132.9, 132.5, 129.2, 128.3, 128.1, 127.9, 126.8, 126.5, 123.5, 119.9, 117.3, 113.9, 106.9, 79.2, 53.5, 40.1, 28.5, 22.2.
IR (thin film): ν_{max} (cm^{-1}) = 2972, 2921, 1704, 1564, 1486, 1451, 1421, 1365, 1341, 1262, 1161, 1099, 1042, 1017, 909, 865, 806, 781, 761, 732, 698, 629, 597, 533, 489, 465, 438.
HRMS (ESI) calcd for $C_{28}H_{28}O_2N_4Na$ $[M+Na]^+$: 475.2110. Found: 475.2109.
HPLC conditions: Chiralcel AD-3 column (4.6 mm × 250 mm), hexane/*i*-PrOH, 90:10 v/v, flow rate = 0.7 mL/min, λ = 230 nm, 30 °C. t_R (major) = 14.63 min, t_R (minor) = 23.67 min, ee = 82%. $[\alpha]_D^{32}$ = -137.0 (*c* = 0.5, $CHCl_3$).

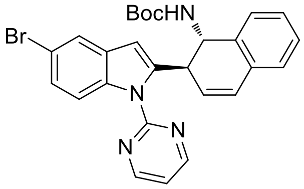

trans-**7ac**, 27.4 mg, 53% yield, yellow oil. **^{1}H NMR** (400 MHz, $CDCl_3$) δ 8.82 (d, *J* = 4.8 Hz, 2H), 8.12 (d, *J* = 8.8 Hz, 1H), 7.54 (s, 1H), 7.32-7.27 (m, 1H), 7.25-7.17 (m, 3H), 7.14 (d, *J* = 7.2 Hz, 1H), 6.66 (d, *J* = 9.6 Hz, 1H), 6.39 (s, 1H), 6.09 (dd, *J* = 9.6, 4.8 Hz, 1H), 5.36 (d, *J* = 9.6 Hz, 1H), 5.25-5.12 (m, 1H), 4.91 (t, *J* = 5.2 Hz, 1H), 1.35 (s, 9H). **^{13}C NMR** (100 MHz, $CDCl_3$) δ 158.4, 157.8, 155.0, 140.5, 137.7, 134.7, 132.3, 130.6, 128.4, 128.3, 128.2, 128.1, 127.9, 126.5, 125.7, 122.7, 117.6, 115.5, 115.0, 106.1, 79.2, 53.2, 40.1, 28.3.
IR (thin film): ν_{max} (cm^{-1}) = 3062, 2920, 2851, 2815, 1726, 1695, 1591, 1568, 1533, 1476, 1450, 1375, 1319, 1282, 1248, 1195, 1154, 1114, 1076, 1034, 1001, 960, 905, 859, 832, 784, 767, 721, 693, 672, 649, 628.
HRMS (ESI) calcd for $C_{27}H_{25}O_2N_4BrNa$ $[M+Na]^+$: 539.1059. Found: 539.1049.
HPLC conditions: Chiralcel IG column (4.6 mm × 250 mm), hexane/*i*-PrOH, 80:20 v/v, flow rate = 0.7 mL/min, λ = 254 nm, 30 °C. t_R (major) = 17.88 min, t_R (minor) = 21.13 min, ee = 86%. $[\alpha]_D^{30}$ = -169.6 (*c* = 0.8, $CHCl_3$).

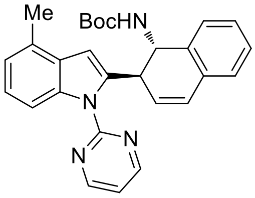

trans-**7ad**, 24.9 mg, 55% yield, yellow oil. **^{1}H NMR** (400 MHz, $CDCl_3$) δ 8.82 (d, *J* = 4.8 Hz, 2H), 8.05 (d, *J* = 8.4 Hz, 1H), 7.28 (d, *J* = 9.6 Hz, 1H), 7.26-7.16 (m, 3H), 7.16-7.09 (m, 2H), 6.95 (d, *J* = 7.2 Hz, 1H), 6.64 (d, *J* = 9.6 Hz, 1H), 6.53 (s, 1H), 6.10 (dd, *J* = 9.6, 4.4 Hz, 1H), 5.55 (d, *J* = 9.2 Hz, 1H), 5.25 (t, *J* = 8.0 Hz, 1H), 4.89 (ddd, *J* = 6.6, 4.4, 1.6 Hz, 1H), 2.44 (s, 3H), 1.36 (s, 9H). **^{13}C NMR** (100 MHz, $CDCl_3$) δ 158.4, 158.2, 155.3, 138.8, 137.1, 135.4, 132.6, 129.7, 129.4, 128.7, 128.2, 128.0, 127.9, 127.6, 126.5, 123.1, 122.3, 117.5, 111.4, 105.3, 79.2, 53.7, 40.1, 28.5, 18.7.
IR (thin film): ν_{max} (cm^{-1}) = 2969, 2922, 2854, 1699, 1564, 1488, 1424, 1364, 1345, 1245, 1157, 1089, 1072, 1043, 1015, 988, 907, 858, 802, 768, 728, 639.
HRMS (ESI) calcd for $C_{28}H_{28}O_2N_4Na$ $[M+Na]^+$: 475.2110. Found: 475.2105.
HPLC conditions: Chiralcel AD-H column (4.6 mm × 250 mm), hexane/*i*-PrOH, 90:10 v/v, flow rate = 0.7 mL/min, λ = 254 nm, 30 °C. t_R (major) = 15.18 min, t_R (minor) = 20.53 min, ee = 77%. $[\alpha]_D^{27}$ = -270.1 (*c* = 0.3, $CHCl_3$).

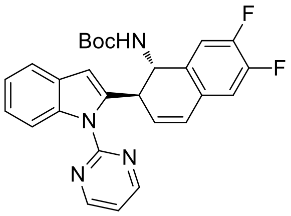

trans-**7ae**, 24.2 mg, 51% yield, yellow oil. **^{1}H NMR** (400 MHz, $CDCl_3$) δ 8.83 (d, *J* = 4.8 Hz, 2H), 8.25 (d, *J* = 8.4 Hz, 1H), 7.47 (d, *J* = 7.6 Hz, 1H), 7.25-7.18 (m, 2H), 7.16 (t, *J* = 7.6 Hz, 1H), 7.13-7.08 (m, 1H), 6.95 (dd, *J* = 10.4, 7.6 Hz, 1H), 6.54 (d, *J* = 9.2 Hz, 1H), 6.49 (s, 1H), 6.13 (dd, *J* = 9.6, 4.4 Hz, 1H), 5.64 (d, *J* = 9.2 Hz, 1H), 5.20 (t, *J* = 8.0 Hz, 1H), 4.84 (t, *J* = 5.6 Hz, 1H), 1.35 (s, 9H). **^{13}C NMR** (100 MHz, $CDCl_3$) δ 158.4, 157.9, 155.1, 149.78 (dd, *J* = 247.1, 13.0 Hz), 149.55 (dd, *J* = 247.1, 13.0 Hz), 138.5, 137.2, 132.2-132.1 (m), 130.0, 129.2-129.1 (m), 126.2, 123.2, 122.1, 120.3, 117.4, 117.0 (d, *J* = 18.0 Hz), 115.0 (d, *J* = 18.0 Hz), 114.0, 107.1, 79.5, 53.1, 39.5, 29.7, 28.3. **^{19}F NMR** (376 MHz, $CDCl_3$) δ -138.07 (d, *J* = 20.8 Hz), -139.89 (d, *J* = 20.8 Hz).

IR (thin film): ν_{max} (cm^{-1}) = 2963, 2924, 1703, 1565, 1505, 1455, 1427, 1390, 1367, 1347, 1308, 1260, 1164, 1098, 1049, 1020, 883, 802, 749, 698, 682, 665, 638, 619.
HRMS (ESI) calcd for $C_{27}H_{24}F_2N_4NaO_2$ $[M+Na]^+$: 497.1765. Found: 497.1769.
HPLC conditions: [Waters upc, SFC system] Chiralcel IG-3 column (4.6 mm × 250 mm), CO_2/EtOH, 80:20 v/v, flow rate = 1.0 mL/min, λ = 210 nm, 20 °C. t_R (major) = 13.58 min, t_R (minor) = 18.45 min, ee = 63%. $[\alpha]_D^{31}$ = -168.4 (*c* = 0.2, $CHCl_3$).

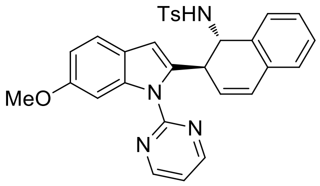

trans-**7af**, 23.0 mg, 44% yield, pale foam. **^{1}H NMR** (400 MHz, $CDCl_3$) δ 8.89 (d, *J* = 4.8 Hz, 2H), 7.97 (d, *J* = 6.4 Hz, 1H), 7.73-7.61 (m, 2H), 7.28 (d, *J* = 4.8 Hz, 1H), 7.26-7.24 (m, 1H), 7.21 (d, *J* = 8.0 Hz, 2H), 7.13 (d, *J* = 8.4 Hz, 1H), 7.10-7.04 (m, 1H), 6.83 (dd, *J* = 8.4, 2.4 Hz, 1H), 6.62 (d, *J* = 8.0 Hz, 2H), 6.50 (dd, *J* = 9.6, 2.4 Hz, 1H), 6.14 (s, 1H), 5.87 (dd, *J* = 9.6, 2.8 Hz, 1H), 4.77 (dd, *J* = 12.0, 6.4 Hz, 1H), 4.52 (dt, *J* = 12.0, 2.8 Hz, 1H), 3.89 (s, 3H), 2.14 (s, 3H). **^{13}C NMR** (100 MHz, $CDCl_3$) δ 158.8, 157.24, 157.15, 142.5, 139.2, 137.3, 137.1, 135.7, 132.8, 131.3, 129.0, 128.3, 128.0, 127.9, 127.4, 126.4, 126.2, 123.5, 120.8, 117.9, 111.2, 107.4, 98.3, 59.6, 56.0, 39.2, 21.7.
IR (thin film): ν_{max} (cm^{-1}) = 3281, 2961, 2833, 2252, 1616, 1564, 1485, 1421, 1344, 1291, 1266, 1236, 1199, 1150, 1112, 1078, 1020, 953, 906, 807, 771, 727, 687, 659, 629, 580, 547, 522, 443.
HRMS (ESI) calcd for $C_{30}H_{26}N_4NaO_3S$ $[M+Na]^+$: 545.1623. Found: 545.1615.
HPLC conditions: Chiralcel AD–H column (4.6 mm × 250 mm), hexane/*i*-PrOH, 70:30 v/v, flow rate = 1.0 mL/min, λ = 254 nm, 25 °C. t_R (major) = 12.61 min, t_R (minor) = 14.23 min, ee = 63%. $[\alpha]_D^{29}$ = -45.9 (*c* = 0.2, $CHCl_3$).

1.3 Gram-scale reaction and derivatization reactions

Gram-scale reaction

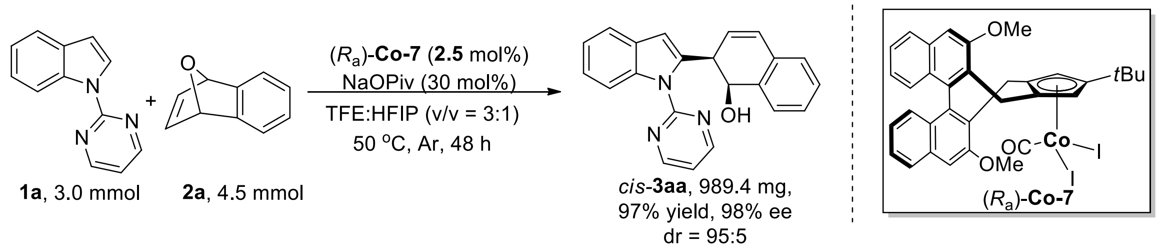

A 25 mL sealed tube with a magnetic stir bar was charged with (R_a)**-Co-7** (60.1 mg, 0.075 mmol), NaOPiv·H_2O (127.9 mg, 0.9 mmol), **1a** (585.6 mg, 3.0 mmol), **2a** (648.9 mg, 4.5 mmol), TFE (9.0 mL) and HFIP (3.0 mL) under argon atmosphere. The mixture was stirred at 50 °C for 48 h. Then, the mixture was cooled to room temperature and quenched by saturated NH_4Cl aqueous solution (50 mL). The resulting mixture was extracted with ethyl acetate (3 × 30 mL). The combined organic phase was dried over anhydrous Na_2SO_4 and filtered. All the volatiles were evaporated under reduced pressure. The remaining residue was purified by column chromatography on silica gel (hexanes/ethyl acetate = 8:1 to 4:1) to afford *cis*-**3aa** in 97% yield and 98% ee (dr = 95:5).

Derivatization reactions

(a) Synthesis of compound **4**

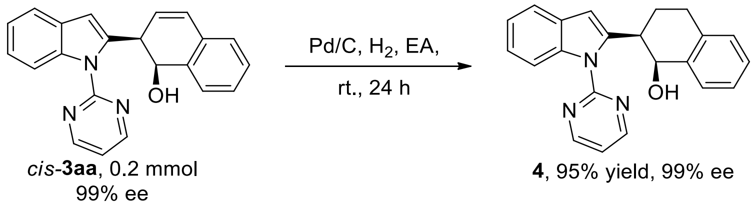

A 25 mL sealed tube with a magnetic stir bar was charged with *cis*-**3aa** (67.9 mg, 0.2 mmol), Pd/C (20.0 mg, palladium on activated carbon, 10% Pd basis, 0.1 equiv) and EtOAc (2 mL) under argon atmosphere. Then, the reaction mixture was exchanged with H_2 atmosphere (1 atm) and stirred at rt for 24 h. After completion (monitored by TLC), the crude reaction mixture was filtered with celite and washed with EtOAc. The solution was concentrated by rotary evaporation. After that, the residue was purified by column chromatography on silica gel (petroleum ether/ethyl acetate = 8/1 to 4/1) to afford **4**.

4, 64.9 mg, 95% yield, yellow oil. **^{1}H NMR** (400 MHz, $CDCl_3$) δ 8.78 (d, *J* = 4.8 Hz, 2H), 8.21 (d, *J* = 8.4 Hz, 1H), 7.59 (d, *J* = 7.2 Hz, 1H), 7.35 (d, *J* = 6.4 Hz, 1H), 7.26-7.14 (m, 6H), 6.69 (s, 1H), 5.01-4.88 (m, 1H), 4.18 (dt, *J* = 12.4, 3.2 Hz, 1H), 3.13-2.86 (m, 2H), 2.49 (qd, *J* = 12.4, 5.6 Hz, 1H), 2.20-1.99 (m, 2H). **^{13}C NMR** (100 MHz, $CDCl_3$) δ 158.5, 158.3, 141.8, 137.6, 137.3, 136.8, 130.6, 129.2, 129.1, 128.1, 126.2, 123.3, 122.2, 120.3, 117.6, 113.8, 107.2, 69.0, 39.4, 29.5, 23.1.
IR (thin film): v_{max} (cm^{-1}) = 2931, 1562, 1453, 1422, 1347, 1210, 1084, 1054, 952, 806, 740, 666, 640, 442.
HRMS (ESI) calcd for $C_{22}H_{19}ON_3Na$ $[M+Na]^+$: 364.1426. Found: 364.1423.
HPLC conditions: Chiralcel AD–H column (4.6 mm × 250 mm), hexane/*i*-PrOH, 80:20 v/v, flow rate = 1 mL/min, λ = 254 nm, 25 °C. t_R (minor) = 24.11 min, t_R (major) = 30.97 min, ee > 99%. $[\alpha]_D^{34}$ = -506.5 (*c* = 1.0, $CHCl_3$).

(b) Synthesis of compound **5**

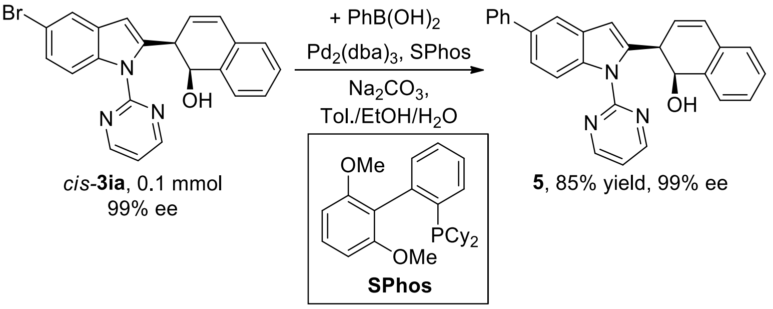

A sealed tube was filled with argon. To this flask were added Pd_2dba_3 (2.4 mg, 0.0025 mmol, 2.5 mol%), SPhos (4.2 mg, 0.01 mmol, 10 mol%), *cis*-**3ia** (41.8 mg, 0.1 mmol, 1.0 equiv), $PhB(OH)_2$ (24.4 mg, 0.2 mmol, 2.0 equiv), Na_2CO_3 (5.3 mg, 0.05 mmol, 0.5 equiv) and toluene/EtOH/H_2O (0.75 mL/0.25 mL/0.25 mL). The reaction was sealed with Teflon plug and stirred at 80 °C for 6 h. After completion (monitored by TLC), the reaction was quenched by saturated NH_4Cl aqueous solution (15 mL), and then extracted with EtOAc (3 × 15 mL). The combined organic fractions were dried over Na_2SO_4, filtered and concentrated by rotary evaporation. The residue was purified by column chromatography on silica gel (petroleum ether/ethyl acetate = 8/1 to 4/1) to afford **5**.

5, 35.2 mg, 85% yield, pale yellow foam. **^{1}H NMR** (400 MHz, $CDCl_3$) δ 8.88 (d, *J* = 4.8 Hz, 2H), 8.19 (d, *J* = 8.8 Hz, 1H), 7.64 (d, *J* = 1.6 Hz, 1H), 7.60 (dt, *J* = 6.4, 1.2 Hz, 2H), 7.54-7.49 (m, 1H), 7.46 (dd, *J* = 8.8, 2.0 Hz, 1H), 7.41 (t, *J* = 7.8 Hz, 2H), 7.33-7.27 (m, 4H), 7.22-7.18 (m, 1H), 6.68 (dd, *J* = 9.6, 1.6 Hz, 1H), 6.49 (s, 1H), 6.05 (dd, *J* = 9.6, 5.2 Hz, 1H), 5.65-5.57 (m, 1H), 5.41 (d, *J* = 7.2 Hz, 1H), 4.74 (ddd, *J* = 7.2, 5.2, 1.6 Hz, 1H). **^{13}C NMR** (100 MHz, $CDCl_3$) δ 158.4, 157.3, 142.0, 137.2, 136.6, 136.3, 135.5, 132.0, 129.6, 128.8, 128.6, 128.3, 127.9, 127.6, 127.3, 126.6, 125.9, 125.9, 122.7, 118.7, 117.6, 114.1, 109.4, 70.6, 38.8.
IR (thin film): v_{max} (cm^{-1}) = 3240, 1602, 1571, 1421, 1345, 1180.21, 1086, 1024, 791, 759, 695, 637, 579.
HRMS (ESI) calcd for $C_{28}H_{21}ON_3Na$ $[M+Na]^+$: 438.1582. Found: 438.1586.
HPLC conditions: Chiralcel OD–H column (4.6 mm × 250 mm), hexane/*i*-PrOH, 80:20 v/v, flow rate = 1 mL/min, λ = 254 nm, 25 °C. t_R (minor) = 15.21 min, t_R (major) = 25.83 min, ee > 99%. $[\alpha]_D^{33}$ = +229.83 (*c* = 1.0, $CHCl_3$).

(c) Attempts to remove the pyrimidine group of the ARO products.

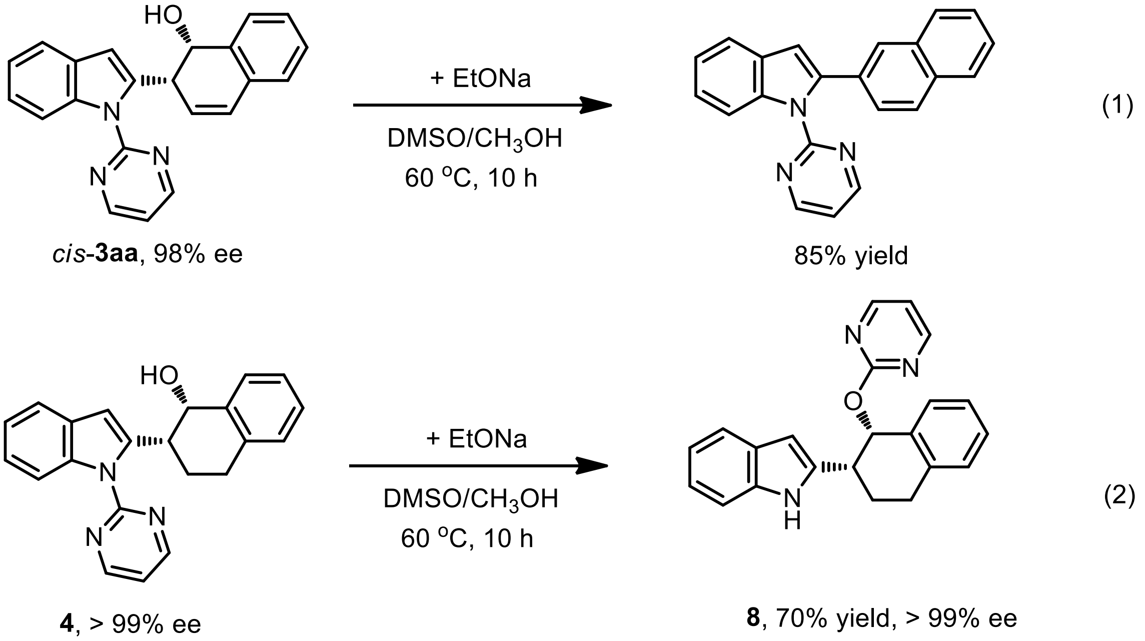

Eq.1: A 10 mL sealed tube with a magnetic stir bar was charged with *cis*-**3aa** (33.9 mg, 0.1 mmol), NaOEt (20.4 mg, 0.3 mmol), MeOH (0.1 mL) and DMSO (0.3 mL). Then the reaction was sealed with Teflon plug and stirred at 60 °C for 10 h. After completion (monitored by TLC), the reaction was quenched by saturated NH_4Cl aqueous solution (15 mL), and then extracted with EtOAc (3 × 15 mL). The combined organic fractions were dried over Na_2SO_4, filtered and concentrated by rotary evaporation. The residue was purified by column chromatography on silica gel (petroleum ether/ethyl acetate = 20/1 to 10/1) to afford the aromatization product in 85% yield (27.3 mg).

^{1}H NMR (400 MHz, $CDCl_3$) δ 8.67 (d, *J* = 4.8 Hz, 2H), 8.23 (d, *J* = 8.4 Hz, 1H), 7.96-7.89 (m, 1H), 7.86-7.78 (m, 2H), 7.74 (d, *J* = 8.4 Hz, 1H), 7.71 (d, *J* = 7.6 Hz, 1H)., 7.54-7.45 (m, 2H), 7.38-7.30 (m, 2H), 7.28-7.26 (m, 1H), 7.13 (t, *J* = 4.8 Hz, 1H), 6.95 (s, 1H). The analytical data are in accordance with those of the previous report.[13]

Eq.2: A 10 mL sealed tube with a magnetic stir bar was charged with **4** (34.1 mg, 0.1 mmol), NaOEt (20.4 mg, 0.3 mmol), MeOH (0.1 mL) and DMSO (0.3 mL). Then the reaction was sealed with Teflon plug and stirred at 60 °C for 10 h. After completion (monitored by TLC), the reaction was quenched by saturated NH_4Cl aqueous solution (15 mL), and then extracted with EtOAc (3 × 15 mL). The combined organic fractions were dried over Na_2SO_4, filtered and concentrated by rotary evaporation. The residue was purified by column chromatography on silica gel (petroleum ether/ethyl acetate = 15/1 to 6/1) to afford **8** in 70% yield (23.9 mg, pale foam).[14]

^{1}H NMR (400 MHz, $CDCl_3$) δ 8.68 (s, 1H), 8.42 (d, *J* = 4.8 Hz, 2H), 7.68 (d, *J* = 7.6 Hz, 1H), 7.49 (d, *J* = 7.6 Hz, 1H), 7.31-7.26 (m, 1H), 7.25-7.18 (m, 2H), 7.16 (t, *J* = 7.6 Hz, 1H), 7.11-6.96 (m, 2H), 6.81 (t, *J* = 4.8 Hz, 1H), 6.67 (d, *J* = 3.2 Hz, 1H), 6.35 (d, *J* = 2.0 Hz, 1H), 3.54 (dt, *J* = 12.4, 3.2 Hz, 1H), 3.16 (ddd, *J* = 17.2, 6.4, 3.2 Hz, 1H), 3.03 (ddd, *J* = 17.2, 10.8, 6.4 Hz, 1H), 2.75 (qd, *J* = 12.4, 6.4 Hz, 1H), 2.22-2.12 (m, 1H). **^{13}C NMR** (100 MHz, $CDCl_3$) δ 165.0, 159.3, 140.1, 137.1, 136.3, 134.8, 130.3, 129.3, 128.8, 128.1, 126.1, 121.2, 120.1, 119.5, 115.2, 110.8, 100.6, 74.9, 39.1, 28.8, 24.1.

IR (thin film): v_{max} (cm^{-1}) = 2961, 2924, 2852, 1572, 1456, 1416, 1309, 1041, 958, 800, 749, 693, 653, 636.

HRMS (ESI) calcd for $C_{22}H_{19}N_3NaO$ $[M+Na]^+$: 364.1426. Found: 364.1420.

HPLC conditions: Chiralcel AD–H column (4.6 mm × 250 mm), hexane/*i*-PrOH, 90:10 v/v, flow rate = 1 mL/min, λ = 254 nm, 25 °C. t_R (minor) = 10.82 min, t_R (major) = 12.80 min, ee > 99%. $[\alpha]_D^{29}$ = -70.64 (*c* = 0.5, $CHCl_3$).

1.4 Mechanistic experiments

(a) Deuterium-labeling experiments

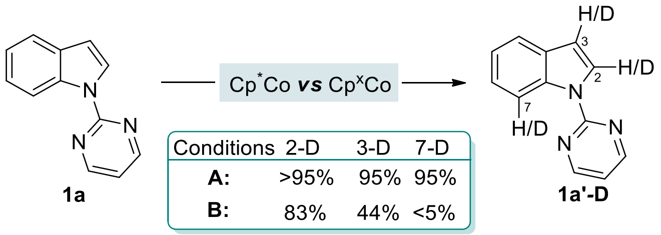

Conditions	2-D	3-D	7-D
**A:**	>95%	95%	95%
**B:**	83%	44%	<5%

Conditions **A:** **Cp*Co(CO)I$_2$**, CsOAc, TFE-D$_3$, 50 °C;
B: **(*R*$_a$)-Co-7**, NaOPiv, TFE-D$_3$/HFIP-D$_2$ (v/v = 3:1), 50 °C.

Conditions **A** (racemic reaction):

A 10 mL sealed tube with a magnetic stir bar was charged with Cp*Co(CO)I$_2$ (1.2 mg, 0.0025 mmol), CsOAc (3.0 mg, 0.015 mmol), **1a** (10.0 mg, 0.05 mmol), TFE-D$_3$ (0.3 mL) under argon atmosphere. The resulting mixture was stirred at 50 °C for 18 h. Then, the mixture was cooled to room temperature and diluted with EtOAc (25 mL), and filtered through a celite pad. The filter cake was washed with EtOAc (30 mL) and the combined filtrate was washed with H$_2$O and brine. The organic layer was dried over Na$_2$SO$_4$, filtered and concentrated. The deuterated ratio was calculated based on ^{1}H NMR analysis of the crude mixture.

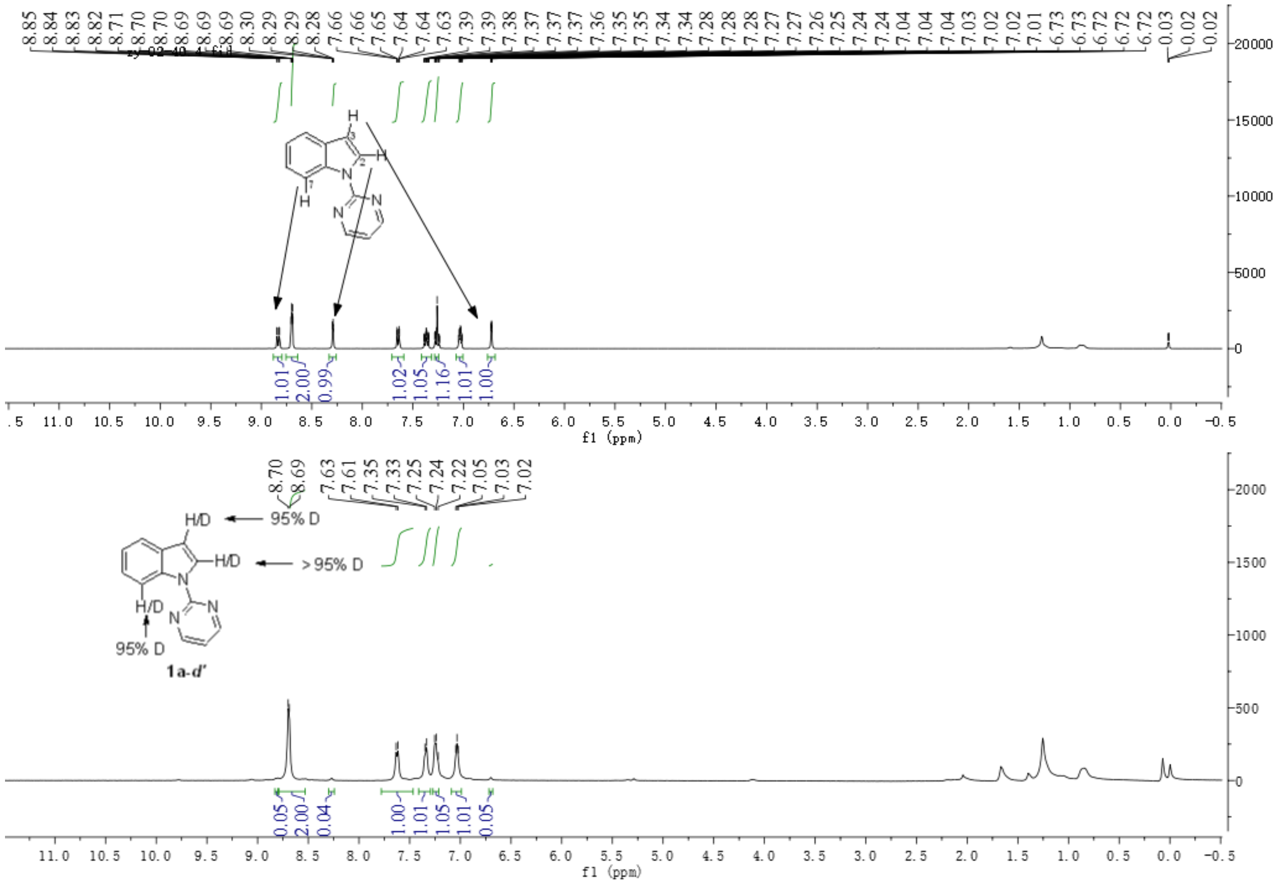

Supplementary Fig. 1 ^{1}H NMR spectrum for deuterium–labeling experiment

Conditions **B** (enantioselective reaction):

To a 10 mL sealed tube with a magnetic stir bar was charged with (*R*a)**-Co-7** (2.4 mg, 0.0025 mmol), NaOPiv·H_2O (2.2 mg, 0.015 mmol), **1a** (10.0 mg, 0.05 mmol), TFE-D_3 (0.3 mL) and HFIP-D_2 (0.1 mL) under argon atmosphere. The resulting mixture was stirred at 50 °C for 18 h. Then, the mixture was cooled to room temperature and diluted with EtOAc (25 mL), and filtered through a celite pad. The filter cake was washed with EtOAc (30 mL) and the combined filtrate was washed with H_2O and brine. The organic layer was dried over Na_2SO_4, filtered and concentrated. The deuterated ratio was calculated based on ^{1}H NMR analysis of the crude mixture.

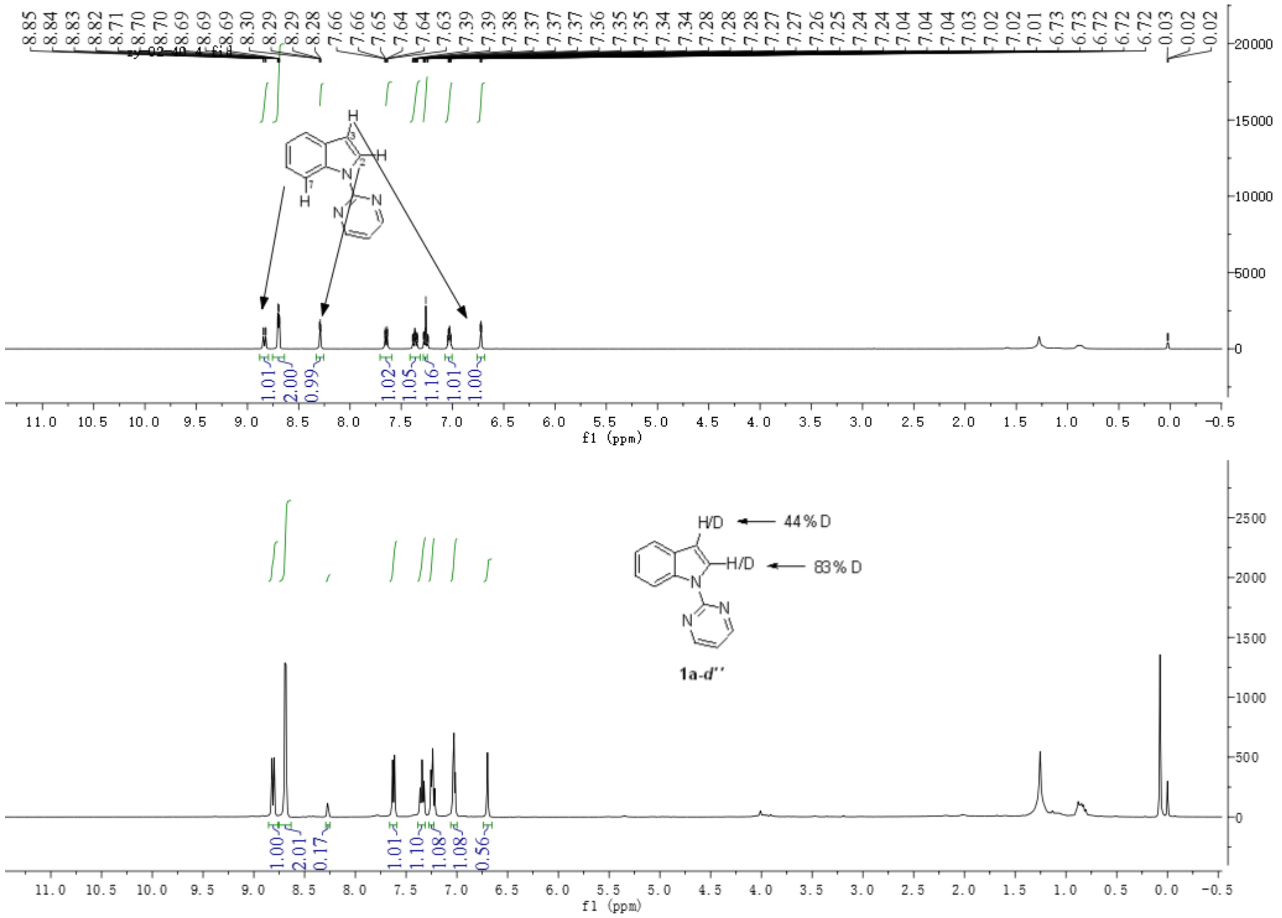

Supplementary Fig. 2 ^{1}H NMR spectrum for deuterium–labeling experiment

(b) Intermolecular kinetic isotope effect of 2-pyrimidyl indole

Racemic reaction:

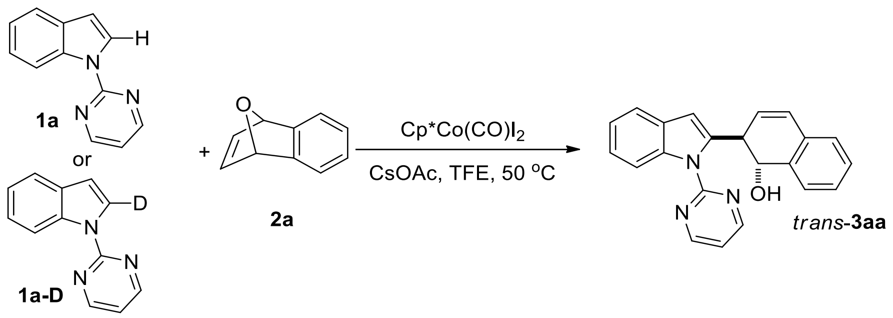

To a 10 mL sealed tube with a magnetic stir bar was charged with Cp*Co(CO)I$_2$ (2.4 mg, 0.005 mmol), CsOAc (5.8 mg, 0.03 mmol), **1a** or **1a-D** (19.5 mg, 0.10 mmol), **2a** (21.6 mg, 0.15 mmol) and TFE (0.5 mL) under argon atmosphere. The resulting mixture was stirred at 50 °C. For each 30 min, 50 μL of the reaction mixture was transferred to a short pad of silica gel and washed with ethyl acetate. The solvent was evaporated, and analyzed by ^{1}H NMR using dibromomethane as an internal standard. The KIE value (k_H/k_D) was calculated to be 1.3 based on the initial reaction rates.

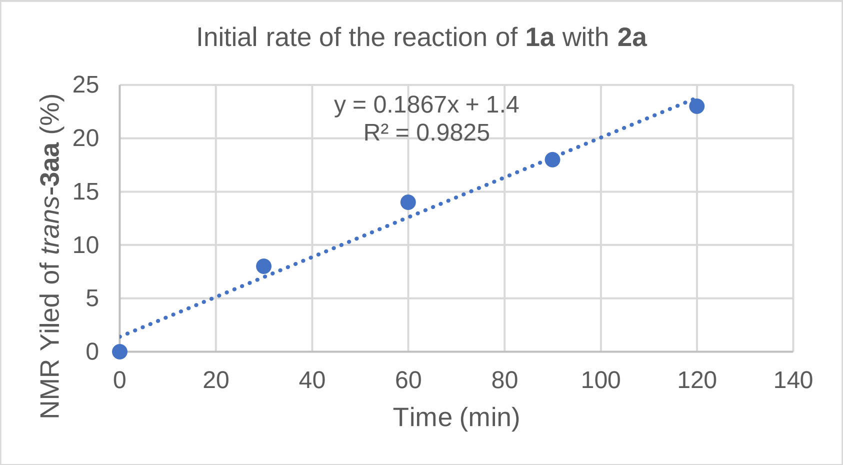

Supplementary Fig. 3 Initial rate of the reaction of **1a** with **2a**

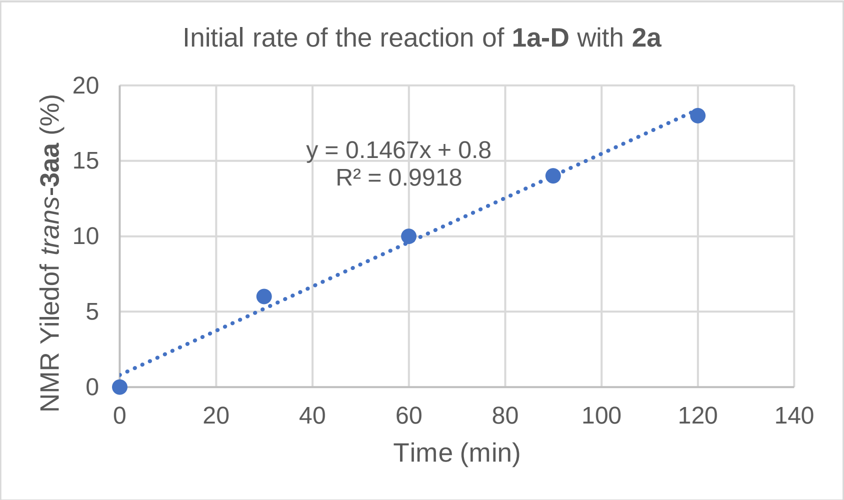

Supplementary Fig. 4 Initial rate of the reaction of **1a-D** with **2a**

Enantioselective reaction:

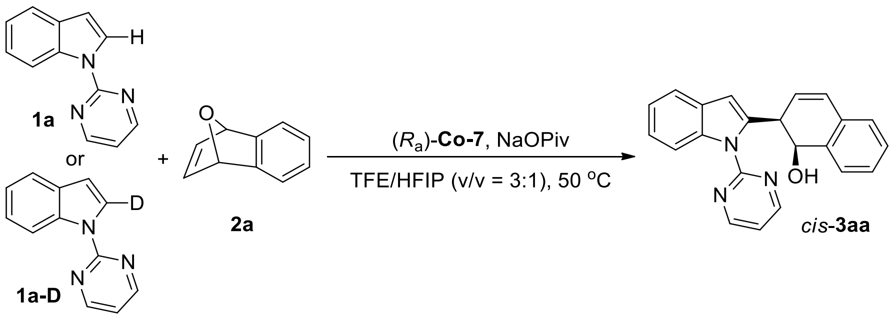

To a 10 mL sealed tube with a magnetic stir bar was charged with (R_a)**-Co-7** (4.0 mg, 0.005 mmol), NaOPiv·H_2O (4.4 mg, 0.03 mmol), **1a** or **1a-D** (19.5 mg, 0.10 mmol), **2a** (21.6 mg, 0.15 mmol), TFE (0.3 mL) and HFIP (0.1 mL) under argon atmosphere. The resulting mixture was stirred at 50 °C. For each 30 min, 50 μL of the reaction mixture was transferred to a short pad of silica gel and washed with ethyl acetate. The solvent was evaporated, and analyzed by ^{1}H NMR using dibromomethane as an internal standard. The KIE value (k_H/k_D) was calculated to be 1.1 based on the initial reaction rates.

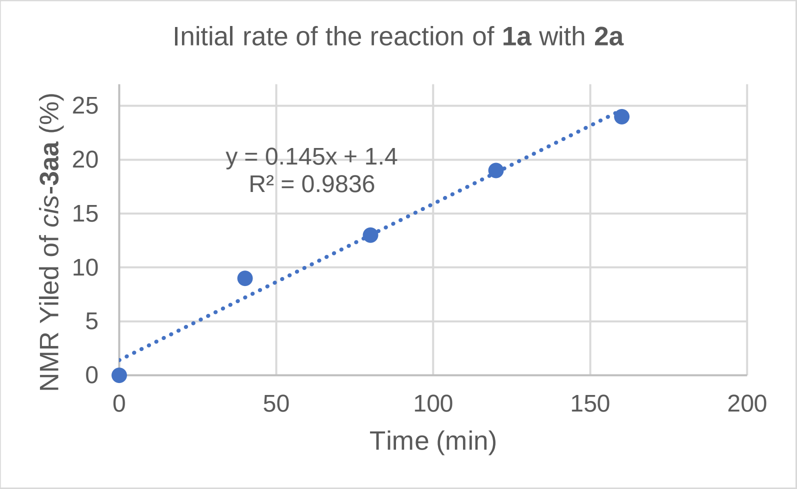

Supplementary Fig. 5 Initial rate of the reaction of **1a** with **2a**

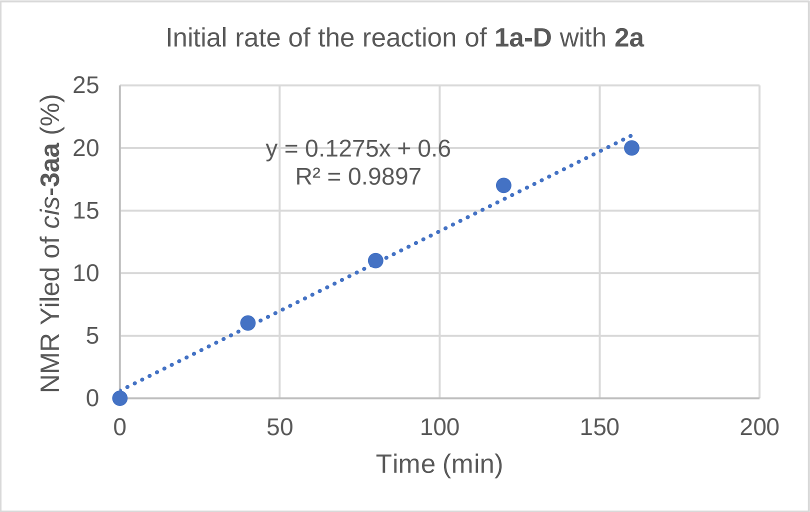

Supplementary Fig. 6 Initial rate of the reaction of **1a-D** with **2a**

(c) ^{13}C KIE Determination

Reference reaction

A sealed tube with a magnetic stir bar was charged with (R_a)**-Co-7** (20.0 mg, 0.025 mmol), NaOPiv·H_2O (44.0 mg, 0.3 mmol), **1a** (195.2 mg, 1.0 mmol), **2** (259.6 mg, 1.5 mmol), TFE (3.0 mL) and HFIP (1.0 mL) under argon atmosphere. The mixture was stirred at 50 °C for 25 h. Then, the mixture was cooled to room temperature and quenched by saturated NH_4Cl aqueous solution (50 mL). The resulting mixture was extracted with ethyl acetate (3 × 25 mL). The combined organic phase was dried over anhydrous Na_2SO_4 and filtered. All the volatiles were evaporated under reduced pressure. The remaining residue was purified by column chromatography on silica gel (hexanes/ethyl acetate = 8:1 to 4:1) to afford *cis*-**3aa** (310.8 mg, 91% yield). A sample of *cis*-**3aa** (150 mg) was subjected to quantitative ^{13}C NMR analysis.

Low conversion reaction procedure

A sealed tube with a magnetic stir bar was charged with (R_a)**-Co-7** (60.1 mg, 0.075 mmol), NaOPiv·H_2O (127.9 mg, 0.9 mmol), **1a** (585.6 mg, 3.0 mmol), **2a** (648.9 mg, 4.5 mmol), TFE (9.0 mL) and HFIP (3.0 mL) under argon atmosphere. The mixture was stirred at 50 °C. Then, the mixture was cooled to room temperature and quenched by saturated NH_4Cl aqueous solution (50 mL). The resulting mixture was extracted with ethyl acetate (3 × 25 mL). The combined organic phase was dried over anhydrous Na_2SO_4 and filtered. All the volatiles were evaporated under reduced pressure. Two parallel experiments were performed at the conversions (of **1a**) of 25% (for 2.3 h) and 12% (for 1.0 h) monitored by ^{1}H NMR. The remaining residues were purified by column chromatography on silica gel (hexanes/ethyl acetate = 8:1 to 4:1) to afford *cis*-**3aa**. The samples of *cis*-**3aa** (120 mg) were subjected to quantitative ^{13}C NMR analysis.

NMR Measurements

Pure samples of *cis*-**3aa** were individually analyzed by ^{13}C NMR spectroscopy, following the protocol of Singleton. The ^{13}C NMR spectra were acquired on a Bruker Avance III 600 MHz spectrometer (150 MHz, $CDCl_3$) at 28 °C with inverse-gated decoupling and calibrated 2π/9 pulses, collecting a total of 512k points. T1 values were determined prior to the acquisitions, and delays of 120 s (120 s > 5 × T1) were utilized between pulses. Five independent acquisitions were obtained for each sample (~19 h acquisition time). Each spectrum was manually integrated three times with a 0th order baseline correction and phase correction.

Results

Carbon-13 KIEs (K_P) were determined from the analysis of the spectra of *cis*-**3aa** via quantitative measurements of ^{13}C peak intensities obtained from peak deconvolution and were calculated using the formula[3] shown below.

$$K_P = \frac{\ln(1-F)}{\ln\left(1 - F\left(\frac{R_P}{R_0}\right)\right)}$$

Where *F* represents the fractional conversion of starting material to product and R represents the carbon peak intensity ratios of the target carbon versus the reference carbon peak (C4 in all cases). R_P is the peak ratio for *cis*-**3aa** at low conversion and R_0 is the peak ratio for the fully converted *cis*-**3aa**.

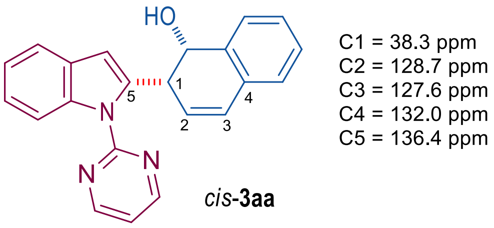

cis-**3aa**

Supplementary Table 1. ^{13}C Kinetic isotope effects sample 1. 25% conversion (*F* = 0.25)

Entry	Carbon	R_0	R_P	R_P/R_0	^{13}C KIE
1	3	104.5221	102.4338	0.9800205	1.023(5)
2	2	103.3009	102.4946	0.9921946	1.009(2)
3	1	100.4979	100.5780	1.0007970	0.999(1)
4	5	102.8170	102.6153	0.9980383	1.002(3)
5	4	100.0000	100.0000	1	1.000

Supplementary Table 2. ^{13}C Kinetic isotope Effects sample 2. 12% conversion (*F* = 0.12)

Entry	Carbon	R_0	R_P	R_P/R_0	^{13}C KIE
1	3	104.5221	101.9446	0.9753401	1.027(0)
2	2	103.3009	101.8309	0.9857697	1.015(4)
3	1	100.4979	100.1539	0.9965770	1.003(7)
4	5	102.8170	102.6600	0.9984730	1.001(7)
5	4	100.0000	100.0000	1	1.000

Supplementary Table 3. ^{13}C Kinetic isotope effects average of the 2 samples.

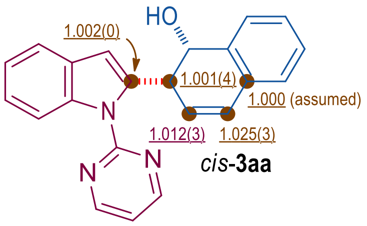

Entry	Carbon	KIE 1	KIE 2	Average
1	3	1.023(5)	1.027(0)	1.025(3)
2	2	1.009(2)	1.015(4)	1.012(3)
3	1	0.999(1)	1.003(7)	1.001(4)
4	5	1.002(3)	1.001(7)	1.002(0)
5	4	1.000	1.000	1.000

1.5 Control experiment

(a) Rh-based catalysts are not effective in the current ARO reactions of 7-oxabenzonorbornadienes

The reactions between _7-oxabenzonorbornadiene_ and indole were conducted under the optimized conditions of Li's work (Conditions 1 and 2. Ref. *Angew. Chem. Int. Ed.* **2019**, *58*, 322-326). *Cis*-**3aa** was not detected, and **1a** and **2a** were recovered. Besides, under our optimized conditions, when the Cp^{x}Co was replaced by Cp^{x}Rh (Conditions 3), the reaction did not occur either. These results demonstrated that the cobalt catalysts exhibit unique reactivity for ARO of 7-oxabenzonorbornadiene.

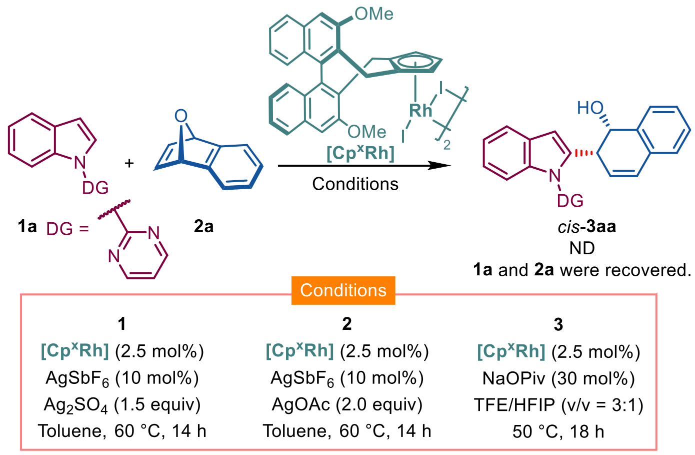

Conditions

1	2	3
[Cp^{x}Rh] (2.5 mol%)	[Cp^{x}Rh] (2.5 mol%)	[Cp^{x}Rh] (2.5 mol%)
$AgSbF_6$ (10 mol%)	$AgSbF_6$ (10 mol%)	NaOPiv (30 mol%)
Ag_2SO_4 (1.5 equiv)	AgOAc (2.0 equiv)	TFE/HFIP (v/v = 3:1)
Toluene, 60 °C, 14 h	Toluene, 60 °C, 14 h	50 °C, 18 h

Conditions 1: A 10 mL sealed tube with a magnetic stir bar was charged with **1a** (78.1 mg, 0.40 mmol), **2a** (28.8 mg, 0.20 mmol), [Cp^{x}Rh] (7.6 mg, 0.005 mmol), $AgSbF_6$ (6.9 mg, 0.020 mmol), Ag_2SO_4 (93.5 mg, 0.30 mmol), toluene (4.0 mL) under argon atmosphere. The resulting mixture was stirred at 60 °C for 14 h. Then, the mixture was cooled to room temperature and diluted with EtOAc (25 mL), and filtered through a celite pad. The filter cake was washed with EtOAc (30 mL) and the combined filtrate was washed with H_2O and brine. The organic layer was dried over Na_2SO_4, filtered and concentrated. Then the crude mixture was subjected to ^{1}H NMR analysis. **1a** and **2a** were recovered by column chromatography on silica gel (petroleum ether/ethyl acetate = 50/1 to 20/1).

Conditions 2: A 10 mL sealed tube with a magnetic stir bar was charged with **1a** (58.6 mg, 0.30 mmol), **2a** (28.8 mg, 0.20 mmol), [Cp^{x}Rh] (7.6 mg, 0.005 mmol), $AgSbF_6$ (6.9 mg, 0.020 mmol), AgOAc (66.8 mg, 0.40 mmol), toluene (4.0 mL) under argon atmosphere. The resulting mixture was stirred at 60 °C for 14 h. Then, the mixture was cooled to room temperature and diluted with EtOAc (25 mL), and filtered through a celite pad. The filter cake was washed with EtOAc (30 mL) and the combined filtrate was washed with H_2O and brine. The organic layer was dried over Na_2SO_4, filtered and concentrated. TLC indicated that no reaction occurred.

Conditions 3: A sealed tube with a magnetic stir bar was charged with **1a** (19.5 mg, 0.1 mmol), **2a** (21.6 mg, 0.15 mmol), [Cp^{x}Rh] (3.8 mg, 0.0025 mmol), NaOPiv·H_2O (4.4 mg, 0.030 mmol),TFE (0.3 mL) and HFIP (0.1 mL) under argon atmosphere. The mixture was stirred at 50 °C for 18 h. Afterwards, the mixture was cooled to room temperature and quenched by saturated NH_4Cl aqueous solution (20 mL). The resulting mixture was extracted with ethyl acetate (3 × 15 mL). The combined organic phase was dried over anhydrous Na_2SO_4 and filtered. TLC indicated that no reaction occurred.

(b) No post-coupling interconversion exits between the *cis*- and *trans*-products

The model reactions yielding *cis*-**3aa** and *trans*-**3aa** were conducted respectively (Supplementary Scheme 1a). Then the *cis*-**3aa** and *trans*-**3aa** were isolated in high yields with good diastereoselectivity (*cis*-**3aa**, 98% yield, 95:5 dr; *trans*-**3aa**, 95% yield, 90:10 dr). After that, *cis*-**3aa** or *trans*-**3aa** was tested under the conditions of *trans* (conditions **A**) or *cis* (conditions **B**), respectively. In both cases, **3aa** was mainly recovered with no change of their dr ratios (Supplementary Scheme 1b and Figure S7).

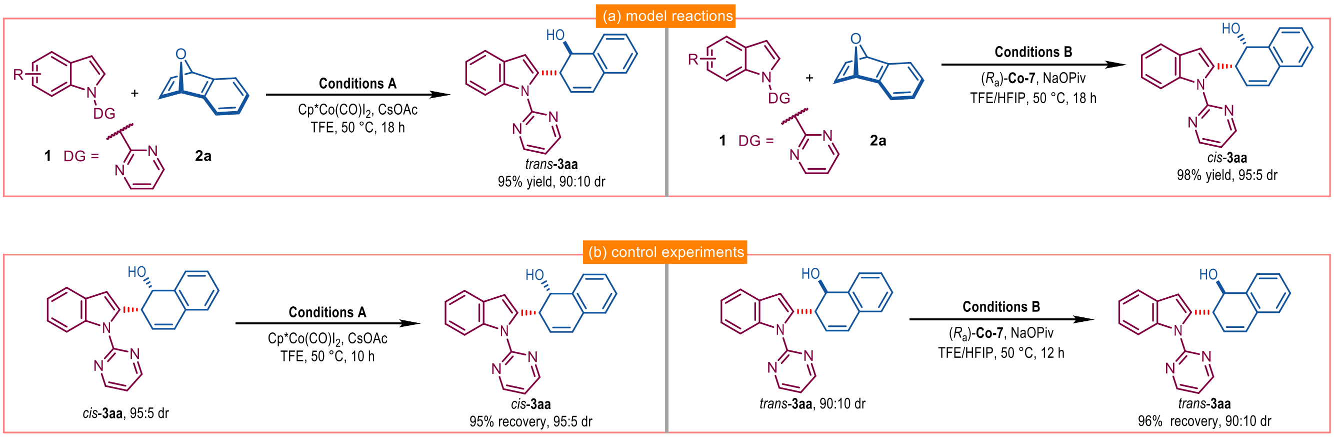

Supplementary Scheme 1. Control experiments, shown that no post-coupling interconversion exits between the *cis*- and *trans*-products. Conditions A: Cp*Co(Co)I$_2$ (0.005 mmol), CsOAc (0.03 mmol) in TFE (0.5 mL) at 50 °C for 10 h. Conditions B: (R_a)-**Co-7** (0.005 mmol), NaOPiv·H_2O (0.03 mmol) in TFE (0.3 mL) and HFIP (0.1 mL) at 50 °C for 12 h.

Investigation of *cis*-**3aa** under Conditions A (Supplementary Scheme 1b, left): A sealed tube with a magnetic stir bar was charged with *cis*-**3aa** (34.0 mg, 0.10 mmol), Cp*Co(Co)I$_2$ (2.4 mg, 0.005 mmol), CsOAc (5.8 mg, 0.03 mmol), TFE (0.5 mL) under argon atmosphere. The mixture was stirred at 50 °C for 10 h. Afterwards, the mixture was cooled to room temperature and quenched by saturated NH_4Cl aqueous solution (20 mL). The resulting mixture was extracted with ethyl acetate (3 × 15 mL). The combined organic phase was dried over anhydrous Na_2SO_4 and filtered. Then the crude mixture was subjected to ^{1}H NMR analysis. And *cis*-**3aa** was recovered by column chromatography on silica gel (petroleum ether/ethyl acetate = 4/1).

Investigation of *trans*-**3aa** under Conditions B (Supplementary Scheme 1b, right): A sealed tube with a magnetic stir bar was charged with *trans*-**3aa** (34.1 mg, 0.10 mmol), (R_a)-**Co-7** (4.0 mg, 0.005 mmol), NaOPiv·H_2O (4.4 mg, 0.03 mmol), TFE (0.3 mL) and HFIP (0.1 mL) under argon atmosphere. The mixture was stirred at 50 °C for 12 h. Afterwards, the mixture was cooled to room temperature and quenched by saturated NH_4Cl aqueous solution (20 mL). The resulting mixture was extracted with ethyl acetate (3 × 15 mL). The combined organic phase was dried over anhydrous Na_2SO_4 and filtered. Then the crude mixture was subjected to ^{1}H NMR analysis. And *trans*-**3aa** was recovered by column chromatography on silica gel (petroleum ether/ethyl acetate = 4/1).

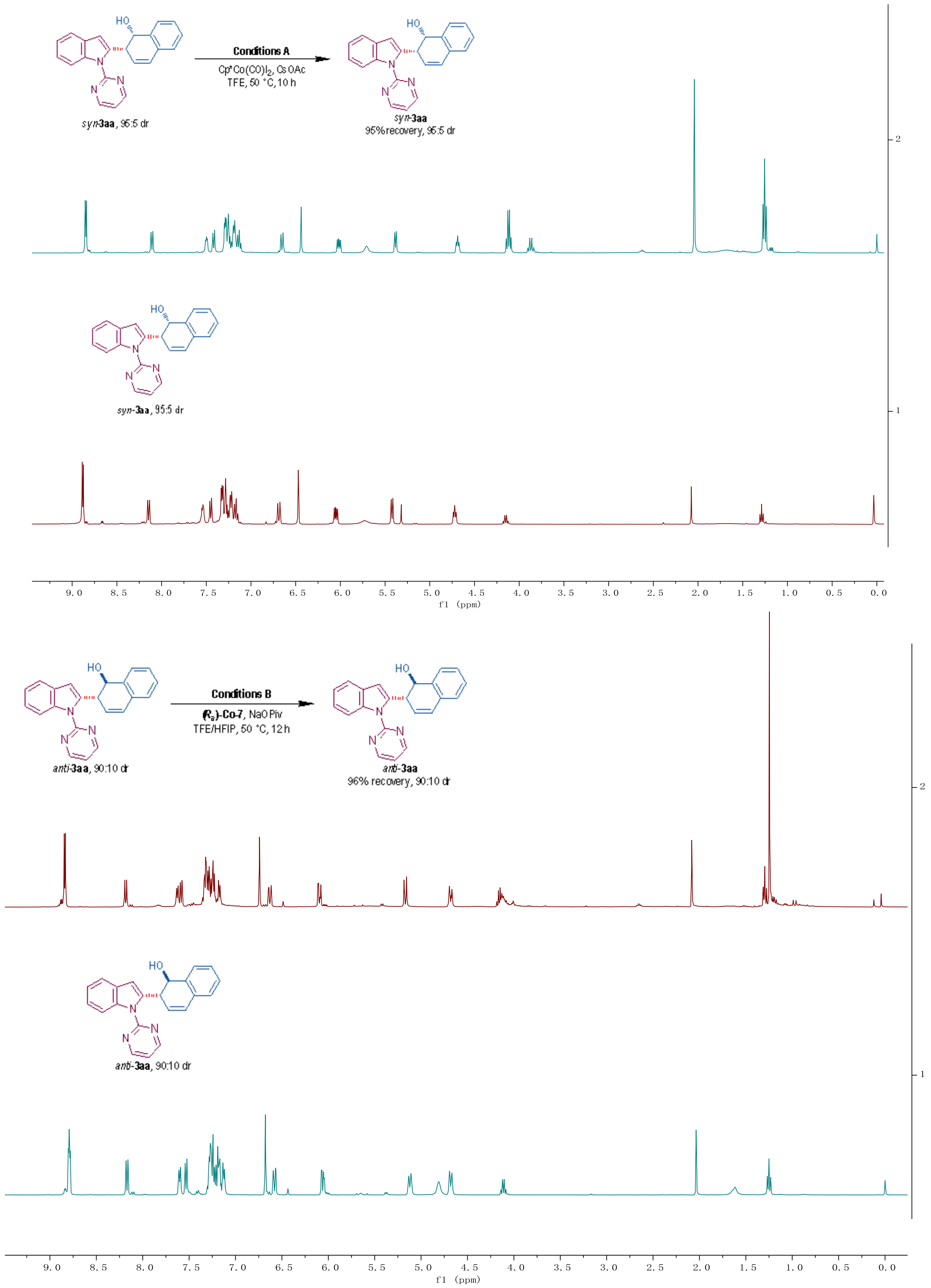

Supplementary Fig. 7 ^{1}H NMR spectra of the crude mixtures (control experiments). Conditions A: Cp*Co(Co)I_2 (0.005 mmol), CsOAc (0.03 mmol) in TFE (0.5 mL) at 50 °C for 10 h. Conditions B: (*R*a)-**Co-7** (0.005 mmol), NaOPiv·H_2O (0.03 mmol) in TFE (0.3 mL) and HFIP (0.1 mL) at 50 °C for 12 h.

1.6 X-Ray crystal structures

(a) X-ray crystal structure of *trans*-**3da**

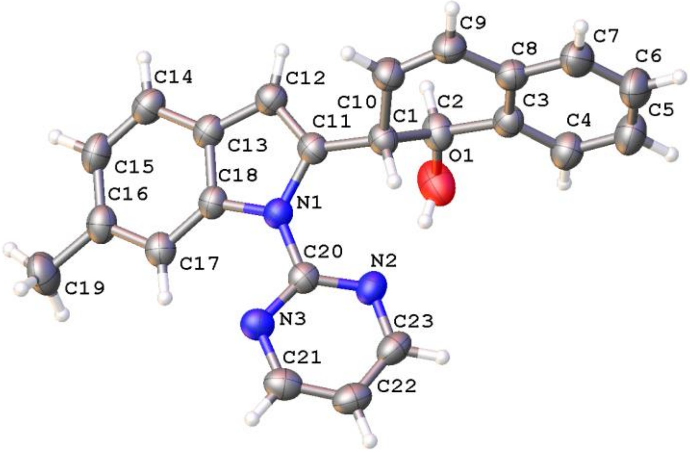

Supplementary Fig. 8 The X-ray crystal structure of enantiopure *trans*-**3da** with thermal ellipsoids at the 30% probability level (CCDC: 2164324).

Supplementary Table 4. Crystal data and structure refinement for mj21175_0m.

Identification code	mj21175_0m	
Empirical formula	C23 H19 N3 O	
Formula weight	353.41	
Temperature	173.01 K	
Wavelength	1.34139 Å	
Crystal system	Monoclinic	
Space group	C 1 2/c 1	
Unit cell dimensions	a = 26.557(3) Å	α = 90°.
	b = 7.0049(9) Å	β = 107.253(7)°.
	c = 20.726(3) Å	γ = 90°.
Volume	3682.0(8) Å^3	
Z	8	
Density (calculated)	1.275 Mg/m^3	
Absorption coefficient	0.405 mm^{-1}	
F(000)	1488	
Crystal size	0.05 x 0.03 x 0.01 mm^3	
Theta range for data collection	3.886 to 55.119°.	
Index ranges	-32<=h<=32, -8<=k<=7, -25<=l<=20	
Reflections collected	15700	
Independent reflections	3519 [R(int) = 0.0893]	
Completeness to theta = 53.594°	99.9 %	
Absorption correction	Semi-empirical from equivalents	
Max. and min. transmission	0.7508 and 0.3677	
Refinement method	Full-matrix least-squares on F^2	

Data / restraints / parameters	3519 / 0 / 246
Goodness-of-fit on F^2	1.066
Final R indices [I>2sigma(I)]	R1 = 0.0841, wR2 = 0.2277
R indices (all data)	R1 = 0.1280, wR2 = 0.2665
Extinction coefficient	n/a
Largest diff. peak and hole	0.505 and -0.278 e.Å^{-3}

(b) X-ray crystal structure of enantiopure *cis*-**3da**

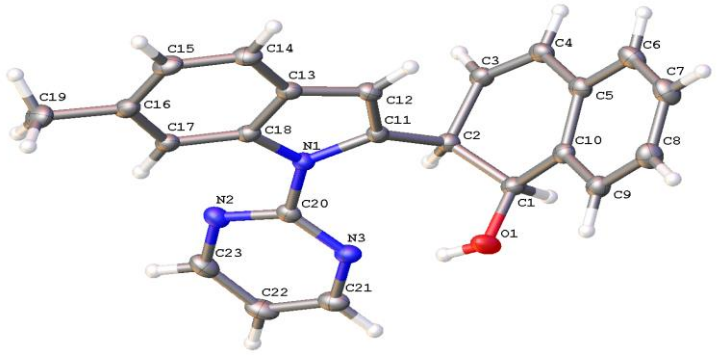

Supplementary Fig. 9 The X-ray crystal structure of enantiopure *cis*-**3da** with thermal ellipsoids at the 30% probability level (CCDC: 2164323).

Supplementary Table 5. Crystal data and structure refinement for mj21150_0m.

Identification code	mj21150_0m	
Empirical formula	C23 H19 N3 O	
Formula weight	353.41	
Temperature	173.01 K	
Wavelength	1.34139 Å	
Crystal system	Orthorhombic	
Space group	$P2_12_12_1$	
Unit cell dimensions	a = 7.0927(2) Å	α = 90°.
	b = 13.5847(3) Å	β = 90°.
	c = 18.5402(5) Å	γ = 90°.
Volume	1786.39(8) Å^3	
Z	4	
Density (calculated)	1.314 Mg/m^3	
Absorption coefficient	0.418 mm^{-1}	
F(000)	744	
Crystal size	0.1 x 0.08 x 0.05 mm^3	
Theta range for data collection	5.025 to 55.035°.	
Index ranges	-8<=h<=7, -16<=k<=16, -22<=l<=22	
Reflections collected	20323	
Independent reflections	3404 [R(int) = 0.0540]	
Completeness to theta = 53.594°	99.4 %	
Absorption correction	Semi-empirical from equivalents	
Max. and min. transmission	0.7508 and 0.5671	
Refinement method	Full-matrix least-squares on F^2	
Data / restraints / parameters	3404 / 0 / 246	
Goodness-of-fit on F^2	1.052	
Final R indices [I>2sigma(I)]	R1 = 0.0304, wR2 = 0.0760	

R indices (all data)	R1 = 0.0315, wR2 = 0.0769
Absolute structure parameter	0.06(12)
Extinction coefficient	n/a
Largest diff. peak and hole	0.122 and -0.152 e.Å^{-3}

(c) X-ray crystal structure of enantiopure *trans*-**7ab’**

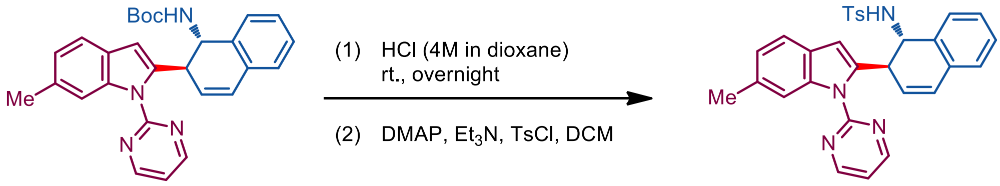

trans-**7ab**, > 99% ee

trans-**7ab'** (CCDC: 2208797)
88% yield over two steps
> 99% ee

<u>Conditions 1:</u> A 4 mL vial with a magnetic stir bar was charged with *trans*-**7ab** (49.2 mg, 95:5 dr, >99% ee, 0.11 mmol) and HCl (2.0 mL, 4 mol/L in dioxane). The resulting mixture was stirred at room temperature for overnight. The reaction mixture was quenched by saturated Na_2CO_3 aqueous solution (30 mL). The resulting mixture was extracted with DCM (3 × 15 mL). The combined organic phase was dried over anhydrous Na_2SO_4, filtered and concentrated, giving the amine as white solid used in the next step without further purification.
<u>Conditions 2:</u> A 10 mL round-bottomed flask with a magnetic stir bar was charged with aforementioned amine (35.2 mg), *p*-toluenesulfonyl chloride (TsCl, 22.9 mg, 0.12 mmol), Et_3N (83 μL, 0.60 mmol), DMAP (12.0 mg, 0.10 mmol) and DCM (2.0 mL). The resulting mixture was stirred at room temperature for 12 h. After the reaction was complete, the solvent was removed by a rotary evaporator. The residue was purified by column chromatography on silica gel (petroleum ether/ethyl acetate = 8/1 to 4/1) to afford *trans*-**7ab’** (45.6 mg, 88% yield over two steps).

trans-**7ab’**, white solid. **^{1}H NMR** (400 MHz, $CDCl_3$) δ 8.89 (d, *J* = 4.8 Hz, 2H), 8.06 (d, *J* = 6.4 Hz, 1H), 7.83 (s, 1H), 7.72 (d, *J* = 7.2 Hz, 1H), 7.28 (t, *J* = 5.2 Hz, 2H), 7.26-7.23 (m, 1H), 7.19 (d, *J* = 8.4 Hz, 2H), 7.13 (d, *J* = 8.0 Hz, 1H), 7.10-7.04 (m, 1H), 6.99 (d, *J* = 8.0 Hz, 1H), 6.57 (d, *J* = 8.0 Hz, 2H), 6.50 (dd, *J* = 9.6, 2.4 Hz, 1H), 6.16 (s, 1H), 5.88 (dd, *J* = 9.6, 2.8 Hz, 1H), 4.78 (dd, *J* = 12.0, 6.4 Hz, 1H), 4.49 (dt, *J* = 12.0, 2.8 Hz, 1H), 2.50 (s, 3H), 2.12 (s, 3H). **^{13}C NMR** (100 MHz, $CDCl_3$) δ 158.8, 157.1, 142.4, 139.6, 137.2, 136.7, 135.7, 133.1, 132.9, 131.2, 129.0, 128.3, 128.0, 127.97, 127.3, 127.1, 126.3, 126.1, 123.8, 120.1, 117.9, 113.4, 107.4, 59.7, 39.1, 22.2, 21.7.
IR (thin film): ν_{max} (cm^{-1}) = 2955, 2919, 2850, 1732, 1645, 1564, 1486, 1425, 1379, 1332, 1261, 1185, 1155, 1118, 1092, 1021, 955, 915, 872, 809, 758, 725, 697, 663.
HRMS (ESI) calcd for $C_{30}H_{26}O_2N_4SNa$ $[M+Na]^+$: 529.1674. Found: 529.1663.
HPLC conditions: Chiralcel AD–H column (4.6 mm × 250 mm), hexane/*i*-PrOH, 70:30 v/v, flow rate = 1.0 mL/min, λ = 254 nm, 25 °C. t_R (major) = 10.35 min, t_R (minor) = 14.45 min, ee > 99%. $[\alpha]_D^{33}$ = -97.1 (*c* = 0.1, $CHCl_3$).

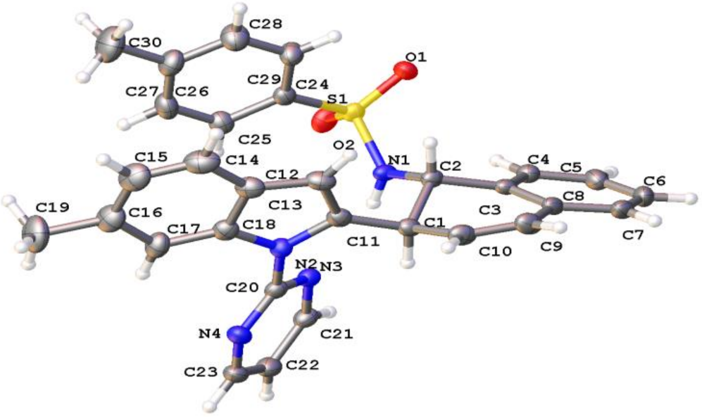

Supplementary Fig. 10 The X-ray crystal structure of enantiopure *trans*-**7ab’** with thermal ellipsoids at the 30% probability level (CCDC: 2208797).

Supplementary Table 6. Crystal data and structure refinement for mj22376_0m.

Identification code	mj22376_0m
Empirical formula	C30 H26 N4 O2 S

Formula weight	506.61	
Temperature	213.00 K	
Wavelength	1.34139 Å	
Crystal system	Monoclinic	
Space group	P 1 21 1	
Unit cell dimensions	a = 10.7690(3) Å	a= 90°.
	b = 12.6152(3) Å	b= 118.946(2)°.
	c = 11.0758(3) Å	g = 90°.
Volume	1316.71(6) Å^3	
Z	2	
Density (calculated)	1.278 Mg/m^3	
Absorption coefficient	0.888 mm^{-1}	
F(000)	532	
Crystal size	0.07 x 0.07 x 0.05 mm^3	
Theta range for data collection	4.090 to 55.015°.	
Index ranges	-12<=h<=13, -15<=k<=15, -13<=l<=13	
Reflections collected	19336	
Independent reflections	4984 [R(int) = 0.0595]	
Completeness to theta = 53.594°	99.7 %	
Absorption correction	Semi-empirical from equivalents	
Max. and min. transmission	0.7508 and 0.5495	
Refinement method	Full-matrix least-squares on F^2	
Data / restraints / parameters	4984 / 1 / 336	
Goodness-of-fit on F^2	1.042	
Final R indices [I>2sigma(I)]	R1 = 0.0506, wR2 = 0.1222	
R indices (all data)	R1 = 0.0756, wR2 = 0.1360	
Absolute structure parameter	0.044(13)	
Extinction coefficient	n/a	
Largest diff. peak and hole	0.639 and -0.376 e.Å^{-3}	

1.7 Absolute configuration of *cis*-7

(a) Reported results (Ref. [12], *Angew. Chem. Int. Ed.* 2019, *58*, 322-326).

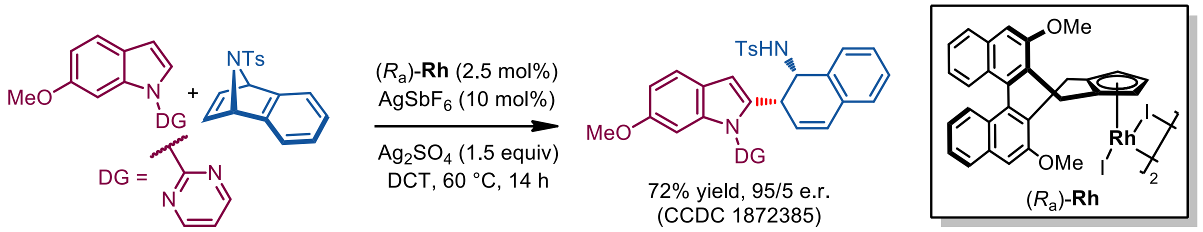

In Ref. [12], HPLC conditions: Chiralcel AD–H, hexane/*i*-PrOH, 70:30 v/v, flow rate = 1.0 mL/min, λ = 254 nm, 40 °C. t_R (minor) = 10.43 min, t_R (major) = 12.73 min, ee = 90%. $[\alpha]_D^{20}$ = -98.07 (*c* = 0.2, CH_2Cl_2).

(b) Conditions in this work for the synthesis of *cis*-7gb.

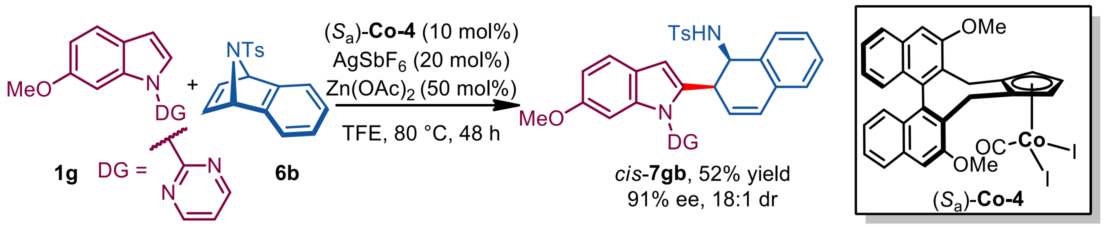

cis-**7gb**, 27.2 mg, 52% yield, pale yellow foam. **^{1}H NMR** (400 MHz, $CDCl_3$) 8.74 (d, *J* = 4.8 Hz, 2H), 7.88 (d, *J* = 2.4 Hz, 1H), 7.43 (d, *J* = 7.2 Hz, 1H), 7.32-7.21 (m, 5H), 7.19-7.10 (m, 2H), 6.85 (dd, *J* = 8.4, 2.4 Hz, 1H), 6.67 (d, *J* = 8.0 Hz, 2H), 6.61 (dd, *J* = 9.6, 2.4 Hz, 1H), 6.35 (s, 1H), 6.11 (dd, *J* = 9.6, 3.6 Hz, 1H), 5.41 (d, *J* = 8.8 Hz, 1H), 5.11 (dd, *J* = 8.8, 6.0 Hz, 1H), 4.82 (dt, *J* = 5.6, 2.8 Hz, 1H), 3.89 (s, 3H), 2.14 (s, 3H). The analytical data is in accordance with the previous report.[12]
HPLC conditions: Chiralcel AD–H column (4.6 mm × 250 mm), hexane/*i*-PrOH, 70:30 v/v, flow rate = 1.0 mL/min, λ = 254 nm, 25 °C. t_R (major) = 12.31 min, t_R (minor) = 16.44 min, ee = 91%. $[\alpha]_D^{26}$ = +97.5 (*c* = 0.2, CH_2Cl_2).

1.8 Computational calculations

Computational methods.

All the computational works were performed with Gaussian16[4] or ORCA 4.1.0 packages.[5] DFT calculations were carried out using the B3LYP-D3(BJ) functional[6] including the D3 version of Grimme's empirical dispersion correction with Becke−Johnson damping.[7] The def2-SVP basis sets[8] were applied for all atoms. Optimizations were conducted without any constraint in gas phase. Frequency analyses were carried out to confirm each structure being a minimum (no imaginary frequency) or a transition state (only one imaginary frequency). Single-point calculations with RI-PWPB95-D3(BJ) method and def2-TZVPP basis sets (with auxiliary basis sets def2-TZVPP/C[9] and def2/JK[10]) were performed using the geometries obtained at the B3LYP-D3(BJ)/def2-SVP level of theory. The calculated structures were visualized with VMD 1.9.3.[11]

Reaction pathway involving chiral CpCo catalyst. The reaction of N-pyrimidinylindole (**1a**) and 7-oxabenzonorbornadiene (**2a**) catalyzed by the chiral CpCo complex with pivalate anion was considered (Figure S12). The cationic Co-complex with **1a** was set as zero-point of the potential energy surface (**INT-0**, 0.0 kcal/mol). In this complex, **1a** was coordinated to the Co center with the nitrogen atom of the pyrimidine moiety. The indole C–H cleavage proceeded via a well-recognized six-membered-ring concerted metallation deprotonation (CMD) transition state **TS-1** (8.8 kcal/mol). The Co–C bond formation [B(Co−C) = 2.03 Å] and the concomitant proton transfer to the ligated pivalate anion [B(C1–H) = 1.24 Å and B(O2–H) = 1.41 Å] gave rise to the cyclometallated intermediate **INT-1** (-3.9 kcal/mol). Then, the pivalic acid was replaced by **2a**, leading to intermediate **INT-2** (0.5 kcal/mol) in which a hydrogen bond was formed between the pivalic acid and the oxygen atom of **2a**. Subsequently, the migratory insertion of the Co–C bond into the olefin moiety of **2a** proceeded via transition state **TS-2** (6.4 kcal/mol), which yielded intermediate **INT-3** (2.6 kcal/mol). The following β-oxygen elimination was the enantioselectivity and diastereoselectivity-determining step of the whole reaction. Among the transition states leading to all possible isomers (Figure S12), the one corresponding to the generation of (1*S*,2*R*)-*cis*-**3aa** was energetically the most favorable [**TS-*cis-SR***, 13.7 kcal/mol, named as **TS-*cis-SR*** in the main text], which well reproduced the predominate formation of this isomer experimentally. Notably, its energy was much higher than those of **TS-1** and **TS-2**, thus making the previous C−H cleavage and olefin insertion steps reversible, which was consistent with the deuterium-labelling experiment. Meanwhile, this C=C and Co–O bond-formation process was highly exergonic, leading to **INT-4** with rather low energy (-22.1 kcal/mol) and the irreversibly established of the chirality of product. On the contrary, the transition state leading to the opposite enantiomer (1*R*,2*S*)-*cis*-**3aa** was also located [**TS-3-*cis-RS***, 21.8 kcal/mol, named as **TS-*cis-RS*** in the main text], whose energy was much higher than that of **TS-3-*cis-SR*** by 8.1 kcal/mol. Stronger steric repulsion was observed for **TS-3-*cis-RS***, which was exemplified by short H–H distances (less than 2.20 Å) between 7-oxabenzonorbornadiene and the tert-butyl group of the catalyst. On the other hand, the energies of the transition states for the formation of the other diastereoisomers of the products (*trans*-**3aa**) are even higher [**TS-3-*trans-RR***, 21.5 kcal/mol and **TS-3-*trans-SS***, 22.3 kcal/mol]. Higher degree of charge separation was observed in these two transition states compared with those in **TS-*cis-SR*** and **TS-3-*cis-RS*** because the Co–O interaction was absence in **TS-3-*trans-RR*** and **TS-3-*trans-SS***.

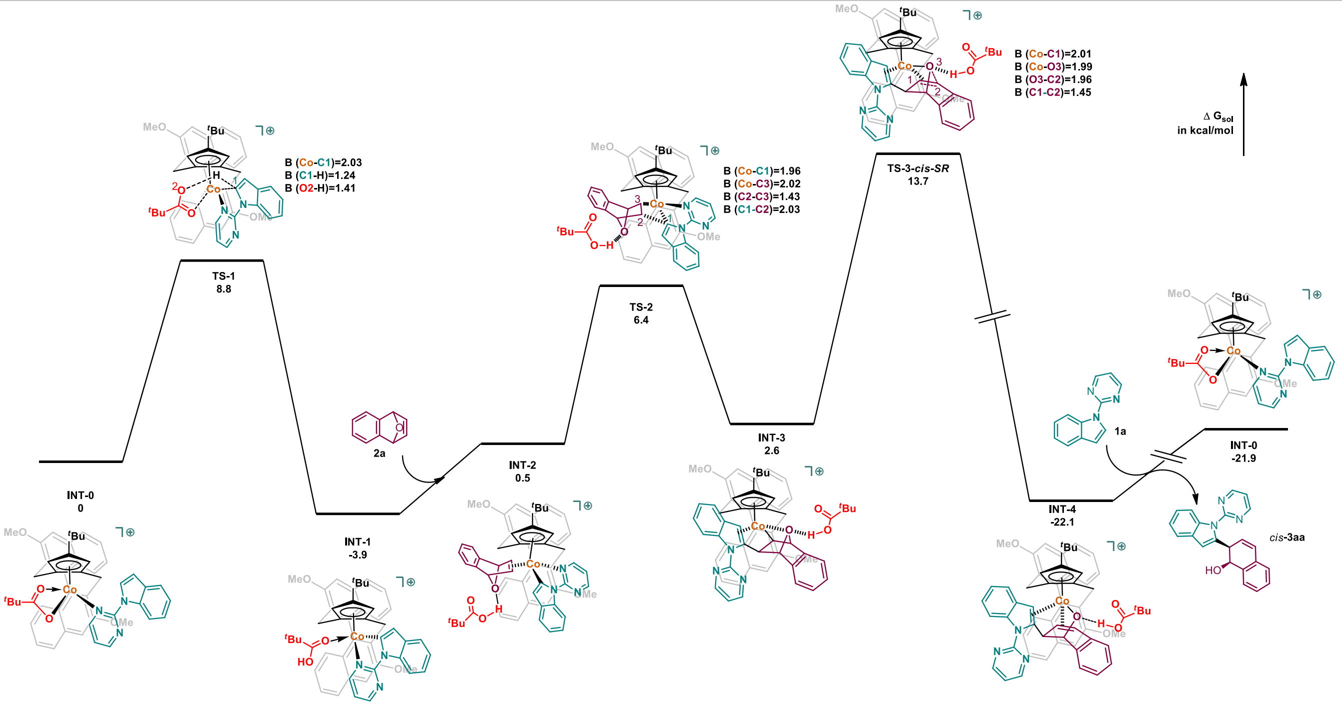

Supplementary Fig. 11 The profile of the alternative reaction pathway involving chiral CpCo catalyst calculated at the PWPB95-D3(BJ)/def2-TZVPP (SMD, TFE)//B3LYP-D3(BJ)/def2-SVP (gas) level of theory. The relative Gibbs free energies (ΔG_{sol}) are in kcal/mol. The distances of forming/cleaving bonds are in Å.

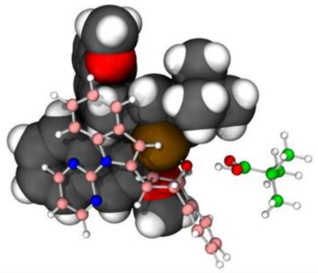

TS-3-*cis*-SR
13.7 kcal/mol

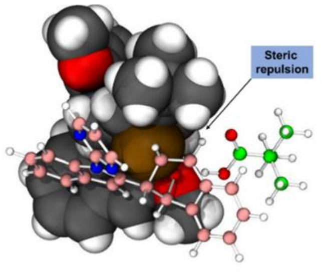

TS-3-*cis*-RS
21.8 kcal/mol

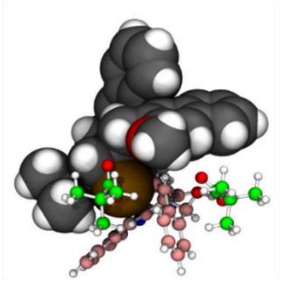

TS-3-*trans*-RR
21.5 kcal/mol

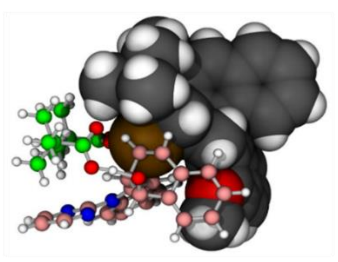

TS-3-*trans*-SS
22.3 kcal/mol

Supplementary Fig. 12 Optimized structures of key β-oxygen elimination transition states. Calculated at the PWPB95-D3(BJ)/def2-TZVPP (SMD, TFE)//B3LYP-D3(BJ)/def2-SVP (gas) level of theory. The cyclopentadienyl ligands are presented in the van der Waals model. The cobalt center and the substrates are shown in the ball-and-stick model. The relative Gibbs free energies (ΔG_{sol}) are represented in kcal/mol. The bond distances are presented in Å.

Reaction pathway involving Cp*Co(CO)I$_2$ as the catalyst. When Cp*Co(CO)I$_2$ was employed as the catalyst, racemic *trans*-**3aa** became the major product (10:90 *trans*/*cis*, entry1 in Table 1 in the main text). The energy profile for the reaction between **1a** and **2a** catalyzed by the Cp*Co catalyst was considered (Figure S13). Starting from complex **INT-0′**(0.0 kcal/mol), the C–H activation of **1a** proceeded via transition state **TS-1′** (12.8 kcal/mol) in which

an acetate anion worked as the internal base [B(C1–H) = 1.24 Å and B(O1–H) = 1.39 Å], leading to cyclometallated complex **INT-1′** (2.6 kcal/mol). The formed acetic acid was next replaced by **2a** via *endo*- or *exo*-coordination to yield intermediates ***INT-2′-trans*** (10.5 kcal/mol) or ***INT-2′-cis*** (-1.4 kcal/mol). In both cases, a hydrogen bond was formed between the acetic acid and **2a**. Migratory insertion of C–Co bond into the olefin moiety of **2a** proceeded via transition states ***TS-2′-trans*** (19.3 kcal/mol) or ***TS-2′-cis*** (8.8 kcal/mol) in which the Co center was located in the opposite or the same side of the oxygen bridge in **2a**. It should be mentioned that the Cp* ligand is sterically less hindered compared with the chiral Cp ligand used in the enantioselective reaction. Thus, an additional acetate anion could coordinate to the Co center in the following *anti*-β-oxygen elimination transition state [***TS-3′-trans***, 19.4 kcal/mol, named as ***TS-trans-rac*** in the main text] from intermediate ***INT-4′-trans*** (2.2 kcal/mol). This stabilizing effect made ***TS-3′-trans*** energetically more favorable than the transition state of *cis*-β-oxygen elimination [***TS-3′-cis***, 22.6 kcal/mol, named as ***TS-cis-rac*** in the main text]. Finally, *trans*-**3aa** was released from intermediate ***INT-5′-trans*** (-5.5 kcal/mol).

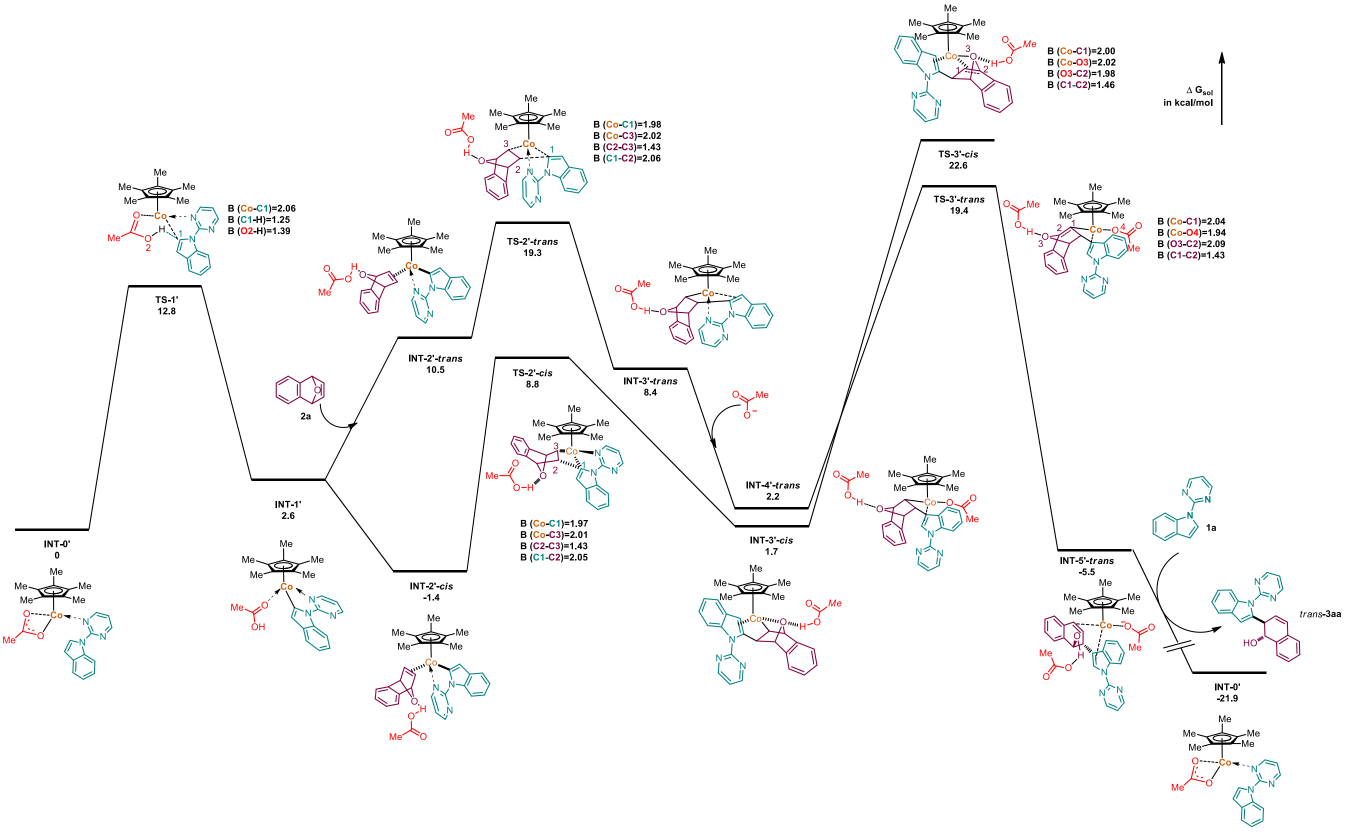

Supplementary Fig. 13 The profile of the alternative reaction pathway involving Cp*Co(CO)I$_2$ catalyst calculated at the PWPB95-D3(BJ)/def2-TZVPP (SMD, TFE)//B3LYP-D3(BJ)/def2-SVP (gas) level of theory. The relative Gibbs free energies (ΔG_{sol}) are in kcal/mol. The distances of forming/cleaving bonds are in Å.

1.9 Copies of NMR spectra

Supplementary Fig. 14 ^{1}H NMR Spectrum of *trans*-**3aa**

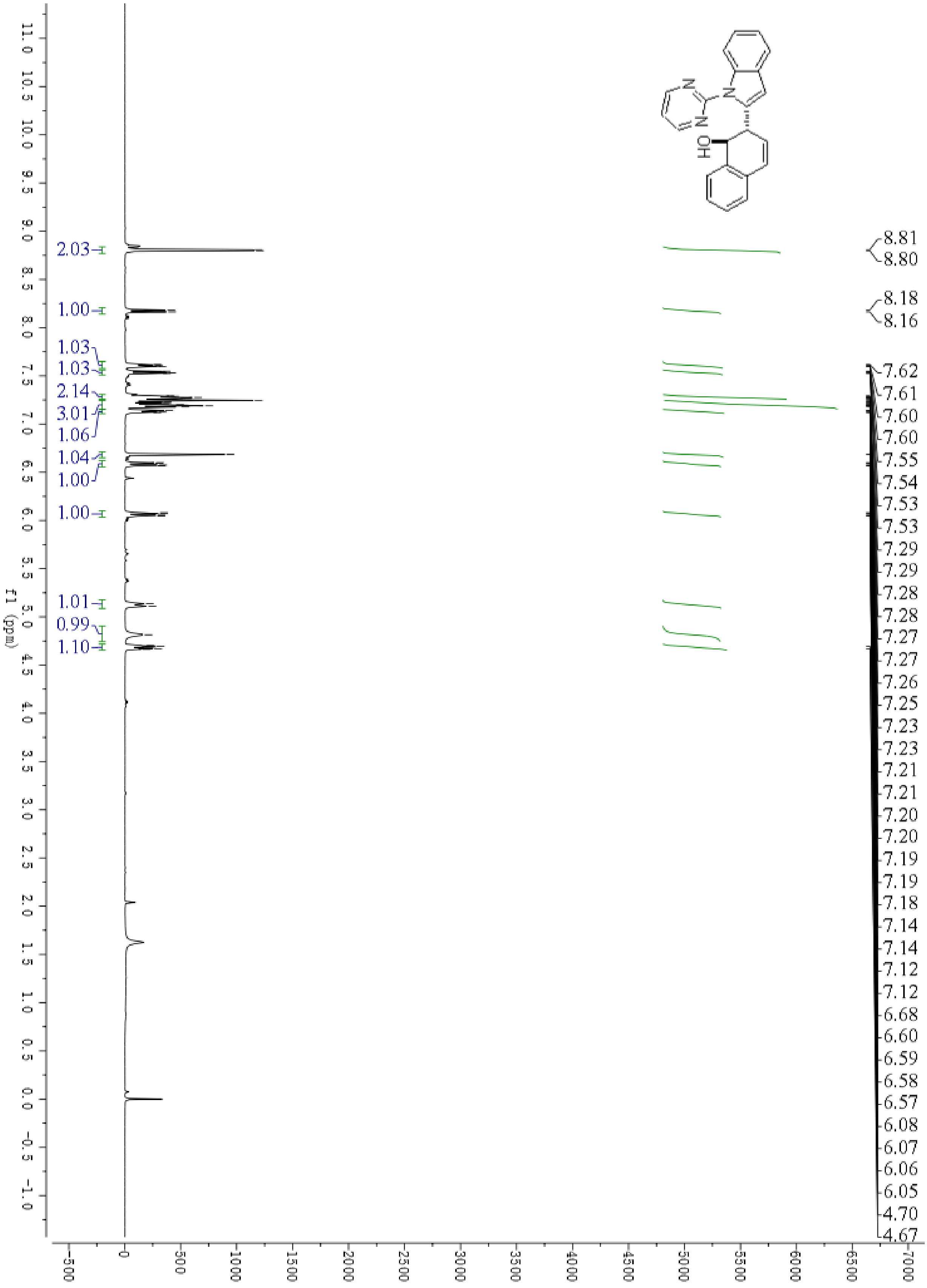

Supplementary Fig. 15 ^{13}C NMR Spectrum of *trans*-**3aa**

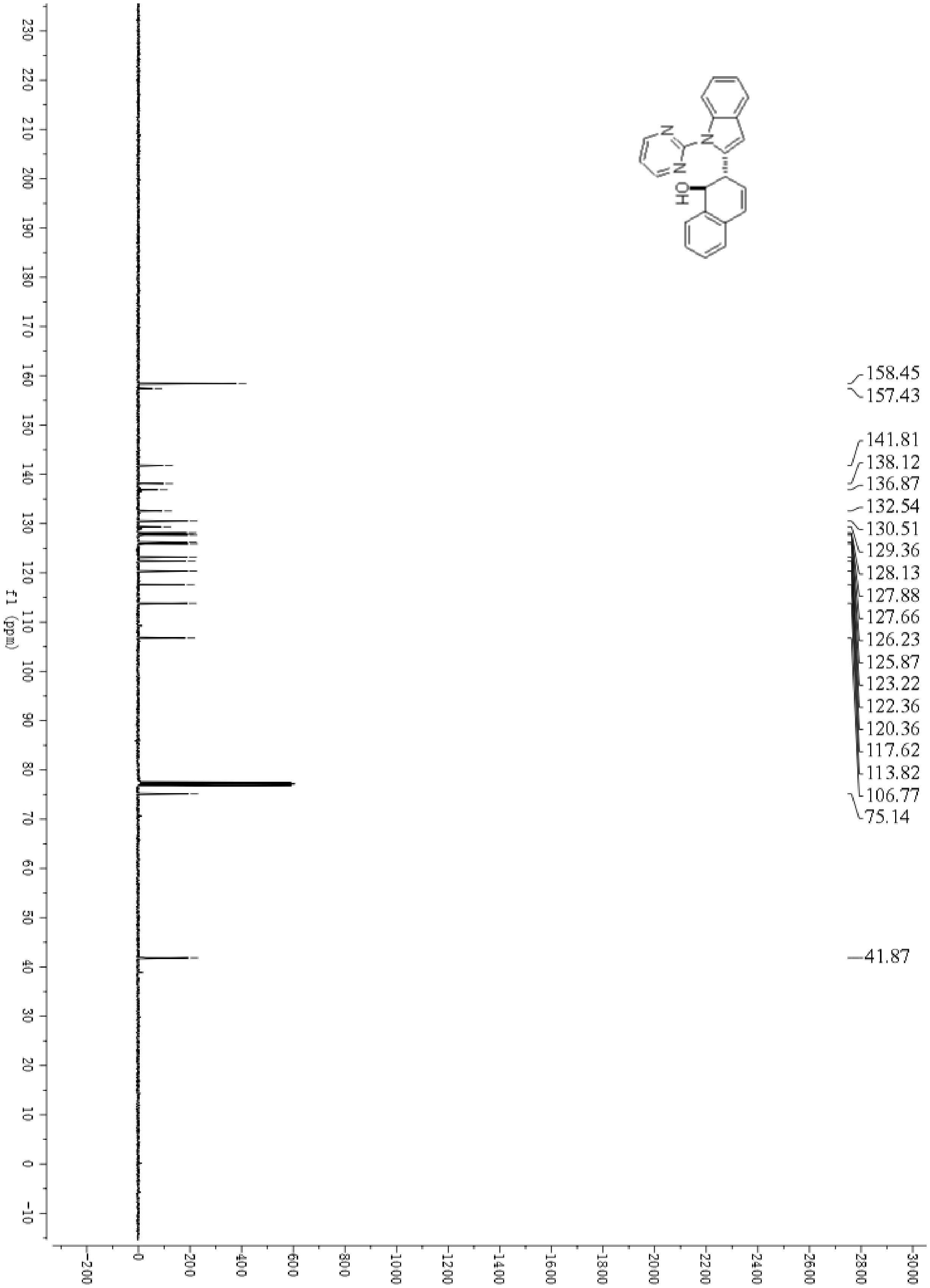

Supplementary Fig. 16 ^{1}H NMR Spectrum of *cis*-**3aa**

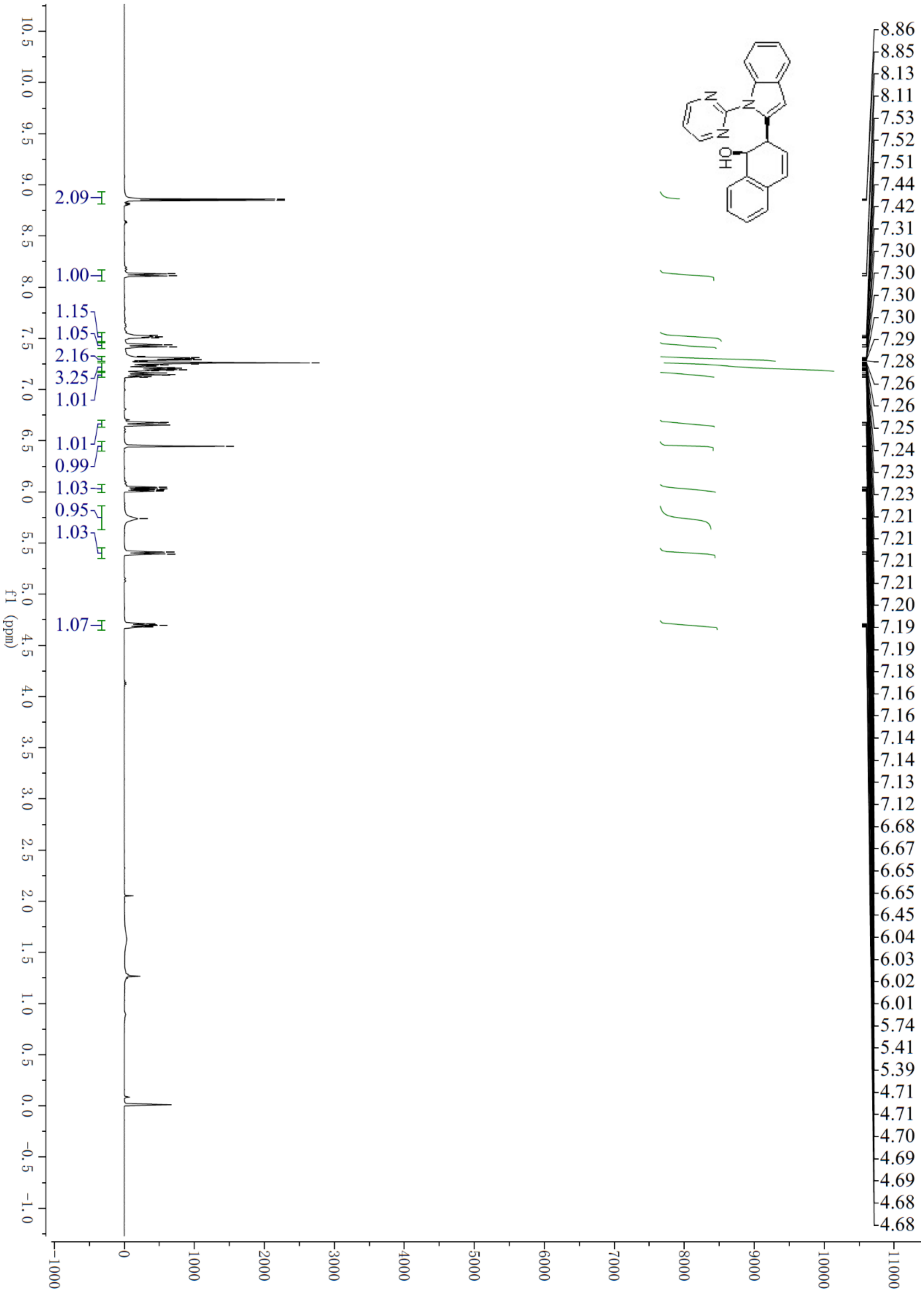

Supplementary Fig. 17 ^{13}C NMR Spectrum of *cis*-**3aa**

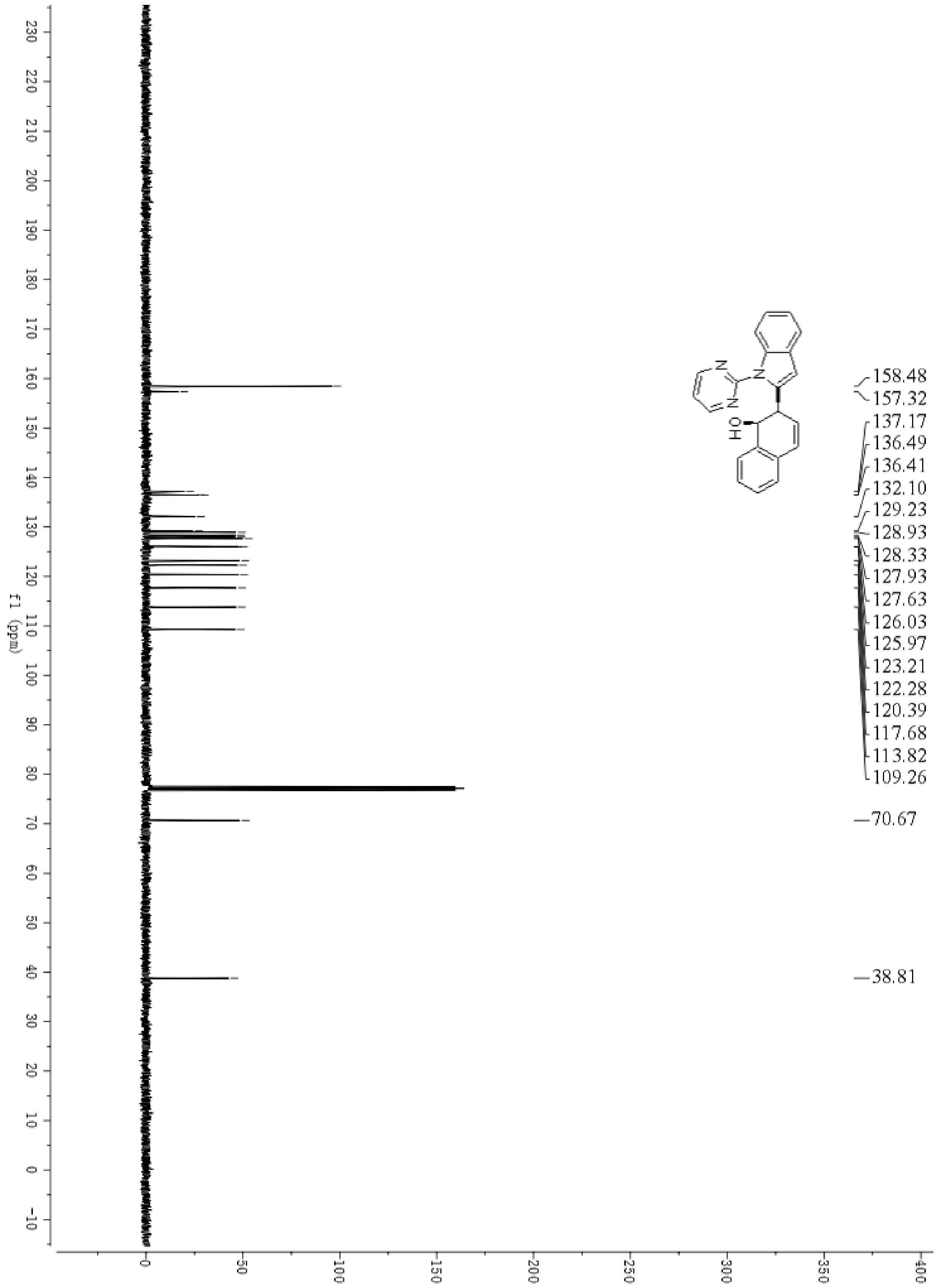

Supplementary Fig. 18 ^{1}H NMR Spectrum of *cis*-**3ba**

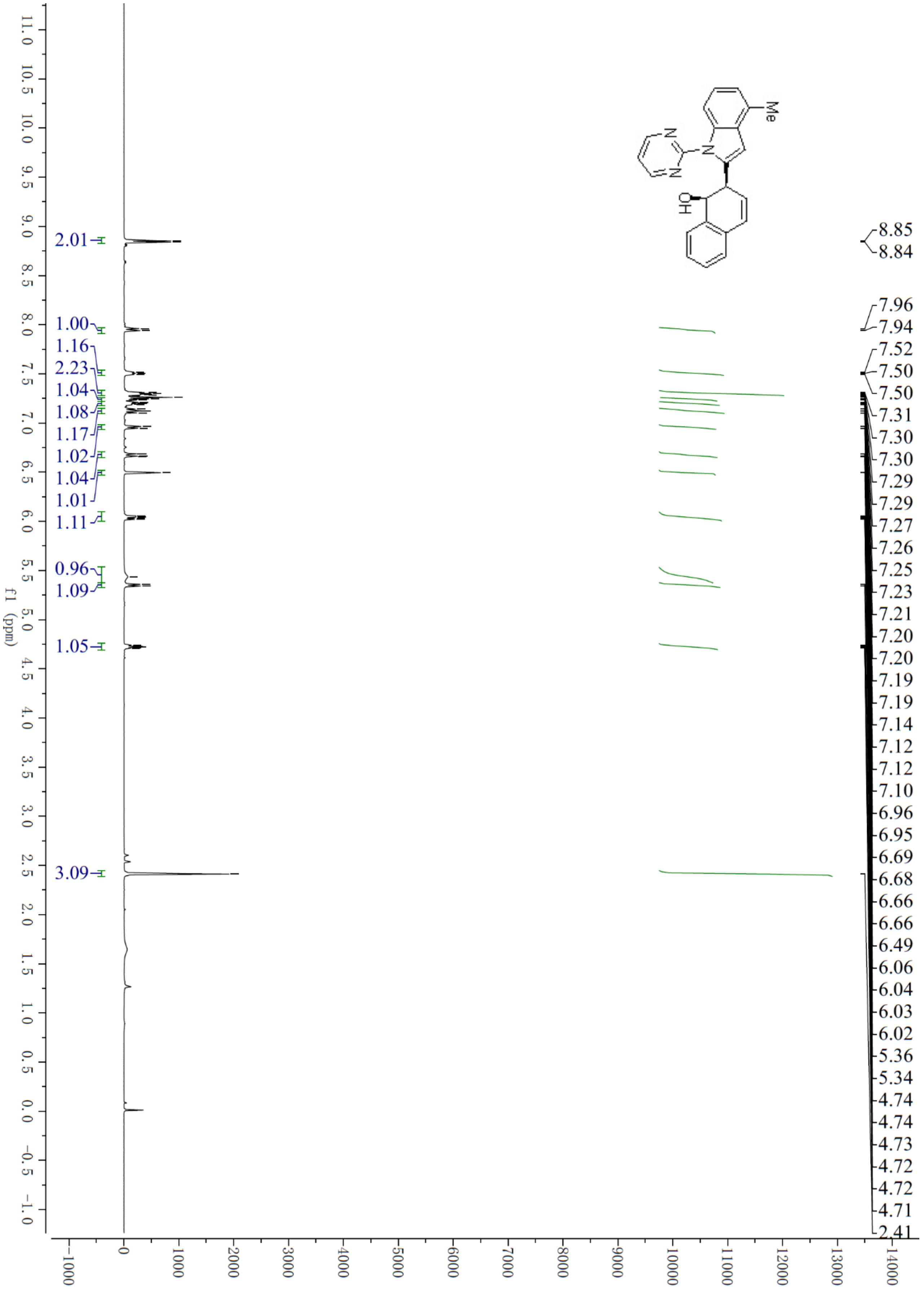

Supplementary Fig. 19 ^{13}C NMR Spectrum of *cis*-**3ba**

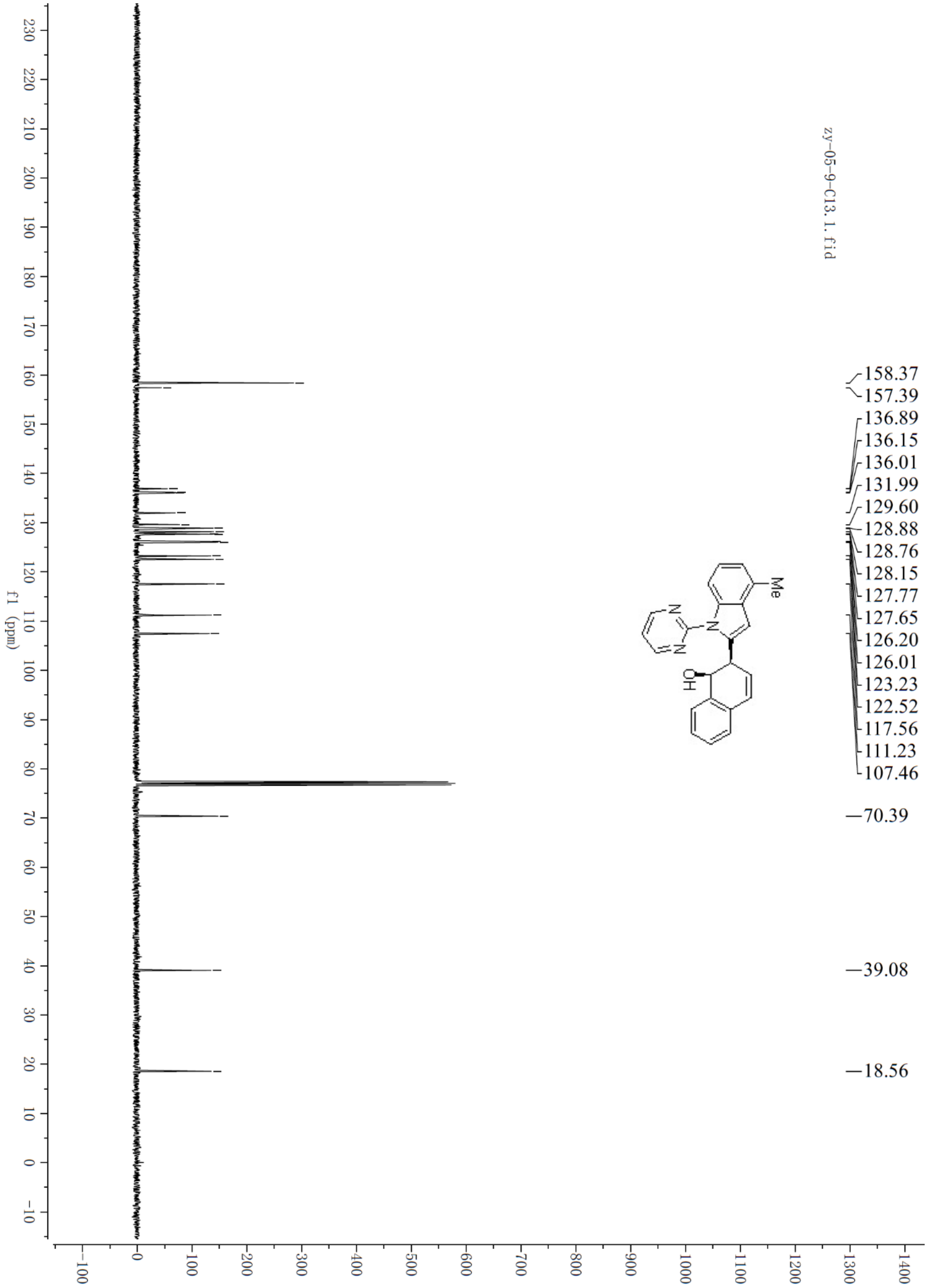

Supplementary Fig. 20 ^{1}H NMR Spectrum of *cis*-**3ca**

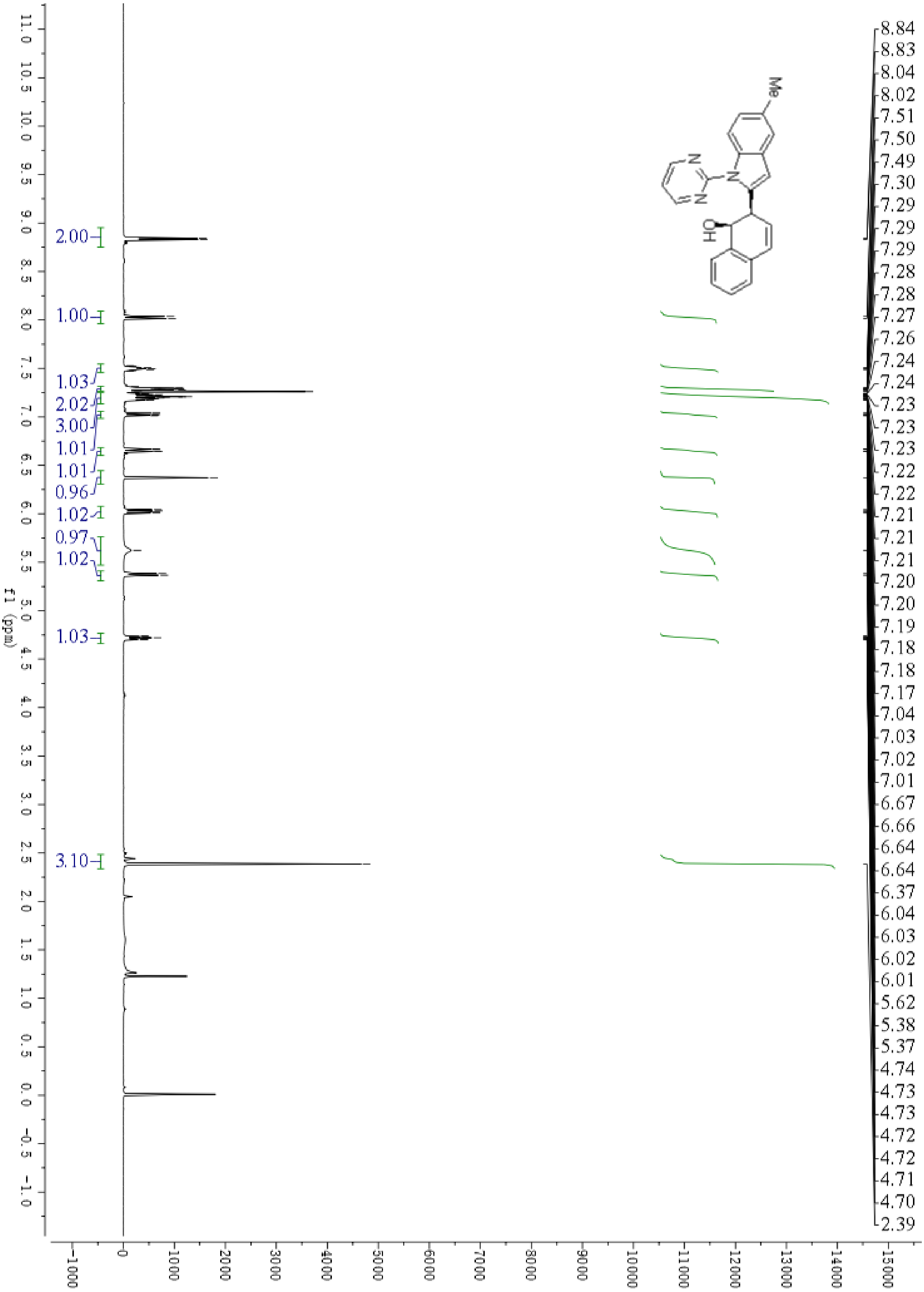

Supplementary Fig. 21 ^{13}C NMR Spectrum of *cis*-**3ca**

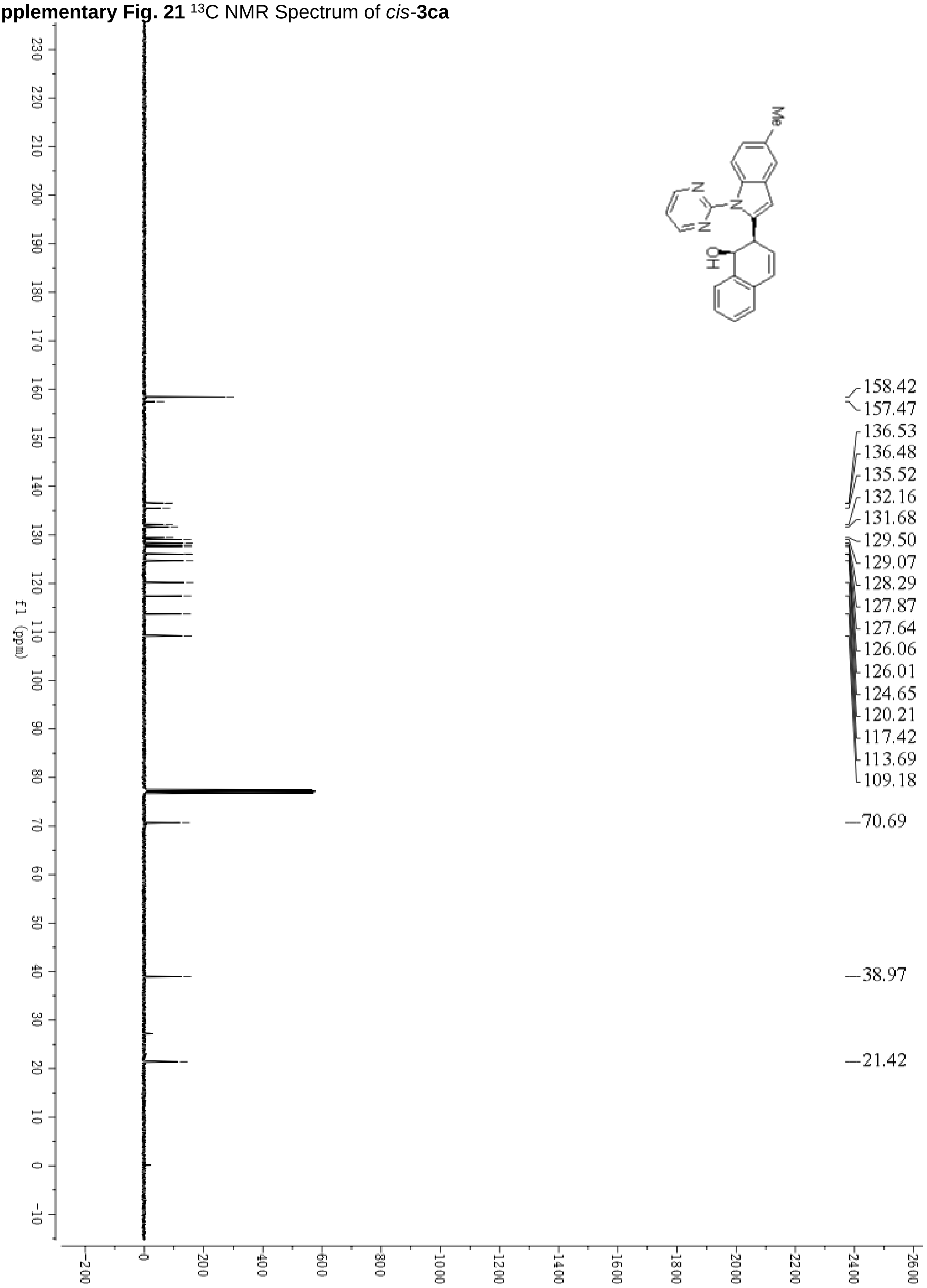

Supplementary Fig. 22 ¹H NMR Spectrum of *cis*-**3da**

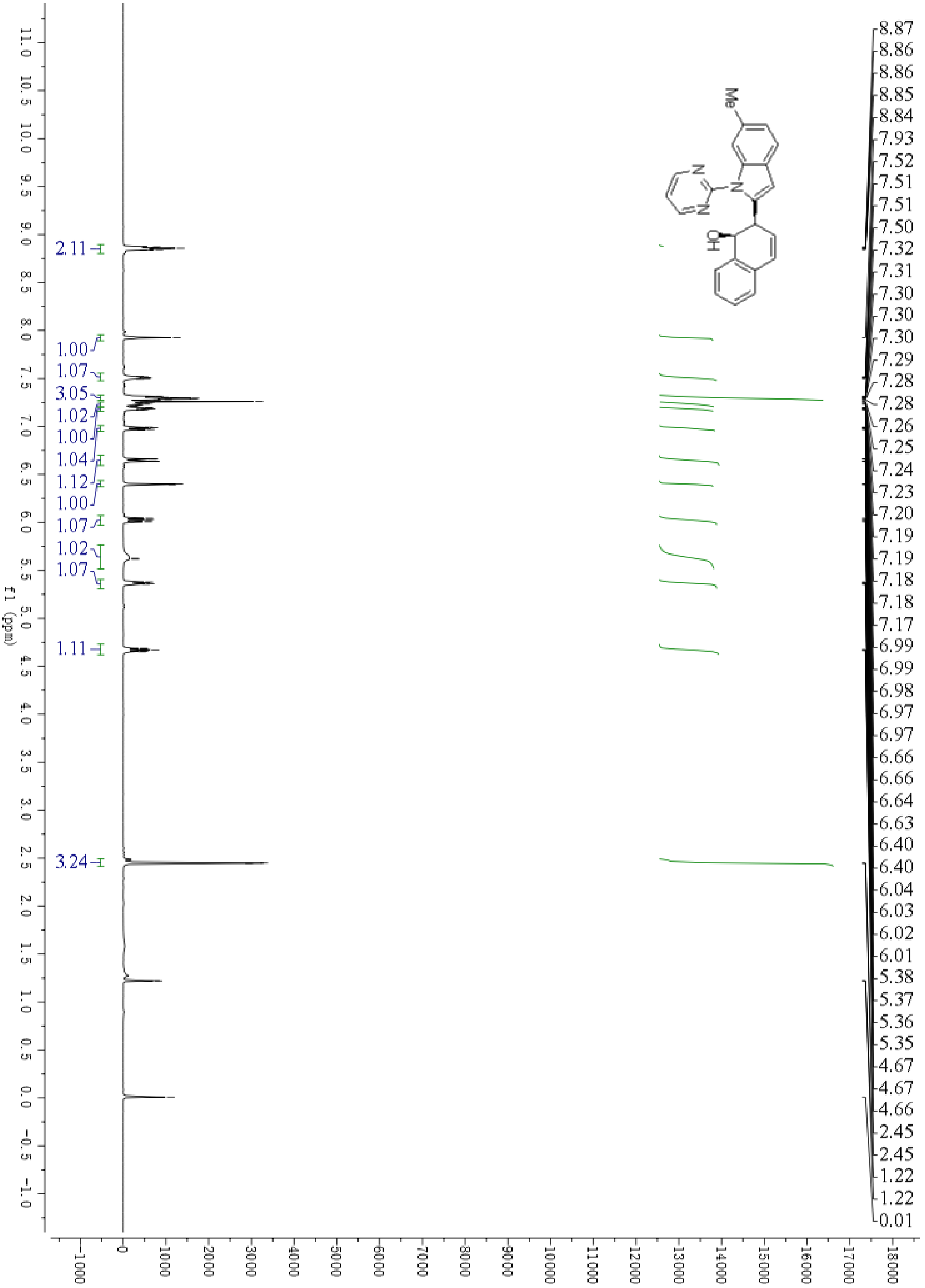

Supplementary Fig. 23 ^{13}C NMR Spectrum of *cis*-**3da**

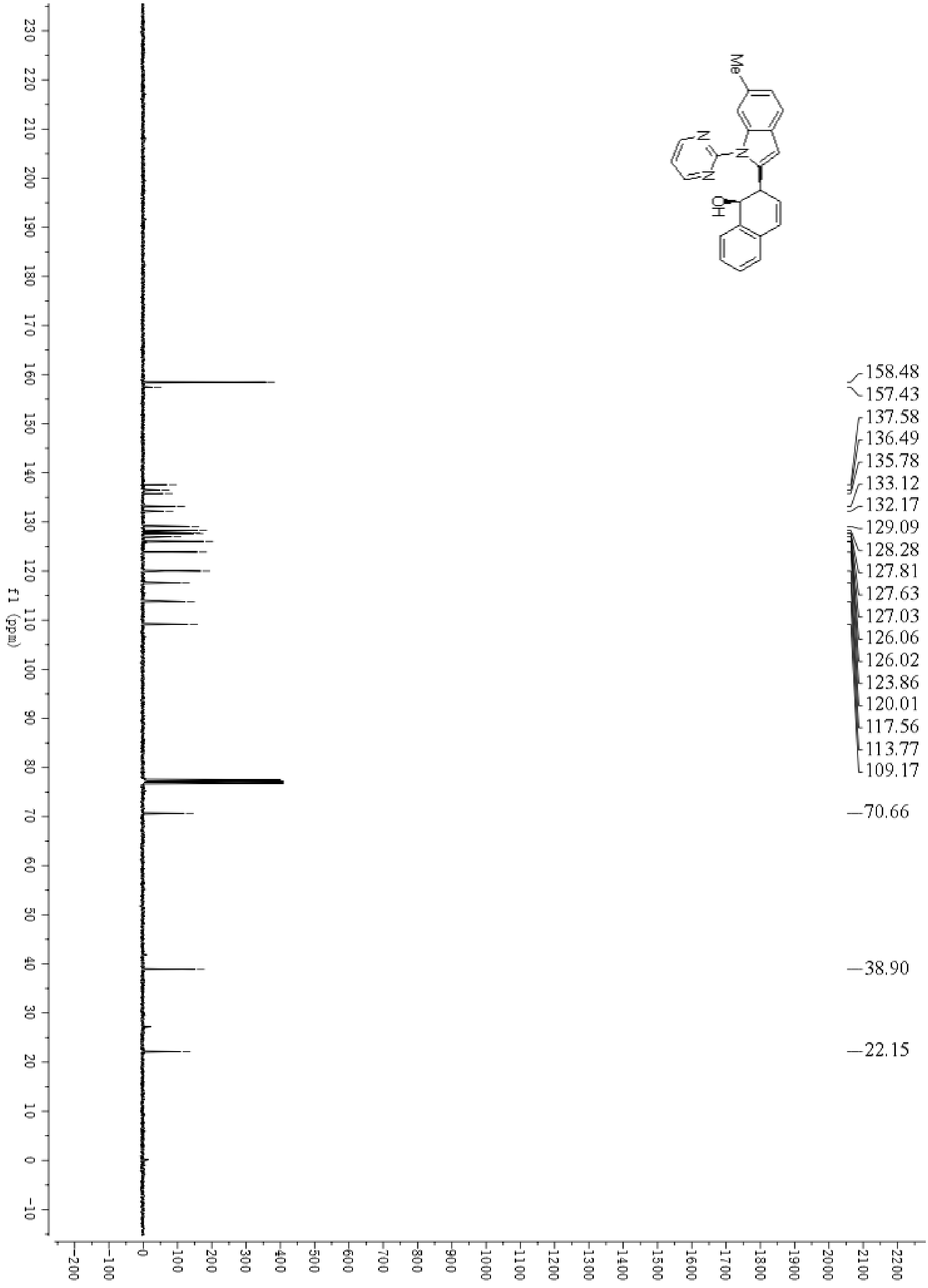

Supplementary Fig. 24 ^{1}H NMR Spectrum of *cis*-**3ea**

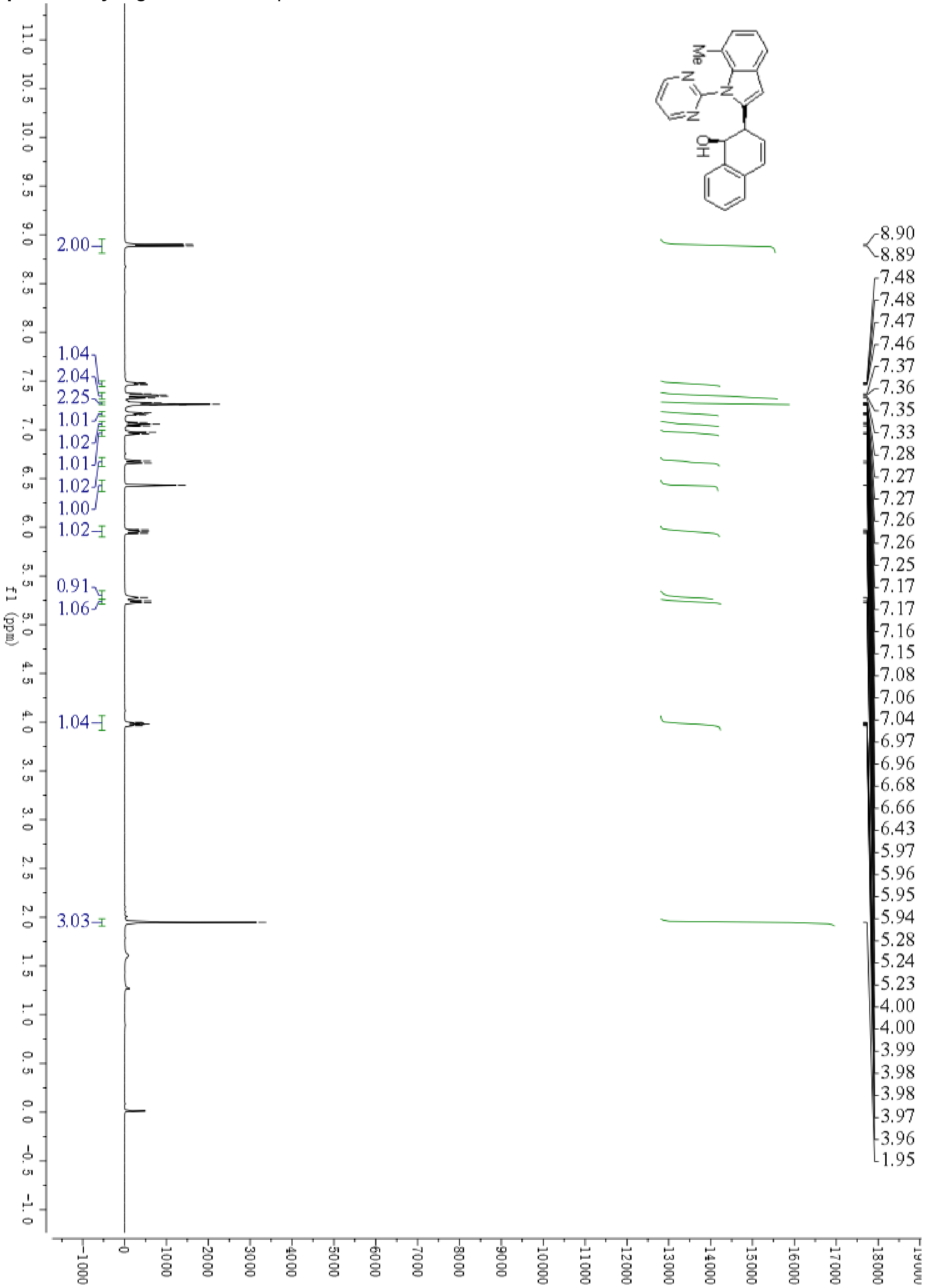

Supplementary Fig. 25 ^{13}C NMR Spectrum of *cis*-**3ea**

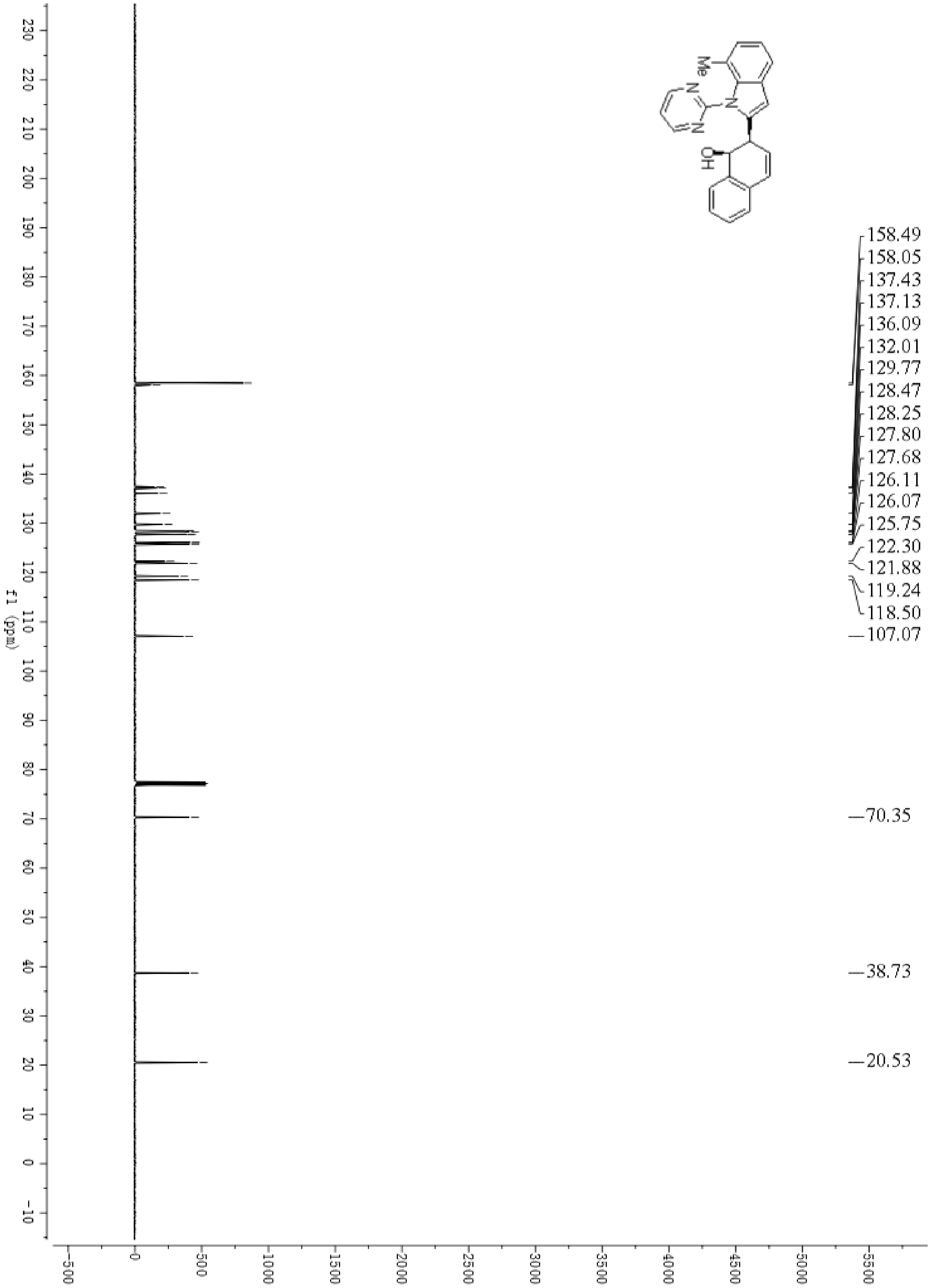

Supplementary Fig. 26 ^{1}H NMR Spectrum of *cis*-**3fa**

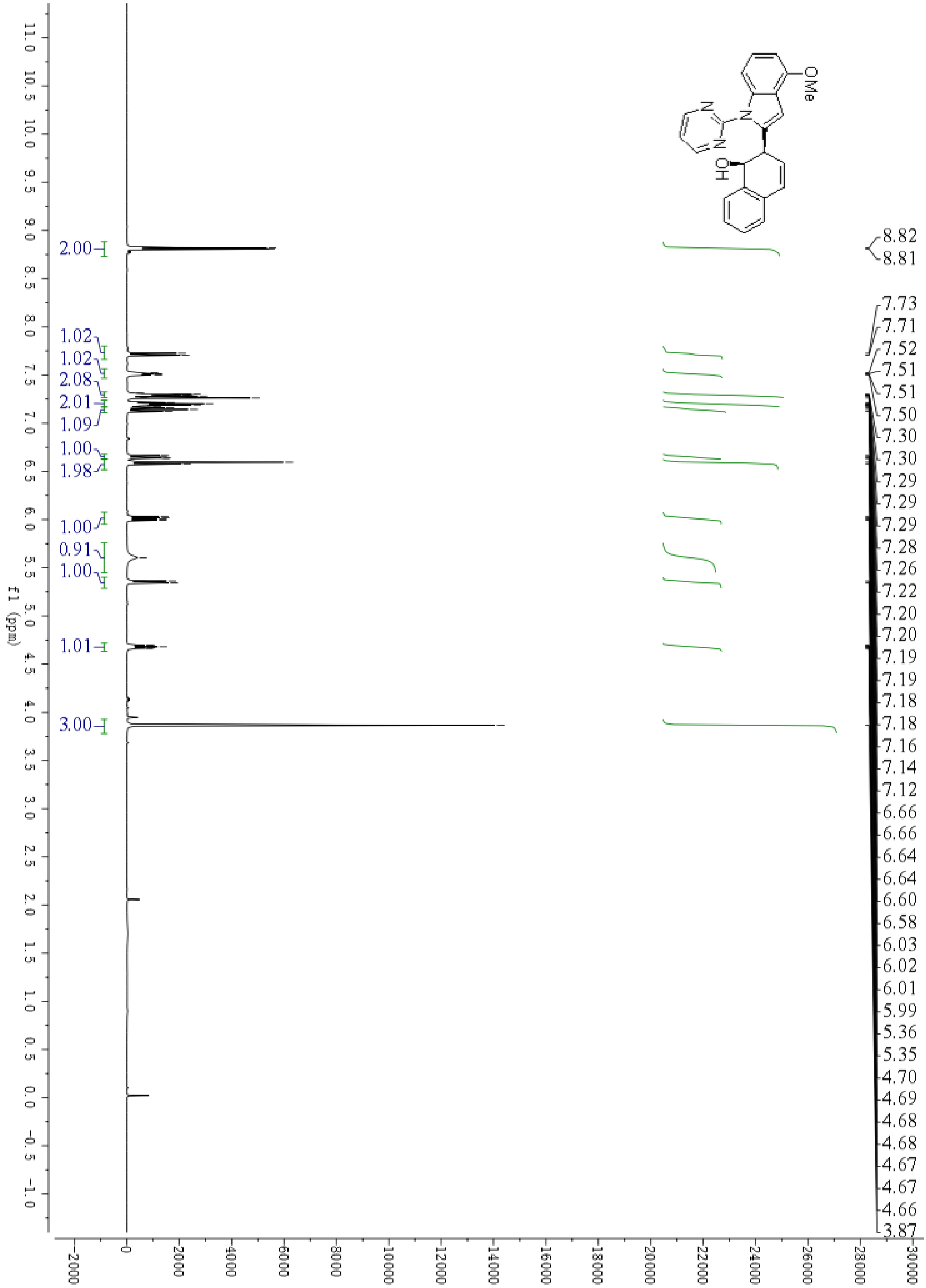

Supplementary Fig. 27 ^{13}C NMR Spectrum of *cis*-**3fa**

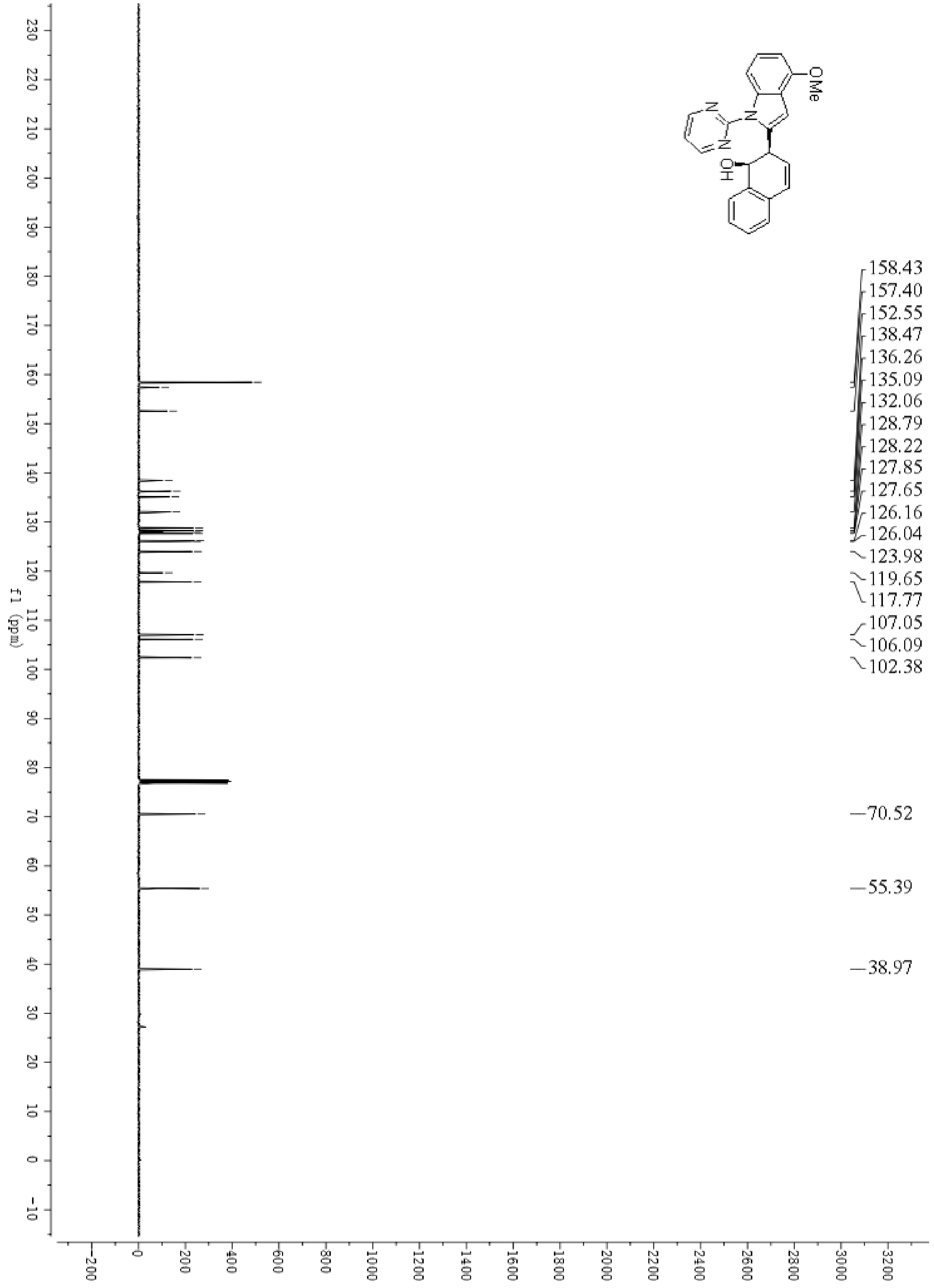

Supplementary Fig. 28 ^{1}H NMR Spectrum of *cis*-**3ga**

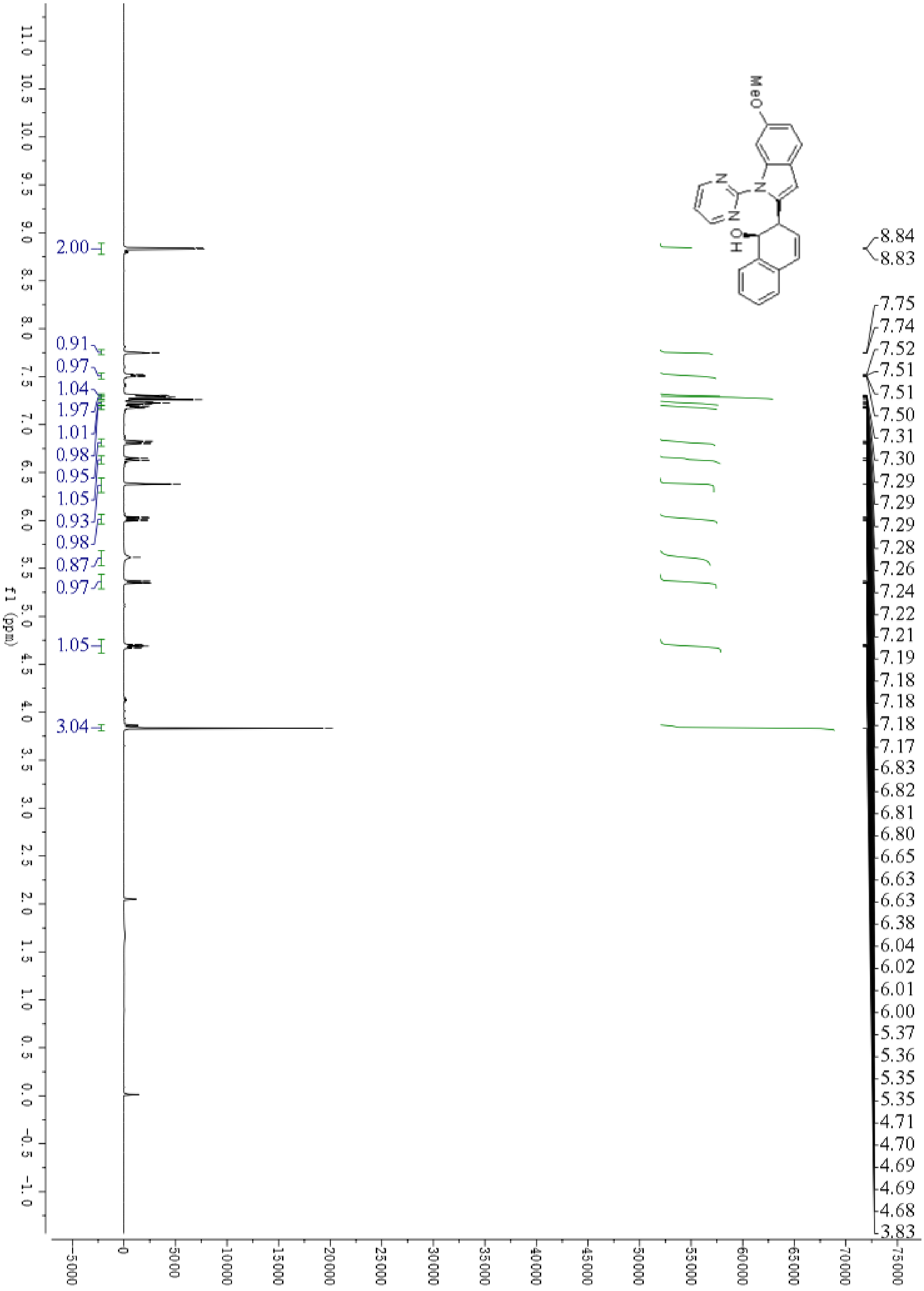

Supplementary Fig. 29 ^{13}C NMR Spectrum of *cis*-**3ga**

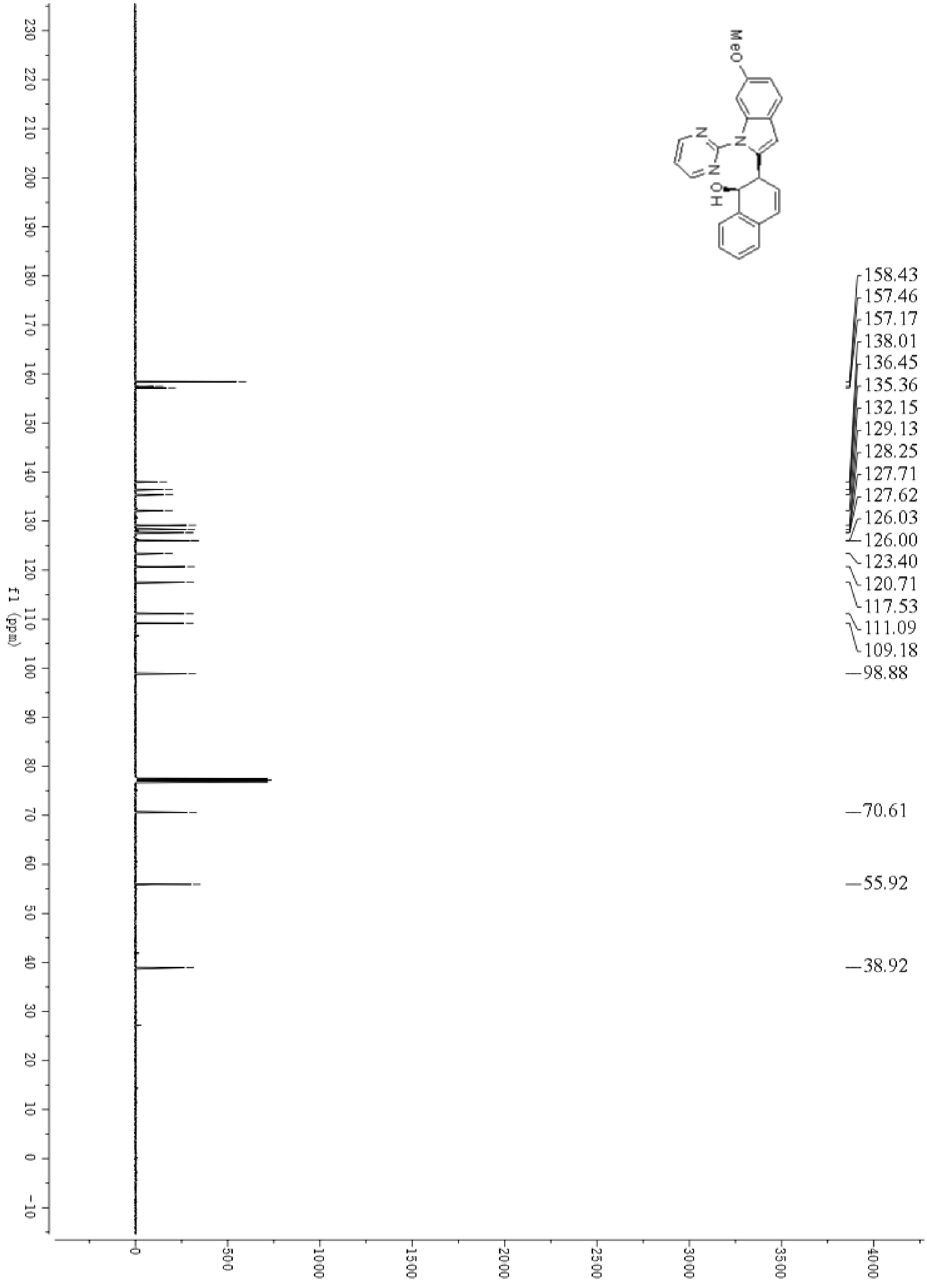

Supplementary Fig. 30 ^{1}H NMR Spectrum of *cis*-**3ha**

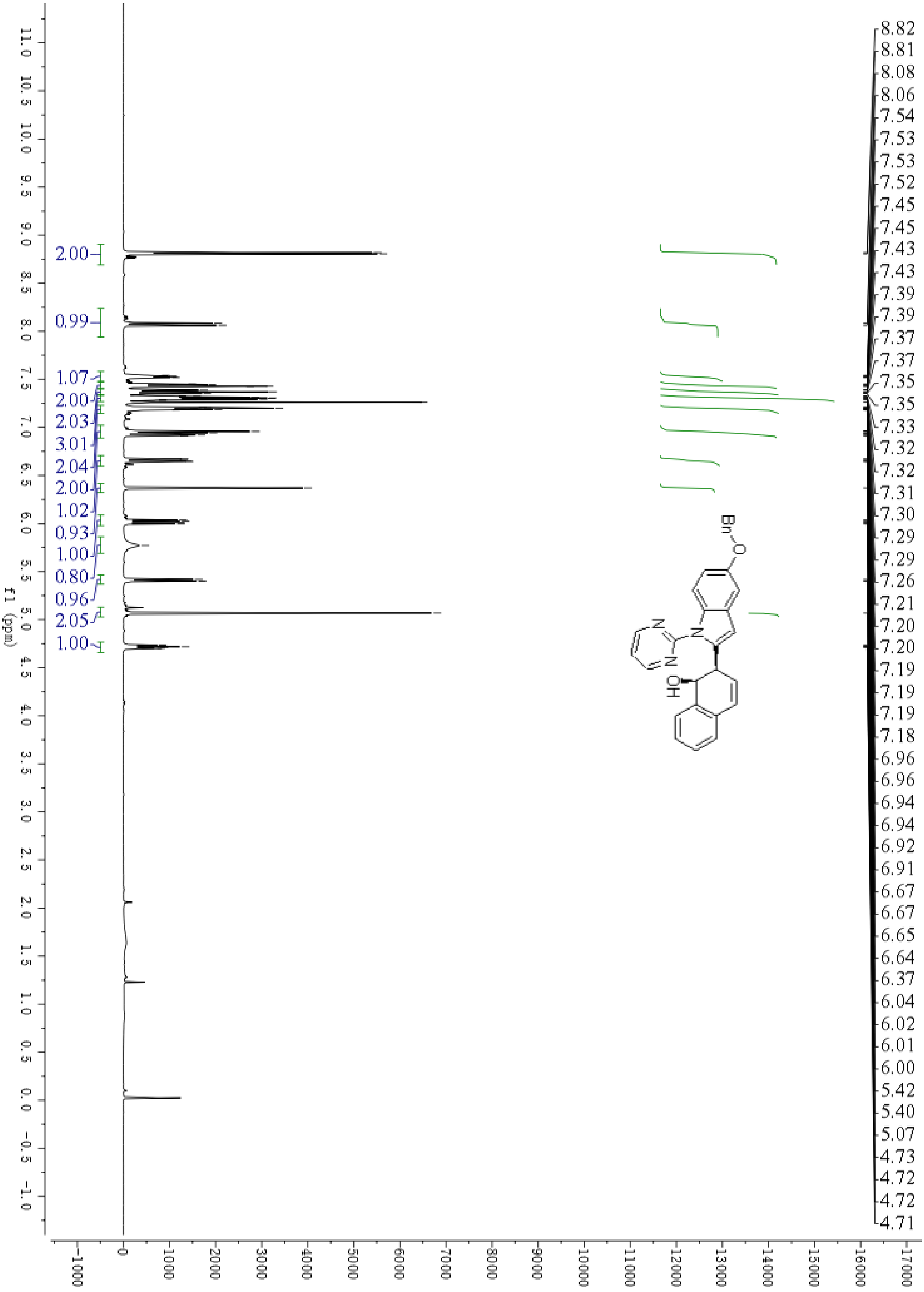

Supplementary Fig. 31 ^{13}C NMR Spectrum of *cis*-**3ha**

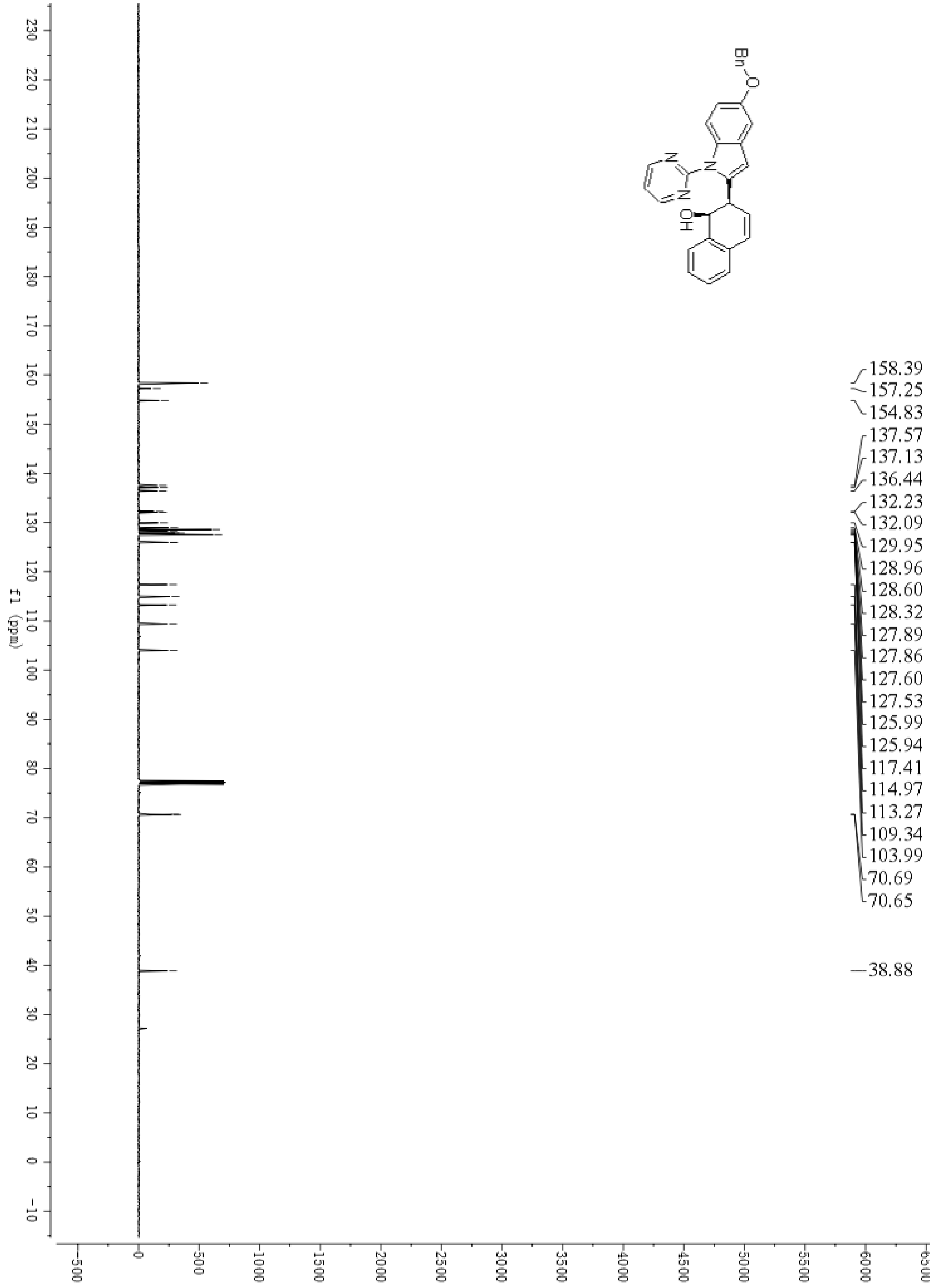

Supplementary Fig. 32 ^{1}H NMR Spectrum of *cis*-**3ia**

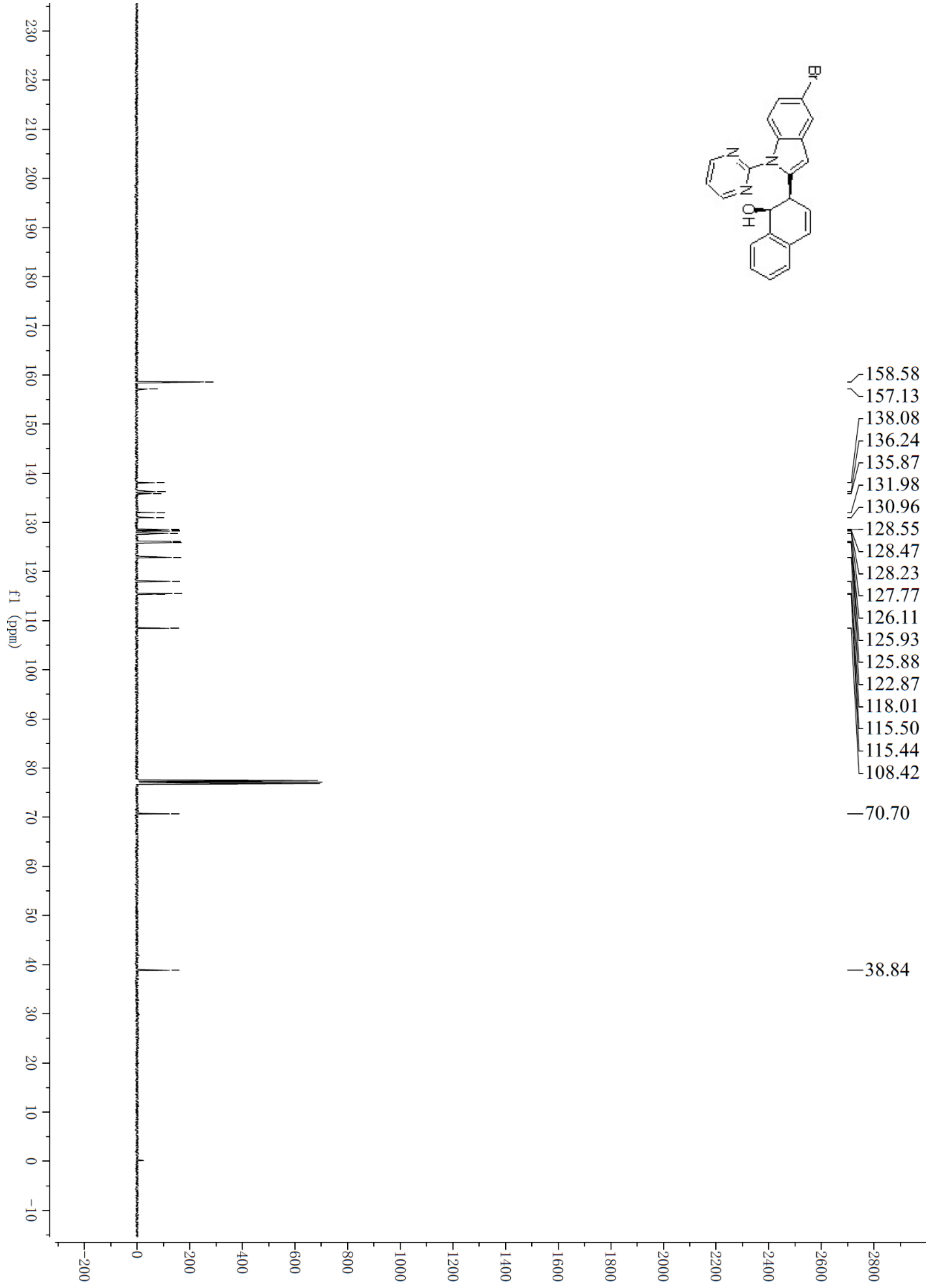

Supplementary Fig. 33 ^{13}C NMR Spectrum of *cis*-**3ia**

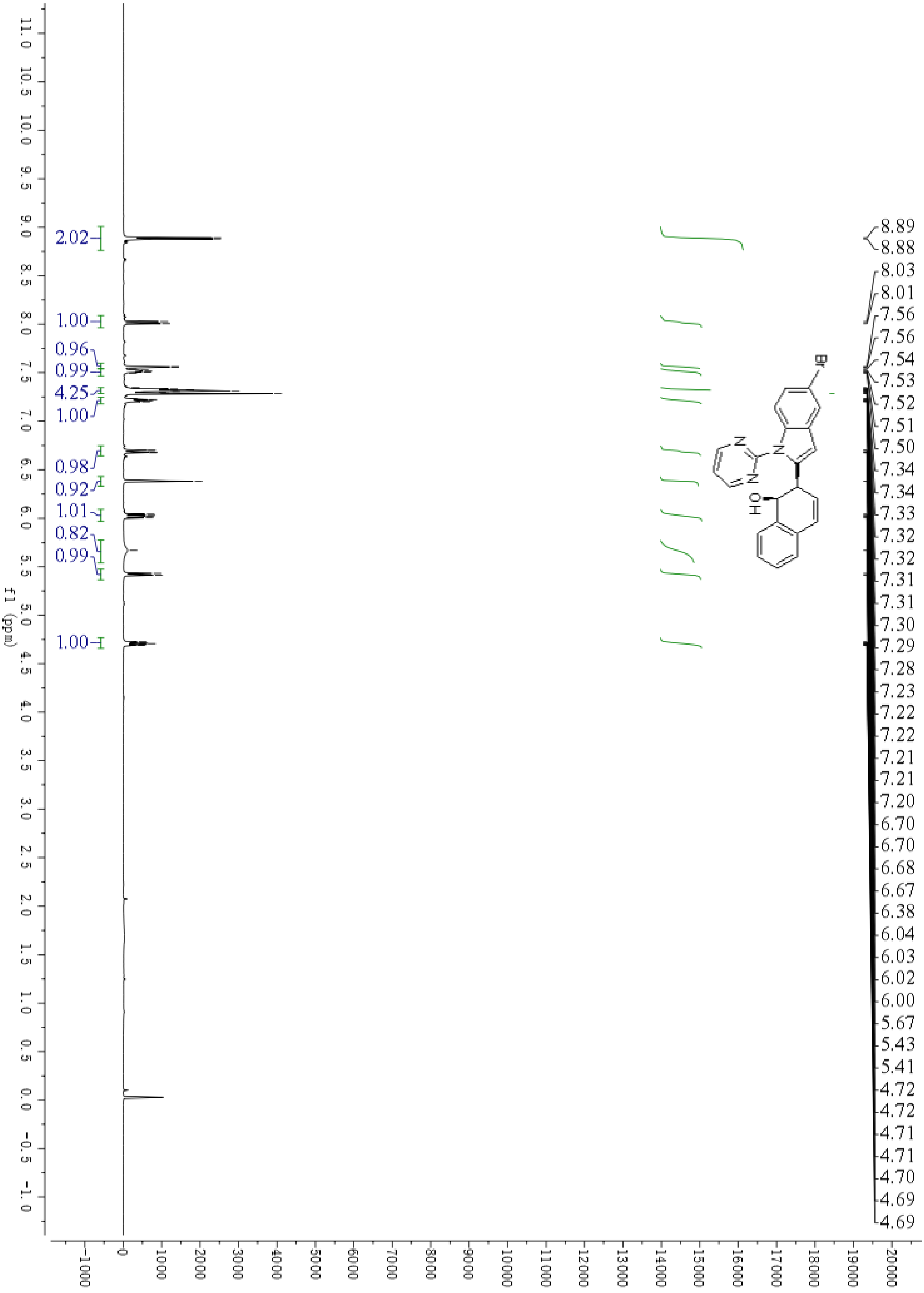

Supplementary Fig. 34 ^{1}H NMR Spectrum of *cis*-**3ja**

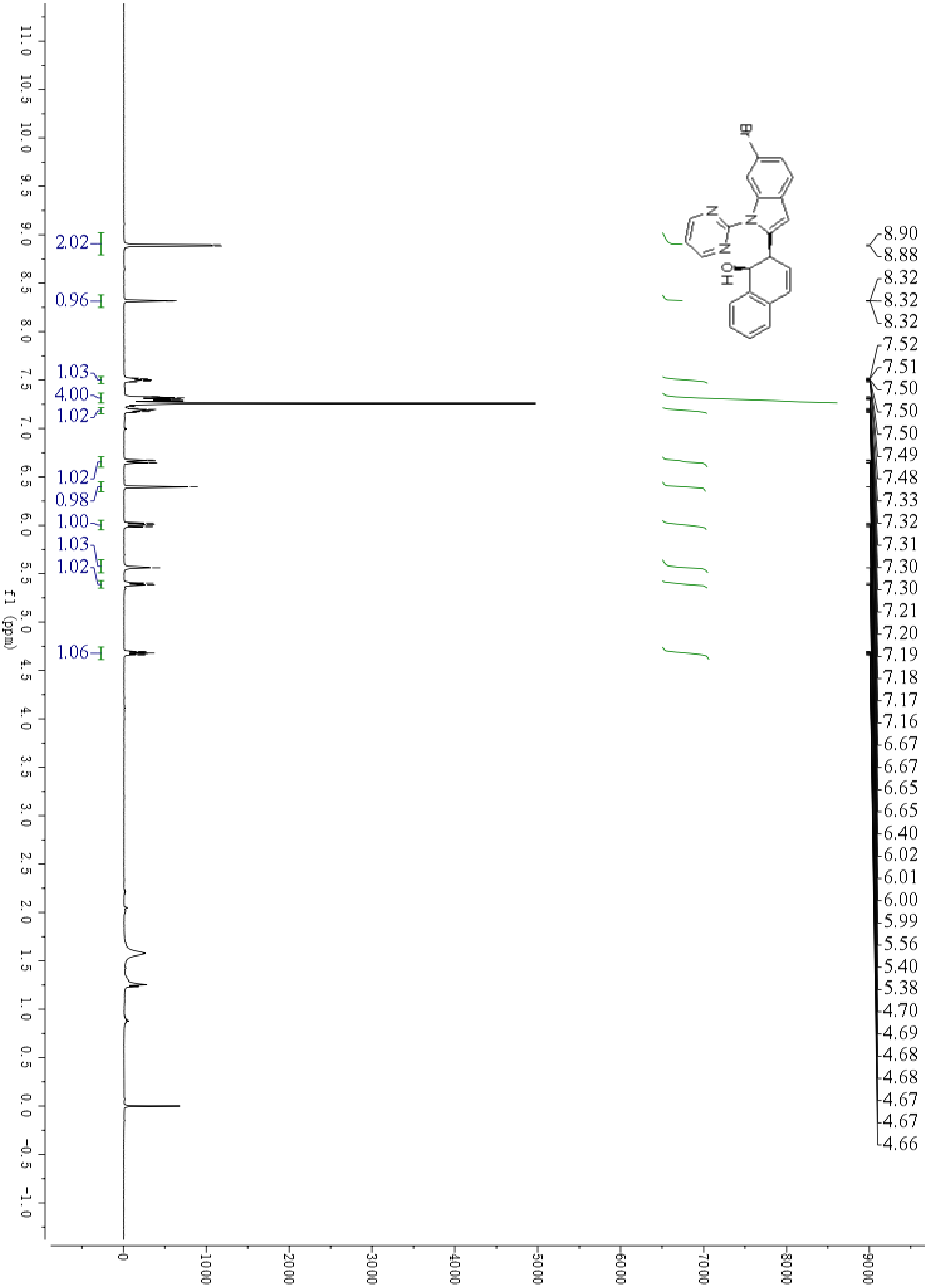

Supplementary Fig. 35 ^{13}C NMR Spectrum of *cis*-**3ja**

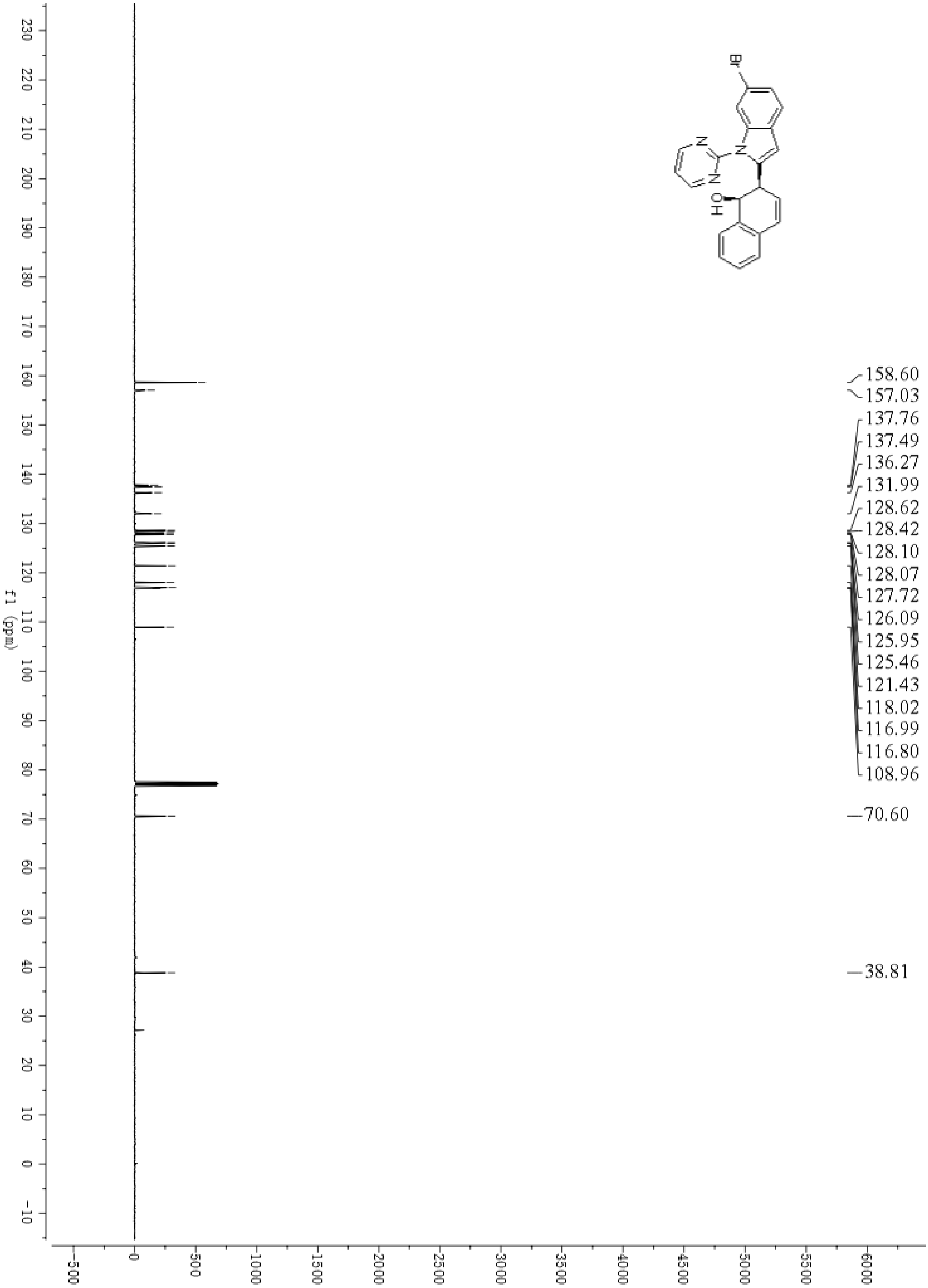

Supplementary Fig. 36 ^{1}H NMR Spectrum of *cis*-**3ka**

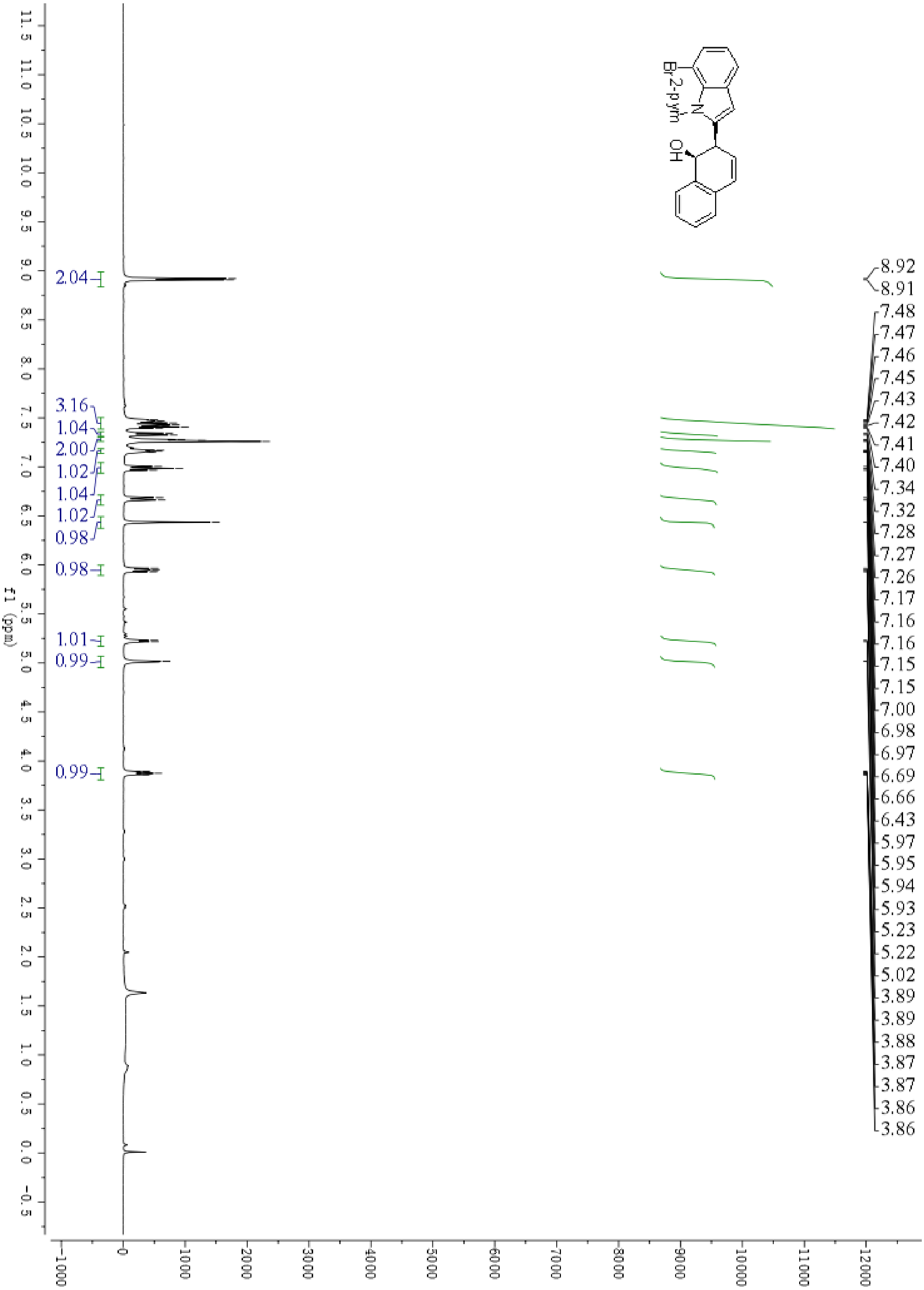

Supplementary Fig. 37 ^{13}C NMR Spectrum of *cis*-**3ka**

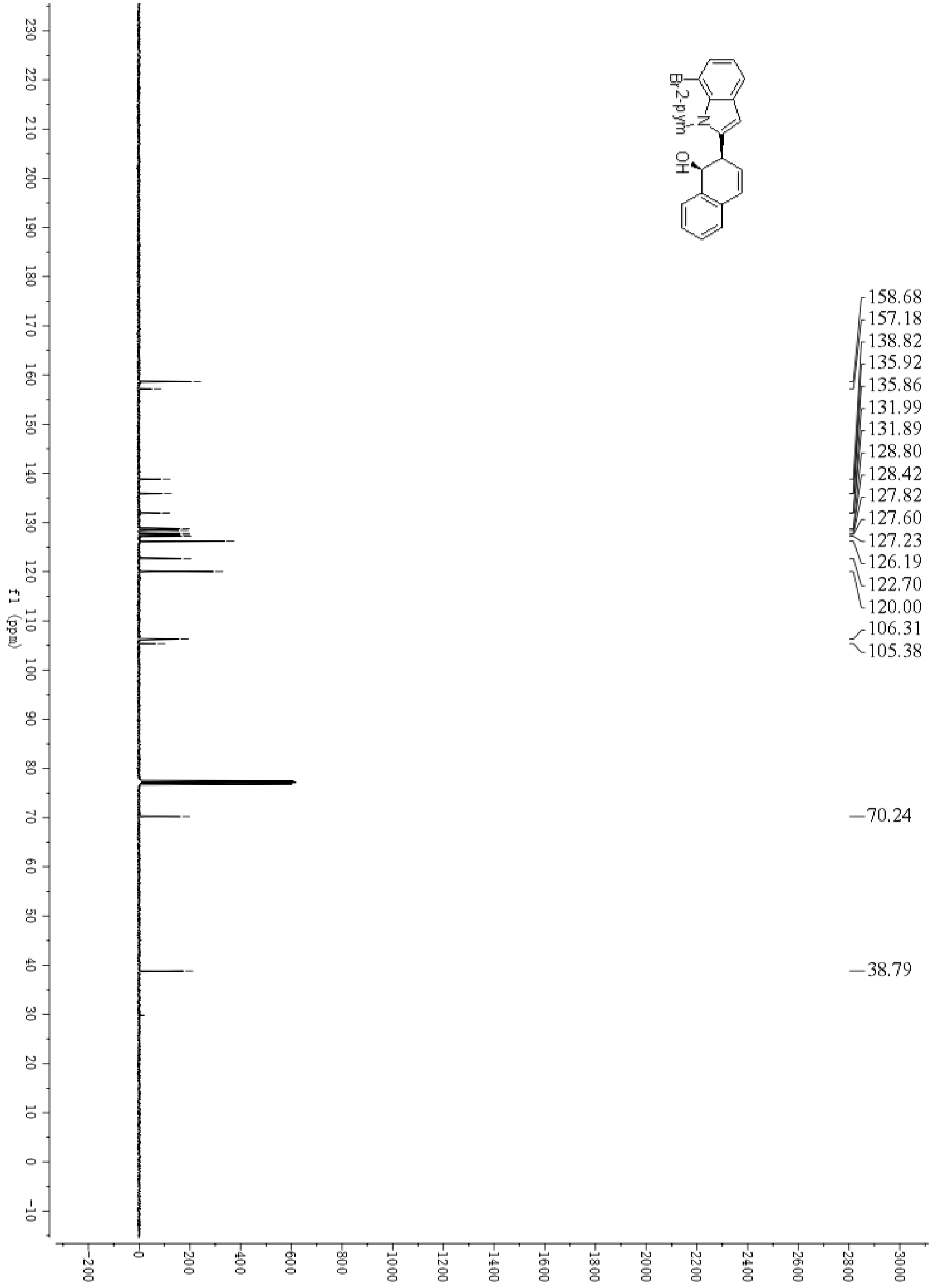

Supplementary Fig. 38 ^{1}H NMR Spectrum of *cis*-**3la**

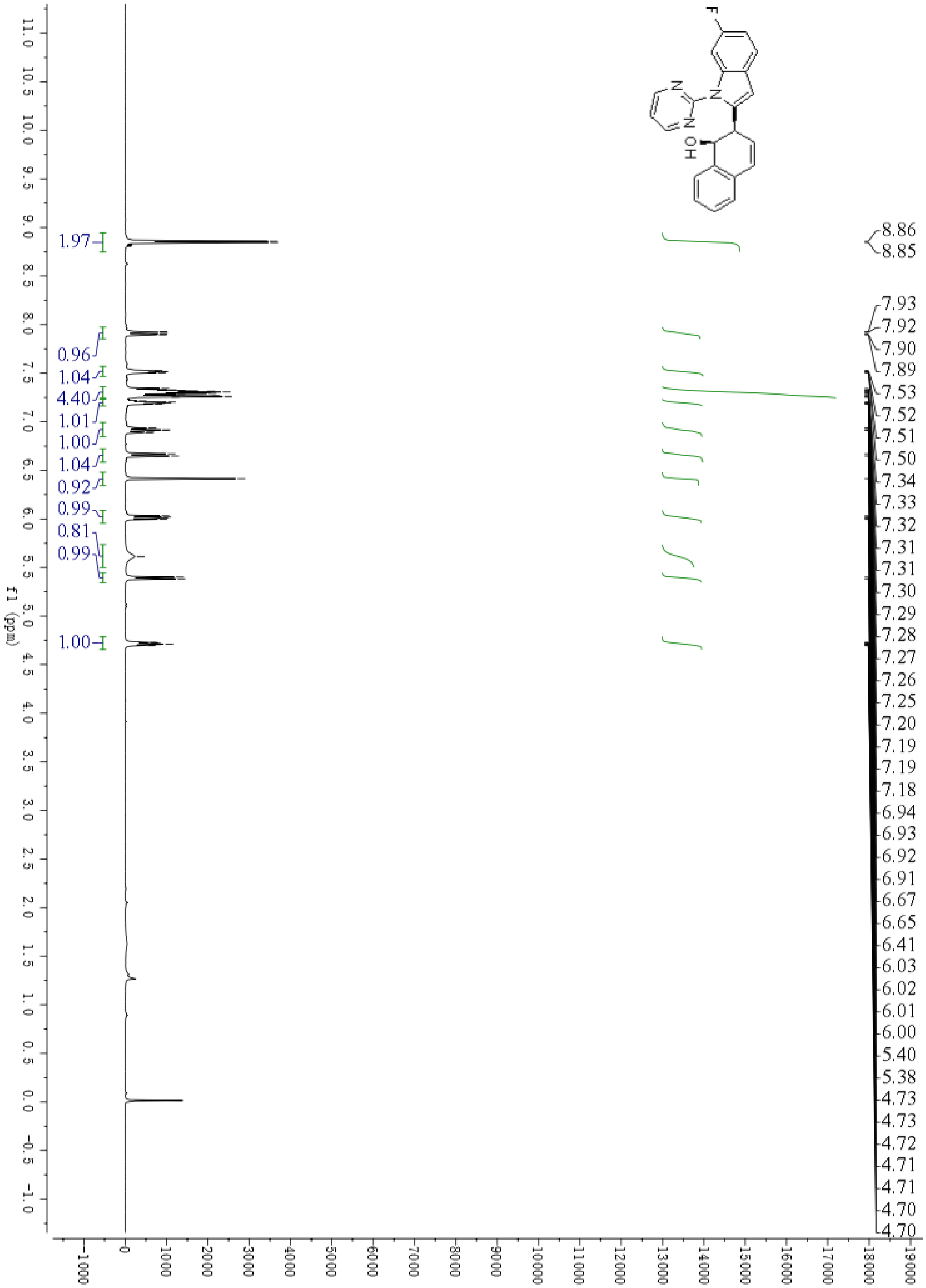

Supplementary Fig. 39 ^{13}C NMR Spectrum of *cis*-**3la**

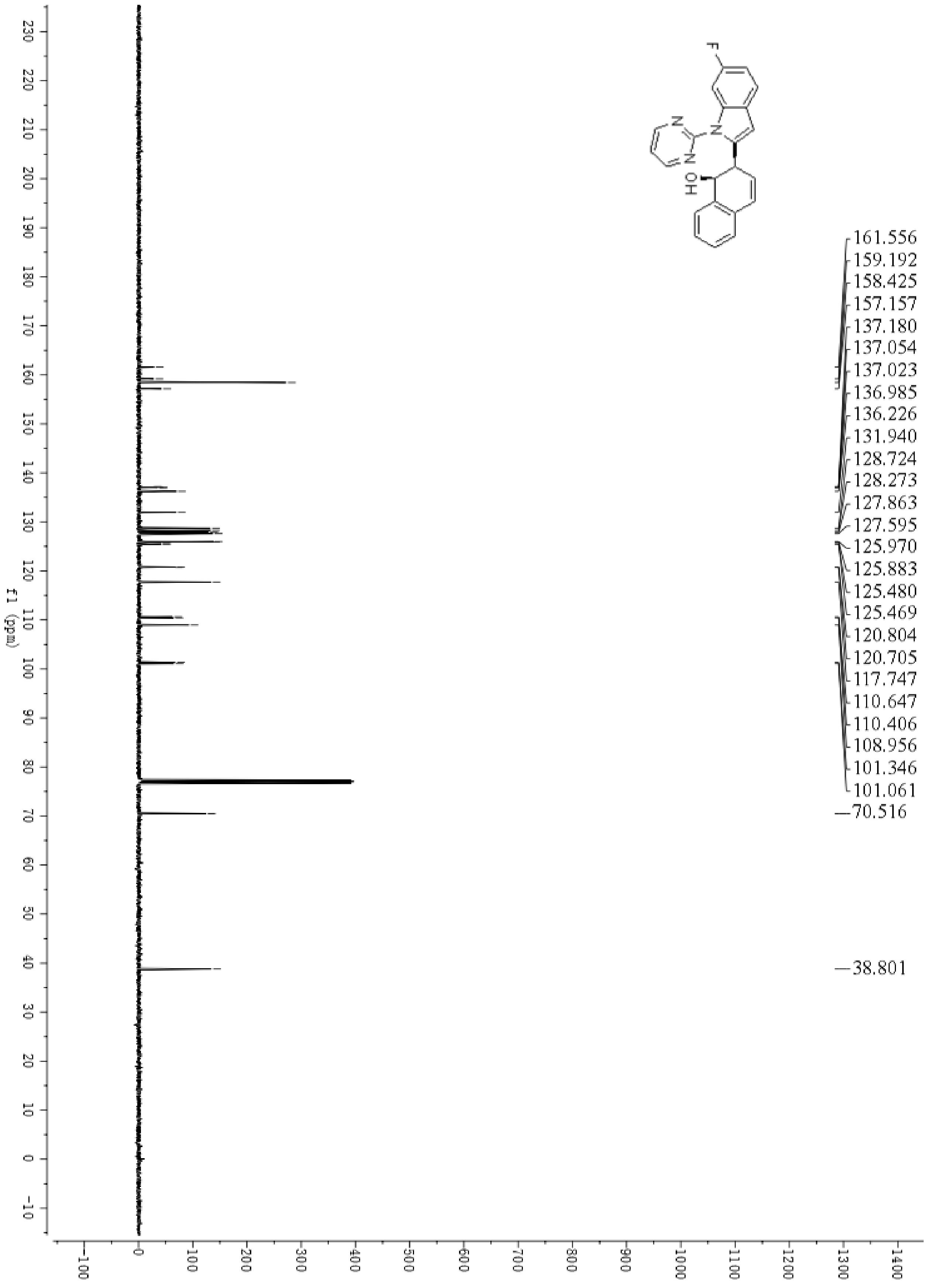

Supplementary Fig. 40 ^{19}F NMR Spectrum of *cis*-**3la**

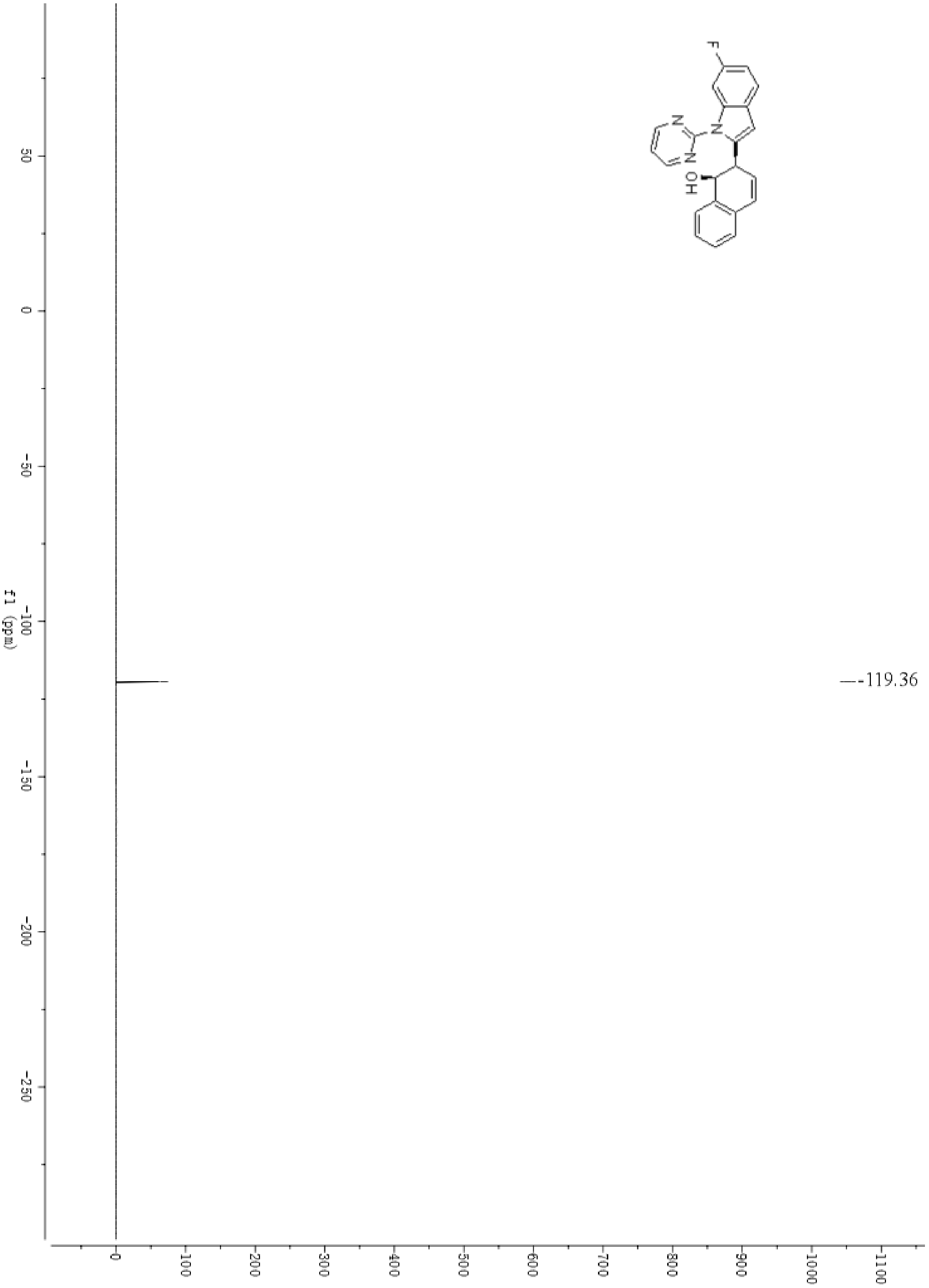

Supplementary Fig. 41 ^{1}H NMR Spectrum of *cis*-**3ma**

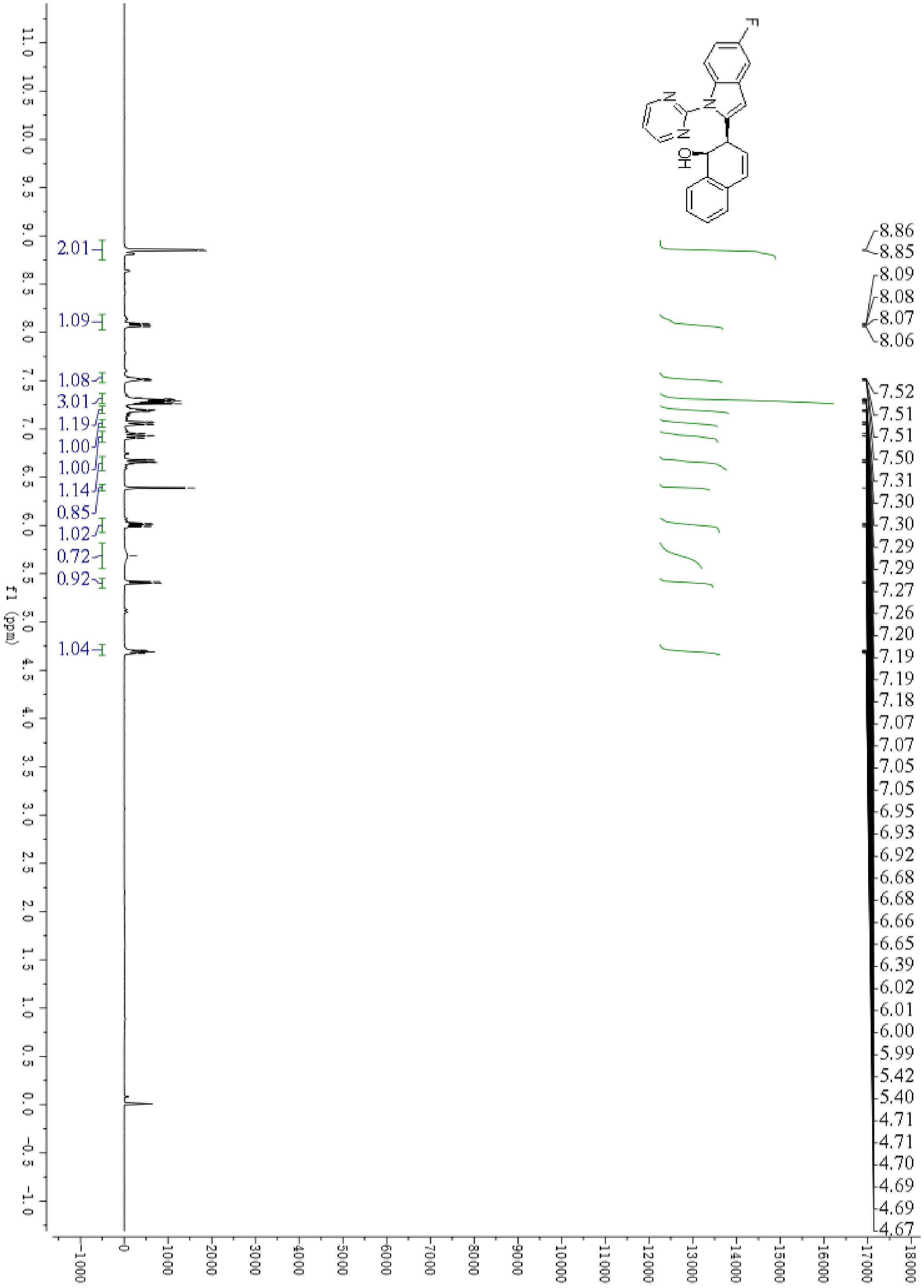

Supplementary Fig. 42 ^{13}C NMR Spectrum of *cis*-**3ma**

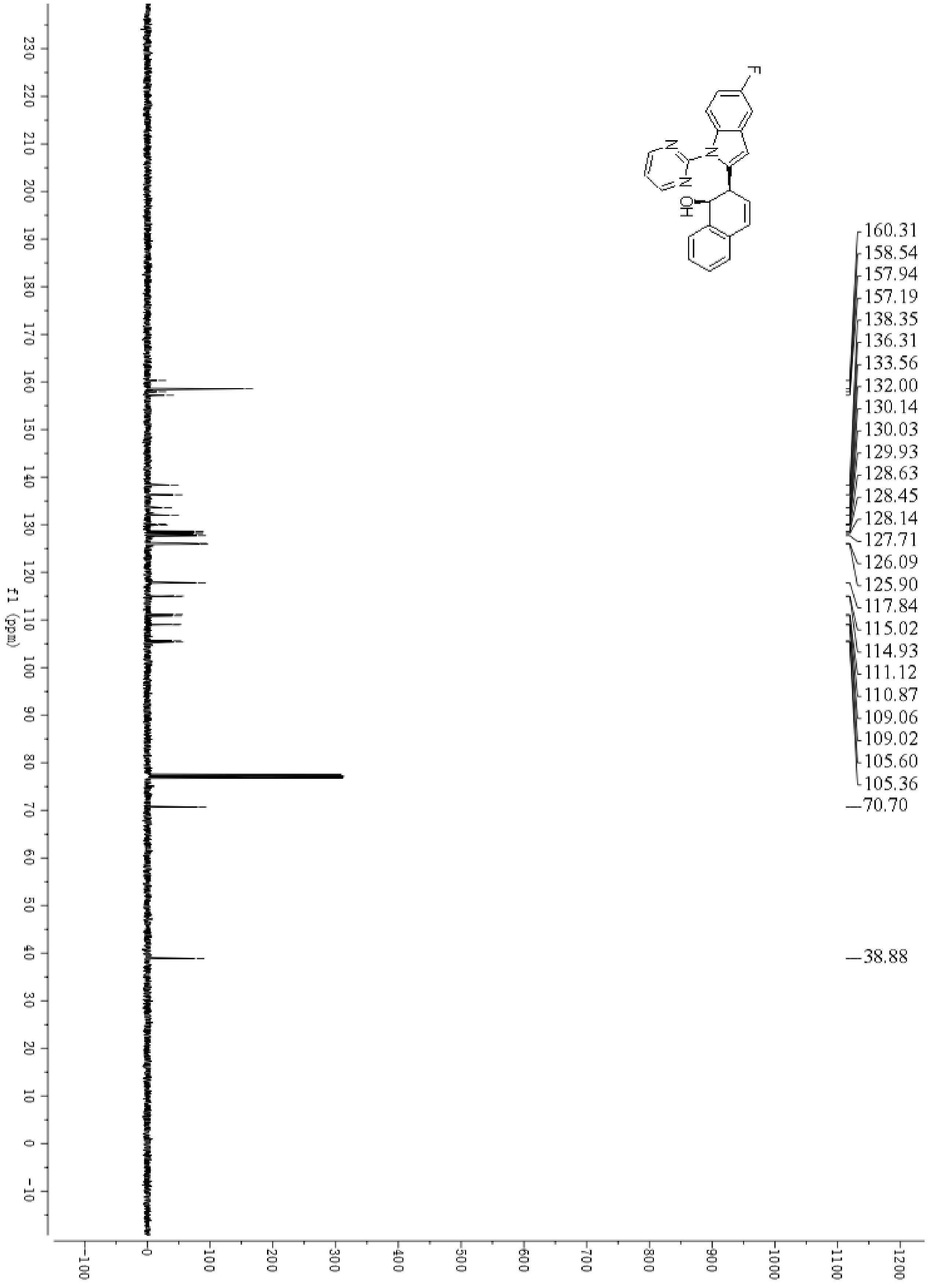

Supplementary Fig. 43 ^{19}F NMR Spectrum of *cis*-**3ma**

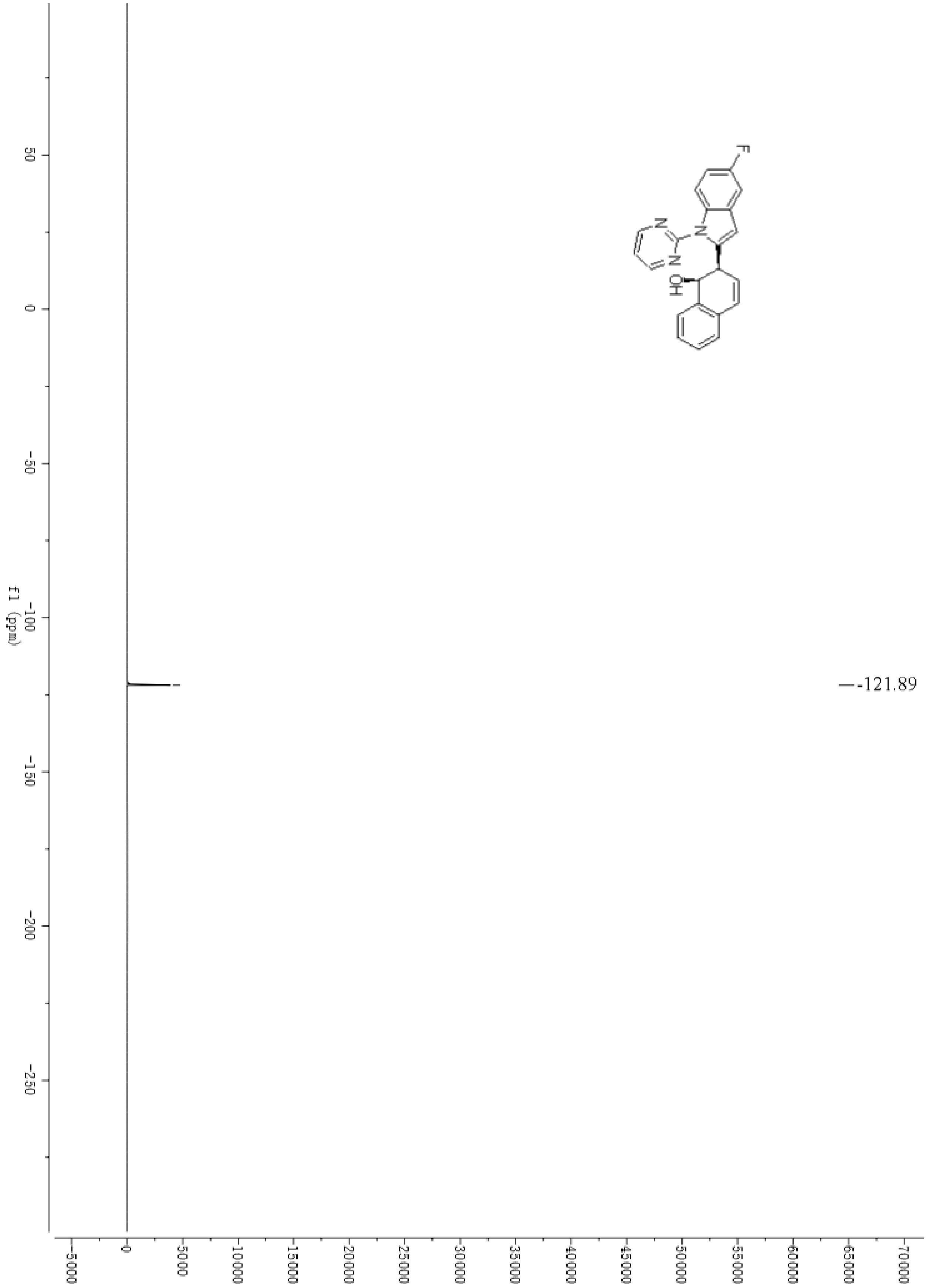

Supplementary Fig. 44 ^{1}H NMR Spectrum of *cis*-**3na**

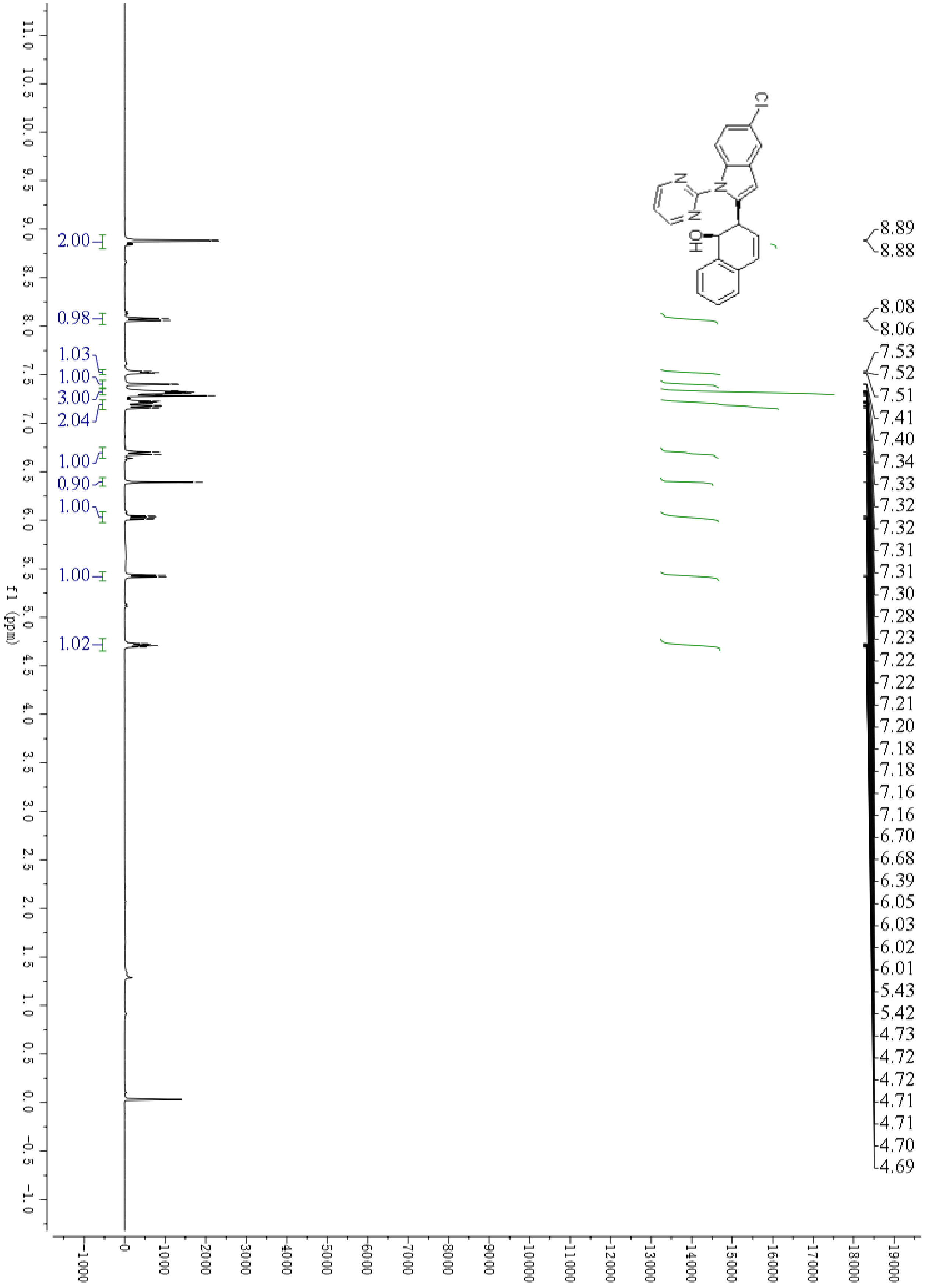

Supplementary Fig. 45 ^{13}C NMR Spectrum of *cis*-**3na**

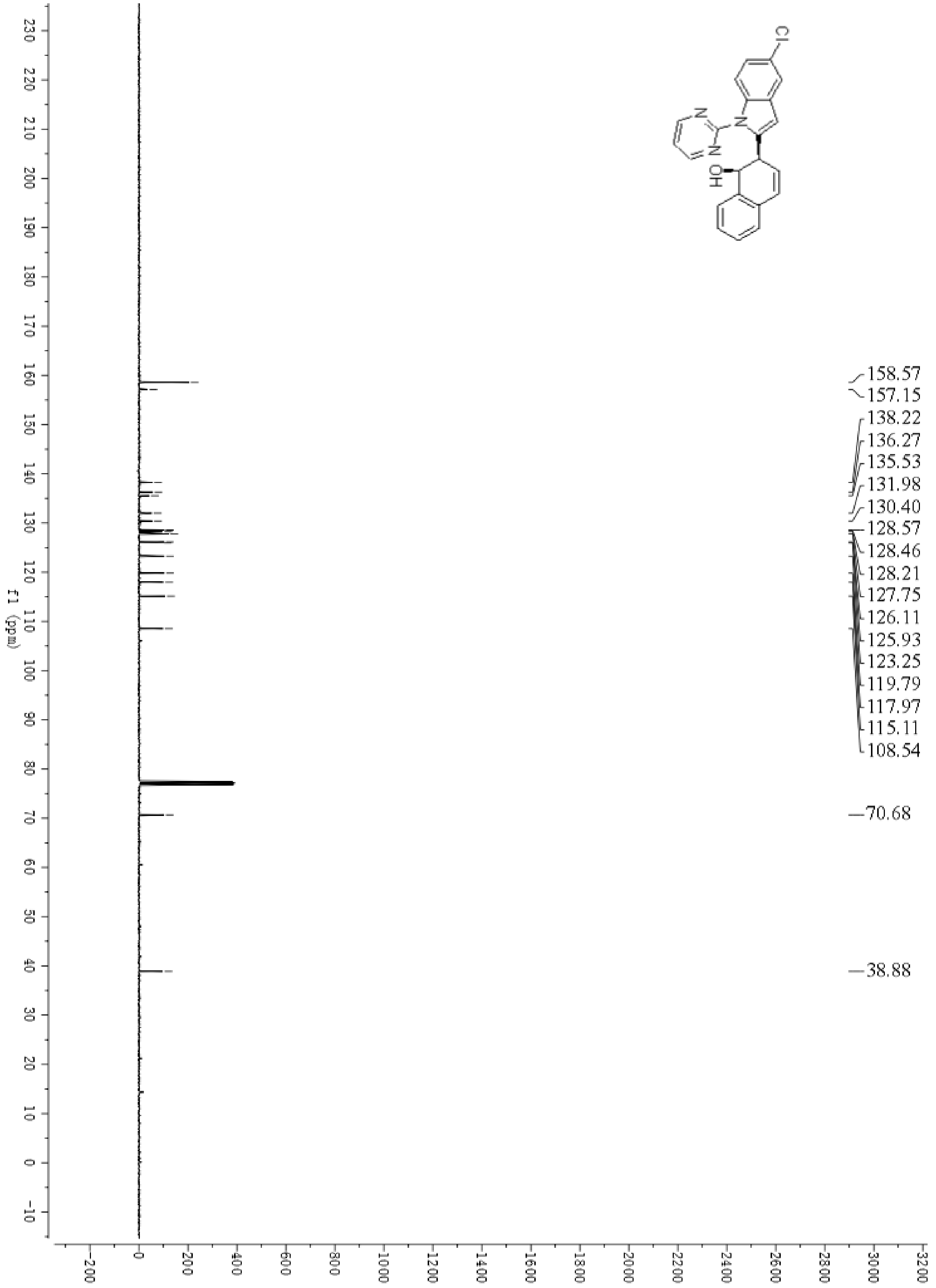

Supplementary Fig. 46 ^{1}H NMR Spectrum of *cis*-**3oa**

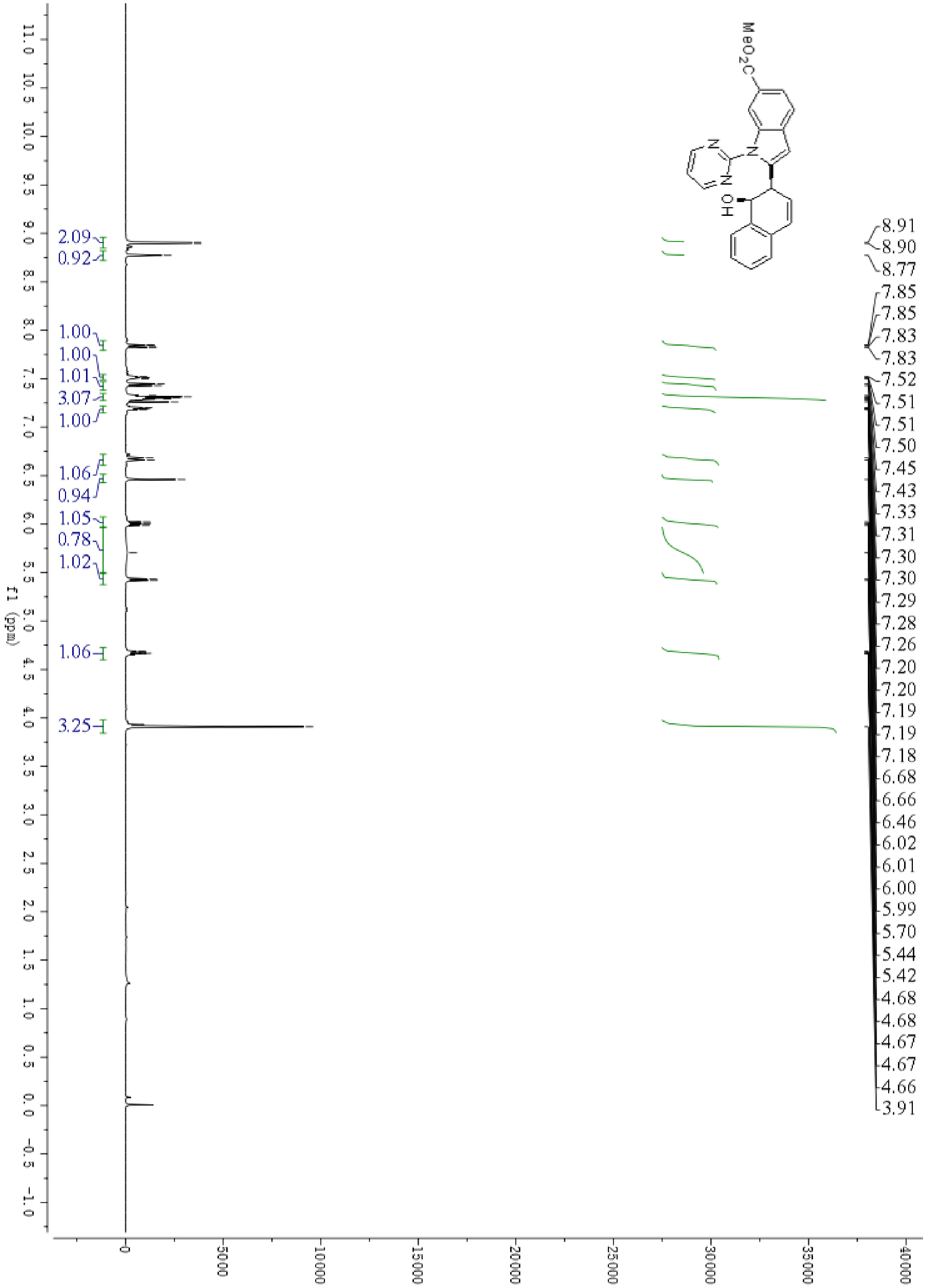

Supplementary Fig. 47 ^{13}C NMR Spectrum of *cis*-**3oa**

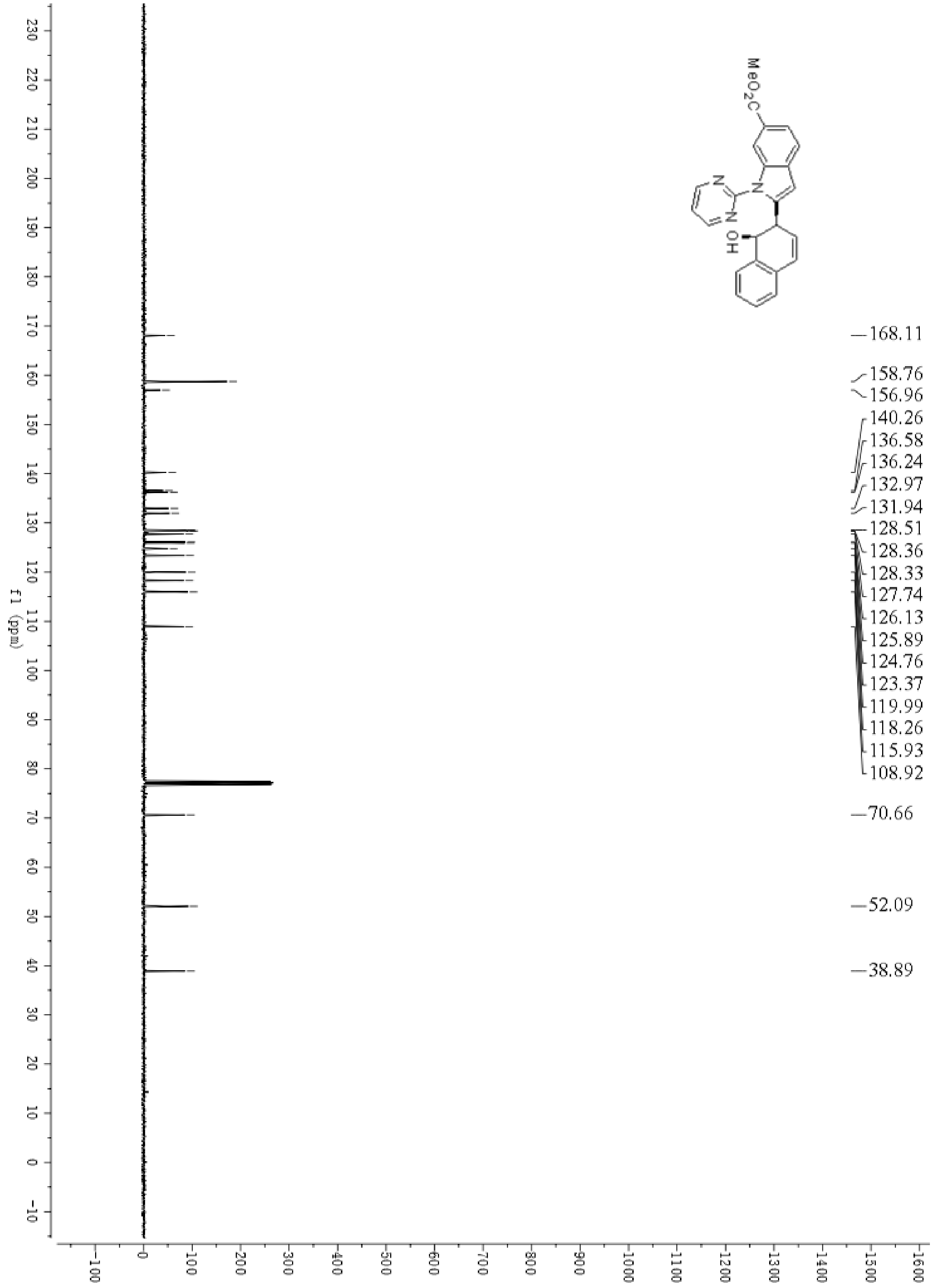

Supplementary Fig. 48 ^{1}H NMR Spectrum of *cis*-**3pa**

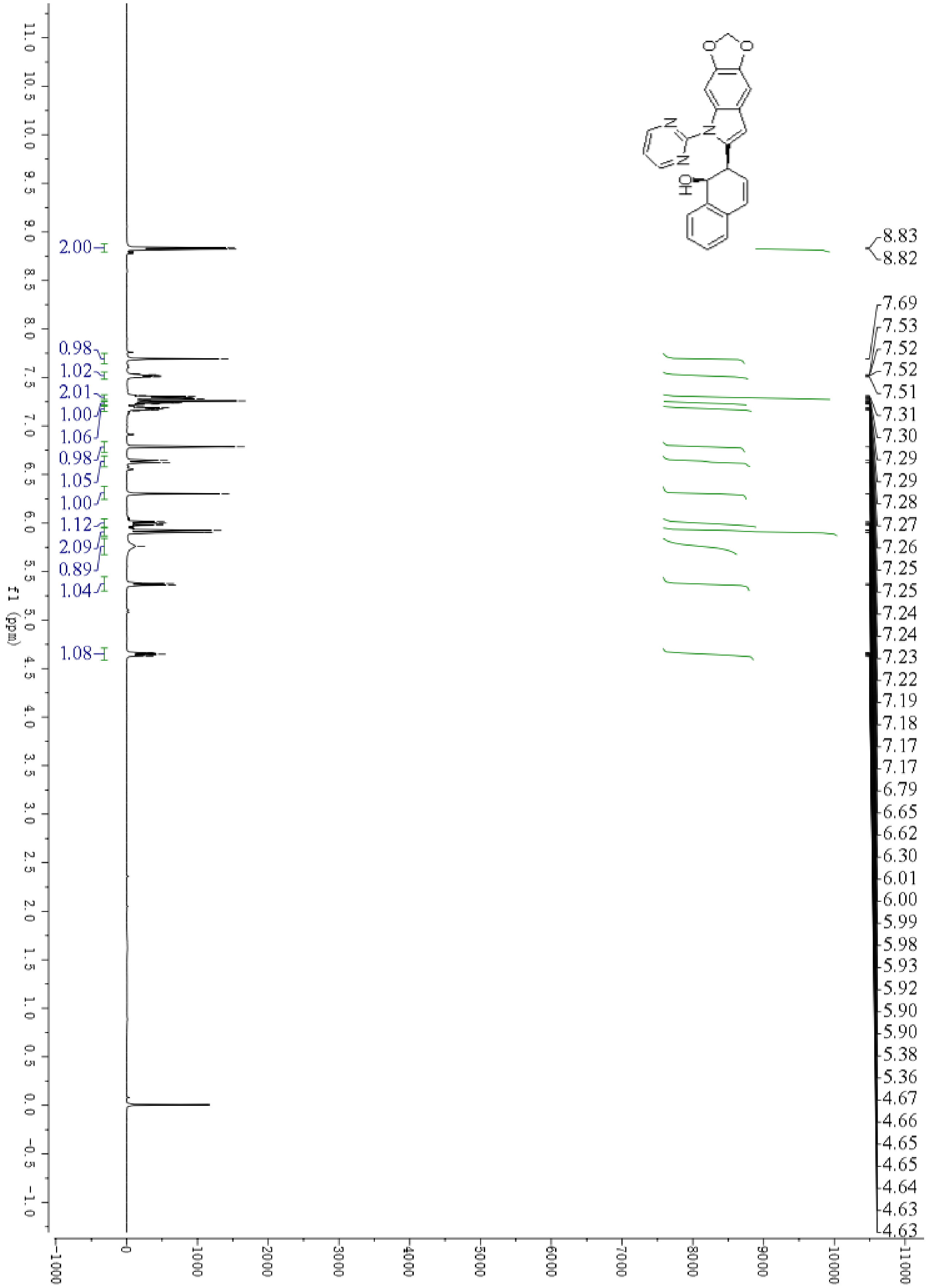

Supplementary Fig. 49 ^{13}C NMR Spectrum of *cis*-**3pa**

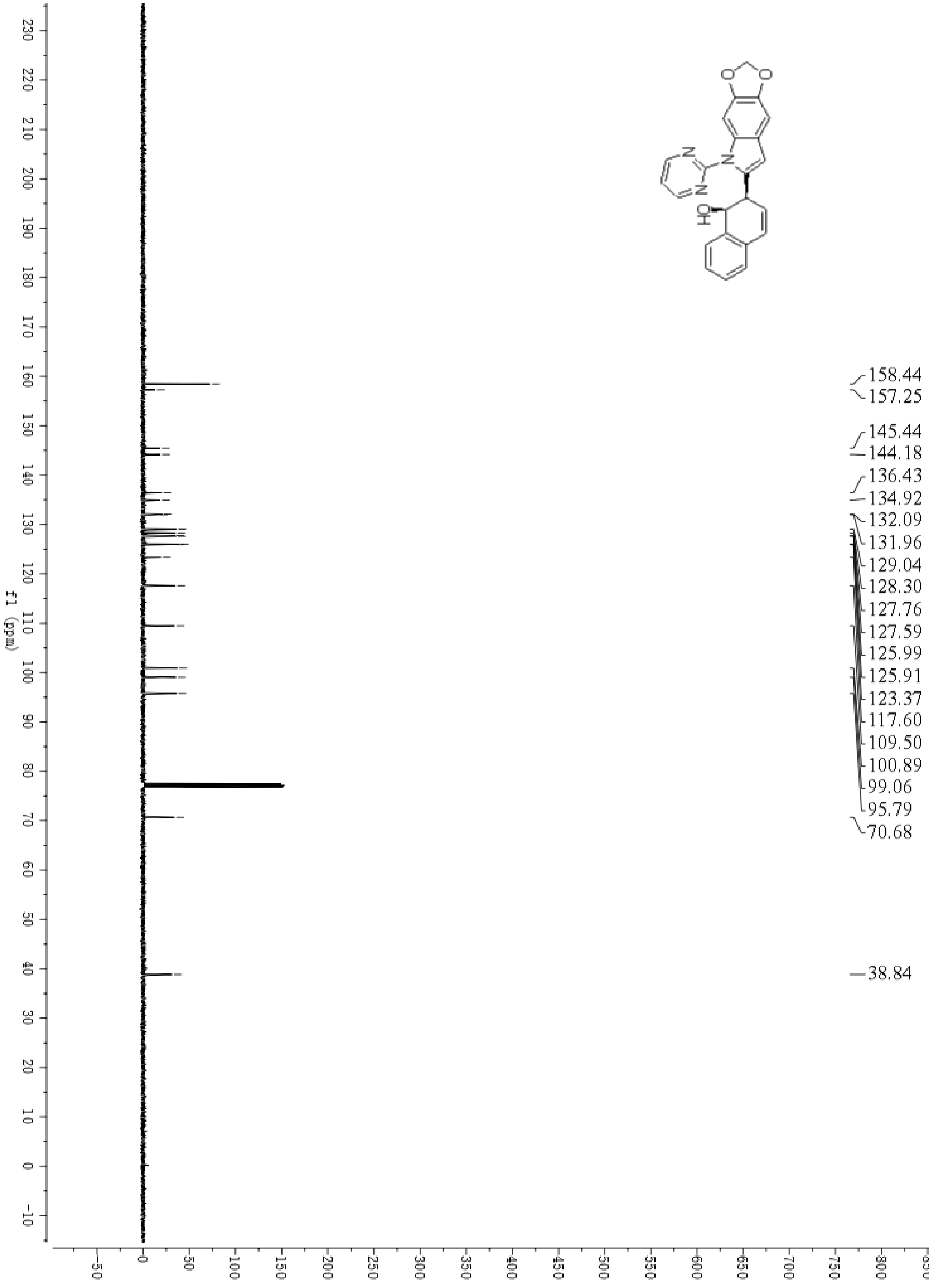

Supplementary Fig. 50 ^{1}H NMR Spectrum of *cis*-**3qa**

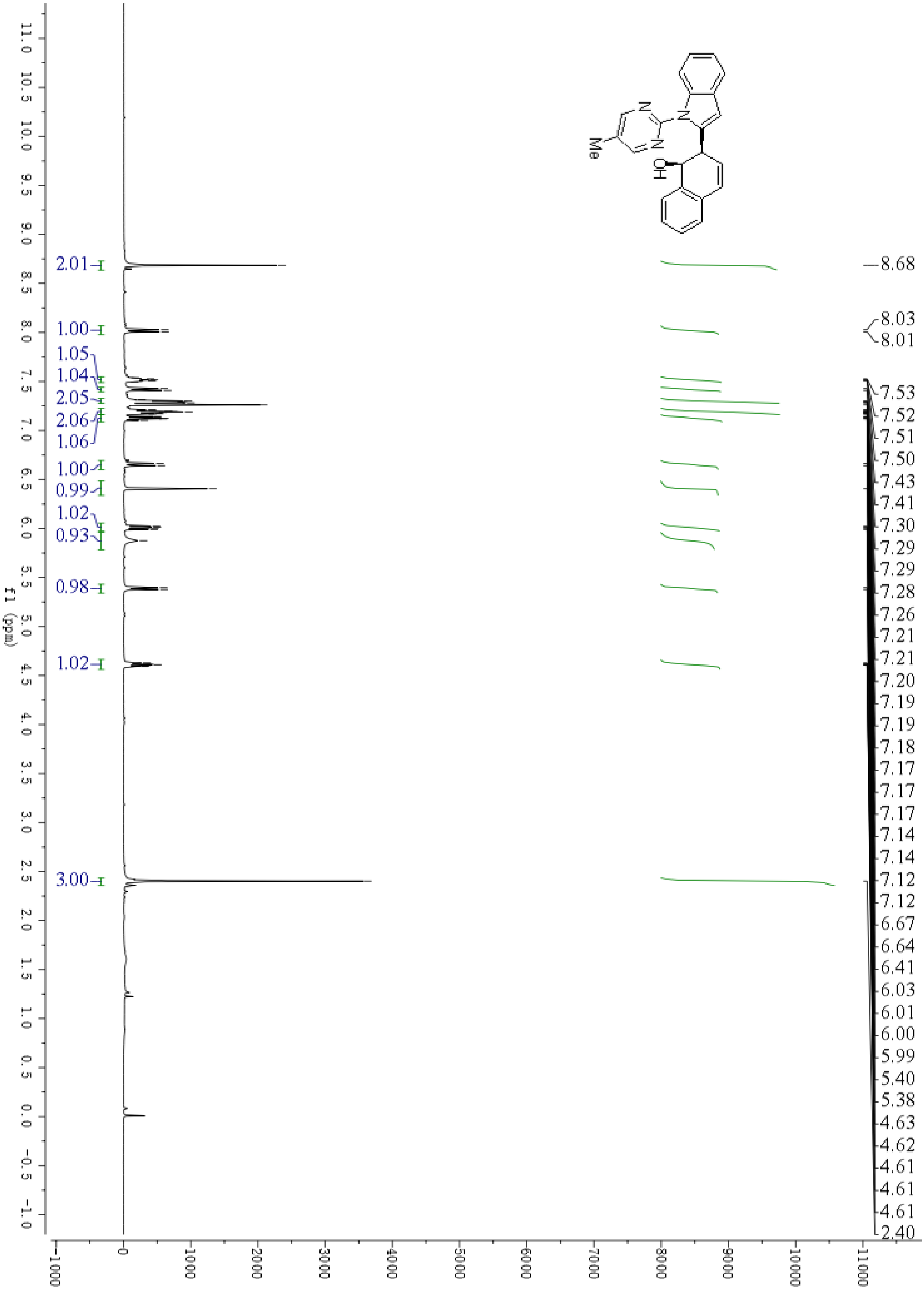

Supplementary Fig. 51 ^{13}C NMR Spectrum of *cis*-**3qa**

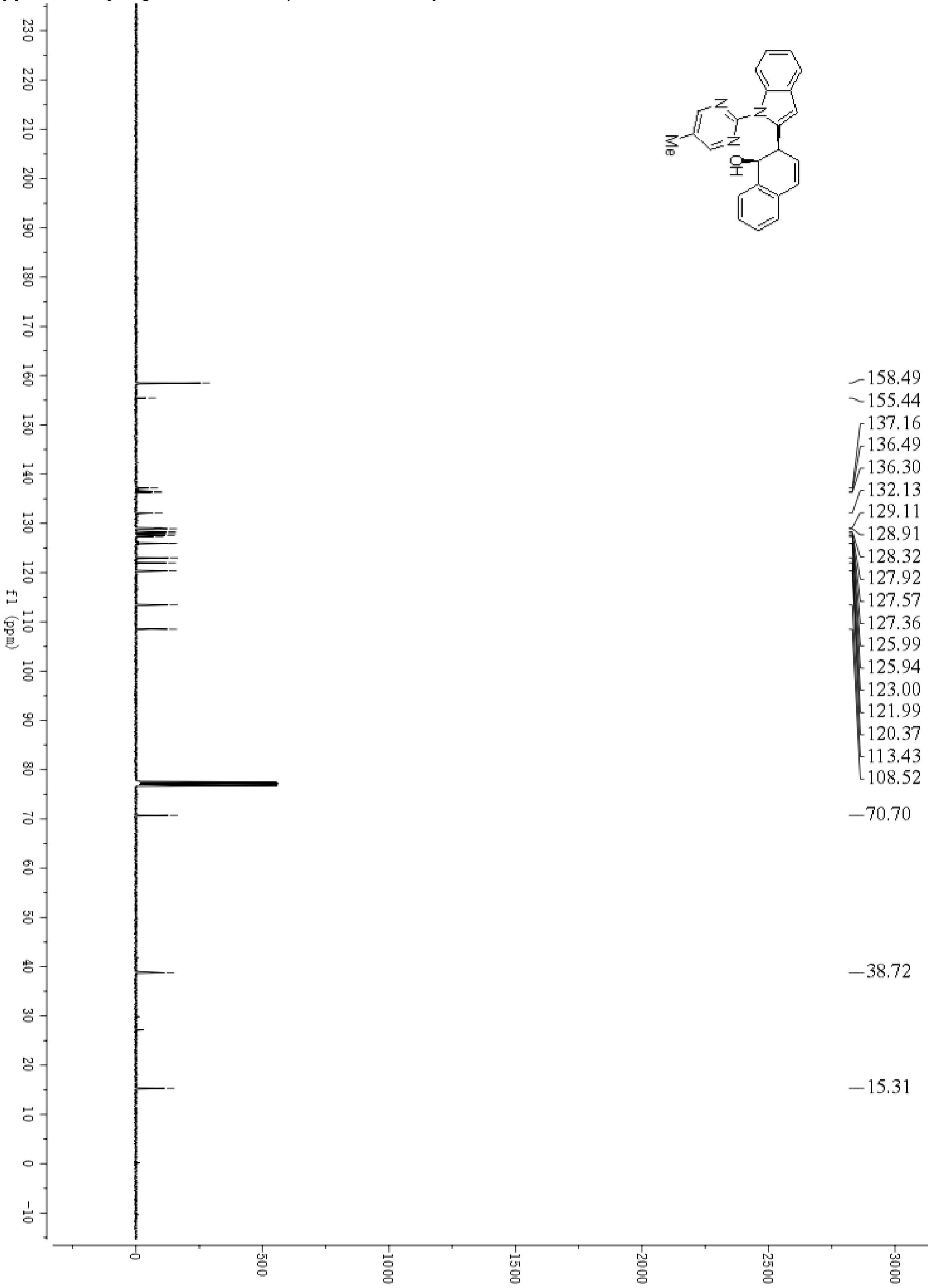

Supplementary Fig. 52 ^{1}H NMR Spectrum of *cis*-**3ra**

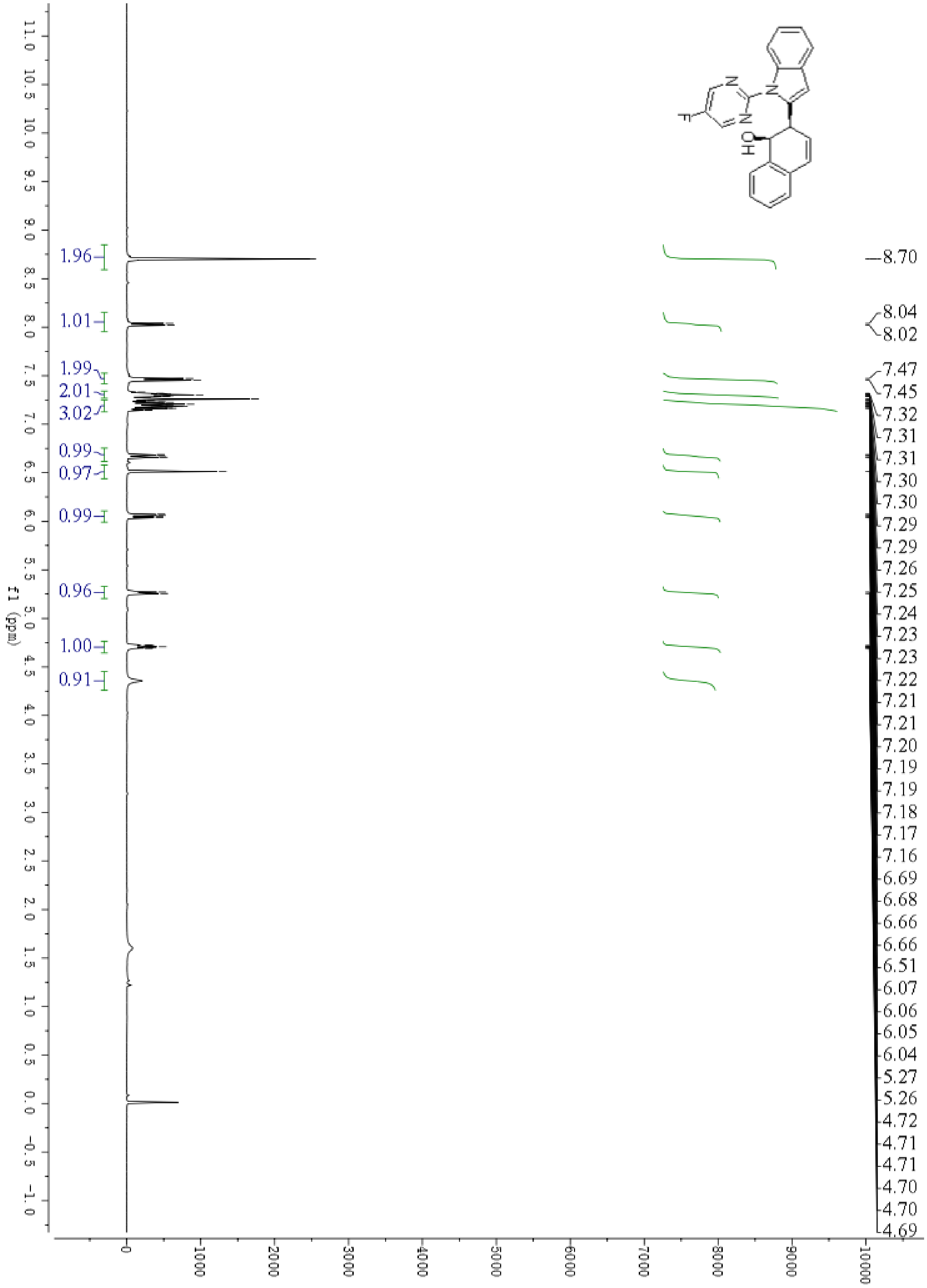

Supplementary Fig. 53 ^{13}C NMR Spectrum of *cis*-**3ra**

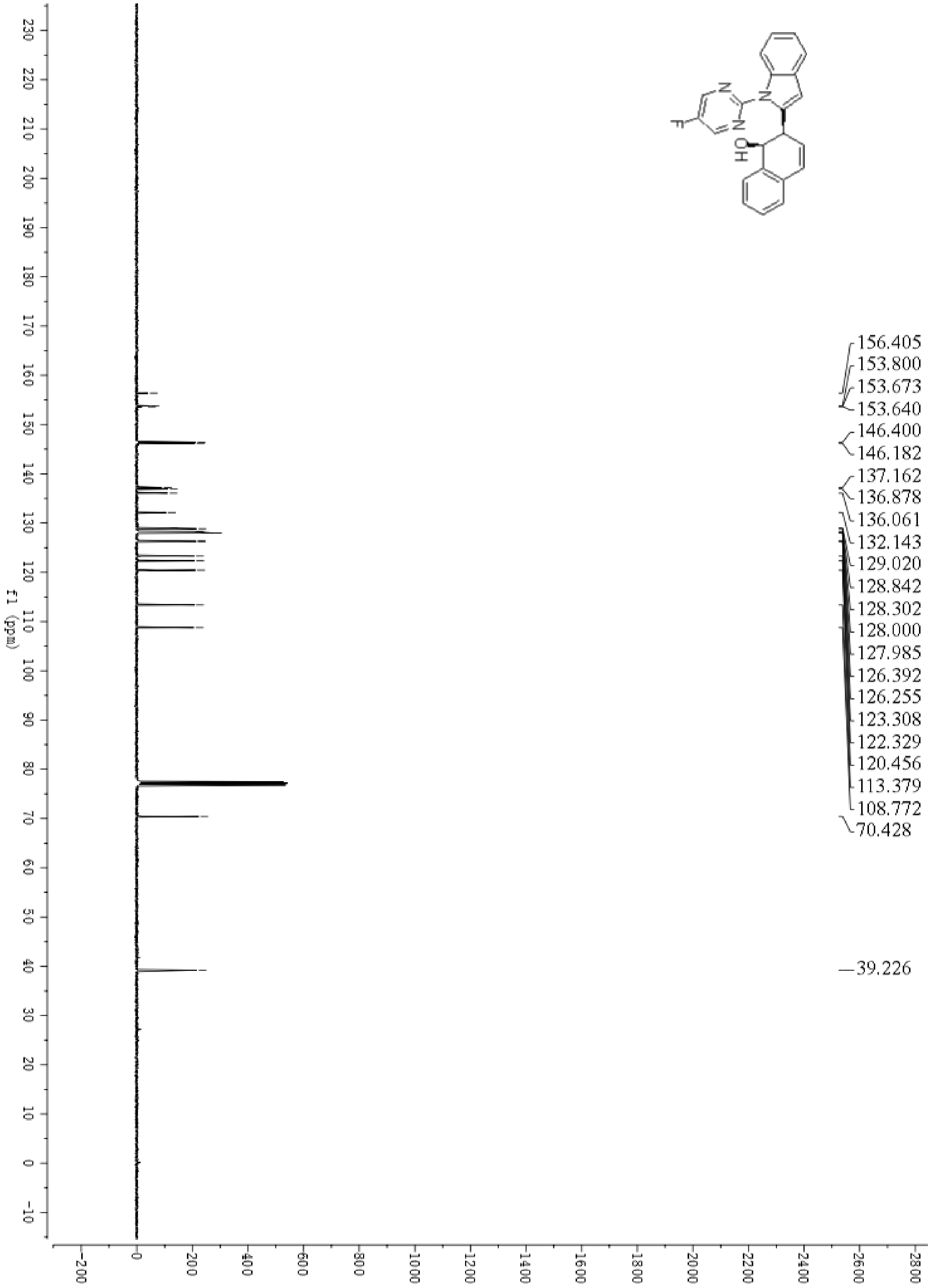

Supplementary Fig. 54 ^{19}F NMR Spectrum of *cis*-**3ra**

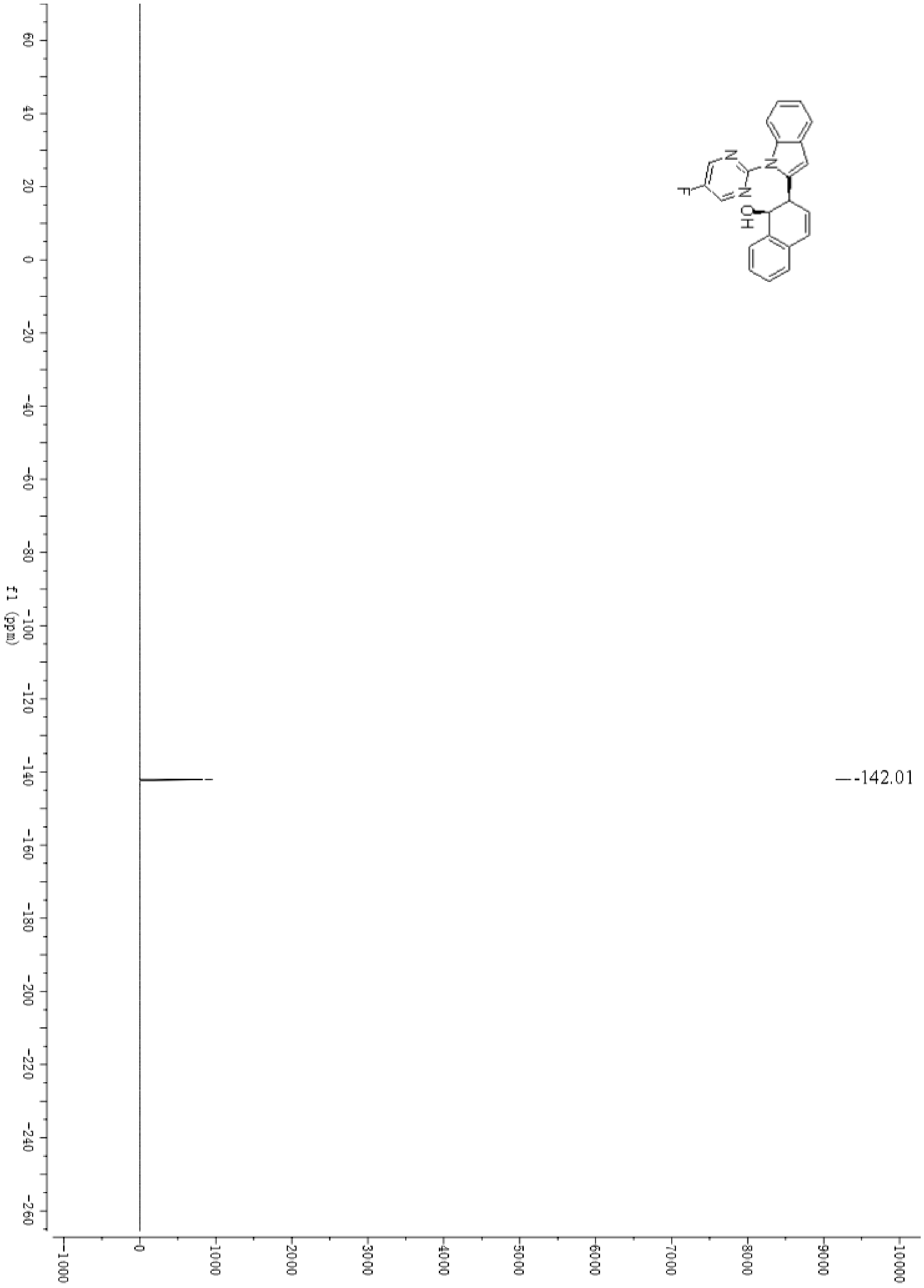

Supplementary Fig. 55 ^{1}H NMR Spectrum of *cis*-**3sa**

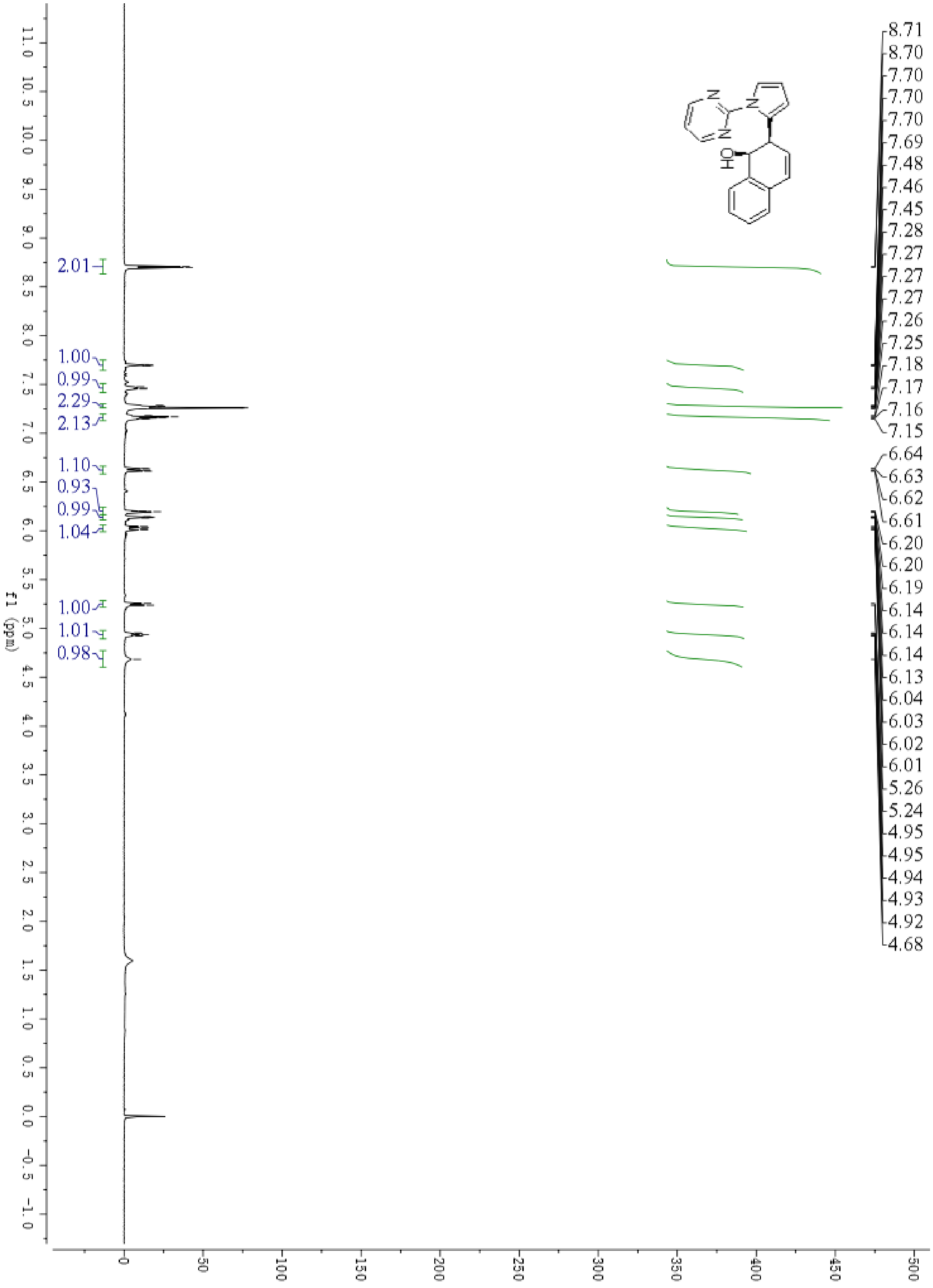

Supplementary Fig. 56 ^{13}C NMR Spectrum of *cis*-**3sa**

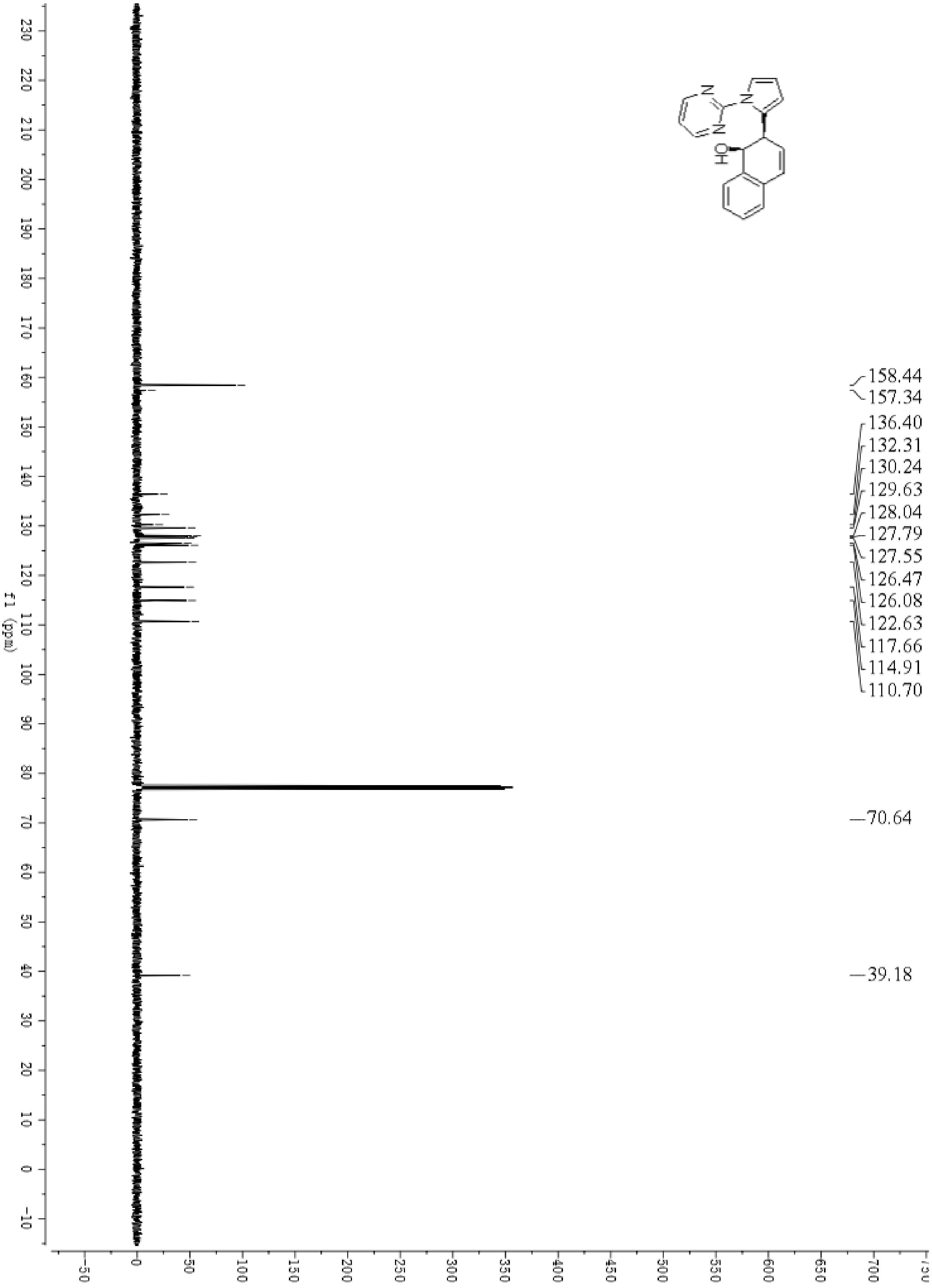

Supplementary Fig. 57 ^{1}H NMR Spectrum of *cis*-**3ta**

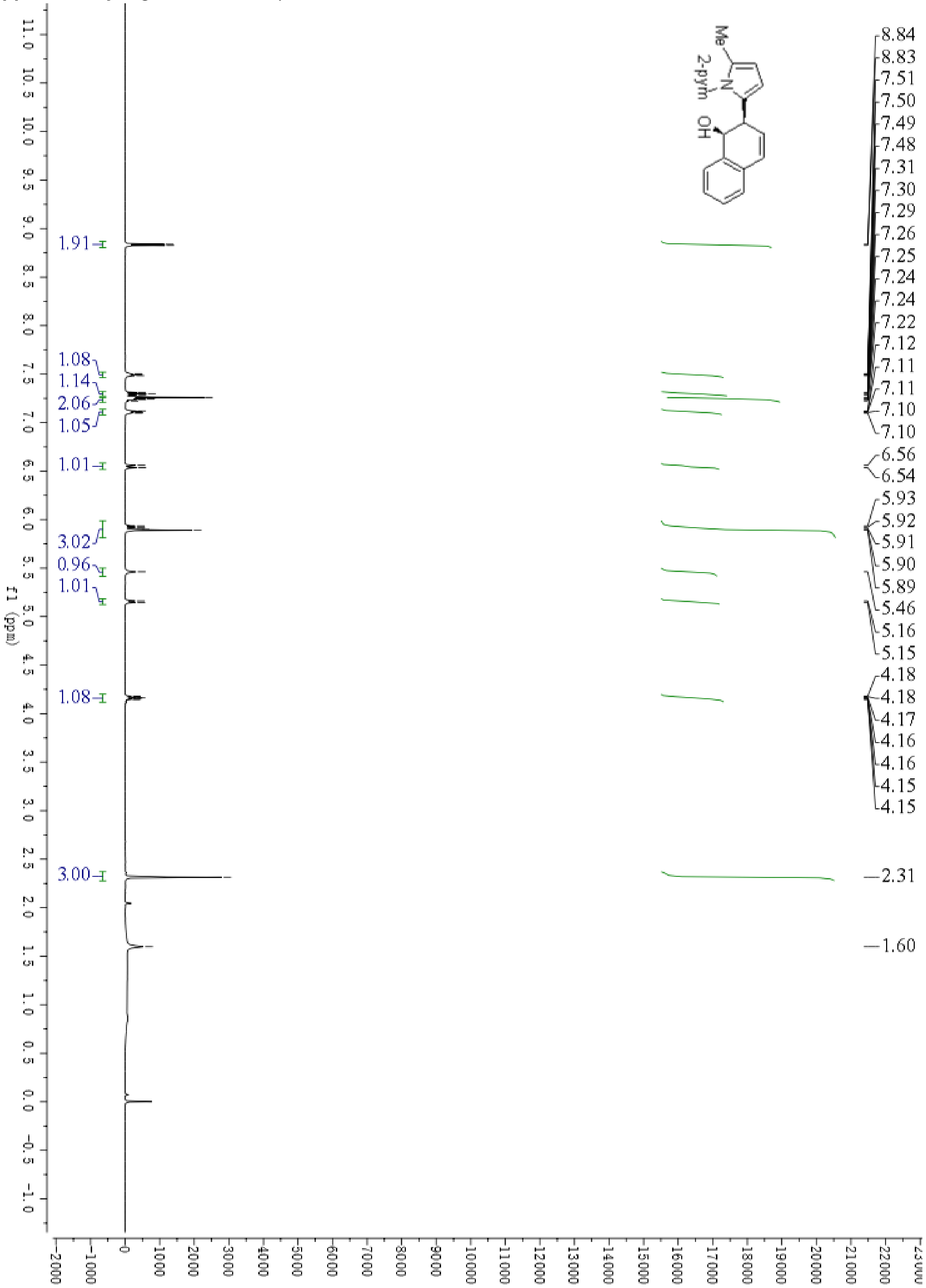

Supplementary Fig. 58 ^{13}C NMR Spectrum of *cis*-**3ta**

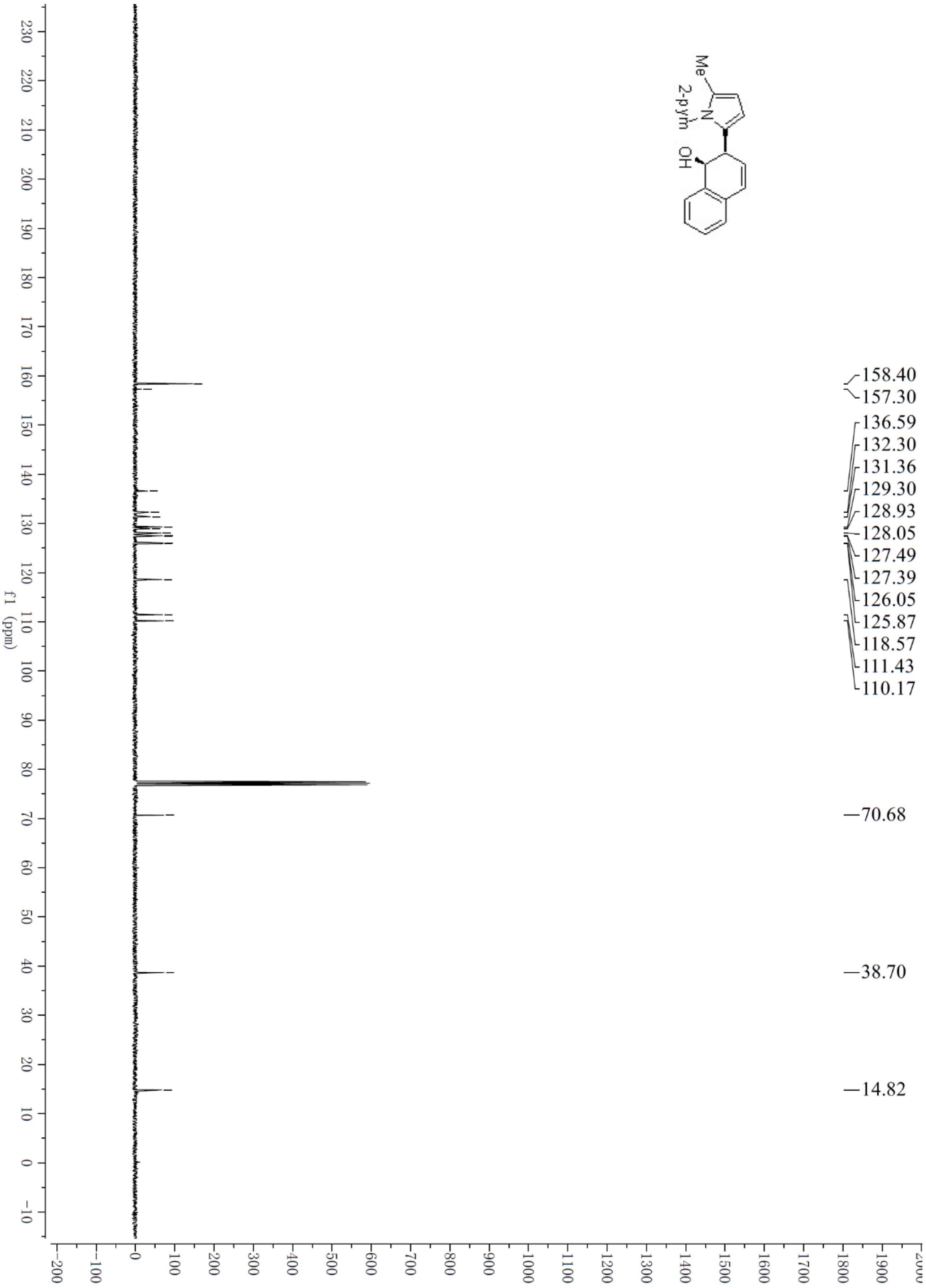

Supplementary Fig. 59 ^{1}H NMR Spectrum of *cis*-**3ua**

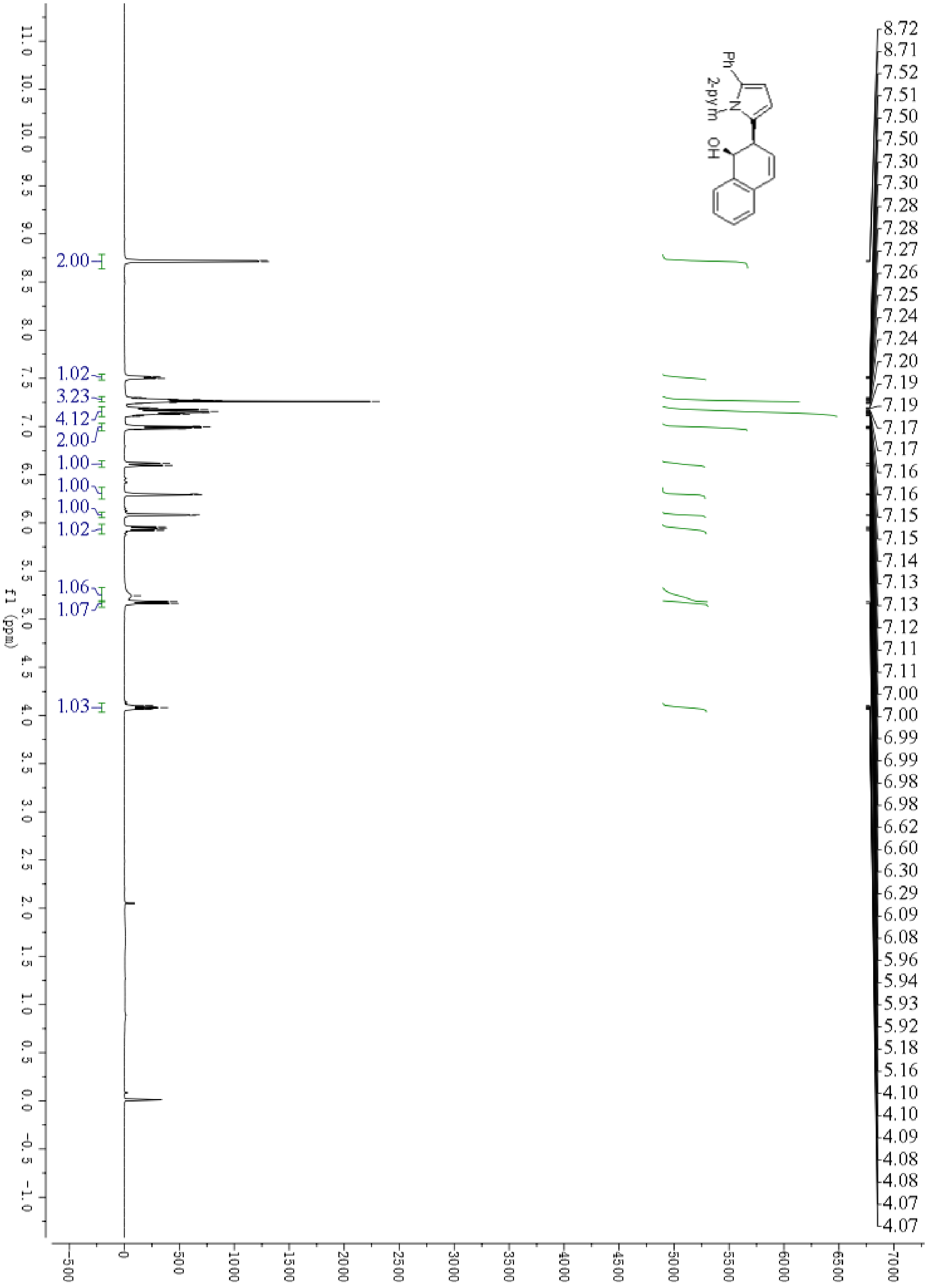

Supplementary Fig. 60 ^{13}C NMR Spectrum of *cis*-**3ua**

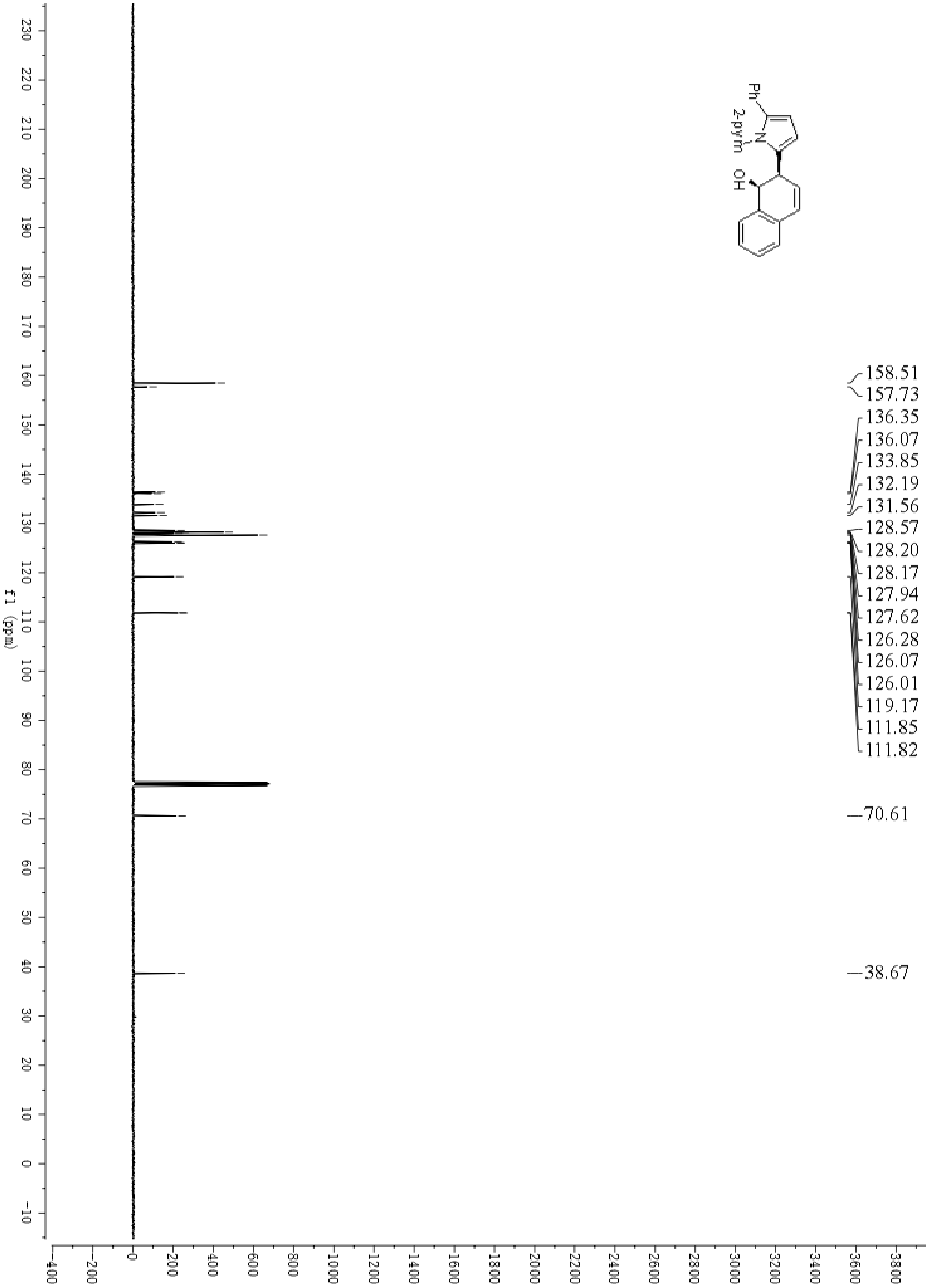

Supplementary Fig. 61 ^{1}H NMR Spectrum of *cis*-**3va**

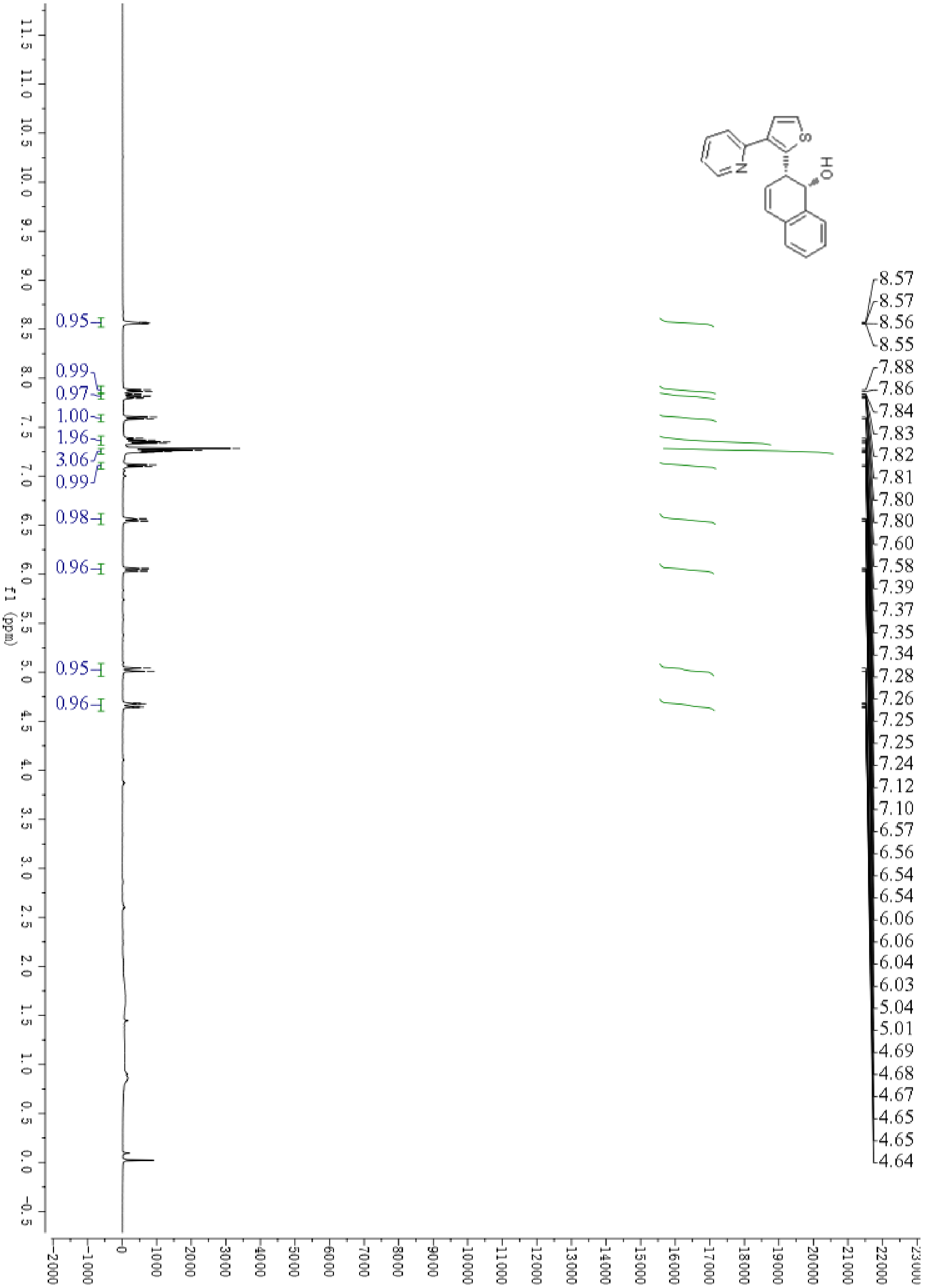

Supplementary Fig. 62 ^{13}C NMR Spectrum of *cis*-**3va**

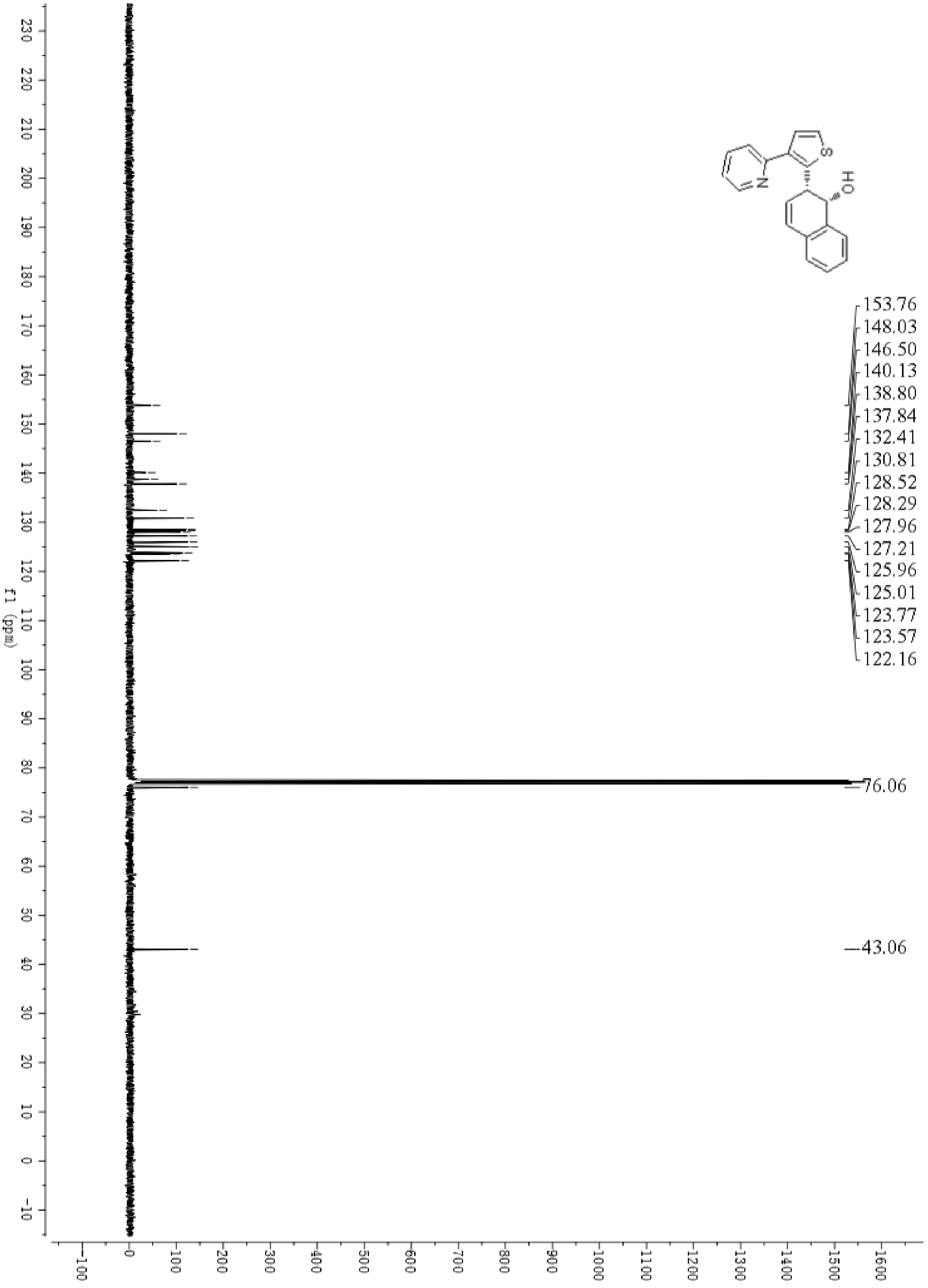

Supplementary Fig. 63 ^{1}H NMR Spectrum of *cis*-**3wa**

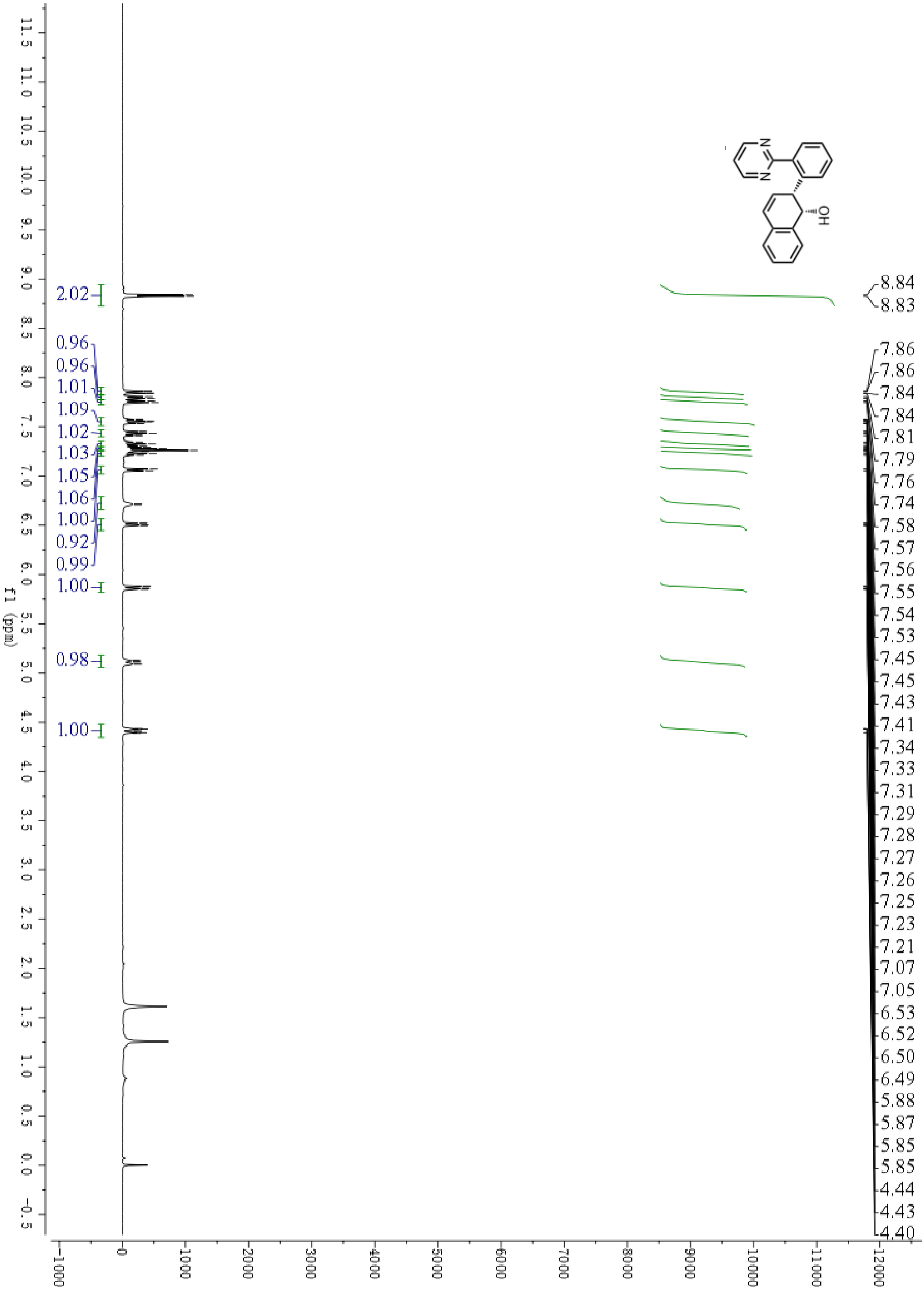

Supplementary Fig. 64 ^{13}C NMR Spectrum of *cis*-**3wa**

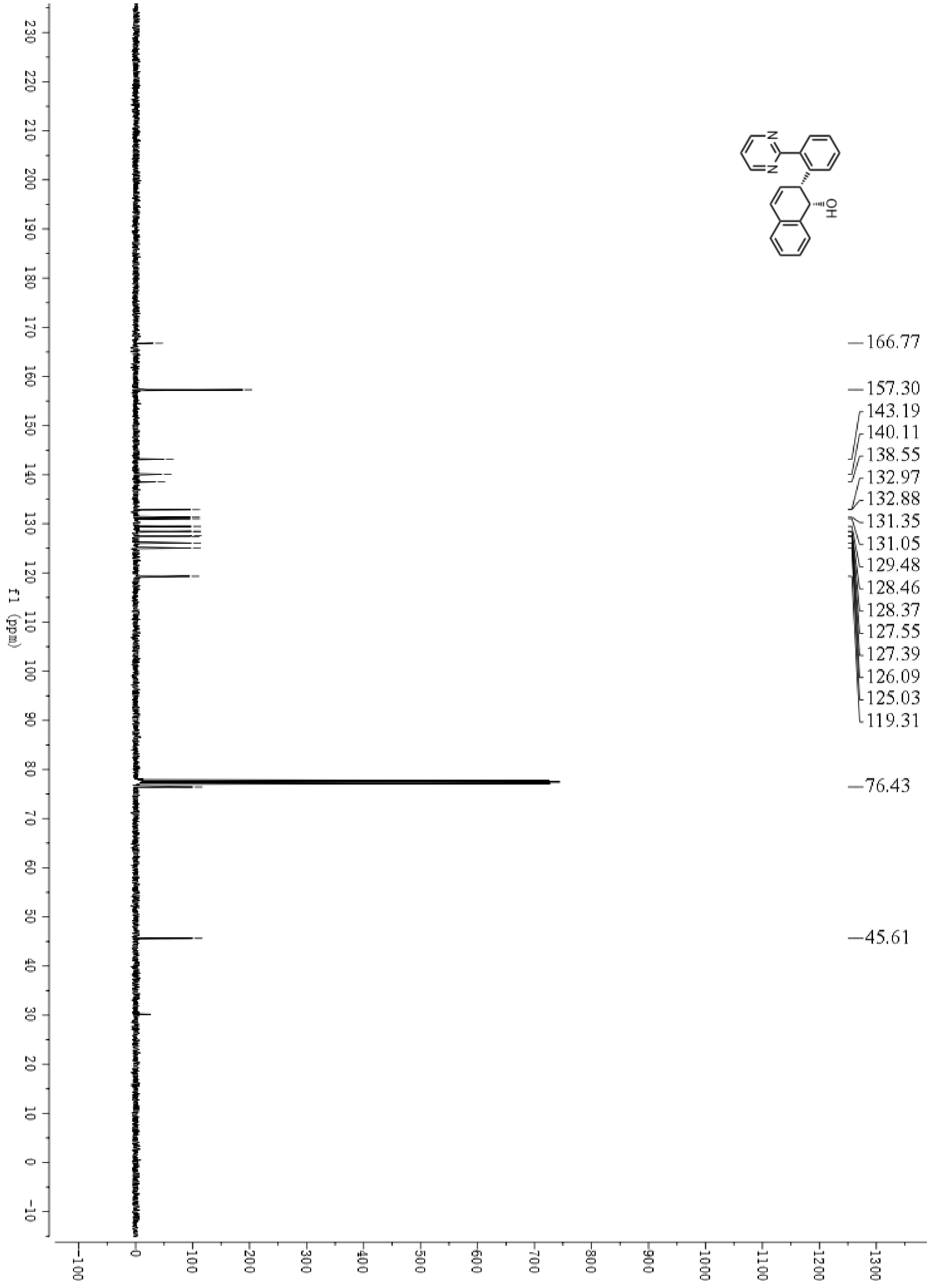

Supplementary Fig. 65 ^{1}H NMR Spectrum of *cis*-**3ab**

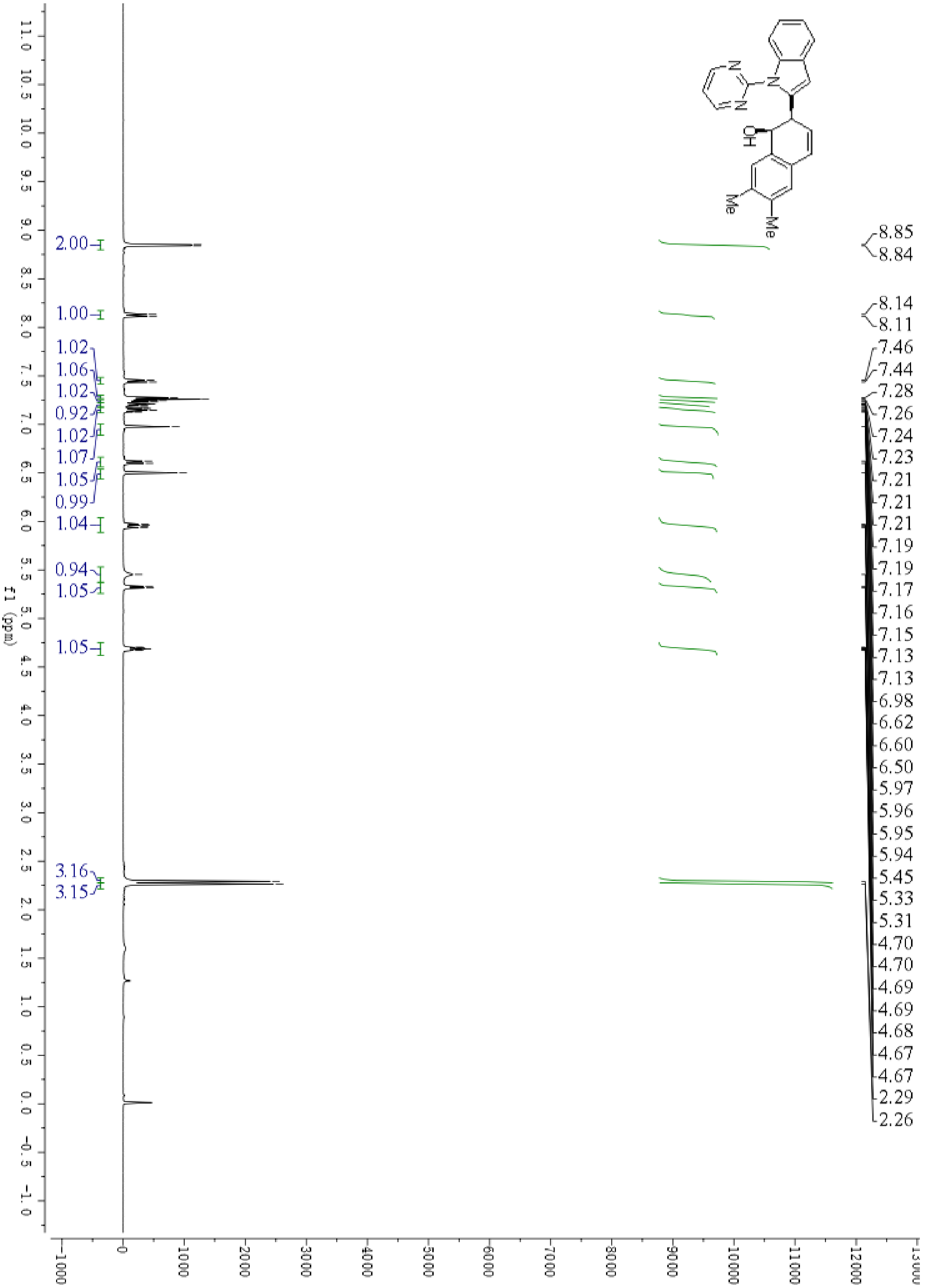

Supplementary Fig. 66 ^{13}C NMR Spectrum of *cis*-**3ab**

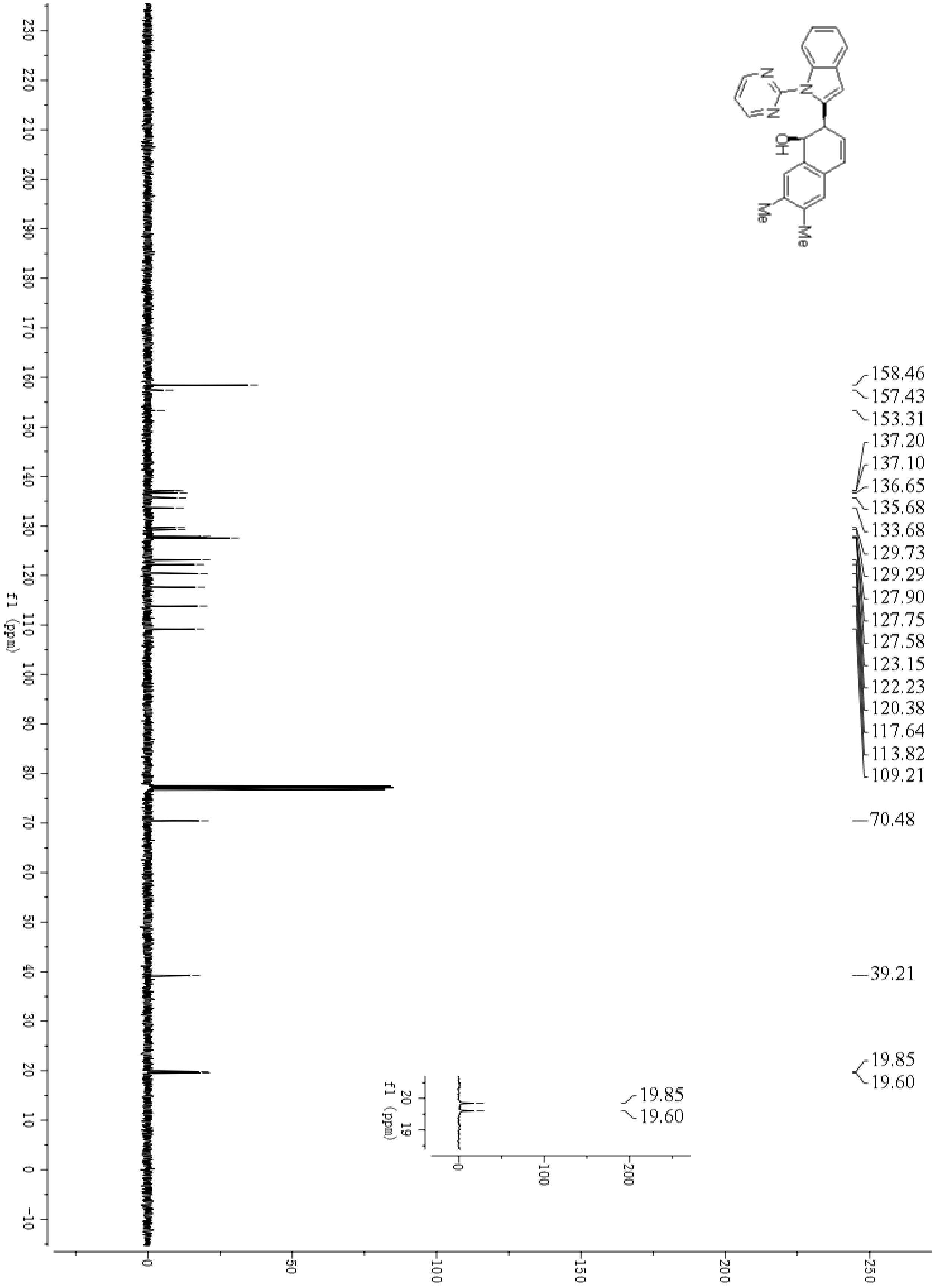

Supplementary Fig. 67 ^{1}H NMR Spectrum of *cis*-**3ac**

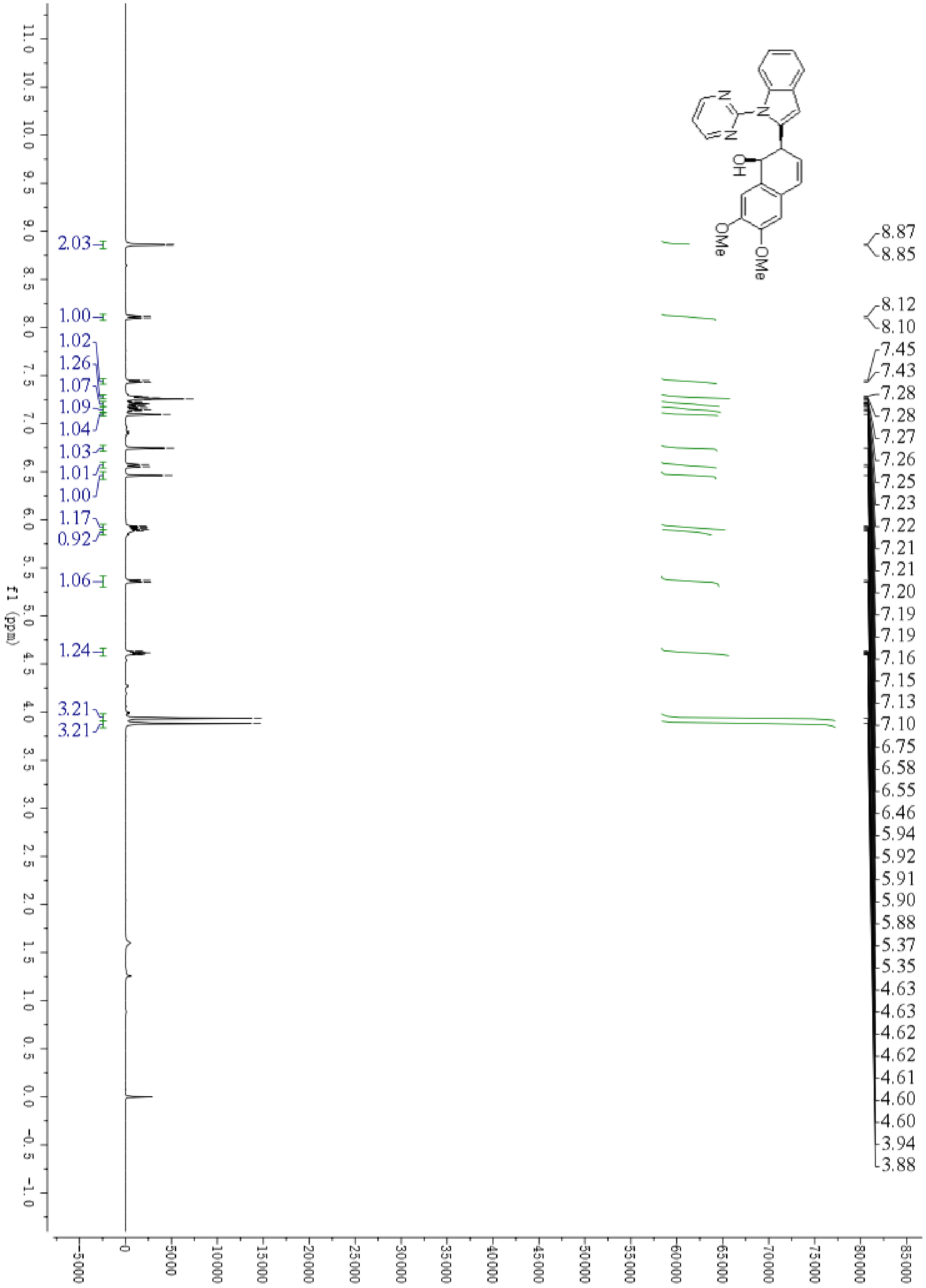

Supplementary Fig. 68 ^{13}C NMR Spectrum of *cis*-**3ac**

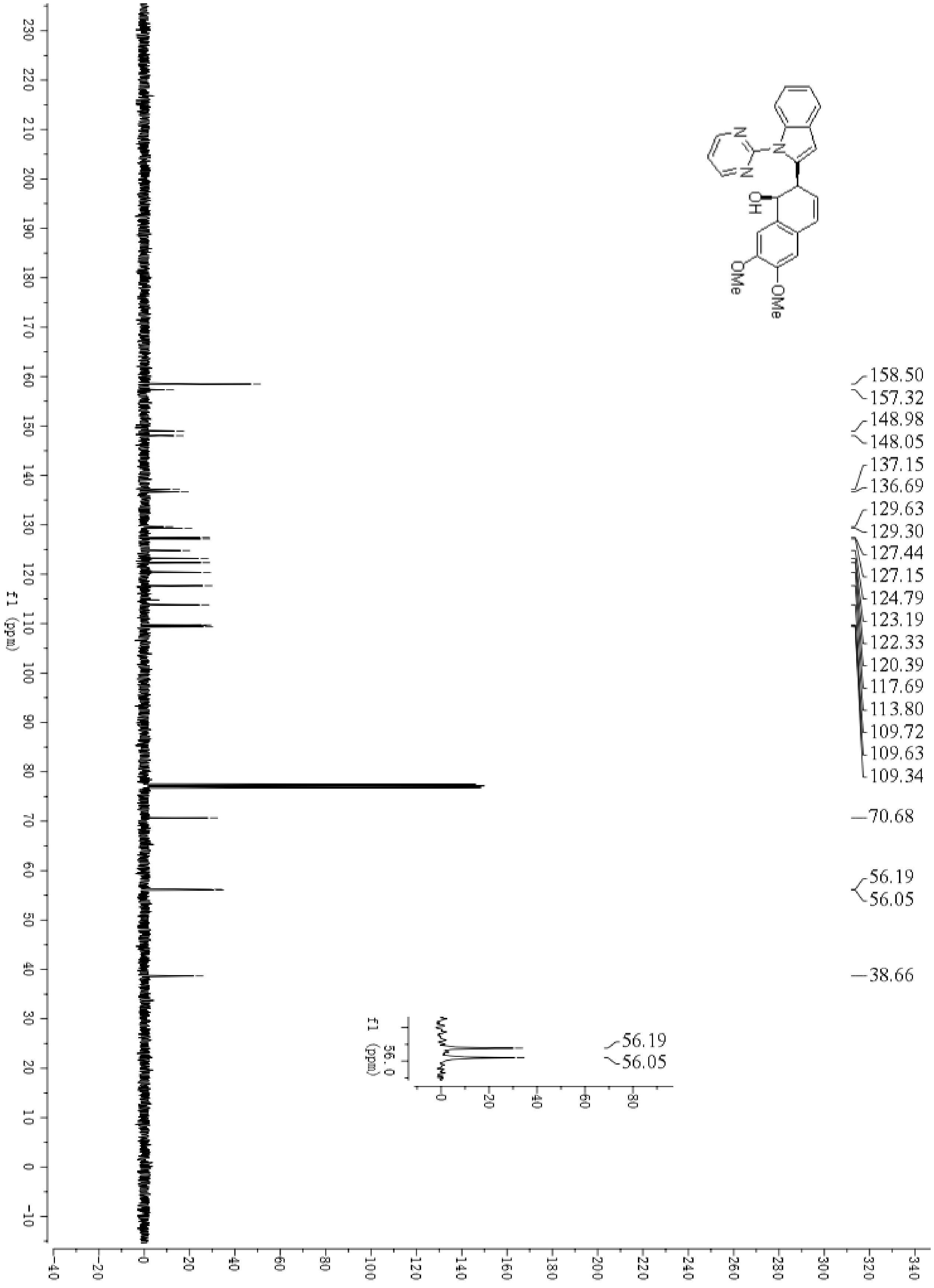

Supplementary Fig. 69 ^{1}H NMR Spectrum of *cis*-**3ad**

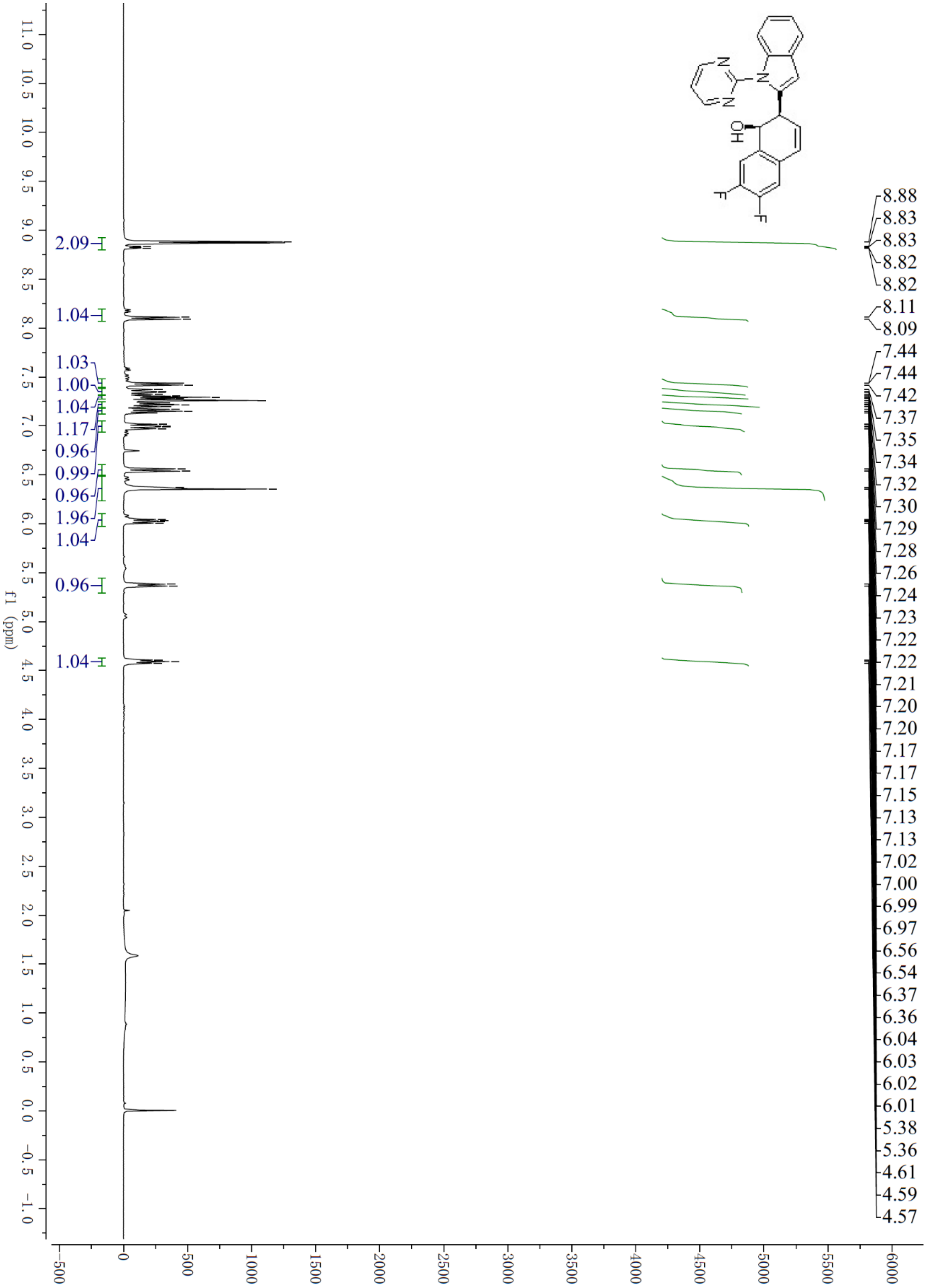

Supplementary Fig. 70 ^{13}C NMR Spectrum of *cis*-**3ad**

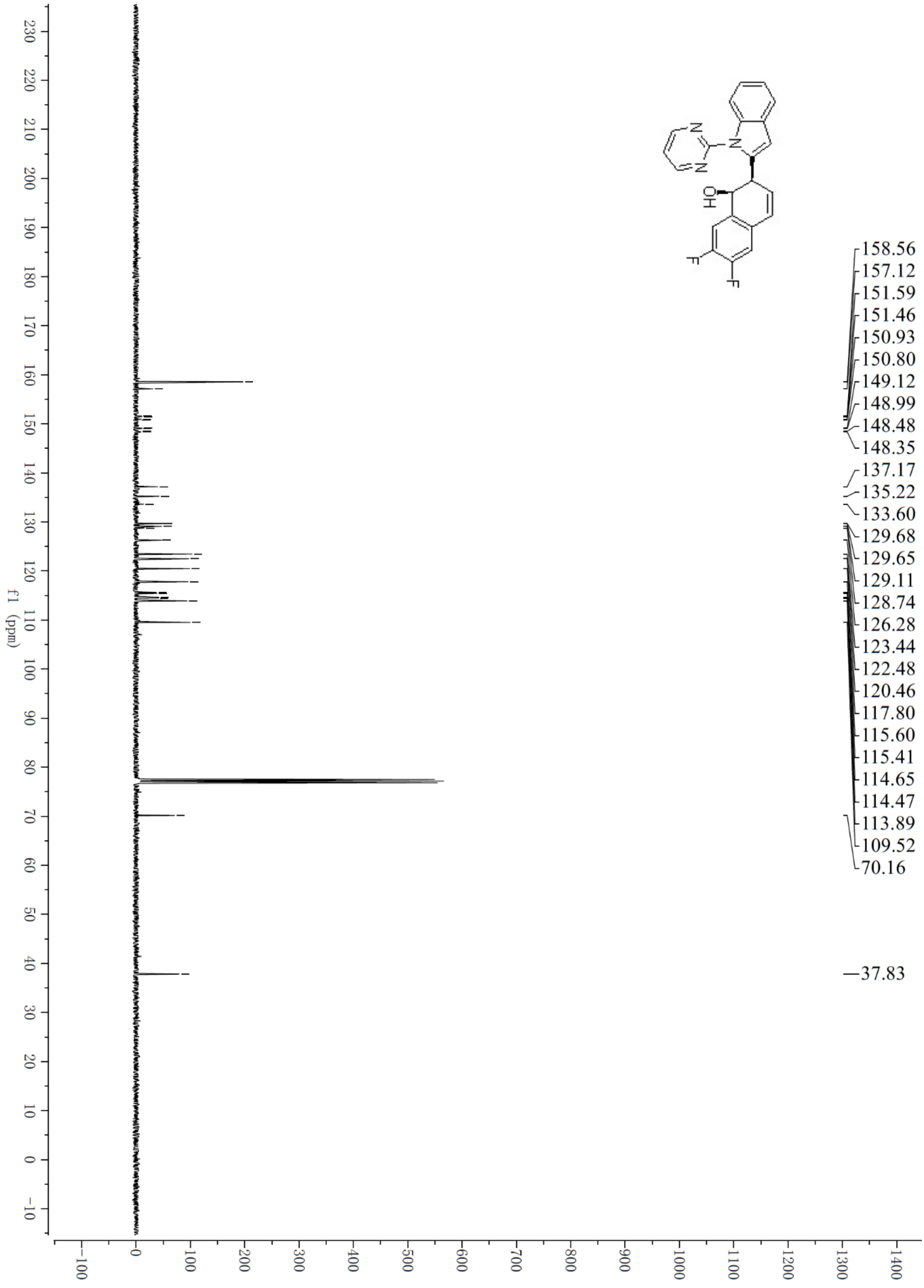

Supplementary Fig. 71 ^{19}F NMR Spectrum of *cis*-**3ad**

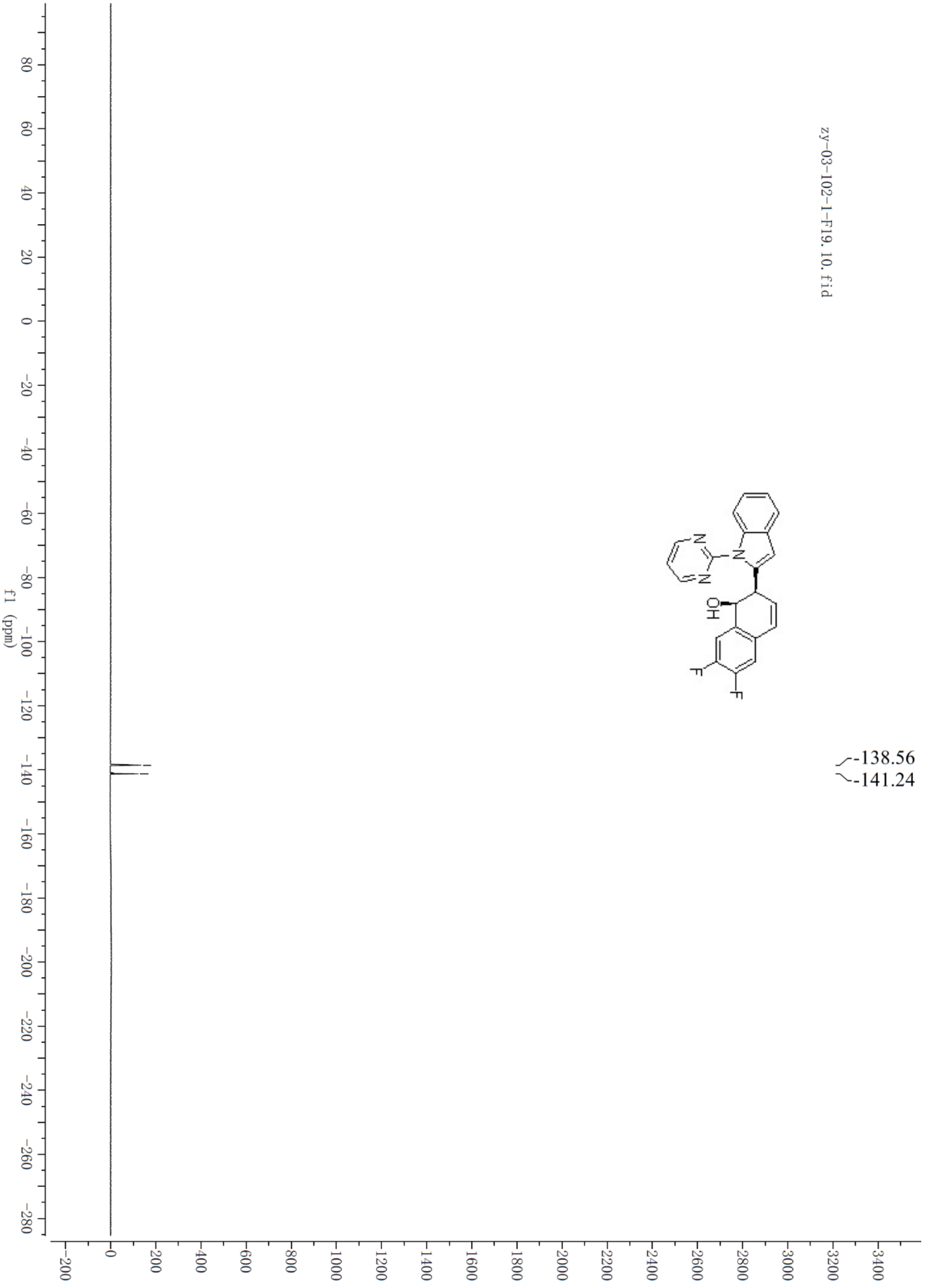

Supplementary Fig. 72 ^{1}H NMR Spectrum of *cis*-**3ae**

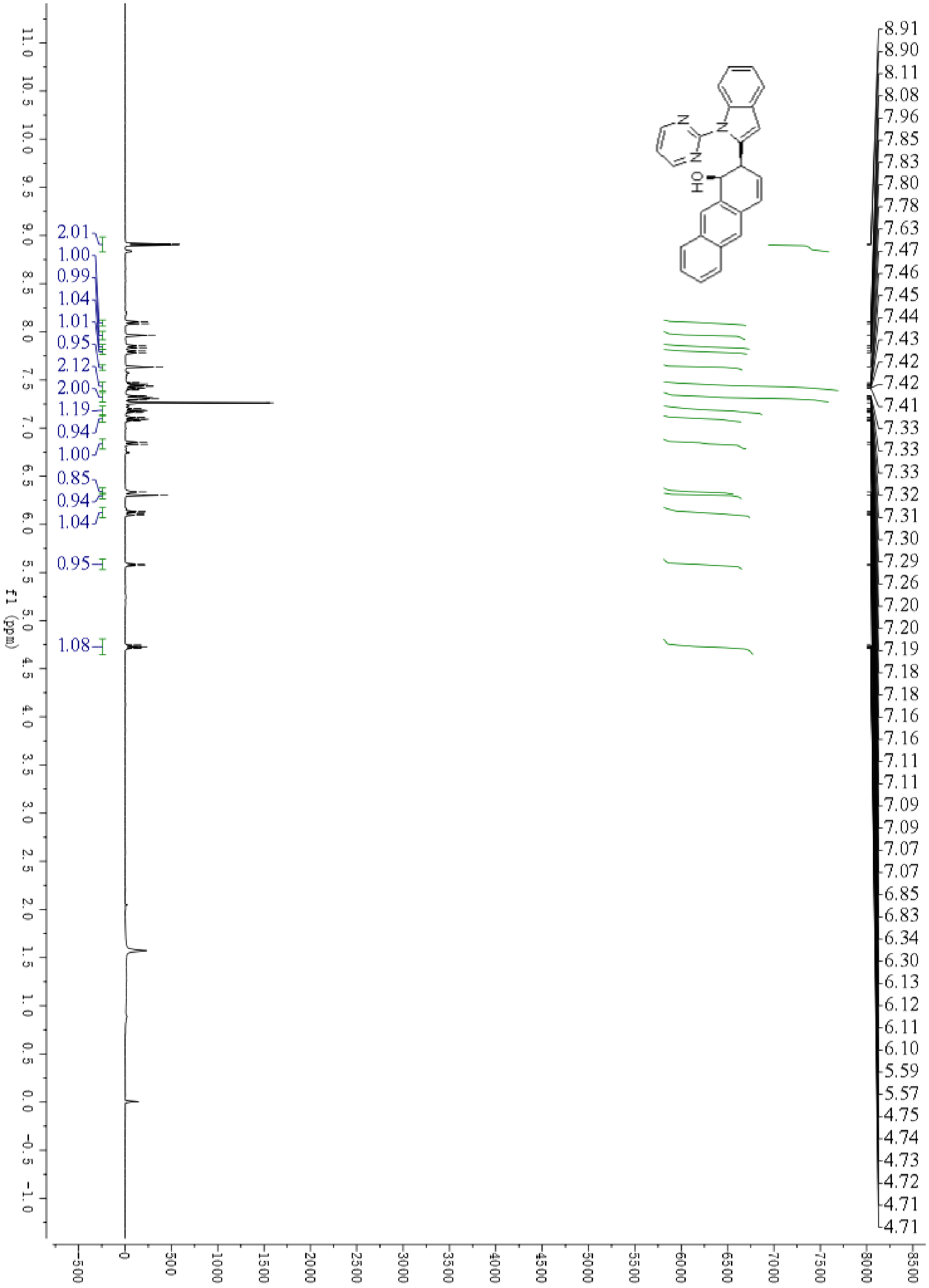

Supplementary Fig. 73 ^{13}C NMR Spectrum of *cis*-**3ae**

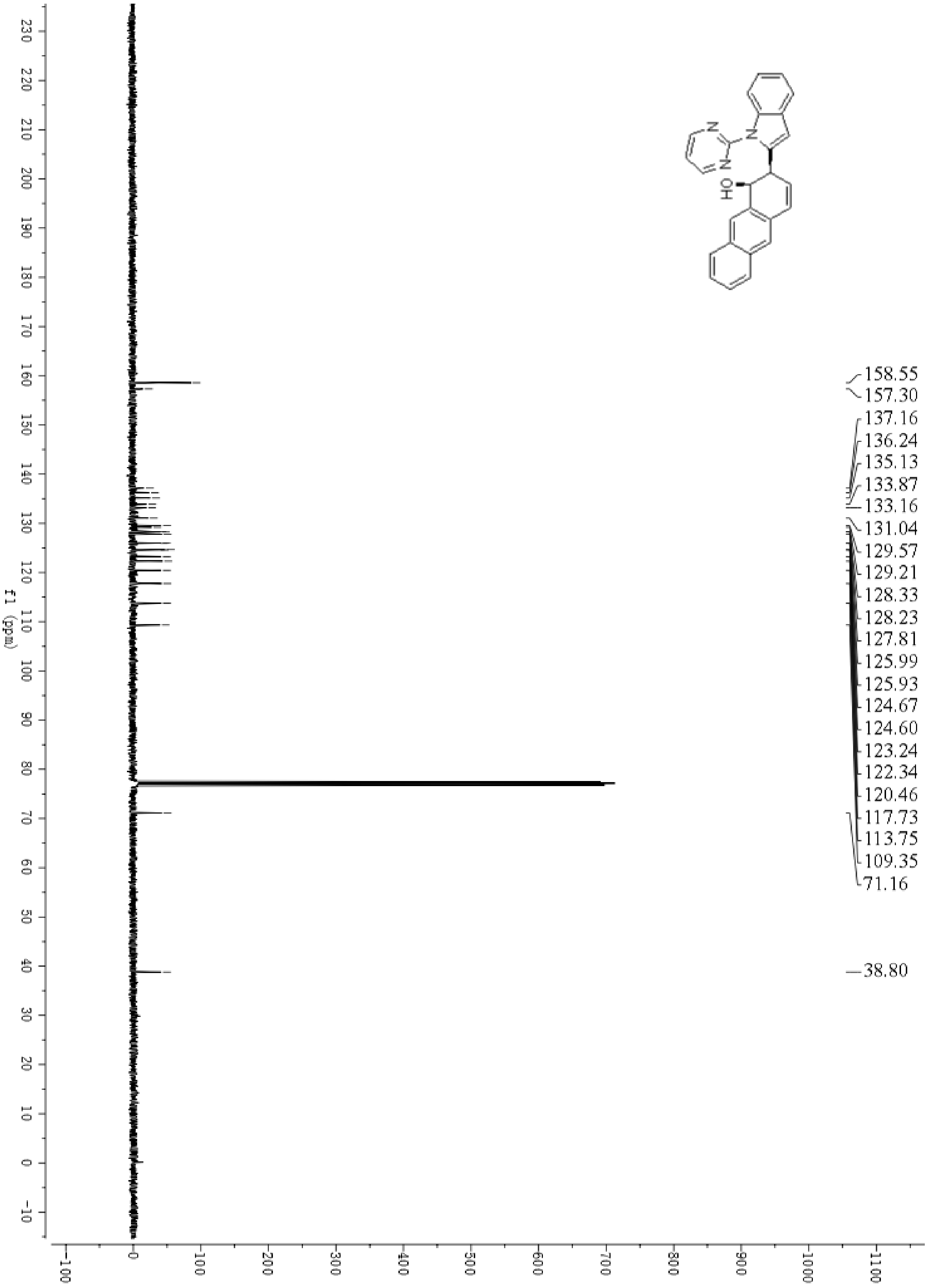

Supplementary Fig. 74 ¹H NMR Spectrum of *cis*-**3af**

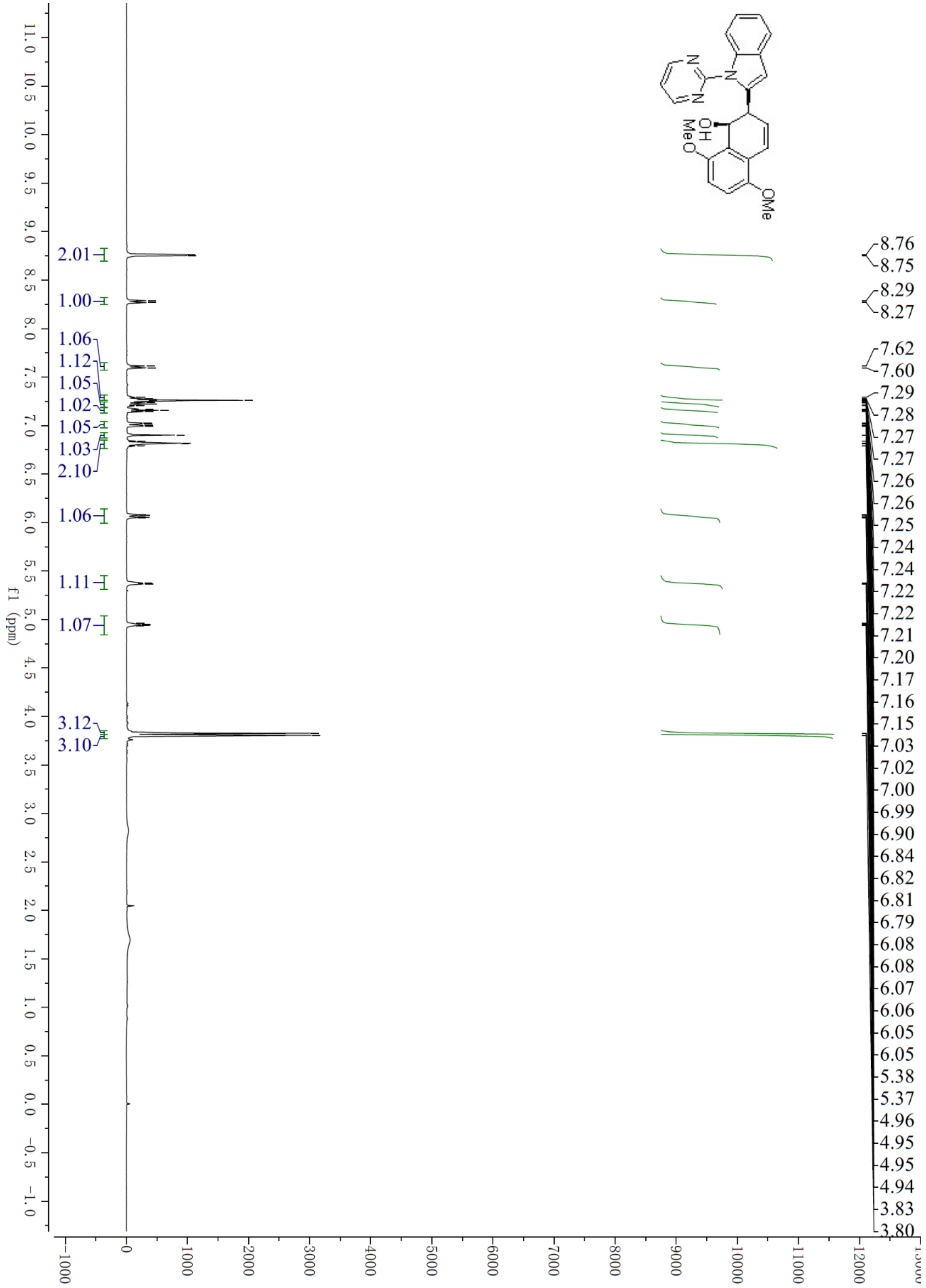

Supplementary Fig. 75 ^{13}C NMR Spectrum of *cis*-**3af**

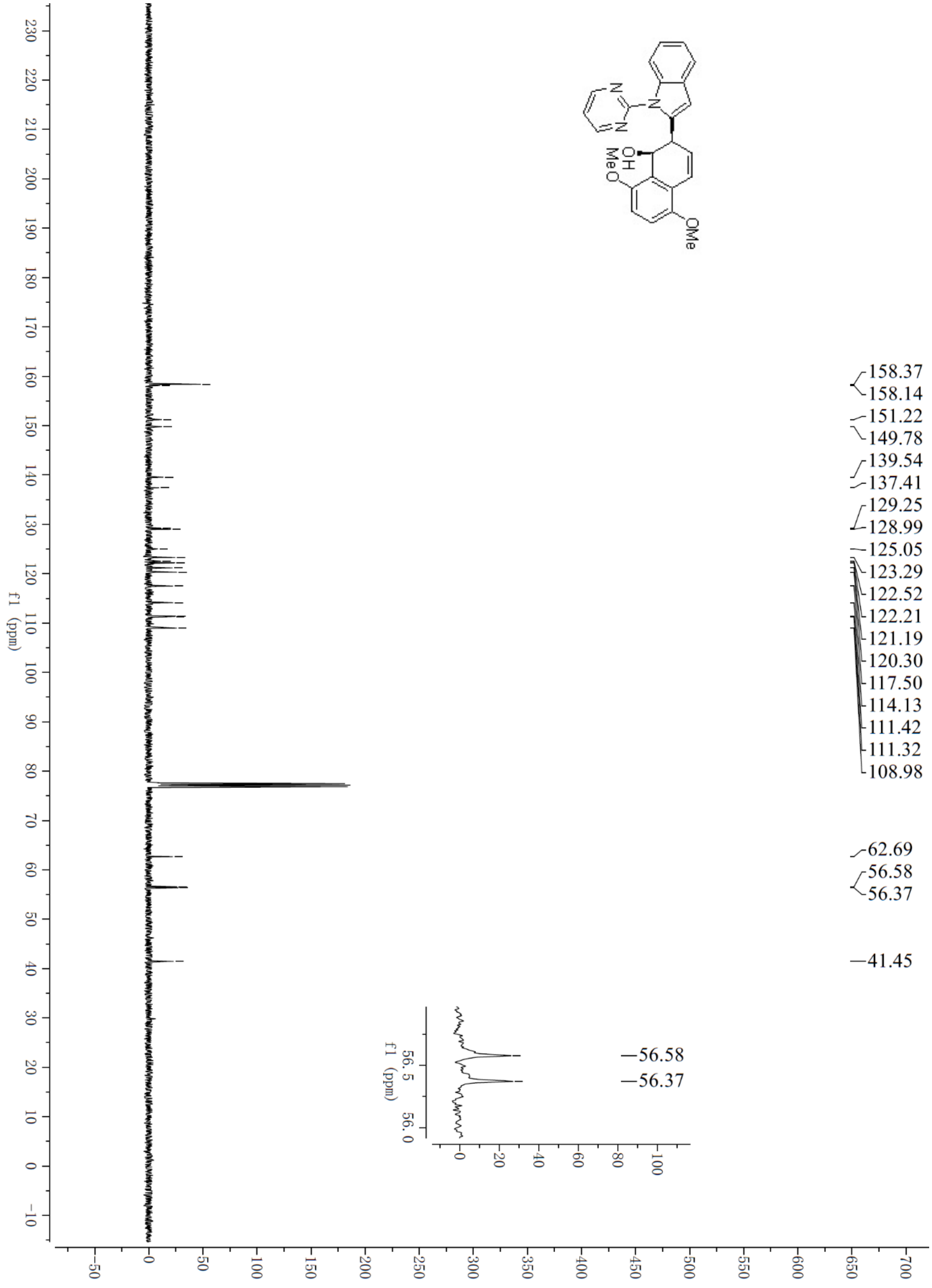

Supplementary Fig. 76 ^{1}H NMR Spectrum of *cis*-**3ag**

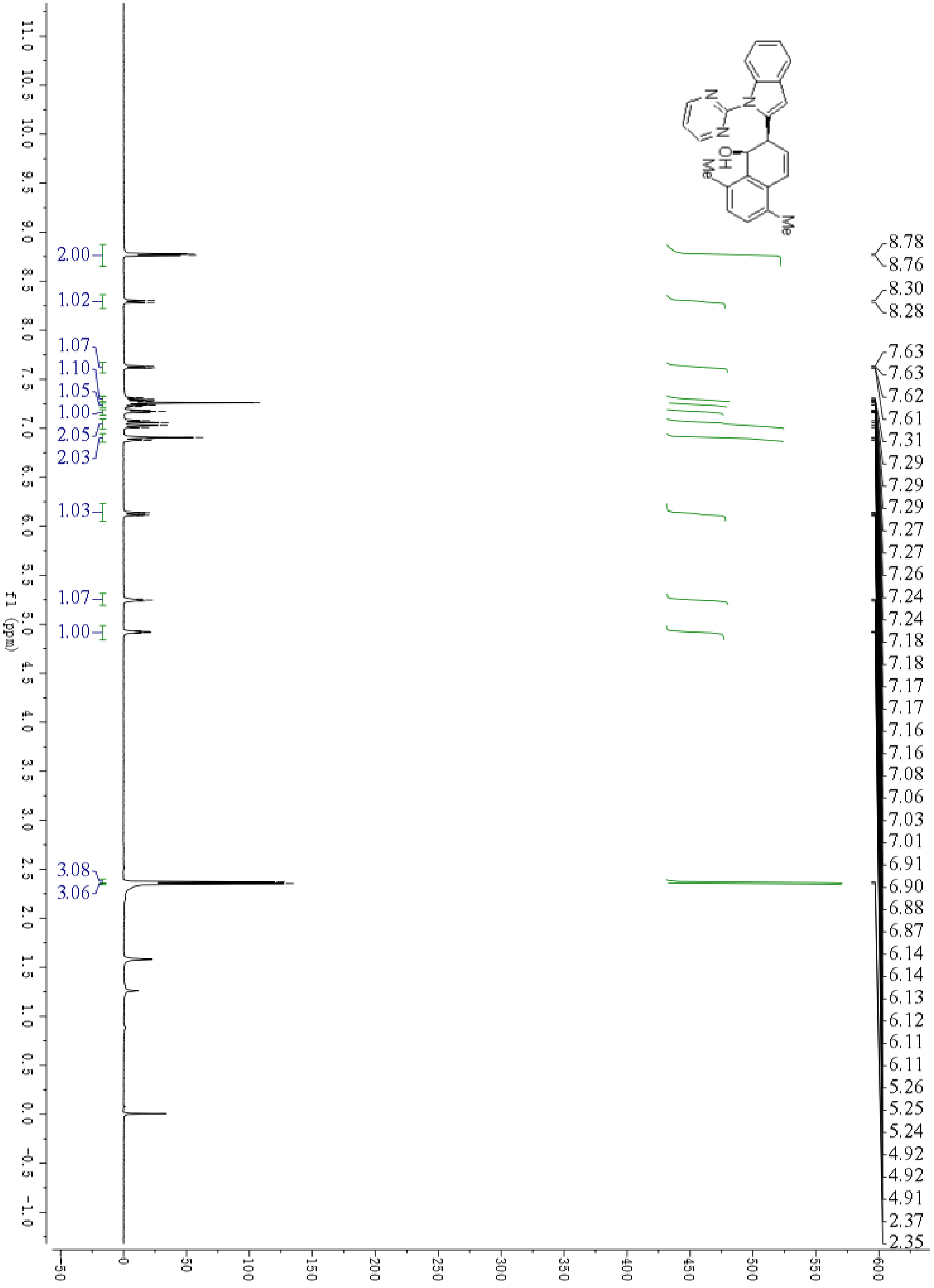

Supplementary Fig. 77 ^{13}C NMR Spectrum of *cis*-**3ag**

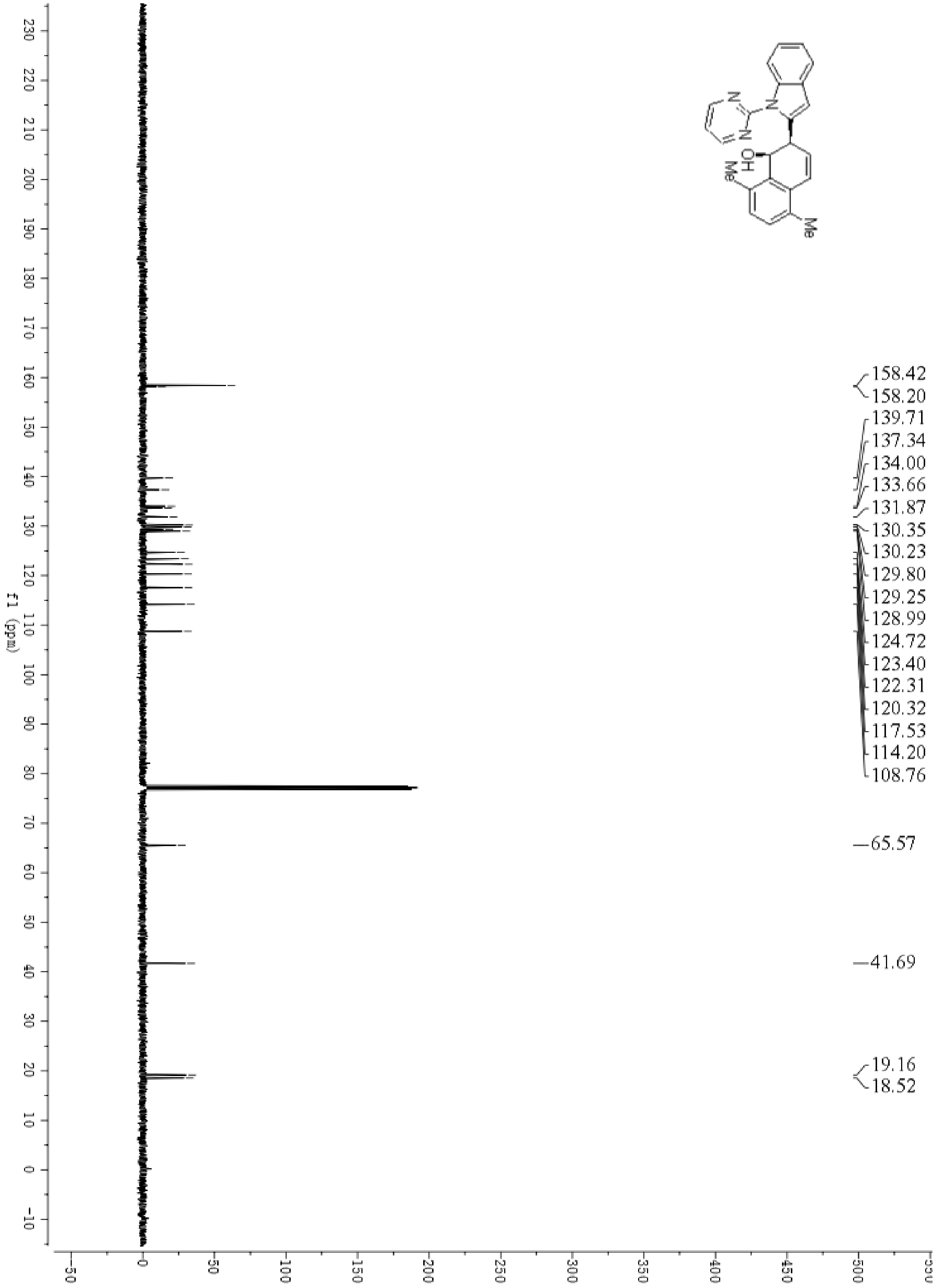

Supplementary Fig. 78 ^{1}H NMR Spectrum of *cis*-**3ah**

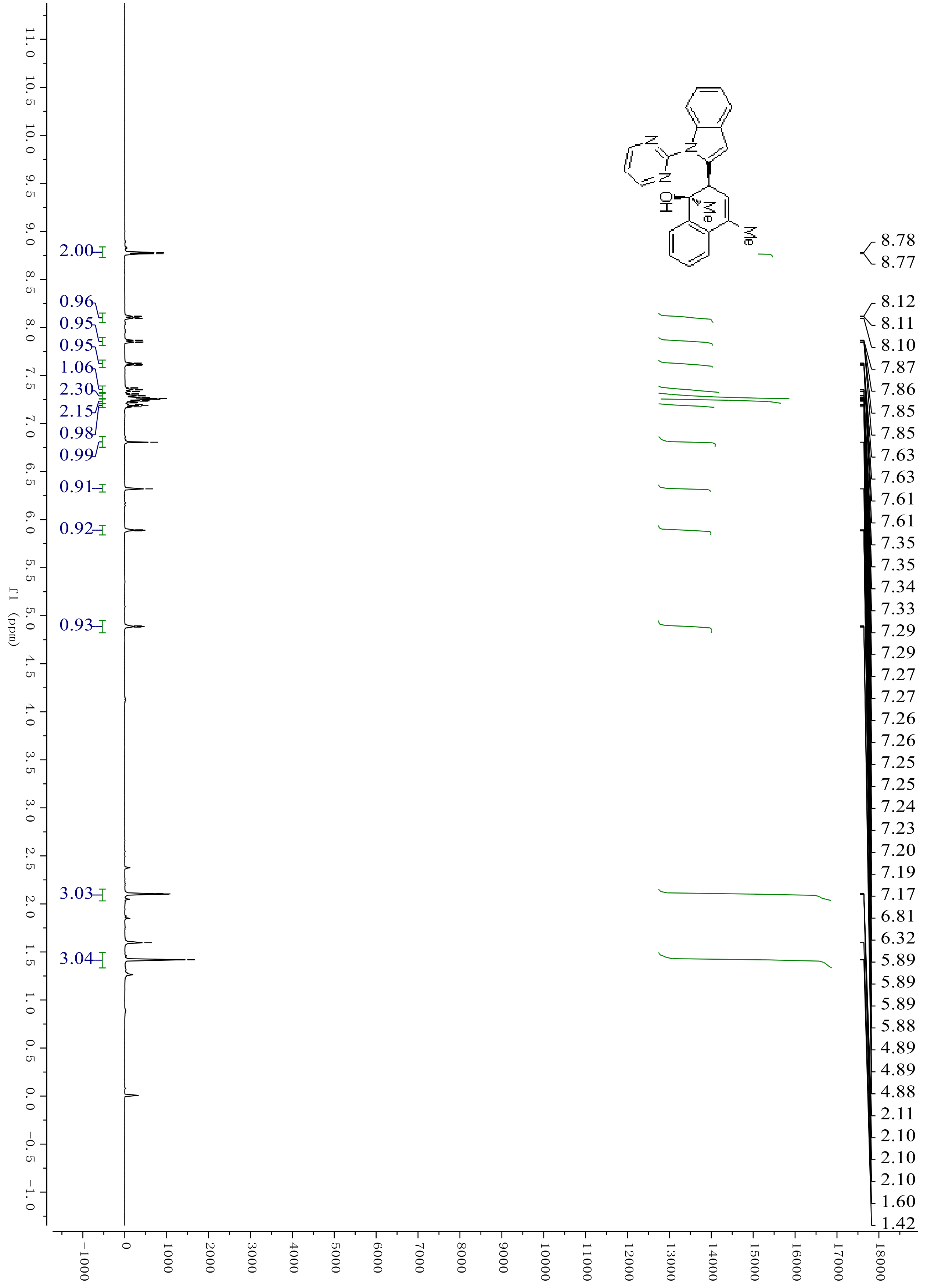

Supplementary Fig. 79 ^{13}C NMR Spectrum of *cis*-**3ah**

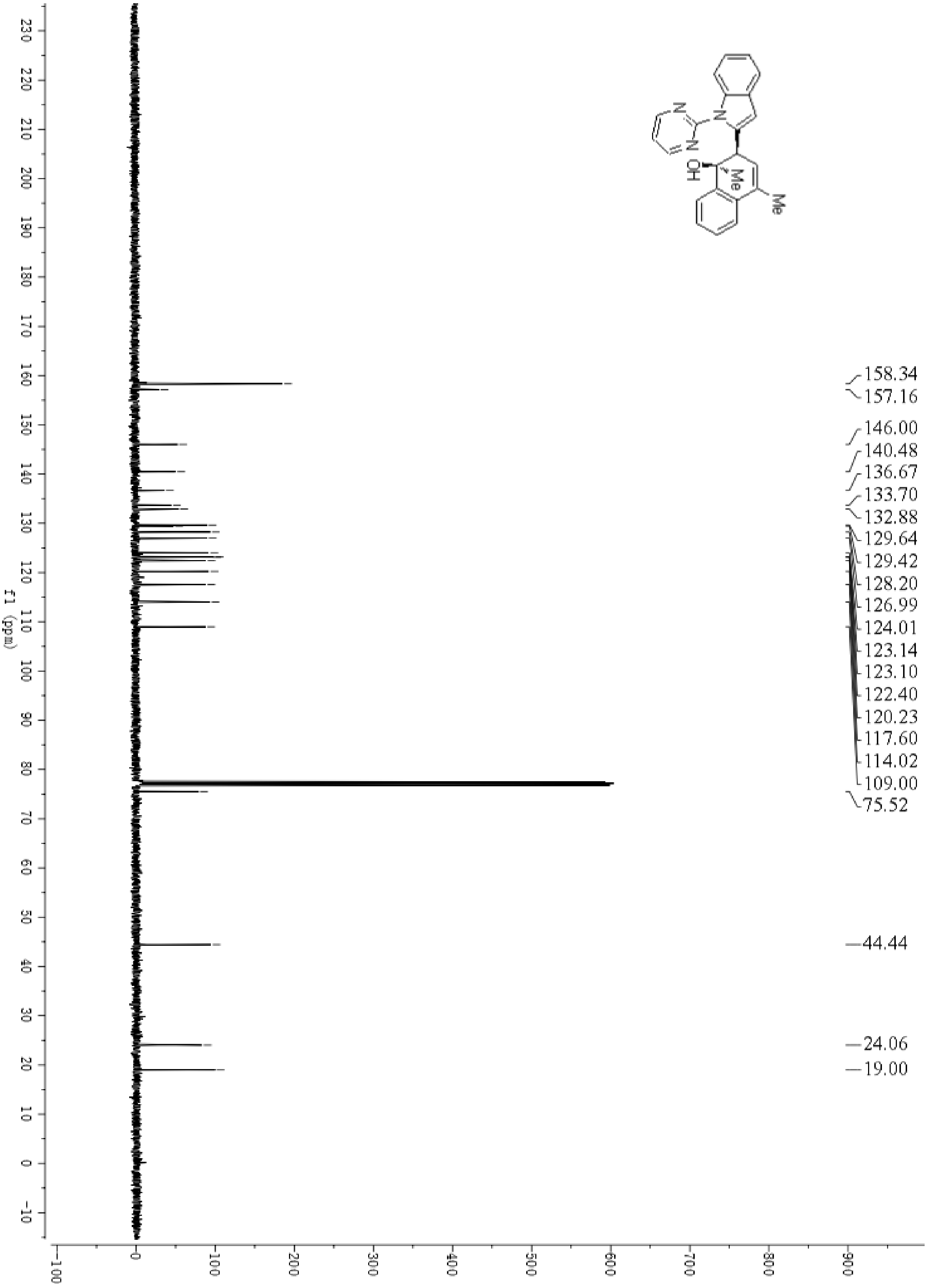

Supplementary Fig. 80 ^{1}H NMR Spectrum of *cis*-**3ai**

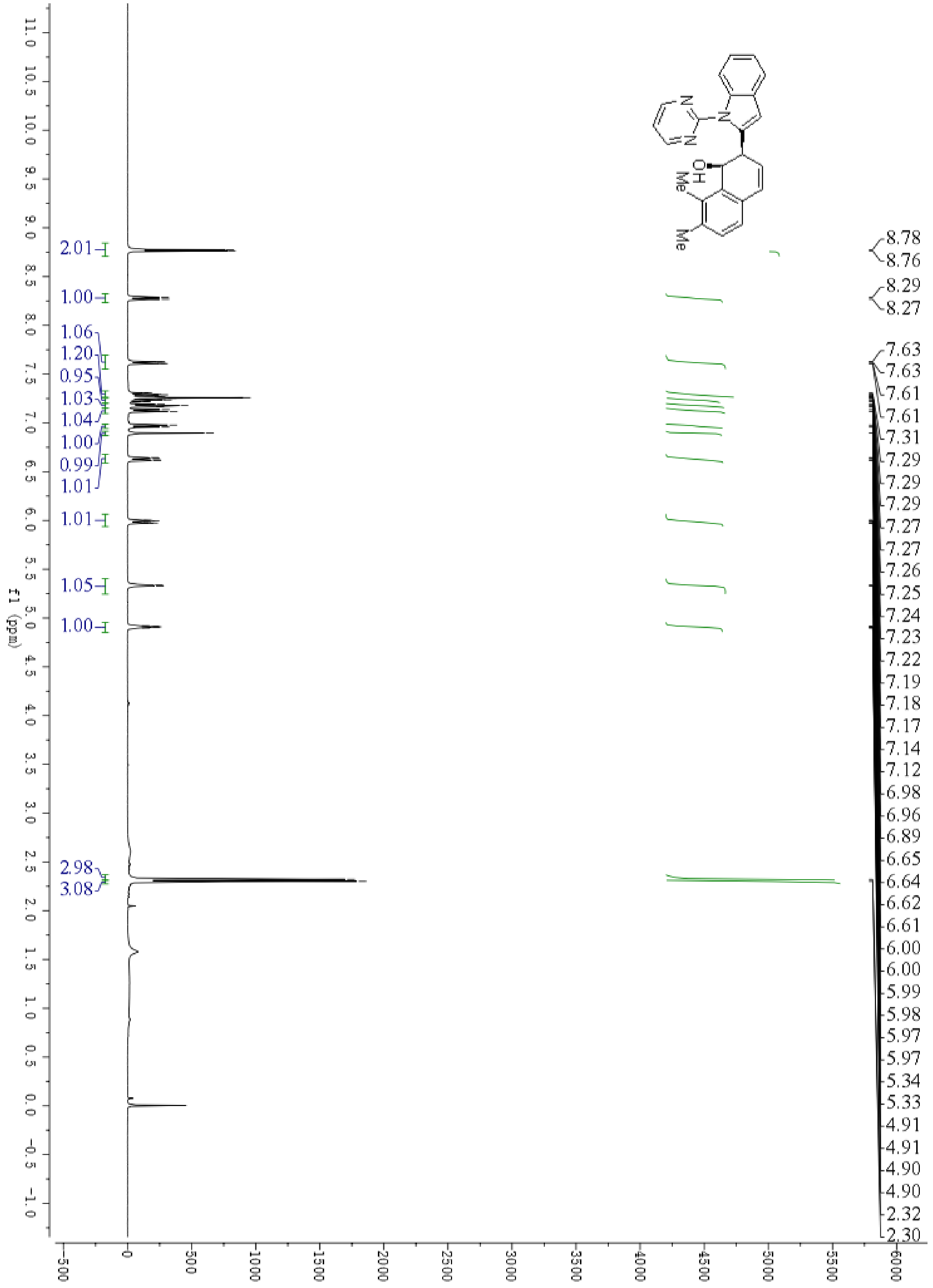

Supplementary Fig. 81 ^{13}C NMR Spectrum of *cis*-**3ai**

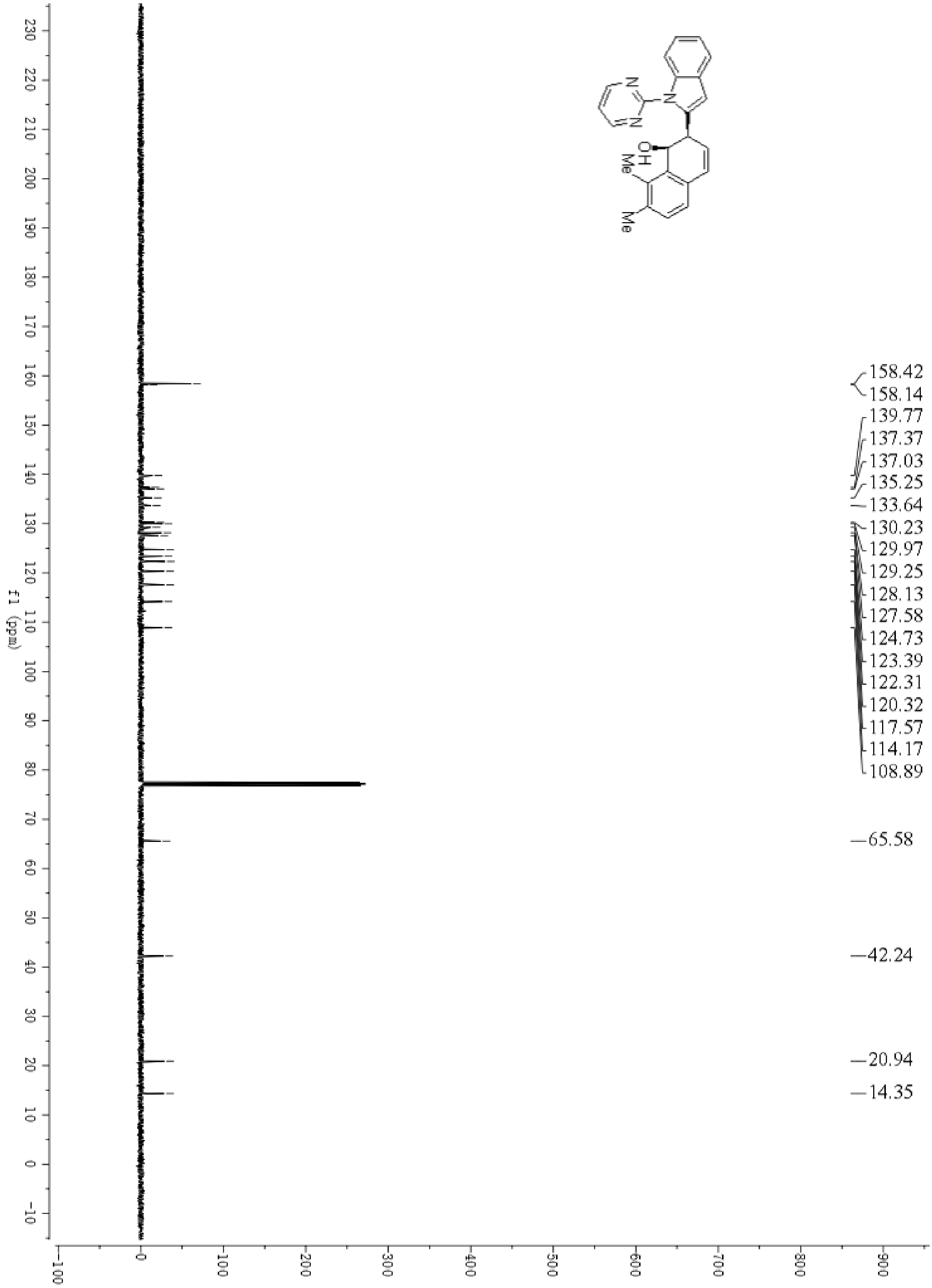

Supplementary Fig. 82 1D-nosey NMR Spectra for *cis*-**3ai**

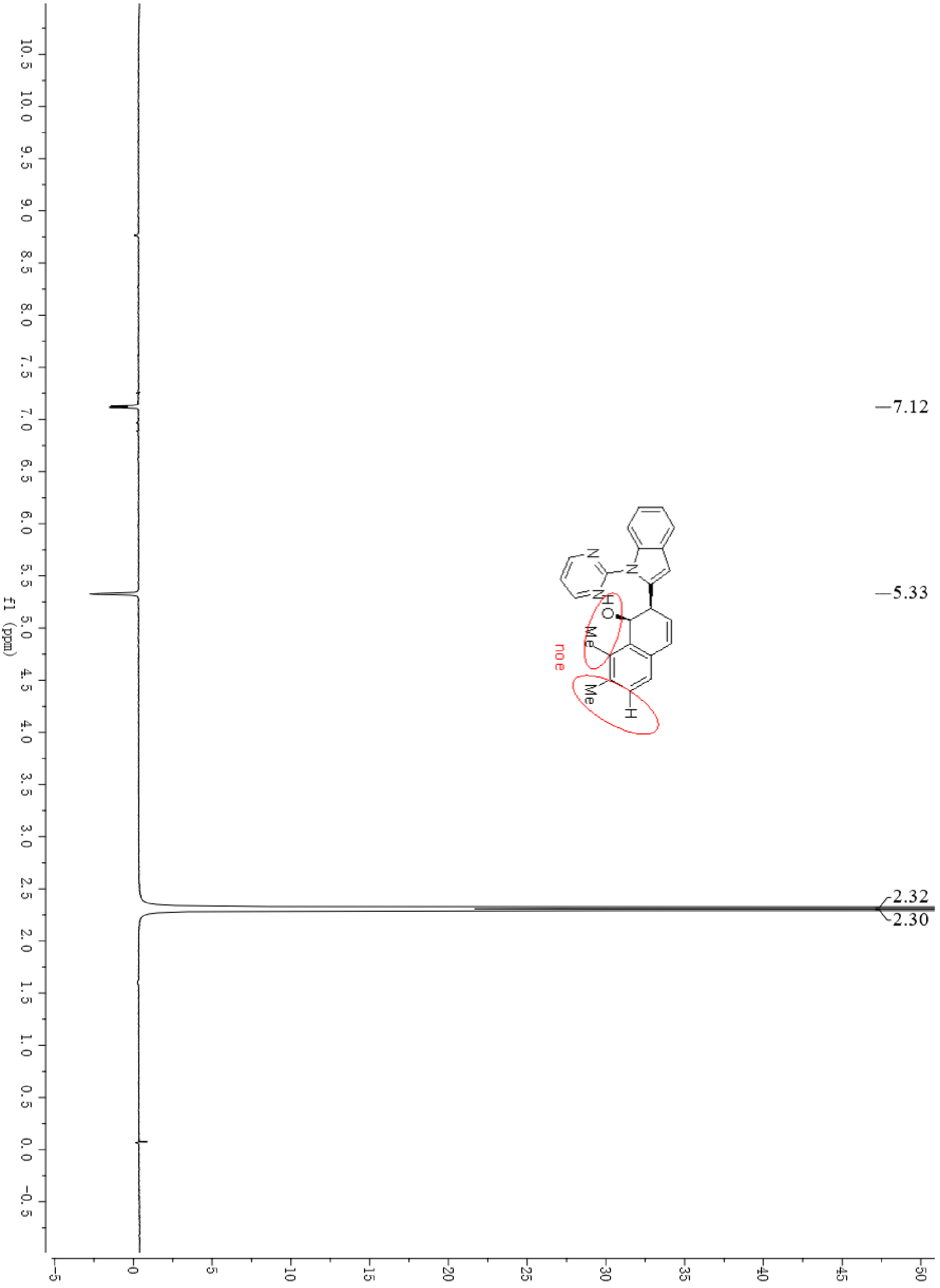

Supplementary Fig. 83 ^{1}H NMR Spectrum of *cis*-**3ai'**

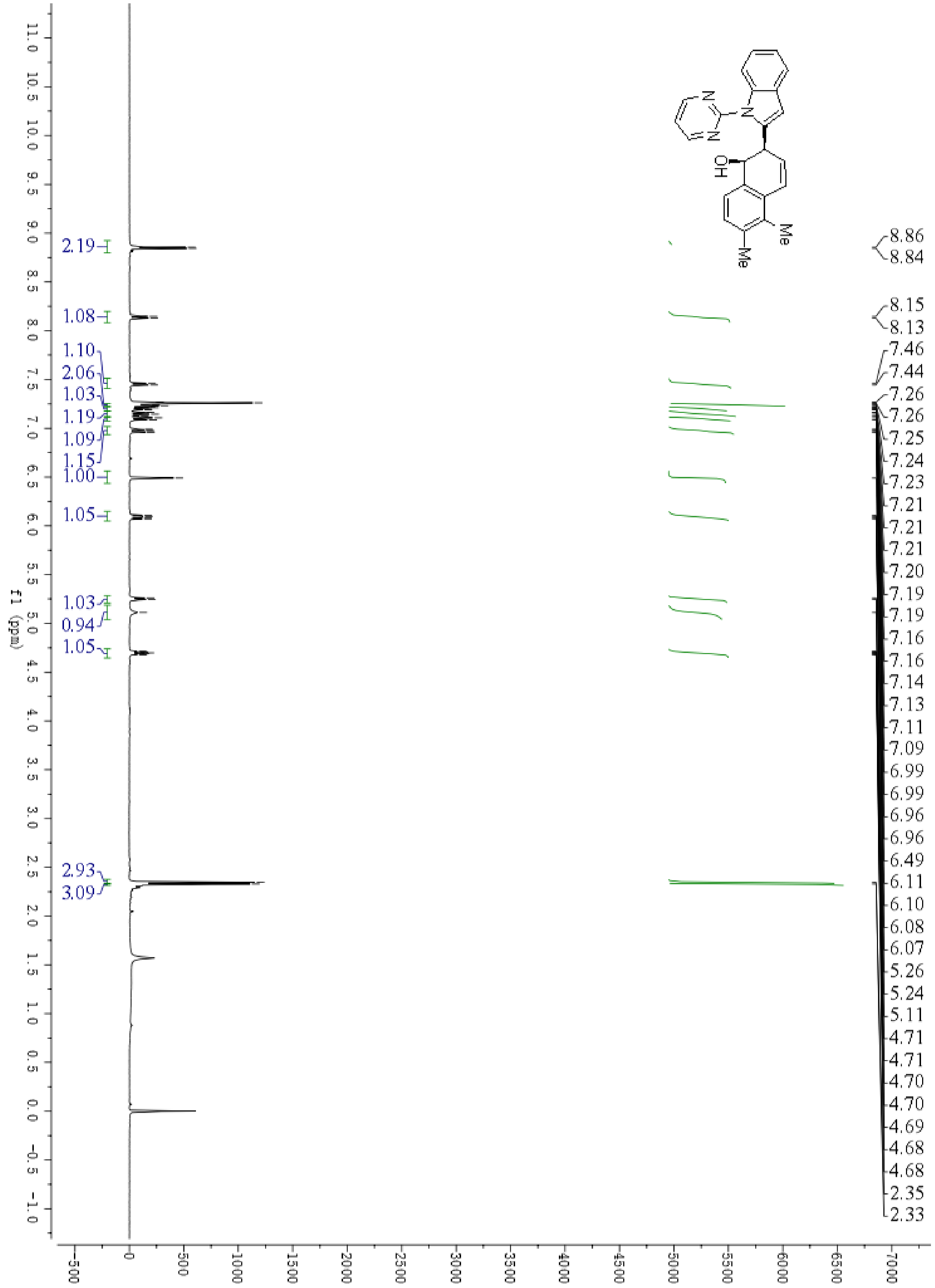

Supplementary Fig. 84 ^{13}C NMR Spectrum of *cis*-**3ai'**

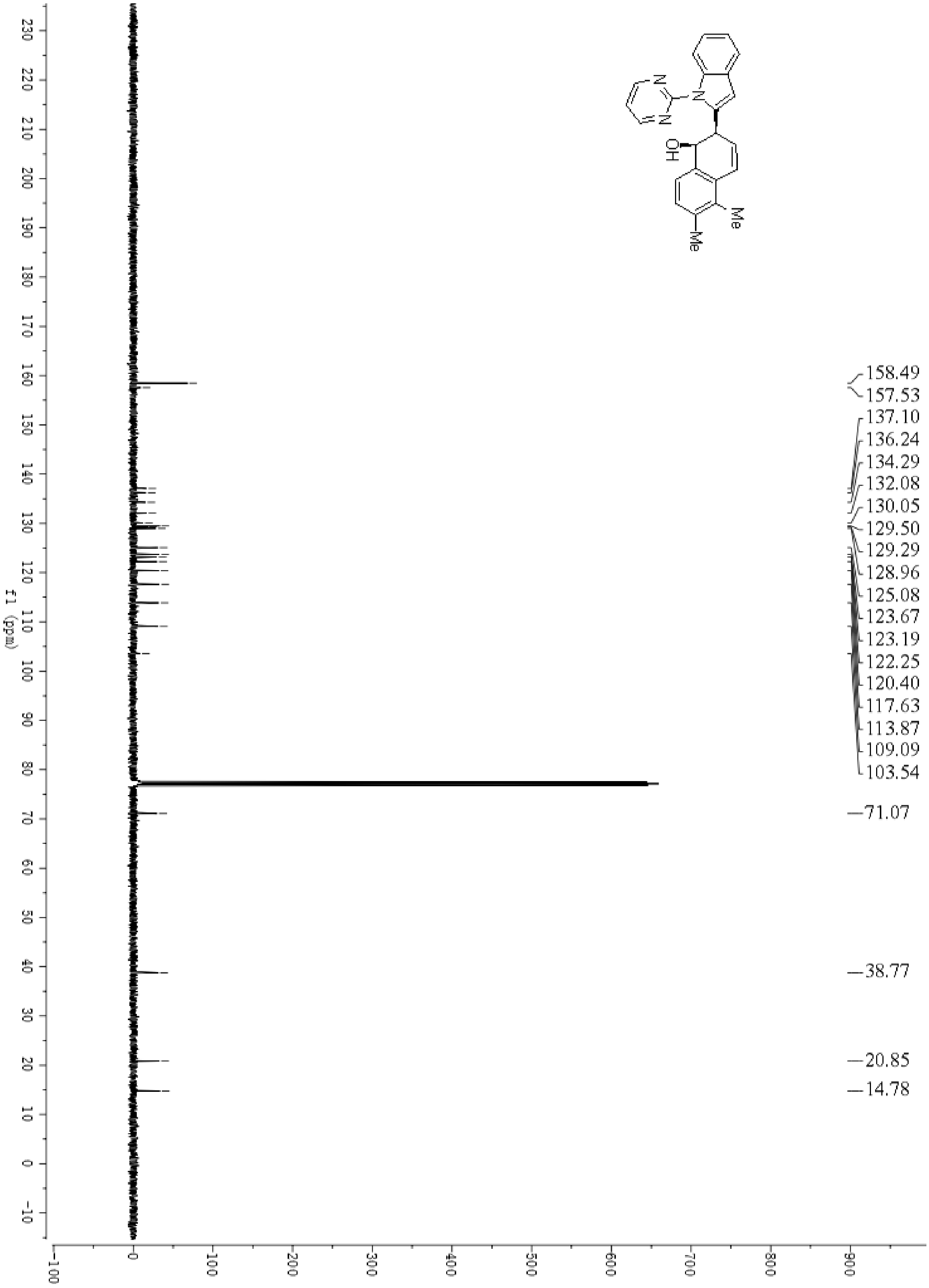

Supplementary Fig. 85 1D-nosey NMR Spectra for *cis*-**3ai'**

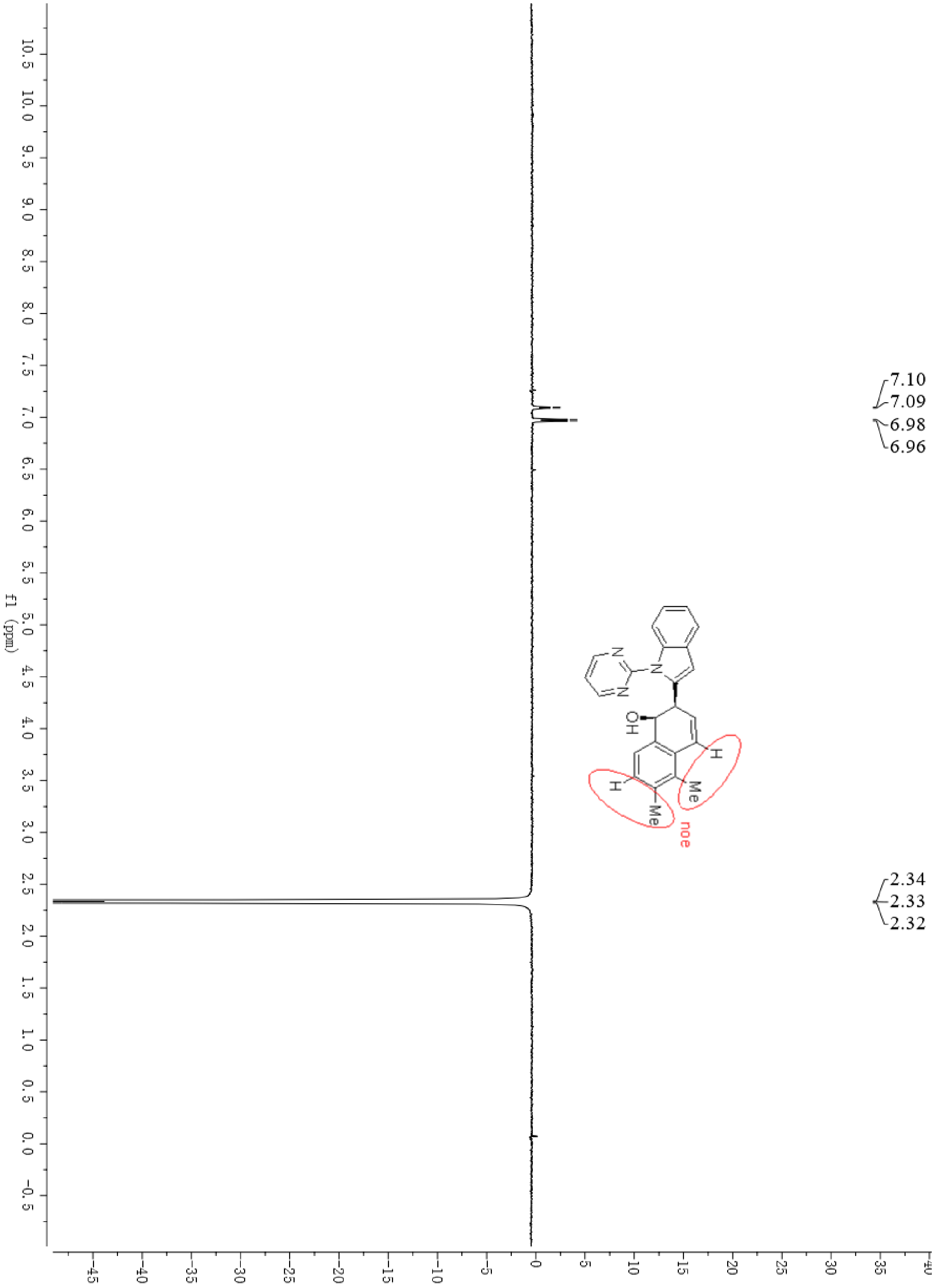

Supplementary Fig. 86 ^{1}H NMR Spectrum of *cis*-**7aa**

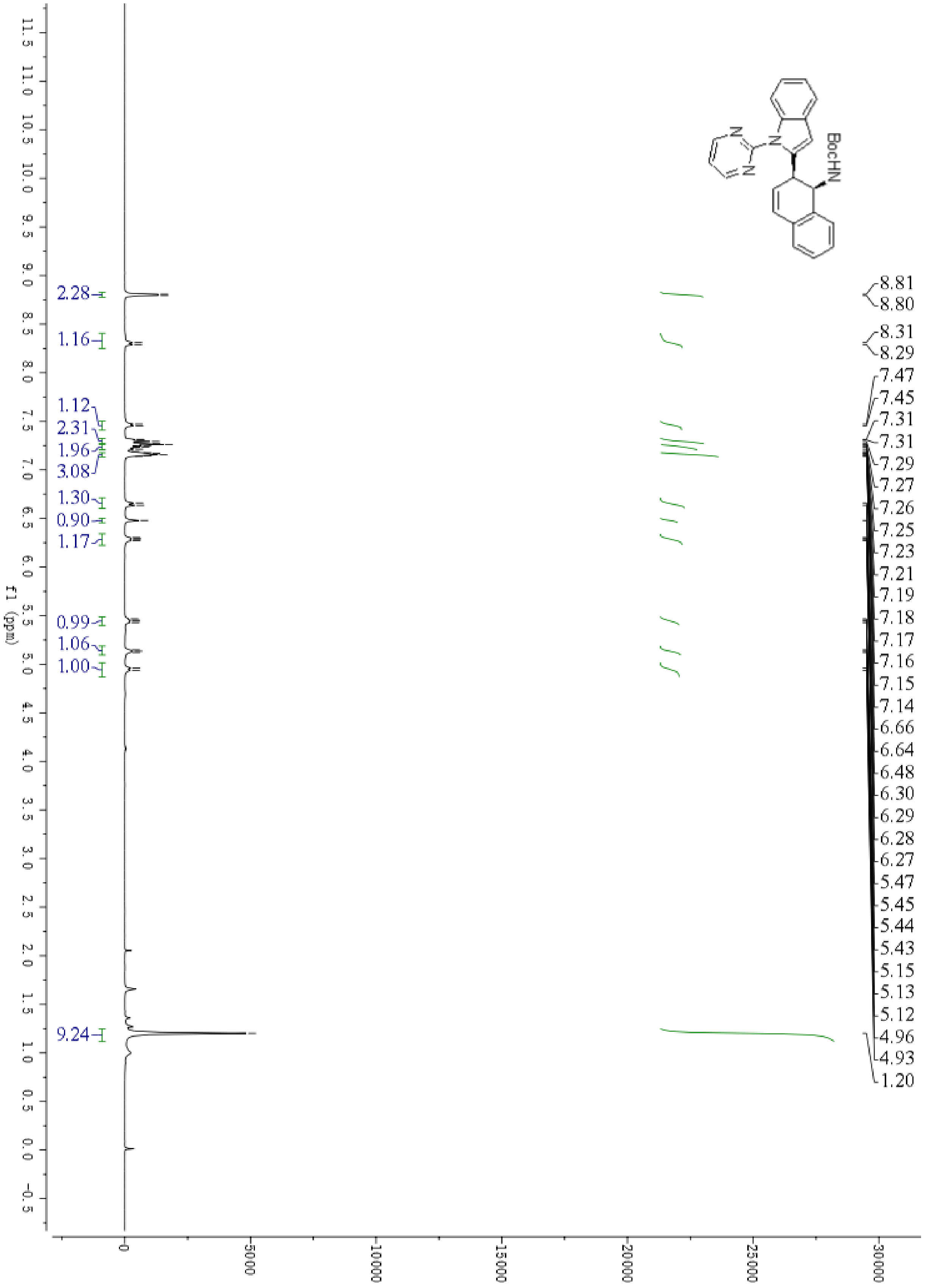

Supplementary Fig. 87 ^{13}C NMR Spectrum of *cis*-**7aa**

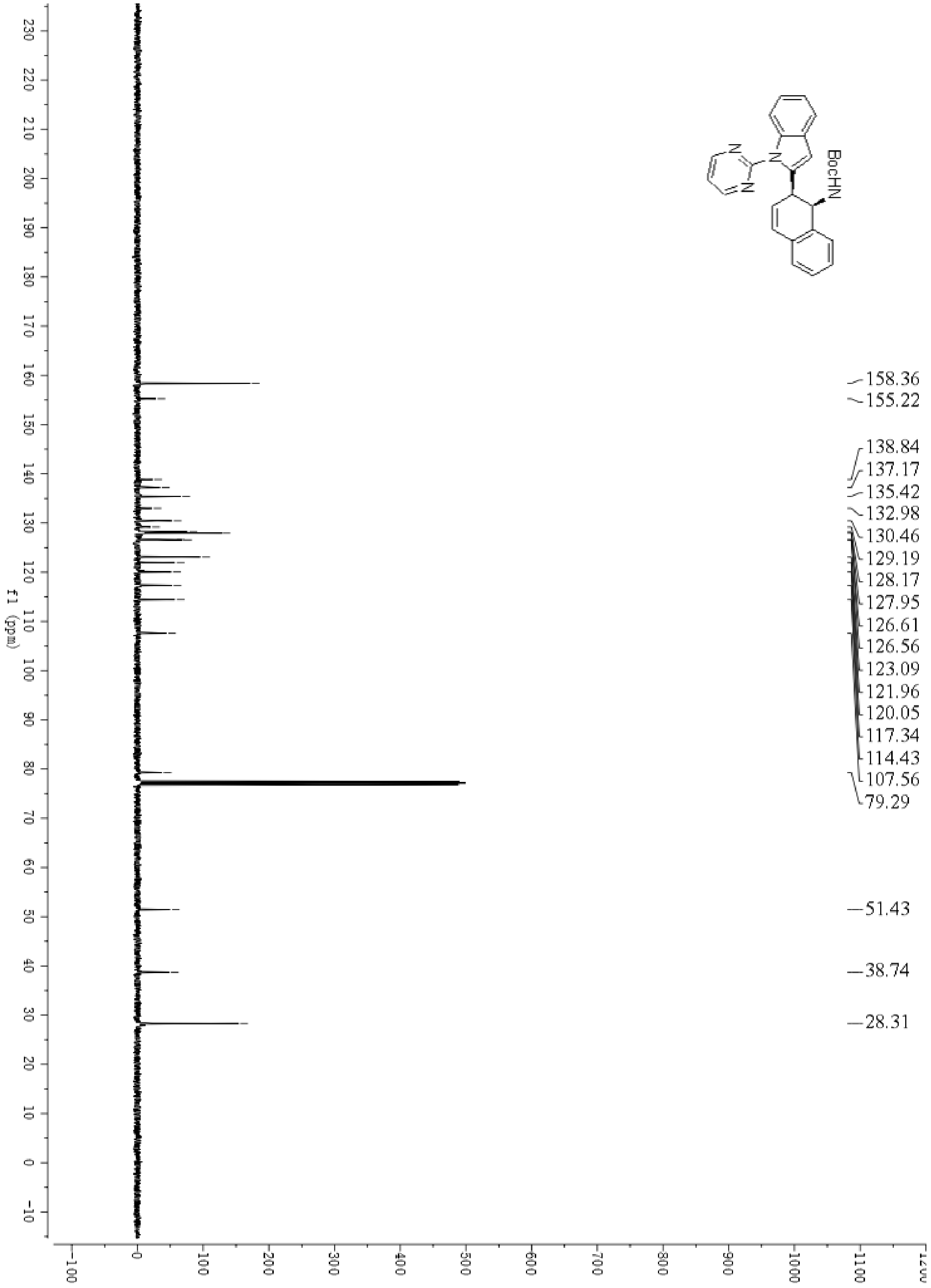

Supplementary Fig. 88 ^{1}H NMR Spectrum of *cis*-**7ab**

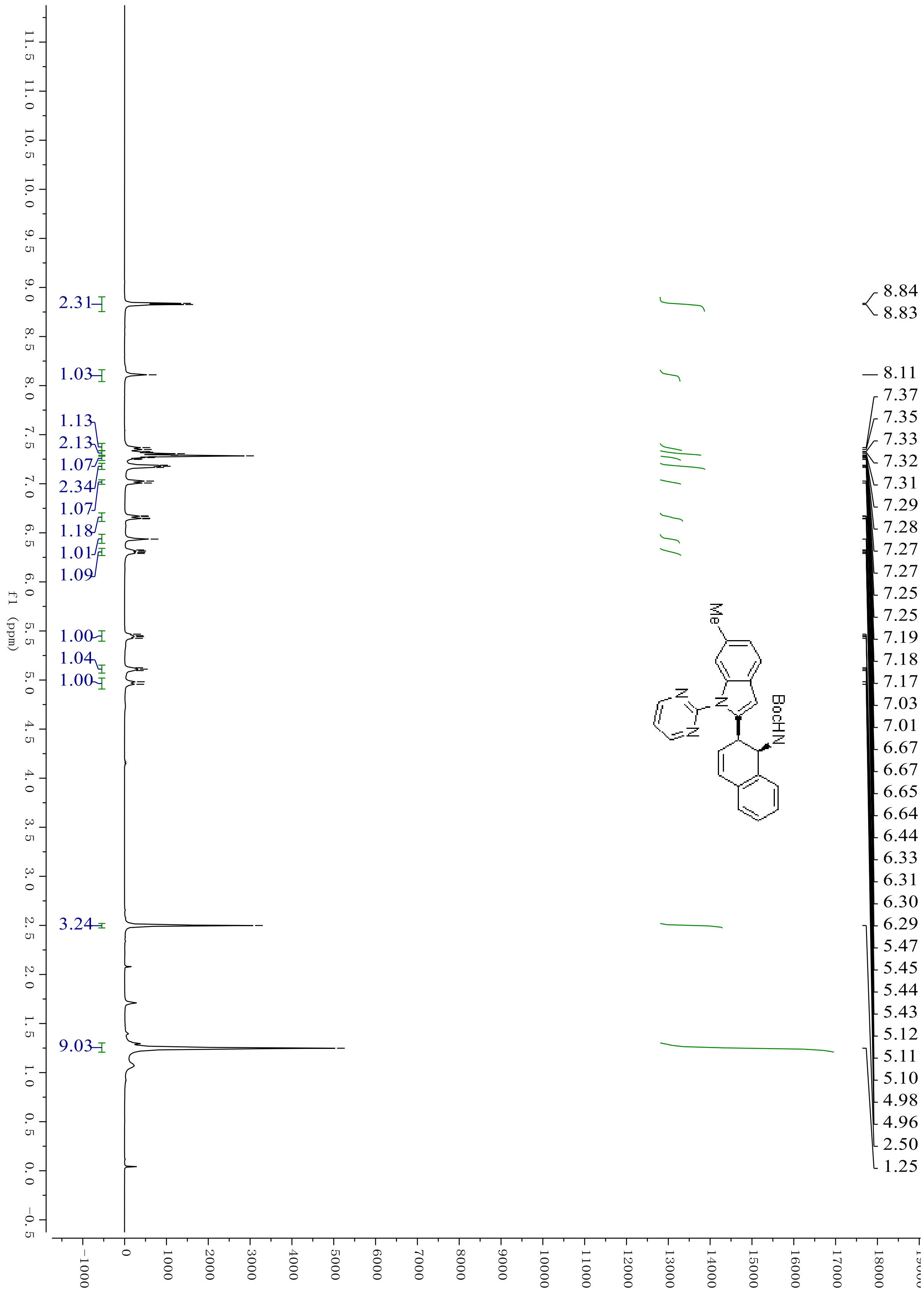

Supplementary Fig. 89 ^{13}C NMR Spectrum of *cis*-**7ab**

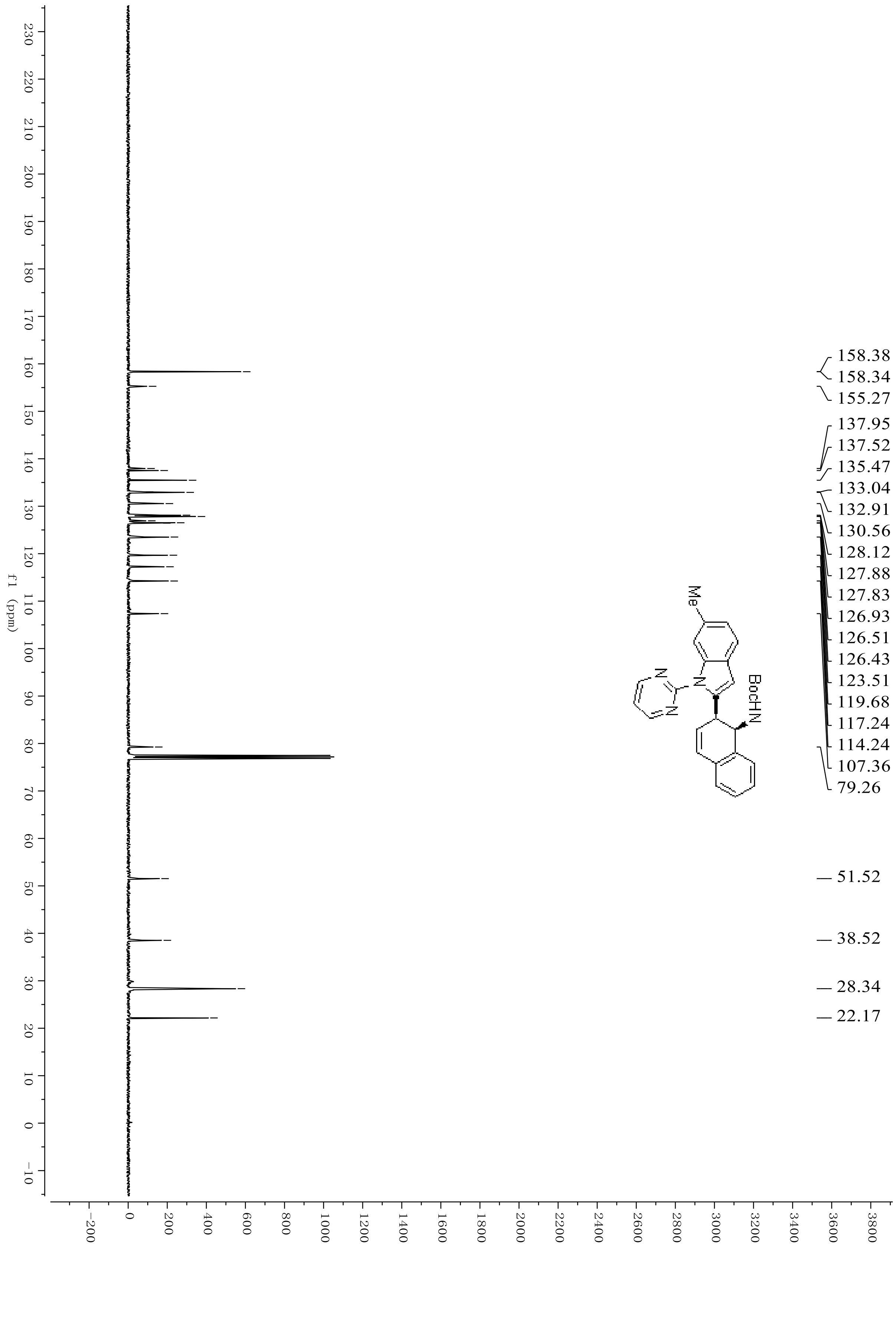

Supplementary Fig. 90 ^{1}H NMR Spectrum of *cis*-**7ac**

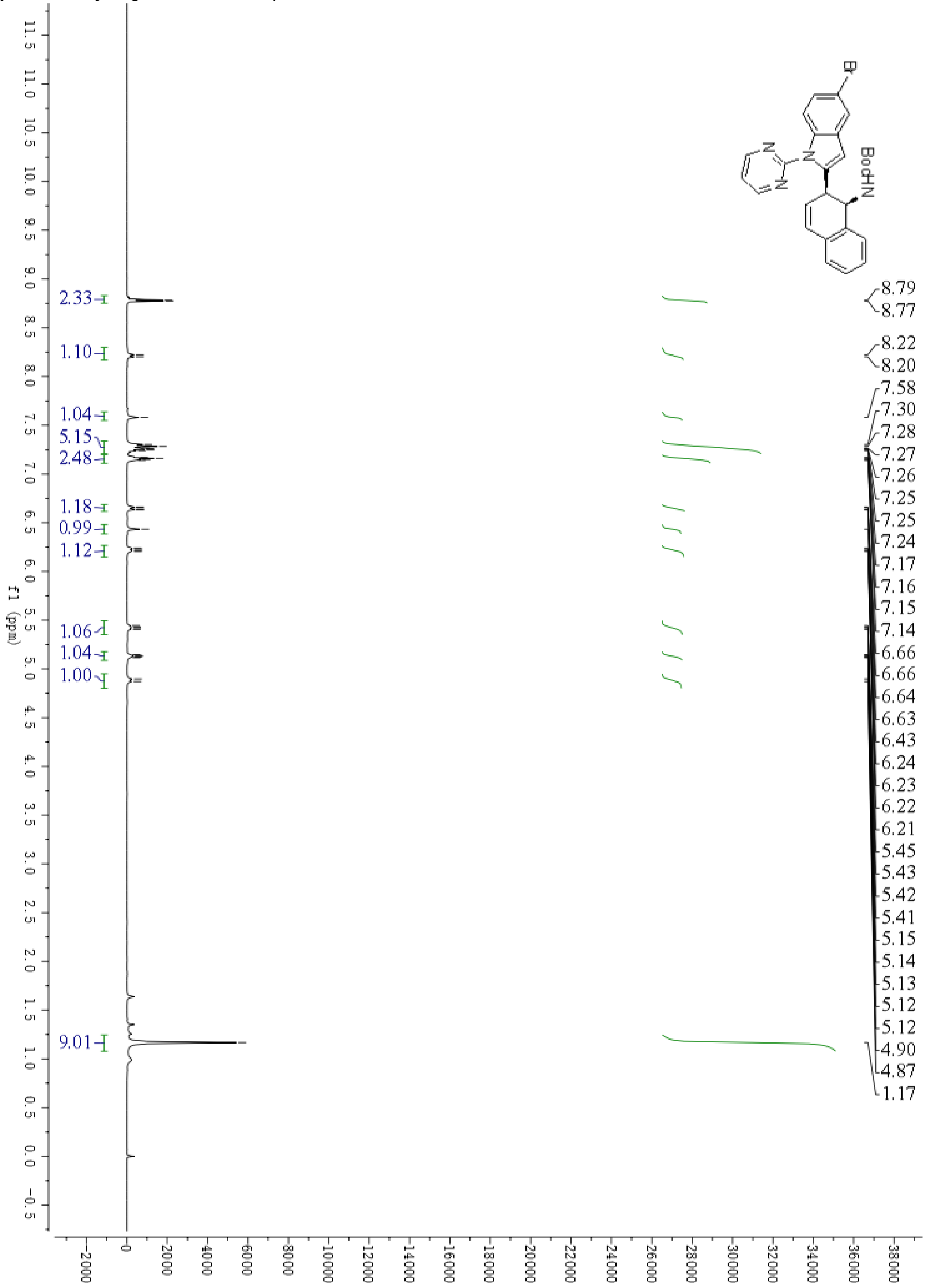

Supplementary Fig. 91 ^{13}C NMR Spectrum of *cis*-**7ac**

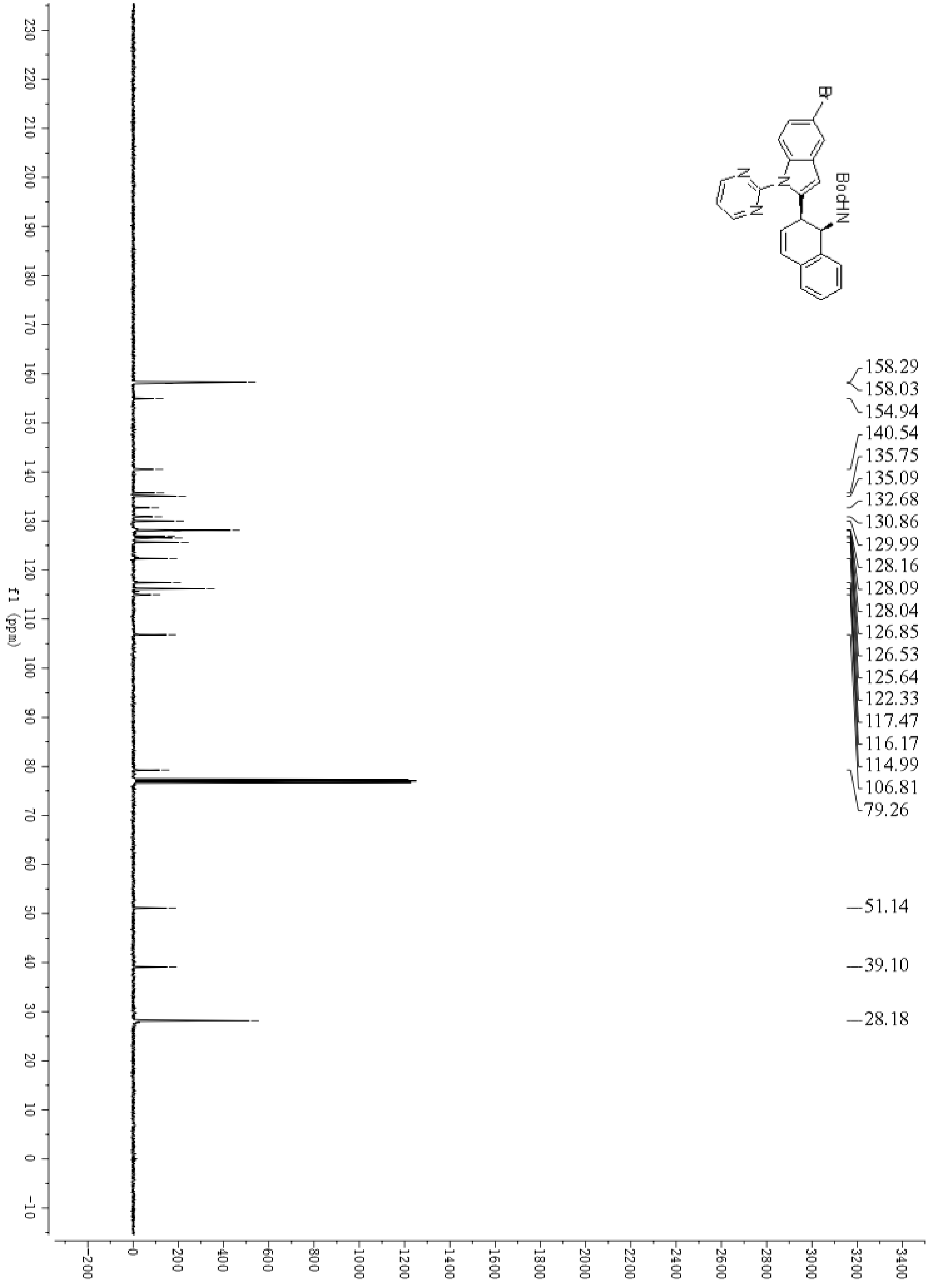

Supplementary Fig. 92 ^{1}H NMR Spectrum of *cis*-**7ad**

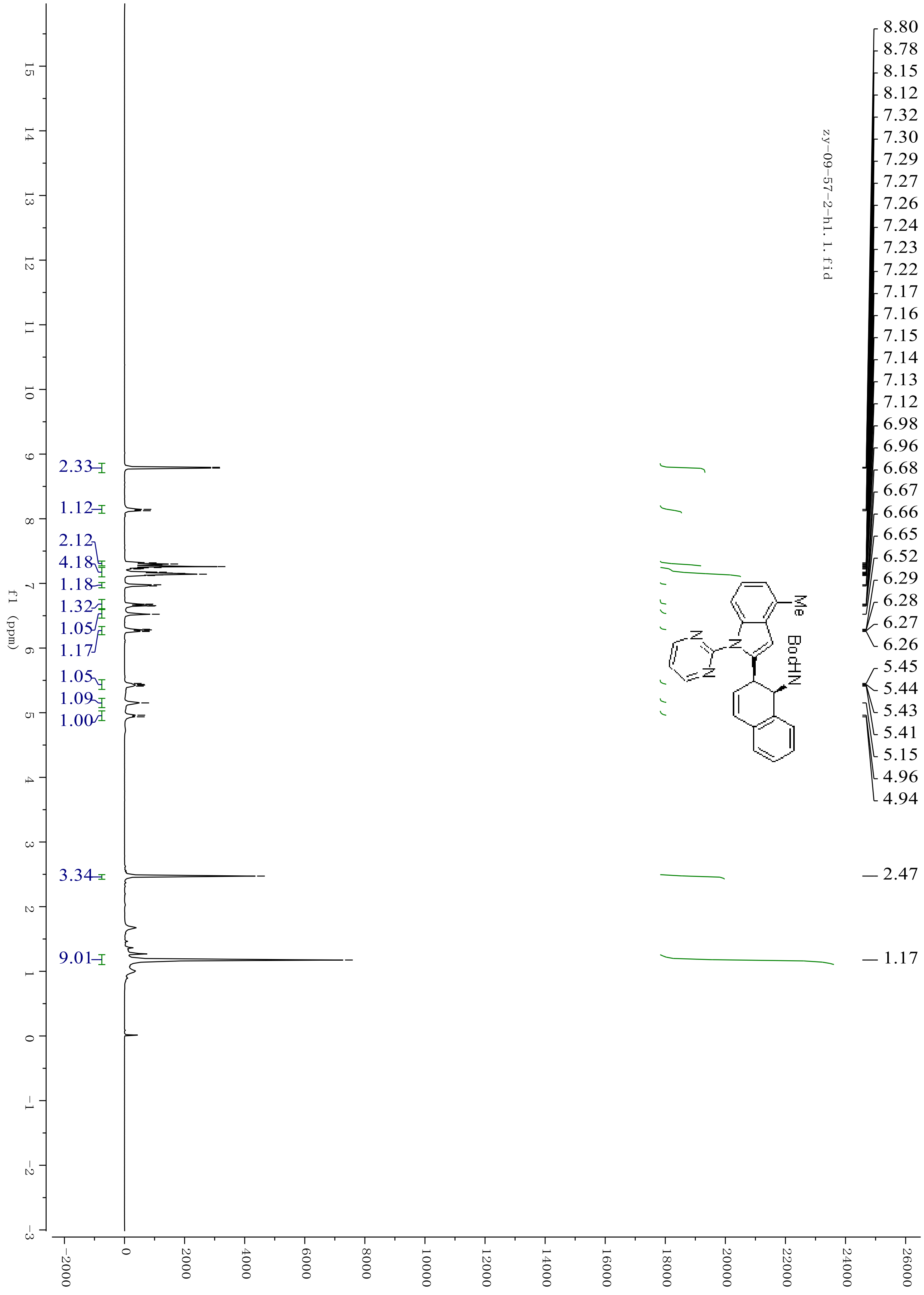

Supplementary Fig. 93 ^{13}C NMR Spectrum of *cis*-**7ad**

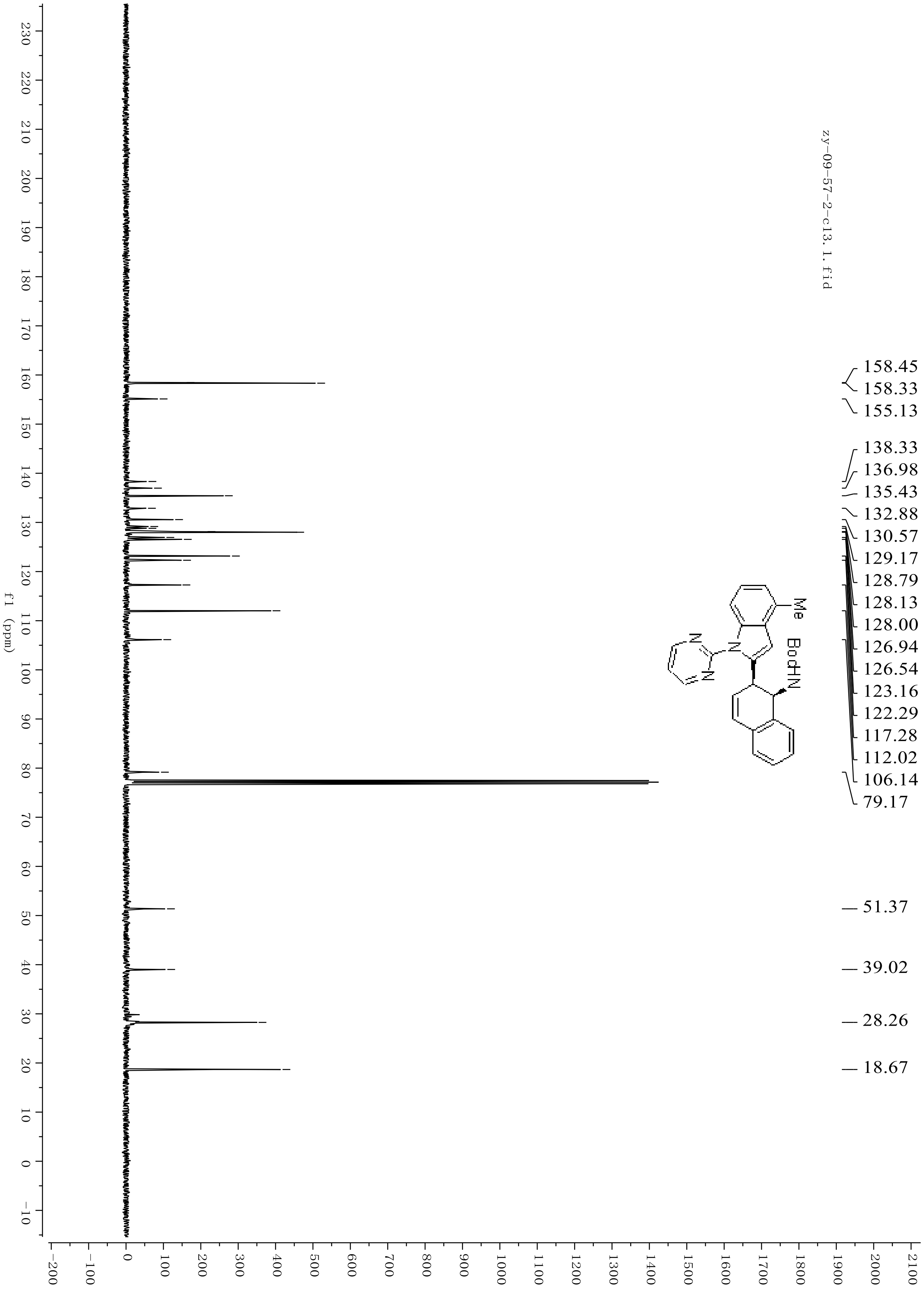

Supplementary Fig. 94 ^{1}H NMR Spectrum of *cis*-**7ae**

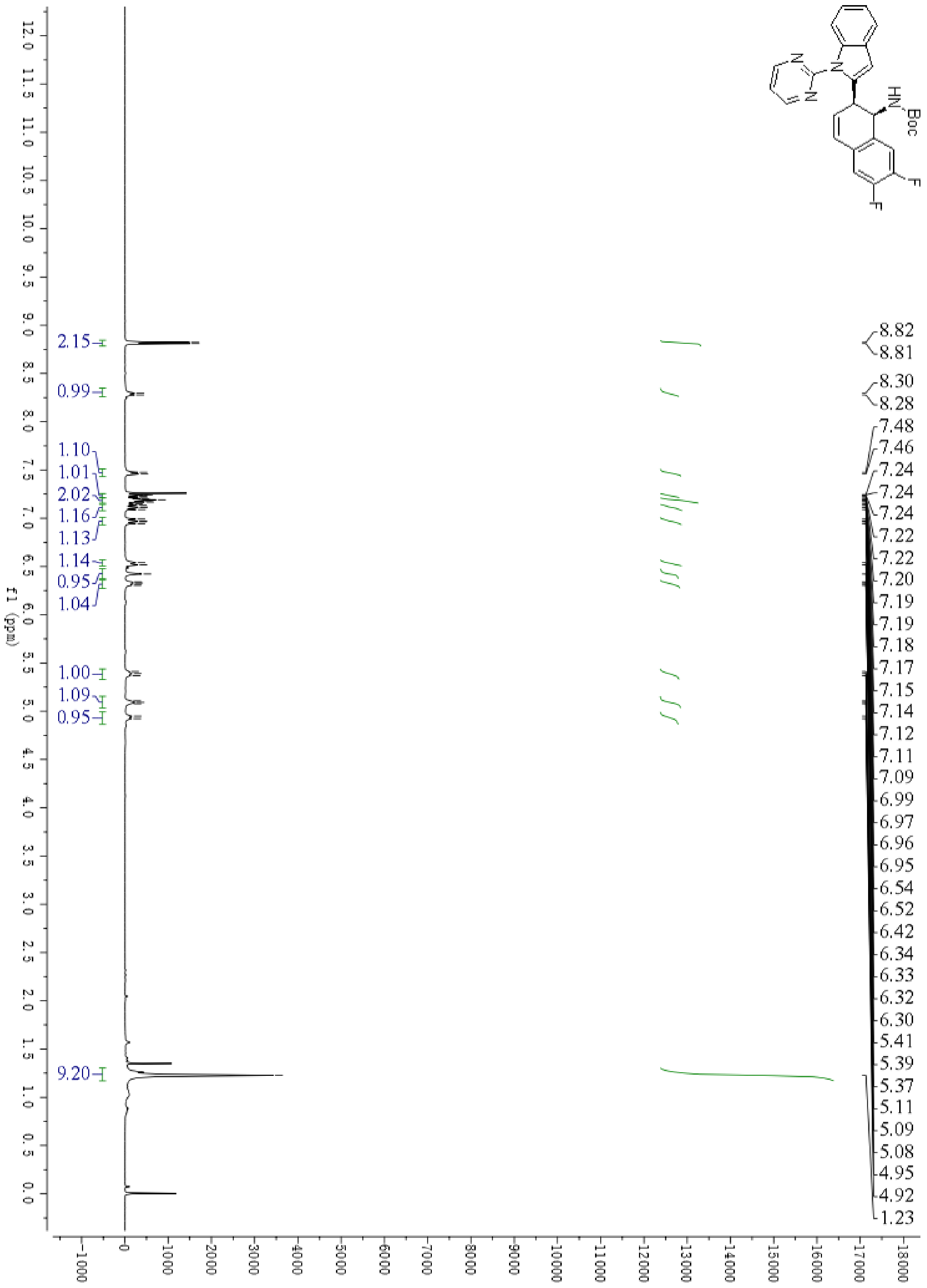

Supplementary Fig. 95 ^{13}C NMR Spectrum of *cis*-**7ae**

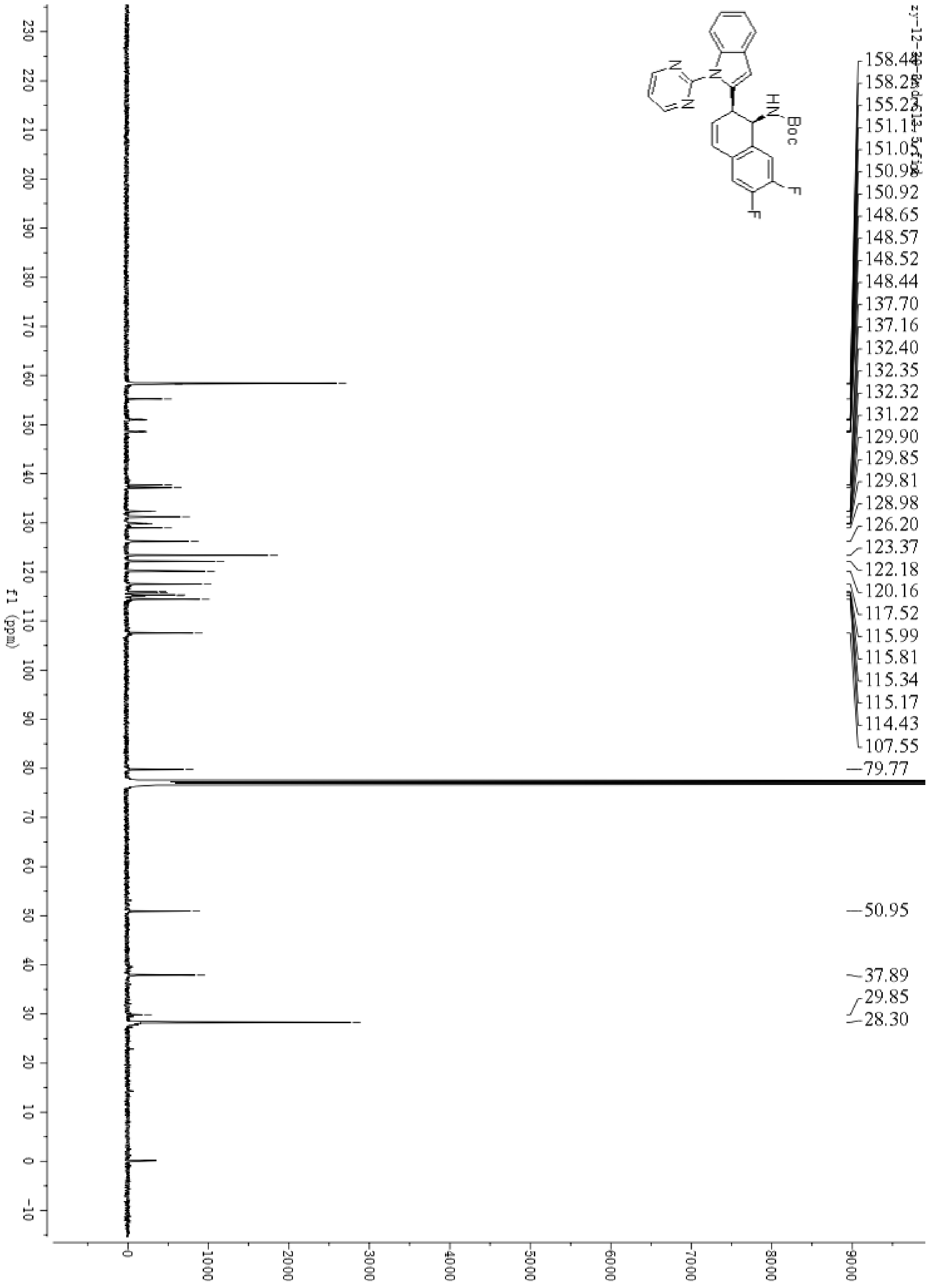

Supplementary Fig. 96 ^{19}F NMR Spectrum of *cis*-**7ae**

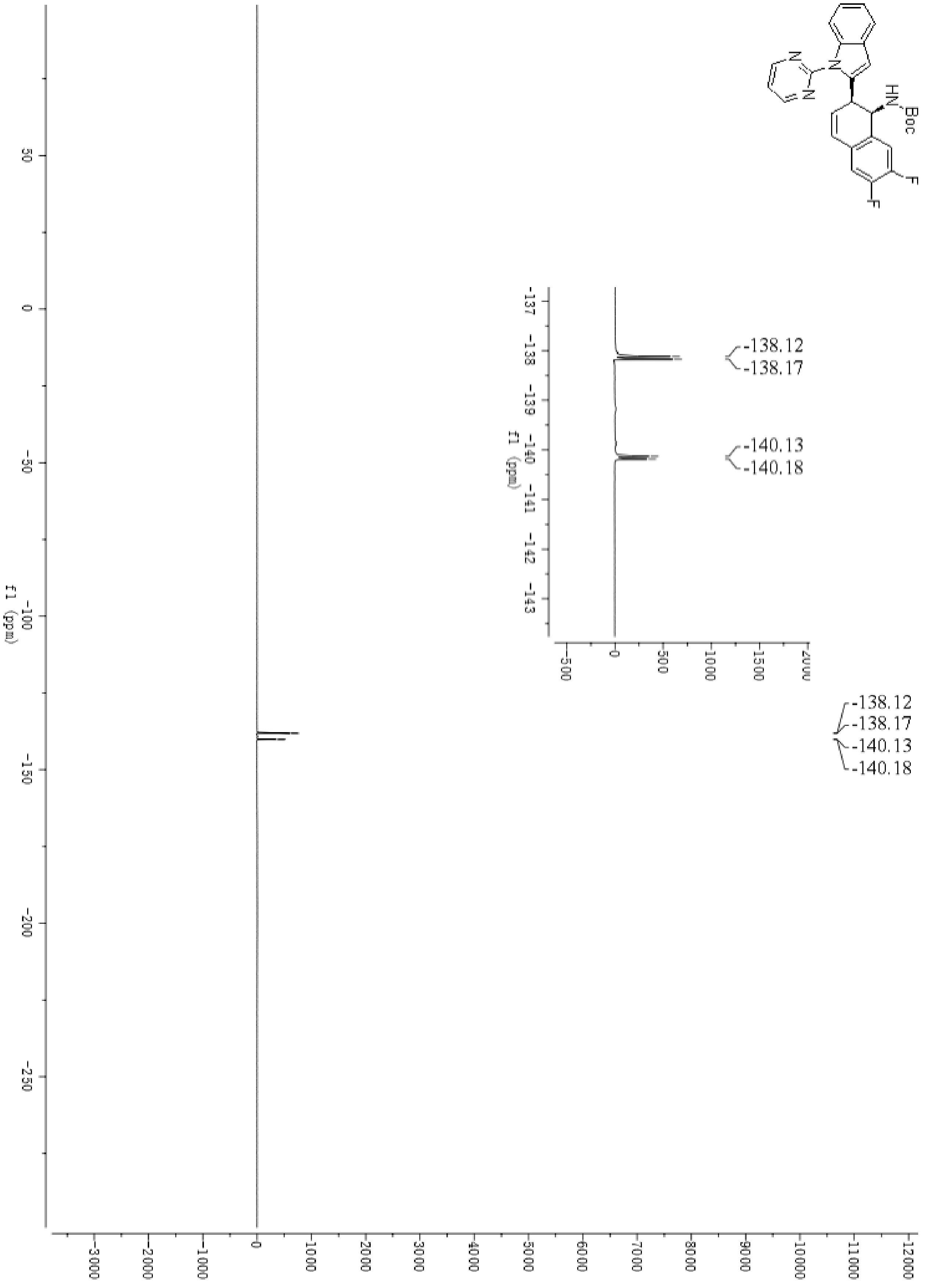

Supplementary Fig. 97 ^{1}H NMR Spectrum of *cis*-**7af**

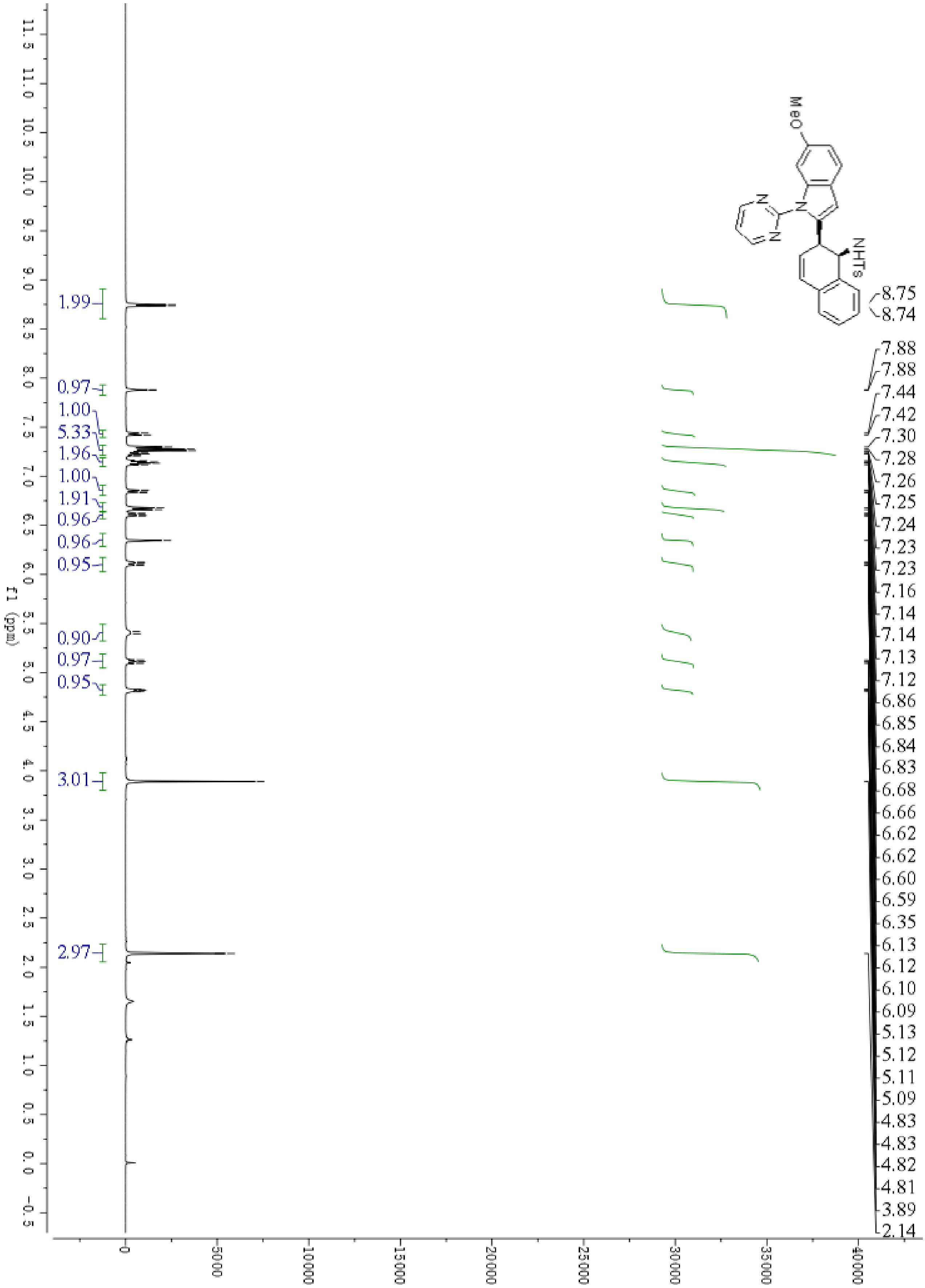

Supplementary Fig. 98 ^{1}H NMR Spectrum of *trans*-**7aa.**

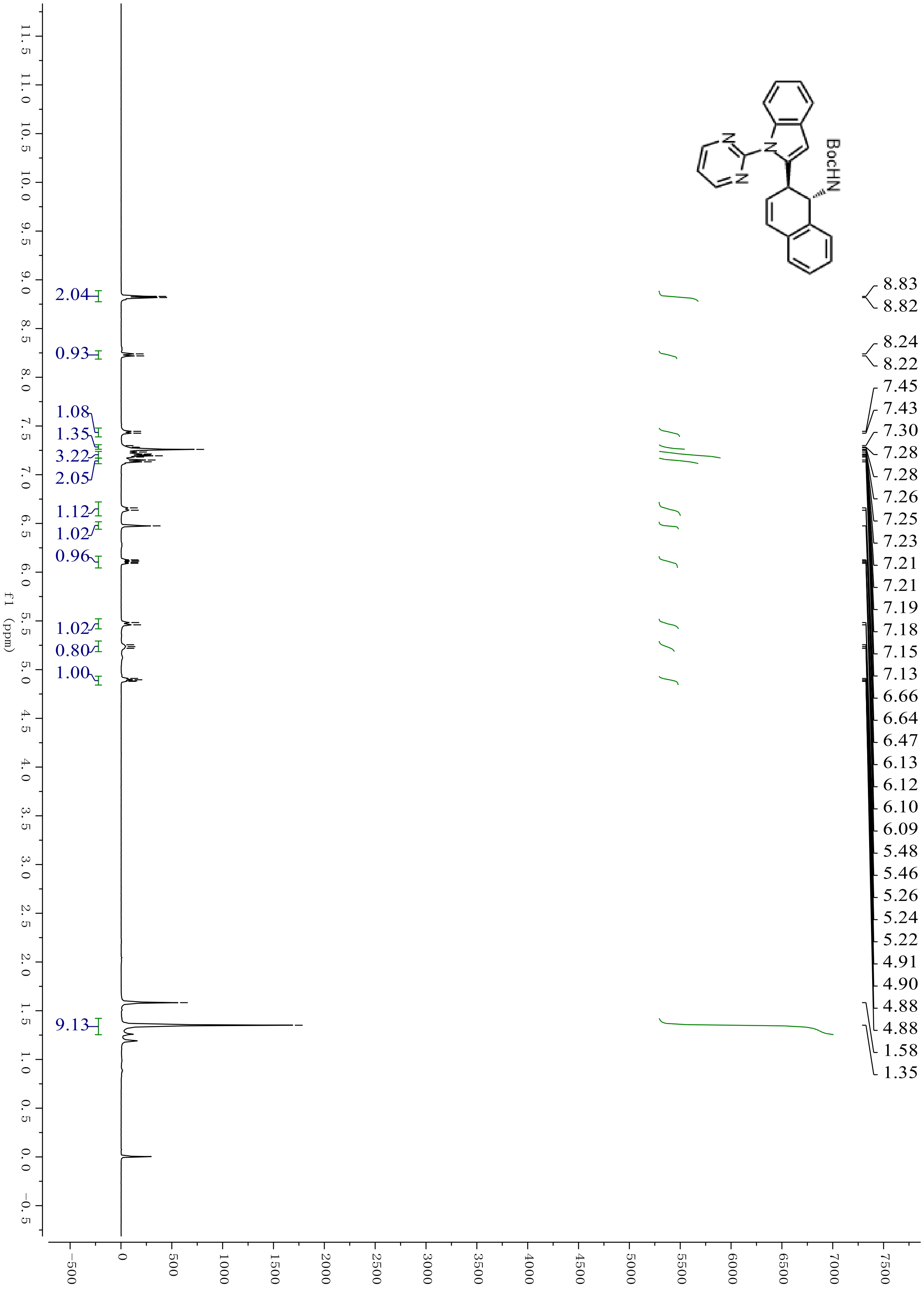

Supplementary Fig. 99 ^{13}C NMR Spectrum of *trans*-**7aa**

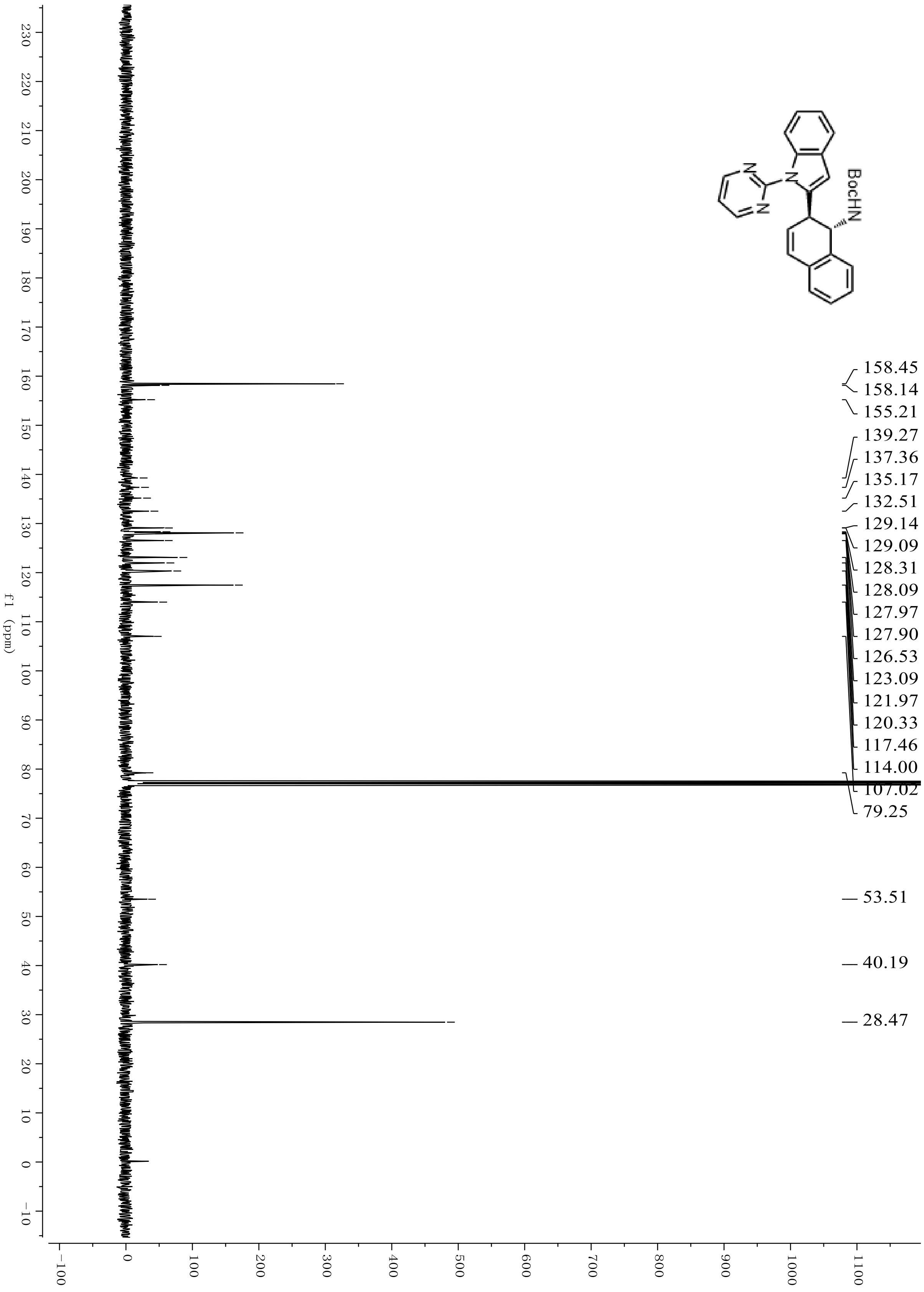

Supplementary Fig. 100 ^{1}H NMR Spectrum of *trans*-**7ab**

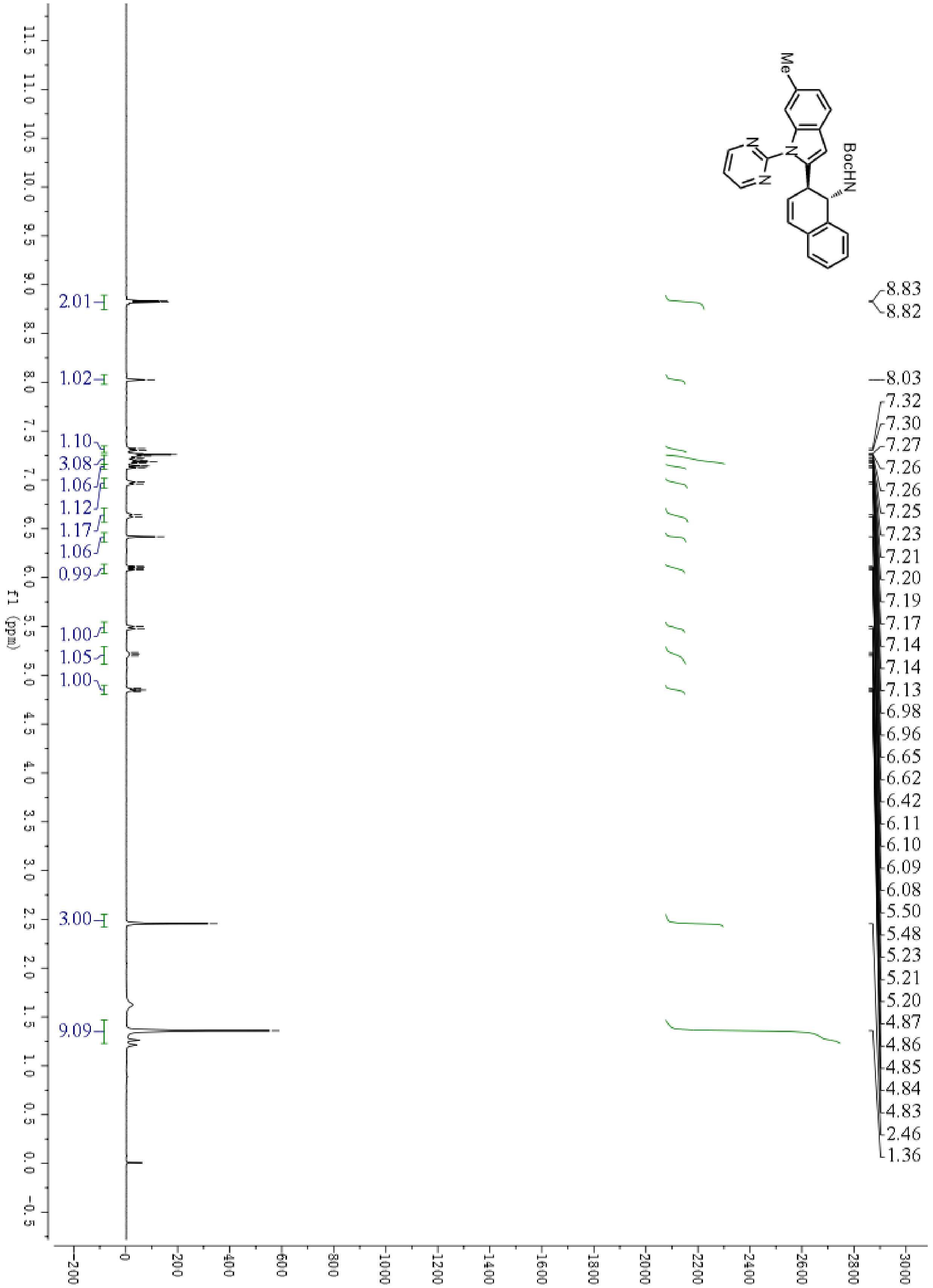

Supplementary Fig. 101 ^{13}C NMR Spectrum of *trans*-**7ab**

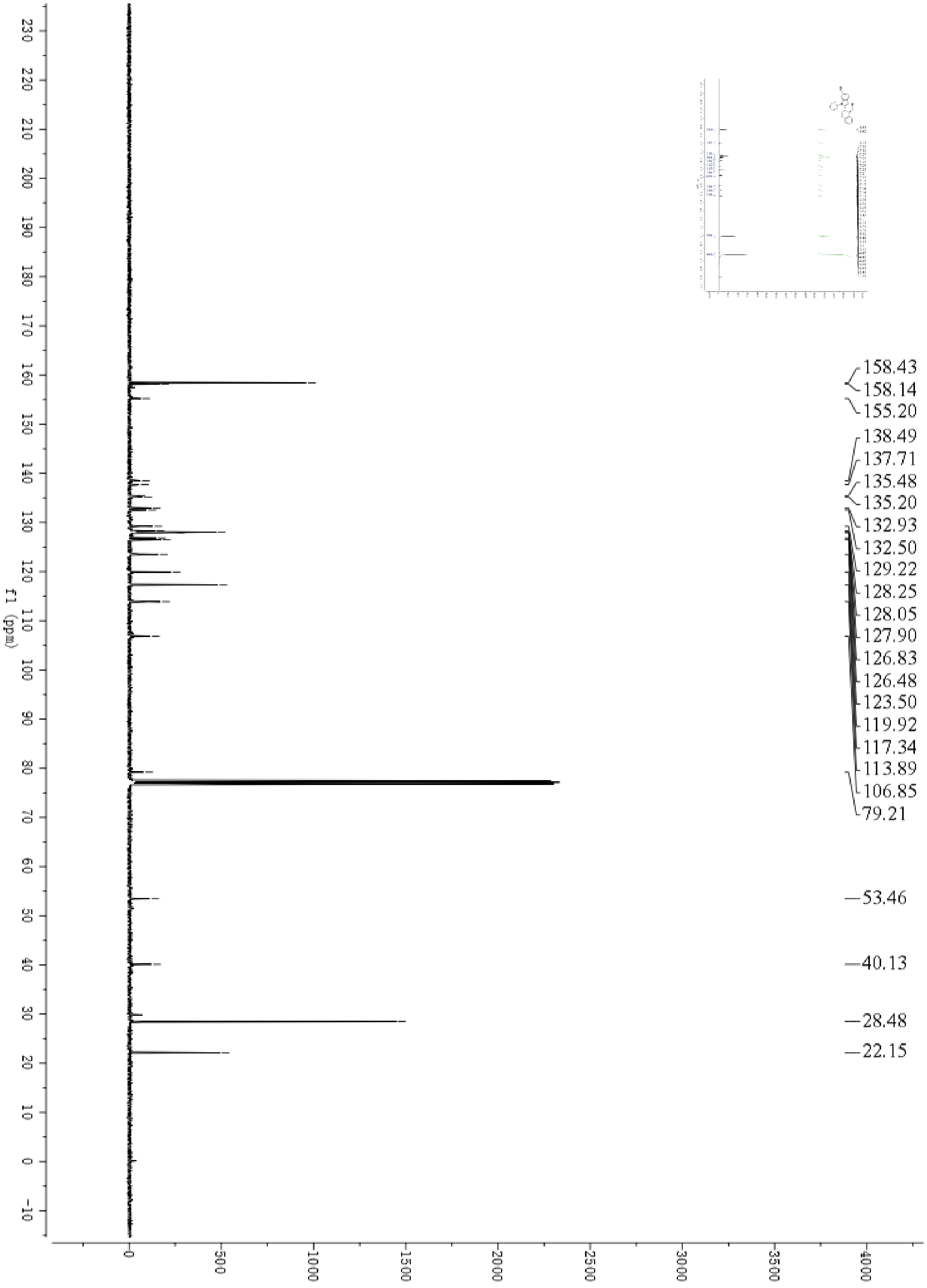

Supplementary Fig. 102 ^{1}H NMR Spectrum of *trans*-**7ab'**

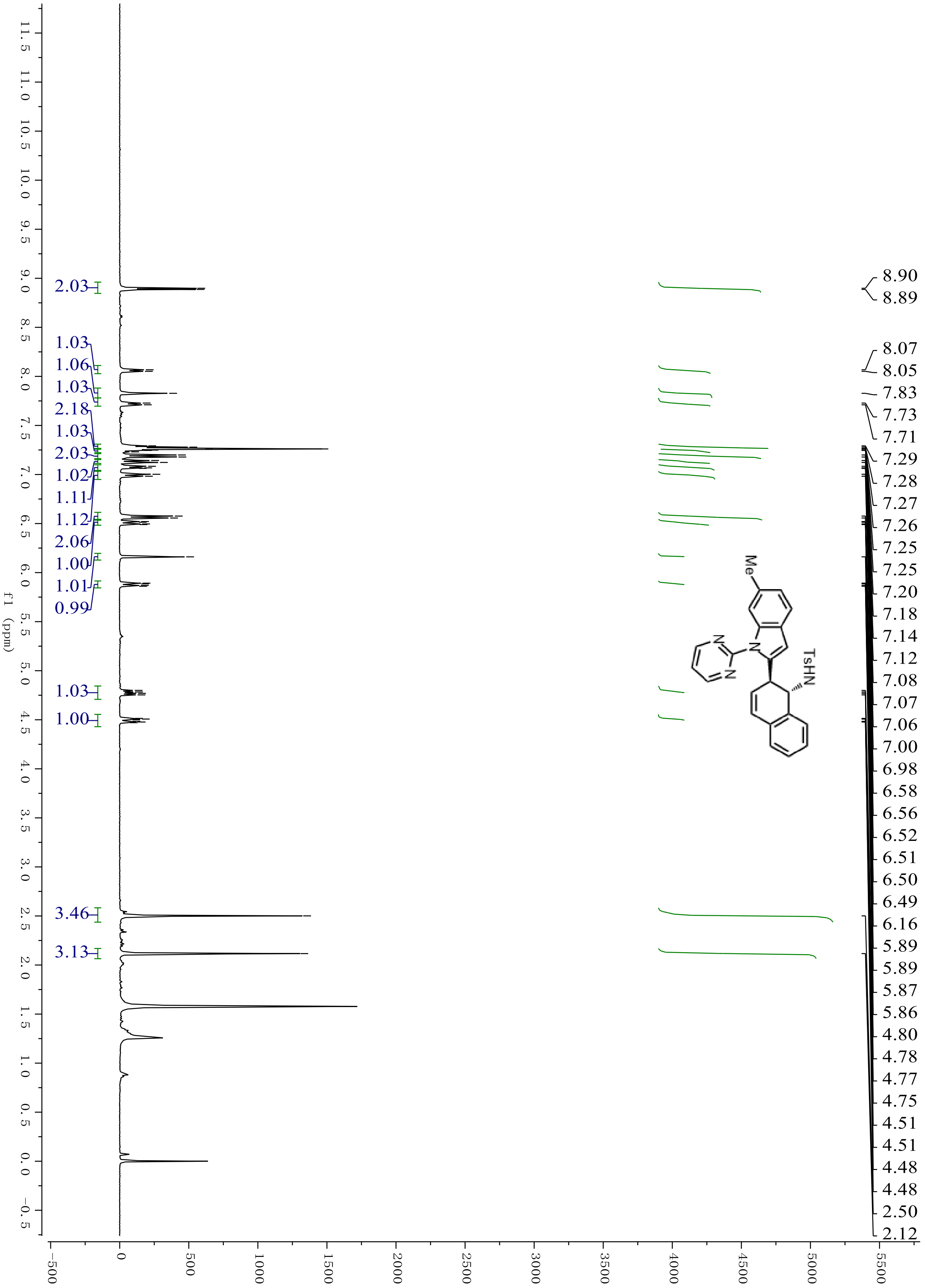

Supplementary Fig. 103 ^{13}C NMR Spectrum of *trans*-**7ab‘**

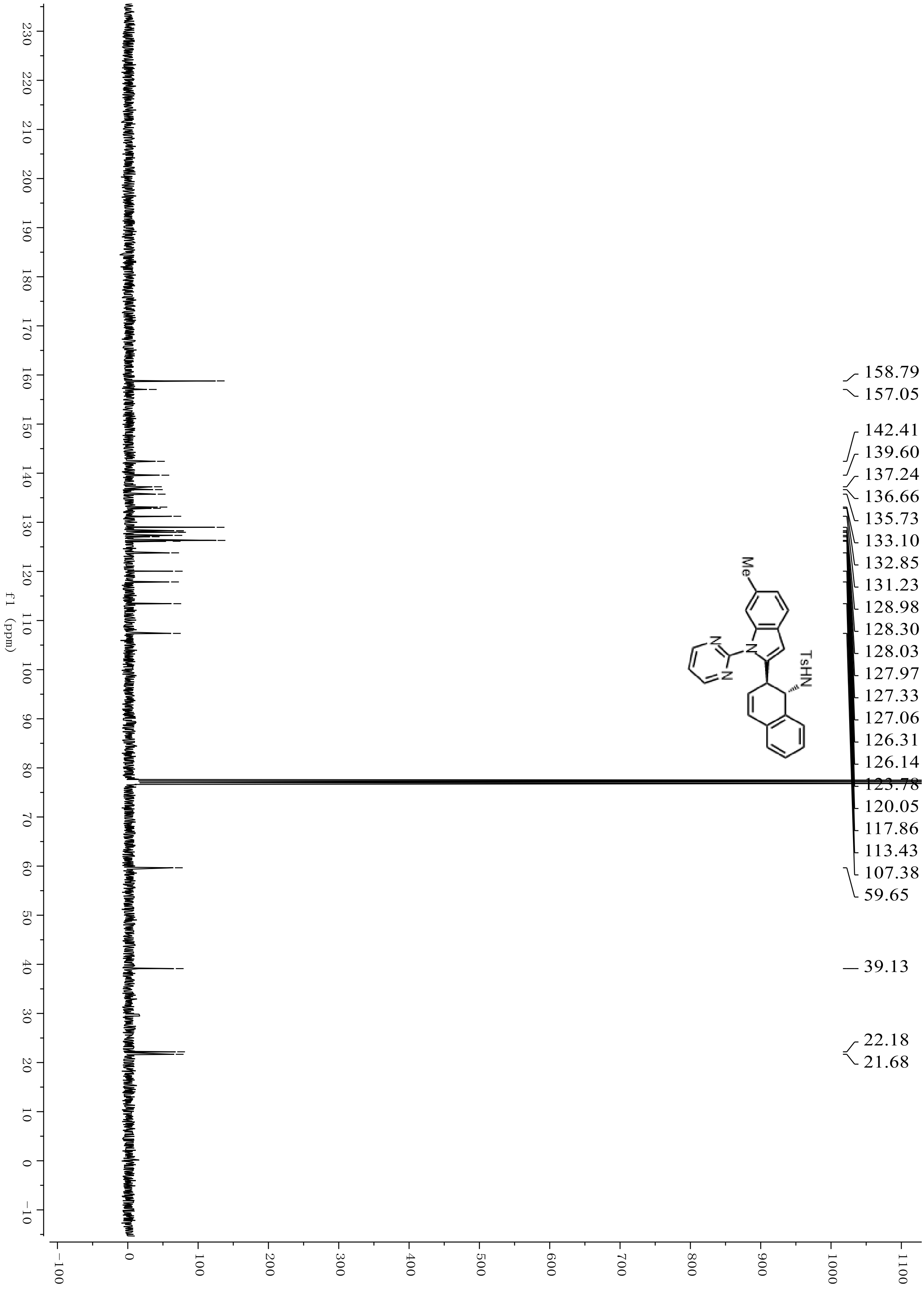

Supplementary Fig. 104 ^{1}H NMR Spectrum of *trans*-**7ac**

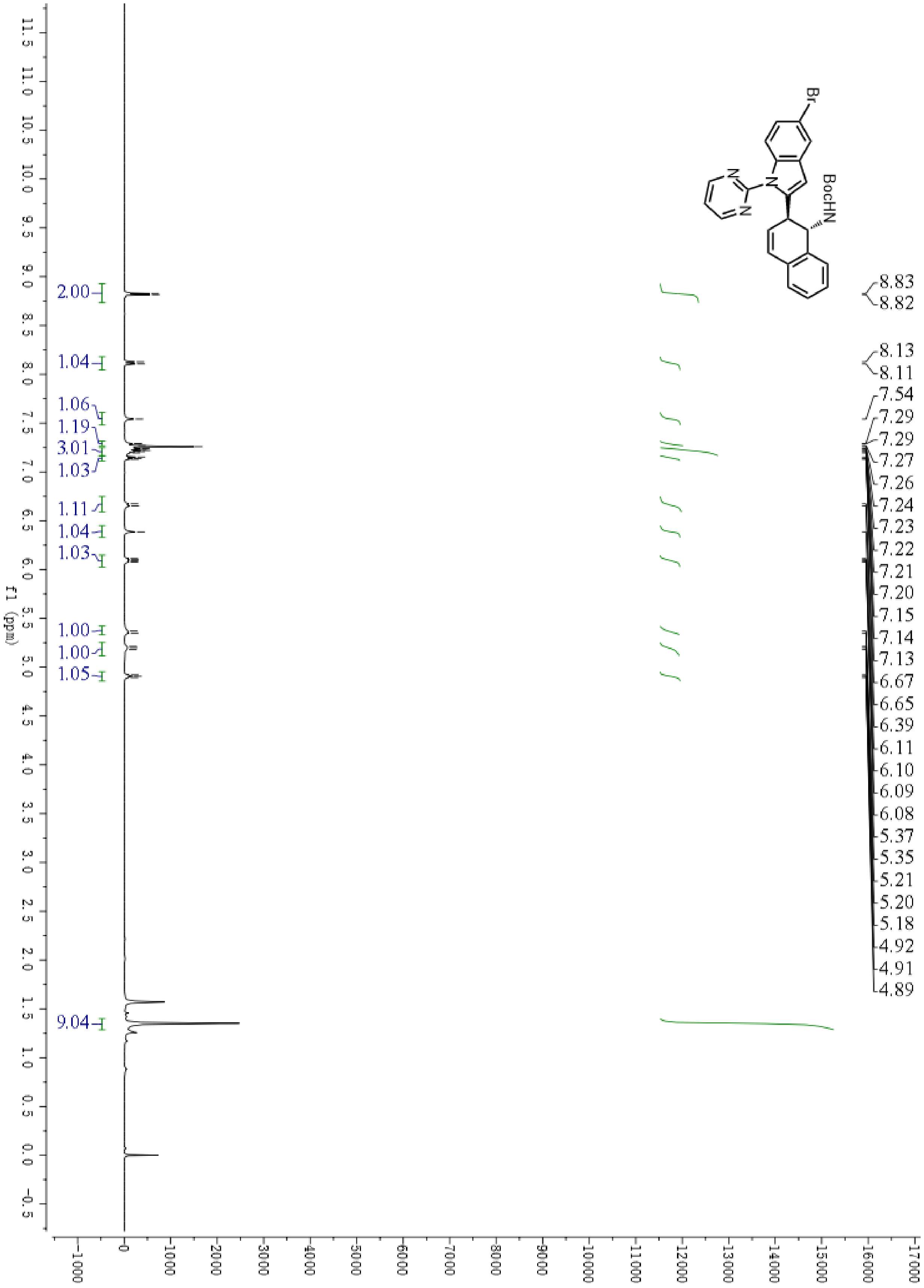

Supplementary Fig. 105 ^{13}C NMR Spectrum of *trans*-**7ac**

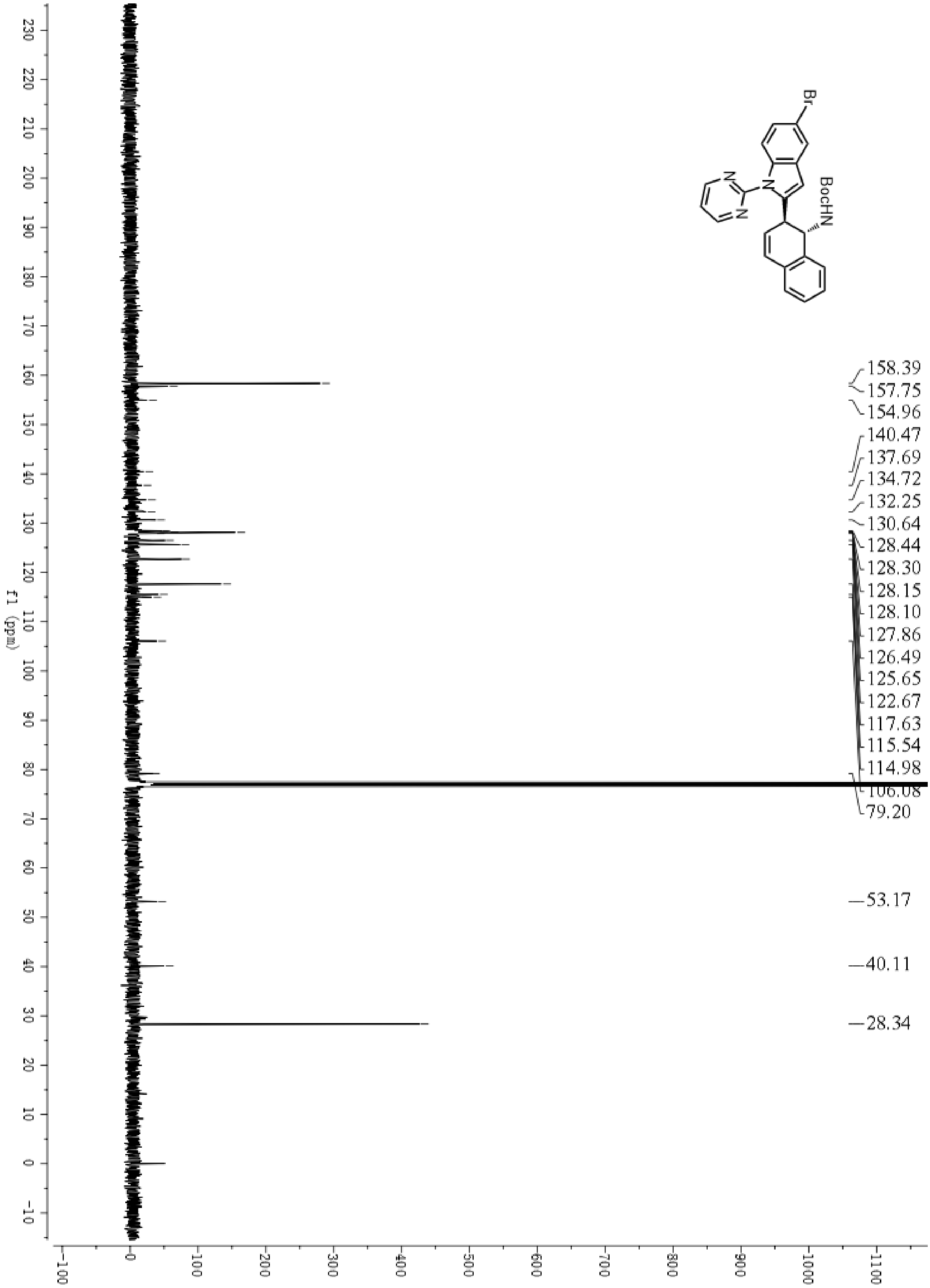

Supplementary Fig. 106 ^{1}H NMR Spectrum of *trans*-**7ad**

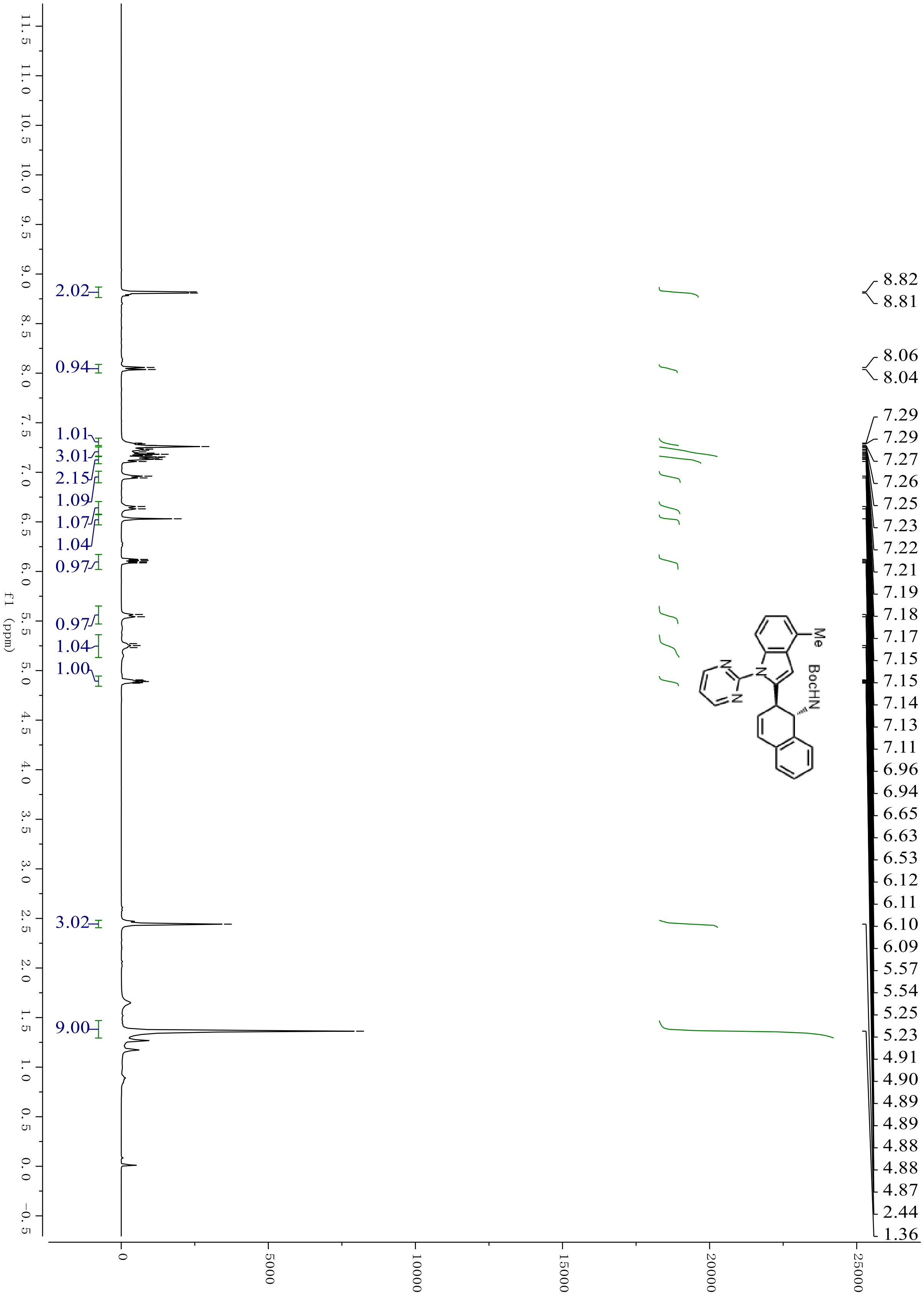

Supplementary Fig. 107 ^{13}C NMR Spectrum of *trans*-**7ad**

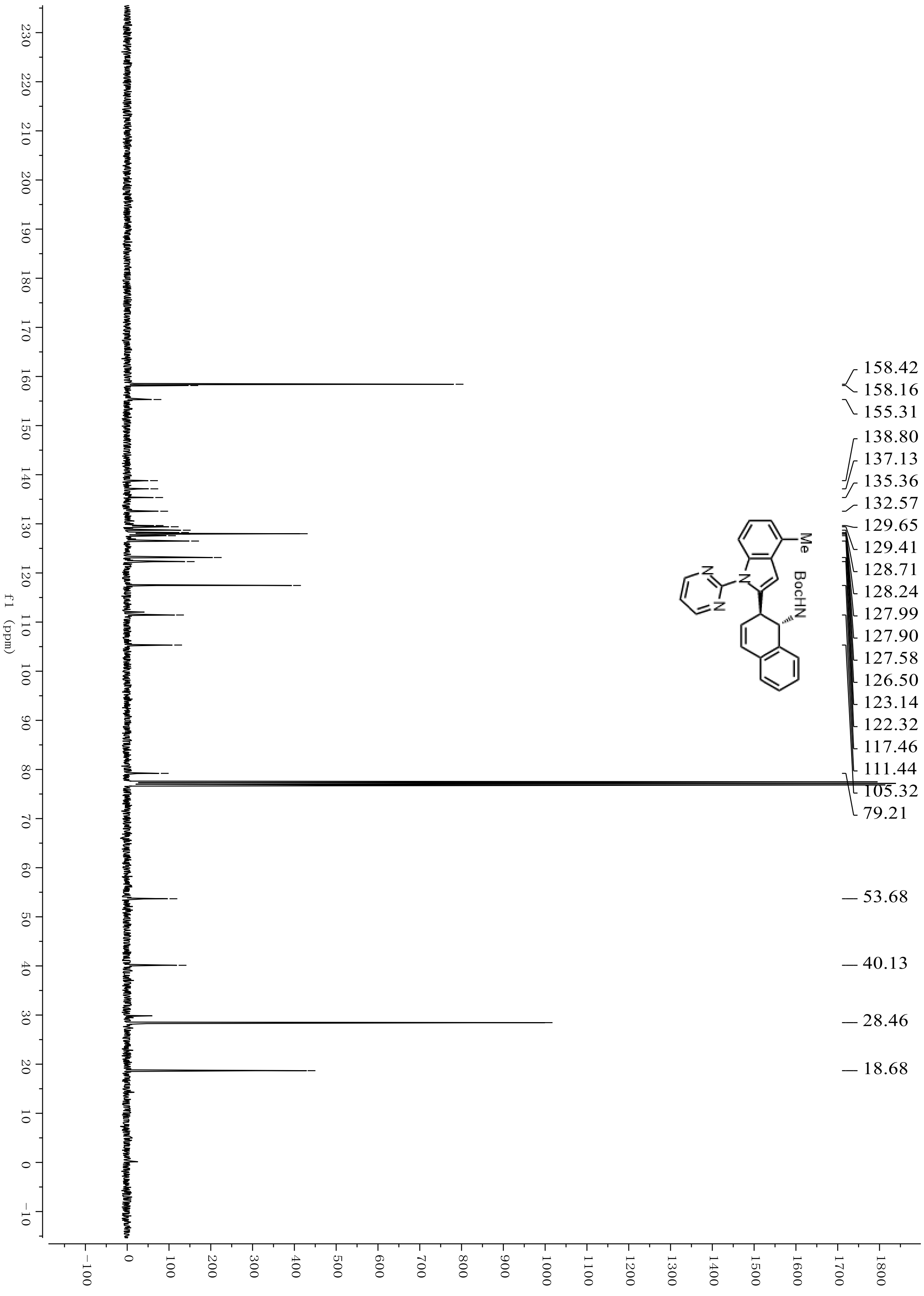

Supplementary Fig. 108 ^{1}H NMR Spectrum of *trans*-**7ae**

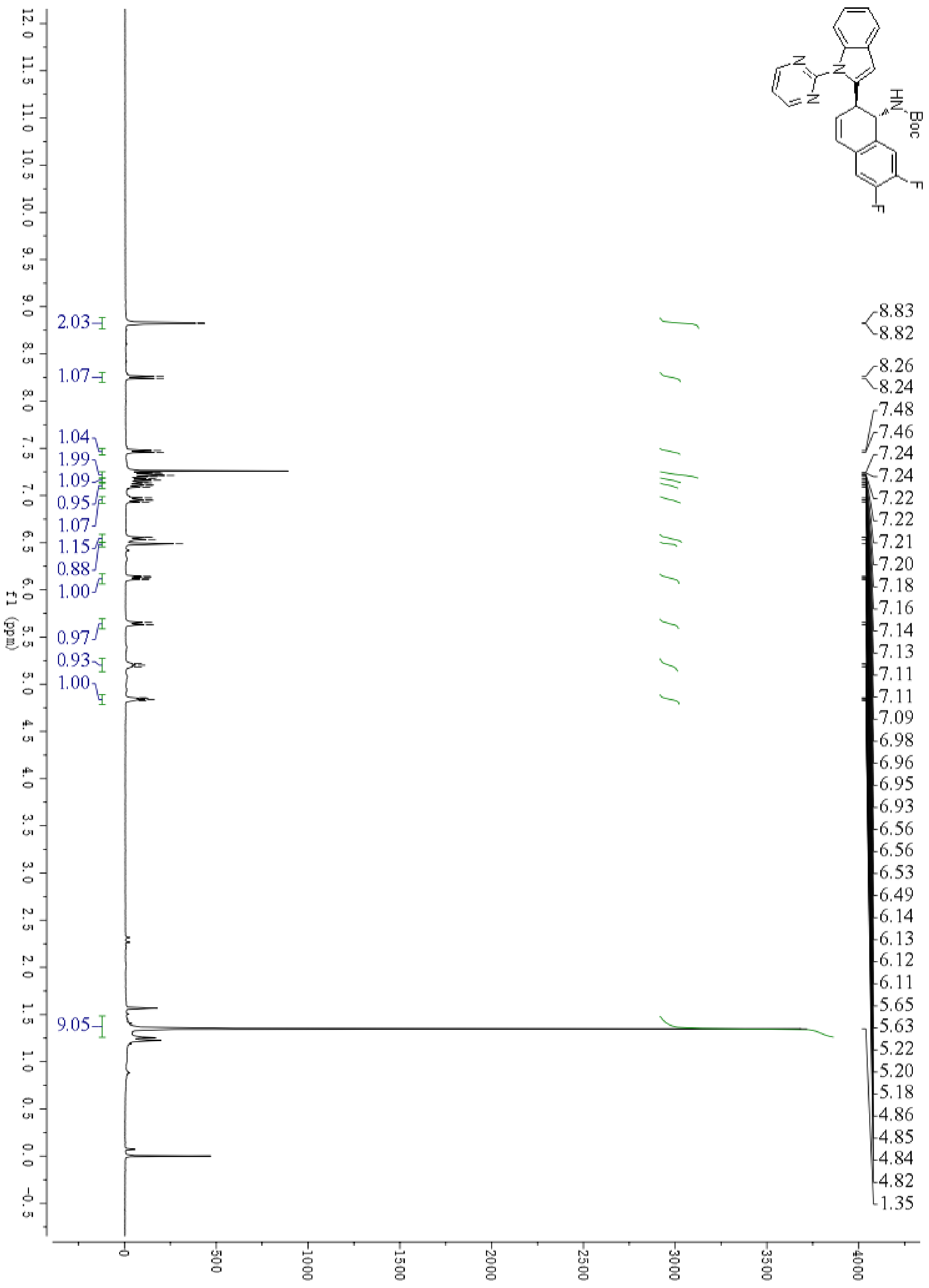

Supplementary Fig. 109 ^{13}C NMR Spectrum of *trans*-**7ae**

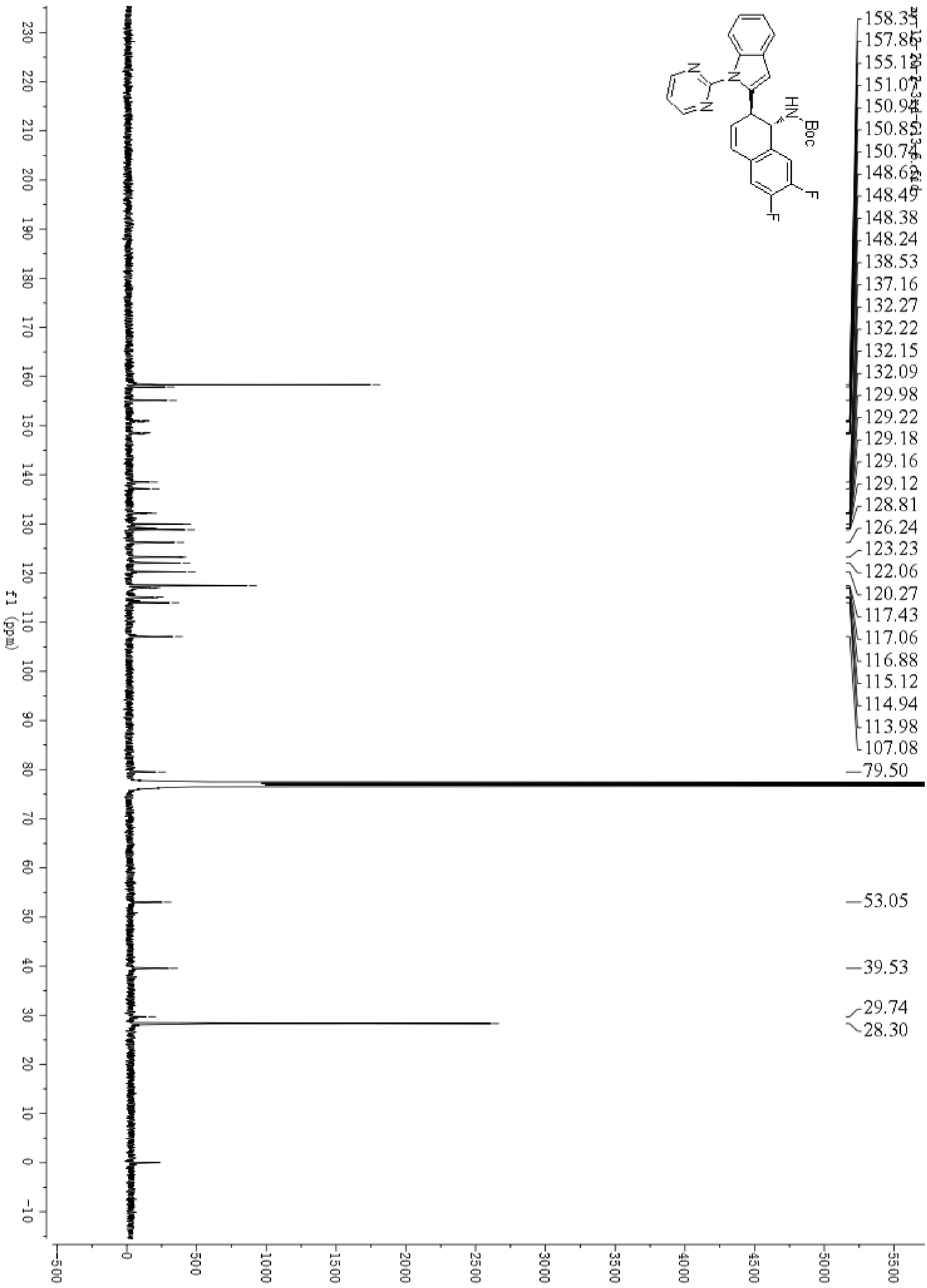

Supplementary Fig. 110 ^{19}F NMR Spectrum of *trans*-**7ae**

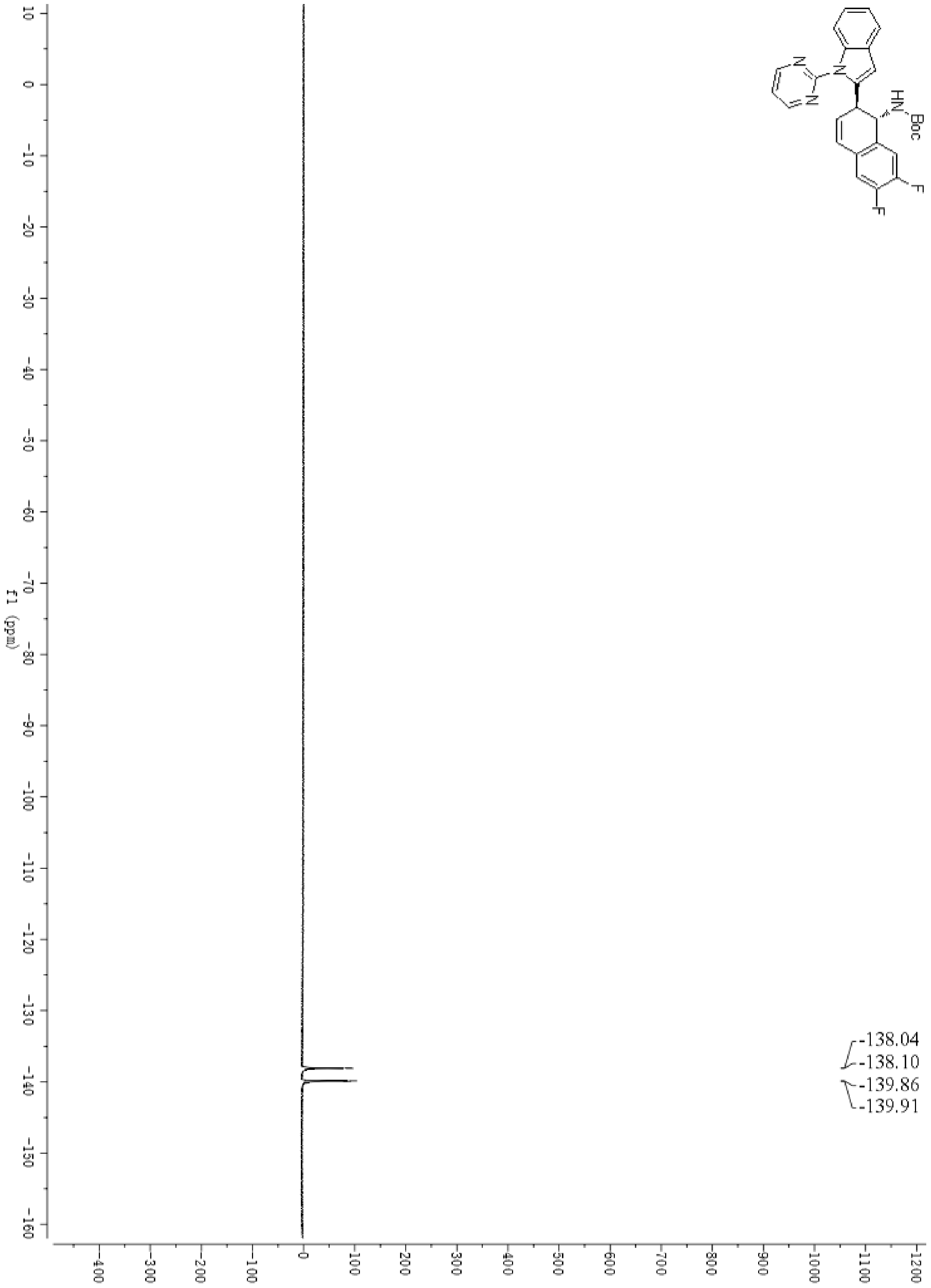

Supplementary Fig. 111 ^{1}H NMR Spectrum of *trans*-**7af**

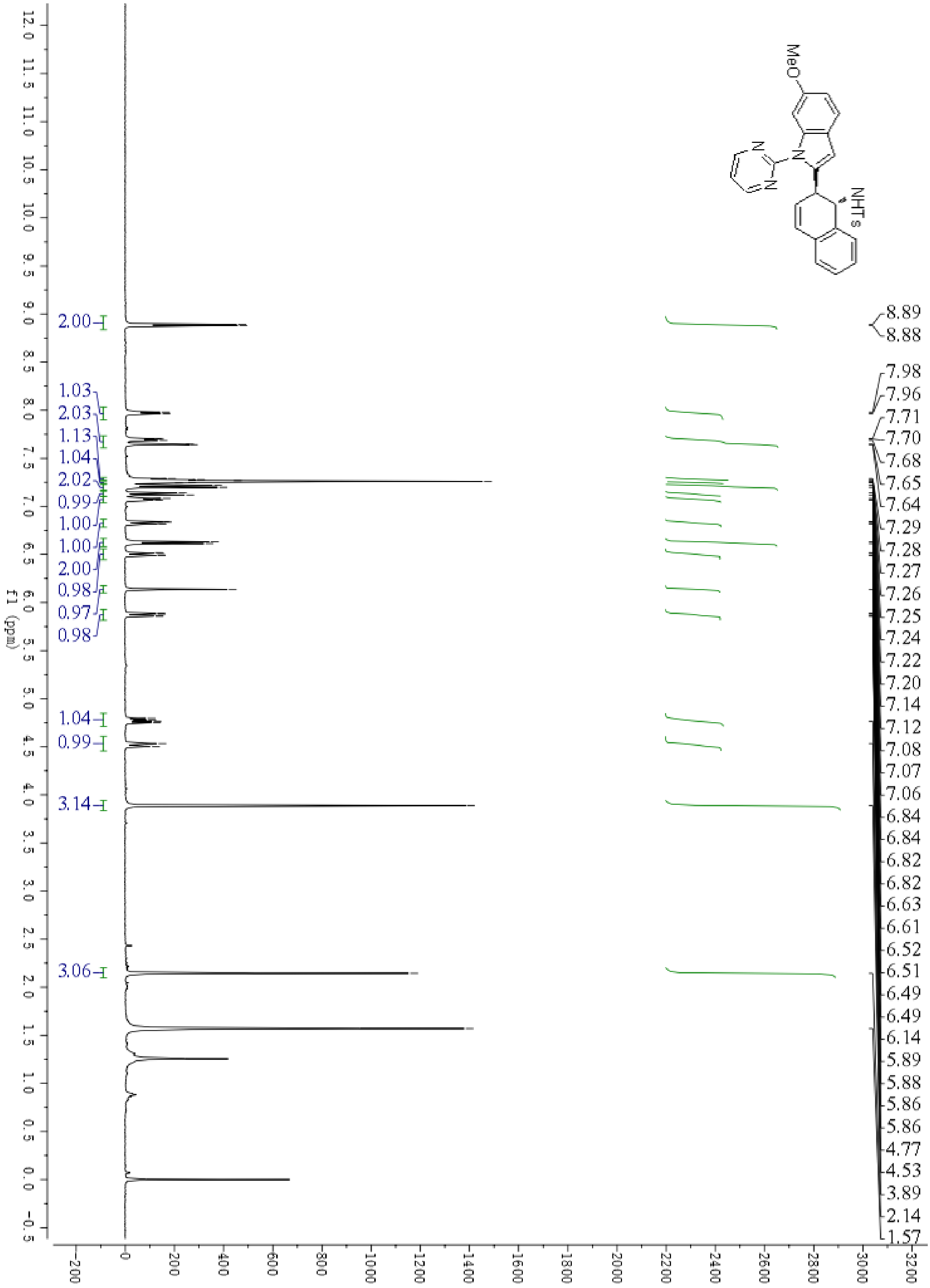

Supplementary Fig. 112 ^{13}C NMR Spectrum of *trans*-**7af**

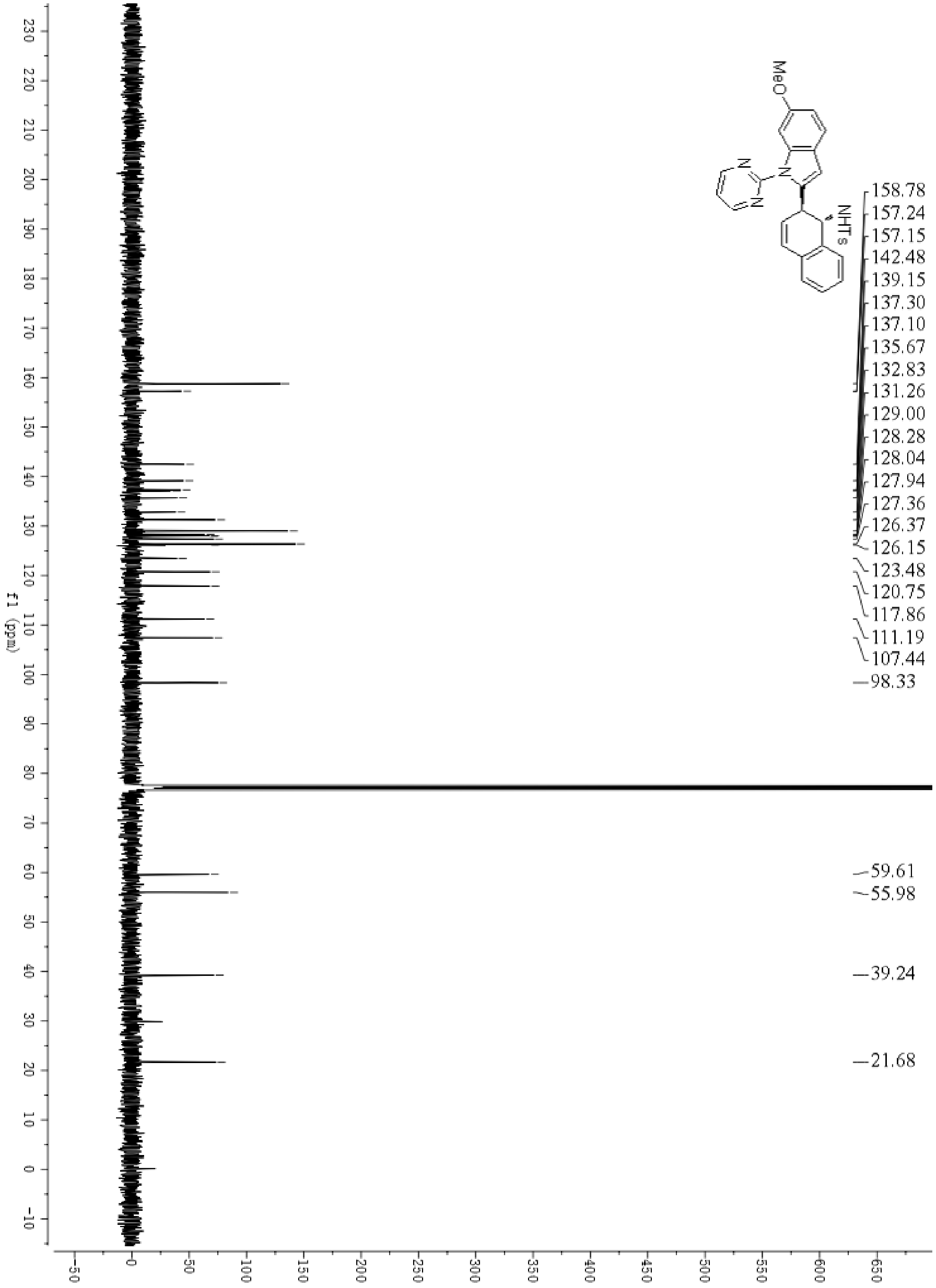

Supplementary Fig. 113 ^{1}H NMR Spectrum of **4**

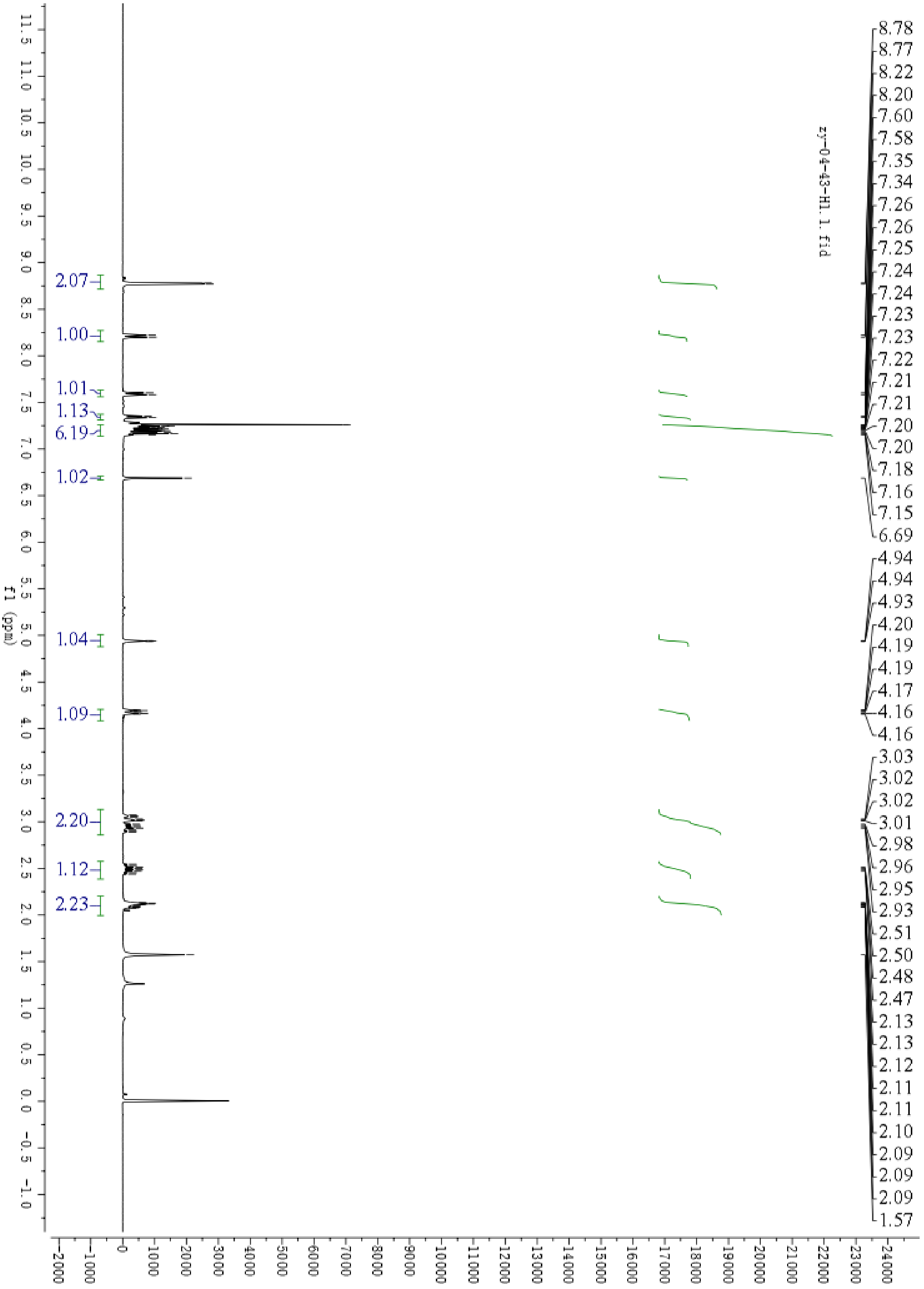

Supplementary Fig. 114 ^{13}C NMR Spectrum of **4**

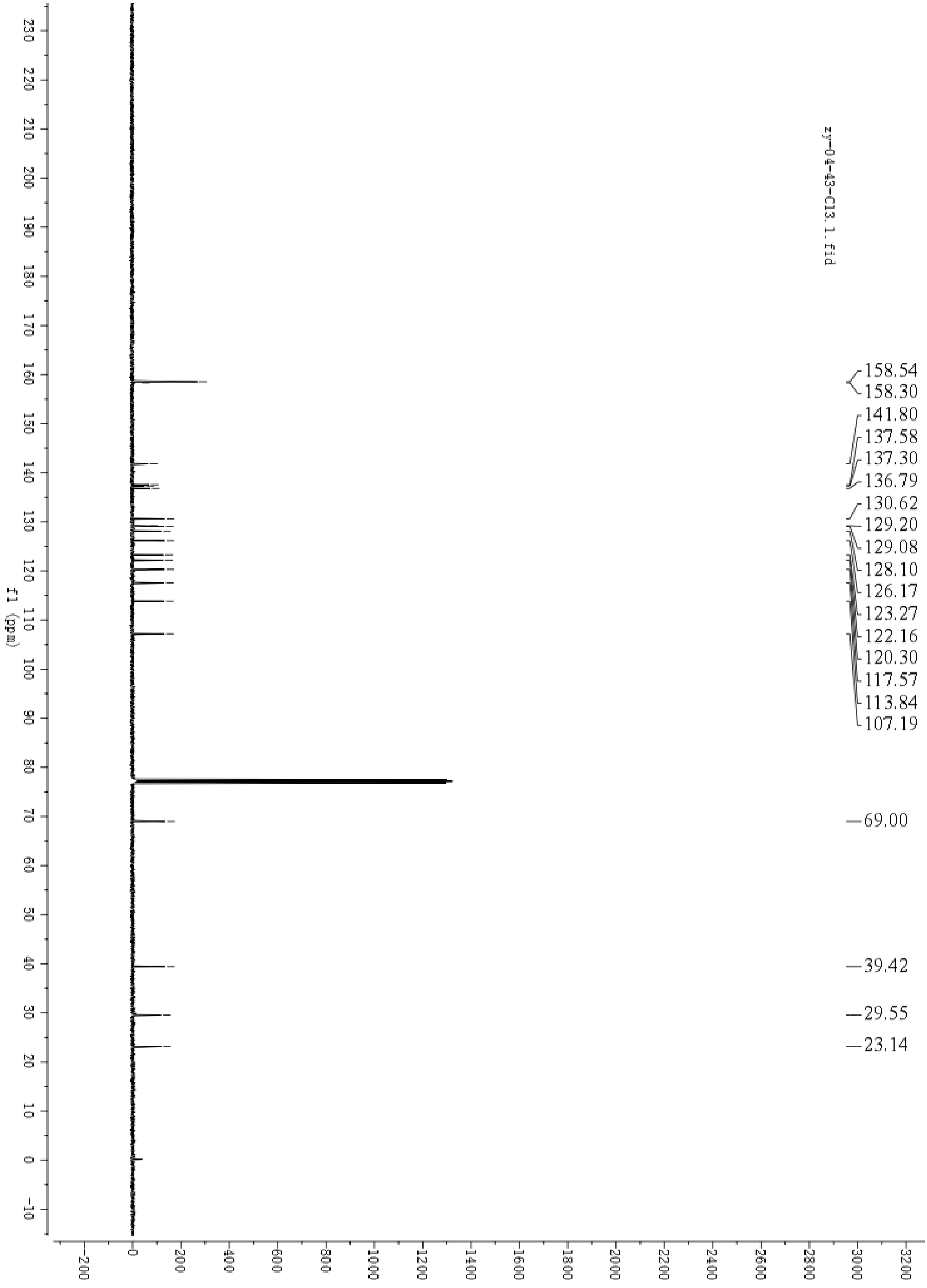

Supplementary Fig. 115 ^{1}H NMR Spectrum of **5**

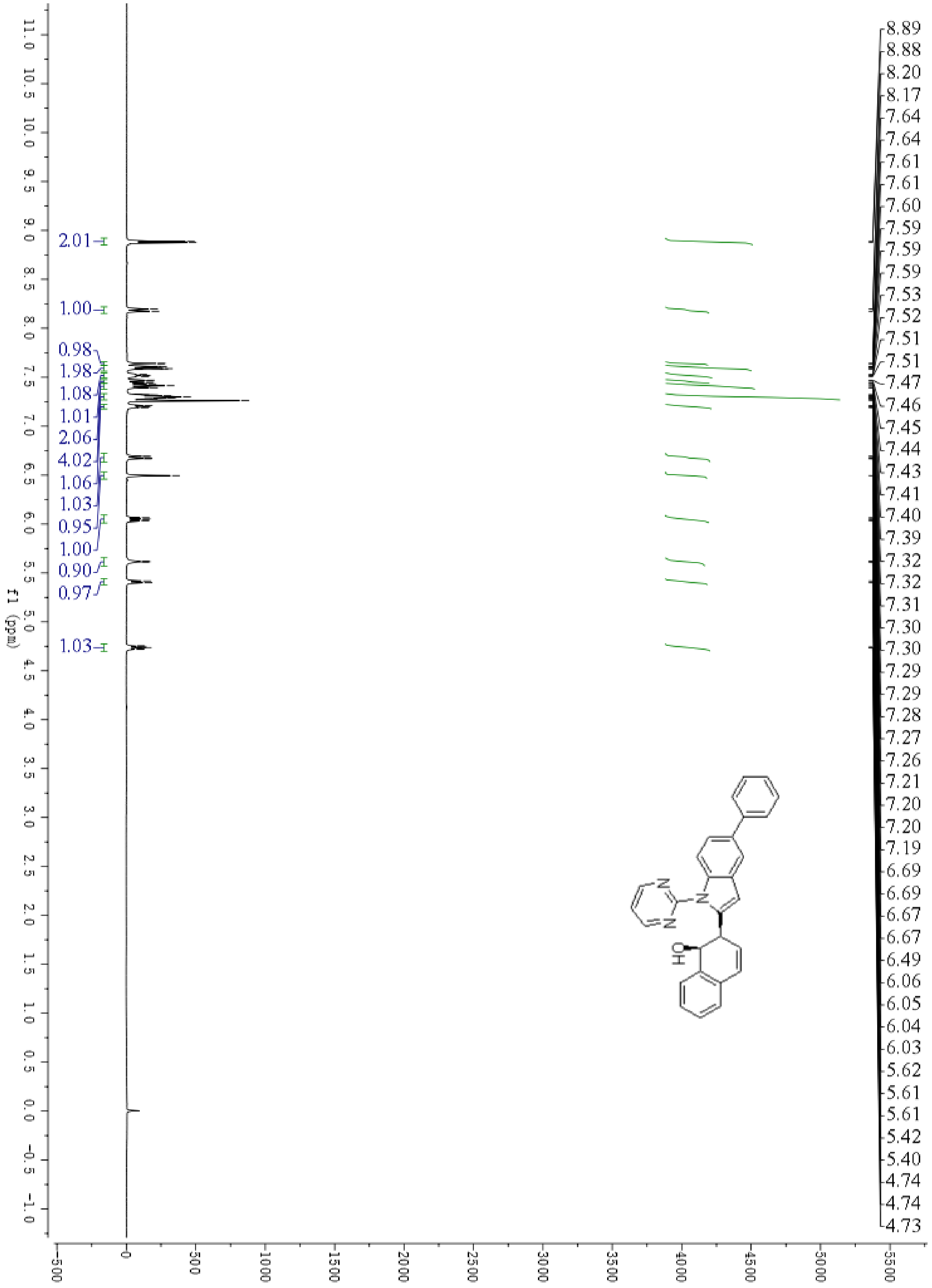

Supplementary Fig. 116 ^{13}C NMR Spectrum of **5**

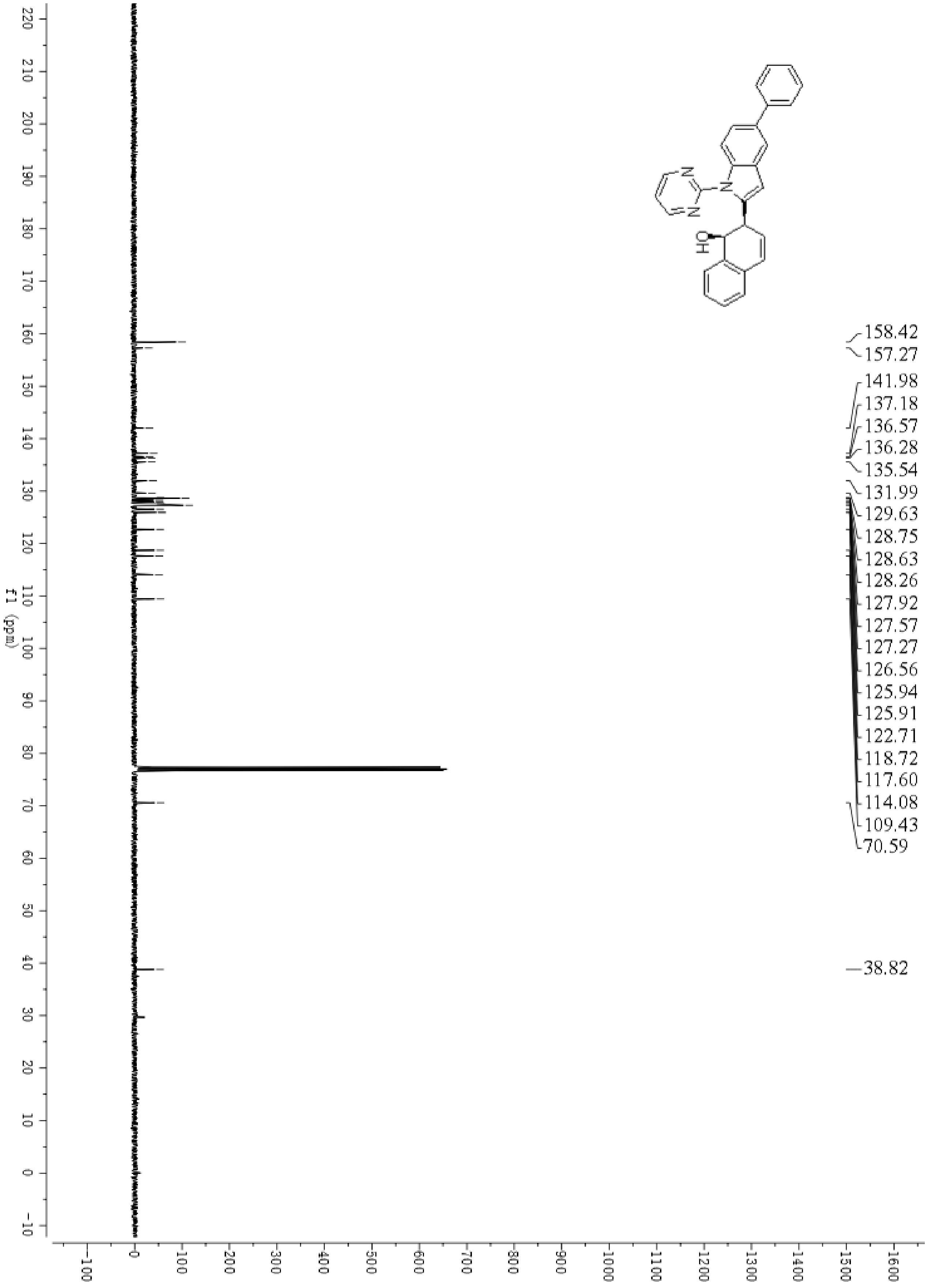

Supplementary Fig. 117 ^{1}H NMR Spectrum of **8**

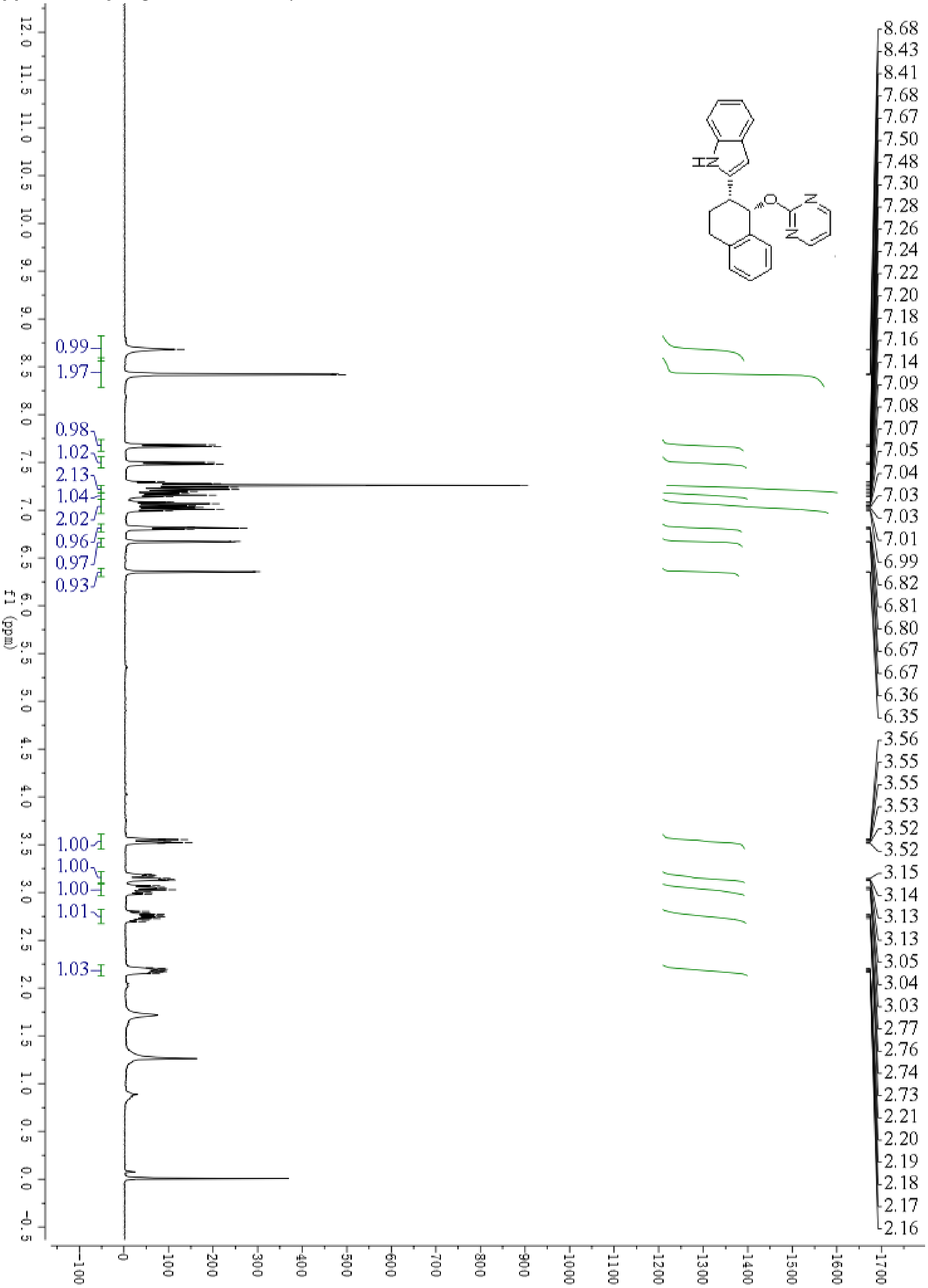

Supplementary Fig. 118 ^{1}H NMR Spectrum of **8**

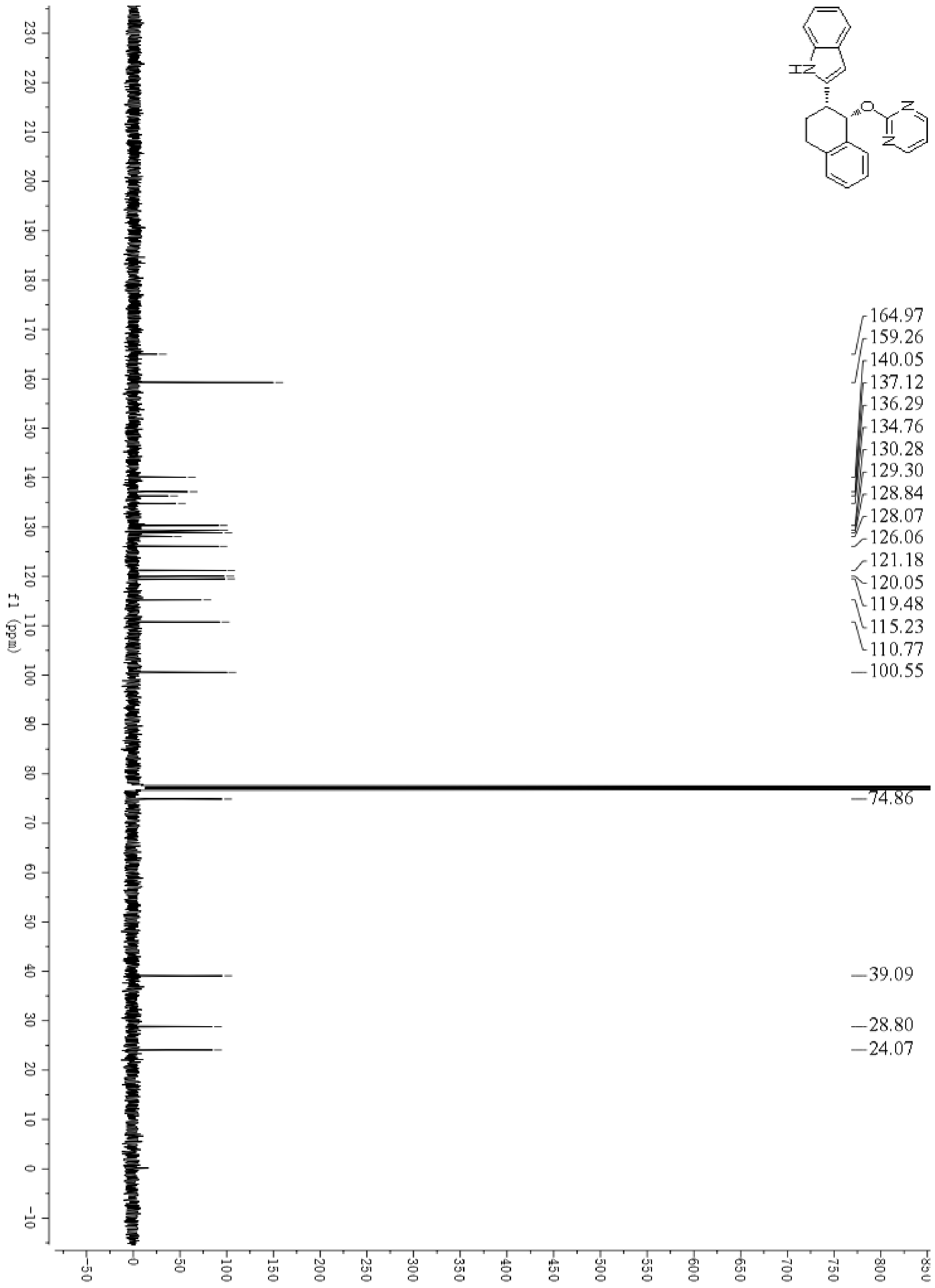

1.10 Copies of HPLC chromatograms

Supplementary Fig. 119 HPLC analysis of *cis*-**3aa**

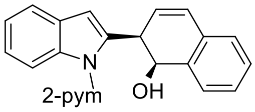

3aa

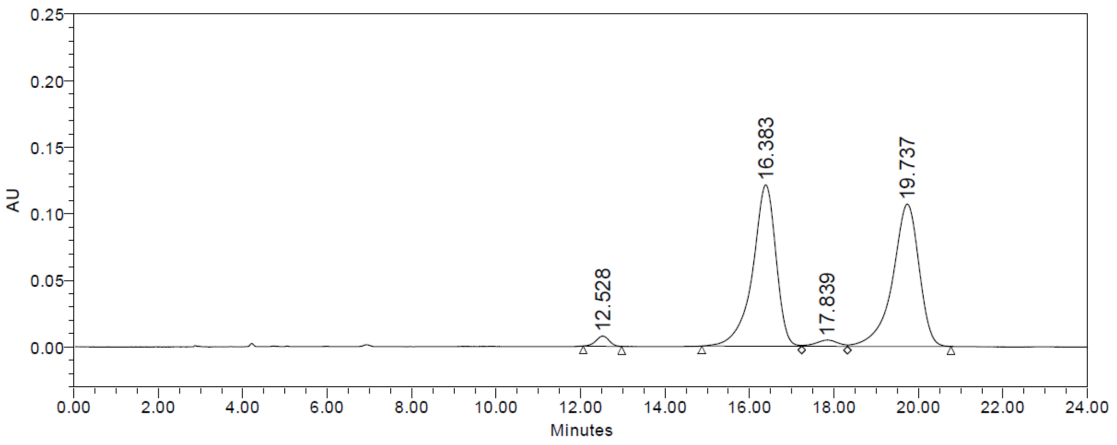

	RT	Area	% Area	Height
1	12.528	164511	1.70	7621
2	16.383	4669008	48.33	121259
3	17.839	167370	1.73	4703
4	19.737	4659366	48.23	106898

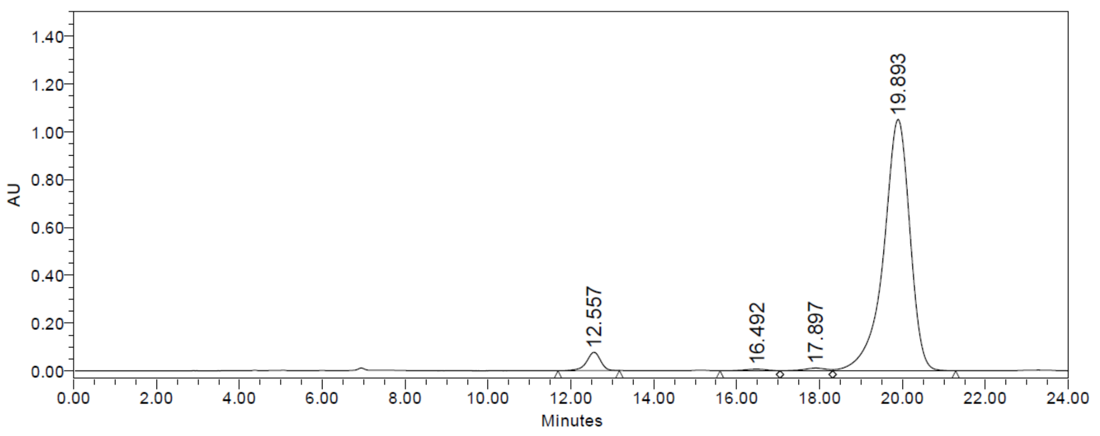

	RT	Area	% Area	Height
1	12.557	1789953	3.67	76744
2	16.492	239879	0.49	6756
3	17.897	401542	0.82	10984
4	19.893	46301329	95.01	1049900

Supplementary Fig. 120 HPLC analysis of *cis*-**3ba**

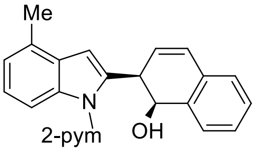

3ba

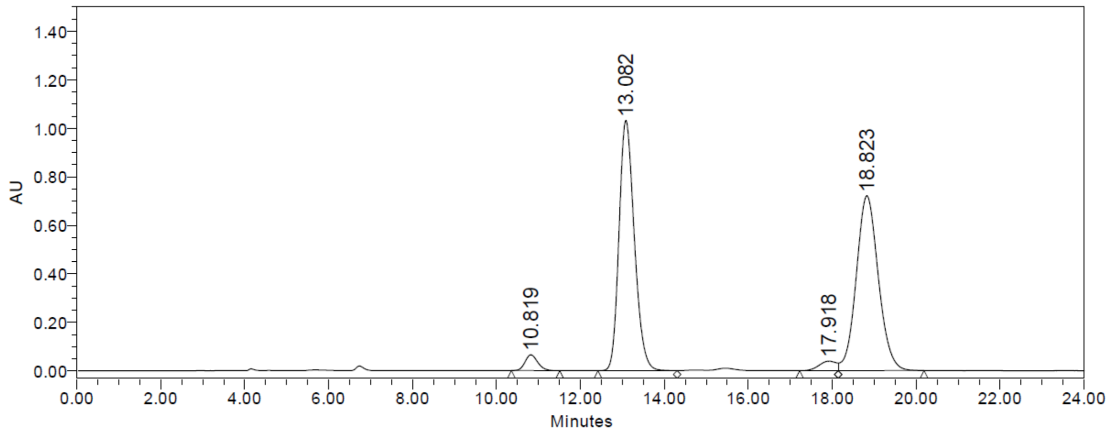

	RT	Area	% Area	Height
1	10.819	1359753	2.47	65036
2	13.082	26184744	47.56	1032066
3	17.918	1099551	2.00	39086
4	18.823	26409723	47.97	720736

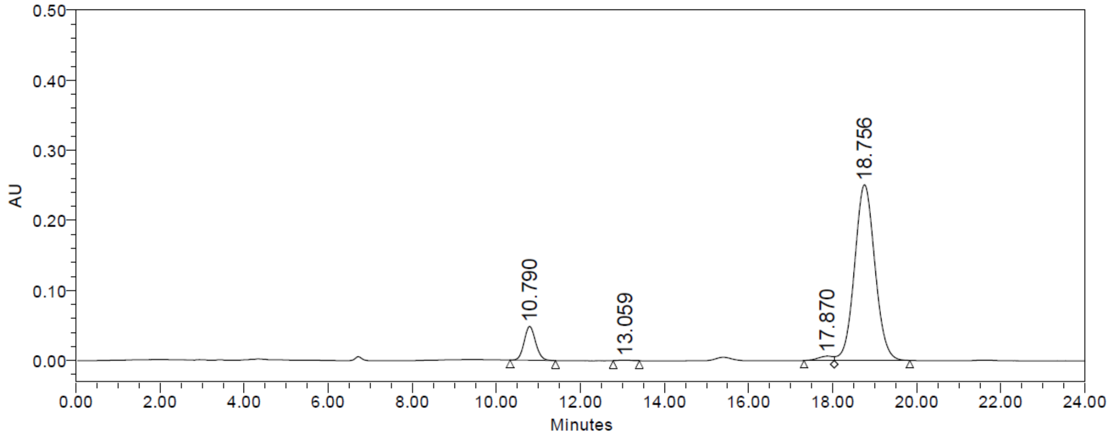

	RT	Area	% Area	Height
1	10.790	917364	9.62	48348
2	13.059	17440	0.18	867
3	17.870	150388	1.58	6116
4	18.756	8445888	88.61	250853

Supplementary Fig. 121 HPLC analysis of *cis*-**3ca**

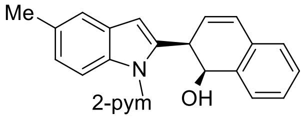

3ca

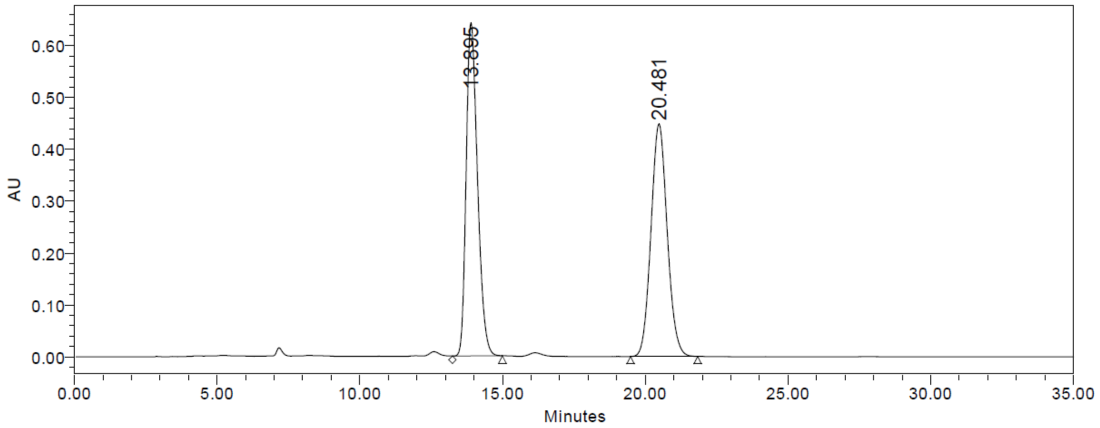

	RT	Area	% Area	Height
1	13.895	17699636	49.99	642417
2	20.481	17708740	50.01	448136

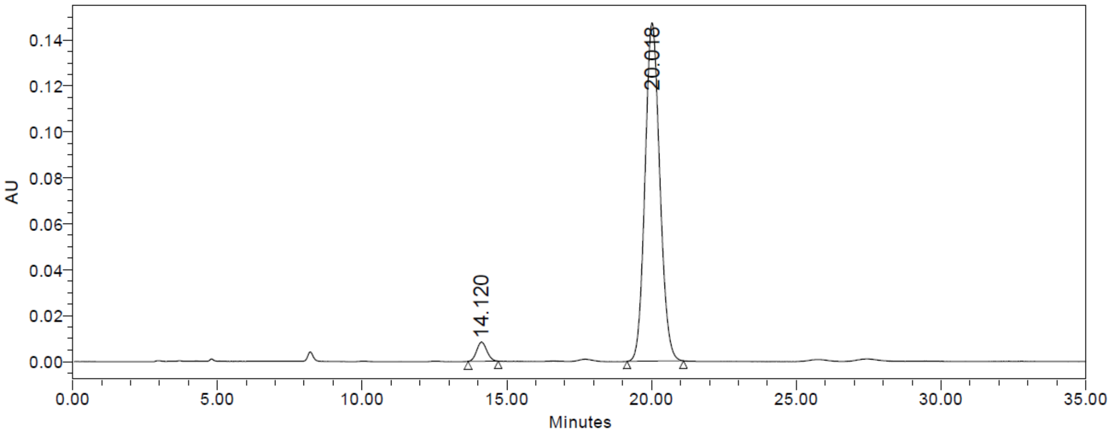

	RT	Area	% Area	Height
1	14.120	204130	3.76	8368
2	20.018	5227396	96.24	147302

Supplementary Fig. 122 HPLC analysis of *cis*-**3da**

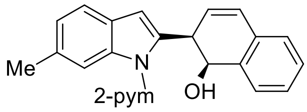

3da

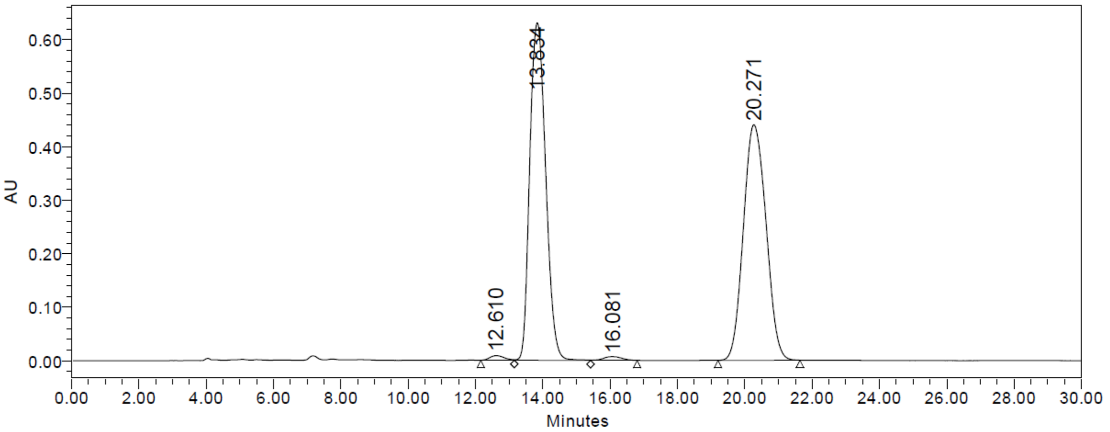

	RT	Area	% Area	Height
1	12.610	253084	0.61	8571
2	13.834	20690578	49.52	630612
3	16.081	259659	0.62	7004
4	20.271	20576228	49.25	440116

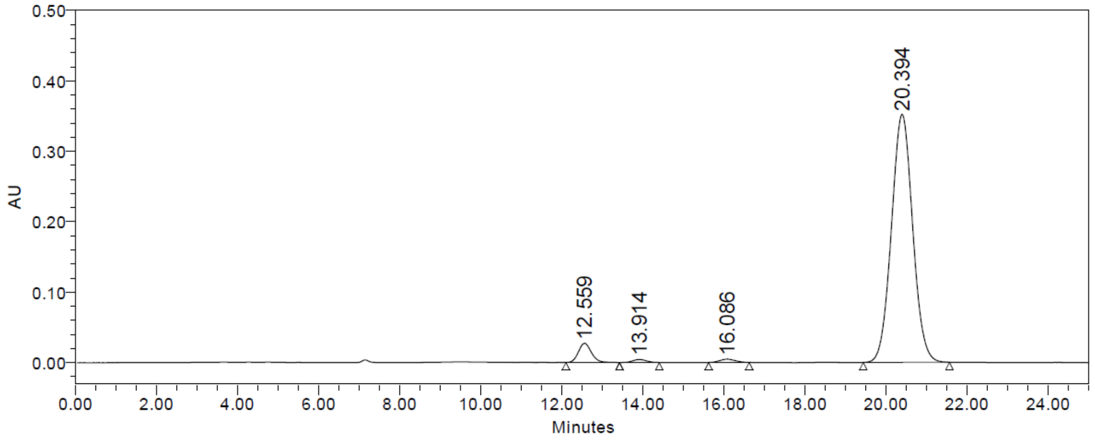

	RT	Area	% Area	Height
1	12.559	608020	4.45	27280
2	13.914	101711	0.74	4318
3	16.086	123669	0.91	4586
4	20.394	12828184	93.90	352170

Supplementary Fig. 123 HPLC analysis of *cis*-**3ea**

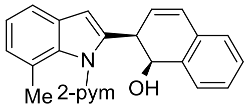

3ea

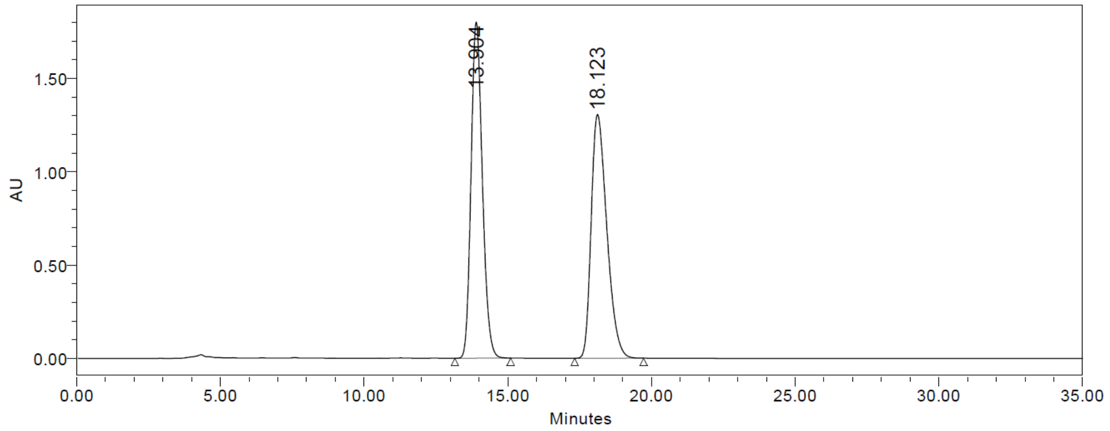

	RT	Area	% Area	Height
1	13.904	49183831	49.92	1801061
2	18.123	49338175	50.08	1306853

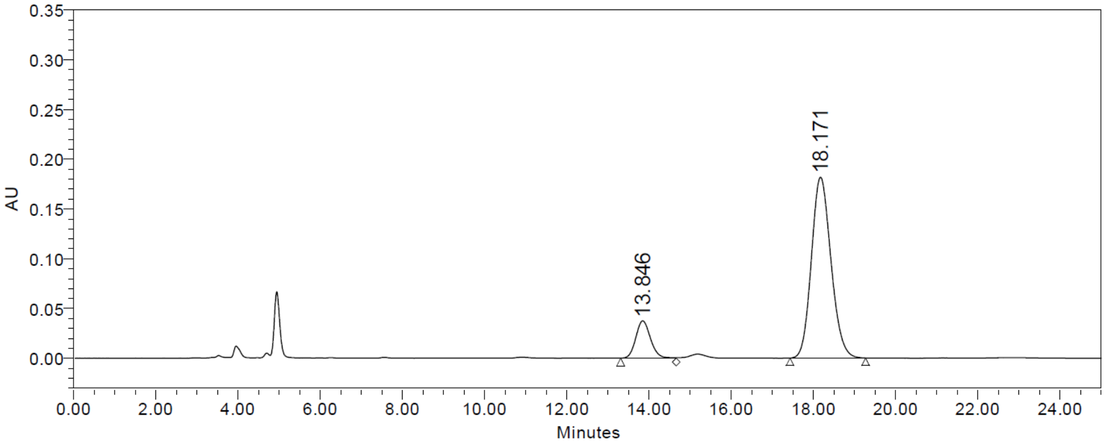

	RT	Area	% Area	Height
1	13.846	925167	13.36	37519
2	18.171	6000979	86.64	181751

Supplementary Fig. 124 HPLC analysis of *cis*-**3fa**

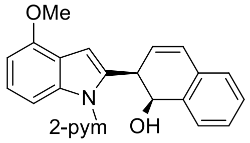

3fa

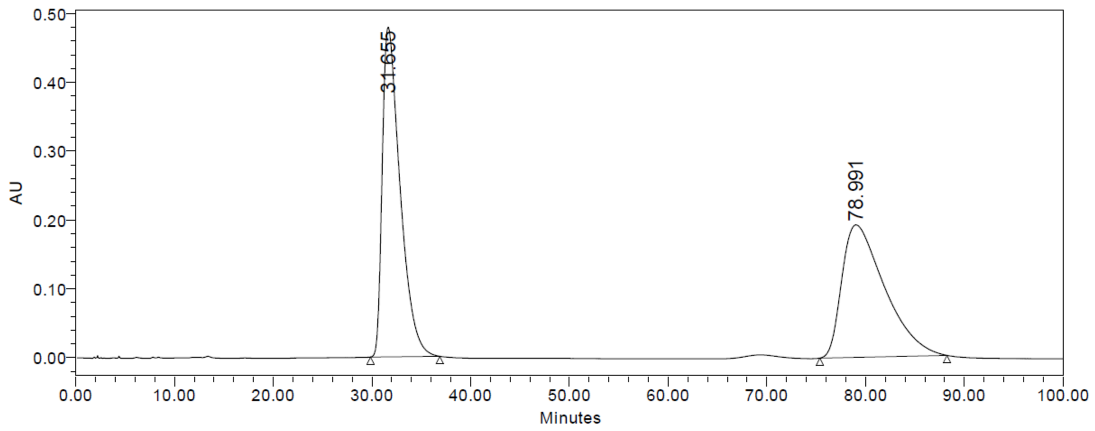

	RT	Area	% Area	Height
1	31.655	60006726	50.88	478889
2	78.991	57940768	49.12	192258

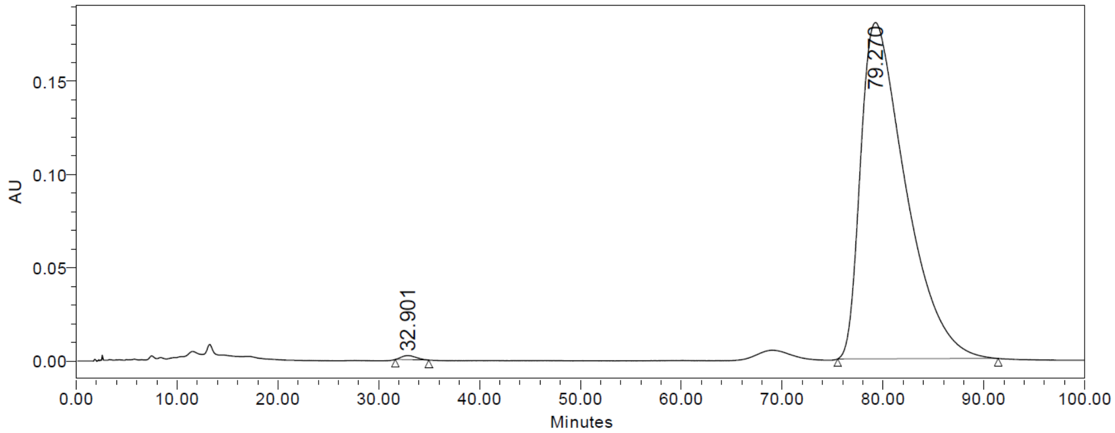

	RT	Area	% Area	Height
1	32.901	221415	0.39	2190
2	79.270	55964247	99.61	180258

Supplementary Fig. 125 HPLC analysis of *cis*-**3ga**

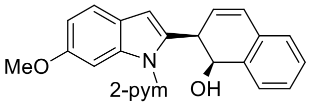

3ga

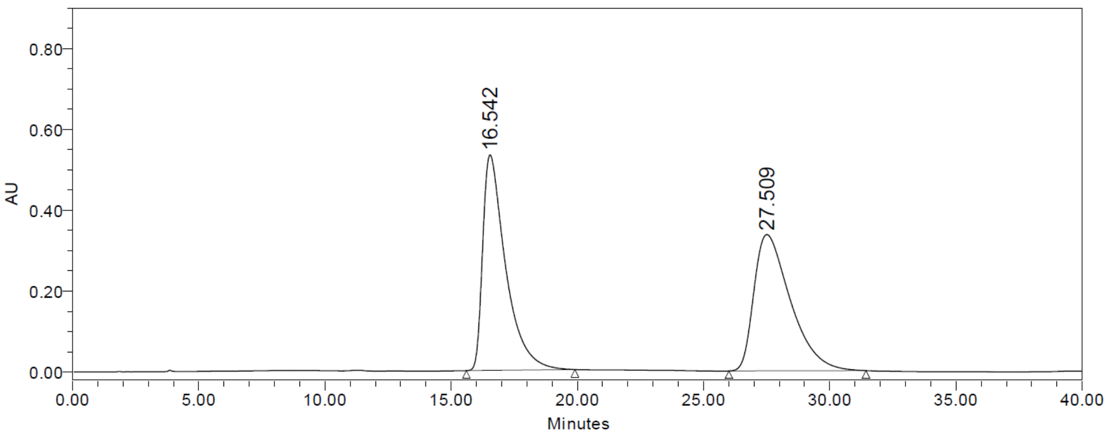

	RT	Area	% Area	Height
1	16.542	33653829	49.79	533136
2	27.509	33940912	50.21	337198

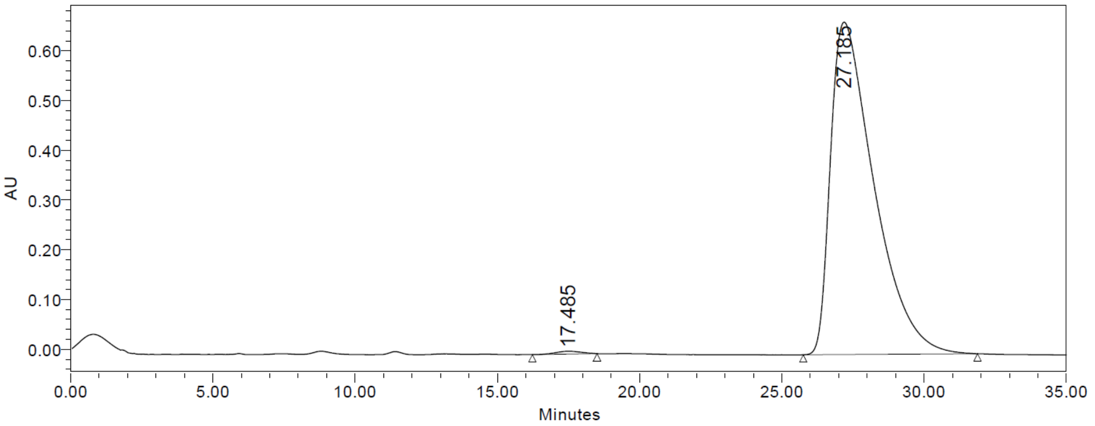

	RT	Area	% Area	Height
1	17.485	343936	0.48	5557
2	27.185	70984927	99.52	667991

Supplementary Fig. 126 HPLC analysis of *cis*-**3ha**

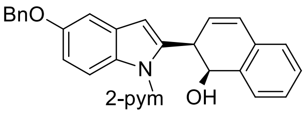

3ha

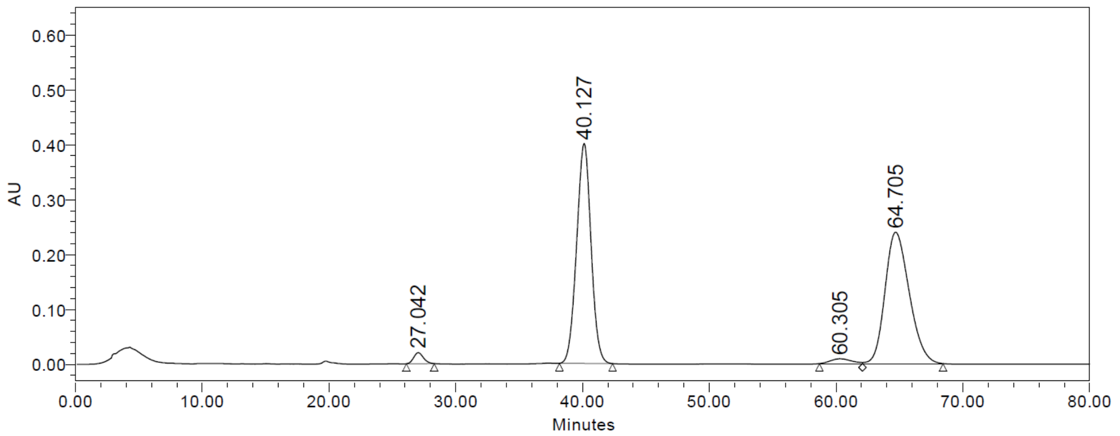

	RT	Area	% Area	Height
1	27.042	1072744	1.64	20094
2	40.127	31716429	48.38	400577
3	60.305	996298	1.52	8801
4	64.705	31775720	48.47	239319

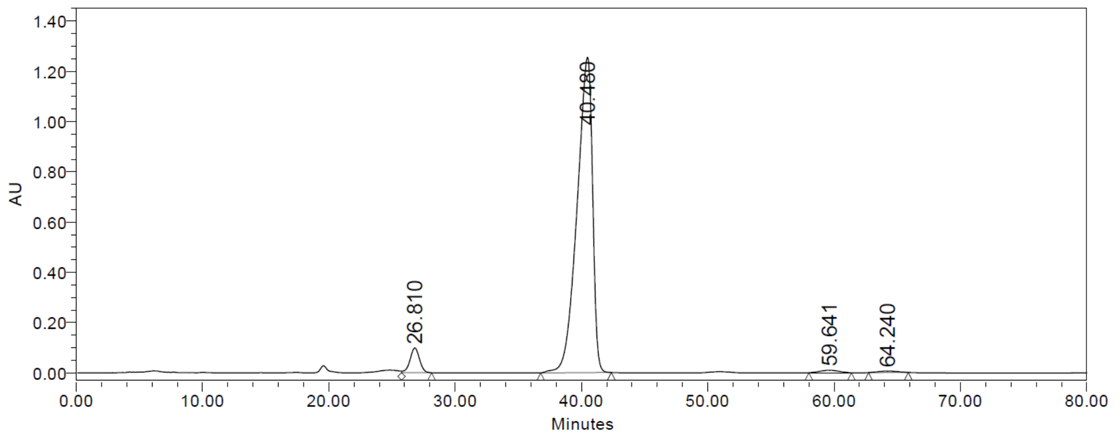

	RT	Area	% Area	Height
1	26.810	5106750	4.50	98212
2	40.480	106877211	94.10	1254598
3	59.641	996099	0.88	9770
4	64.240	603363	0.53	5880

Supplementary Fig. 127 HPLC analysis of *cis*-**3ia**

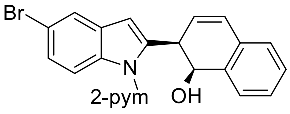

3ia

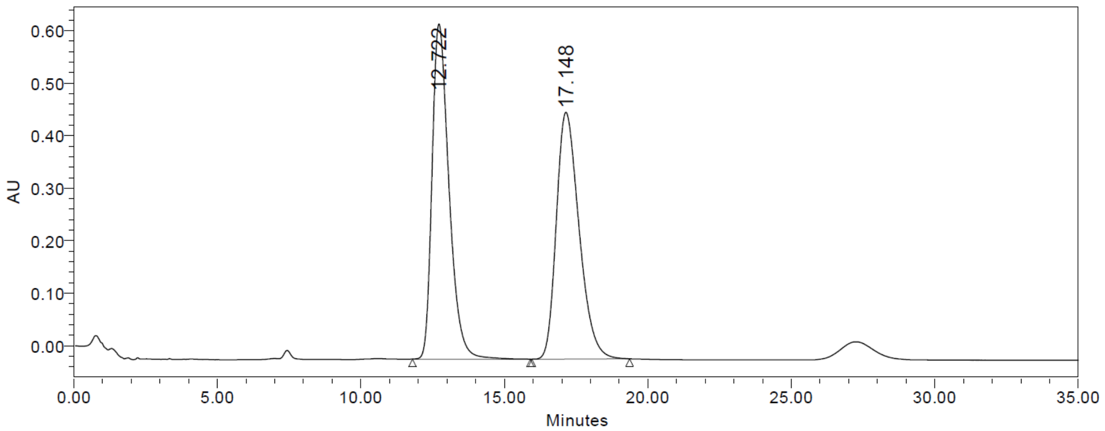

	RT	Area	% Area	Height
1	12.722	26229748	50.32	638505
2	17.148	25899940	49.68	470117

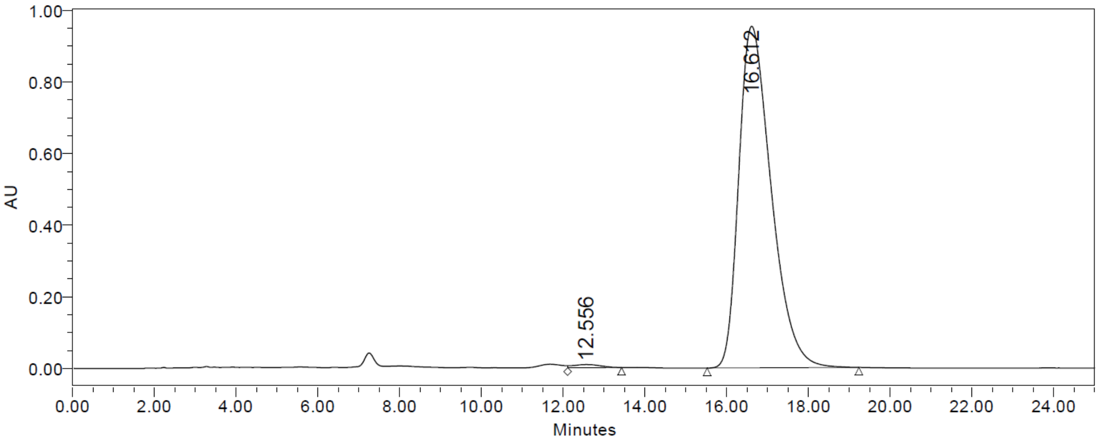

	RT	Area	% Area	Height
1	12.556	406895	0.77	8795
2	16.612	52506220	99.23	953879

Supplementary Fig. 128 HPLC analysis of *cis*-**3ja**

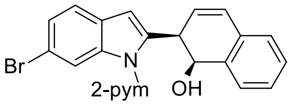

3ja

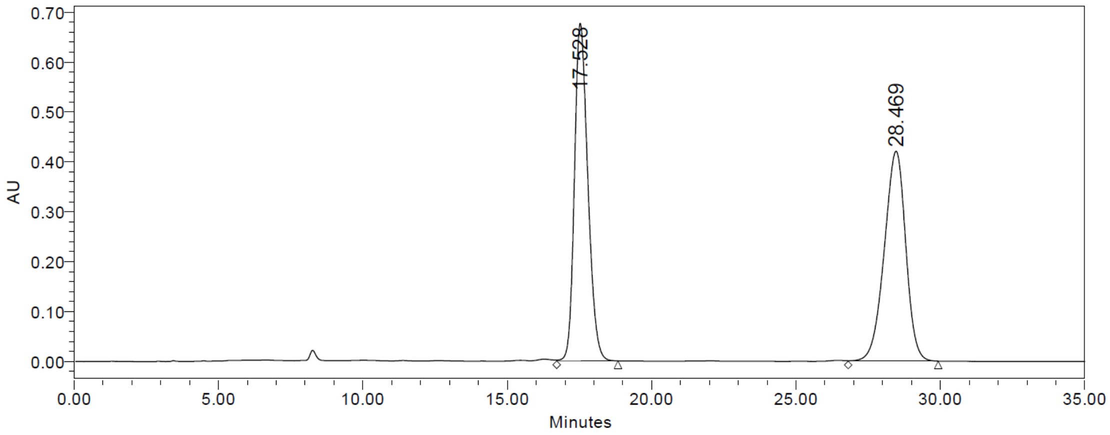

	RT	Area	% Area	Height
1	17.528	21806508	50.05	676703
2	28.469	21765859	49.95	420811

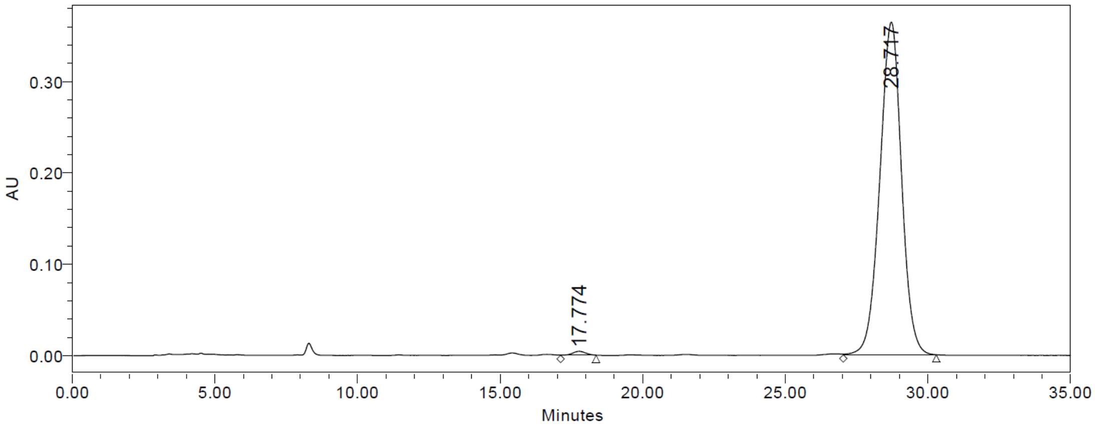

	RT	Area	% Area	Height
1	17.774	129745	0.67	4235
2	28.717	19262418	99.33	364151

Supplementary Fig. 129 HPLC analysis of *cis*-**3ka**

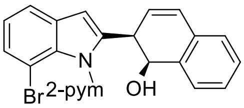

3ka

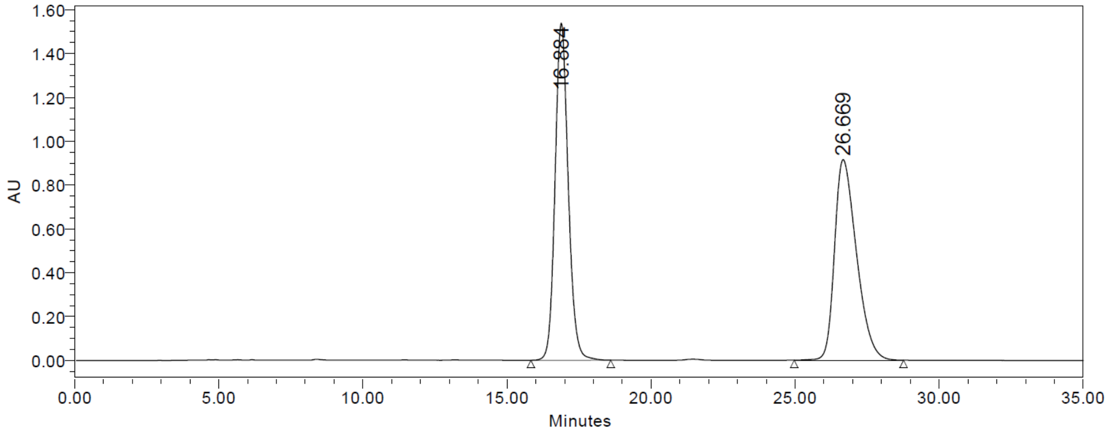

	RT	Area	% Area	Height
1	16.884	48732882	50.14	1537753
2	26.669	48466192	49.86	914660

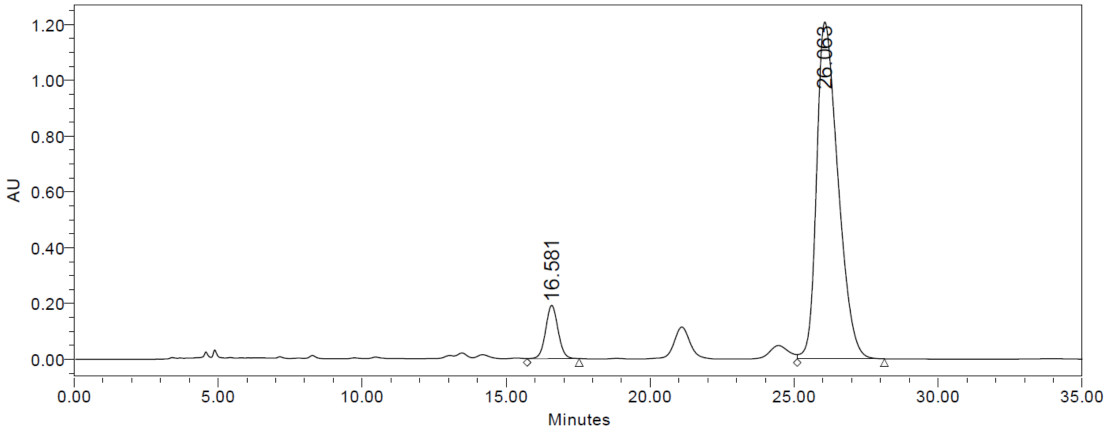

	RT	Area	% Area	Height
1	16.581	5691673	8.38	190959
2	26.063	62251444	91.62	1207289

Supplementary Fig. 130 HPLC analysis of *cis*-**3la**

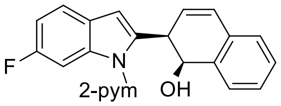

3la

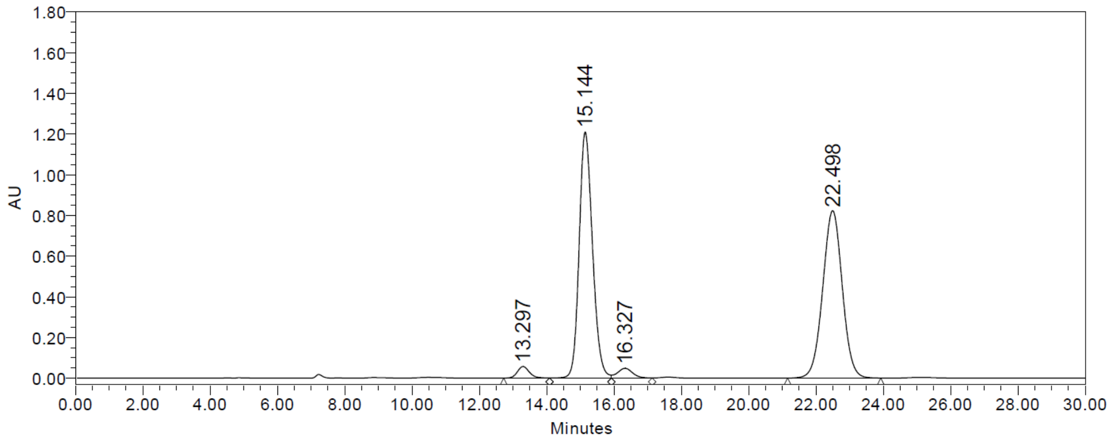

	RT	Area	% Area	Height
1	13.297	1396510	2.02	56905
2	15.144	33215102	47.96	1209330
3	16.327	1486202	2.15	48130
4	22.498	33157955	47.88	822463

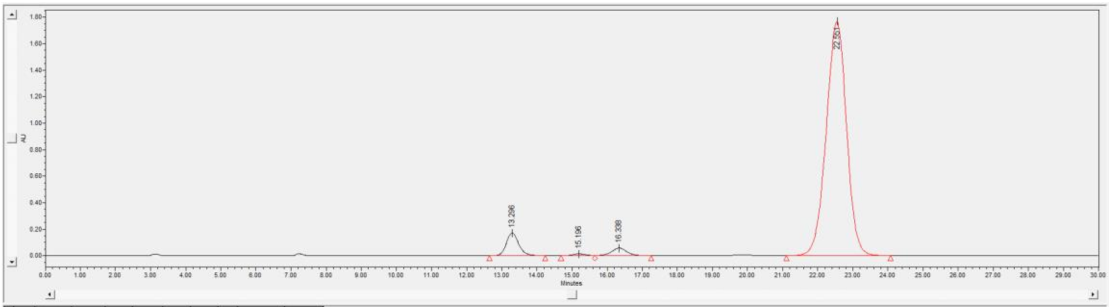

	Name	Retention Time (min)	Area (µV*sec)	% Area	Height (µV)	Int Type	Amount	Units	Peak Type	Peak Codes
1		13.296	4133951	5.35	171890	BB			Unknown	
2		15.196	307388	0.40	12021	BV			Unknown	
3		16.338	1702687	2.20	56669	VB			Unknown	
4		22.551	71089769	92.04	1764799	BB			Unknown	

Supplementary Fig. 131 HPLC analysis of *cis*-**3ma**

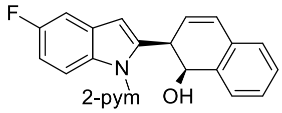

3ma

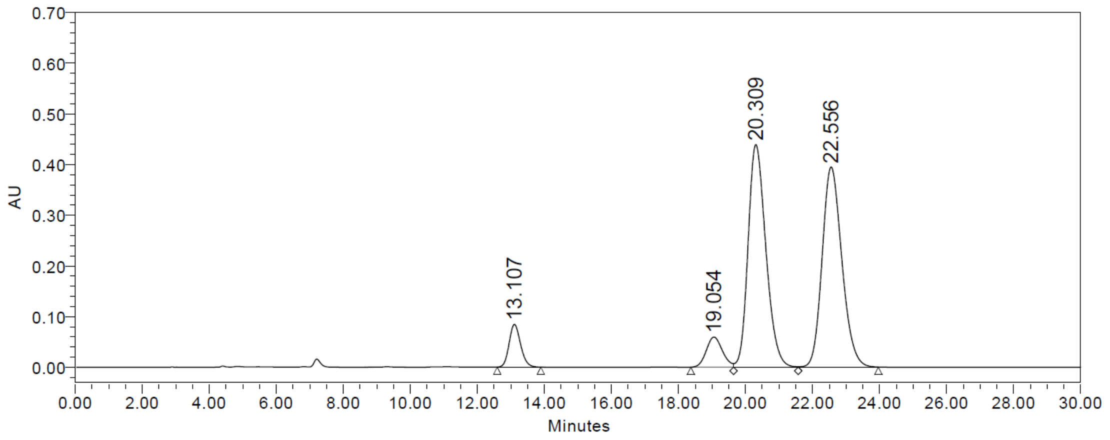

	RT	Area	% Area	Height
1	13.107	2006280	5.53	84223
2	19.054	1979883	5.46	59395
3	20.309	16166905	44.55	439131
4	22.556	16138573	44.47	394803

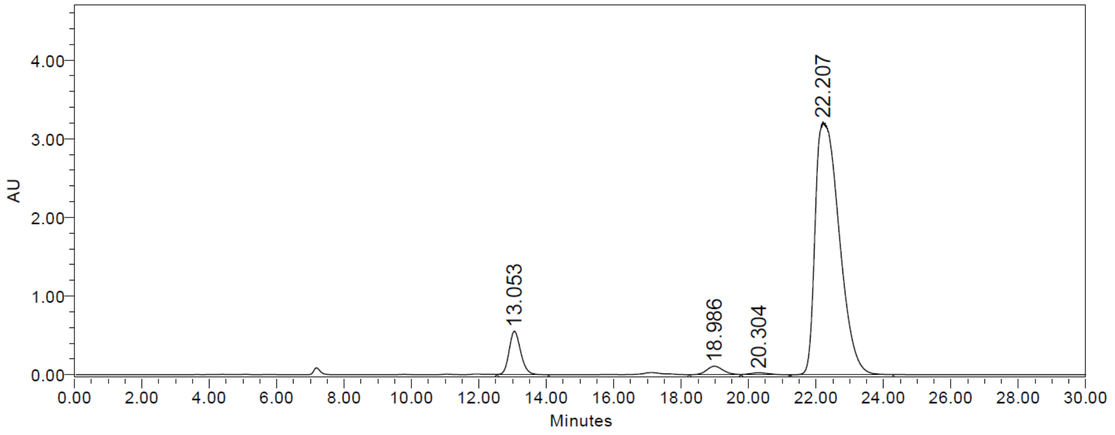

	RT	Area	% Area	Height
1	13.053	13172155	7.46	551671
2	18.986	3613771	2.05	107161
3	20.304	880486	0.50	24423
4	22.207	158800725	89.99	3213856

Supplementary Fig. 132 HPLC analysis of *cis*-**3na**

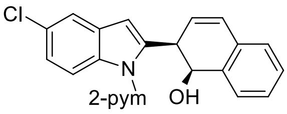

3na

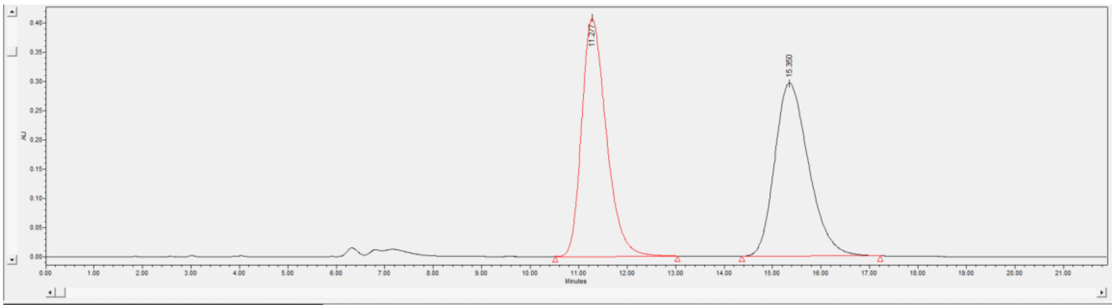

	Name	Retention Time (min)	Area (μV*sec)	% Area	Height (μV)	Int Type	Amount	Units	Peak Type	Peak Codes
1		11.277	14364469	49.91	406962	BB			Unknown	
2		15.350	14418294	50.09	296195	BB			Unknown	

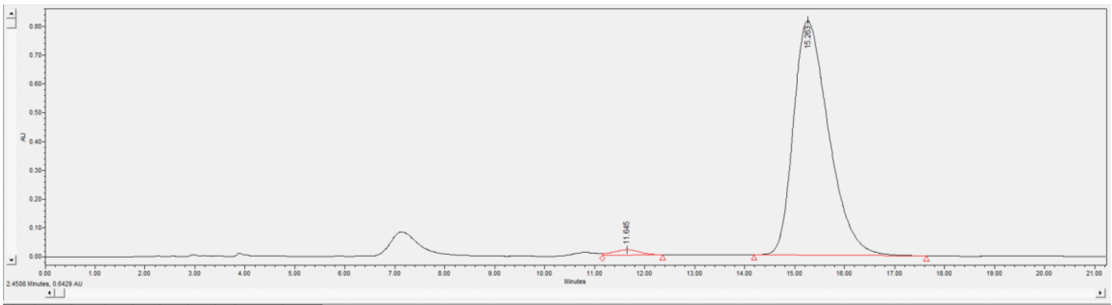

	Name	Retention Time (min)	Area (μV*sec)	% Area	Height (μV)	Int Type	Amount	Units	Peak Type	Peak Codes
1		11.645	694295	1.71	18826	VB			Unknown	I08
2		15.263	39843373	98.29	813196	BB			Unknown	

Supplementary Fig. 133 HPLC analysis of *cis*-**3oa**

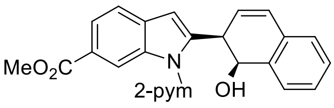

3oa

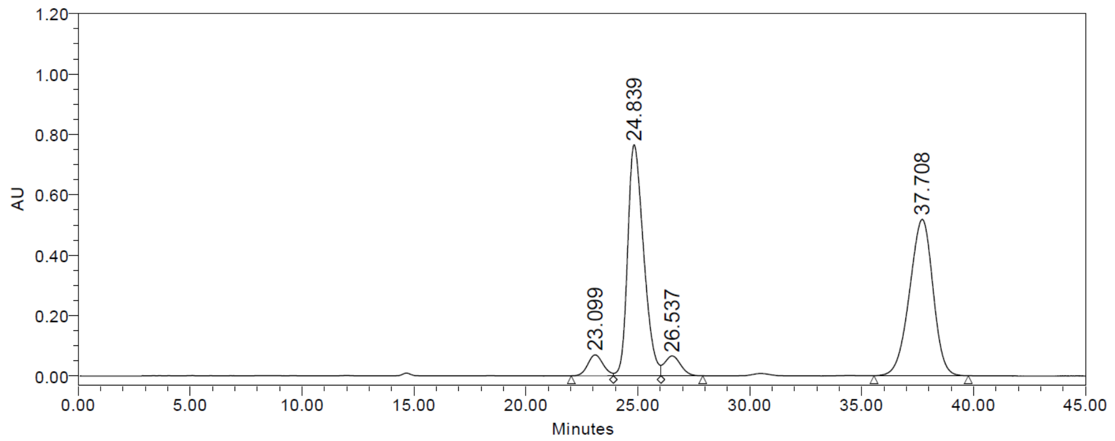

	RT	Area	% Area	Height
1	23.099	3326325	4.04	69643
2	24.839	37951716	46.09	765425
3	26.537	3306635	4.02	65887
4	37.708	37762480	45.86	517889

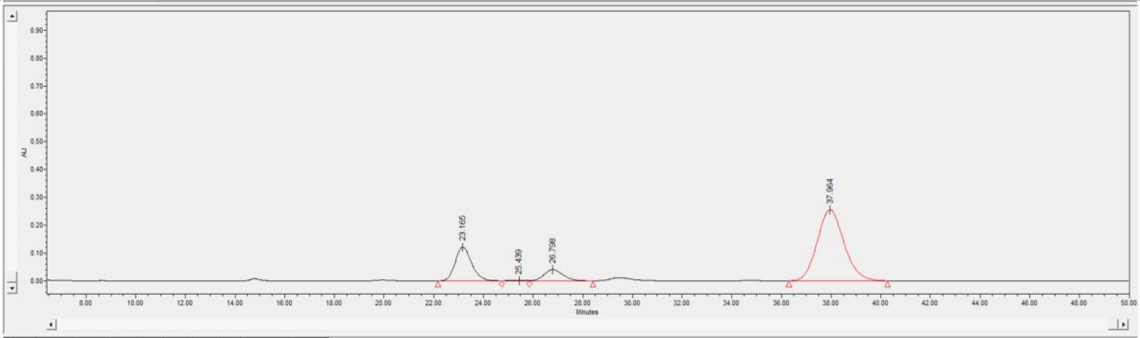

	Name	Retention Time (min)	Area (µV*sec)	% Area	Height (µV)	Int Type	Amount	Units	Peak Type	Peak Codes
1		23.165	5624728	20.76	121914	BV			Unknown	
2		25.439	98269	0.36	2210	VV			Unknown	
3		26.798	2172009	8.02	40654	VB			Unknown	
4		37.964	19192718	70.85	255325	BB			Unknown	

Supplementary Fig. 134 HPLC analysis of *cis*-**3pa**

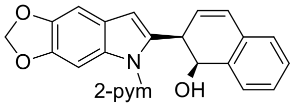

3pa

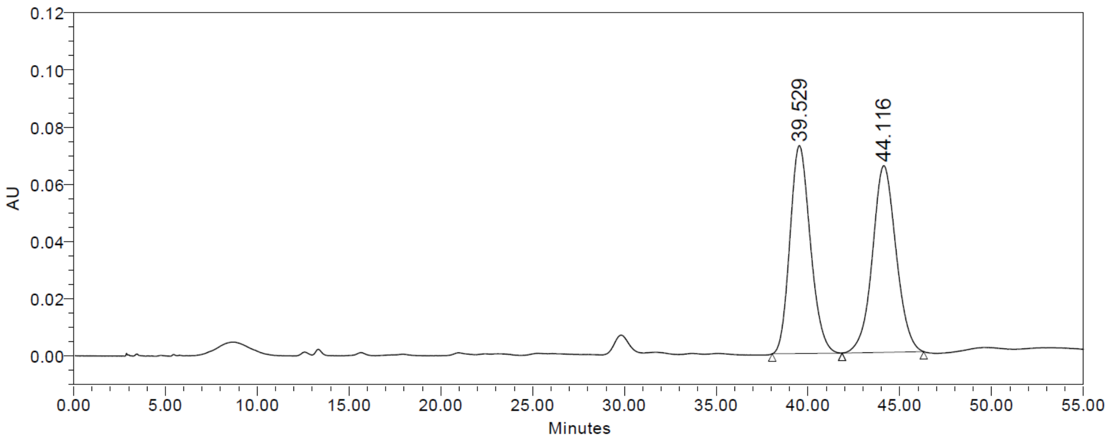

	RT	Area	% Area	Height
1	39.529	5625556	49.02	72705
2	44.116	5850708	50.98	65267

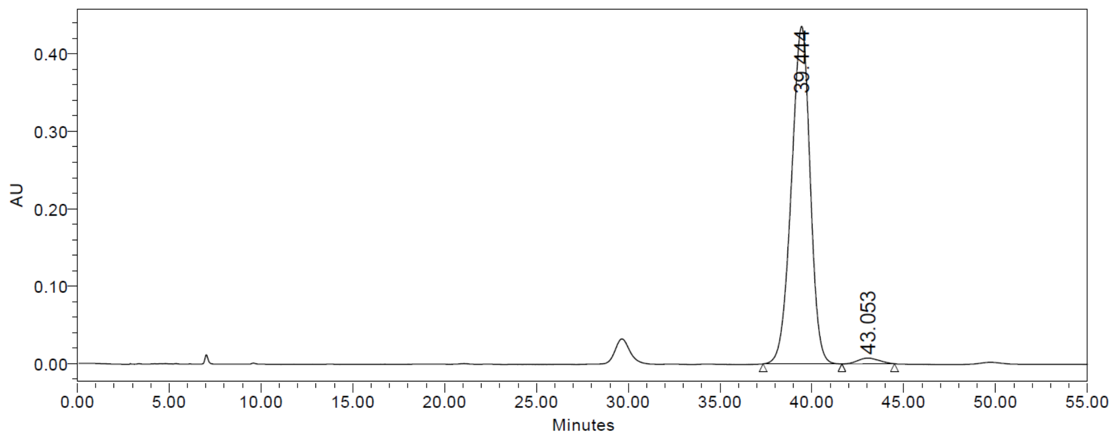

	RT	Area	% Area	Height
1	39.444	30951166	98.10	435998
2	43.053	597927	1.90	7399

Supplementary Fig. 135 HPLC analysis of *cis*-**3qa**

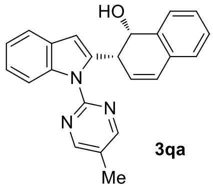

3qa

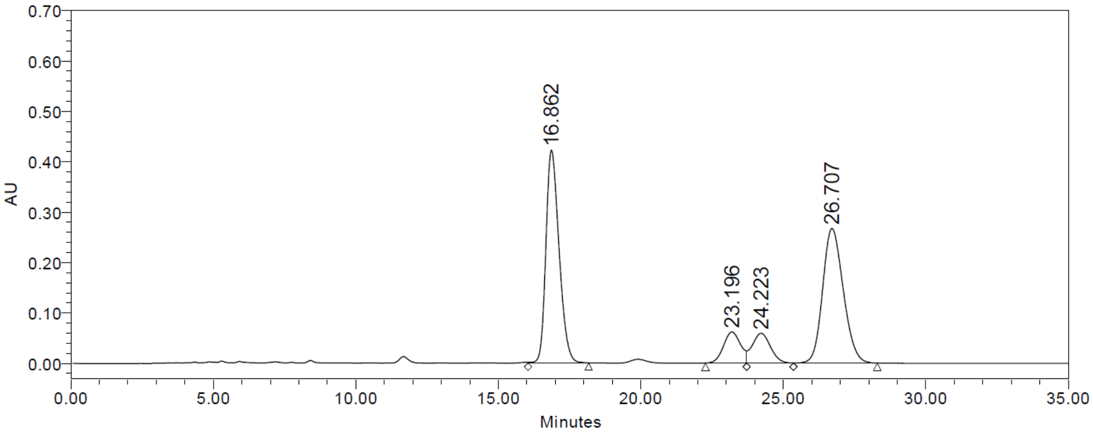

	RT	Area	% Area	Height
1	16.862	13524654	42.08	422029
2	23.196	2569074	7.99	61677
3	24.223	2623896	8.16	59065
4	26.707	13424913	41.77	267113

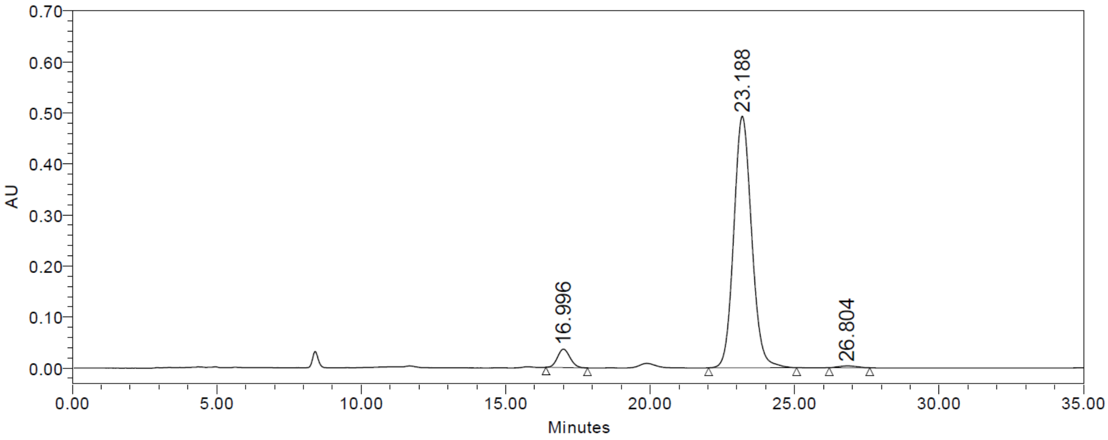

	RT	Area	% Area	Height
1	16.996	1086970	4.85	36133
2	23.188	21187476	94.47	492941
3	26.804	153622	0.68	3598

Supplementary Fig. 136 HPLC analysis of *cis*-**3ra**

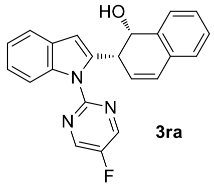

3ra

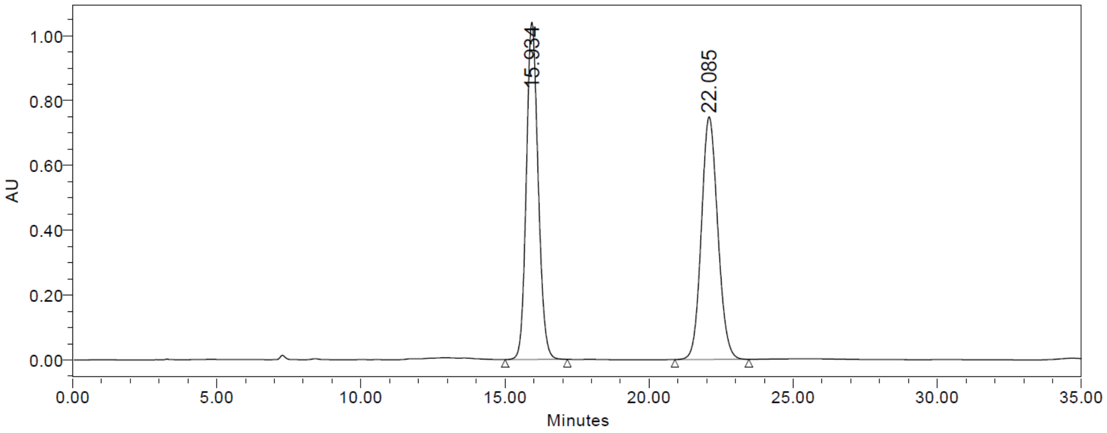

	RT	Area	% Area	Height
1	15.934	29666489	50.02	1040395
2	22.085	29644123	49.98	748596

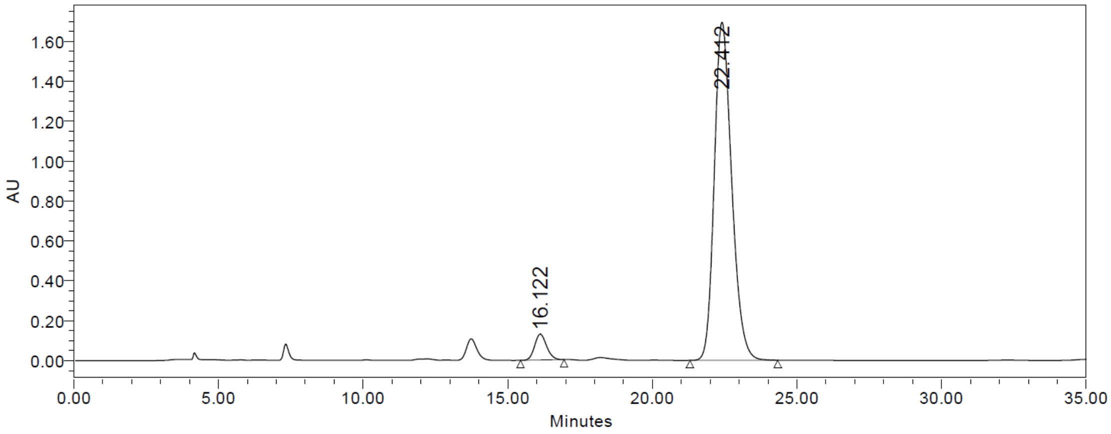

	RT	Area	% Area	Height
1	16.122	3724376	4.99	129645
2	22.412	70952257	95.01	1694513

Supplementary Fig. 137 HPLC analysis of *cis*-**3sa**

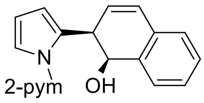

3sa

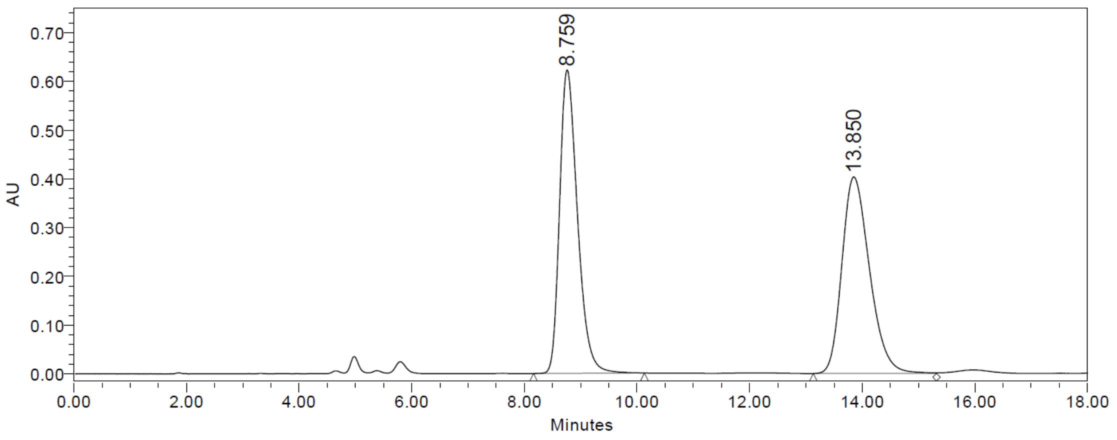

	RT	Area	% Area	Height
1	8.759	13738403	50.02	622547
2	13.850	13727319	49.98	403155

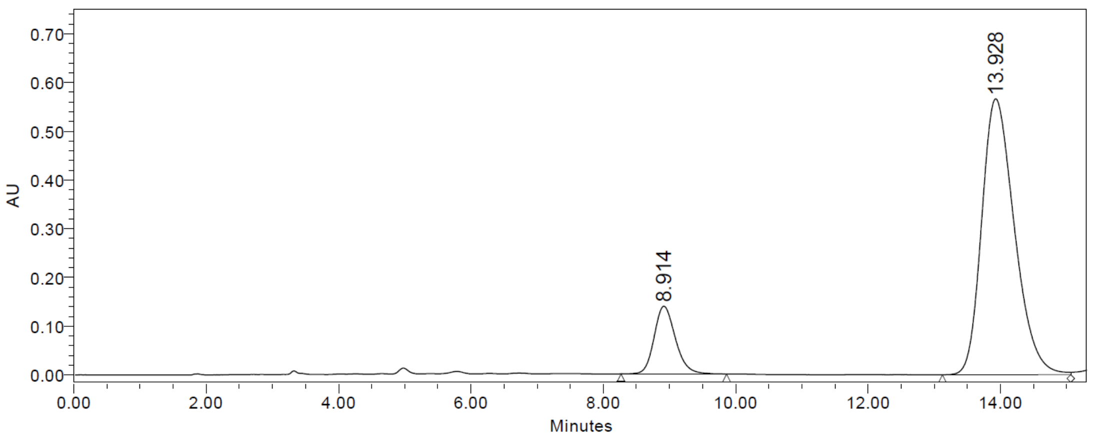

	RT	Area	% Area	Height
1	8.914	3116803	13.79	139162
2	13.928	19491317	86.21	566282

Supplementary Fig. 138 HPLC analysis of *cis*-**3ta**

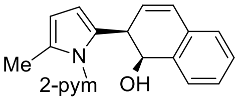

3ta

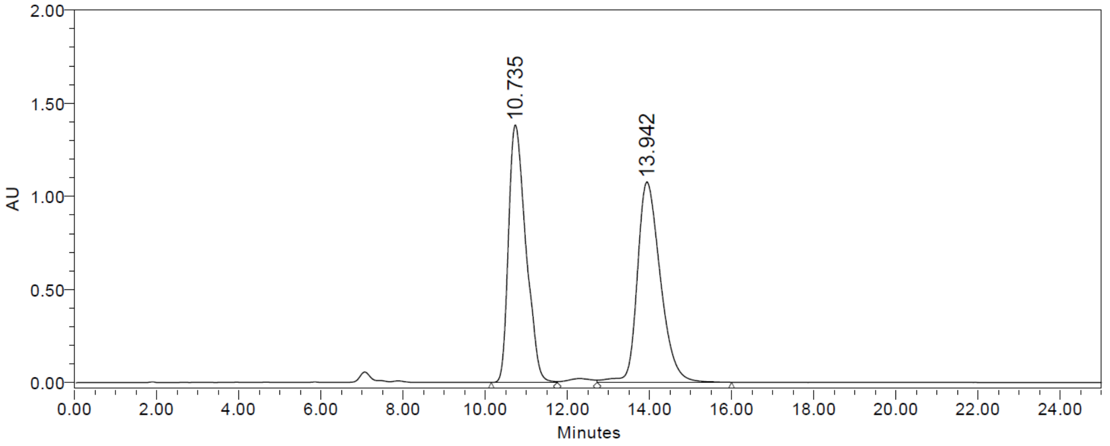

	RT	Area	% Area	Height
1	10.735	40656682	49.21	1382487
2	13.942	41957166	50.79	1075260

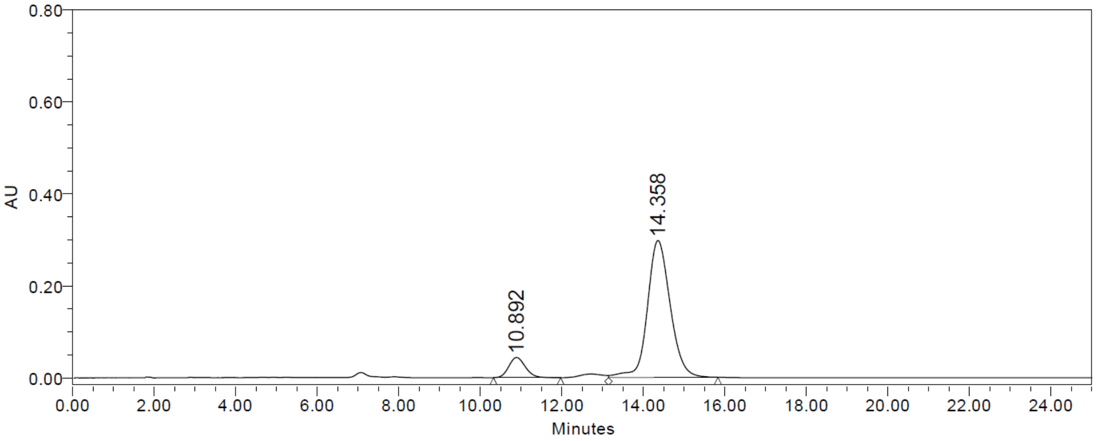

	RT	Area	% Area	Height
1	10.892	1240294	9.67	43866
2	14.358	11581038	90.33	297783

Supplementary Fig. 139 HPLC analysis of *cis*-**3ua**

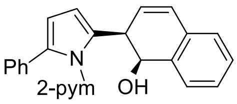

3ua

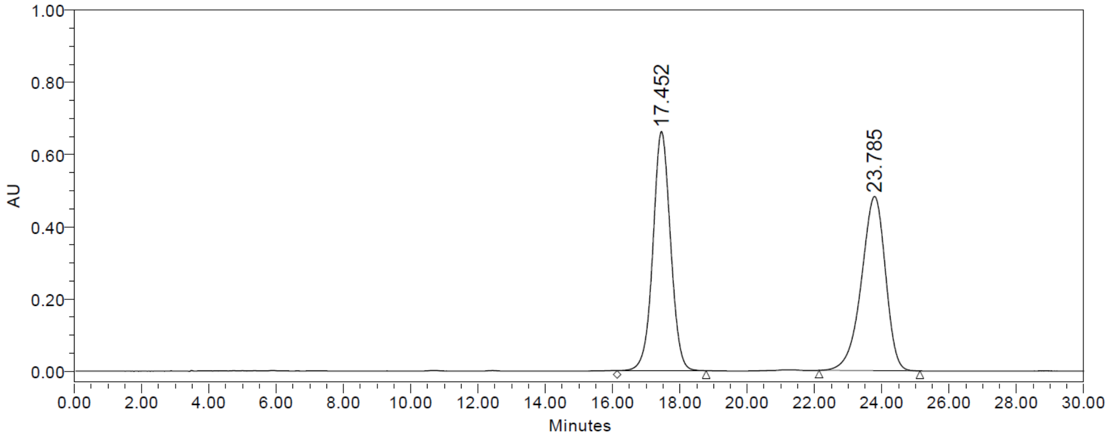

	RT	Area	% Area	Height
1	17.452	24154385	50.14	662451
2	23.785	24016828	49.86	482805

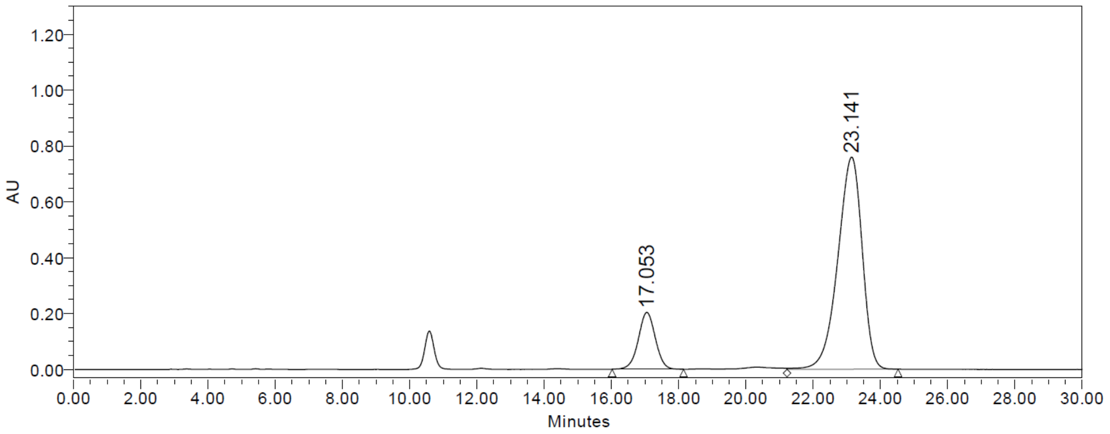

	RT	Area	% Area	Height
1	17.053	7166806	15.81	203626
2	23.141	38159115	84.19	759305

Supplementary Fig. 140 HPLC analysis of *cis*-**3va**

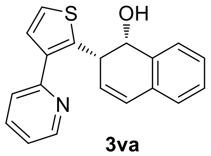

3va

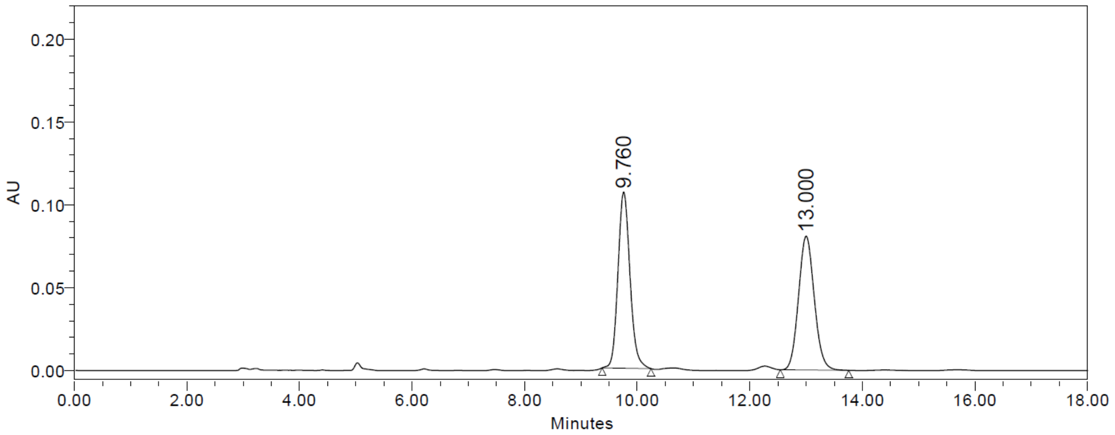

	RT	Area	% Area	Height
1	9.760	1577576	50.11	106307
2	13.000	1570419	49.89	80732

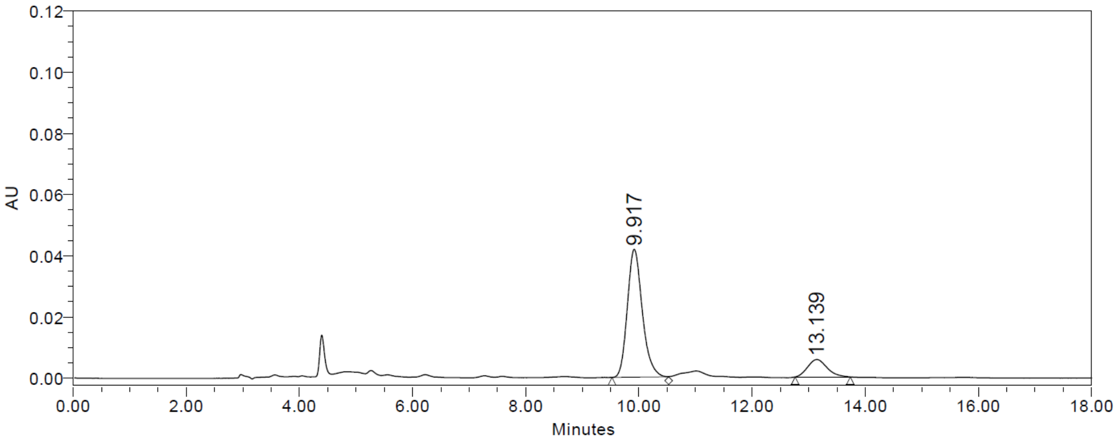

	RT	Area	% Area	Height
1	9.917	769198	85.00	41811
2	13.139	135785	15.00	5690

Supplementary Fig. 141 HPLC analysis of *cis*-**3wa**

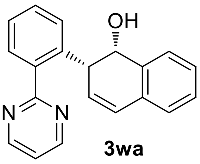

3wa

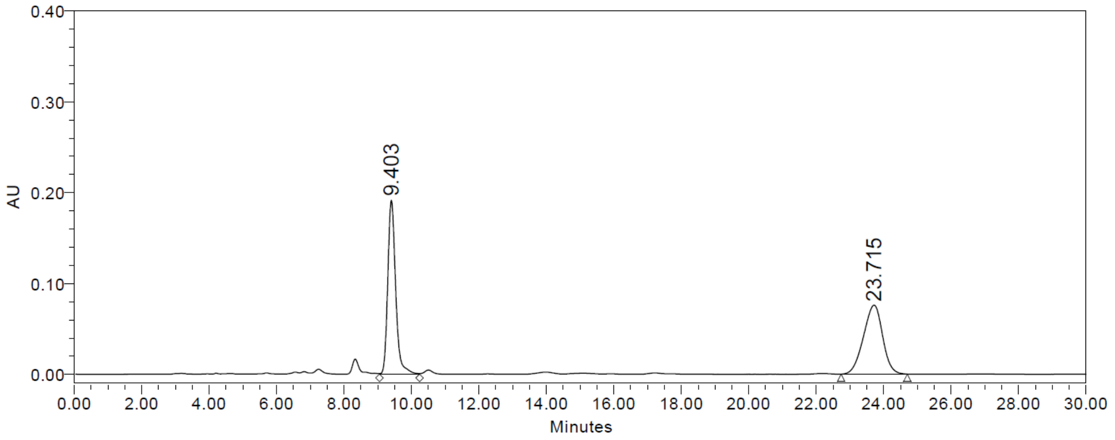

	RT	Area	% Area	Height
1	9.403	3097001	50.86	191453
2	23.715	2992585	49.14	75881

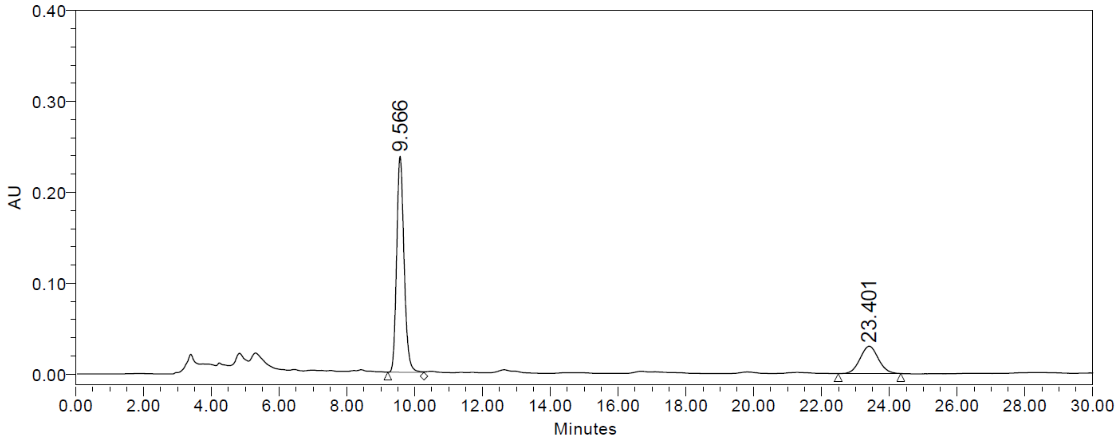

	RT	Area	% Area	Height
1	9.566	3622881	76.27	237776
2	23.401	1126975	23.73	30088

Supplementary Fig. 142 HPLC analysis of *cis*-**3ab**

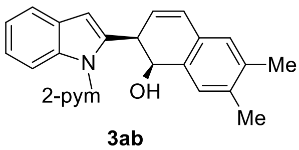

3ab

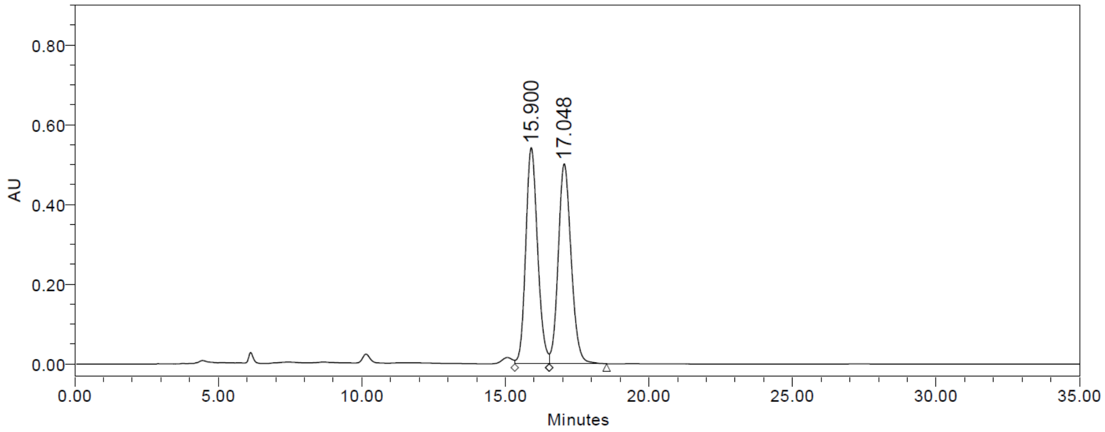

	RT	Area	% Area	Height
1	15.900	15545802	49.74	542407
2	17.048	15706302	50.26	501771

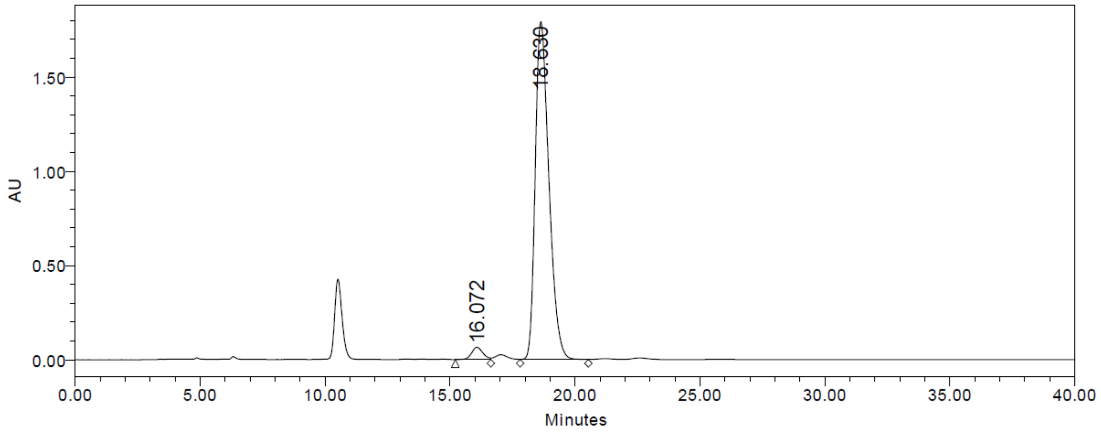

Channel: 2998 Ch1 254nm@1.2nm; Processed Channel: 2998 Ch1 254nm@1.2nm; Result Id: 4781; Processing Method: test

Processed Channel Descr.: 2998 Ch1 254nm@1.2nm

	Processed Channel Descr.	RT	Area	% Area	Height
1	2998 Ch1 254nm@1.2nm	16.072	2035549	2.90	64755
2	2998 Ch1 254nm@1.2nm	18.630	68234605	97.10	1792588

Supplementary Fig. 143 HPLC analysis of *cis*-**3ac**

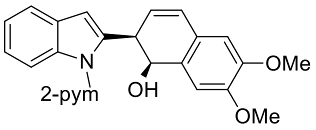

3ac

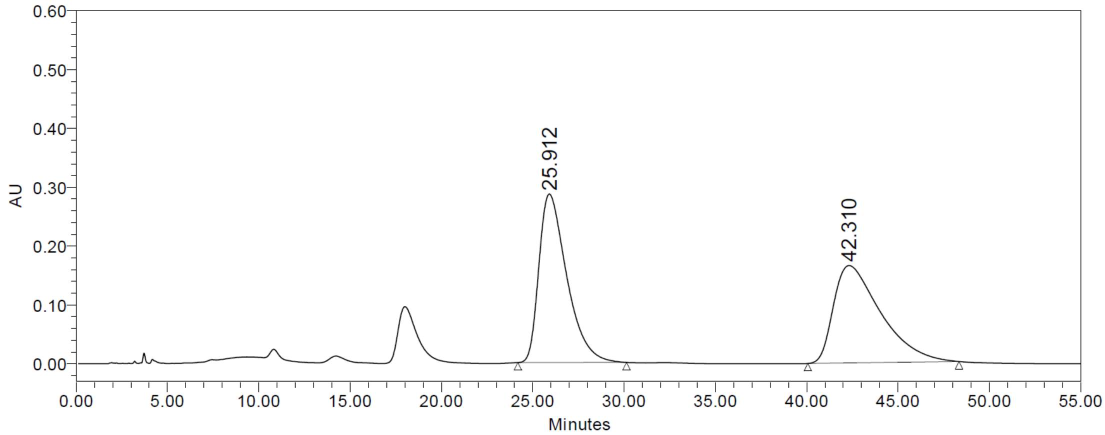

	RT	Area	% Area	Height
1	25.912	30378128	50.52	286539
2	42.310	29750203	49.48	165577

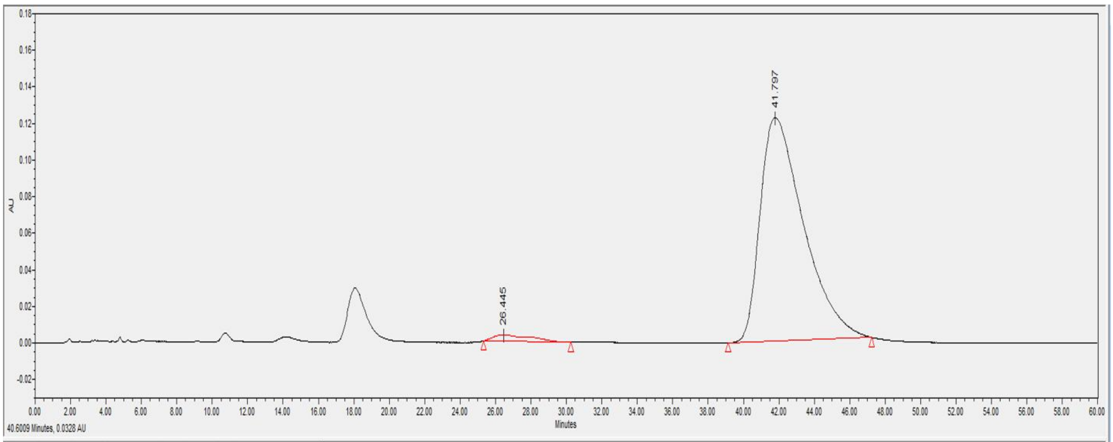

	Name	Retention Time (min)	Area (µV*sec)	% Area	Height (µV)	Int Type	Amount	Units	Peak Type	Peak Codes
1		26.445	532806	2.49	3574	BB			Unknown	
2		41.797	20858441	97.51	122209	BB			Unknown	

Supplementary Fig. 144 HPLC analysis of *cis*-**3ad**

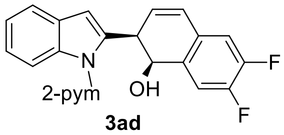

3ad

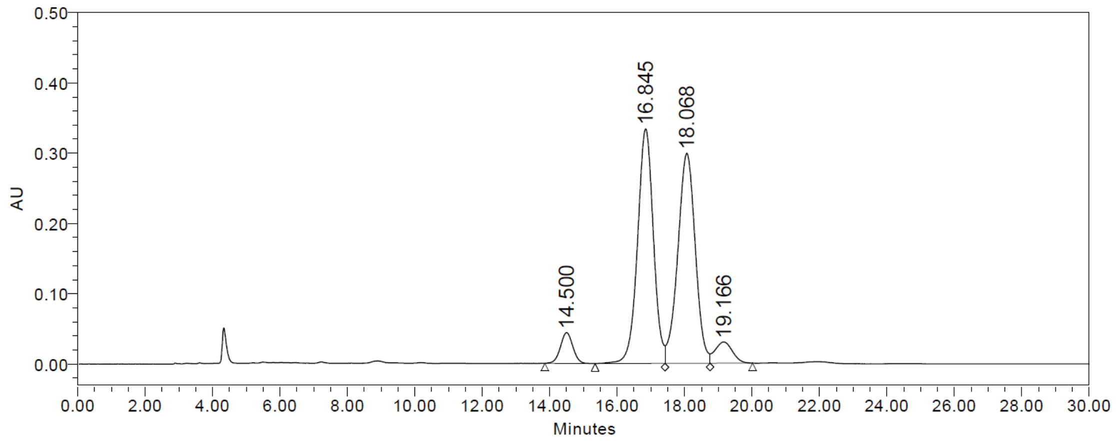

	RT	Area	% Area	Height
1	14.500	1122281	4.70	43758
2	16.845	10878405	45.60	333736
3	18.068	10773377	45.16	298538
4	19.166	1083725	4.54	30241

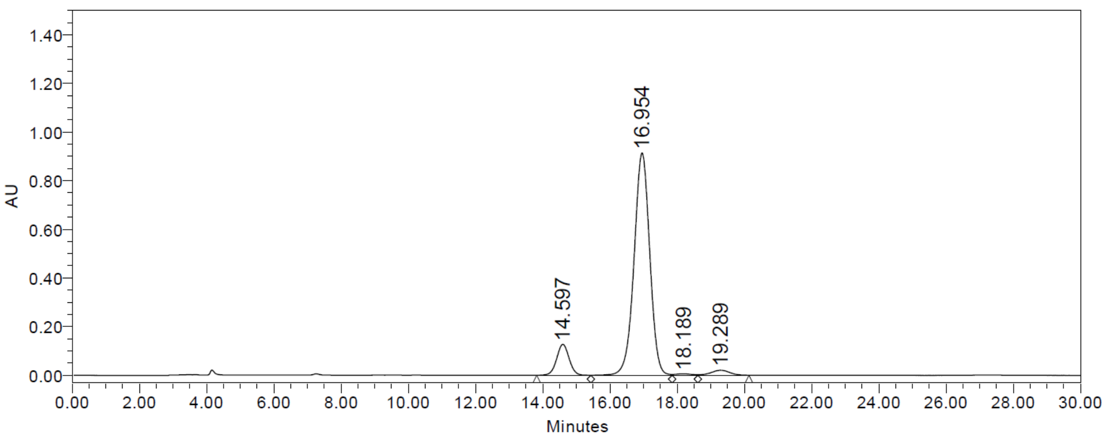

	RT	Area	% Area	Height
1	14.597	3285248	9.68	127426
2	16.954	29669664	87.43	914588
3	18.189	195285	0.58	5702
4	19.289	783665	2.31	20566

Supplementary Fig. 145 HPLC analysis of *cis*-**3ae**

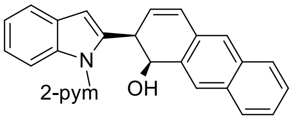

3ae

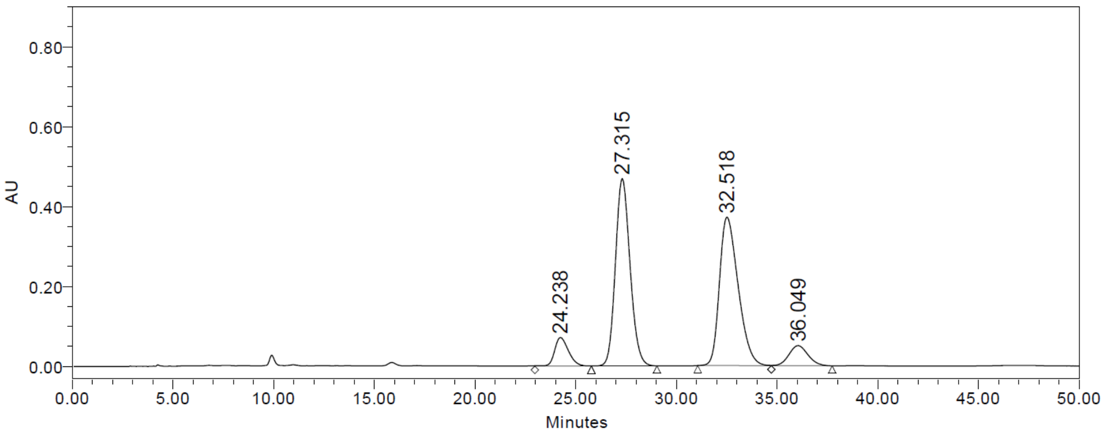

	RT	Area	% Area	Height
1	24.238	3390898	6.24	71323
2	27.315	23684334	43.55	468727
3	32.518	23984136	44.11	371615
4	36.049	3318963	6.10	50331

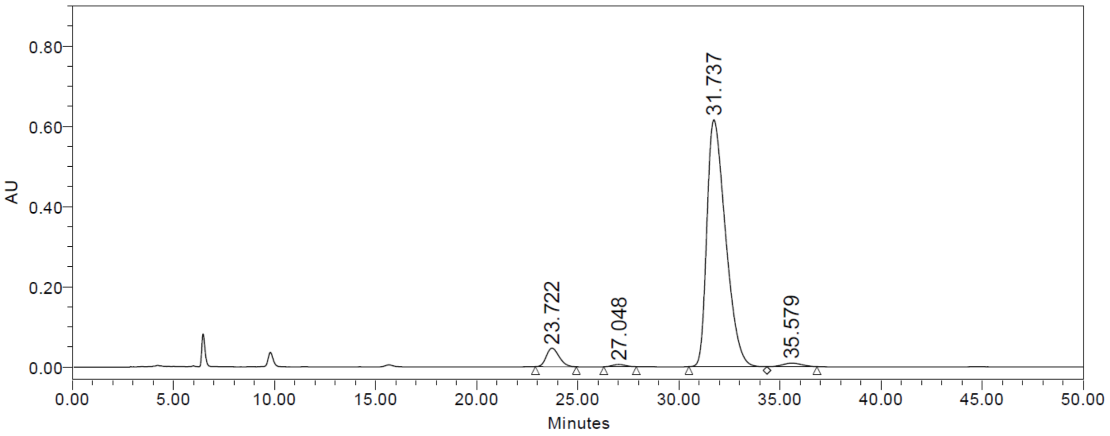

	RT	Area	% Area	Height
1	23.722	2045423	4.83	46380
2	27.048	254094	0.60	5609
3	31.737	39513901	93.23	615339
4	35.579	570311	1.35	8943

Supplementary Fig. 146 HPLC analysis of *cis*-**3af**

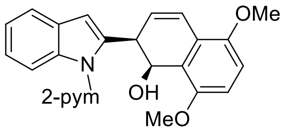

3af

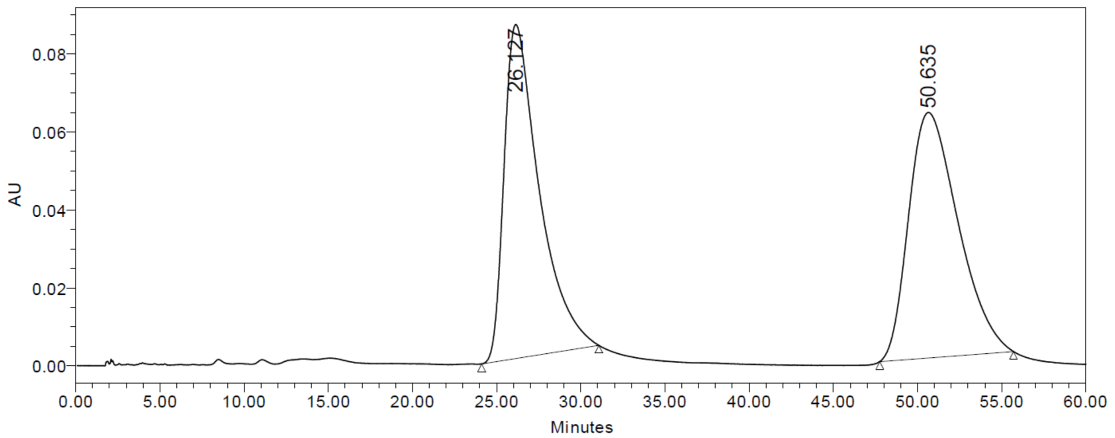

Channel: 2998 Ch1 254nm@1.2nm; Processed Channel: 2998 Ch1 254nm@1.2nm; Result Id: 4743; Processing Method: zds

Processed Channel Descr.: 2998 Ch1 254nm@1.2nm

	Processed Channel Descr.	RT	Area	% Area	Height
1	2998 Ch1 254nm@1.2nm	26.127	12470331	49.20	85708
2	2998 Ch1 254nm@1.2nm	50.635	12878125	50.80	63007

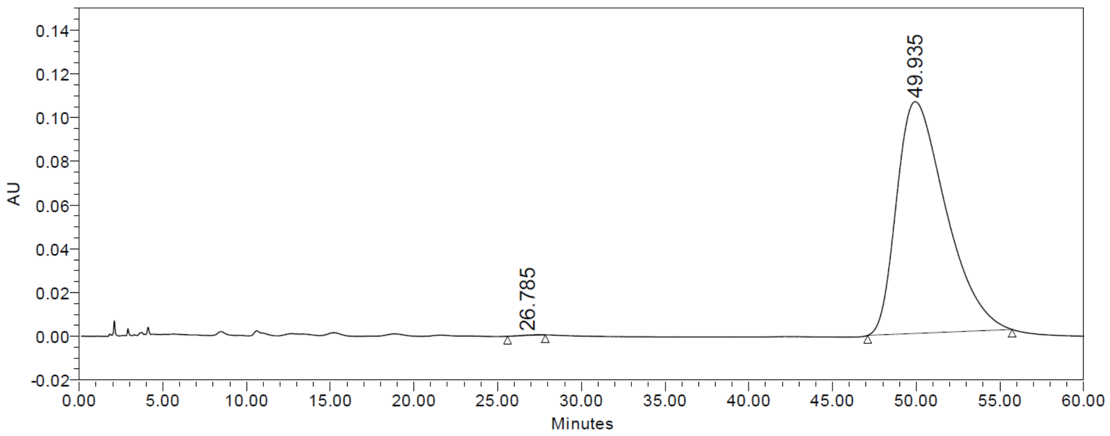

	RT	Area	% Area	Height
1	26.785	17352	0.08	252
2	49.935	21703335	99.92	106010

Supplementary Fig. 147 HPLC analysis of *cis*-**3ag**

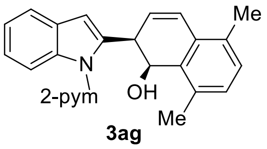

3ag

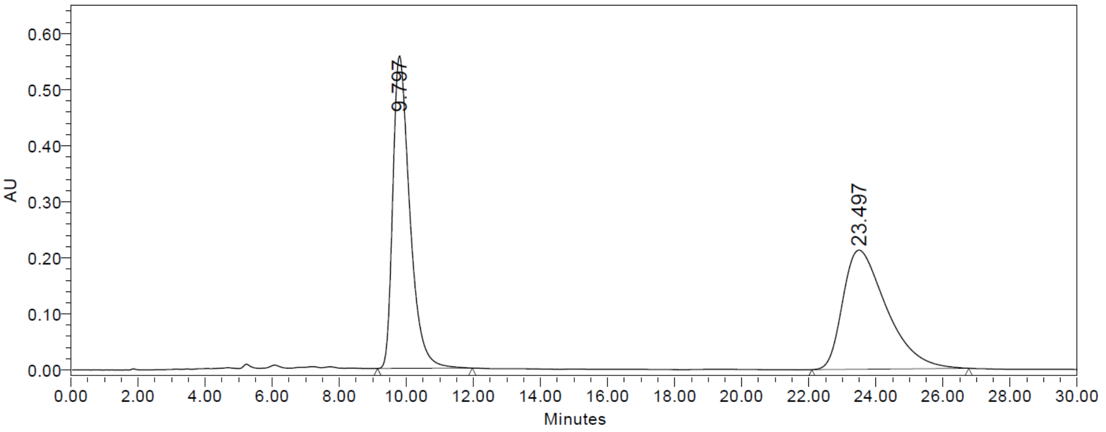

	RT	Area	% Area	Height
1	9.797	19604433	50.49	556877
2	23.497	19220950	49.51	212333

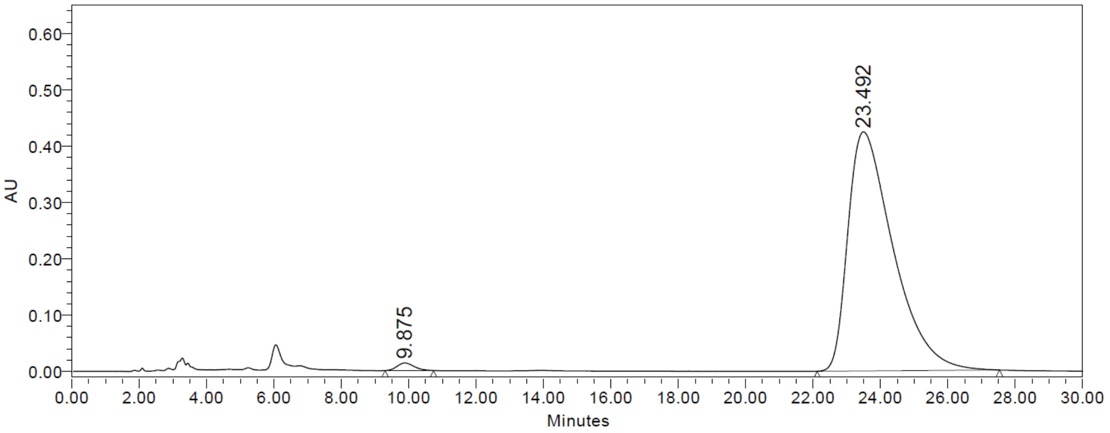

	RT	Area	% Area	Height
1	9.875	466209	1.16	13358
2	23.492	39581775	98.84	424281

Supplementary Fig. 148 HPLC analysis of *cis*-**3ah**

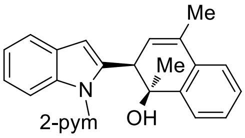

3ah

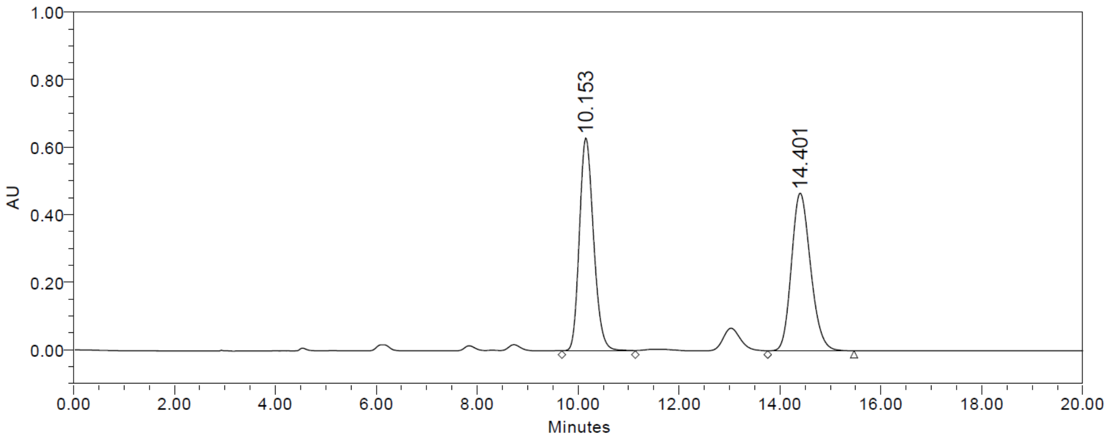

	RT	Area	% Area	Height
1	10.153	12318615	50.05	629669
2	14.401	12293527	49.95	467028

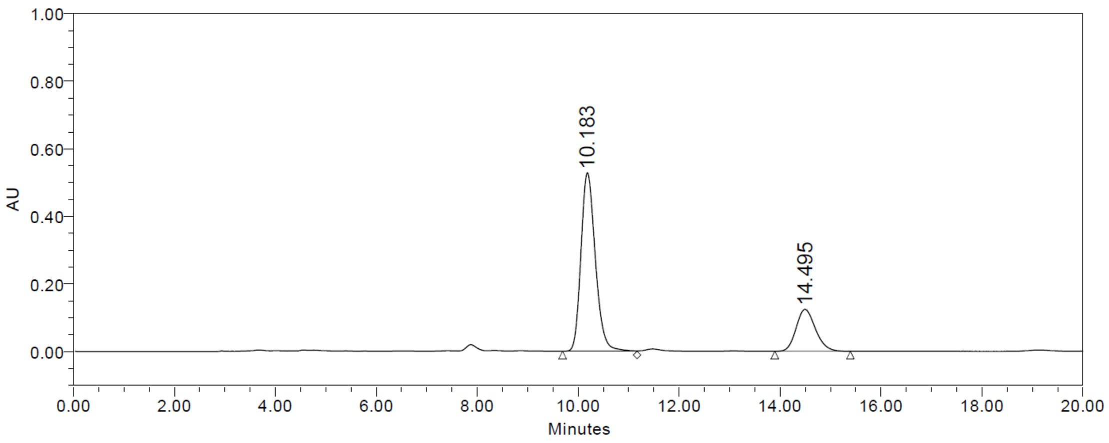

	RT	Area	% Area	Height
1	10.183	10470869	76.00	528265
2	14.495	3306139	24.00	123932

Supplementary Fig. 149 HPLC analysis of *cis*-**3ai**

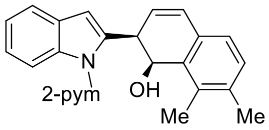

3ai

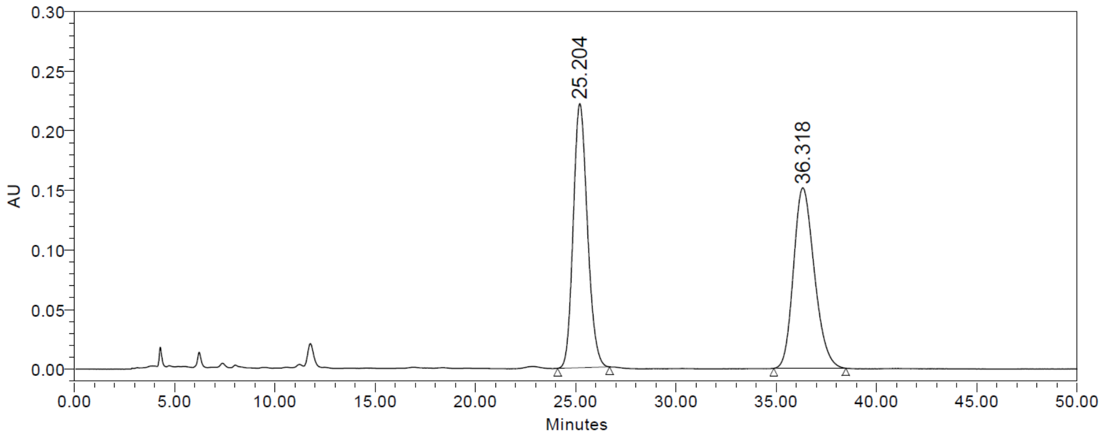

	RT	Area	% Area	Height
1	25.204	10894885	49.89	221745
2	36.318	10941382	50.11	151477

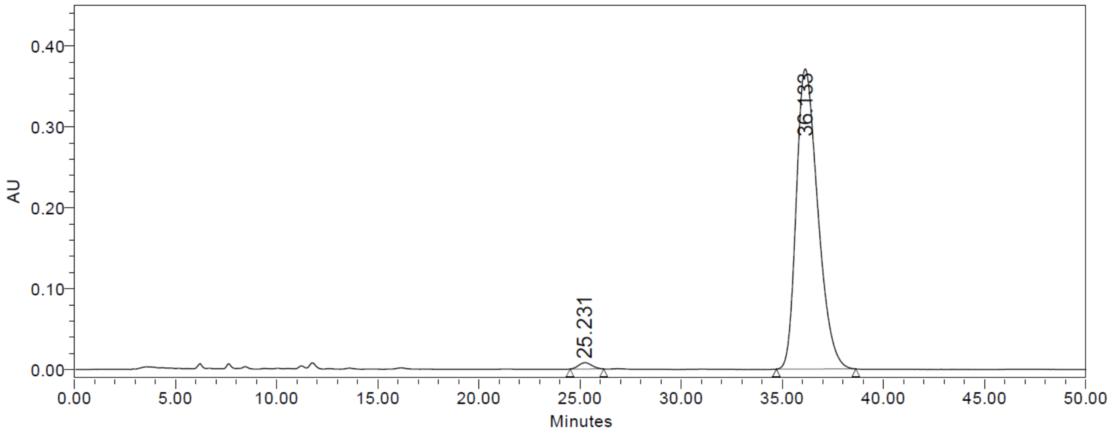

	RT	Area	% Area	Height
1	25.231	357594	1.28	7849
2	36.133	27489711	98.72	371009

Supplementary Fig. 150 HPLC analysis of *cis*-**3ai**'

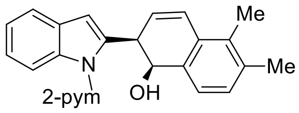

3ai'

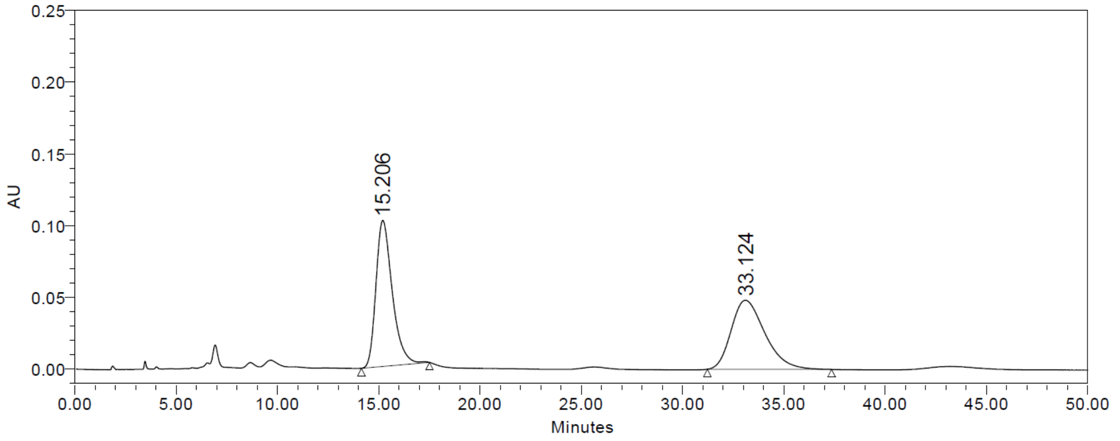

	RT	Area	% Area	Height
1	15.206	5709319	50.63	101826
2	33.124	5567101	49.37	48155

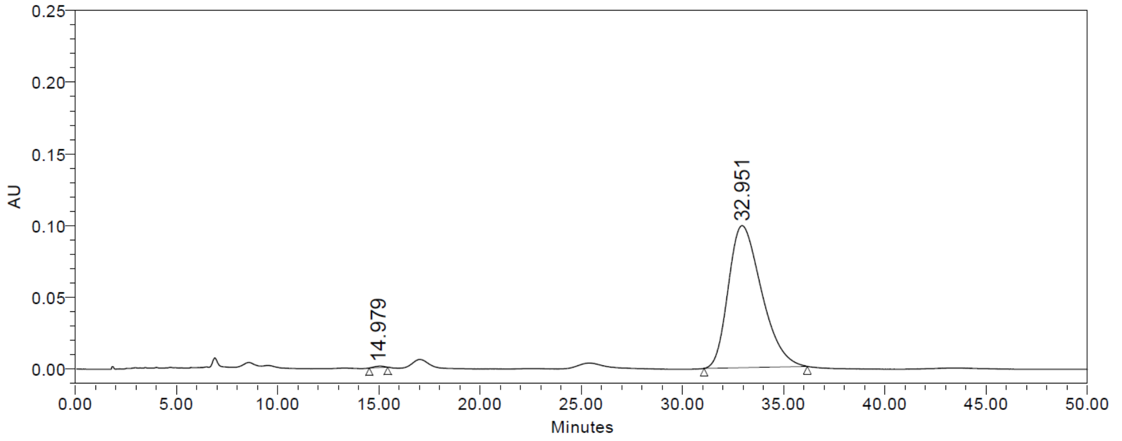

	RT	Area	% Area	Height
1	14.979	32560	0.29	990
2	32.951	11302564	99.71	99041

Supplementary Fig. 151 HPLC analysis of *cis*-**7aa**

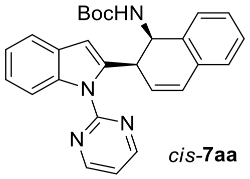

cis-**7aa**

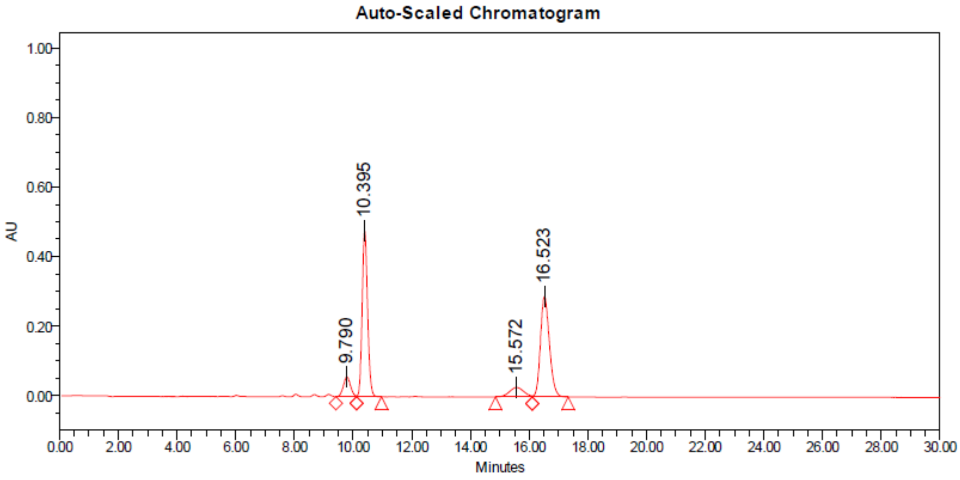

Peak Results

	SampleName	RT	Width (sec)	Height	Area	% Area
1	zy-08-62-rac	9.790	42.000	57063	968161	6.85
2	zy-08-62-rac	10.395	51.300	476892	6131092	43.35
3	zy-08-62-rac	15.572	75.500	27073	924101	6.53
4	zy-08-62-rac	16.523	73.700	288306	6120655	43.27

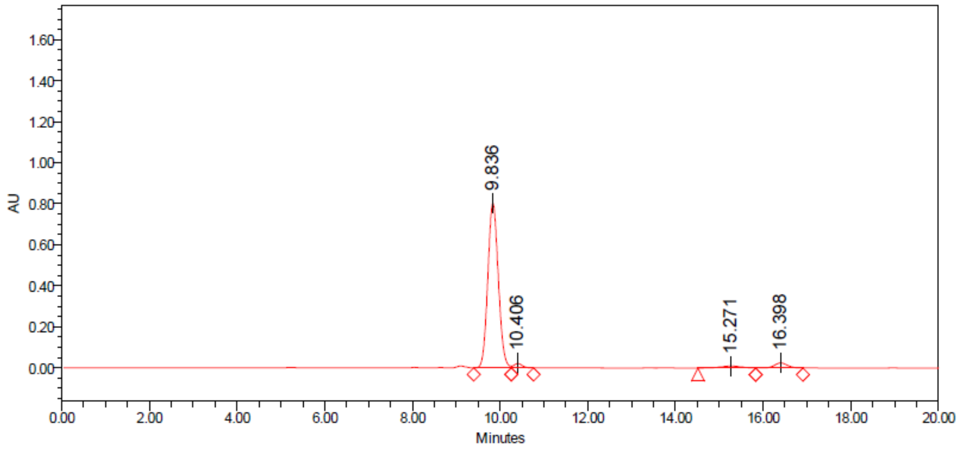

Peak Results

	SampleName	RT	Width (sec)	Height	Area	% Area
1	ZY-08-105	9.836	51.400	802981	13293555	92.34
2	ZY-08-105	10.406	30.300	21399	284576	1.98
3	ZY-08-105	15.271	79.100	9095	300569	2.09
4	ZY-08-105	16.398	64.600	24583	518297	3.60

Supplementary Fig. 152 HPLC analysis of *cis*-**7ab**

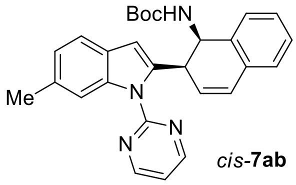

cis-**7ab**

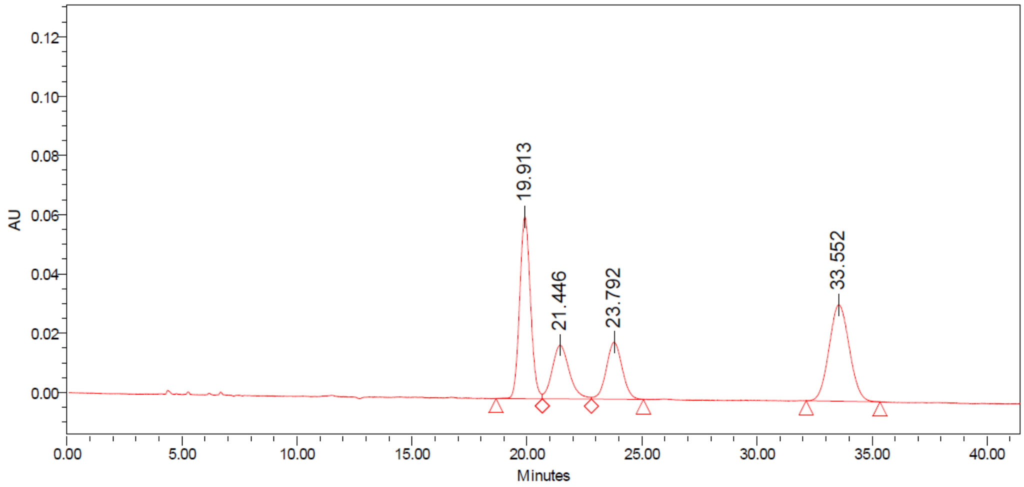

Peak Results

	SampleName	RT	Width (sec)	Height	Area	% Area
1	zy-10-12-rac	19.913	120.100	61390	2000489	34.20
2	zy-10-12-rac	21.446	128.500	18133	938051	16.03
3	zy-10-12-rac	23.792	135.500	19253	922676	15.77
4	zy-10-12-rac	33.552	192.100	32520	1988879	34.00

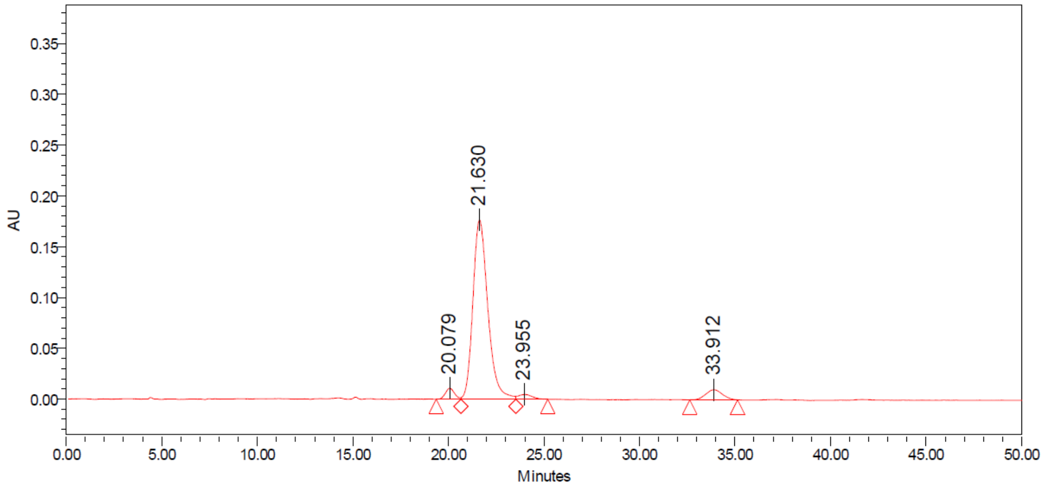

Peak Results

	SampleName	RT	Width (sec)	Height	Area	% Area
1	zy-10-3-1-ch	20.079	78.000	10865	358533	3.33
2	zy-10-3-1-ch	21.630	172.600	176579	9553853	88.82
3	zy-10-3-1-ch	23.955	100.300	4688	234099	2.18
4	zy-10-3-1-ch	33.912	150.400	10014	610068	5.67

Supplementary Fig. 153 HPLC analysis of *cis*-**7ac**

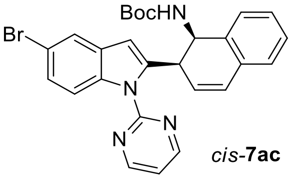

cis-**7ac**

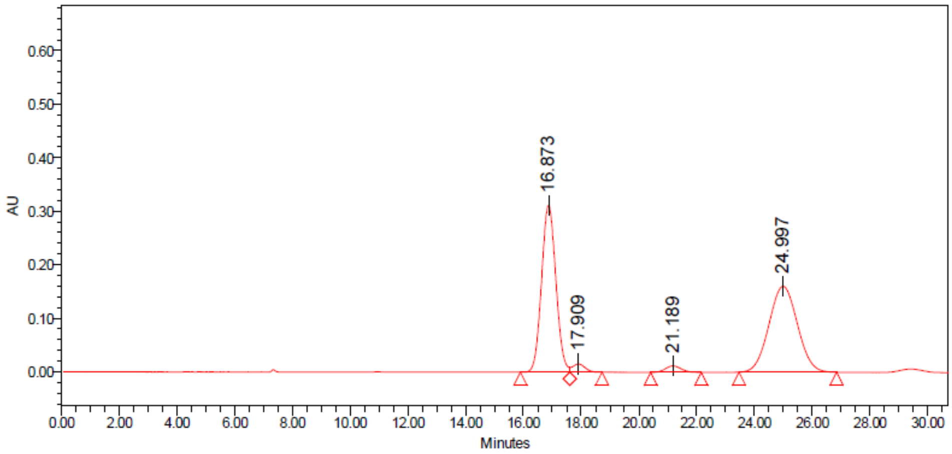

Peak Results

	SampleName	RT	Width (sec)	Height	Area	% Area
1	ZY-09-49-2-RAC	16.873	102.800	311620	10702487	47.35
2	ZY-09-49-2-RAC	17.909	66.800	15272	458308	2.03
3	ZY-09-49-2-RAC	21.189	105.100	12075	450187	1.99
4	ZY-09-49-2-RAC	24.997	202.800	160514	10992990	48.63

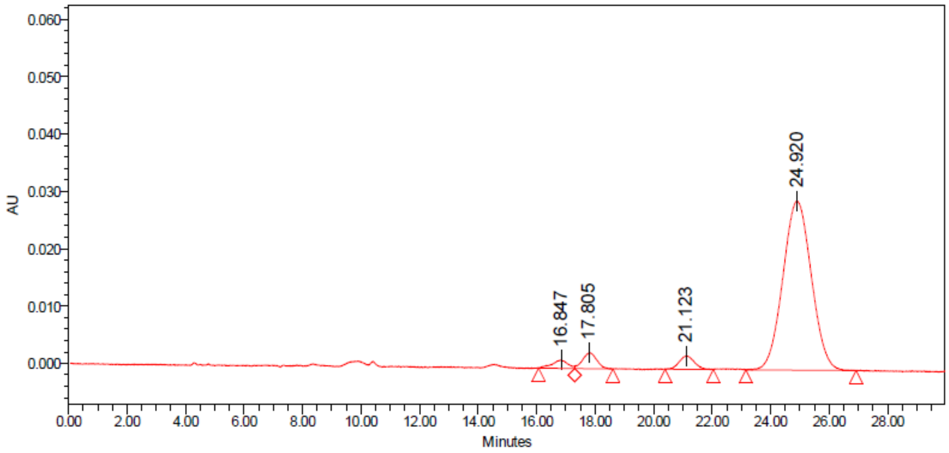

Peak Results

	SampleName	RT	Width (sec)	Height	Area	% Area
1	ZY-09-49-1-CH	16.847	73.800	1461	53023	2.38
2	ZY-09-49-1-CH	17.805	78.700	2788	93335	4.19
3	ZY-09-49-1-CH	21.123	98.800	2333	83319	3.74
4	ZY-09-49-1-CH	24.920	226.000	29486	1996061	89.68

Supplementary Fig. 154 HPLC analysis of *cis*-**7ad**

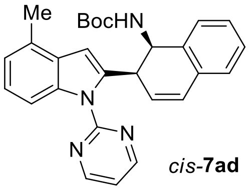

cis-**7ad**

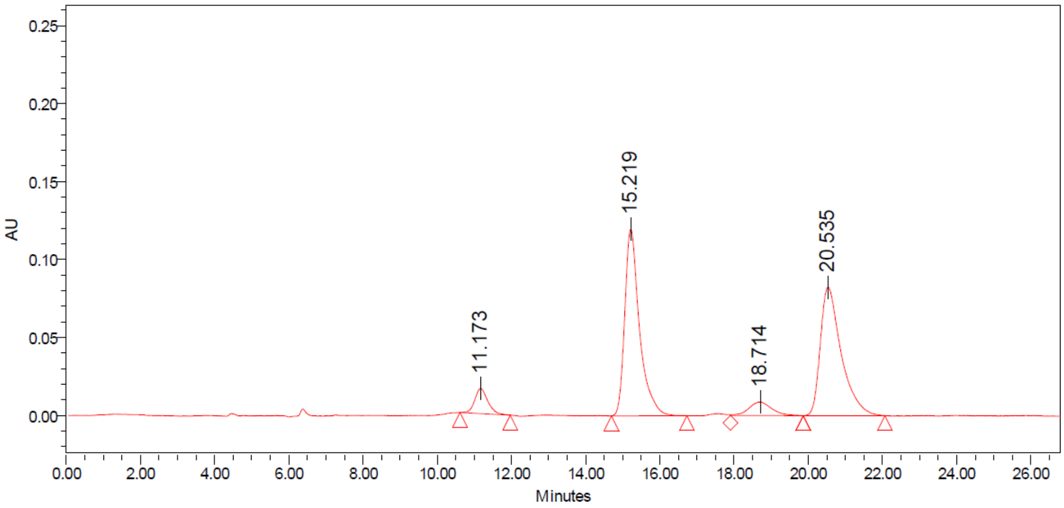

Peak Results

	SampleName	RT	Width (sec)	Height	Area	% Area
1	ZY-09-56-2-rac	11.173	81.000	16088	392462	5.39
2	ZY-09-56-2-rac	15.219	122.000	119739	3312759	45.47
3	ZY-09-56-2-rac	18.714	117.500	8622	367914	5.05
4	ZY-09-56-2-rac	20.535	132.000	82338	3212565	44.09

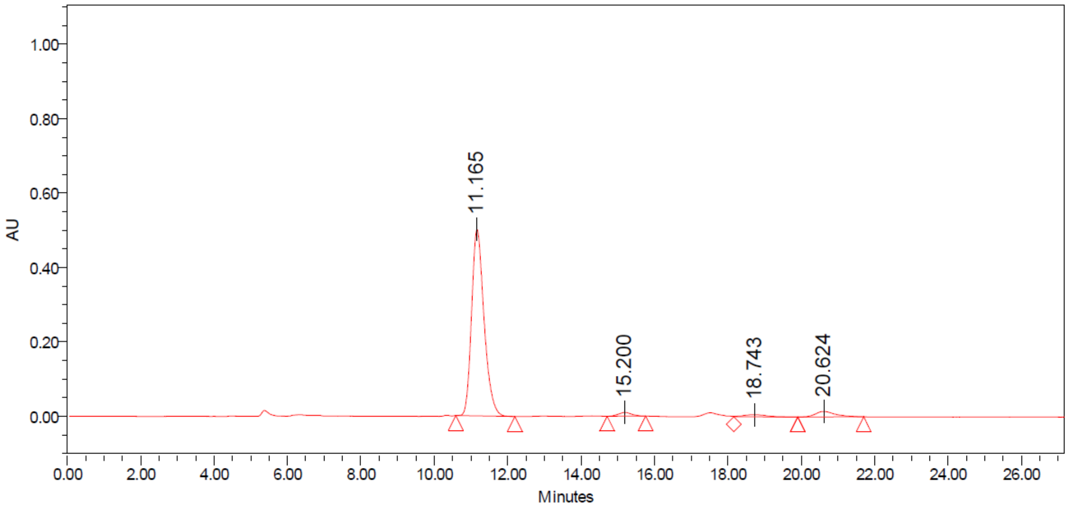

Peak Results

	SampleName	RT	Width (sec)	Height	Area	% Area
1	ZY-09-57-1-ch	11.165	96.600	501648	11883771	91.68
2	ZY-09-57-1-ch	15.200	62.900	10085	258781	2.00
3	ZY-09-57-1-ch	18.743	104.800	5836	254031	1.96
4	ZY-09-57-1-ch	20.624	107.700	14902	565928	4.37

Supplementary Fig. 155 HPLC analysis of *cis*-**7ae**

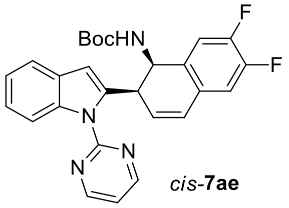

cis-**7ae**

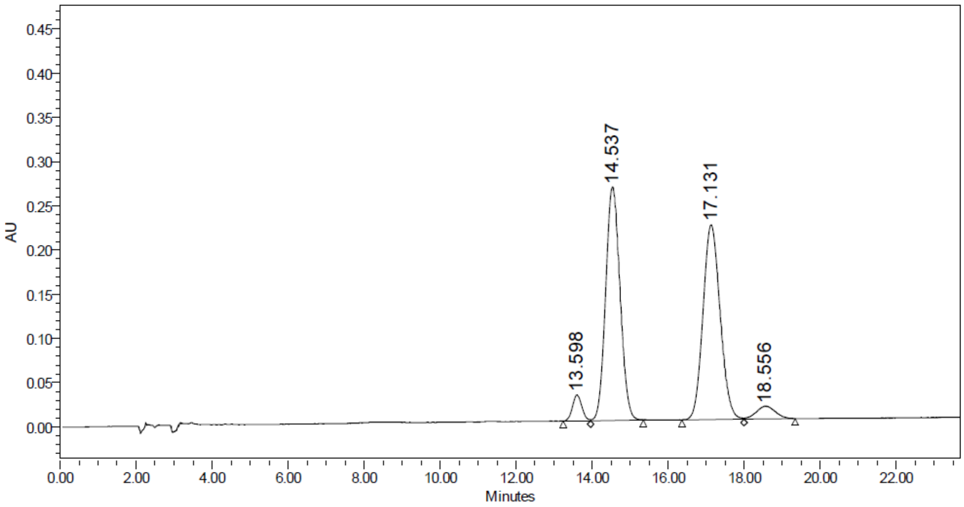

	RT	Peak Type	Height	Width (sec)	Area	% Area
1	13.598	Unknown	29337	43.300	505745	3.41
2	14.537	Unknown	263811	83.450	6887099	46.50
3	17.131	Unknown	220340	98.000	6906686	46.63
4	18.556	Unknown	14406	80.800	510880	3.45

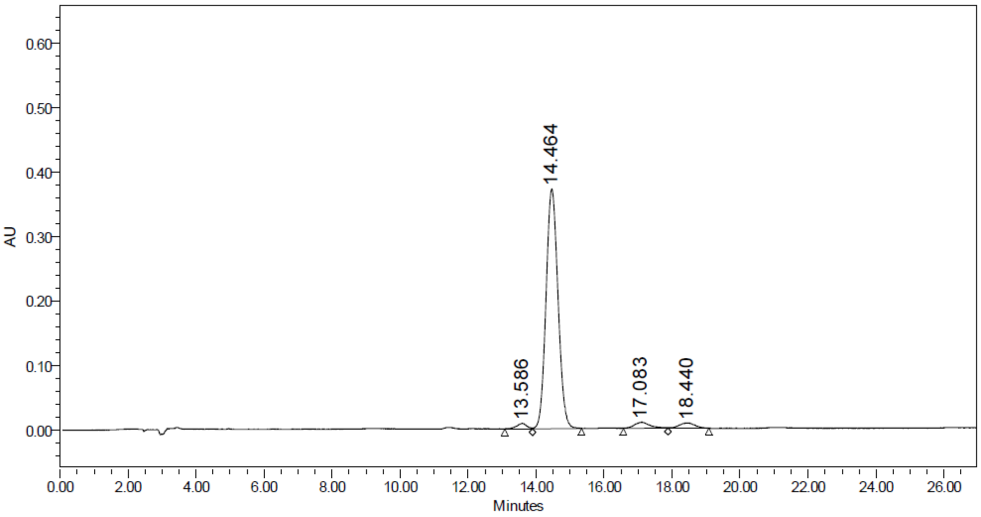

	RT	Peak Type	Height	Width (sec)	Area	% Area
1	13.586	Unknown	8220	49.250	163300	1.65
2	14.464	Unknown	372154	86.500	9168483	92.64
3	17.083	Unknown	9247	78.850	300193	3.03
4	18.440	Unknown	8307	72.000	264952	2.68

Supplementary Fig. 156 HPLC analysis of *cis*-**7af**

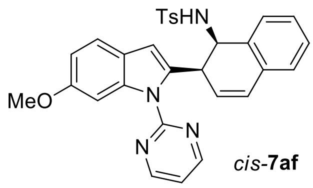

cis-**7af**

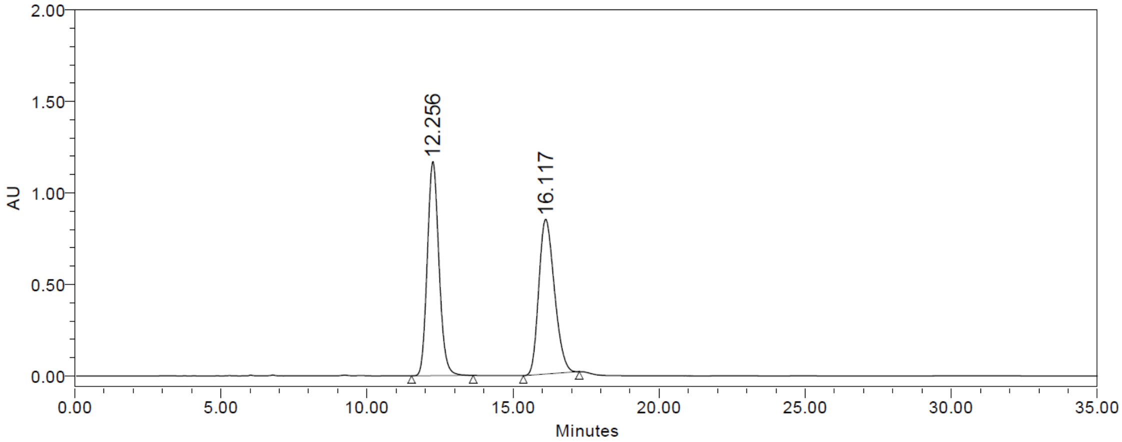

	RT	Area	% Area	Height
1	12.256	32455415	50.78	1169946
2	16.117	31453773	49.22	845455

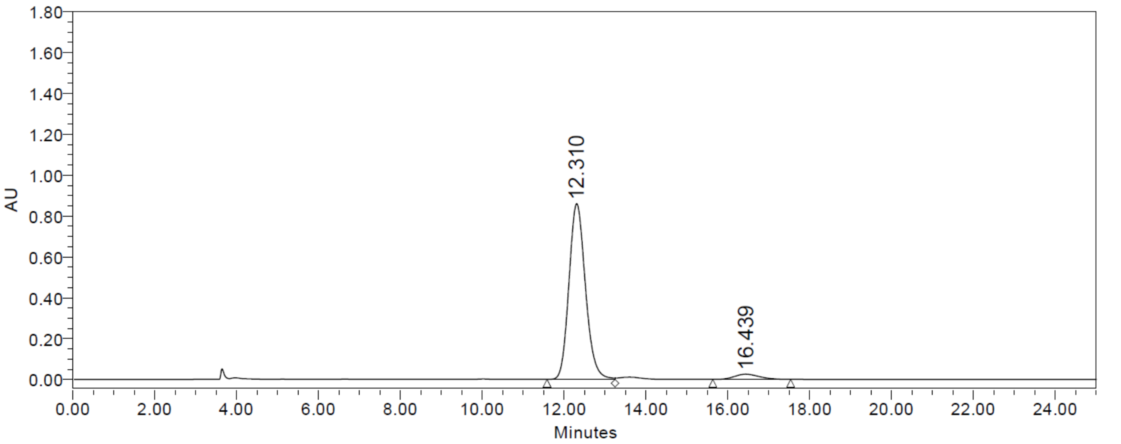

	RT	Area	% Area	Height
1	12.310	24043500	95.73	859615
2	16.439	1073191	4.27	25407

Supplementary Fig. 157 HPLC analysis of *trans*-**7aa**

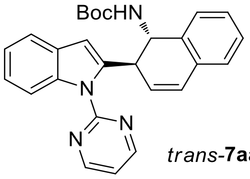

trans-**7aa**

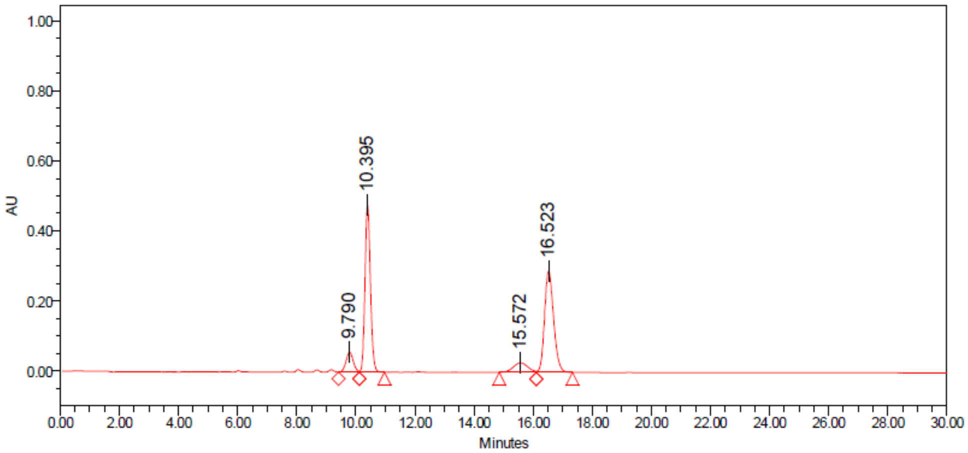

Peak Results

	SampleName	RT	Width (sec)	Height	Area	% Area
1	zy-08-62-rac	9.790	42.000	57063	968161	6.85
2	zy-08-62-rac	10.395	51.300	476892	6131092	43.35
3	zy-08-62-rac	15.572	75.500	27073	924101	6.53
4	zy-08-62-rac	16.523	73.700	288306	6120655	43.27

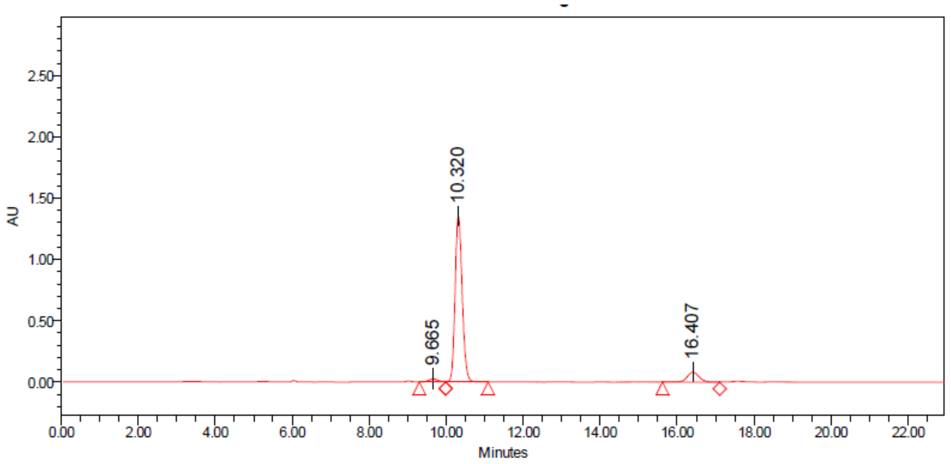

Peak Results

	SampleName	RT	Width (sec)	Height	Area	% Area
1	zy-09-5-2	9.665	41.300	26019	446889	2.29
2	zy-09-5-2	10.320	66.000	1352548	17405594	89.00
3	zy-09-5-2	16.407	89.200	80346	1704913	8.72

Supplementary Fig. 158 HPLC analysis of *trans*-**7ab**

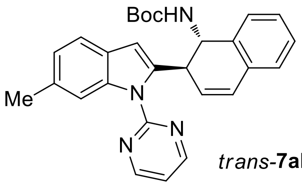

trans-**7ab**

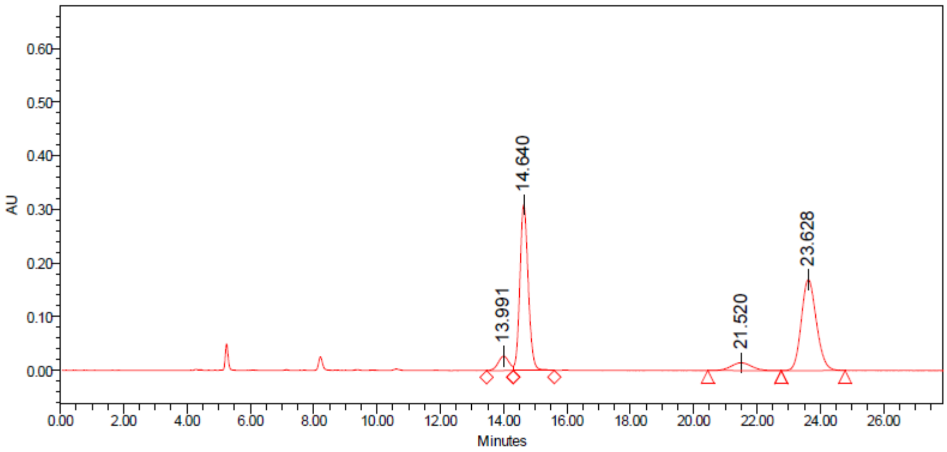

Peak Results

	SampleName	RT	Width (sec)	Height	Area	% Area
1	ZY-09-37-1-RAC	13.991	50.300	26908	629523	5.02
2	ZY-09-37-1-RAC	14.640	77.900	308716	5646665	45.06
3	ZY-09-37-1-RAC	21.520	139.100	14414	640512	5.11
4	ZY-09-37-1-RAC	23.628	120.900	169582	5613388	44.80

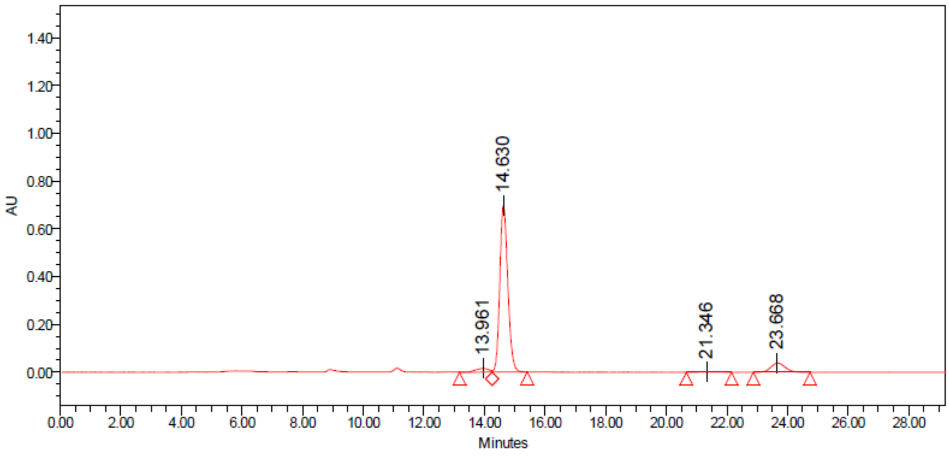

Peak Results

	SampleName	RT	Width (sec)	Height	Area	% Area
1	ZY-09-36-1-CH	13.961	64.400	15635	469581	3.22
2	ZY-09-36-1-CH	14.630	69.800	696677	12779610	87.76
3	ZY-09-36-1-CH	21.346	89.200	1659	50039	0.34
4	ZY-09-36-1-CH	23.668	112.200	38353	1262320	8.67

Supplementary Fig. 159 HPLC analysis of *trans*-**7ab'**

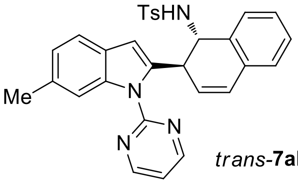

trans-**7ab'**

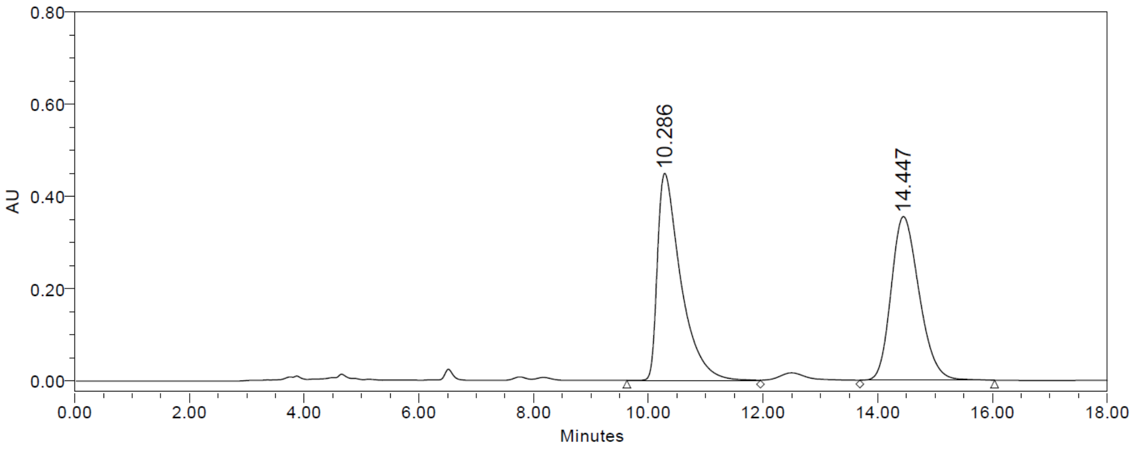

	RT	Area	% Area	Height
1	10.286	12733819	51.37	448858
2	14.447	12056300	48.63	354922

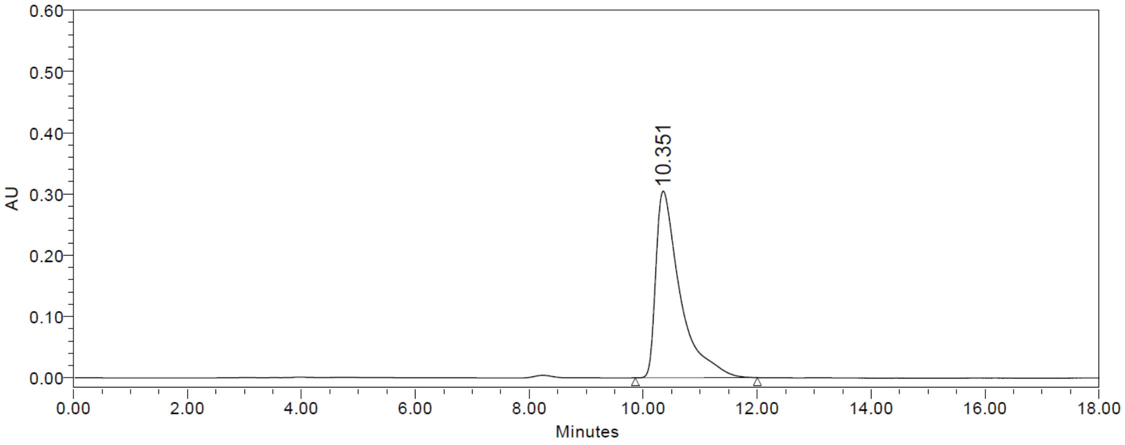

	RT	Area	% Area	Height
1	10.351	9051573	100.00	304860

Supplementary Fig. 160 HPLC analysis of *trans*-**7ac**

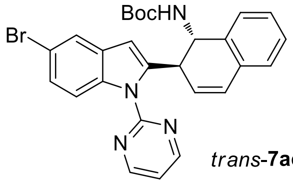

trans-**7ac**

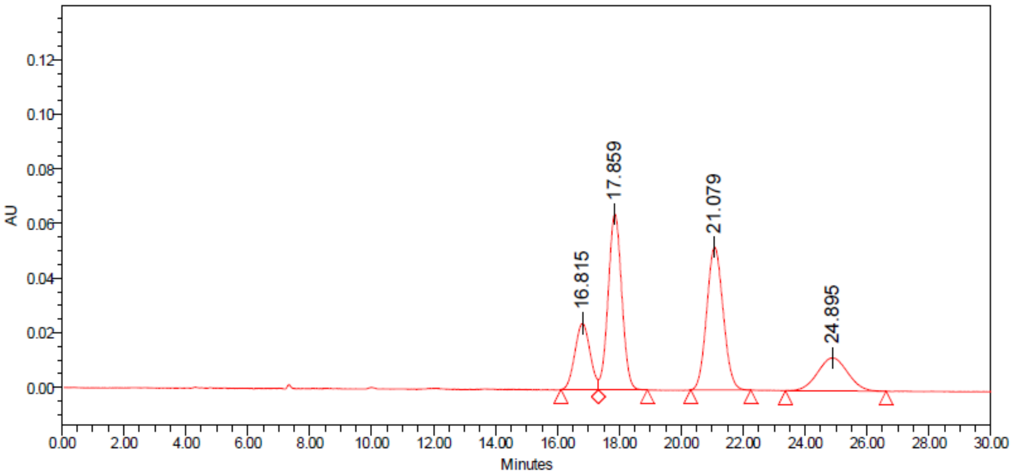

Peak Results

	SampleName	RT	Width (sec)	Height	Area	% Area
1	ZY-09-48-2-RAC	16.815	72.500	24228	822620	14.89
2	ZY-09-48-2-RAC	17.859	95.200	64259	1955427	35.40
3	ZY-09-48-2-RAC	21.079	116.600	52294	1928384	34.91
4	ZY-09-48-2-RAC	24.895	194.900	12124	818093	14.81

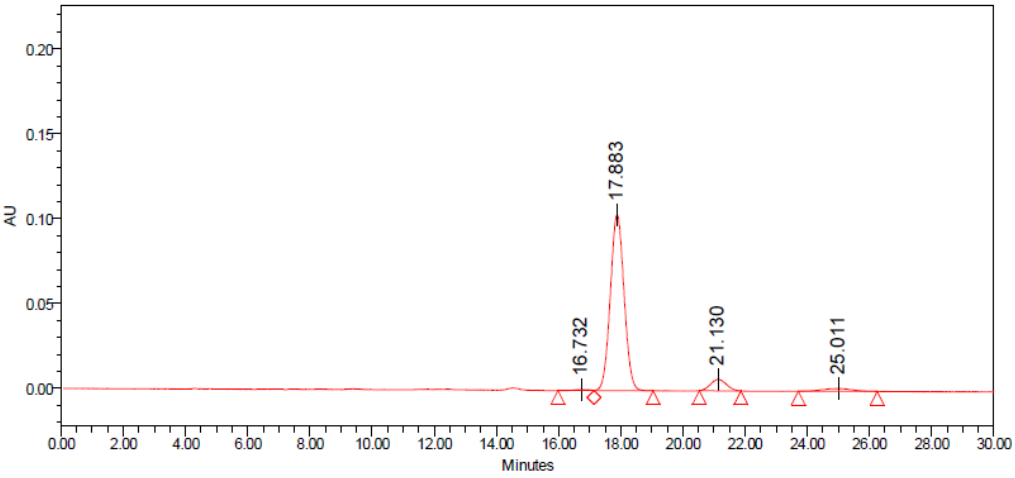

Peak Results

	SampleName	RT	Width (sec)	Height	Area	% Area
1	ZY-09-48-1-CH	16.732	69.200	595	19427	0.55
2	ZY-09-48-1-CH	17.883	114.200	103669	3178850	89.71
3	ZY-09-48-1-CH	21.130	80.300	6745	238176	6.72
4	ZY-09-48-1-CH	25.011	152.400	1666	107066	3.02

Supplementary Fig. 161 HPLC analysis of *trans*-**7ad**

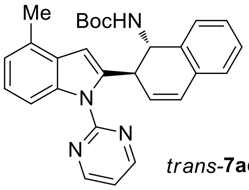

trans-**7ad**

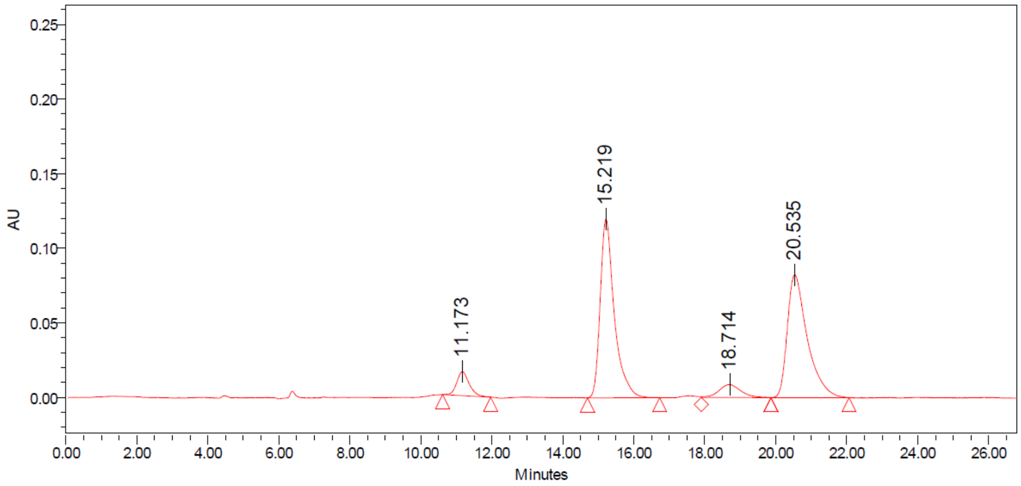

Peak Results

	SampleName	RT	Width (sec)	Height	Area	% Area
1	ZY-09-56-2-rac	11.173	81.000	16088	392462	5.39
2	ZY-09-56-2-rac	15.219	122.000	119739	3312759	45.47
3	ZY-09-56-2-rac	18.714	117.500	8622	367914	5.05
4	ZY-09-56-2-rac	20.535	132.000	82338	3212565	44.09

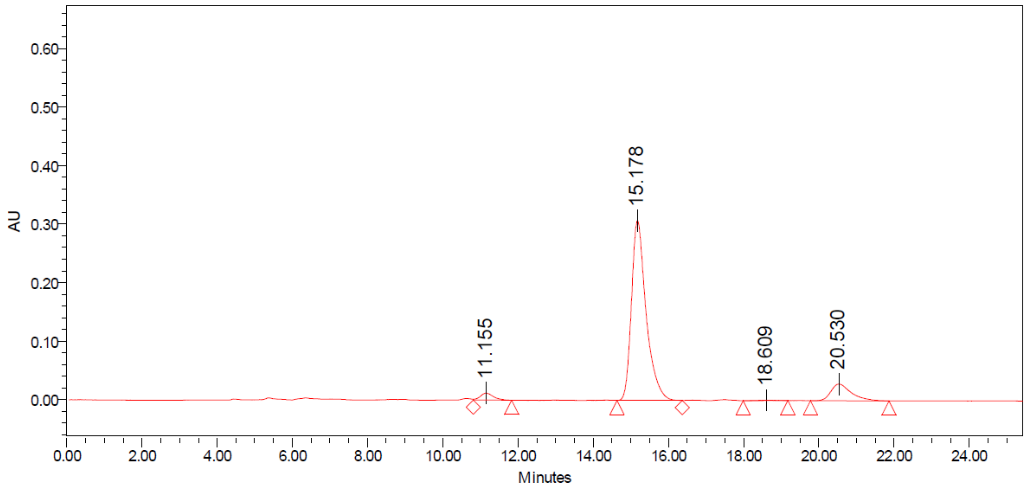

Peak Results

	SampleName	RT	Width (sec)	Height	Area	% Area
1	ZY-09-56-1-ch	11.155	61.300	11876	280750	2.91
2	ZY-09-56-1-ch	15.178	104.100	307068	8278482	85.78
3	ZY-09-56-1-ch	18.609	71.800	466	14708	0.15
4	ZY-09-56-1-ch	20.530	125.400	28475	1076600	11.16

Supplementary Fig. 162 HPLC analysis of *trans*-**7ae**

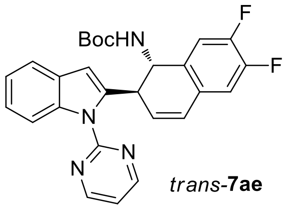

trans-**7ae**

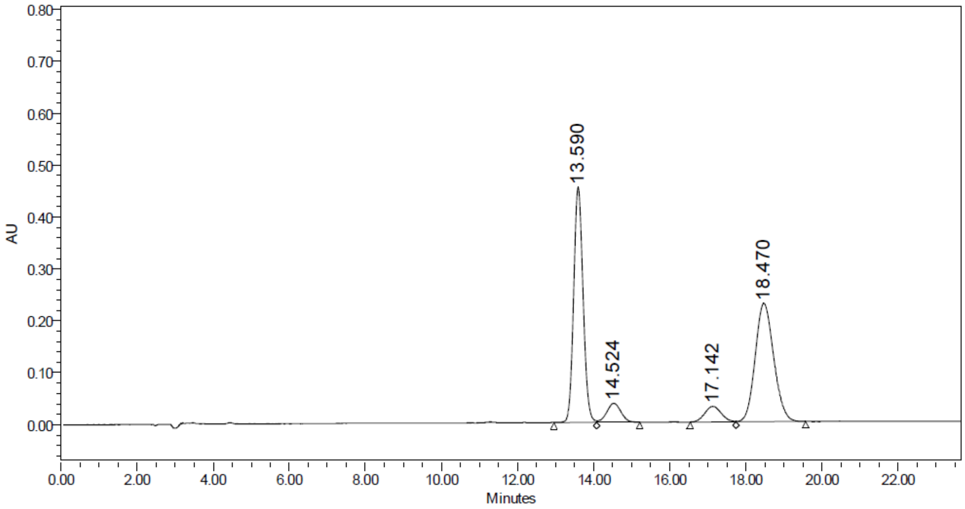

	RT	Peak Type	Height	Width (sec)	Area	% Area
1	13.590	Unknown	453633	67.300	7833791	44.87
2	14.524	Unknown	36760	67.850	959757	5.50
3	17.142	Unknown	30223	72.450	914189	5.24
4	18.470	Unknown	228215	109.550	7750194	44.39

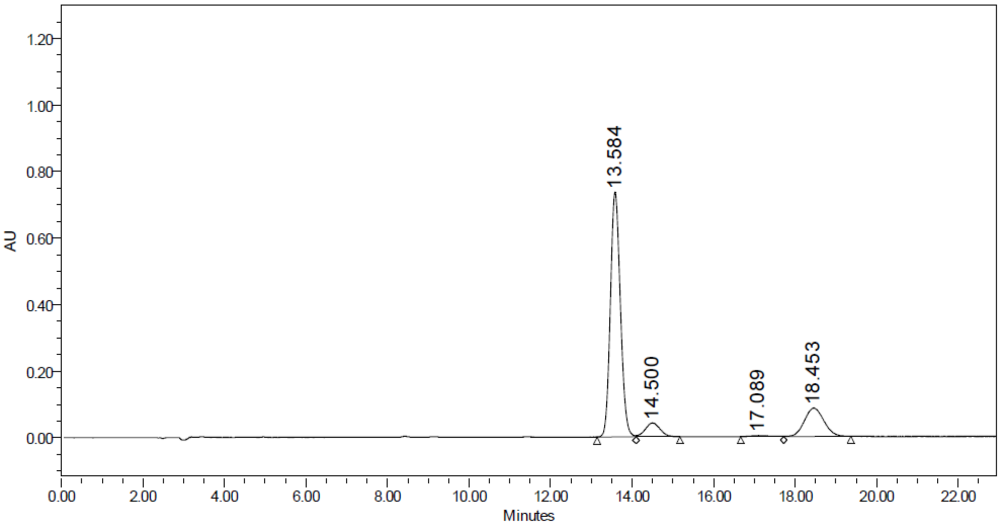

	RT	Peak Type	Height	Width (sec)	Area	% Area
1	13.584	Unknown	735719	57.400	12558996	75.81
2	14.500	Unknown	42006	64.250	1065043	6.43
3	17.089	Unknown	3013	63.350	95698	0.58
4	18.453	Unknown	85291	98.850	2847372	17.19

Supplementary Fig. 163 HPLC analysis of *trans*-**7af**

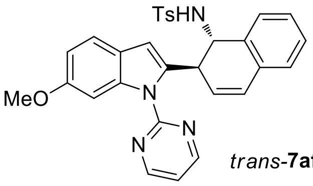

trans-**7af**

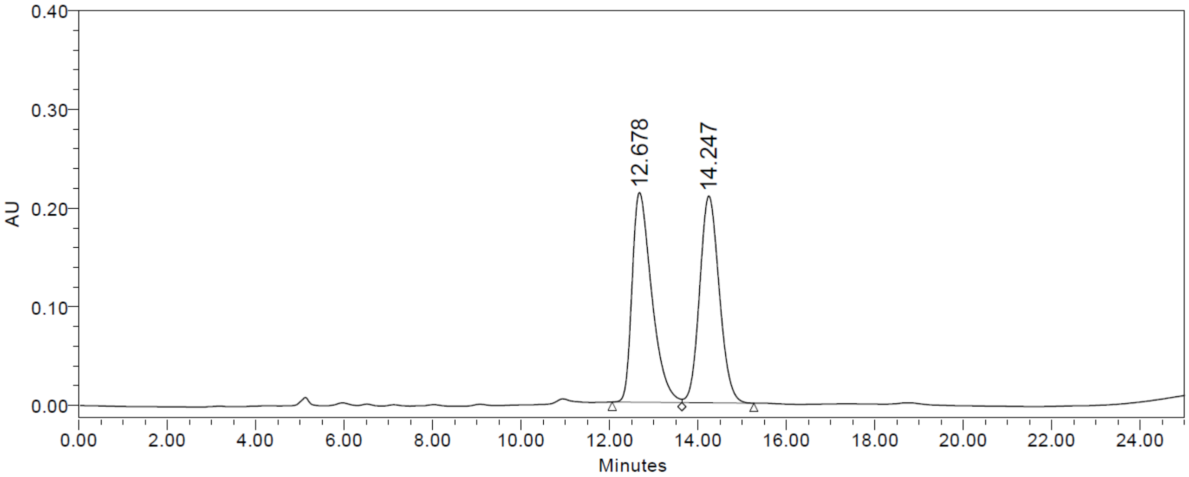

	RT	Area	% Area	Height
1	12.678	6552277	50.35	212517
2	14.247	6461530	49.65	209587

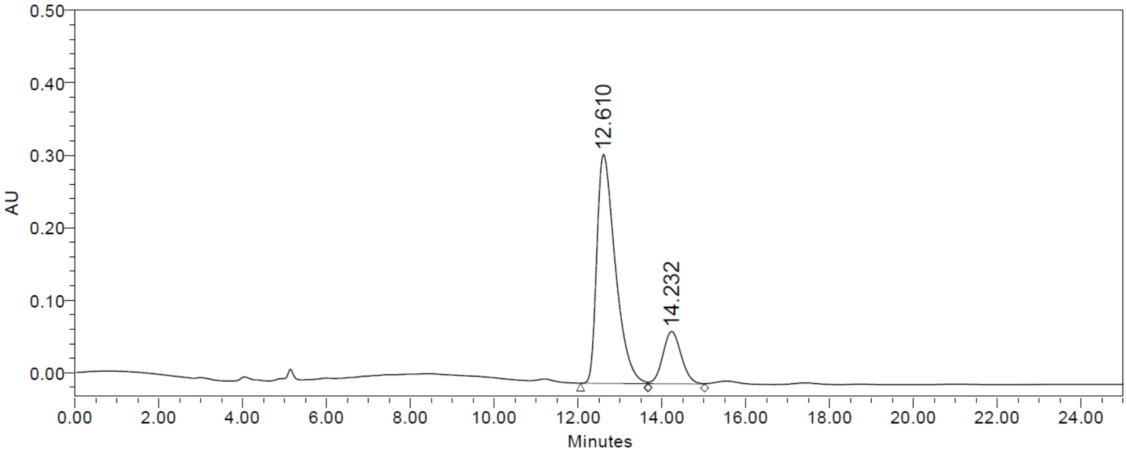

	RT	Area	% Area	Height
1	12.610	9680877	81.51	315831
2	14.232	2196046	18.49	72146

Supplementary Fig. 164 HPLC analysis of **4**

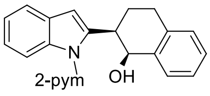

4

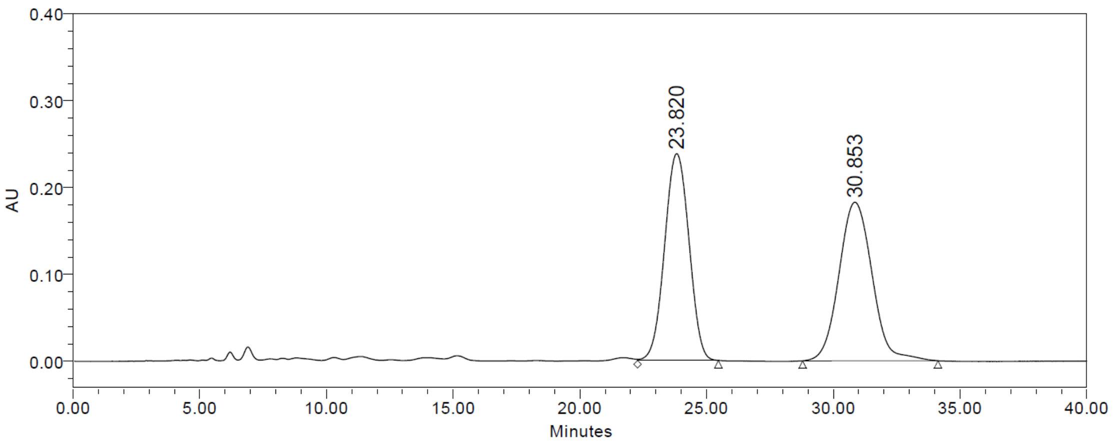

	RT	Area	% Area	Height
1	23.820	15963263	49.04	238063
2	30.853	16590149	50.96	182568

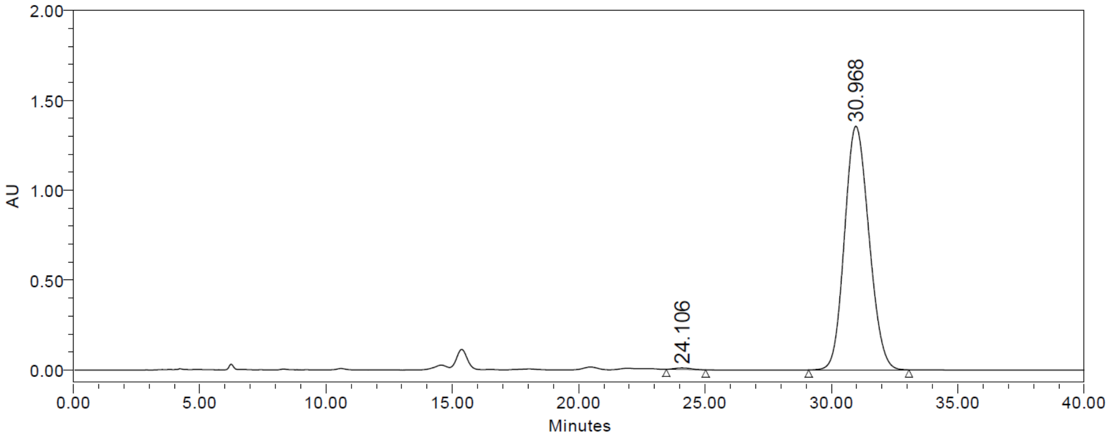

	RT	Area	% Area	Height
1	24.106	383360	0.42	8868
2	30.968	91056873	99.58	1355276

Supplementary Fig. 165 HPLC analysis of **5**

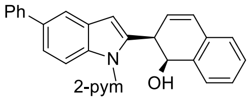

5

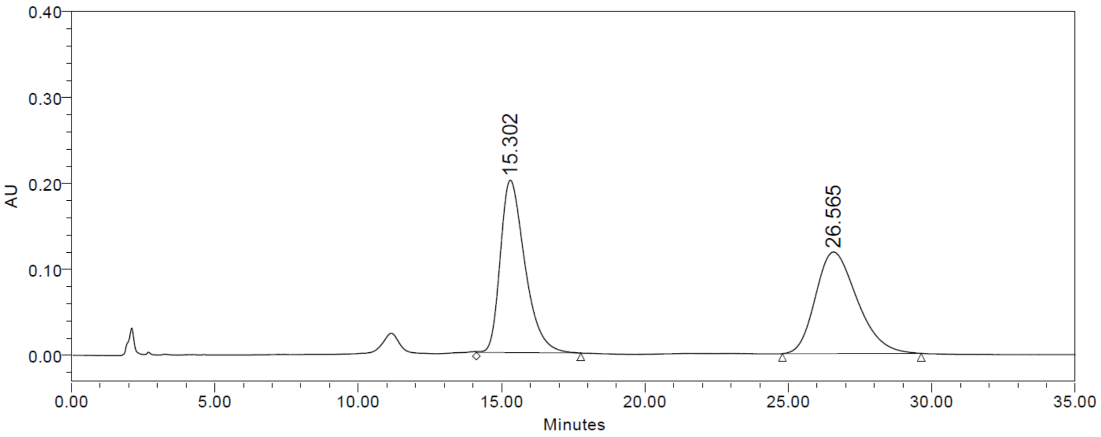

	RT	Area	% Area	Height
1	15.302	11996222	50.17	200744
2	26.565	11913363	49.83	118083

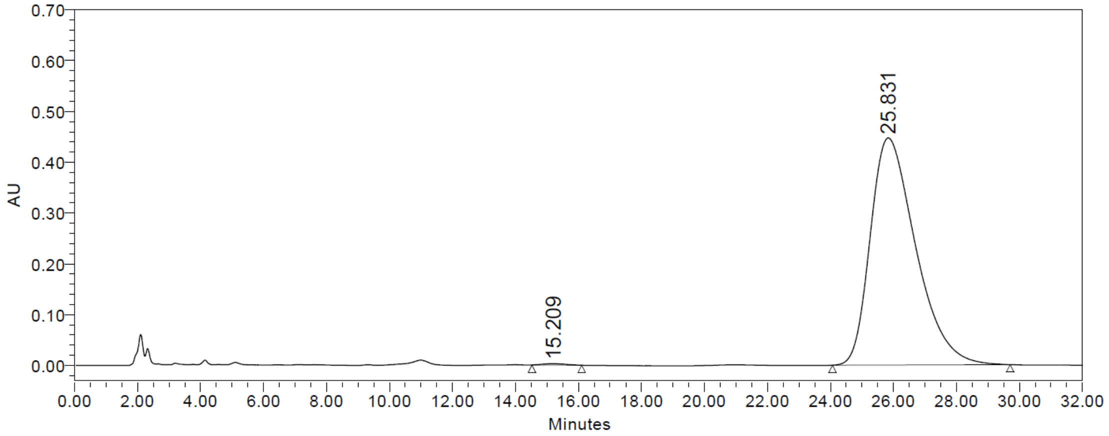

	RT	Area	% Area	Height
1	15.209	142111	0.32	2890
2	25.831	44701567	99.68	447041

Supplementary Fig. 166 HPLC analysis of **8**

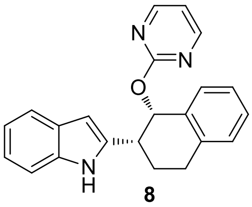

8

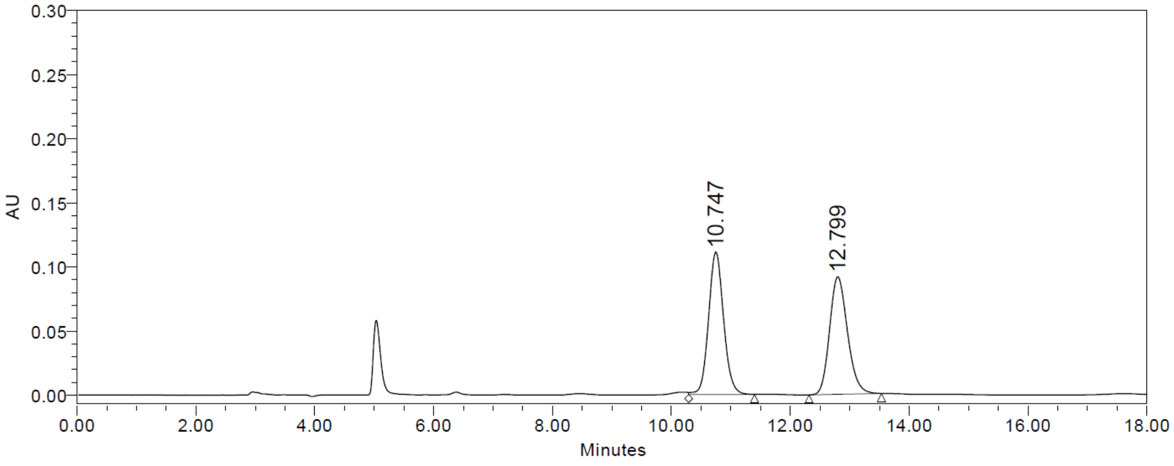

	RT	Area	% Area	Height
1	10.747	1957180	50.50	111094
2	12.799	1918228	49.50	91728

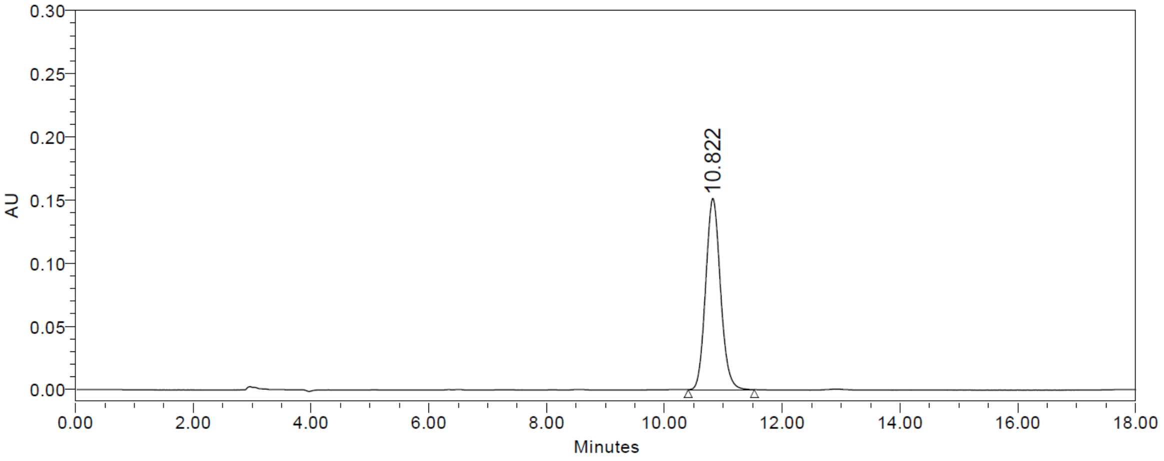

	RT	Area	% Area	Height
1	10.822	2645343	100.00	151313

2. References

1. K. Ozols, Y. S. Jang, N. Cramer, *J. Am. Chem. Soc.* **2019,** *141*, 5675-5680.
2. K. Muralirajan, S. Prakash, C.-H. Cheng, *Adv. Synth. Catal.* **2017,** *359*, 513-518.
3. a) Y.-H. Liu, P.-P. Xie, L. Liu, J. Fan, Z.-Z. Zhang, X. Hong, B.-F. Shi, *J. Am. Chem. Soc.* **2021**, *143*, 19112-19120; b) C. M. Rathbun, J. B. Johnson, *J. Am. Chem. Soc.* **2011**, *133*, 2031-2033; c) L. van Dijk, R. Ardkhean, M. Sidera, S. Karabiyikoglu, Ö. Sari, T. D. W. Claridge, G. C. Lloyd-Jones, R. S. Paton, S. P. Fletcher, *Nat. Catal.* **2021**, *4*, 284-292.
4. Gaussian 16, Revision A.03, M. J. Frisch, G. W. Trucks, H. B. Schlegel, G. E. Scuseria, M. A. Robb, J. R. Cheeseman, G. Scalmani, V. Barone, G. A. Petersson, H. Nakatsuji, X. Li, M. Caricato, A. V. Marenich, J. Bloino, B. G. Janesko, R. Gomperts, B. Mennucci, H. P. Hratchian, J. V. Ortiz, A. F. Izmaylov, J. L. Sonnenberg, D. Williams-Young, F. Ding, F. Lipparini, F. Egidi, J. Goings, B. Peng, A. Petrone, T. Henderson, D. Ranasinghe, V. G. Zakrzewski, J. Gao, N. Rega, G. Zheng, W. Liang, M. Hada, M. Ehara, K. Toyota, R. Fukuda, J. Hasegawa, M. Ishida, T. Nakajima, Y. Honda, O. Kitao, H. Nakai, T. Vreven, K. Throssell, J. A. Jr. Montgomery, J. E. Peralta, F. Ogliaro, M. J. Bearpark, J. J. Heyd, E. N. Brothers, K. N. Kudin, V. N. Staroverov, T. A. Keith, R. Kobayashi, J. K. Normand, Rendell, A. P. Raghavachari, J. C. Burant, S. S. Iyengar, J. Tomasi, M. Cossi, J. M. Millam, M. Klene, C. Adamo, R. Cammi, J. W. Ochterski, R. L. Martin, K. Morokuma, O. Farkas, J. B. Foresman, D. J. Fox, Gaussian, Inc., Wallingford CT, **2016**.
5. a) F. Neese, *WIREs Comput. Mol. Sci.* **2012**, *2*, 73-78; b) F. Neese, *WIREs Comput. Mol. Sci.* **2017**, *8*, e1327; c) F. Neese, F. Wennmohs, U. Becker, C. Riplinger, *J. Chem. Phys.* **2020**, *152*, 224108.
6. a) A. D. Becke, *J. Chem. Phys.* **1993**, *98*, 5648-5652; b) C. Lee, W. Yang, R. G. Parr, *Phys. Rev. B: Condens. Matter Mater. Phys.* **1988**, *37*, 785-789; c) P. J. Stephens, F. J. Devlin, C. F. Chabalowski, M. J. Frisch, *J. Phys. Chem.* **1994**, *98*, 11623-11627.
7. a) E. R. Johnson, A. D. Becke, *J. Chem. Phys.* **2005**, *123*, 024101; b) A. D. Becke, E. R. Johnson, *J. Chem. Phys.* **2005**, *122*, 154104; c) E. R. Johnson, A. D. Becke, *J. Chem. Phys.* **2006**, *124*, 174104; d) S. Grimme, J. Antony, S. Ehrlich, H. Krieg, *J. Chem. Phys.* **2010**, *132*, 154104; e) S. Grimme, S. Ehrlich, L. Goerigk, *J. Comput. Chem.* **2011**, *32*, 1456-1465.
8. a) F. Weigend, R. Ahlrichs, *Phys. Chem. Chem. Phys.* **2005**, *7*, 3297-3305; b) F. Weigend, *Phys. Chem. Chem. Phys.* **2006**, *8*, 1057-1065.
9. A. Hellweg, C. Hattig, S. Hofener, Klopper, W. *Theor. Chem. Acc.* **2007**, *117*, 587-597.
10. F. Weigend, *J. Comput. Chem.* **2008**, *29*, 167-175.
11. W. Humphrey, A. Dalke, K. Schulten, *J. Mol. Graph.* **1996**, *14*, 33-38.
12. X. Yang, G. Zheng, X. Li, *Angew. Chem. Int. Ed.* **2019**, *58*, 322-326.
13. L. Kong, S. Yu, G. Tang, H. Wang, X. Zhou, X. Li, *Org. Lett.* **2016**, *18*, 3802-3805.
14. X. Zhou, Y. Luo, L. Kong, Y. Xu, G. Zheng, Y. Lan, X. Li, *ACS Catal.* **2017**, *7*, 7296-7304.
