# Supplementary material for: Cobalt(III)-catalyzed asymmetric ring-opening of 7-oxabenzonorbornadienes via indole C–H functionalization
Source: Nat Commun. 2023 Feb 25;14:1094. doi: 10.1038/s41467-023-36723-6 (PMC9968317; doi:10.1038/s41467-023-36723-6)
Supplement: Supplementary file 3 — Supplementary Data 1 [file 41467_2023_36723_MOESM3_ESM.pdf]

Cartesian coordinates of the calculated structures

Pivalate anion
Opt @ B3LYP-D3(BJ)/def2-SVP in gas phase
SCF Done: E(RB3LYP) = -346.223139982 a.u.
Zero-point correction = 0.132114 Hartree/Particle
Sum of electronic and thermal Free Energies = -346.123239 a.u.
Sp @ RI-PWPB95-D3(BJ)/def2-TZVPP in 2,2,2-trifluoroethanol
FINAL SINGLE POINT ENERGY = -346.455611582580 a.u.

```
------------------------------------------------------------------
C,0,9.2800277519,2.5385165248,0.0027312148
C,0,9.7864704332,3.2891132152,-1.2359813602
H,0,9.3855584814,2.8609052349,-2.176497029
H,0,9.4885717343,4.3455070718,-1.1608296303
H,0,10.8886462782,3.2536245305,-1.2923511192
C,0,7.7459464561,2.5669733501,0.0138946766
H,0,7.3134530215,2.1277785774,-0.9073770407
H,0,7.3496898818,2.001061254,0.8752601418
H,0,7.4082978987,3.6092454104,0.1137742349
C,0,9.7894281891,1.0972769991,0.0014679078
H,0,10.889619163,1.0789139194,0.0251941732
H,0,9.4508668964,0.5694892355,0.9059990323
H,0,9.4404161562,0.5395884082,-0.8909982711
C,0,9.7915551492,3.3302291995,1.2946092883
O,0,10.5072016971,2.6866987211,2.0899550124
O,0,9.4061283019,4.5199922881,1.3531936084
```

1a
Opt @ B3LYP-D3(BJ)/def2-SVP in gas phase
SCF Done: E(RB3LYP) = -626.561300409 a.u.
Zero-point correction = 0.187837 Hartree/Particle
Sum of electronic and thermal Free Energies = -626.410228 a.u.
Sp @ RI-PWPB95-D3(BJ)/def2-TZVPP in 2,2,2-trifluoroethanol
FINAL SINGLE POINT ENERGY = -626.747664218624 a.u.

```
------------------------------------------------------------------
C,0,1.2008728743,2.5195536486,1.4497749554
C,0,1.0563059286,2.2508624908,0.0593640125
C,0,1.2313198864,3.2544933481,-0.9006799349
C,0,1.5539860735,4.5340327282,-0.446111053
C,0,1.7004162115,4.8158335769,0.9244534689
C,0,1.5258136427,3.8156114605,1.8776315034
C,0,0.9566906566,1.2823654006,2.1537041961
C,0,0.6807866211,0.3275604012,1.2224255683
H,0,1.1173838846,3.0325786011,-1.9577644117
H,0,1.695567645,5.3342835126,-1.1765385019
H,0,1.9536789939,5.8302358747,1.2416426529
H,0,1.638737384,4.0307071322,2.9428921924
H,0,0.9856456599,1.1316950846,3.2307194351
N,0,0.7338067028,0.8864378072,-0.063108485
C,0,0.4953205971,0.1513927634,-1.2196082161
C,0,0.3392955271,0.0560345096,-3.4848909467
C,0,-0.0240688293,-1.8597125167,-2.1459040464
C,0,0.0287605173,-1.30294287,-3.4270760134
H,0,0.4036062239,0.5806704113,-4.4451036296
H,0,-0.2605693672,-2.9202887932,-2.0007591695
```

```
H,0,-0.1605664841,-1.8933363424,-4.3239151909
N,0,0.572140099,0.7849422088,-2.3951097643
N,0,0.2054557354,-1.1473013515,-1.0483631922
H,0,0.443082096,-0.7260165966,1.3215014407
----------------------------------------------------------------
```

2a

```
Opt @ B3LYP-D3(BJ)/def2-SVP in gas phase
SCF Done: E(RB3LYP) = -460.737316559 a.u.
Zero-point correction = 0.151988 Hartree/Particle
Sum of electronic and thermal Free Energies = -460.617111 a.u.
Sp @ RI-PWPB95-D3(BJ)/def2-TZVPP in 2,2,2-trifluoroethanol
FINAL SINGLE POINT ENERGY = -460.889448353949 a.u.
----------------------------------------------------------------
C,0,6.8781074539,-0.3678137333,-3.6661313124
C,0,7.3806566157,0.5477145269,-2.7432148286
C,0,6.7536768438,0.732982801,-1.4921206408
C,0,5.6343590718,-0.0267516072,-1.2116910895
C,0,5.1267303882,-0.9515991263,-2.1440101466
C,0,5.7289285609,-1.1337878408,-3.3739801788
C,0,4.6631579554,-0.1357284599,-0.0228167862
C,0,3.3251213256,0.4683817521,-0.4992697753
C,0,2.8432405211,-0.4099467149,-1.3843024007
C,0,3.8920697285,-1.5408916646,-1.4389721721
O,0,4.3585304396,-1.5375175479,-0.0812185675
H,0,7.3820184035,-0.4982665163,-4.6268915088
H,0,8.2741054775,1.1269903679,-2.9885585805
H,0,7.1538477827,1.4522478992,-0.7728877906
H,0,5.3408523734,-1.8505878654,-4.1022404104
H,0,5.0368114462,0.1608624599,0.9639614824
H,0,2.9343694252,1.4464446876,-0.2201845293
H,0,1.957592993,-0.3338362929,-2.0143544356
H,0,3.5570831937,-2.5358571251,-1.7539243288
```

INT-0

```
Opt @ B3LYP-D3(BJ)/def2-SVP in gas phase
SCF Done: E(RB3LYP) = -3781.09671297 a.u.
Zero-point correction = 0.882223 Hartree/Particle
Sum of electronic and thermal Free Energies = -3780.298646 a.u.
Sp @ RI-PWPB95-D3(BJ)/def2-TZVPP in 2,2,2-trifluoroethanol
FINAL SINGLE POINT ENERGY = -3781.953623880364 a.u.
----------------------------------------------------------------
Co,0,1.9548982766,0.7138051871,-0.2459496807
O,0,-2.0795474137,3.1447163952,2.0994302483
O,0,0.1209160178,-1.22704935,-3.4960177092
C,0,1.908316384,2.7813512155,-0.115825926
C,0,1.0638018703,2.2152094784,0.9032596342
C,0,0.0318710144,1.4640429707,0.3027327305
C,0,0.2205422599,1.5235976357,-1.1331940674
C,0,1.3547376588,2.3523565269,-1.3599562016
C,0,3.0198442097,3.7896690243,0.0953083018
C,0,2.3395812478,5.1787238298,0.0385284509
C,0,3.6814324128,3.6043223558,1.4711553901
C,0,4.0802544515,3.7032476358,-1.0138549498
C,0,-1.115109594,0.8248166065,1.0174420798
C,0,-2.4034631515,1.4354666916,0.5273868417
```

C,0,-2.8762392371,2.6459390562,1.1191147815
C,0,-4.0554545243,3.2166084532,0.6854565685
C,0,-4.7943645247,2.6228393806,-0.376003006
C,0,-6.0064493532,3.1987097873,-0.8449164539
C,0,-6.7053520131,2.6271991211,-1.8872820585
C,0,-6.2209405739,1.4533746401,-2.5132655587
C,0,-5.0488936487,0.8684625722,-2.0811594788
C,0,-4.3106581532,1.4276389693,-1.0026613944
C,0,-3.0917531925,0.8498202224,-0.5190322413
C,0,-2.5304167487,-0.3781672075,-1.1573609182
C,0,-3.1285303281,-1.6539125615,-0.8955328022
C,0,-4.1857677413,-1.8167878984,0.0407370588
C,0,-4.711767185,-3.0648315071,0.301784572
C,0,-4.2097630771,-4.2064724406,-0.3690749105
C,0,-3.1901279098,-4.0793557449,-1.2893429608
C,0,-2.6199148005,-2.808385025,-1.5720961956
C,0,-1.5491615351,-2.6601427791,-2.4935823611
C,0,-0.945436053,-1.4328385282,-2.6720071058
C,0,-1.4177054985,-0.2729067591,-1.9810289071
C,0,-0.7178796041,1.0481255311,-2.2119565574
C,0,-2.4925072327,4.3101167848,2.7850098755
C,0,0.3739111609,-2.1587109421,-4.5308905341
H,0,1.206920636,2.2986980988,1.9772549347
H,0,1.7864423211,2.5422598504,-2.3413602784
H,0,1.8727431332,5.3560688429,-0.942524307
H,0,3.0870020694,5.9685800815,0.2104445544
H,0,1.5599851742,5.2716078795,0.8101721526
H,0,2.9639354213,3.7656561902,2.2901103244
H,0,4.4876061356,4.3422089088,1.5971226223
H,0,4.1080000189,2.5982148271,1.5796388682
H,0,4.590741831,2.7313474525,-1.0184553947
H,0,4.8395349692,4.4842474597,-0.8608165089
H,0,3.6463761307,3.8609537269,-2.0126577615
H,0,-1.0001521355,0.9654350328,2.096208363
H,0,-1.1478540158,-0.2458406427,0.8108702325
H,0,-4.4407443295,4.1313352207,1.1345816881
H,0,-6.3767115763,4.1076685314,-0.3645106031
H,0,-7.6356447445,3.0817133127,-2.2351308731
H,0,-6.7791646714,1.010781759,-3.3409698748
H,0,-4.6787081245,-0.036365192,-2.5647834128
H,0,-4.5681713868,-0.9399764993,0.5639037859
H,0,-5.5192575677,-3.1750644215,1.0286544148
H,0,-4.6349617891,-5.1899150282,-0.1569528128
H,0,-2.8044015699,-4.9586538618,-1.8115958315
H,0,-1.1997965881,-3.5404394708,-3.0319895215
H,0,-0.166932614,1.0106134989,-3.15927158
H,0,-1.4805008226,1.8395301894,-2.3110209748
H,0,-2.5778575635,5.1723034566,2.1010423092
H,0,-1.7222790315,4.5207697897,3.5378097125
H,0,-3.4611496886,4.1573211069,3.2909023761
H,0,-0.5303541546,-2.3291223007,-5.1384493574
H,0,0.7228066146,-3.1284647114,-4.1389489957
H,0,1.1622482426,-1.7239960463,-5.1587018204
C,0,-0.2927117937,-0.9014523412,4.0424758111
C,0,-0.4679110301,-1.6666130088,2.8613505424
C,0,-1.6353494559,-2.3940982333,2.6145832054

```
C,0,-2.6292950406,-2.3565203372,3.5925390118
C,0,-2.4660961985,-1.6221403697,4.7820785385
C,0,-1.3042541772,-0.8908096652,5.0157017743
C,0,0.9796505855,-0.2224642181,3.9302909352
C,0,1.5206053427,-0.54082257,2.7210364268
H,0,-1.7693618024,-2.9701386216,1.7023147431
H,0,-3.552083463,-2.9155338538,3.4251269225
H,0,-3.2638481471,-1.6240264387,5.5277905866
H,0,-1.1823992293,-0.3102522863,5.9326103304
H,0,1.4469655259,0.4075344562,4.684340986
N,0,0.6563912096,-1.4174646977,2.0399694276
C,0,0.8829599697,-1.9816004197,0.8009636126
C,0,0.7398712095,-3.8731403715,-0.4784337469
C,0,1.9275230514,-1.9846134075,-1.2548977179
C,0,1.5891790603,-3.3146577063,-1.4359015106
H,0,0.3178124446,-4.8767224731,-0.5966671318
H,0,2.4976964891,-1.4363820937,-2.00104715
H,0,1.9191817242,-3.8645893457,-2.315908164
N,0,0.4183286738,-3.2172018817,0.62501398
N,0,1.5529297077,-1.2837630485,-0.1593028247
H,0,2.4813254294,-0.2821884148,2.2892890637
C,0,4.2468518459,0.1842501345,-0.3686349174
O,0,3.6654179754,0.4051301316,0.7397055896
O,0,3.5105176607,0.3242098337,-1.3997936797
C,0,5.6850946178,-0.278820682,-0.4458117029
C,0,5.6632293291,-1.7898954007,-0.1139296975
H,0,5.0559969119,-2.3503653921,-0.8419154756
H,0,6.6885641191,-2.1875245282,-0.1502064669
H,0,5.2578137885,-1.9696408703,0.8926990002
C,0,6.2398891763,-0.0498579425,-1.8581665773
H,0,6.2442655465,1.0192900445,-2.1187457353
H,0,7.2752019129,-0.4177340751,-1.9118720598
H,0,5.6442123623,-0.5789754997,-2.6152508417
C,0,6.5156377793,0.4792362318,0.6039800186
H,0,6.1131502571,0.3280930259,1.6152039358
H,0,7.554285405,0.1172914557,0.5845726518
H,0,6.5291574518,1.5603551905,0.3968168913
```

TS-1

Opt @ B3LYP-D3(BJ)/def2-SVP in gas phase
SCF Done: E(RB3LYP) = -3781.09034234 a.u.
Imaginary frequency = -150.8991 cm^{-1}
Zero-point correction = 0.878674 Hartree/Particle
Sum of electronic and thermal Free Energies = -3780.291667 a.u.
Sp @ RI-PWPB95-D3(BJ)/def2-TZVPP in 2,2,2-trifluoroethanol
FINAL SINGLE POINT ENERGY = -3781.940237444023 a.u.

```
Co,0,-1.3242410345,-0.9499365684,0.4858259253
C,0,-4.5471677413,0.1528885614,-1.926220281
C,0,-5.0941916752,-0.3573110831,-0.710374009
C,0,-6.4385310045,-0.7267272304,-0.5956999986
C,0,-7.2313861617,-0.5751243521,-1.7309870151
C,0,-6.7141372121,-0.0676286413,-2.9449853217
C,0,-5.3811855222,0.3013914766,-3.0537960897
C,0,-3.1624740712,0.4136991627,-1.6960331978
```

C,0,-2.842698049,0.0889254251,-0.3808706605
H,0,-6.8374810803,-1.103208958,0.3440280931
H,0,-8.2871664409,-0.8500560803,-1.6790859116
H,0,-7.3789064861,0.0366666724,-3.8045967898
H,0,-4.9803477204,0.69545661,-3.9898783075
H,0,-2.4579097199,0.8534124714,-2.3978461346
N,0,-4.0522819626,-0.4060726333,0.2071443909
C,0,-3.9814224238,-0.9818250785,1.4466456659
C,0,-4.8767635766,-1.73830492,3.3875943889
C,0,-2.5201892664,-1.6889447521,3.0906842073
C,0,-3.6010347919,-1.9493405399,3.9257660651
H,0,-5.7766664182,-1.9677629202,3.9674423538
H,0,-1.484406894,-1.8440218238,3.3976629684
H,0,-3.4582598268,-2.3194621571,4.9405532727
N,0,-5.0670645493,-1.2438334185,2.1645216091
N,0,-2.7118381321,-1.2500268176,1.841358733
H,0,-2.3471983536,1.008282173,0.2905363819
C,0,-1.0797047328,1.5093913115,1.9235268188
O,0,-2.0045382123,2.0012421937,1.2352512983
O,0,-0.5854509821,0.3543048234,1.7038785342
C,0,-0.572836634,2.2552489999,3.1654404236
C,0,0.8857535893,1.8902411236,3.4713902963
H,0,0.9978596463,0.8129156358,3.6552239207
H,0,1.220866778,2.429721776,4.3699728494
H,0,1.5509518385,2.1696430558,2.6434671274
C,0,-1.4888365238,1.7781527741,4.3162907125
H,0,-2.5442139735,2.0115057763,4.1078470756
H,0,-1.2031222555,2.2848218174,5.2507575734
H,0,-1.3927967247,0.6931773239,4.4773216646
C,0,-0.7275666789,3.7696107566,2.9774000704
H,0,-0.0794441293,4.1313418698,2.1682996146
H,0,-0.4373474783,4.2892136515,3.9029755147
H,0,-1.7654708173,4.0360868858,2.7358383781
O,0,3.3565605078,-3.492527574,0.7902191653
O,0,-0.6488640894,2.2749731825,-1.5513833427
C,0,-1.2375712788,-2.8801068467,-0.3599303913
C,0,-0.230530518,-2.7321086698,0.6620106412
C,0,0.6658199224,-1.6981205481,0.2999872843
C,0,0.1908731209,-1.1199796725,-0.93360466
C,0,-0.9440593467,-1.8922010735,-1.3373304572
C,0,-2.2462278595,-4.0059071535,-0.4948019474
C,0,-1.4634749692,-5.1988036383,-1.0941171202
C,0,-2.8353088213,-4.4277535097,0.8614091339
C,0,-3.3875651555,-3.6154306654,-1.4463769123
C,0,1.8502389559,-1.2403661466,1.0920096508
C,0,3.0841021894,-1.2300448487,0.2193388045
C,0,3.8416347722,-2.4355021281,0.086040892
C,0,4.9676526123,-2.4704861364,-0.7103560018
C,0,5.3698806118,-1.3166563757,-1.4382364104
C,0,6.5178182936,-1.3341903972,-2.2764888801
C,0,6.8851647924,-0.2173641844,-2.996958987
C,0,6.1163163882,0.9685515341,-2.9154777381
C,0,4.9986397496,1.0185880538,-2.1086891736
C,0,4.6001801587,-0.1119850343,-1.3441184216
C,0,3.4475341041,-0.0982429859,-0.490549829
C,0,2.6048683495,1.1304601645,-0.3883950284

C,0,3.0813791183,2.2613754886,0.3522959113
C,0,4.3144642786,2.2450439158,1.0596230681
C,0,4.7312664125,3.3458161003,1.779020028
C,0,3.9357654213,4.5165326259,1.8179219033
C,0,2.7342601497,4.5623467988,1.1429723822
C,0,2.2718391938,3.4398676672,0.4034370674
C,0,1.0144171663,3.4537955653,-0.2561984807
C,0,0.5561262017,2.3353169851,-0.9211705075
C,0,1.3593956334,1.1530399035,-0.9984258257
C,0,0.8416918958,-0.0447733673,-1.764074116
C,0,4.0605223573,-4.716981284,0.7492215083
C,0,-1.454164346,3.4425857219,-1.5716904799
H,0,-0.1400386766,-3.329860908,1.5667975991
H,0,-1.5152720584,-1.715376756,-2.2439035832
H,0,-1.0134477277,-4.9310519965,-2.0620428156
H,0,-2.1399261446,-6.0526126593,-1.2541708963
H,0,-0.6549266138,-5.5233463073,-0.4213052231
H,0,-2.0544630632,-4.6359808335,1.6081952729
H,0,-3.4234756581,-5.3491155021,0.7382976541
H,0,-3.5083831304,-3.6620912983,1.2686302203
H,0,-3.9228101179,-2.7227295129,-1.0948680523
H,0,-4.1147164536,-4.4377525522,-1.5171438871
H,0,-3.0230413195,-3.4151326949,-2.46472664
H,0,1.991823969,-1.9134763449,1.9459457396
H,0,1.6463290238,-0.2338599367,1.4774168424
H,0,5.5633500108,-3.3771576607,-0.8093688509
H,0,7.1059821306,-2.2528126784,-2.3428872081
H,0,7.7703253836,-0.2462912474,-3.636323877
H,0,6.4110517887,1.8465805934,-3.4941358365
H,0,4.409519251,1.9342078325,-2.0478480895
H,0,4.9295392508,1.3450862094,1.0303666569
H,0,5.6793004001,3.316600909,2.3202023141
H,0,4.2769922337,5.3846323814,2.3862894492
H,0,2.1160196954,5.4628268481,1.1747299419
H,0,0.4173664827,4.3623599544,-0.2089933244
H,0,0.1230631937,0.2882901221,-2.522609868
H,0,1.6822435602,-0.5158441828,-2.2991027494
H,0,4.1085072993,-5.1218740174,-0.2766603898
H,0,3.5088916664,-5.4183164338,1.3884165611
H,0,5.0876579037,-4.605625297,1.1371886788
H,0,-0.9293960834,4.2772359178,-2.0654952349
H,0,-1.7510007312,3.7322933103,-0.5522152572
H,0,-2.354009273,3.1928846812,-2.1474838378

INT-1
Opt @ B3LYP-D3(BJ)/def2-SVP in gas phase
SCF Done: E(RB3LYP) = -3781.10941621 a.u.
Zero-point correction = 0.882846 Hartree/Particle
Sum of electronic and thermal Free Energies = -3780.309619 a.u.
Sp @ RI-PWPB95-D3(BJ)/def2-TZVPP in 2,2,2-trifluoroethanol
FINAL SINGLE POINT ENERGY = -3781.961528030582 a.u.

Co,0,-1.3383514999,-0.7457127037,0.3018206536
C,0,-4.1921887764,0.8184398831,-2.3667846119
C,0,-4.8195174977,0.7848616085,-1.0934005871

C,0,-6.1338381073,1.2055733281,-0.8909726262
C,0,-6.8302683974,1.6666401491,-2.0114833702
C,0,-6.2316665089,1.7066627877,-3.283246438
C,0,-4.9146975796,1.2869296725,-3.4726733622
C,0,-2.8375333921,0.3184150649,-2.1995846499
C,0,-2.6418047656,-0.0015388723,-0.8815907372
H,0,-6.5875805478,1.1724198888,0.0974198991
H,0,-7.8628241228,2.002164476,-1.8941981483
H,0,-6.809166145,2.0721474842,-4.1349574562
H,0,-4.4557252992,1.3184589695,-4.46331738
H,0,-2.0929220508,0.2151018582,-2.9848566932
N,0,-3.8588830369,0.2782804179,-0.2002228695
C,0,-3.8934492294,0.0809054638,1.1451305079
C,0,-4.8654578332,0.2006781093,3.193867287
C,0,-2.6373768921,-0.5883486868,2.9687850043
C,0,-3.7004879132,-0.2777530164,3.8085171256
H,0,-5.7520040689,0.4482357577,3.7872383556
H,0,-1.6874787763,-0.9682476822,3.3482757039
H,0,-3.6285841224,-0.4080234959,4.8877726237
N,0,-4.9598462024,0.3873927547,1.8800655055
N,0,-2.7354717741,-0.4375580538,1.6423001803
H,0,-1.7803967278,1.7801987554,-0.8178331506
C,0,-0.8143234812,2.146357212,0.7683582461
O,0,-1.5167516781,2.5575934085,-0.2535449224
O,0,-0.5457310033,0.9526548922,0.9596600229
C,0,-0.4418320846,3.2025916658,1.7942648935
C,0,0.8450188492,2.793155902,2.5248980817
H,0,0.7325642217,1.8168847705,3.0160073488
H,0,1.0807015837,3.5418830795,3.2949022913
H,0,1.6964772817,2.7371011037,1.8330629332
C,0,-1.6362391866,3.2127488887,2.7833115961
H,0,-2.5761199629,3.4765942843,2.2747714479
H,0,-1.4488895874,3.9616292252,3.5671113801
H,0,-1.75982555,2.2331529646,3.2693613905
C,0,-0.2892675057,4.5868973731,1.1492375016
H,0,0.5455010178,4.5976482173,0.4366113703
H,0,-0.0760438137,5.3290719613,1.9323283136
H,0,-1.2041394036,4.892774042,0.6238694265
O,0,2.9194790124,-3.4980556037,2.0519914368
O,0,0.0926888911,1.6091582308,-2.5739189313
C,0,-1.4297252133,-2.7932040455,0.0614194171
C,0,-0.4873590813,-2.5205831805,1.1232583768
C,0,0.5541843448,-1.7166622359,0.6359016376
C,0,0.3006165924,-1.4754873526,-0.7778285971
C,0,-0.8843199009,-2.1799838241,-1.1112327215
C,0,-2.6045529868,-3.7530939888,0.0982624301
C,0,-2.0508100399,-5.1259582428,-0.3501879925
C,0,-3.1838186503,-3.8854403244,1.5153802154
C,0,-3.717586576,-3.3104796895,-0.8659798806
C,0,1.6897960497,-1.1479396594,1.4328341658
C,0,3.0131671459,-1.5114505283,0.802279164
C,0,3.6183394801,-2.7597211939,1.1502878629
C,0,4.8183521054,-3.137753177,0.5844695736
C,0,5.453061592,-2.3062119428,-0.3788966072
C,0,6.681593255,-2.6844215691,-0.9858842628
C,0,7.2771380932,-1.8837661412,-1.937368212

C,0,6.6662448164,-0.6684894086,-2.3294307088
C,0,5.4758275133,-0.2717620893,-1.7565664964
C,0,4.8425171593,-1.0685330966,-0.763610574
C,0,3.6062013561,-0.6899918519,-0.1408330018
C,0,2.9339290328,0.5813621236,-0.5454981532
C,0,3.4780559066,1.8393701762,-0.1231667205
C,0,4.5829085361,1.9296354031,0.7667113522
C,0,5.0722219182,3.1567056899,1.1647416312
C,0,4.4844929505,4.3506952529,0.6816246483
C,0,3.4122820732,4.2958595358,-0.1840655508
C,0,2.8756468847,3.0467851792,-0.5992678909
C,0,1.7431806005,2.9721000089,-1.4540267499
C,0,1.200496397,1.7502655168,-1.7933331173
C,0,1.7975930801,0.530649379,-1.3394424921
C,0,1.1977938954,-0.7877937005,-1.7740064507
C,0,3.4521248182,-4.7365916209,2.4757910138
C,0,-0.4009010563,2.7447664666,-3.2648092622
H,0,-0.5811471143,-2.8455291214,2.1571189783
H,0,-1.3342834136,-2.1965562541,-2.1001520509
H,0,-1.6460065367,-5.0760906462,-1.3724287359
H,0,-2.8519644511,-5.8810448113,-0.3358973603
H,0,-1.2455743056,-5.4676736626,0.3183001311
H,0,-2.427110898,-4.2193188713,2.2416279653
H,0,-3.9898570052,-4.6339854638,1.5190045489
H,0,-3.6106982697,-2.9366876542,1.8688066036
H,0,-4.1480205284,-2.3434450571,-0.5713881764
H,0,-4.5263441684,-4.0563341354,-0.8679706653
H,0,-3.3559208672,-3.2155820488,-1.9000283946
H,0,1.6378513662,-1.5372250629,2.4569586543
H,0,1.5834383704,-0.0562758597,1.472837856
H,0,5.2981512229,-4.0788460993,0.8505443054
H,0,7.1469950017,-3.6267615849,-0.6866499975
H,0,8.2212736713,-2.1880034977,-2.3946531911
H,0,7.140719056,-0.0435702464,-3.0890302711
H,0,5.0098323058,0.6651448389,-2.0630114628
H,0,5.0398684862,1.0111292677,1.1364577654
H,0,5.9188805606,3.2092393083,1.8523748013
H,0,4.8843948792,5.317242302,0.9960829674
H,0,2.9569697519,5.2159453656,-0.5588078733
H,0,1.3075334697,3.900432068,-1.8198018736
H,0,0.6318214241,-0.642359201,-2.7017379122
H,0,2.0162967798,-1.4911282051,-1.998767447
H,0,3.5656033781,-5.4378101471,1.6308908385
H,0,2.7392697366,-5.1544466706,3.1981636577
H,0,4.4317903308,-4.6080107038,2.9673935929
H,0,0.3777952929,3.1837315922,-3.9102047753
H,0,-0.7726721057,3.5116389148,-2.5663634473
H,0,-1.2354325963,2.3944594224,-3.8848050413

INT-2
Opt @ B3LYP-D3(BJ)/def2-SVP in gas phase
SCF Done: E(RB3LYP) = -4241.86973506 a.u.
Zero-point correction = 1.037869 Hartree/Particle
Sum of electronic and thermal Free Energies = -4240.925678 a.u.
Sp @ RI-PWPB95-D3(BJ)/def2-TZVPP in 2,2,2-trifluoroethanol

```
FINAL SINGLE POINT ENERGY = -4242.867998829570 a.u.
-----------------------------------------------------------
Co,0,1.3282318136,-0.6439350114,0.145339713
O,0,-2.3909253145,-3.6026512921,-2.2504171367
O,0,-0.9986318195,1.5058543005,3.0469361843
C,0,1.3704313245,-2.742114309,0.4766501674
C,0,0.6678181235,-2.4237391087,-0.7324817105
C,0,-0.4725485601,-1.6335966145,-0.4252747437
C,0,-0.5038967156,-1.4418948792,1.0091601458
C,0,0.6359864515,-2.1064934066,1.5240434753
C,0,2.4797285296,-3.7594661585,0.6900011021
C,0,1.8370017118,-4.9275505543,1.4759396651
C,0,3.0132587968,-4.2977054445,-0.6449001814
C,0,3.6389446898,-3.1722977422,1.514406788
C,0,-1.5032968683,-1.1718847797,-1.4173315941
C,0,-2.8751693558,-1.6639157513,-1.0163267909
C,0,-3.2987584636,-2.949315935,-1.4794880205
C,0,-4.5418775001,-3.4402830961,-1.1387484569
C,0,-5.4051339465,-2.6893370085,-0.2944824949
C,0,-6.6851950422,-3.18281483,0.077882181
C,0,-7.505873959,-2.4597123619,0.9171797684
C,0,-7.0796934401,-1.2099954415,1.4273936666
C,0,-5.8443654106,-0.7023765991,1.0827602366
C,0,-4.9793148942,-1.417467863,0.2094803737
C,0,-3.6889135002,-0.9216544184,-0.1774948097
C,0,-3.2133012079,0.3810659727,0.3838988025
C,0,-3.7499302437,1.6161852417,-0.1086787305
C,0,-4.657054484,1.6666897642,-1.2021434582
C,0,-5.1455622803,2.8726751955,-1.6595046611
C,0,-4.7548971229,4.083196277,-1.0375916239
C,0,-3.8787127427,4.0665318012,0.0269429159
C,0,-3.3487659156,2.8413225558,0.5146609466
C,0,-2.4326585356,2.8082375646,1.601189713
C,0,-1.9008539737,1.6109242356,2.0312909798
C,0,-2.2746314371,0.3769123258,1.4067297758
C,0,-1.6258161285,-0.9065028033,1.8674503191
C,0,-2.7318089592,-4.8681055882,-2.7812168986
C,0,-0.7705194388,2.6325502089,3.8872352459
H,0,0.9451326296,-2.7413144547,-1.733848779
H,0,0.9103769141,-2.1231493015,2.5777209612
H,0,1.5006731352,-4.6095208464,2.4747289168
H,0,2.5706637386,-5.7373224012,1.6086705241
H,0,0.9685850544,-5.3384560712,0.9382264593
H,0,2.2165522368,-4.7836358914,-1.2295338416
H,0,3.7900793078,-5.0533133681,-0.4555107359
H,0,3.4583513759,-3.4982960896,-1.2508814306
H,0,4.2130975477,-2.4430590162,0.9299277163
H,0,4.3273303286,-3.9765879835,1.813603038
H,0,3.2844751863,-2.6817179389,2.4342083956
H,0,-1.2406560487,-1.5635845609,-2.4070660982
H,0,-1.5257427307,-0.0766922551,-1.4878793603
H,0,-4.8843833123,-4.4105370638,-1.4963848486
H,0,-7.008491044,-4.1510369714,-0.3120863253
H,0,-8.4870412735,-2.8519202235,1.1937451816
H,0,-7.7332107973,-0.6463492713,2.0965761177
H,0,-5.5225731521,0.2609084185,1.4792955502
```

H,0,-4.9628720145,0.7342000486,-1.6781507754
H,0,-5.8395784116,2.8960150332,-2.5022168858
H,0,-5.1537828113,5.0323193731,-1.4024269081
H,0,-3.5818369156,4.9995213686,0.5125360934
H,0,-2.1521865204,3.7478961196,2.0759246542
H,0,-1.2513171202,-0.782419588,2.8904411941
H,0,-2.3900205909,-1.7003532788,1.9002540677
H,0,-2.9314947536,-5.6007974103,-1.9802782034
H,0,-1.8691402115,-5.2035014718,-3.3708025591
H,0,-3.6166120278,-4.8048894893,-3.4375217237
H,0,-1.7234522224,3.0281719462,4.2737531296
H,0,-0.208687006,3.4243835024,3.3691642202
H,0,-0.1654827481,2.269238726,4.7280720334
C,0,4.9253514852,-0.8129770477,-2.0463787191
C,0,5.2506309462,-0.2320566036,-0.791027045
C,0,6.5460825701,0.158464516,-0.4523694727
C,0,7.5398232516,-0.0453093763,-1.4134335452
C,0,7.2441521956,-0.6191133506,-2.6630206025
C,0,5.9445083332,-1.0065263575,-2.9903486111
C,0,3.4987804152,-1.0872443268,-2.0450627501
C,0,2.9857344172,-0.6981160559,-0.8393932405
H,0,6.7605778517,0.6013895604,0.5179425906
H,0,8.5669830295,0.2480738823,-1.1868803859
H,0,8.047668238,-0.7634118564,-3.38849526
H,0,5.72335139,-1.4525164858,-3.9624930147
H,0,2.9378392742,-1.5162946637,-2.8728735903
N,0,4.0449330196,-0.1687657826,-0.0698661032
C,0,3.7663551164,0.2881758972,1.1803765758
C,0,4.3162449422,1.2492443607,3.158949083
C,0,2.064893722,0.6406139373,2.7063270433
C,0,2.9861010507,1.2006728841,3.5851819315
H,0,5.0976568284,1.6750019741,3.7957546881
H,0,0.9981280323,0.5932593261,2.9306961297
H,0,2.6726157793,1.6003362779,4.5481569509
N,0,4.7037404554,0.7965975443,1.9682606885
N,0,2.4519553112,0.171204501,1.5148768475
C,0,0.2970371848,4.9858190469,-3.1889305974
C,0,-0.4915072479,5.3090066826,-2.0833715091
C,0,-0.3798433182,4.5908846414,-0.8769662751
C,0,0.5426661792,3.5624530114,-0.8242684795
C,0,1.339186413,3.2363496901,-1.9397908641
C,0,1.2289876738,3.930329622,-3.1315981484
C,0,0.940998622,2.5405036975,0.2433975333
C,0,0.3670284943,1.2232684523,-0.2735314764
C,0,1.1581648099,0.8882127138,-1.364569486
C,0,2.1761075614,2.0428176348,-1.4720005542
O,0,2.3436997021,2.3763698333,-0.07862261
H,0,0.1936609432,5.5611571786,-4.1113873789
H,0,-1.2063335063,6.1315846928,-2.1533804733
H,0,-1.0014059514,4.8431571692,-0.0181637604
H,0,1.8439011649,3.6831027504,-3.9999070575
H,0,0.814088732,2.8156388889,1.2900321373
H,0,-0.6904757689,1.0230952707,-0.1631538338
H,0,0.8350534975,0.3444495921,-2.2508222954
H,0,3.1329286962,1.8416284043,-1.9613224863
C,0,3.7687439423,5.5454548985,3.3389139834

C,0,2.8250674623,5.9735574089,4.4673661123
H,0,1.9143044541,6.4430630616,4.0680487361
H,0,2.5158391029,5.1122130854,5.0777621652
H,0,3.3297047759,6.698990133,5.123006863
C,0,5.0321421906,4.881245948,3.9228360768
H,0,4.768682447,4.0213064085,4.5596567936
H,0,5.7062404218,4.5353993836,3.12650653
H,0,5.5761604408,5.6038650256,4.5500099033
C,0,4.1693651317,6.7628440143,2.4816463992
H,0,4.8555509236,6.4722623921,1.6742635129
H,0,3.2845816826,7.2377380345,2.0292646028
H,0,4.6698899404,7.5128555902,3.1131118268
C,0,3.059477057,4.5312253199,2.4421715389
O,0,1.9328683343,4.1113678267,2.6416911206
O,0,3.8058277031,4.1359337584,1.414748314

TS-2
Opt @ B3LYP-D3(BJ)/def2-SVP in gas phase
SCF Done: E(RB3LYP) = -4241.86494139 a.u.
Imaginary frequency = -266.0531 cm^{-1}
Zero-point correction = 1.037508 Hartree/Particle
Sum of electronic and thermal Free Energies = -4240.920141 a.u.
Sp @ RI-PWPB95-D3(BJ)/def2-TZVPP in 2,2,2-trifluoroethanol
FINAL SINGLE POINT ENERGY = -4242.859332369071 a.u.

Co,0,0.1918095443,-1.1387925523,0.2599247758
O,0,-4.0675884177,-3.3056939368,-2.1364833104
O,0,-1.5371480363,1.6102295417,2.9165218251
C,0,-0.2185647137,-3.2028821057,0.6653331234
C,0,-0.8017002865,-2.7936154066,-0.5722588253
C,0,-1.7405240737,-1.7450208691,-0.3491865926
C,0,-1.7569757796,-1.4762736585,1.0676381798
C,0,-0.8053985666,-2.3573945052,1.6544551796
C,0,0.6721653897,-4.4032803206,0.9409932147
C,0,-0.1718162472,-5.3978167925,1.7703280505
C,0,1.1024051309,-5.0817892166,-0.3676177665
C,0,1.9225241513,-4.0045853714,1.7452310967
C,0,-2.6160907079,-1.1241835541,-1.4033113086
C,0,-4.0741945258,-1.2367789898,-1.0233663822
C,0,-4.7967801709,-2.4023037112,-1.4305279303
C,0,-6.1289486587,-2.5490166815,-1.1048493005
C,0,-6.7929912129,-1.5592193849,-0.3294658563
C,0,-8.16169763,-1.6986827603,0.0282479106
C,0,-8.7914300446,-0.7479850777,0.8031144885
C,0,-8.0764705101,0.3845661762,1.2608601256
C,0,-6.7481638249,0.5504838511,0.927963111
C,0,-6.0730474001,-0.4047012746,0.1191937894
C,0,-4.6930090128,-0.266452744,-0.2537337518
C,0,-3.9193412478,0.9103285148,0.2514927374
C,0,-4.1479523271,2.2132693185,-0.302554864
C,0,-5.0016638716,2.4239075233,-1.4195620359
C,0,-5.2037118762,3.6907115335,-1.9262960989
C,0,-4.568571212,4.8071023801,-1.330573084
C,0,-3.7360035535,4.6349612255,-0.2449260976
C,0,-3.4945945071,3.3405085211,0.2905665761

```
C,0,-2.6209920341,3.143613686,1.3945474272
C,0,-2.3754784819,1.8742624795,1.8743727387
C,0,-3.0228231197,0.7354660552,1.2963798541
C,0,-2.7291844015,-0.6354642332,1.8541976245
C,0,-4.7040923386,-4.4769403776,-2.6063962162
C,0,-1.1230290878,2.6851086664,3.7537814417
H,0,-0.5715247833,-3.2075822945,-1.5502693722
H,0,-0.564780167,-2.3729683019,2.7167481804
H,0,-0.4545704127,-4.9726424138,2.7458093557
H,0,0.4027019983,-6.3183418037,1.9567211014
H,0,-1.0958000151,-5.672858909,1.2387763286
H,0,0.2326898406,-5.4387652528,-0.9407825579
H,0,1.7328696042,-5.955662897,-0.1456717016
H,0,1.6839685318,-4.3987212957,-1.0013357228
H,0,2.6161483342,-3.4151239579,1.1324586688
H,0,2.4583952916,-4.9065535665,2.0766806368
H,0,1.6671215747,-3.4220518883,2.6437496266
H,0,-2.4387655301,-1.638716371,-2.3552210644
H,0,-2.3713250438,-0.0638501359,-1.5500717091
H,0,-6.6959284876,-3.4236512576,-1.4213571015
H,0,-8.7083713069,-2.578284433,-0.3204358199
H,0,-9.843813407,-0.8689681024,1.0693968834
H,0,-8.580133502,1.1297388743,1.8802905393
H,0,-6.2040733231,1.4252857394,1.2845457684
H,0,-5.4980848323,1.5653187181,-1.8735373501
H,0,-5.8601433235,3.8360800336,-2.7867505815
H,0,-4.743987516,5.8077780378,-1.7318542478
H,0,-3.2505124948,5.4967081792,0.2203878859
H,0,-2.1438081916,4.0135379933,1.8448416994
H,0,-2.3585401488,-0.5417979284,2.8818355597
H,0,-3.6687173294,-1.2101409985,1.902605524
H,0,-5.0938012831,-5.0866237023,-1.7728549368
H,0,-3.9426541795,-5.0517606625,-3.1488449217
H,0,-5.5342661436,-4.2375015307,-3.2929371356
H,0,-1.994680734,3.2623310496,4.1021640646
H,0,-0.4013584142,3.3433136778,3.2487490036
H,0,-0.6254633687,2.2262025934,4.6181839072
C,0,3.5923155176,-2.3002849065,-1.9439122577
C,0,4.0612263578,-1.7477251323,-0.7193746823
C,0,5.4021221544,-1.8033931384,-0.3355888028
C,0,6.2865620098,-2.4290689916,-1.2165597337
C,0,5.8475147498,-2.9797907931,-2.436482752
C,0,4.5075890675,-2.9221847394,-2.8104825982
C,0,2.1703355988,-2.0741700706,-2.0053275856
C,0,1.7778692531,-1.4173835326,-0.8602595578
H,0,5.7314545543,-1.3711089412,0.6066240246
H,0,7.3445811637,-2.4885454918,-0.9528390942
H,0,6.5723755919,-3.4590380535,-3.0977640921
H,0,4.1709059902,-3.3528767898,-3.7558224259
H,0,1.5224745535,-2.3318427932,-2.8408576008
N,0,2.9369093609,-1.2134717227,-0.0714297561
C,0,2.7945107057,-0.7250369857,1.1978389486
C,0,3.6008145596,0.0780388448,3.1569164381
C,0,1.2594680773,0.0124807531,2.7581312046
C,0,2.3074323436,0.3523505076,3.6076794932
H,0,4.4745500495,0.3034287889,3.7755890326
```

```
H,0,0.2148605568,0.2148862573,3.000408505
H,0,2.1192251555,0.8242300684,4.5707336667
N,0,3.8430829073,-0.4519539579,1.9579937019
N,0,1.5002120435,-0.553602065,1.569919497
C,0,0.5832684357,4.3251303986,-3.4221751719
C,0,-0.2130936039,4.8247079528,-2.3890671037
C,0,-0.2888736701,4.1683539175,-1.1467648549
C,0,0.4593286597,3.0164468583,-0.9771502416
C,0,1.265007216,2.5163661717,-2.0186390014
C,0,1.3356887638,3.1482697126,-3.2490606948
C,0,0.612715145,2.0001736453,0.1529605344
C,0,-0.137491592,0.76255966,-0.350772606
C,0,0.7079383841,0.2378921685,-1.3814241266
C,0,1.8808676413,1.2461546855,-1.4474942298
O,0,1.9935003055,1.598449799,-0.0558289223
H,0,0.6270801596,4.8565433023,-4.3751910997
H,0,-0.7878337898,5.739913206,-2.5460684584
H,0,-0.9191183719,4.5592498333,-0.3500491935
H,0,1.9587655911,2.7612499601,-4.0585953364
H,0,0.4683656397,2.3401749925,1.1782732564
H,0,-1.2073280699,0.8761122912,-0.4899255656
H,0,0.2989871258,-0.1506539022,-2.3140304263
H,0,2.8318444491,0.8916596567,-1.855689632
C,0,3.8909470128,4.3955316802,3.4251950737
C,0,4.9543883582,3.4745285447,4.0568787923
H,0,5.6038100022,4.0597260468,4.7256800244
H,0,4.4836091279,2.6822380113,4.6612387034
H,0,5.5825109932,3.0034631853,3.2876056811
C,0,4.5825645898,5.5069976125,2.6106433135
H,0,5.2299534695,5.0838865457,1.8301886462
H,0,3.8420683059,6.1633646379,2.1271340315
H,0,5.1995909524,6.1276887577,3.2783699233
C,0,3.0029648335,5.0049040161,4.5148462559
H,0,2.233193454,5.659508312,4.0809625234
H,0,2.4883062509,4.2238179431,5.0938729948
H,0,3.6152354095,5.6020766165,5.2071638087
C,0,3.0300363254,3.5611334304,2.4772157508
O,0,1.8313370178,3.3893667262,2.6121772947
O,0,3.7258677346,3.0193300031,1.481893338
H,0,3.121854397,2.5236780243,0.8737690252
-----------------------------------------------------------------
```

INT-3

```
Opt @ B3LYP-D3(BJ)/def2-SVP in gas phase
SCF Done: E(RB3LYP) = -4241.86948328 a.u.
Zero-point correction = 1.037996 Hartree/Particle
Sum of electronic and thermal Free Energies = -4240.927519 a.u.
Sp @ RI-PWPB95-D3(BJ)/def2-TZVPP in 2,2,2-trifluoroethanol
FINAL SINGLE POINT ENERGY = -4242.862695341757 a.u.
-----------------------------------------------------------------
Co,0,-1.0480047905,-0.7827314548,0.7528304858
O,0,3.3010333254,-3.5367876066,0.5041831946
O,0,-0.3925386855,2.4130709692,-2.197611205
C,0,-1.1396796488,-2.7945601714,-0.0147320691
C,0,0.0311419059,-2.6024213354,0.7383233473
C,0,0.7857173659,-1.4957287661,0.2026703038
```

C,0,0.1059997579,-1.0535855498,-0.9827182693
C,0,-1.11301748,-1.7893660421,-1.0534361036
C,0,-2.1684055761,-3.8930042284,0.1935146818
C,0,-3.3803928058,-3.7026541969,-0.728310288
C,0,-1.4751856936,-5.2311800153,-0.145340868
C,0,-2.6467585945,-3.9175809112,1.6559786864
C,0,2.1516010853,-1.0739201667,0.6633237503
C,0,3.142976022,-1.3887031392,-0.4294604296
C,0,3.706602193,-2.7018487707,-0.4884479868
C,0,4.5838998074,-3.0434401405,-1.4963128137
C,0,4.9107844146,-2.1041269992,-2.5136924355
C,0,5.7983783537,-2.4408048916,-3.5715949096
C,0,6.0897445159,-1.533534918,-4.5684088328
C,0,5.5001745083,-0.2470687506,-4.5539888175
C,0,4.6378606229,0.1116481161,-3.5384420864
C,0,4.3263679823,-0.7962632287,-2.4897373646
C,0,3.4366652435,-0.4622870273,-1.4130866854
C,0,2.8035428397,0.8907821756,-1.371921031
C,0,3.6028848276,2.0307675151,-1.0240568995
C,0,4.9608031649,1.9102701867,-0.6198687093
C,0,5.7118584716,3.0272226216,-0.3137709696
C,0,5.1373034996,4.3188424272,-0.4014034148
C,0,3.818572438,4.4663704736,-0.7781553975
C,0,3.0175266911,3.3339397848,-1.0896809214
C,0,1.657896479,3.4695219359,-1.4772618207
C,0,0.9035610794,2.3549193438,-1.7839686976
C,0,1.4649656413,1.0379623837,-1.7067954846
C,0,0.6012884601,-0.1472300251,-2.0795574042
C,0,3.8252024628,-4.847103694,0.5509465294
C,0,-0.9637529502,3.6751025283,-2.4749444241
H,0,0.3284374914,-3.1520314332,1.6269653009
H,0,-1.8990913104,-1.621794004,-1.7890349168
H,0,-3.9172243175,-2.7737362982,-0.4996230131
H,0,-4.0859338585,-4.5362765956,-0.5942743464
H,0,-3.0889234968,-3.6783972515,-1.7884632611
H,0,-1.1051191769,-5.2309890283,-1.1820671356
H,0,-2.1835287221,-6.066999281,-0.0349512767
H,0,-0.6184750406,-5.4176128218,0.5204359892
H,0,-1.8076002275,-4.0288815097,2.3589002825
H,0,-3.3327016477,-4.7626836396,1.8184773083
H,0,-3.1895762393,-2.9926780006,1.9052973624
H,0,2.4088795508,-1.617807888,1.5768304195
H,0,2.1803938245,-0.004129953,0.8942614178
H,0,5.0323693675,-4.0351036187,-1.545434707
H,0,6.2458108815,-3.4377670641,-3.5840202349
H,0,6.7736090255,-1.8077719614,-5.3747679952
H,0,5.7293457881,0.4635076793,-5.3510569041
H,0,4.1862515903,1.1041724132,-3.5339637705
H,0,5.4063202812,0.9171011039,-0.5581976967
H,0,6.7555128836,2.9168683718,-0.0116329126
H,0,5.7434944883,5.1985037985,-0.1728282769
H,0,3.3710533408,5.4611910375,-0.8470871222
H,0,1.2359792785,4.4715668128,-1.5416443943
H,0,-0.2604568475,0.1973957771,-2.6635868408
H,0,1.1885820506,-0.8038696889,-2.745255504
H,0,3.562935835,-5.4205429997,-0.3552408437

```
H,0,3.3774924176,-5.3319494745,1.4279446922
H,0,4.9231928046,-4.8394894934,0.663010057
H,0,-0.3448420151,4.2493961032,-3.1841701693
H,0,-1.1054266567,4.2679089537,-1.5543647091
H,0,-1.9418507605,3.4810204765,-2.9311078193
C,0,0.1195447931,-1.4564743628,3.6917600835
C,0,1.1018429227,-0.4395159764,3.6846705047
C,0,2.3738159689,-0.6514209719,4.2265706008
C,0,2.6355455365,-1.9031042193,4.7833301248
C,0,1.6639948907,-2.9210883342,4.8067958986
C,0,0.4000828957,-2.7048305756,4.2644859291
C,0,-1.0579229822,-0.9193193591,3.0416548785
C,0,-0.7831119699,0.4147583112,2.7292100885
H,0,3.1349356163,0.1231704622,4.194694484
H,0,3.6213553855,-2.0939470368,5.2125477845
H,0,1.9042642198,-3.8850220879,5.2596401264
H,0,-0.3619112408,-3.4868663726,4.2884489441
H,0,-2.0462235895,-1.3676494337,3.0857486137
N,0,0.5316439763,0.708286619,3.0921051971
C,0,1.2212054689,1.9103907211,2.8097075723
C,0,3.1924030078,2.9359053537,2.3363462058
C,0,1.1736958994,4.1609660631,2.5200587445
C,0,2.5486633854,4.1733291629,2.2934135321
H,0,4.2640562563,2.8455132846,2.1387702694
H,0,0.5836520573,5.0831318535,2.4937821634
H,0,3.0926407552,5.0917941288,2.0783738598
N,0,2.5304215014,1.8053848774,2.5883105907
N,0,0.5088553192,3.0345182501,2.7887115581
C,0,-5.6148585417,3.6482968672,0.993726186
C,0,-5.2166458533,3.7601887987,-0.3433045959
C,0,-4.1985544363,2.9465064933,-0.8636980549
C,0,-3.5967427795,2.0316134868,-0.0096050294
C,0,-4.0065133531,1.9134103621,1.3271798781
C,0,-5.0110346557,2.7147415238,1.8510966315
C,0,-2.5283240297,0.9953522659,-0.2166808871
C,0,-1.245182902,1.1999988351,0.5611367262
C,0,-1.6987184231,1.3696754861,2.0229657593
C,0,-3.1491088674,0.8268539789,1.9141381867
O,0,-3.0103304634,-0.0901831691,0.750491093
H,0,-6.4174632334,4.285120247,1.3714191021
H,0,-5.718068318,4.479193249,-0.9946837895
H,0,-3.9100304617,3.0156228986,-1.913408343
H,0,-5.3352875395,2.6205797839,2.8897131175
H,0,-2.4536648725,0.5805297944,-1.2217670523
H,0,-0.4573329144,1.8505793327,0.1881757035
H,0,-1.6631474508,2.3812315807,2.4390600018
H,0,-3.5432399948,0.2789478278,2.7754364419
H,0,-4.536910936,-0.6415439648,-0.0809803387
C,0,-5.1175318135,-0.6576975329,-1.8946435747
O,0,-5.3783355226,-0.7184786848,-0.5831682871
O,0,-3.982557319,-0.6137209512,-2.3302568332
C,0,-6.3796815796,-0.6357714628,-2.7496453457
C,0,-7.2272007184,-1.8801362198,-2.4185614217
H,0,-7.5198391329,-1.8938297944,-1.3595269847
H,0,-8.1408131415,-1.8796059011,-3.0324882791
H,0,-6.6736464151,-2.8069567806,-2.637506567
```

C,0,-7.1652388982,0.6453607524,-2.3970933648
H,0,-6.5661011366,1.5468738996,-2.6008642045
H,0,-8.0791383986,0.6991043027,-3.0083302626
H,0,-7.4525925146,0.6577172727,-1.3364263355
C,0,-5.9869010366,-0.6318769424,-4.2304227452
H,0,-5.4087788549,-1.5302755788,-4.4916296097
H,0,-6.8910447416,-0.606890292,-4.8570322979
H,0,-5.3685430109,0.2435445439,-4.4762478374

TS-3-*cis*-*SR* [named as TS-*cis*-*SR* in the main text]
Opt @ B3LYP-D3(BJ)/def2-SVP in gas phase
SCF Done: E(RB3LYP) = -4241.85790298 a.u.
Imaginary frequency = -430.9518 cm^{-1}
Zero-point correction = 1.035808 Hartree/Particle
Sum of electronic and thermal Free Energies = -4240.915924 a.u.
Sp @ RI-PWPB95-D3(BJ)/def2-TZVPP in 2,2,2-trifluoroethanol
FINAL SINGLE POINT ENERGY = -4242.845011841107 a.u.

Co,0,1.838520132,-0.6425065132,-1.652928579
O,0,-0.2125920142,4.1043568632,-1.8073217538
O,0,-1.5388010921,-3.1597000559,-0.4475308636
C,0,1.6793516145,0.3599731029,-3.5551000524
C,0,1.6093153004,1.2832661681,-2.4946600146
C,0,0.5257707626,0.9392817786,-1.6080891888
C,0,-0.1449858114,-0.1870155857,-2.1842184551
C,0,0.6220877714,-0.5925208479,-3.3213224521
C,0,2.650279619,0.4167471704,-4.7232017609
C,0,2.4159318181,-0.7517533359,-5.6903267584
C,0,2.3988036084,1.7475010301,-5.4646345482
C,0,4.1043845771,0.3747483664,-4.2183202373
C,0,0.0598546169,1.7656681168,-0.4423689681
C,0,-1.3096148589,2.3133904602,-0.7592217032
C,0,-1.4064570538,3.5343510344,-1.4979653095
C,0,-2.6384636395,4.0464138795,-1.8475197722
C,0,-3.8301005368,3.34609998,-1.5104541801
C,0,-5.1090376246,3.8465111397,-1.8768918453
C,0,-6.2580874581,3.1486313374,-1.5698495767
C,0,-6.1780437226,1.9127034539,-0.8851718318
C,0,-4.9517374337,1.4023056532,-0.5118810212
C,0,-3.7494580169,2.1030661782,-0.8027099812
C,0,-2.4532700334,1.609428465,-0.4299765129
C,0,-2.3410079805,0.3091445824,0.297970419
C,0,-2.7343325672,0.2394737485,1.6762497092
C,0,-3.1384891908,1.3894235139,2.4086830364
C,0,-3.5371690074,1.2872604087,3.7261113233
C,0,-3.5521936033,0.0263251655,4.3714785173
C,0,-3.1518427933,-1.1047544208,3.6912385531
C,0,-2.7262511497,-1.0287866744,2.3368052416
C,0,-2.3082641508,-2.1835570209,1.6239064181
C,0,-1.9116406195,-2.0864427044,0.305220164
C,0,-1.8984065382,-0.8227921256,-0.3726924542
C,0,-1.4867507564,-0.7740265593,-1.828622326
C,0,-0.2019251624,5.3276032788,-2.5127494361
C,0,-1.7013084986,-4.4579786415,0.0878454617
H,0,2.2804745534,2.1197511447,-2.3205302899

H,0,0.4229273399,-1.4800906691,-3.920273048
H,0,2.6304143153,-1.7153954835,-5.2138142077
H,0,3.0829196442,-0.6564172198,-6.5601807334
H,0,1.3806016171,-0.7687736955,-6.0625258058
H,0,1.3586265548,1.8107738986,-5.8196299003
H,0,3.0638865053,1.8275608297,-6.3384645971
H,0,2.588329791,2.6140103893,-4.812562397
H,0,4.3149657514,1.2040967574,-3.5256194661
H,0,4.8033291122,0.4614530282,-5.0641047175
H,0,4.3094924735,-0.5749451372,-3.7020520707
H,0,0.7660206721,2.5845743799,-0.2771704333
H,0,0.0207245512,1.1724251232,0.4775593368
H,0,-2.7276122678,4.9828062343,-2.3972028059
H,0,-5.1666997582,4.7972992447,-2.4125590229
H,0,-7.2334182646,3.5464997918,-1.8588087673
H,0,-7.0916753817,1.3614753541,-0.6524026301
H,0,-4.8977008202,0.4488670092,0.0145484968
H,0,-3.1337521142,2.3596628709,1.9115612262
H,0,-3.8509269029,2.1796868479,4.2717632694
H,0,-3.8846978918,-0.0474284344,5.4094743106
H,0,-3.1610042695,-2.0797097277,4.1851759267
H,0,-2.3288777788,-3.1436339537,2.1376259605
H,0,-1.5595008051,-1.7774452686,-2.2652724889
H,0,-2.2183468636,-0.1477648619,-2.3690485627
H,0,-0.6756455553,5.2267240337,-3.5047874184
H,0,0.8521149497,5.6049012386,-2.6418974214
H,0,-0.7190425701,6.1235482094,-1.9494847744
H,0,-2.7373561883,-4.6254960475,0.4254669047
H,0,-1.0115396673,-4.6391637912,0.9305449055
H,0,-1.4717526331,-5.1605803561,-0.7223368263
C,0,3.6872594166,1.5610837725,-0.0971767131
C,0,2.9273171353,1.559120928,1.0955348354
C,0,2.6802780734,2.738893077,1.8049835204
C,0,3.2166378841,3.9203366569,1.2937131885
C,0,3.9806308154,3.9367345933,0.1113679181
C,0,4.2238414973,2.7592700837,-0.590100069
C,0,3.7322106199,0.1978258974,-0.5787931819
C,0,3.063656108,-0.5937357638,0.35726861
H,0,2.0743083443,2.7375241754,2.7068415538
H,0,3.0371585933,4.8557968506,1.8277371244
H,0,4.3892797769,4.881444399,-0.252620739
H,0,4.8240291064,2.7630121273,-1.5026930957
H,0,4.3901759644,-0.1685023311,-1.3603799704
N,0,2.5524355102,0.2252211498,1.3645470169
C,0,1.708042495,-0.187599422,2.42033582
C,0,0.0083754812,0.3208373228,3.8391831202
C,0,1.0873261925,-1.7872142358,3.9054715696
C,0,0.1154658813,-0.938294857,4.43176052
H,0,-0.7480727187,1.0403260199,4.1644670715
H,0,1.2340510394,-2.7985674846,4.2991301611
H,0,-0.538073204,-1.2411957135,5.2486661622
N,0,0.7985566241,0.6947718339,2.8316201689
N,0,1.8916121608,-1.4126970418,2.907318864
C,0,3.7493459845,-6.5593195651,-0.8286377436
C,0,2.4098004992,-6.7768428557,-1.1671068687
C,0,1.5401551019,-5.697143775,-1.3689701854

C,0,2.036645617,-4.4063301458,-1.1919483334
C,0,3.389716425,-4.1909209167,-0.8729920577
C,0,4.2564907281,-5.2560988372,-0.6905533727
C,0,1.3105441828,-3.137436715,-1.3556730185
C,0,1.4032705237,-2.0984150297,-0.3355281425
C,0,2.8132109321,-2.0722928928,0.2606828677
C,0,3.6237581794,-2.7032134342,-0.9036615416
O,0,2.9148953318,-2.2648905891,-2.0906714023
H,0,4.4173186856,-7.4132919812,-0.697850381
H,0,2.0443039618,-7.7974276902,-1.2975171332
H,0,0.503790337,-5.8614523604,-1.667164367
H,0,5.3134446185,-5.091985727,-0.4698384991
H,0,0.4830336119,-3.1269495959,-2.0645520457
H,0,0.5419281799,-1.9158203838,0.3082440574
H,0,2.962873936,-2.5924414436,1.2133996195
H,0,4.6791502272,-2.4032791455,-0.9383636923
H,0,3.0402441579,-3.2197781858,-3.4837978139
C,0,1.9131069546,-4.2590559533,-4.6382320236
O,0,3.1204086852,-3.8870530218,-4.2164895299
O,0,0.8848431137,-3.7620926642,-4.2119971629
C,0,1.9639180415,-5.3511965418,-5.7033359653
C,0,2.7339887491,-4.7987461916,-6.9204977077
H,0,3.762388287,-4.5228207564,-6.6483387848
H,0,2.7756162047,-5.5628166718,-7.7118651609
H,0,2.2342742286,-3.9088824034,-7.3351303683
C,0,2.7076964672,-6.5694274866,-5.1204111402
H,0,2.188158106,-6.9642039159,-4.2333394337
H,0,2.7537875947,-7.3716626924,-5.8728521325
H,0,3.7330837917,-6.3061116237,-4.8262641805
C,0,0.5364481742,-5.7362729431,-6.1040992547
H,0,-0.0121094898,-4.8717018445,-6.504731362
H,0,0.5622520819,-6.5204488957,-6.8756807665
H,0,-0.0300607436,-6.1172999617,-5.2415602818

TS-3-*cis*-*RS* [named as TS-*cis*-*RS* in the main text]
Opt @ B3LYP-D3(BJ)/def2-SVP in gas phase
SCF Done: E(RB3LYP) = -4241.84580311 a.u.
Imaginary frequency = -425.2437 cm^{-1}
Zero-point correction = 1.035316 Hartree/Particle
Sum of electronic and thermal Free Energies = -4240.905124 a.u.
Sp @ RI-PWPB95-D3(BJ)/def2-TZVPP in 2,2,2-trifluoroethanol
FINAL SINGLE POINT ENERGY = -4242.830659194292 a.u.

Co,0,-1.1021129848,-1.1740440474,-0.0007563715
O,0,3.9036828927,-3.0287053029,-0.6050173093
O,0,-0.8794723864,2.5647811948,-1.3437456357
C,0,-0.6053958727,-2.9114662038,-1.0843969046
C,0,0.3486624004,-2.6478452557,-0.0645367967
C,0,1.0405986899,-1.4225238163,-0.3692028254
C,0,0.401692491,-0.8255927862,-1.483495955
C,0,-0.6377455444,-1.7353828965,-1.9061357399
C,0,-1.2444687035,-4.2614775958,-1.3540716294
C,0,-0.2626995428,-4.9517061355,-2.3354079073
C,0,-1.3424014373,-5.1048216718,-0.072613585
C,0,-2.6258029265,-4.174314706,-2.0223707713

C,0,2.3021929672,-0.9883839784,0.3098402758
C,0,3.3679891233,-0.7465141804,-0.731171921
C,0,4.1796329883,-1.830728033,-1.1846050966
C,0,5.1493935057,-1.6209077168,-2.1443761736
C,0,5.3303817575,-0.3302441908,-2.7163346233
C,0,6.3179778812,-0.0902796819,-3.7100692017
C,0,6.4700012958,1.1610666977,-4.2699958609
C,0,5.6371568996,2.2316434861,-3.8641596908
C,0,4.6693823585,2.032007845,-2.9014600397
C,0,4.4944996726,0.7564816831,-2.2998558523
C,0,3.5065790295,0.5088363833,-1.2936524401
C,0,2.5803130659,1.5880695295,-0.8390909715
C,0,3.0445380899,2.5685574264,0.098247358
C,0,4.3654439293,2.5509128817,0.6227010942
C,0,4.7628200823,3.4728951423,1.5695495671
C,0,3.8568021398,4.4593811277,2.0287691375
C,0,2.569418714,4.5046060114,1.5346397999
C,0,2.1280909924,3.5623467199,0.5660086238
C,0,0.7965336659,3.5704516445,0.0702269504
C,0,0.3717385696,2.6064759472,-0.8238326546
C,0,1.2609218788,1.5710937942,-1.2656003547
C,0,0.7421581034,0.4734509624,-2.1747472676
C,0,4.6758220396,-4.153294443,-0.9699047633
C,0,-1.7865994966,3.6029135976,-1.0389625606
H,0,0.6166540862,-3.3063003485,0.7588491254
H,0,-1.3226553733,-1.551374929,-2.7328064417
H,0,-0.2091529946,-4.4044261046,-3.2884896463
H,0,-0.600716619,-5.9783890225,-2.5460141056
H,0,0.7521236113,-4.9995836822,-1.9122555438
H,0,-0.3535636129,-5.301912577,0.365922186
H,0,-1.8033264289,-6.0776984602,-0.2997671899
H,0,-1.9577342364,-4.6118986953,0.6942159311
H,0,-3.4075247222,-3.900271877,-1.3008479309
H,0,-2.8995188552,-5.1599378026,-2.4275540452
H,0,-2.6488142107,-3.4558286094,-2.8546891727
H,0,2.613789175,-1.7641218874,1.0186176784
H,0,2.1525632599,-0.0685514009,0.877314801
H,0,5.7873330411,-2.4329711617,-2.4915683777
H,0,6.9577047813,-0.9180203335,-4.0262425875
H,0,7.2345488187,1.3289443021,-5.0318988917
H,0,5.7633061399,3.2179440106,-4.3158604487
H,0,4.0278926294,2.8569658046,-2.5877955073
H,0,5.0630252052,1.7898791253,0.2715484549
H,0,5.780399977,3.4451041342,1.9650545724
H,0,4.1810214479,5.1871525917,2.7760407726
H,0,1.8683231522,5.2651285181,1.8874144782
H,0,0.1248651259,4.3573831592,0.4106276341
H,0,-0.1508208945,0.8275326395,-2.7046283407
H,0,1.50579962,0.2494711766,-2.9378534649
H,0,4.5733146652,-4.3831302315,-2.0449432098
H,0,4.2962651935,-4.9991039195,-0.3822034552
H,0,5.7445136826,-4.0029626126,-0.7383427623
H,0,-1.3877233997,4.5861869088,-1.3409398082
H,0,-2.7008383043,3.3869859131,-1.6026202243
C,0,0.6650667722,0.1043190245,2.7923133107
C,0,0.4780125405,-1.1777456571,3.3730576801

C,0,1.4770027664,-1.7630504177,4.1614133747
C,0,2.6374739398,-1.0213235746,4.3812031626
C,0,2.819623104,0.2672147477,3.8375622333
C,0,1.8395997716,0.8397353891,3.0384291504
C,0,-0.4961871676,0.3873453992,2.0103200199
C,0,-1.4057963614,-0.6561988808,2.1802840953
H,0,1.3559410521,-2.7584006986,4.5799420337
H,0,3.4273008809,-1.4549526493,4.9986815093
H,0,3.7416819831,0.8150550093,4.0393429812
H,0,1.9750198222,1.8294852497,2.6016678758
H,0,-0.6635876648,1.3036581056,1.4535472551
N,0,-0.8042702845,-1.6253092313,3.0211785374
C,0,-1.3342155646,-2.8904196978,3.3240333833
C,0,-0.9637292783,-5.0907588455,3.7552932834
C,0,-3.1540716115,-4.1900952388,3.7259908256
C,0,-2.3348231705,-5.3067705235,3.9016792474
H,0,-0.2443371474,-5.9083595041,3.8711386583
H,0,-4.2429217493,-4.264358777,3.8185779632
H,0,-2.7421177433,-6.2894301064,4.1409276705
N,0,-0.4617901815,-3.8918203109,3.4543012304
N,0,-2.6590480944,-2.9830481896,3.4483032167
C,0,-6.7939906442,1.4182536891,0.0986978387
C,0,-7.0416321636,0.3396949072,-0.7572033409
C,0,-6.0400941082,-0.6022786688,-1.0259963915
C,0,-4.8058960315,-0.4487694513,-0.3933186111
C,0,-4.5541505337,0.6439899478,0.4535787916
C,0,-5.537463754,1.5864516493,0.7064050846
C,0,-3.6356226329,-1.3197612783,-0.523505669
C,0,-2.8971885724,-1.7673362837,0.6459584657
C,0,-2.8513755428,-0.6767130986,1.7196094198
C,0,-3.1025896391,0.5867074591,0.8426467575
O,0,-2.4067352278,0.2669843569,-0.3831839276
H,0,-7.5810429479,2.1537393622,0.2784627746
H,0,-8.0187097235,0.241896444,-1.2345377862
H,0,-6.2207537948,-1.4363812596,-1.7070799161
H,0,-5.3399494669,2.4519778449,1.3428135156
H,0,-3.5237804157,-1.848841638,-1.4694050543
H,0,-2.9800150829,-2.817495542,0.9276824393
H,0,-3.5580259846,-0.7715705101,2.5557479086
H,0,-2.7284868215,1.5184392396,1.2837758318
C,0,-4.2856711755,0.9475959229,-4.7949813734
C,0,-4.6220216815,-0.1943948062,-5.7592181931
H,0,-5.1572299397,0.2007638037,-6.6357168265
H,0,-5.2582662883,-0.9498477272,-5.2754821623
H,0,-3.7113517495,-0.7021587697,-6.1086410671
C,0,-3.381244004,1.9872177522,-5.4866581199
H,0,-2.4424239737,1.5279047603,-5.834326883
H,0,-3.1298706698,2.8132802042,-4.8068806096
H,0,-3.8984517265,2.403692387,-6.36464951
C,0,-5.5781599861,1.6258613425,-4.2957798835
H,0,-5.3549141265,2.4505504642,-3.6045298952
H,0,-6.2276240704,0.9070677154,-3.7721633733
H,0,-6.1396836815,2.0301396434,-5.1519684956
C,0,-3.5452822359,0.3861319502,-3.5829014693
O,0,-3.2986276566,-0.7981975491,-3.4207313351
O,0,-3.1978689459,1.3201508628,-2.7062530654

H,0,-2.7832552488,0.9281362252,-1.8885226642
H,0,-2.0184466453,3.6340457455,0.0411528436

TS-3-*trans*-*RR* [named as TS-*trans*-*RR* in the main text]
Opt @ B3LYP-D3(BJ)/def2-SVP in gas phase
SCF Done: E(RB3LYP) = -4588.24632640 a.u.
Imaginary frequency = -278.1746 cm^{-1}
Zero-point correction = 1.169027 Hartree/Particle
Sum of electronic and thermal Free Energies = -4587.181524 a.u.
Sp @ RI-PWPB95-D3(BJ)/def2-TZVPP in 2,2,2-trifluoroethanol
FINAL SINGLE POINT ENERGY = 4589.311045246756 a.u.

O,0,4.9711653149,1.1067309911,-0.6380115816
O,0,-0.8780055415,-2.2294870568,2.6606329744
C,0,1.3022100643,2.7984930693,1.6874256783
C,0,1.7797762893,2.2784957804,0.4415659399
C,0,1.8772584415,0.86540761,0.5105689737
C,0,1.4218099351,0.4670764566,1.8220613885
C,0,1.0764521184,1.668018152,2.5158396834
C,0,1.1953101193,4.2508324967,2.118284955
C,0,1.6463682775,5.1832904029,0.9859874524
C,0,-0.2476533016,4.5784919192,2.536929487
C,0,2.1324510343,4.4508163412,3.3289873935
C,0,2.4829458783,-0.0218090703,-0.5418674626
C,0,3.6788646229,-0.7374855526,0.033658334
C,0,4.958929486,-0.1042847651,-0.0132684553
C,0,6.0651983004,-0.7100835039,0.5451274678
C,0,5.9386370587,-1.9694474278,1.1961936673
C,0,7.0583867825,-2.6144660431,1.7880173896
C,0,6.920732805,-3.8287417989,2.4281067946
C,0,5.6527243095,-4.4524302988,2.5080432409
C,0,4.5465038365,-3.8519900542,1.942457379
C,0,4.6569023461,-2.6049343214,1.2702541087
C,0,3.5267962718,-1.9555945868,0.6711604137
C,0,2.1716548098,-2.5847102198,0.7403313032
C,0,1.8480710928,-3.6567236675,-0.1565268141
C,0,2.7665293615,-4.1428862055,-1.1270660039
C,0,2.4026504904,-5.1369209699,-2.0122740087
C,0,1.0985750801,-5.6873457395,-1.9698568286
C,0,0.1871466906,-5.2400207689,-1.0374006331
C,0,0.5342558722,-4.2249951448,-0.1045172097
C,0,-0.3939267618,-3.7650905038,0.8644744437
C,0,-0.0526384106,-2.7388484965,1.721099251
C,0,1.2278705624,-2.0952933488,1.6307644563
C,0,1.4865098435,-0.8812015848,2.4970885793
C,0,6.173368289,1.8379165122,-0.6795454953
C,0,-2.0744820226,-2.9105949177,3.0012625641
H,0,2.0304418591,2.8642024236,-0.4404010268
H,0,0.6495275483,1.6715076223,3.5144454351
H,0,2.6941882129,4.9909166075,0.7063654428
H,0,1.5714140566,6.2329633571,1.3083467155
H,0,1.0281399878,5.0661050521,0.0897499434
H,0,-0.9475347834,4.4081422797,1.7098921587
H,0,-0.3250691874,5.6298089596,2.8568993197
H,0,-0.5670125829,3.9433938369,3.3749605046

H,0,1.8311804553,3.8202593135,4.1792483484
H,0,2.1070326722,5.5013193205,3.6604451524
H,0,3.17265938,4.1979186411,3.0697825699
H,0,2.7867451957,0.5928284959,-1.3945335357
H,0,1.7649189335,-0.7704744895,-0.893469711
H,0,7.0488081357,-0.2424823157,0.5093378178
H,0,8.0358466894,-2.1283285783,1.7291898027
H,0,7.7918281881,-4.3110640271,2.8781758178
H,0,5.5507074898,-5.4118987821,3.0200900277
H,0,3.5682833062,-4.3306210091,2.0028559173
H,0,3.7687327487,-3.7137302498,-1.1681394236
H,0,3.1206974299,-5.4978565696,-2.752638211
H,0,0.814544545,-6.4653470365,-2.6826370841
H,0,-0.8244226279,-5.6513318537,-1.0055085094
H,0,-1.3980787404,-4.1866632697,0.875063813
H,0,0.7633553005,-0.862909934,3.3207781753
H,0,2.4988034627,-0.9577600387,2.9314808026
H,0,6.5541776027,2.0518289791,0.3346719365
H,0,5.946722013,2.7870123227,-1.1835895413
H,0,6.957944251,1.304166107,-1.2456006149
H,0,-1.8516542066,-3.9336399802,3.3529729678
H,0,-2.775124822,-2.969886252,2.1543783687
H,0,-2.521772394,-2.3269692876,3.8155607433
Co,0,-0.1355853877,1.439107577,0.8075855561
C,0,-1.0153756677,4.0144121579,-1.2478840198
C,0,0.0393090802,3.9537649247,-2.1919973767
C,0,0.6207653199,5.1068714569,-2.7239556519
C,0,0.1023848338,6.3358826768,-2.311201117
C,0,-0.9631396329,6.4141934801,-1.3951034085
C,0,-1.5284306354,5.2598290842,-0.8579013464
C,0,-1.3672082203,2.6540822183,-0.9100864495
C,0,-0.5912761154,1.8077396495,-1.7028547762
H,0,1.4502714657,5.0506575383,-3.4258596754
H,0,0.535345755,7.2554814116,-2.7110579844
H,0,-1.346145599,7.3934362555,-1.0998957886
H,0,-2.3548603941,5.314814765,-0.1470162504
H,0,-2.2266017473,2.3488306829,-0.3228481774
N,0,0.2808620762,2.5920380172,-2.4679737
C,0,1.3829055355,2.1010315211,-3.1908121978
C,0,3.5913871457,2.2768941126,-3.6938449487
C,0,2.2767064876,0.4818036216,-4.5082431514
C,0,3.5295649367,1.0925140023,-4.4296991921
H,0,4.5289923639,2.8332675902,-3.5910336197
H,0,2.1278872881,-0.4431568864,-5.0755495394
H,0,4.4074354571,0.6719985806,-4.920944317
N,0,2.5278155085,2.7786440158,-3.0665547315
N,0,1.2005420973,0.9917407639,-3.9077611883
C,0,-5.4885803925,0.8518193363,-0.6084456232
C,0,-5.3489323281,1.166289604,-1.9617964428
C,0,-4.1656337795,0.8489884641,-2.652526571
C,0,-3.1264021557,0.2426406691,-1.9674118339
C,0,-3.2508647496,-0.0415371093,-0.5896487046
C,0,-4.4403112657,0.2297097644,0.0825520105
C,0,-1.8328835265,-0.3661791657,-2.472347957
C,0,-0.6390848751,0.3163845656,-1.7130739618
C,0,-0.7574718708,-0.1904271154,-0.273489342

C,0,-2.068827707,-0.6619525127,0.0038716526
O,0,-1.8158991563,-1.6754935568,-2.0269834768
H,0,-6.421775186,1.0784617347,-0.0883126855
H,0,-6.1791731926,1.6332509786,-2.4973031839
H,0,-4.0787077425,1.0500540561,-3.7229962229
H,0,-4.5433365614,-0.0346179987,1.1335918334
H,0,-1.6962418739,-0.2557740214,-3.5642395672
H,0,0.269405118,-0.0899583277,-2.1583567696
H,0,-0.0207390112,-0.9428084811,-0.0055919835
H,0,-2.2198042913,-1.3576438246,0.825920203
C,0,-5.801018631,-3.8208569923,-0.855247039
C,0,-4.3689181232,-3.2553606773,-0.8542329896
O,0,-3.7111209114,-3.1496703856,0.1758373946
O,0,-3.9550963031,-2.9026609105,-2.0396791808
H,0,-3.0053631625,-2.3993161727,-2.029990165
C,0,-6.7440026644,-2.7340922872,-1.4056388257
H,0,-6.4497560863,-2.4421204346,-2.42327252
H,0,-7.7816798655,-3.1055798462,-1.4279870991
H,0,-6.7159200783,-1.8310556412,-0.776219302
C,0,-5.839100313,-5.0558017233,-1.7742088017
H,0,-5.1666328305,-5.8460490968,-1.402805118
H,0,-6.8596984796,-5.4704732681,-1.8140382918
H,0,-5.526092261,-4.7918060801,-2.7940717026
C,0,-6.2092133245,-4.2045736628,0.5699975819
H,0,-7.2324857751,-4.6134642996,0.5762602307
H,0,-5.5283777475,-4.960967064,0.9873146214
H,0,-6.1789691708,-3.3313128858,1.2382296742
C,0,-3.3384875486,1.9210948502,3.5767301696
C,0,-2.0355347331,1.3286727728,2.9712038854
O,0,-1.3439095636,0.5864402005,3.6600868786
O,0,-1.7753451221,1.7087698426,1.7583035118
C,0,-4.0609024693,2.8676334844,2.6124642066
H,0,-4.3518407942,2.3596384711,1.6838274842
H,0,-4.9712223128,3.2697516571,3.0871832178
H,0,-3.4152941952,3.714538403,2.3366356894
C,0,-4.245709004,0.7441269557,3.9779724251
H,0,-3.714909282,0.0753422128,4.6701724456
H,0,-5.1593058725,1.1126543107,4.4719962398
H,0,-4.553176647,0.1492862657,3.1047674731
C,0,-2.9363662263,2.6843355933,4.8532584427
H,0,-3.8336391019,3.0535574656,5.376022281
H,0,-2.3759175724,2.0258050951,5.5314653277
H,0,-2.3052971267,3.5551223211,4.616241973

TS-3-*trans-SS* [named as TS-*trans-SS* in the main text]
Opt @ B3LYP-D3(BJ)/def2-SVP in gas phase
SCF Done: E(RB3LYP) = -4588.24493649 a.u.
Imaginary frequency = -306.1894 cm^{-1}
Zero-point correction = 1.168779 Hartree/Particle
Sum of electronic and thermal Free Energies = -4587.178620 a.u.
Sp @ RI-PWPB95-D3(BJ)/def2-TZVPP in 2,2,2-trifluoroethanol
FINAL SINGLE POINT ENERGY = -4589.311316451352 a.u.

Co,0,0.584114403,0.4441737855,-0.070861975
O,0,-3.0457273054,3.9759173756,0.5803860875

```
O,0,-1.8411213194,-3.1260498756,-1.1176945669
C,0,0.4135305436,2.1609188211,-1.2358382086
C,0,-0.382082115,2.2984253685,-0.0607778677
C,0,-1.3945972318,1.310684244,-0.0322910137
C,0,-1.2657781573,0.5345075081,-1.2366867083
C,0,-0.1625832827,1.0570767731,-1.9597538271
C,0,1.4381449948,3.1812633564,-1.7048037166
C,0,2.3018092894,3.6739050636,-0.5311689875
C,0,2.3612877362,2.6456890166,-2.8103528225
C,0,0.613982877,4.3629685856,-2.2701453892
C,0,-2.5547250575,1.3113909472,0.9173002488
C,0,-3.7316003005,1.7831006083,0.0907518268
C,0,-3.915910229,3.1905610265,-0.1037340261
C,0,-4.9025489361,3.6665537605,-0.9428189184
C,0,-5.7371509399,2.7633995964,-1.657299784
C,0,-6.7489862708,3.2268695911,-2.5413490104
C,0,-7.5375968642,2.3404131342,-3.2452806045
C,0,-7.345654988,0.9461313973,-3.0975889614
C,0,-6.3738521032,0.4654674289,-2.2434489409
C,0,-5.5506596416,1.3527903284,-1.4985700861
C,0,-4.5294497192,0.8890395506,-0.603049198
C,0,-4.3221105397,-0.5754209385,-0.3982834638
C,0,-5.2711166218,-1.2968561845,0.400701037
C,0,-6.3878548078,-0.6656702789,1.0158227031
C,0,-7.2854434825,-1.3910106828,1.7710007796
C,0,-7.1073818028,-2.7846404588,1.9492229061
C,0,-6.0293007932,-3.4237747733,1.3754440415
C,0,-5.0845905774,-2.7016249509,0.5951162468
C,0,-3.9436855491,-3.332684347,0.0388178648
C,0,-3.0099979291,-2.6012148682,-0.6679185606
C,0,-3.2052052819,-1.2063757008,-0.930150924
C,0,-2.2226166358,-0.476707864,-1.8263494689
C,0,-3.1126762366,5.3740147636,0.4214410585
C,0,-1.4644434178,-4.4073583823,-0.6481364251
H,0,-0.2304025272,3.0155753651,0.7392709189
H,0,0.1554555673,0.6955327191,-2.9331481873
H,0,1.6869825015,4.1230124207,0.2625055672
H,0,3.0037804753,4.4441089837,-0.8861140982
H,0,2.8904642029,2.8558756077,-0.0973978263
H,0,3.0893272079,1.9232123409,-2.4191335198
H,0,2.9424117873,3.4792736726,-3.2335666049
H,0,1.7923690412,2.1958648698,-3.6397941778
H,0,-0.0034653427,4.0431710264,-3.1247333203
H,0,1.2845026154,5.1677423161,-2.6117851373
H,0,-0.0598877332,4.7774529225,-1.5039809124
H,0,-2.3324135863,1.9852729039,1.7488920865
H,0,-2.7393272548,0.3189598468,1.3354065037
H,0,-5.0540303213,4.7356572783,-1.0883390226
H,0,-6.8902401788,4.3047166681,-2.6571158205
H,0,-8.3106835087,2.7135609253,-3.9213061643
H,0,-7.9696123902,0.2489493176,-3.6614722815
H,0,-6.2283138367,-0.6095738165,-2.1296856212
H,0,-6.5266524175,0.4075034393,0.8847821683
H,0,-8.1362649981,-0.8886784965,2.2367075348
H,0,-7.8240829174,-3.3510365328,2.5487451828
H,0,-5.8796666867,-4.4970044132,1.5185300662
```

H,0,-3.7975555884,-4.394431573,0.2285688639
H,0,-1.6430756804,-1.2154737847,-2.3948928269
H,0,-2.8180106833,0.0891865571,-2.5645403753
H,0,-2.9464202126,5.6701992193,-0.6296697009
H,0,-2.3161038812,5.7982927142,1.0463396115
H,0,-4.0858280178,5.7757703544,0.7548504432
H,0,-2.1144139514,-5.195710572,-1.0655921343
H,0,-1.502554511,-4.4466946654,0.4531695597
H,0,-0.4378682762,-4.568183496,-0.9861430293
C,0,-0.4019173854,-1.8207080015,1.9979947808
C,0,0.5650736182,-1.9928770993,3.0154968072
C,0,0.2107555995,-2.194817029,4.3511099718
C,0,-1.1503240353,-2.2271053311,4.6593945509
C,0,-2.1260470622,-2.0664070707,3.6637249143
C,0,-1.7598949537,-1.8658934007,2.3347553079
C,0,0.3169095687,-1.6240072894,0.7468917628
C,0,1.6870259154,-1.7338810657,1.039702636
H,0,0.9685884358,-2.3048343081,5.1208537191
H,0,-1.4552465289,-2.3736571932,5.6980227418
H,0,-3.1853238846,-2.0919108386,3.9287107386
H,0,-2.5222418639,-1.7444948905,1.573174848
H,0,-0.0918951786,-1.819798427,-0.2447207197
N,0,1.8427207813,-1.9534762705,2.3951291672
C,0,3.0808412208,-2.0757977953,3.0566606431
C,0,4.3081726346,-1.8384557564,4.952307481
C,0,5.3077437652,-2.5156053615,2.9162741343
C,0,5.4706559327,-2.1831422637,4.2624898241
H,0,4.331431423,-1.5902461044,6.0188807233
H,0,6.1547106773,-2.8190414746,2.2925606123
H,0,6.446555451,-2.2043368029,4.7481162532
N,0,3.118825256,-1.7736893529,4.3539137447
N,0,4.1154562502,-2.4906011356,2.3246503542
C,0,1.1088804793,-4.768530769,-2.9072866992
C,0,0.5087585084,-3.7720281862,-3.6781886011
C,0,0.8354170298,-2.4250008361,-3.4656982605
C,0,1.7447270931,-2.0943926355,-2.4618411287
C,0,2.3326928687,-3.1035711543,-1.6662954036
C,0,2.0224232288,-4.4359398181,-1.8904739866
C,0,2.259567929,-0.7513929811,-2.1646426299
C,0,2.3508379875,-0.2724493919,-0.8390424221
C,0,2.7965545766,-1.3859481176,0.1117224147
C,0,3.4223133642,-2.4840001963,-0.8198813741
O,0,4.2064848862,-1.8061414823,-1.7449482812
H,0,0.8734815309,-5.8179191815,-3.1014563699
H,0,-0.2013442198,-4.0411973038,-4.4629411034
H,0,0.386962516,-1.6465839242,-4.0880817296
H,0,2.5108122288,-5.2201436643,-1.3067828737
H,0,2.3816075144,-0.0537119076,-2.9956291446
H,0,2.946378626,0.6354259731,-0.7468351873
H,0,3.6235432163,-0.9805178227,0.6995615652
H,0,3.9785415106,-3.2148563881,-0.2098487718
C,0,6.9907283744,1.3105171631,0.4895580328
C,0,7.1178223032,0.532771991,1.8118335321
H,0,7.3403106657,-0.5255829433,1.6178746046
H,0,7.9293303895,0.9558344263,2.4264543385
H,0,6.1867228995,0.5863442717,2.3963282543

C,0,8.2898602958,1.1577306006,-0.3254475431
H,0,8.4861140547,0.0998011001,-0.5516064688
H,0,8.2230672174,1.7058555814,-1.2788205335
H,0,9.1453182,1.5624260481,0.2400186085
C,0,6.7121324493,2.7911538623,0.7663571173
H,0,6.5811190473,3.3481138365,-0.172060653
H,0,5.7927418579,2.9186588589,1.3555951541
H,0,7.5487116248,3.2370493312,1.3283850006
C,0,5.8420588781,0.7074781889,-0.3444757604
O,0,4.9443265514,1.4020763872,-0.8056128335
O,0,5.949514922,-0.5855829829,-0.5032016985
H,0,5.1652394601,-1.0551447316,-1.1040019799
C,0,2.192353617,1.9857850002,3.5151198183
C,0,1.8544492536,1.4592718986,4.9181603769
H,0,1.9764812854,0.3676719137,4.9681152378
H,0,2.5191361534,1.9180198852,5.669277495
H,0,0.8119789842,1.6973235832,5.1705546886
C,0,3.6278855639,1.621080459,3.1215671685
H,0,3.7809895541,0.5336172952,3.1273175682
H,0,3.8672867603,1.9754433129,2.110550161
H,0,4.3449282938,2.0662962117,3.8308892426
C,0,2.0227736221,3.5177483264,3.4848727203
H,0,2.2640638515,3.9224033155,2.4897037446
H,0,0.9874501714,3.7940953037,3.7338544809
H,0,2.6980242633,3.992375876,4.2156029092
C,0,1.152017002,1.4186007079,2.5161101375
O,0,1.6667081955,0.9812801804,1.4070325724
O,0,-0.0435573679,1.4606321868,2.7838414756

***cis*-3aa**
Opt @ B3LYP-D3(BJ)/def2-SVP in gas phase
SCF Done: E(RB3LYP) = -1087.37035690 a.u.[1]
Zero-point correction = 0.342572 Hartree/Particle
Sum of electronic and thermal Free Energies = -1087.076805 a.u.
Sp @ RI-PWPB95-D3(BJ)/def2-TZVPP in 2,2,2-trifluoroethanol
FINAL SINGLE POINT ENERGY = -1087.694295038172 a.u.

C,0,1.2146881428,-0.4342732373,-4.4737020777
C,0,-0.0224476383,-0.6345896448,-3.8077327193
C,0,-0.7130878722,-1.8477015232,-3.9138500244
C,0,-0.1437825763,-2.8544867692,-4.695932895
C,0,1.084637895,-2.6713442831,-5.3554153095
C,0,1.7696120504,-1.4639038374,-5.2486600756
C,0,1.6539761149,0.9009008402,-4.1693672043
C,0,0.71732119,1.4980625275,-3.3742302706
H,0,-1.6500901271,-2.0038834658,-3.3880882103
H,0,-0.6658920143,-3.8100791365,-4.7870410807
H,0,1.5028377233,-3.4842878605,-5.9538154929
H,0,2.7233360078,-1.3115710019,-5.7594448068
H,0,2.5941988996,1.362101516,-4.4532027591
N,0,-0.3382279638,0.5705659907,-3.1388102802
C,0,-1.5266669854,0.8096452769,-2.4463523379
C,0,-3.314183071,-0.026677594,-1.3072233082
C,0,-3.0513654359,2.2979984092,-1.6338988315
C,0,-3.8097870617,1.2602245218,-1.0897602724

H,0,-3.8453956284,-0.9079453567,-0.9289434252
H,0,-3.3605743784,3.3439822861,-1.5253717284
H,0,-4.7320402686,1.4420341557,-0.537241668
N,0,-2.1820747211,-0.255956794,-1.96655657
N,0,-1.9318273775,2.0797706758,-2.3167946197
C,0,2.2082255191,7.5177499095,-2.6167354546
C,0,1.980991892,7.55499289,-1.2389138227
C,0,1.6681059267,6.3811869381,-0.5492346072
C,0,1.5751504859,5.1540551297,-1.223186112
C,0,1.8168696806,5.1192606884,-2.6179225548
C,0,2.1298054649,6.297177597,-3.2978140594
C,0,1.2215553236,3.910646189,-0.5346574272
C,0,0.8589013448,2.8178384004,-1.2250013698
C,0,0.7715002211,2.8593783473,-2.7380658528
C,0,1.8596542256,3.7717858518,-3.3127358582
O,0,3.1436476608,3.1818538089,-3.1253117967
H,0,2.4468055905,8.4344591709,-3.1609125614
H,0,2.0451629287,8.5027638555,-0.6988831725
H,0,1.4845450159,6.4121042511,0.5281330089
H,0,2.3120699494,6.2605146678,-4.3760714011
H,0,-0.1795314756,3.3678492338,-2.9685873773
H,0,1.2391018417,3.9040861265,0.5586408351
H,0,0.5887923734,1.8948974553,-0.7072016944
H,0,1.6606454514,3.9105012503,-4.3925592865
H,0,3.7993620956,3.8882698533,-3.1859105173

Acetate anion
Opt @ B3LYP-D3(BJ)/def2-SVP in gas phase
SCF Done: E(RB3LYP) = -228.338549359 a.u.
Zero-point correction = 0.047751 Hartree/Particle
Sum of electronic and thermal Free Energies = -228.318298 a.u.
Sp @ RI-PWPB95-D3(BJ)/def2-TZVPP in 2,2,2-trifluoroethanol
FINAL SINGLE POINT ENERGY = -228.571267587488 a.u.

C,0,0.1998805071,1.6943061756,0.0026848859
H,0,0.1183537371,2.0174201981,-1.051603588
H,0,-0.4795782464,2.3486148604,0.5795671331
H,0,-0.1359794565,0.6472275135,0.0921793342
C,0,1.6833171659,1.8901388134,0.5066485014
O,0,2.255627301,0.8608316734,0.925278926
O,0,2.1046492119,3.0650887755,0.4226168074

Int-0’
Opt @ B3LYP-D3(BJ)/def2-SVP in gas phase
SCF Done: E(RB3LYP) = -2627.29691726 a.u.
Zero-point correction = 0.464885 Hartree/Particle
Sum of electronic and thermal Free Energies = -2626.891421 a.u.
Sp @ RI-PWPB95-D3(BJ)/def2-TZVPP in 2,2,2-trifluoroethanol
FINAL SINGLE POINT ENERGY = 2627.873764569340 a.u.

Co,0,-1.5828651212,-0.0920412211,0.0360432684
C,0,3.3777563276,-1.3960492705,0.5057243043

```
C,0,3.0758556139,-0.160699276,-0.1238687869
C,0,3.9381729209,0.4137594444,-1.0618110526
C,0,5.1158486711,-0.2755769974,-1.3590102895
C,0,5.4331937676,-1.4968106846,-0.7390464602
C,0,4.5704899635,-2.0650153203,0.1951482349
C,0,2.2784807708,-1.7098243878,1.3916385862
C,0,1.3500829993,-0.7227727636,1.2763755983
H,0,3.7132152092,1.3695801221,-1.5261570341
H,0,5.8105815033,0.1547638892,-2.0833802813
H,0,6.3679434756,-2.0012750003,-0.99190177
H,0,4.8129766363,-3.01361995,0.678659894
H,0,2.2105403545,-2.563547575,2.062744213
N,0,1.7987519546,0.2401607144,0.3499524981
C,0,1.1111607224,1.3745836909,-0.0112006397
C,0,1.2290431999,3.5496664724,-0.7281441809
C,0,-0.8335209207,2.6241163336,-0.0473335953
C,0,-0.1294475029,3.7451682973,-0.4525754371
H,0,1.8492002982,4.3509627867,-1.1446420453
H,0,-1.9088147905,2.6557872769,0.1321307621
H,0,-0.6230872806,4.7068164359,-0.5900566407
N,0,1.8288529577,2.3930757676,-0.4915642145
N,0,-0.2443811238,1.4193248822,0.1303440402
H,0,0.4194570414,-0.5784067807,1.812027928
C,0,-2.4691933183,0.3497836532,2.1795389644
O,0,-1.5821175754,-0.5395950997,1.9913669638
O,0,-2.8686816499,0.9519548483,1.1293926869
C,0,-2.9837863834,0.6913530042,3.5376984137
C,0,-1.6527621811,-0.1642033532,-2.0403508096
C,0,-2.9491613712,-0.4098513769,-1.5027084925
C,0,-2.8540896933,-1.5418330488,-0.6157052727
C,0,-1.5081065821,-2.0530127015,-0.6943894888
C,0,-0.7605424546,-1.2036269387,-1.5577725642
C,0,-1.2678696802,0.9219065796,-2.9903628567
H,0,-1.3412822571,0.558993464,-4.0292838624
H,0,-1.9215593677,1.7991458437,-2.8973653685
H,0,-0.2311019979,1.2486811396,-2.8316319658
H,0,-2.979308246,-0.1983531388,4.1811734199
H,0,-2.3116120235,1.4396317412,3.9895263083
H,0,-3.989886503,1.1248745297,3.4714242601
C,0,-4.1774215949,0.4106837707,-1.7032622084
H,0,-3.979365879,1.3106866465,-2.2988611353
H,0,-4.9399757432,-0.1849431401,-2.231118757
H,0,-4.6036128576,0.7189906178,-0.736948838
C,0,-3.9701905515,-2.1275934298,0.1839069852
H,0,-4.7085706748,-1.3632654733,0.4616667866
H,0,-4.495048031,-2.9023362902,-0.4001318778
H,0,-3.5978830885,-2.5994996357,1.1032873552
C,0,-0.9983718647,-3.2226162131,0.0757869522
H,0,-1.2716893626,-3.1364628208,1.1381005713
H,0,-1.4513238609,-4.1501921441,-0.3115269121
H,0,0.0919800213,-3.3114741747,0.008603176
C,0,0.6421367117,-1.4142044037,-2.0179990654
H,0,1.2505793216,-1.9443127077,-1.2765663767
H,0,0.6253285794,-2.024914316,-2.9371190088
H,0,1.1481108415,-0.4736191782,-2.2669739222
----------------------------------------------------------------
```

TS-1′

Opt @ B3LYP-D3(BJ)/def2-SVP in gas phase
SCF Done: E(RB3LYP) = -2627.27993382 a.u.
Imaginary frequency = -217.9088 cm^{-1}
Zero-point correction = 0.460633 Hartree/Particle
Sum of electronic and thermal Free Energies = -2626.877281 a.u.
Sp @ RI-PWPB95-D3(BJ)/def2-TZVPP in 2,2,2-trifluoroethanol
FINAL SINGLE POINT ENERGY = -2627.850443837798 a.u.

```
Co,0,-1.3729935552,-0.446979506,0.5183845507
C,0,-3.7059215558,0.8832687724,-2.7246507817
C,0,-4.5875664487,0.5016321419,-1.6699344245
C,0,-5.9542174219,0.2899638003,-1.8816874023
C,0,-6.4239562946,0.4659420986,-3.1814061837
C,0,-5.5705742146,0.8473472245,-4.2416190242
C,0,-4.2166233658,1.0626644639,-4.0263981025
C,0,-2.3973193421,1.009321448,-2.1622432018
C,0,-2.4442137782,0.7161776107,-0.8054027956
H,0,-6.6134877664,0.015668538,-1.0606625028
H,0,-7.4860325363,0.3129662358,-3.3850020837
H,0,-5.9892343127,0.9786868852,-5.2412040191
H,0,-3.5574913406,1.3635067219,-4.8432059354
H,0,-1.5122493608,1.3573291148,-2.6909432737
N,0,-3.8127364289,0.3990015472,-0.5208111985
C,0,-4.1364451033,0.0118834755,0.7497554087
C,0,-5.5862331793,-0.3265987084,2.4603668966
C,0,-3.2449045189,-0.5178348307,2.815989341
C,0,-4.5267777763,-0.5335921828,3.3541103675
H,0,-6.626103003,-0.3833727762,2.7983852894
H,0,-2.350239185,-0.7002432585,3.4146950239
H,0,-4.6961422701,-0.7234596072,4.4136924288
N,0,-5.3937019535,-0.0428178259,1.172950245
N,0,-3.0531763245,-0.288394705,1.5111395194
H,0,-1.9770629184,1.6080723894,-0.0671562573
C,0,-0.8054307325,2.1146432813,1.6290976187
O,0,-1.5750348065,2.6308692192,0.788718612
O,0,-0.560516202,0.8601929149,1.6907662001
C,0,-0.0835421112,2.9994906374,2.6139166171
C,0,-1.4033271408,-2.5571686324,0.6766383087
C,0,-0.2328490312,-2.0073318338,1.2898398882
C,0,0.5191955257,-1.3248749548,0.2601338028
C,0,-0.2079170313,-1.4102476626,-0.9551664449
C,0,-1.4337186998,-2.1401141762,-0.6915123798
C,0,-2.4442290149,-3.3945156548,1.3423231831
H,0,-2.2367225679,-4.4598097881,1.1456220255
H,0,-2.4541273432,-3.2591867206,2.4315189598
H,0,-3.4508276669,-3.1830252176,0.9560445195
H,0,0.8515117777,3.3475229324,2.1450293971
H,0,-0.6924363477,3.8829049492,2.845119276
H,0,0.1723162833,2.4457643509,3.5260942848
C,0,0.2071856015,-2.1344665208,2.7112862481
H,0,-0.5702549227,-2.5812643195,3.3456636405
H,0,1.1010178635,-2.7754950031,2.7856596143
H,0,0.470354508,-1.1478888445,3.1212376234
C,0,1.8269172461,-0.6436773154,0.4788122583
```

H,0,1.7827470254,0.0006307277,1.3682090424
H,0,2.6187068198,-1.3934924674,0.6427992089
H,0,2.117749393,-0.0284399682,-0.3824230772
C,0,0.2653262975,-0.9236188349,-2.2863894231
H,0,0.696326015,0.085699183,-2.2272809083
H,0,1.0560713602,-1.5953664194,-2.6604067434
H,0,-0.5419231842,-0.9133843537,-3.0276987316
C,0,-2.4709343854,-2.5217516307,-1.697796623
H,0,-2.5339238655,-1.7912733847,-2.5138751801
H,0,-2.2331729979,-3.5035434424,-2.1406366745
H,0,-3.4686275807,-2.6002511979,-1.2437800874

INT-1′
Opt @ B3LYP-D3(BJ)/def2-SVP in gas phase
SCF Done: E(RB3LYP) = -2627.29655404 a.u.
Zero-point correction = 0.465097 Hartree/Particle
Sum of electronic and thermal Free Energies = -2626.891747 a.u.
Sp @ RI-PWPB95-D3(BJ)/def2-TZVPP in 2,2,2-trifluoroethanol
FINAL SINGLE POINT ENERGY = -2627.869001211833 a.u.

Co,0,-0.9476954836,-0.2052039811,-0.2614804175
C,0,2.8771221931,-1.1171433325,1.3086584965
C,0,2.9727243004,0.1779067157,0.7383803833
C,0,4.1596737269,0.9115279141,0.7357932437
C,0,5.2755738727,0.3094030824,1.3225462949
C,0,5.2049419447,-0.9746908818,1.8919458698
C,0,4.011802744,-1.6968578279,1.8925422451
C,0,1.5067942781,-1.5688927693,1.132234383
C,0,0.7897618637,-0.5939756937,0.4832566025
H,0,4.2052199967,1.9056719423,0.2963332212
H,0,6.223853008,0.850716065,1.3381142975
H,0,6.0997167459,-1.4106981831,2.3409407825
H,0,3.9611679041,-2.6927287478,2.3377752061
H,0,1.1230631761,-2.5320255858,1.4611552054
N,0,1.6909938275,0.4818164895,0.2451023414
C,0,1.1801784989,1.6397513493,-0.2545312764
C,0,1.3051819172,3.8340004924,-0.8261587043
C,0,-0.7752284,2.6930171117,-0.8976882944
C,0,-0.075720441,3.883008396,-1.0598195528
H,0,1.9304449998,4.722232099,-0.9656385443
H,0,-1.8517002183,2.6260886813,-1.06071257
H,0,-0.5783761165,4.8008513025,-1.3632488429
N,0,1.9248906279,2.7297839792,-0.4180140385
N,0,-0.1526814073,1.566125867,-0.5290463707
H,0,0.1372425626,-0.3833108888,2.3001709448
C,0,-1.5334577839,0.4911589314,2.5763482261
O,0,-0.4208702324,-0.0044010553,3.0392309348
O,0,-1.8497748206,0.4777784172,1.3775730106
C,0,-2.4322160151,1.0873023444,3.6134703006
C,0,-1.8893523459,-0.2582507762,-2.1944577896
C,0,-2.8340924397,-0.592747031,-1.2003814447
C,0,-2.3283453884,-1.7543281796,-0.483421383
C,0,-1.0956694428,-2.1432216274,-1.0719545154
C,0,-0.7571346092,-1.152466434,-2.0642147821
C,0,-1.9989894208,0.8181335539,-3.2229520235

H,0,-2.2880341815,0.3679289813,-4.187652631
H,0,-2.7634821023,1.5646423327,-2.9712262193
H,0,-1.042828847,1.3343707745,-3.3858630368
H,0,-2.7298711818,0.3025544256,4.3265965404
H,0,-1.8775503794,1.8458579993,4.1862802767
H,0,-3.3188592708,1.5301874704,3.1472221251
C,0,-4.1296691001,0.0860806157,-0.896711841
H,0,-4.2619901243,1.0021965773,-1.4877595248
H,0,-4.979484967,-0.5794851469,-1.1193278703
H,0,-4.1889121983,0.355152685,0.1686893392
C,0,-3.0488733759,-2.4500276976,0.6244155453
H,0,-3.5876943766,-1.7347781203,1.2611449049
H,0,-3.7945978273,-3.1518768138,0.2140637436
H,0,-2.3624852658,-3.0289605236,1.257314986
C,0,-0.3351676987,-3.3950269954,-0.7909569434
H,0,-0.4788471736,-3.7462592854,0.2392816046
H,0,-0.7057680759,-4.1894309622,-1.4613736412
H,0,0.7392658804,-3.2764032387,-0.9727327608
C,0,0.4588016918,-1.1619013395,-2.9331604663
H,0,1.324006837,-1.5817999048,-2.4023459182
H,0,0.2893601946,-1.7705768162,-3.8375500362
H,0,0.7260849297,-0.1484763065,-3.2642024263

Int-2′-*trans*
Opt @ B3LYP-D3(BJ)/def2-SVP in gas phase
SCF Done: E(RB3LYP) = -3088.05106094 a.u.
Zero-point correction = 0.620236 Hartree/Particle
Sum of electronic and thermal Free Energies = -3087.499691 a.u.
Sp @ RI-PWPB95-D3(BJ)/def2-TZVPP in 2,2,2-trifluoroethanol
FINAL SINGLE POINT ENERGY = -3088.772122758301 a.u.

Co,0,-0.5012445463,-1.1352696591,0.0898365526
C,0,-1.8387010141,-1.8593586499,-1.3877269255
C,0,-2.342846842,-2.2653087379,-0.1035627225
C,0,-1.3508936835,-3.0559760231,0.5425452081
C,0,-0.2428733593,-3.1890225602,-0.3722531671
C,0,-0.5576411604,-2.4807652391,-1.5722298724
C,0,2.0102116055,1.0286323448,-2.4993775857
C,0,2.8527171055,0.4263541718,-1.5281813583
C,0,4.2388400043,0.574339332,-1.5346435935
C,0,4.7880407439,1.3520343353,-2.5581274044
C,0,3.9757521434,1.9577317068,-3.5330571352
C,0,2.5884997668,1.8040951815,-3.5140010332
C,0,0.6376352079,0.6909607295,-2.1604814885
C,0,0.6461079632,-0.0802939598,-1.0348948028
H,0,4.8553117526,0.1055649195,-0.7705034783
H,0,5.8704320869,1.4902184636,-2.5996775334
H,0,4.4400754514,2.5585112949,-4.3179311313
H,0,1.9644555478,2.2810429371,-4.2728867078
H,0,-0.244289768,1.0115344272,-2.7110743612
N,0,1.9981403773,-0.2535635586,-0.6411528663
C,0,2.2414159411,-0.8418361301,0.5578960829
C,0,3.5980022988,-1.5621760054,2.2309953231
C,0,1.2414776462,-1.7628814416,2.4273618133
C,0,2.4922929254,-1.9455223937,3.0023000107

```
H,0,4.6183634043,-1.7023181897,2.6031207375
H,0,0.3221226123,-2.0373147221,2.945571883
H,0,2.6021884245,-2.3752647447,3.9974282544
N,0,3.4749006206,-1.0114346659,1.0286121478
N,0,1.1072906045,-1.2341403396,1.2061203997
C,0,2.8302086886,1.8956133861,2.5271990142
C,0,2.8821251176,2.4965266685,1.2664364137
C,0,1.7255016826,2.6289546926,0.4801875302
C,0,0.5379714472,2.1316191666,0.9914812668
C,0,0.4854007584,1.528210276,2.2583719169
C,0,1.6187314446,1.4059152515,3.0461369279
C,0,-0.9052215406,2.1606979166,0.5253303447
C,0,-1.477749391,0.7452540716,0.2527037476
C,0,-1.5184658659,0.137046476,1.4969739208
C,0,-0.985967421,1.2247614158,2.4710806459
O,0,-1.5669894193,2.3913299673,1.8091520316
H,0,3.7413715151,1.8203162353,3.1244423571
H,0,3.8327422182,2.8750881458,0.8867490177
H,0,1.7731625706,3.1045974935,-0.4998726458
H,0,1.5795399994,0.9631231113,4.0434954176
H,0,-1.1884930349,2.9204184087,-0.2088176833
H,0,-2.2011507534,0.6301166058,-0.5450966959
H,0,-2.27542338,-0.5565357024,1.8504218171
H,0,-1.3419200589,1.1411195699,3.5032315025
C,0,-2.6103625133,-1.0917500026,-2.4116697636
H,0,-3.1865438307,-1.7885638303,-3.0438168061
H,0,-3.3317871528,-0.3993648964,-1.9542704648
H,0,-1.9472949954,-0.5207868888,-3.0745555782
C,0,0.254454914,-2.507487649,-2.824185109
H,0,0.0891855618,-3.4690116808,-3.3396314407
H,0,-0.022888257,-1.6986225056,-3.5087467061
H,0,1.329712445,-2.4183688824,-2.6189261568
C,0,-3.7080585344,-1.950544023,0.4097141296
H,0,-3.986923534,-0.8983662973,0.2482126582
H,0,-4.4434400324,-2.564565001,-0.1375668561
H,0,-3.8153250939,-2.1901645188,1.4762221267
C,0,-1.5065962938,-3.7496667117,1.859391043
H,0,-1.9612026728,-3.0978445724,2.6205872311
H,0,-2.1655239205,-4.6274709008,1.7529602879
H,0,-0.5467787812,-4.1147187892,2.2472355479
C,0,0.9909840997,-4.004966974,-0.1665253219
H,0,0.8605238223,-4.9983663568,-0.6277370094
H,0,1.8675042313,-3.5410919113,-0.6404598253
H,0,1.2158975285,-4.1622440985,0.8961995917
C,0,-6.3632810093,2.3823422575,0.6277177656
H,0,-6.5333934463,3.4595156775,0.4772222813
H,0,-6.8235423337,2.1188939342,1.5923820428
H,0,-6.8265284724,1.8107301214,-0.1843490605
C,0,-4.8845096055,2.108314426,0.6775145085
O,0,-4.3024298416,1.3586036054,-0.0900837832
O,0,-4.2775567203,2.7707447636,1.6531221014
H,0,-3.303148119,2.5864679054,1.668186133
----------------------------------------------------------------
```

INT-2′-*cis*

Opt @ B3LYP-D3(BJ)/def2-SVP in gas phase

```
SCF Done: E(RB3LYP) = -3088.06201122 a.u.
Zero-point correction = 0.619979 Hartree/Particle
Sum of electronic and thermal Free Energies = -3087.511358 a.u.
Sp @ RI-PWPB95-D3(BJ)/def2-TZVPP in 2,2,2-trifluoroethanol
FINAL SINGLE POINT ENERGY = -3088.790404078929 a.u.
-----------------------------------------------------------------
Co,0,-0.9445568783,-1.2781601245,0.2884580285
C,0,-2.2364992259,-1.9517176282,1.8709591546
C,0,-1.3843960291,-3.0305948079,1.4638241304
C,0,-1.6562077803,-3.2882111115,0.0827784845
C,0,-2.6334534598,-2.3456051118,-0.3779931764
C,0,-3.0001220251,-1.5178068916,0.7412008755
C,0,-2.3773956659,-1.3949253347,3.2484670768
C,0,-1.0829246542,-4.4161604909,-0.7099324316
H,0,0.005318466,-4.5139061883,-0.5846898276
H,0,-1.3019991871,-4.3255930163,-1.7813929551
C,0,-2.070200335,2.3122648047,-1.6142822757
C,0,-1.4864152286,2.6861211107,-0.3764025376
C,0,-1.352963534,4.0144313169,0.0244146828
C,0,-1.8297586252,4.9914836236,-0.8536491621
C,0,-2.4165000363,4.6468127458,-2.0836637998
C,0,-2.5419330543,3.3128945903,-2.4747010401
C,0,-2.0223716424,0.8602284394,-1.7006131655
C,0,-1.4330021909,0.3776629374,-0.5686052851
H,0,-0.8961974588,4.2684681175,0.9783239641
H,0,-1.7430101076,6.0441387192,-0.5765316042
H,0,-2.7782750409,5.4378099935,-2.7440493984
H,0,-2.9961014835,3.0534216548,-3.4335465163
H,0,-2.3788973071,0.2712272519,-2.5421791461
N,0,-1.1116428391,1.4850282029,0.2537814737
C,0,-0.4101189638,1.254483036,1.3910664368
C,0,0.6475488245,1.896918232,3.2962677917
C,0,0.6206595718,-0.3834997599,2.6575132582
C,0,1.0317913932,0.5772009502,3.5662050486
H,0,0.933895821,2.7118379063,3.9687807535
H,0,0.8799699157,-1.4365666125,2.7732503907
H,0,1.6414575525,0.3183538683,4.4305666256
N,0,-0.061908409,2.2326010196,2.2250079117
N,0,-0.1153071726,-0.0604466428,1.5839579638
C,0,4.8891140659,-1.9786749189,-2.7168169118
C,0,4.1658859811,-1.7136318567,-3.8809357135
C,0,2.8666418318,-1.1711316536,-3.8209487095
C,0,2.3410035516,-0.9029405102,-2.5696439372
C,0,3.070391463,-1.1712817012,-1.3952393051
C,0,4.3424162964,-1.7125883555,-1.4455291665
C,0,1.0071335937,-0.3421206705,-2.0687743302
C,0,0.2896758825,-1.5449395742,-1.4342849396
C,0,1.0170187122,-1.8250222514,-0.2821130136
C,0,2.1302263478,-0.7575221008,-0.260233521
O,0,1.4422125318,0.3454596573,-0.8830049929
H,0,5.89721957,-2.3918268044,-2.7920017362
H,0,4.6159140589,-1.9229193855,-4.8536905183
H,0,2.3090274358,-0.9616737297,-4.7366275723
H,0,4.9165654737,-1.9166277933,-0.5390104322
H,0,0.4281991319,0.311936124,-2.7246602321
H,0,-0.241597795,-2.2639820372,-2.054172132
```

H,0,1.1801028295,-2.8095928816,0.1539946054
H,0,2.5714387249,-0.4402073571,0.6879140369
H,0,-2.4981447797,-0.3028426986,3.2373998139
H,0,-3.2800281599,-1.8172783433,3.7213075165
H,0,-1.5229633463,-1.6410030634,3.8907668183
H,0,-1.5288694153,-5.3636531105,-0.3635419679
C,0,-0.5008297515,-3.8637045828,2.3373976105
H,0,-0.9979849344,-4.8158059119,2.5881585906
H,0,0.4479196827,-4.1218862011,1.8431110342
H,0,-0.2670958471,-3.3643420981,3.2870767326
C,0,-4.0742637691,-0.4810330221,0.7803705123
H,0,-5.0013301379,-0.9202736295,1.1858831134
H,0,-3.7956613099,0.3634549102,1.4254318594
H,0,-4.287077624,-0.0777731275,-0.2160104305
C,0,-3.2755116389,-2.3452637231,-1.7270080342
H,0,-3.9896757885,-3.1819610596,-1.8037184803
H,0,-3.8287171246,-1.416634291,-1.9080724425
H,0,-2.5421912814,-2.4675736362,-2.5372430621
C,0,3.0816124521,4.1619326871,1.4177568551
H,0,3.590481318,4.7818118348,0.6657412437
H,0,2.1401726159,4.6731862064,1.6762800528
H,0,3.7031690156,4.0535700716,2.3140680835
C,0,2.7501250956,2.8087484327,0.8506230288
O,0,2.9546906702,1.7551169351,1.4268005256
O,0,2.1665531338,2.8828842782,-0.3412712271
H,0,1.9434736372,1.9667493133,-0.6376333946

TS-2′-*trans*
Opt @ B3LYP-D3(BJ)/def2-SVP in gas phase
SCF Done: E(RB3LYP) = -3088.04410893 a.u.
Imaginary frequency = -239.1592 cm^{-1}
Zero-point correction = 0.620105 Hartree/Particle
Sum of electronic and thermal Free Energies = -3087.491853 a.u.
Sp @ RI-PWPB95-D3(BJ)/def2-TZVPP in 2,2,2-trifluoroethanol
FINAL SINGLE POINT ENERGY = -3088.758982484106 a.u.

Co,0,-1.019143514,-0.0374309999,-0.398353981
C,0,-2.1590145113,-1.6207673191,-1.2070880502
C,0,-2.9713681761,-0.4422210144,-1.1896355793
C,0,-3.0483148067,0.0268461487,0.1555321989
C,0,-2.3499737026,-0.9303716737,0.9911583161
C,0,-1.812778063,-1.9447638342,0.1577789157
C,0,2.6094517131,-2.2244255281,-0.2360472873
C,0,2.5906647168,-1.3272108341,0.8647214023
C,0,3.5633206336,-1.3503685408,1.8657974998
C,0,4.5759756809,-2.3046846233,1.7427968398
C,0,4.6160731253,-3.2027618672,0.6595439605
C,0,3.6406960158,-3.1720534215,-0.3348396413
C,0,1.4764651698,-1.9079978471,-1.0724240621
C,0,0.7815000989,-0.8624104586,-0.5131264407
H,0,3.5278230854,-0.6501421141,2.6966967046
H,0,5.3560570919,-2.3512386971,2.505563945
H,0,5.4257172796,-3.9329287339,0.5996571902
H,0,3.6752597558,-3.8690036707,-1.174800484
H,0,1.2414176093,-2.3867015574,-2.0203565827

N,0,1.4661878339,-0.5050206632,0.6856901248
C,0,0.9603951687,0.4712909757,1.4906865097
C,0,1.0225611558,1.8298489108,3.3092266149
C,0,-0.7333522519,2.0205444797,1.7259217841
C,0,-0.1403894484,2.487731929,2.8916212485
H,0,1.5305708649,2.1165322848,4.2358128677
H,0,-1.6450546593,2.4607702821,1.3224211241
H,0,-0.5695490864,3.3165642508,3.4537356013
N,0,1.574524476,0.8431017535,2.6098070129
N,0,-0.2078381365,0.9988115325,1.0405244832
C,0,2.7416126949,3.4679896816,0.8051270725
C,0,3.6327030168,2.4403976194,0.4798230076
C,0,3.3546413489,1.5520792473,-0.5716042266
C,0,2.1681967027,1.7290672253,-1.2649830878
C,0,1.2647000299,2.7515194001,-0.9333815034
C,0,1.5383450281,3.6363130192,0.0989971366
C,0,1.5535841204,1.0615204302,-2.4730932564
C,0,0.2396614645,0.3125868541,-2.1220687313
C,0,-0.6991872653,1.352068242,-1.827708824
C,0,0.1557467554,2.6401857718,-1.9622674453
O,0,0.9564558548,2.220293315,-3.1230025853
H,0,2.9907549156,4.1582255282,1.6141405929
H,0,4.5627112522,2.334469792,1.0415874542
H,0,4.0540108602,0.7547924182,-0.828868272
H,0,0.8563417361,4.4513780828,0.3509811844
H,0,2.2181922073,0.5169328897,-3.1511452962
H,0,-0.0393028521,-0.4978659114,-2.7901007976
H,0,-1.6237417936,1.3886879741,-2.3982380228
H,0,-0.4024293577,3.5529404232,-2.1948168419
C,0,-1.8809899221,-2.4531382036,-2.4169613861
H,0,-2.7287837753,-3.1360225795,-2.5977750752
H,0,-1.7565605964,-1.8394338709,-3.3204113627
H,0,-0.9864953067,-3.0750492829,-2.2849378917
C,0,-1.0881592002,-3.1615375563,0.6315467739
H,0,-1.8125652161,-3.9029678624,1.0084969348
H,0,-0.5101350218,-3.6314819826,-0.1721773902
H,0,-0.3929778756,-2.9303904162,1.4508379821
C,0,-3.669018574,0.1389160161,-2.3739500693
H,0,-3.060604992,0.0659426981,-3.2866659479
H,0,-4.6002490316,-0.4251626103,-2.5540897845
H,0,-3.9486875251,1.1890139858,-2.2138889209
C,0,-3.8849064758,1.1763814396,0.6239303187
H,0,-3.7641081494,2.0615556482,-0.0178230728
H,0,-4.9530278144,0.9023905509,0.6009498903
H,0,-3.6526049472,1.4628802917,1.6579119217
C,0,-2.2461909688,-0.8997939641,2.4813161297
H,0,-3.0115123388,-1.560074917,2.9224629088
H,0,-1.2674697363,-1.2609140502,2.8287970958
H,0,-2.4064564323,0.1057355102,2.8910767213
C,0,-1.6197178509,0.6500129664,-7.0193136486
H,0,-0.8265930405,0.6037947882,-7.7810649969
H,0,-2.2264970512,1.5406012579,-7.2449194216
H,0,-2.2403681283,-0.2520412795,-7.0632032932
C,0,-1.0034113614,0.791575937,-5.6540753589
O,0,-1.2024806679,0.0173517065,-4.7304895314
O,0,-0.2208075857,1.8558942447,-5.5556293767

H,0,0.1751114071,1.935238582,-4.6482727117

TS-2′-*cis*
Opt @ B3LYP-D3(BJ)/def2-SVP in gas phase
SCF Done: E(RB3LYP) = -3088.05432535 a.u.
Imaginary frequency = -254.7658 cm^{-1}
Zero-point correction = 0.619703 Hartree/Particle
Sum of electronic and thermal Free Energies = -3087.503942 a.u.
Sp @ RI-PWPB95-D3(BJ)/def2-TZVPP in 2,2,2-trifluoroethanol
FINAL SINGLE POINT ENERGY = -3088.77386587615 a.u.

Co,0,0.0249630507,1.2397941157,-0.2657725964
C,0,1.0581413226,3.0865989113,-0.0353690932
C,0,-0.3700973959,3.2956610228,-0.1186076547
C,0,-0.7906073329,2.8382618395,-1.4079026885
C,0,0.3401774302,2.2763773144,-2.0761211279
C,0,1.4960071444,2.4506141616,-1.2295542101
C,0,1.9451795072,3.4955022372,1.0944775784
C,0,-2.1493145851,2.9950860149,-2.0069120787
H,0,-2.9218302762,3.1739247982,-1.2473526697
H,0,-2.4435820745,2.1208428759,-2.6058931675
C,0,2.510970257,-1.9660454173,-1.4516393344
C,0,2.598850986,-1.8708092445,-0.0357838368
C,0,3.5038065957,-2.6263353799,0.71160378
C,0,4.3326818374,-3.5005408632,0.0051235389
C,0,4.2608343379,-3.6160743021,-1.3965890774
C,0,3.3565867916,-2.8565523606,-2.1344704719
C,0,1.4824827884,-1.0526678767,-1.8848615011
C,0,0.9648929846,-0.410980319,-0.782189201
H,0,3.5482050807,-2.5359667181,1.7944818959
H,0,5.0503306001,-4.1120054137,0.5560914075
H,0,4.9255710116,-4.3137403982,-1.9101192771
H,0,3.3045800259,-2.9478641743,-3.2214713309
H,0,1.1298563321,-0.942718943,-2.9072539582
N,0,1.6481578282,-0.9123752038,0.3543926874
C,0,1.4089451064,-0.3533743327,1.5784140377
C,0,1.6161327935,-0.2732988175,3.8360618617
C,0,0.182077194,1.2393064742,2.7051680318
C,0,0.7021938837,0.7796651026,3.9095981848
H,0,2.0797636715,-0.6845683983,4.7384063514
H,0,-0.5534633997,2.0422672278,2.6671672584
H,0,0.3824366388,1.208666404,4.8576240827
N,0,1.9626579568,-0.8380624176,2.679494373
N,0,0.5552737012,0.7041088827,1.5381269824
C,0,-5.4776682028,-2.2720425419,0.0198894273
C,0,-4.80967237,-3.2222549192,-0.7563032224
C,0,-3.4063921157,-3.2095485603,-0.8671610579
C,0,-2.7165179789,-2.2324114857,-0.1691608563
C,0,-3.3889798175,-1.2724191637,0.6119131255
C,0,-4.7698766516,-1.2720355711,0.7139426667
C,0,-1.2424770909,-1.8687181439,-0.0333404312
C,0,-1.0809612916,-0.5636945627,-0.8484751011
C,0,-1.7991856635,0.4136231069,-0.0816441253
C,0,-2.2827185347,-0.3687318797,1.1514544187
O,0,-1.2144840515,-1.3389071252,1.3043103791

H,0,-6.5665960755,-2.3094099165,0.0951011868
H,0,-5.3833724684,-3.9906774685,-1.2786867258
H,0,-2.8891344295,-3.9568814572,-1.47317582
H,0,-5.2993205028,-0.5338916028,1.3204197149
H,0,-0.4920472735,-2.6518424166,-0.1762532213
H,0,-1.1653070826,-0.6153269524,-1.9338745048
H,0,-2.5281886728,1.0637744534,-0.5603880728
H,0,-2.4575694514,0.1461134629,2.0999861948
H,0,2.7407822594,2.7599279833,1.277538617
H,0,2.4335182511,4.4537963866,0.8506348227
H,0,1.3908270647,3.6409802456,2.0300730152
H,0,-2.1506285605,3.8640596757,-2.686693048
C,0,-1.2297586219,4.0257503512,0.8661744858
H,0,-1.4200208166,5.0562855486,0.5226704274
H,0,-2.2064353003,3.5380335866,0.9988380381
H,0,-0.7517844237,4.1034937872,1.8517170306
C,0,2.9056127491,2.0929133478,-1.5688408336
H,0,3.3937930874,2.9248584496,-2.1034552773
H,0,3.4976935581,1.8854874795,-0.6668973997
H,0,2.9535037946,1.2023917657,-2.2085999696
C,0,0.3347804864,1.7613032567,-3.4795570492
H,0,0.2352607793,2.6025736016,-4.1859384524
H,0,1.2634201895,1.2321171672,-3.7229383256
H,0,-0.5110386174,1.0835464958,-3.6695877006
C,0,-1.6896019103,-1.8535260643,6.0832067878
H,0,-2.2248940005,-2.8095047968,6.1878851306
H,0,-0.6568488016,-2.028745329,6.423234278
H,0,-2.166857936,-1.0836938783,6.6999974869
C,0,-1.683390746,-1.4290715295,4.6393592418
O,0,-2.1275880871,-0.3713706601,4.2346095283
O,0,-1.1139951202,-2.336637236,3.8467838041
H,0,-1.1592062461,-2.037189113,2.9061715701

INT-3′-*trans*
Opt @ B3LYP-D3(BJ)/def2-SVP in gas phase
SCF Done: E(RB3LYP) = -3088.06982313 a.u.
Zero-point correction = 0.621678 Hartree/Particle
Sum of electronic and thermal Free Energies = -3087.517743 a.u.
Sp @ RI-PWPB95-D3(BJ)/def2-TZVPP in 2,2,2-trifluoroethanol
FINAL SINGLE POINT ENERGY = -3088.776261338239 a.u.

Co,0,-0.4155498378,-0.8330356983,0.8519895717
C,0,-1.6077039871,-2.4530096517,0.273954712
C,0,-2.1314168026,-1.86454704,1.4547896852
C,0,-1.0927722003,-1.8905423299,2.4514954299
C,0,0.0344036361,-2.6301204662,1.9014299173
C,0,-0.2657748971,-2.9477198229,0.5635734956
C,0,1.7719312198,-1.2631232263,-2.7744690219
C,0,2.5896391396,-0.7164935962,-1.74982494
C,0,3.9709493603,-0.9415213699,-1.7169242228
C,0,4.5154616842,-1.717863214,-2.7386190588
C,0,3.7194552135,-2.2603042563,-3.7693924748
C,0,2.3492623826,-2.038046389,-3.7978013591
C,0,0.4283470439,-0.8668133961,-2.4955603057
C,0,0.3940672198,-0.1233696649,-1.3333362491

```
H,0,4.5944690072,-0.5023485342,-0.9427668811
H,0,5.5926856983,-1.8974948896,-2.746866466
H,0,4.1910692878,-2.855596818,-4.5536294062
H,0,1.7269670042,-2.4538592473,-4.5928839771
H,0,-0.4393688724,-1.0426731766,-3.1283363779
N,0,1.746116537,-0.0244509284,-0.8572130872
C,0,2.1174770414,0.2910071484,0.4422735139
C,0,3.6958201025,0.9549368344,1.9427439872
C,0,1.5122516803,0.3963045627,2.6661259242
C,0,2.7859123132,0.8336712962,2.9963764431
H,0,4.7267393377,1.2789361995,2.1172342928
H,0,0.7276165224,0.2794563532,3.4126378735
H,0,3.055249527,1.0719769726,4.0250089553
N,0,3.3546533543,0.7043994933,0.6813028447
N,0,1.1863074715,0.0863375722,1.4016770436
C,0,2.2522168624,3.9392732003,1.3228119369
C,0,2.6415850493,3.897420334,-0.0188332221
C,0,1.7937175743,3.3516431926,-0.9965866141
C,0,0.5630626763,2.863660672,-0.5856869843
C,0,0.1771925606,2.8802885838,0.7673309936
C,0,1.0116608656,3.4213328617,1.7340487062
C,0,-0.6355473954,2.3032071746,-1.3097943991
C,0,-0.7972140251,0.7687652348,-0.9970724195
C,0,-1.3392820568,0.809914247,0.4532398236
C,0,-1.2300362874,2.3166244469,0.7871164899
O,0,-1.7070845653,2.8401670624,-0.5021801365
H,0,2.917553682,4.3914281196,2.0619515824
H,0,3.6086691335,4.3099353067,-0.3133605026
H,0,2.0924064076,3.3348486443,-2.0467797401
H,0,0.713424424,3.4671458927,2.784166914
H,0,-0.7633002589,2.568585018,-2.3652624016
H,0,-1.6055486542,0.3834573091,-1.629442993
H,0,-2.4086359638,0.588077251,0.4375970772
H,0,-1.8839837427,2.6646482547,1.5946224428
C,0,-2.3551463259,-2.6292408671,-1.0084186709
H,0,-3.0114921925,-3.5132416872,-0.9324379557
H,0,-2.9911069768,-1.7623349437,-1.2362516897
H,0,-1.675232271,-2.7986268316,-1.8525374308
C,0,0.6284745795,-3.6761199352,-0.3875356507
H,0,0.5744862503,-4.7630384835,-0.2078152822
H,0,0.3455162482,-3.4958594963,-1.4310994758
H,0,1.6804867255,-3.3755029452,-0.2742655893
C,0,-3.5171932052,-1.3419384155,1.6390572645
H,0,-3.9348843107,-0.9590250514,0.6981505757
H,0,-4.173111985,-2.1561515324,1.9916072513
H,0,-3.5546451444,-0.5396984834,2.3887663223
C,0,-1.2577734374,-1.4437629147,3.8688629643
H,0,-1.7455507949,-0.4600282199,3.9323516848
H,0,-1.8955322865,-2.1581302714,4.4169718171
H,0,-0.3007812558,-1.3966613097,4.405730903
C,0,1.3019171565,-2.9645027165,2.6180652873
H,0,1.2259248913,-3.9653772227,3.0750957704
H,0,2.1634536666,-2.9803468785,1.9353568489
H,0,1.5218468257,-2.2566191107,3.4286596781
C,0,-6.0677481891,1.198997687,-2.0383551097
H,0,-6.2111530348,1.8208974213,-2.9349783139
```

H,0,-6.7358426251,1.6015380527,-1.2611595791
H,0,-6.3250235367,0.1558847677,-2.25415869
C,0,-4.6397533949,1.2980073461,-1.5742687839
O,0,-3.9032169685,0.3344547867,-1.423915231
O,0,-4.2639630877,2.5446154357,-1.3372957761
H,0,-3.3204078843,2.5875066465,-1.025037965

INT-3′-*cis*
Opt @ B3LYP-D3(BJ)/def2-SVP in gas phase
SCF Done: E(RB3LYP) = -3088.07791076 a.u.
Zero-point correction = 0.621335 Hartree/Particle
Sum of electronic and thermal Free Energies = -3087.526859 a.u.
Sp @ RI-PWPB95-D3(BJ)/def2-TZVPP in 2,2,2-trifluoroethanol
FINAL SINGLE POINT ENERGY = -3088.78590348142 a.u.

Co,0,-1.0078986212,-1.098497213,0.1929064124
C,0,-2.4286435322,-1.7949398081,1.6346612001
C,0,-1.2776056118,-2.6712119059,1.4737645296
C,0,-1.2852748189,-3.1645952468,0.1261149144
C,0,-2.3394221898,-2.5078785603,-0.568694383
C,0,-3.072534489,-1.6877786807,0.3879629442
C,0,-2.8406224576,-1.1135421227,2.8991327799
C,0,-0.3773732771,-4.2159611455,-0.4195495264
H,0,0.6255062748,-4.1650315622,0.0264666973
H,0,-0.2730491735,-4.1465494265,-1.5108296715
C,0,-3.1154557844,1.7888967416,-1.6143168734
C,0,-2.4814253761,2.3327217945,-0.4625811728
C,0,-3.0794674347,3.3377655728,0.3075375284
C,0,-4.3208838401,3.80400289,-0.1158066139
C,0,-4.9604253756,3.2902192203,-1.2679537601
C,0,-4.3697820349,2.2881431525,-2.0226397682
C,0,-2.26176363,0.7643335014,-2.1147397814
C,0,-1.150591696,0.6491655161,-1.291745269
H,0,-2.5750637064,3.7537041163,1.1776340419
H,0,-4.8083092658,4.6001630833,0.4510437577
H,0,-5.9298314525,3.6950689518,-1.5646456708
H,0,-4.8616312388,1.8883417589,-2.9117813801
H,0,-2.397179108,0.2068067657,-3.03942485
N,0,-1.2809832031,1.6426966777,-0.2739052017
C,0,-0.6137406093,1.5638900847,0.9482528679
C,0,0.3032489291,2.5356317173,2.7786043587
C,0,0.4203970087,0.1845195905,2.4649696531
C,0,0.7446393895,1.2968233951,3.2362975988
H,0,0.4989571245,3.4548839324,3.3391171332
H,0,0.7477246688,-0.8146545582,2.7422113827
H,0,1.3351031583,1.1876383679,4.1446907096
N,0,-0.3615949143,2.6660974788,1.6250450816
N,0,-0.3031422655,0.3097516811,1.3453209577
C,0,4.5688850964,-1.424573256,-3.15797186
C,0,4.1817412237,-0.3729737452,-3.9946705553
C,0,3.1519381652,0.5068849658,-3.619172466
C,0,2.5498992923,0.3074795578,-2.3857628518
C,0,2.9384948715,-0.749948144,-1.5425247252
C,0,3.9404621077,-1.6330399144,-1.9180836195
C,0,1.4122843789,0.9840436993,-1.6502427257

C,0,0.1771224668,0.0273977623,-1.7460739781
C,0,0.6268330691,-1.1514361505,-0.8558328972
C,0,2.0007090835,-0.6677652141,-0.3497960878
O,0,1.8004871705,0.7749615213,-0.2749715074
H,0,5.3776740928,-2.0891045573,-3.4698035655
H,0,4.6918752412,-0.2287953608,-4.9494508635
H,0,2.8524167062,1.3253701846,-4.2779147664
H,0,4.247088823,-2.4580128698,-1.2708594773
H,0,1.2179315499,2.044490246,-1.846369505
H,0,0.0096837842,-0.2871594132,-2.7835120637
H,0,0.7175272556,-2.0959831559,-1.4008239537
H,0,2.369190438,-1.0350380582,0.6126908672
H,0,-3.3772054834,-0.1757978457,2.6970752397
H,0,-3.5155544428,-1.7620781393,3.4825677881
H,0,-1.9802586681,-0.8807202533,3.5422587349
H,0,-0.7899813579,-5.2139655393,-0.1936217039
C,0,-0.3764517742,-3.1667115582,2.5595828892
H,0,-0.6652006979,-4.1912914485,2.8498505641
H,0,0.6743772887,-3.2072623469,2.2359779395
H,0,-0.4386463063,-2.5491325537,3.465039349
C,0,-4.3049116526,-0.8874961106,0.1106350913
H,0,-5.1885750149,-1.3954192651,0.5316919448
H,0,-4.2568862532,0.1152616318,0.5603056672
H,0,-4.4766486531,-0.7564844162,-0.9638051667
C,0,-2.7069809009,-2.7457631186,-1.9998136405
H,0,-3.2171830288,-3.7175007676,-2.1082356156
H,0,-3.3860611535,-1.9729775253,-2.3808608167
H,0,-1.8192677874,-2.7702434496,-2.6490696965
C,0,5.051062681,1.4615078522,3.204683542
H,0,5.9901669463,1.5654445754,2.6394111828
H,0,4.8055883139,2.4592974118,3.5996834848
H,0,5.1853305726,0.746720239,4.0244674575
C,0,3.9585710804,0.9925233254,2.2813877123
O,0,3.353749175,-0.0569449,2.4178973223
O,0,3.7154371226,1.8559712679,1.3008748818
H,0,3.0221714608,1.4966080061,0.6897246434

INT-4′-*trans*
Opt @ B3LYP-D3(BJ)/def2-SVP in gas phase
SCF Done: E(RB3LYP) = -3316.61338035 a.u.
Zero-point correction = 0.672223 Hartree/Particle
Sum of electronic and thermal Free Energies = -3316.017342 a.u.
Sp @ RI-PWPB95-D3(BJ)/def2-TZVPP in 2,2,2-trifluoroethanol
FINAL SINGLE POINT ENERGY = -3317.381095537188 a.u.

Co,0,1.2565915082,0.4453093056,0.9481815328
C,0,0.3373830114,-0.6596071816,2.5312137789
C,0,0.1812944968,0.7340281182,2.7571865671
C,0,1.4924007475,1.3202726347,2.7838567551
C,0,2.4546093941,0.2362320981,2.720613722
C,0,1.7559409068,-0.9707737843,2.530816537
C,0,0.9823077078,-2.3962826589,-0.8016357169
C,0,-0.3221289672,-2.9262075596,-0.6513075684
C,0,-0.5517429242,-4.3029665457,-0.5721630176
C,0,0.5617508503,-5.1416709256,-0.663055736

C,0,1.8620878957,-4.6276450982,-0.8244687277
C,0,2.0859417607,-3.2541005034,-0.8933180178
C,0,0.8578981256,-0.9571050574,-0.8368974978
C,0,-0.4945899543,-0.6456945767,-0.744911381
H,0,-1.5540463408,-4.6988995687,-0.4297923175
H,0,0.4147441062,-6.222733542,-0.6029477741
H,0,2.7067806029,-5.3178559158,-0.8867503661
H,0,3.0835700419,-2.824919378,-0.9918851979
H,0,1.6205566116,-0.2757911454,-1.19381478
N,0,-1.2204883665,-1.829891873,-0.6340256583
C,0,-2.6149416763,-1.9036049487,-0.4959037651
C,0,-4.4371335245,-2.9688572395,0.3358970664
C,0,-4.6346043058,-0.914764586,-0.8293341181
C,0,-5.2651890537,-1.946429631,-0.1300796161
H,0,-4.8485631347,-3.8318039821,0.8713479605
H,0,-5.1956304338,-0.0663067502,-1.2329849473
H,0,-6.3432079644,-1.957810154,0.0340238177
N,0,-3.1137250268,-2.9446308697,0.175078104
N,0,-3.3209212237,-0.9126579489,-1.0434093108
C,0,2.3590211295,1.0056563136,-4.0314321144
C,0,1.2976946148,0.2670171242,-4.5651143999
C,0,0.0125703385,0.3511617317,-4.0055581543
C,0,-0.1694267276,1.1933428294,-2.9184687878
C,0,0.9008139095,1.9157224461,-2.3642486367
C,0,2.1709986214,1.8378352663,-2.916154326
C,0,-1.3329261289,1.4794040436,-2.0027517006
C,0,-1.0572145478,0.7359721367,-0.6430767894
C,0,0.007522722,1.6495534721,0.014380687
C,0,0.332110908,2.6147470899,-1.149011261
O,0,-1.0477579506,2.8489072628,-1.626556072
H,0,3.3493318036,0.9317179972,-4.487388397
H,0,1.4704615805,-0.3794125339,-5.4288048621
H,0,-0.8144942267,-0.2339567974,-4.4146328929
H,0,3.0028909002,2.3968439183,-2.4896257778
H,0,-2.3519914876,1.3739111958,-2.3834474163
H,0,-1.9914539843,0.7346797168,-0.0771501541
H,0,-0.4711730649,2.2812594235,0.7680089497
H,0,0.7971380737,3.5631028302,-0.8604742634
C,0,-0.7724554237,-1.6597566588,2.5050182001
H,0,-0.9479165369,-2.050715799,3.5227145697
H,0,-1.7155040214,-1.2153190118,2.1610284539
H,0,-0.5473619185,-2.519398601,1.8625058406
C,0,2.3686493678,-2.3258032326,2.3748907217
H,0,2.757077316,-2.696202861,3.3391955956
H,0,1.6415924398,-3.0576766797,2.0009313276
H,0,3.1970218586,-2.2849102201,1.6542639872
C,0,-1.1207552456,1.4386338629,2.9734625318
H,0,-1.9103619953,1.0753166114,2.3005524358
H,0,-1.4673153674,1.2750484929,4.0083684937
H,0,-1.0290704659,2.5232609174,2.8262970468
C,0,1.831614238,2.7643386222,2.9665148332
H,0,0.9892423253,3.4119630209,2.6850984992
H,0,2.0958954137,2.9904485234,4.014270195
H,0,2.6869970295,3.0414078451,2.3334077095
C,0,3.9346181812,0.396775408,2.8299373144
H,0,4.2340837162,0.4080984288,3.8925639712

H,0,4.4636945721,-0.4207274645,2.3261144482
H,0,4.2660457852,1.3440507296,2.3833801055
C,0,-4.2657048401,3.8033248125,1.8722760165
H,0,-5.1539647721,3.2111680568,2.1221147636
H,0,-4.5440018683,4.839241833,1.6298795057
H,0,-3.5944414503,3.8426730147,2.745365054
C,0,-3.5301358703,3.1688193012,0.7153928822
O,0,-3.7840440759,2.059513028,0.2848203777
O,0,-2.5643705129,3.9474929799,0.2476744836
H,0,-2.0577815119,3.4963249321,-0.4950696035
C,0,4.9696378134,1.3159911921,-0.8266973013
H,0,5.8753728846,1.0376619904,-0.2669264069
H,0,4.8134705362,2.4010700427,-0.7690780406
H,0,5.1343372411,1.0204413917,-1.8745197284
C,0,3.7866897897,0.5306296388,-0.2738350256
O,0,3.8577134742,-0.694025574,-0.1747255769
O,0,2.76499497,1.2575995374,0.0548833412

TS-3′-rac-*cis* [named as TS-*rac-cis* in the main text]
Opt @ B3LYP-D3(BJ)/def2-SVP in gas phase
SCF Done: E(RB3LYP) = -3088.04402302 a.u.
Imaginary frequency = -443.2730 cm^{-1}
Zero-point correction = 0.618593 Hartree/Particle
Sum of electronic and thermal Free Energies = -3087.495044 a.u.
Sp @ RI-PWPB95-D3(BJ)/def2-TZVPP in 2,2,2-trifluoroethanol
FINAL SINGLE POINT ENERGY = -3088.750387788606 a.u.

Co,0,0.3576136014,-1.0118652265,0.3900695866
C,0,0.5667133493,-3.0755332836,0.864087011
C,0,-0.7754338564,-2.8370509781,0.4793003575
C,0,-1.310481758,-1.8267740943,1.3709485838
C,0,-0.2970371439,-1.4586683424,2.3012841814
C,0,0.882913444,-2.223759522,1.986465308
C,0,1.5003398902,-4.0359261053,0.2044595332
C,0,-2.7206549516,-1.3422490993,1.4226271946
C,0,-0.4668204544,-0.5055602657,3.4404421233
H,0,-3.2658518255,-1.5245876077,0.490835388
H,0,-2.7767776838,-0.2694183278,1.6499808786
H,0,0.4979476169,-0.1176208969,3.7939159021
H,0,-0.9536203424,-1.0088400736,4.2929930491
C,0,-2.3515381537,-0.1983456079,-1.6134934082
C,0,-2.7790773298,0.9281737225,-0.8688905776
C,0,-4.1390895415,1.2381913941,-0.7446629363
C,0,-5.0515130361,0.4050003431,-1.3913068784
C,0,-4.6375964935,-0.6998204831,-2.1599395743
C,0,-3.2866669872,-1.0070439909,-2.2790292332
C,0,-0.9179246728,-0.2542506936,-1.5240087189
C,0,-0.4936514306,0.8313653225,-0.7542118656
H,0,-4.4663030236,2.1078750282,-0.1837935202
H,0,-6.1170159732,0.6310624995,-1.3120324333
H,0,-5.3836432026,-1.3118665179,-2.6703981337
H,0,-2.954624329,-1.8544333662,-2.8814875875
H,0,-0.2832051585,-0.8884313027,-2.1348674644
N,0,-1.6284487063,1.5455778939,-0.328621874
C,0,-1.6270028271,2.6888887517,0.4926359038

C,0,-2.6985347038,4.5596716581,1.2105707368
C,0,-0.5490641256,3.9869101692,2.0170728632
C,0,-1.6175665648,4.8827810601,2.0334116464
H,0,-3.5888637402,5.1959160794,1.1660755242
H,0,0.3384761081,4.1500590047,2.6377930354
H,0,-1.6126196624,5.7773199305,2.6566680825
N,0,-2.7022227185,3.4739526732,0.4371262966
N,0,-0.5553788853,2.8864738042,1.2616468281
C,0,5.3783551828,1.8483186998,-2.1054995122
C,0,5.8777484072,1.5400927223,-0.8346026907
C,0,5.060135212,0.9331188614,0.1269715798
C,0,3.7307904657,0.6768769928,-0.2100233704
C,0,3.23711972,0.9710415042,-1.490875079
C,0,4.0489204219,1.5535393388,-2.4511795464
C,0,2.7007384776,0.0617111946,0.6333556224
C,0,1.3788650863,0.665259561,0.763032975
C,0,0.9408148293,1.2723057523,-0.5739013088
C,0,1.8282942156,0.4531027752,-1.5566015718
O,0,1.8835320371,-0.8529142033,-0.9165571244
H,0,6.0392218043,2.3010651046,-2.8477672049
H,0,6.9209030823,1.7582375975,-0.5969248045
H,0,5.4513877477,0.6671991821,1.1106604988
H,0,3.6775590617,1.7579435902,-3.4577316445
H,0,3.0529233118,-0.6343173978,1.3936593378
H,0,1.1130117167,1.1774383446,1.6887177475
H,0,1.0730945721,2.3570428435,-0.696149552
H,0,1.4243031971,0.3819562217,-2.5735739218
H,0,-3.2502285394,-1.873223261,2.2324351396
H,0,-1.0927907765,0.3524539197,3.1582617574
H,0,1.4445010395,-3.9646713155,-0.8916827392
H,0,2.5387104186,-3.8590800245,0.5070148588
H,0,1.2295096428,-5.0692706828,0.4796149397
C,0,-1.5159701711,-3.5783981998,-0.585523529
H,0,-1.8070164792,-4.5784646262,-0.2218506907
H,0,-2.4325845194,-3.0566298804,-0.8838991077
H,0,-0.8955075214,-3.7293408436,-1.4817042493
C,0,2.1264199348,-2.296826124,2.8119353313
H,0,2.3107030003,-1.3613657558,3.3592493025
H,0,2.0129153472,-3.0913238748,3.5694138604
H,0,3.0128142625,-2.5357466278,2.2094960421
C,0,6.0935748632,-3.1483712191,-1.1607047858
H,0,5.9358464053,-3.993632046,-1.8466897076
H,0,6.6483470983,-2.3782116092,-1.7198753982
H,0,6.6752523941,-3.4632738518,-0.286971213
C,0,4.7716935539,-2.5784069367,-0.7244579011
O,0,4.4704586589,-2.3624443586,0.4367156733
O,0,3.9605492011,-2.3195322113,-1.7449264971
H,0,3.1417154395,-1.8604258753,-1.4153619246

TS-3′-*rac-trans* [named as TS-*rac-trans* in the main text]
Opt @ B3LYP-D3(BJ)/def2-SVP in gas phase
SCF Done: E(RB3LYP) = -3316.58070421 a.u.
Imaginary frequency = -390.0211 cm^{-1}
Zero-point correction = 0.667040 Hartree/Particle
Sum of electronic and thermal Free Energies = -3315.988636 a.u.

```
Sp @ RI-PWPB95-D3(BJ)/def2-TZVPP in 2,2,2-trifluoroethanol
FINAL SINGLE POINT ENERGY = -3317.349558764568 a.u.
----------------------------------------------------------------
Co,0,-0.4848636737,-0.8796957787,1.2672245443
C,0,-1.3304447759,-2.6783700327,0.5000460585
C,0,-2.3449208672,-1.9220024967,1.1453806595
C,0,-1.9207901395,-1.6802717854,2.4975171399
C,0,-0.7001177768,-2.4247573882,2.7100342693
C,0,-0.3107013326,-3.004019439,1.4797164153
C,0,2.0372245792,-1.436157517,-0.8137044068
C,0,1.5155150972,-1.7959056744,-2.0794011651
C,0,2.1273742738,-2.7597673173,-2.8851083914
C,0,3.2983332586,-3.3458475651,-2.3987797842
C,0,3.8401383076,-2.9832976083,-1.1514783208
C,0,3.2168980465,-2.030541521,-0.3475659023
C,0,1.1512853542,-0.4349708003,-0.2561212429
C,0,0.160345988,-0.1767307395,-1.2090441655
H,0,1.6986794908,-3.0478578832,-3.8421582463
H,0,3.8014288521,-4.1038073265,-3.0038748491
H,0,4.7577336416,-3.4653953218,-0.8061226104
H,0,3.59956867,-1.7530623512,0.6348689013
H,0,1.4214852998,0.2591680767,0.5298799635
N,0,0.3693772888,-0.9960999972,-2.3057973322
C,0,-0.4702345566,-1.0483560961,-3.4377519455
C,0,-1.4736180135,-2.2737301137,-5.0571349484
C,0,-1.9500042813,0.0310620116,-4.774323183
C,0,-2.2072767022,-1.1577114758,-5.4597278474
H,0,-1.5864414464,-3.2398608238,-5.5611184808
H,0,-2.4704121835,0.9603590199,-5.0230799932
H,0,-2.9349906675,-1.2089424435,-6.2701535214
N,0,-0.6186684216,-2.2317812123,-4.0333547972
N,0,-1.0500705871,0.0942250443,-3.7951832972
C,0,2.5266339574,3.4551940431,1.0097851434
C,0,2.7272764846,3.4917138604,-0.3725448774
C,0,1.7028243721,3.1119797864,-1.2557903783
C,0,0.4936644704,2.684444134,-0.7314124894
C,0,0.2990792053,2.6206743253,0.6632185228
C,0,1.3021807236,3.0252297958,1.5400130796
C,0,-0.7810916225,2.2366066822,-1.3995157407
C,0,-1.0046452256,0.7334514525,-1.0199580469
C,0,-1.3928534114,0.7735346383,0.4706429711
C,0,-1.0096999765,2.0408632595,1.0178930179
O,0,-1.7780230774,2.9584621908,-0.6988337233
H,0,3.3281280304,3.7668904791,1.6834658448
H,0,3.6851244074,3.8343653023,-0.7710415858
H,0,1.8529942977,3.1613106653,-2.3366753482
H,0,1.1466201101,2.9755142776,2.6175519358
H,0,-0.8278395428,2.3859491558,-2.4859685762
H,0,-1.8778516036,0.391347821,-1.5945385471
H,0,-2.4599585605,0.6126897925,0.6265884285
H,0,-1.5431477077,2.4360767728,1.8878775468
C,0,-1.410292658,-3.2057896923,-0.8941016611
H,0,-1.8952214635,-4.1977657477,-0.8876160621
H,0,-2.0120322235,-2.5541567842,-1.5403602615
H,0,-0.4235814897,-3.3312837925,-1.3544095298
C,0,0.9005388709,-3.8487075989,1.257877399
```

```
H,0,0.7648926867,-4.844809405,1.7131842804
H,0,1.1070471512,-3.9893017744,0.1898861591
H,0,1.7808796984,-3.372789449,1.7101690084
C,0,-3.6413838825,-1.5107781757,0.5275894706
H,0,-3.5382426343,-1.1972090085,-0.5207269313
H,0,-4.3382499714,-2.3665038695,0.5520245448
H,0,-4.1183011488,-0.6880840027,1.0772127502
C,0,-2.6485004523,-0.897046496,3.5413566317
H,0,-3.3496921611,-0.182379935,3.0882213602
H,0,-3.2272581399,-1.5571667909,4.2102856645
H,0,-1.9399505442,-0.3260303373,4.1586168896
C,0,0.0066708994,-2.5611325564,4.0164982705
H,0,-0.4438237446,-3.3877119728,4.5933734636
H,0,1.0731514254,-2.7700983262,3.8766103856
H,0,-0.0883713747,-1.6476766723,4.6188026775
C,0,-5.9662699723,0.9264021281,-1.6058645419
H,0,-6.2777216757,0.3000320624,-2.4524415204
H,0,-6.5131140724,1.8800461962,-1.6014832718
H,0,-6.2188360462,0.399433163,-0.6700027754
C,0,-4.4593550647,1.1643318496,-1.6380848708
O,0,-3.7390347949,0.3883425425,-2.2898619484
O,0,-4.0602886236,2.1496549946,-0.9236206107
H,0,-2.8220547286,2.5407745989,-0.8694286402
C,0,2.2197368693,0.7367051718,4.0842220258
H,0,2.73986796,0.1070672293,4.8203176156
H,0,1.4418481909,1.3356529517,4.5758708281
H,0,2.9697857504,1.412822346,3.6439372301
C,0,1.6539481497,-0.1450205831,2.9785251826
O,0,2.3290938627,-1.0616042169,2.5148481289
O,0,0.4564609781,0.1785772849,2.5960117907
-----------------------------------------------------------------
```

INT-5′-*trans*

```
Opt @ B3LYP-D3(BJ)/def2-SVP in gas phase
SCF Done: E(RB3LYP) = -3316.61464545 a.u.
Zero-point correction = 0.671275 Hartree/Particle
Sum of electronic and thermal Free Energies = -3316.018219 a.u.
Sp @ RI-PWPB95-D3(BJ)/def2-TZVPP in 2,2,2-trifluoroethanol
FINAL SINGLE POINT ENERGY = -3317.393724954175 a.u.
-----------------------------------------------------------------
Co,0,1.2948897109,0.511668283,0.8733423804
C,0,0.2076603669,-0.2583204479,2.5540042477
C,0,0.2815327289,1.1532989045,2.7031523096
C,0,1.6657360047,1.5429672642,2.6411777734
C,0,2.4511539816,0.3425540097,2.6102054233
C,0,1.5621840864,-0.7696309131,2.5052727428
C,0,0.8179715911,-2.3682238214,-0.5400279968
C,0,-0.5142529071,-2.8017938891,-0.348181399
C,0,-0.846702042,-4.1349048141,-0.1053752913
C,0,0.2025996384,-5.0568202681,-0.0960004723
C,0,1.52963419,-4.6534480454,-0.3292232733
C,0,1.8511030572,-3.3137310185,-0.5491025657
C,0,0.7753963501,-0.9205617818,-0.7257775693
C,0,-0.595929293,-0.5665911474,-0.7513786878
H,0,-1.8776559156,-4.4345243542,0.0748192712
H,0,-0.016487809,-6.1109359777,0.0891753592
```

```
H,0,2.3242961662,-5.4031552289,-0.3270441382
H,0,2.8780738982,-2.9825424508,-0.6988540613
H,0,1.5023564563,-0.3718858608,-1.3179637191
N,0,-1.3523965647,-1.6740154806,-0.4984142251
C,0,-2.7742130834,-1.6837646316,-0.4286961875
C,0,-4.649914437,-2.279038802,0.6704050374
C,0,-4.7249645465,-0.8934870439,-1.2572815598
C,0,-5.4127288513,-1.5151867479,-0.2138355152
H,0,-5.1181359595,-2.8332276291,1.4907966376
H,0,-5.2384745785,-0.2857153938,-2.0062295252
H,0,-6.4931077959,-1.418394644,-0.102177769
N,0,-3.3220678877,-2.3668928663,0.5706816065
N,0,-3.4022224524,-1.0073971989,-1.3800814165
C,0,2.5208936406,2.2496346958,-4.1006476651
C,0,1.534331727,1.6612860177,-4.8987443626
C,0,0.2923073541,1.3246979306,-4.3487586192
C,0,0.0221610426,1.554662569,-3.0006777895
C,0,1.0258312979,2.1423430884,-2.1916174429
C,0,2.2632948429,2.4937814139,-2.7523319921
C,0,-1.3375678496,1.2385468559,-2.3972083638
C,0,-1.1703423587,0.7938615602,-0.9276368072
C,0,-0.3291682536,1.7949460054,-0.1747514916
C,0,0.7249454304,2.4034308827,-0.7868040048
O,0,-2.1294644292,2.3887511679,-2.4350067833
H,0,3.4881124568,2.5186964401,-4.5315055276
H,0,1.7304328751,1.4695081559,-5.9564917997
H,0,-0.4825496006,0.8778925308,-4.976546829
H,0,3.024893195,2.9423564234,-2.1128001822
H,0,-1.7922642699,0.3983739533,-2.9548096455
H,0,-2.157371586,0.8061333662,-0.4508026047
H,0,-0.7487750125,2.1923236695,0.7409579003
H,0,1.3051910736,3.1708926681,-0.2699325676
C,0,-1.0504528559,-1.0574625461,2.6203624377
H,0,-1.2809150373,-1.2815307051,3.6772058683
H,0,-1.9059709959,-0.5087089931,2.2019719278
H,0,-0.959804101,-2.0182873908,2.1015190311
C,0,1.9778018504,-2.2003603287,2.50699535
H,0,2.3272326174,-2.4822994772,3.515410739
H,0,1.1515901024,-2.8661868036,2.2330532178
H,0,2.7989482634,-2.3584112479,1.796332076
C,0,-0.8790093793,2.0493260723,2.9791254294
H,0,-1.800824791,1.7239111013,2.4674750202
H,0,-1.0741708381,2.0248705045,4.0662506682
H,0,-0.6670483657,3.0949373314,2.7145774917
C,0,2.2112713748,2.9322127929,2.7142239649
H,0,1.4592091448,3.6763941229,2.4172963036
H,0,2.5372914924,3.1799895503,3.7389690084
H,0,3.0800305022,3.041836219,2.049077749
C,0,3.9348910929,0.2782338299,2.7308841783
H,0,4.2052140966,0.2849482085,3.801473918
H,0,4.338414751,-0.6293268191,2.2699999209
H,0,4.4131988469,1.1476554725,2.261089287
C,0,-5.4560490694,1.9141209815,1.3511947231
H,0,-5.8398351435,0.9892726538,1.8107461491
H,0,-6.2270395902,2.3695454008,0.7152837524
H,0,-5.2119024558,2.6015715871,2.1774085003
```

C,0,-4.1836208369,1.6029855463,0.5545686115
O,0,-3.2715868281,0.9803593301,1.1713893609
O,0,-4.1467254617,1.9700247736,-0.6462667494
H,0,-2.8994880269,2.2458484392,-1.820785975
C,0,4.9677672718,0.6995679698,-1.2109848087
H,0,5.8558440704,0.0576499328,-1.148070003
H,0,5.2054421869,1.7244978416,-0.8926940093
H,0,4.6367995239,0.7460706602,-2.2609440271
C,0,3.8414951765,0.1108810222,-0.3751728575
O,0,3.8403655778,-1.0746703778,-0.0583196534
O,0,2.914012265,0.971346606,-0.0762818698

***trans*-3aa**
Opt @ B3LYP-D3(BJ)/def2-SVP in gas phase
SCF Done: E(RB3LYP) = -1087.36514772 a.u.
Zero-point correction = 0.342391 Hartree/Particle
Sum of electronic and thermal Free Energies = -1087.072957 a.u.
Sp @ RI-PWPB95-D3(BJ)/def2-TZVPP in 2,2,2-trifluoroethanol
FINAL SINGLE POINT ENERGY = -1087.694791436100 a.u.

C,0,0.9849152745,-1.9636549345,-2.504430728
C,0,-0.4095866381,-1.6952655148,-2.5463093765
C,0,-1.1612389288,-1.8662289365,-3.7137020282
C,0,-0.4918258157,-2.3187722906,-4.8496841318
C,0,0.8900324672,-2.5956213047,-4.8273740878
C,0,1.6337336704,-2.4218571564,-3.6640933538
C,0,1.4185226995,-1.6838355877,-1.162480855
C,0,0.3402003928,-1.237257655,-0.4389686766
H,0,-2.2332161562,-1.6762577316,-3.7242161539
H,0,-1.0543124782,-2.4687131037,-5.7742905735
H,0,1.3801099942,-2.9522628288,-5.7364044453
H,0,2.7055100856,-2.6340485127,-3.6484956461
H,0,2.4155518007,-1.8422766126,-0.7555454909
N,0,-0.7870010257,-1.2310142974,-1.2796450563
C,0,-2.0608602669,-0.7222004471,-0.9740105817
C,0,-4.3146913161,-0.96486953,-1.0536823731
C,0,-3.3102407263,0.9130386814,-0.0180387179
C,0,-4.4798211925,0.2304495967,-0.3510040264
H,0,-5.1794101653,-1.558156831,-1.3719914953
H,0,-3.3406835121,1.8621889471,0.5256157907
H,0,-5.4656261088,0.6104337602,-0.0818817332
N,0,-3.1124952302,-1.4515755977,-1.3600880754
N,0,-2.1045963383,0.4480442227,-0.3417245164
C,0,-4.0071665147,0.3925229021,3.5653151681
C,0,-4.2157553442,-0.929009055,3.1613372507
C,0,-3.1723852699,-1.6398233738,2.5599997563
C,0,-1.9309439165,-1.0422580495,2.3430978747
C,0,-1.7240560578,0.3004682085,2.721873011
C,0,-2.7701839027,1.000260022,3.344737961
C,0,-0.785181031,-1.858095973,1.7896766894
C,0,0.3122569087,-1.0244550412,1.0570683529
C,0,0.4175996572,0.4032803976,1.5343254859
C,0,-0.4781103055,0.9696720411,2.3548600426
O,0,-0.2272315882,-2.5421771348,2.9075446637
H,0,-4.8124171208,0.9531628523,4.0464561314

```
H,0,-5.1837179439,-1.4088940123,3.3229882332
H,0,-3.3229920326,-2.6791207142,2.2563571083
H,0,-2.6105230996,2.0409385008,3.6399333192
H,0,-1.1964203693,-2.5931876034,1.073638483
H,0,1.2551486796,-1.4941989643,1.3866126048
H,0,1.2979726028,0.9595595509,1.201424034
H,0,-0.334513411,1.9973252028,2.7000460682
H,0,0.3744955739,-3.2179260921,2.566282094
----------------------------------------------------------------
```
